# Complex trans-ridge normal faults controlling large earthquakes

**DOI:** 10.1038/s41598-022-14406-4

**Published:** 2022-06-23

**Authors:** Simone Bello, Giusy Lavecchia, Carlo Andrenacci, Maurizio Ercoli, Daniele Cirillo, Filippo Carboni, Massimiliano R. Barchi, Francesco Brozzetti

**Affiliations:** 1 DiSPuTer, University G. d’Annunzio, via dei Vestini 31, 66100 Chieti, Italy; 2 CRUST - Centro inteRUniversitario per l’analisi Sismotettonica Tridimensionale, Chieti, Italy; 3 Dipartimento Di Fisica E Geologia, Università Degli Studi Di Perugia, Perugia, Italy

**Keywords:** Natural hazards, Solid Earth sciences, Geology, Geophysics, Tectonics

## Abstract

Studying faults capable of releasing moderate-to-strong earthquakes is fundamental for seismic hazard studies, especially in a territory that was subject to the strongest peninsular Italy earthquake (1857, M_w_ 7.1) and hosting the largest European oil field on-land. Fieldwork-based observations in the Campania-Lucania area highlight a SSW-dipping ~ 65 km-long normal-oblique-segmented fault, showing evidence of recent activity and possibly responsible for the 1857 earthquake. It crosses the Maddalena ridge, linking separate Quaternary basins. Two seismic reflection profiles cross the fault trace where it is buried beneath the Val d’Agri Quaternary deposits. Similarities between fault-controlled small basins in the highest portion of the massifs in the study area and the neighboring 1980 Irpinia area (1980 earthquake, M_w_ 6.9) are interpreted as evidence of trans-ridge fault activity. Kinematic analyses and the stress field inversion provide a N032-trending near-horizontal s3-axis, the same computed in literature for the Irpinia area, highlighting a deviation from the ~N045-axis which characterizes most of the Apennines. This study demonstrates how detailed fieldwork, supported by geophysics and innovative data analysis techniques, can unravel unknown faults while giving a novel interpretation of the trans-ridge faults' style in controlling strong earthquakes, moving away from classical interpretations, and providing a helpful approach in similar contexts worldwide.

## Introduction

Highlighting unknown active faults (*i.e.*, late Quaternary in age) and determining the interactions with already known and mapped faults, lays the foundations for seismic hazard studies, assessing maximum expected earthquake magnitudes and rupture pattern behaviors occurring during strong earthquakes. This type of study consequently influences urban and infrastructure planning (*e.g.*,^1,2^). In this regard, an important contribution from geoscientists lays in the interpretation of the geometric and structural segmentation of active and potentially seismogenic faults. In most cases, the segmentation is defined based on the geometric relationships between neighboring and interconnected fault segments, measuring the distances and the relationships between their tips or points where a sharp change in fault-strike occurs. For these reasons, a key role is played by surveys and data collection in the field. In this paper, data deriving from remote analysis and fieldwork surveys reveal key clues for better understanding the structural style and its control on the seismotectonic setting of a large portion of the intra-Apennine extensional belt of southern Italy.

**Figure 1:**
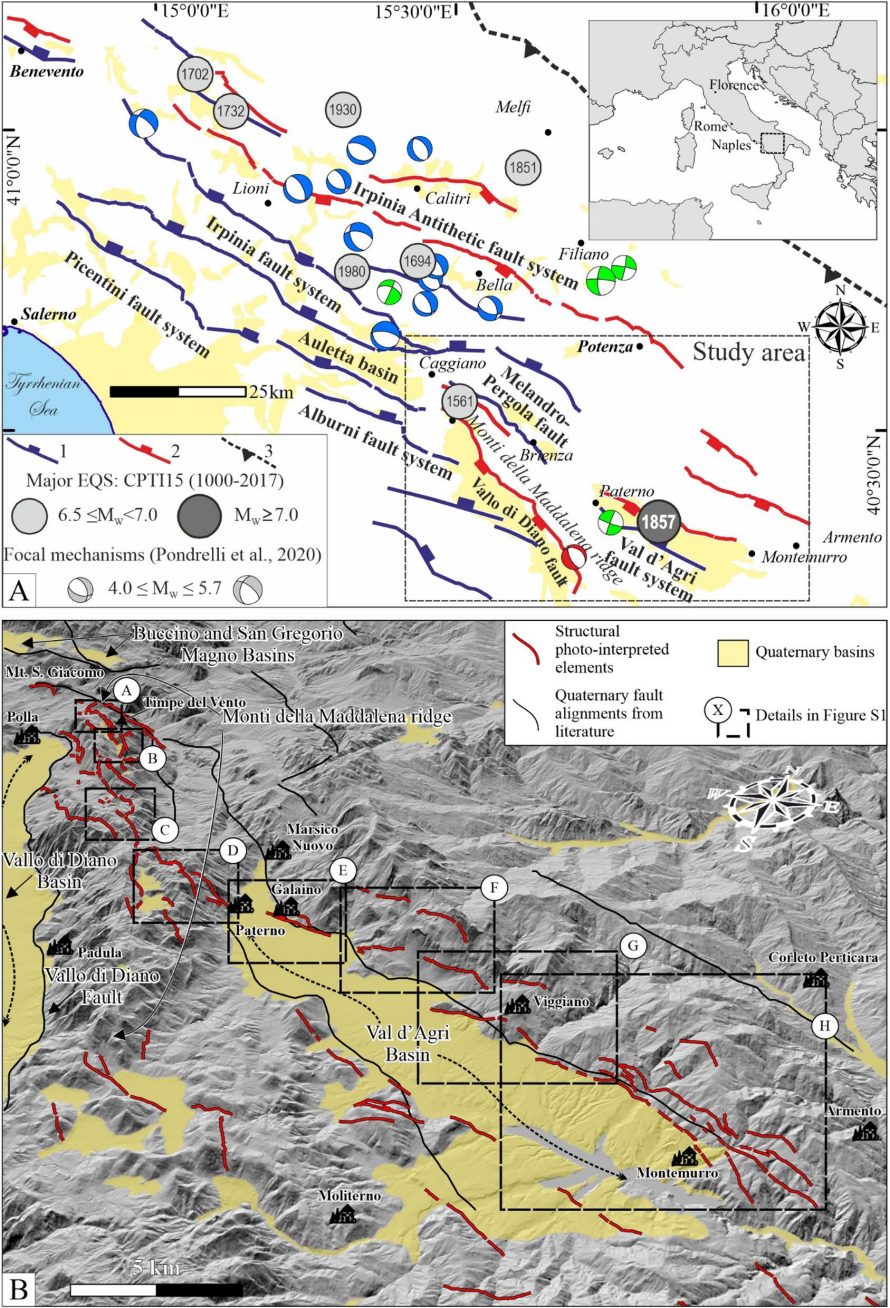
**(A)** Regional structural map and study area. The major normal fault alignments are from Brozzetti et al.^10^ and Bello et al.^9^. Key: 1 =  ~ NE-dipping normal faults; 2 =  ~ SW-dipping normal faults; 3 = thrust faults. Major historical earthquakes reported as grey circles are from the Parametric Catalogue of Italian Earthquakes, CPTI15 v4.0^5,6^. Earthquake focal mechanisms (0–15 km; 4.3 ≤ M_w_ ≤ 6.8; 1962–2002) are from Pondrelli et al.^4^, divided into blue (normal) and green (strike-slip). The red focal mechanism is from López-Comino et al.^11^. Yellow areas represent Quaternary deposits. **(B)** Bird's-eye view map of the photo-interpreted elements. Background is a 10 m hillshaded-DEM^12^. Red lines are the structural elements observed during the preliminary phase of photointerpretation, while black lines are faults from literature. Yellow areas represent Quaternary deposits in-fill. Dashed squares are the details reported in the Figure S1 of the Supplementary Material. Black dotted arrows indicate the Vallo di Diano and Val d’Agri basins.

The study area (from the Auletta to Val d’Agri basins) and its surroundings (Fig. 1) correspond to one of the most seismically active areas in Italy^3,4^. In historical and early instrumental times, numerous destructive earthquakes^5,6^ (M_w_ 6.5–7; Fig. 1A) caused tens of thousands of victims. However, even if the seismogenic sources have been intensely studied^3^, there are still many open questions regarding their geometries (*i.e.*, strike, dip direction, and dip), their sense of slip, and size. An example is given by the sources of the Campania-Lucania 1980 earthquake (also Irpinia earthquake; M_w_ 6.9), of which source models and location of the mainshocks^7–9^ have been long debated.

Several unanswered questions within the study area (Fig. 1A) concern the 1857 Basilicata earthquake^13^ (I_max_ = XI MCS; M_w_ 7.1), one of the strongest Italian earthquakes, with effects widely felt throughout the southern portion of the country. The damage produced was firstly reported in a document by Robert Mallet^13^, who was sent on reconnaissance by the Royal Society of London in the years immediately following the earthquake. The earthquake had two sub-events in a few minutes^13,14^, generating devastation and victims between the Vallo di Diano and the Val d’Agri, in a large area of southern Italy (Fig. 1). The causative fault (one or more seismogenic sources?), the possibility of a hidden source fault, and the lack of evidence of coseismic surface faulting are still matter of scientific debate. Further questions regard the fault segments’ size, depth, and dip direction (NE rather than SW)^15,16^. Therefore, answering these open questions is crucial, due to the study area’s social and economic structure and heritage, and the related seismic and anthropogenic hazard^17–19^. Furthermore, it should be specially considered that the Val d’Agri hosts a significant oil treatment center above the largest onshore hydrocarbons field in Europe.

With a structural approach for seismotectonic purposes, this work aims to define the surface geometry, kinematics, stress state and seismic potential of the tectonic structures that possibly controlled the 1857 earthquake and could release other destructive earthquakes. The work proposes and documents a partially unknown, ~65 km-long extensional fault system, which profoundly differs from classical ridge-bounding normal faults systems, widely studied in intermountain seismic belts such as the Basin and Range province of the USA and the Apennines of Italy (*e.g.*,^20–23^). Instead, it is a “trans-ridge” fault that crosses an intermediate mountain ridge (Monti della Maddalena) and connects along-strike two late Pleistocene-Holocene basins (Fig. 1) and several minor en-échelon ones. This fault, organized in multi-scale segments, sections and sub-sections, is referred to as Caggiano-Montemurro fault (CMF). It develops in an average WNW-ESE direction, transversal to the near N–S trend of the neighboring Quaternary Vallo di Diano basin, the largest intramountain basin of southern Italy.

The new mapped fault would directly impact the 1857 fault rupture propagation model and the active fault pattern of southern Italy, in general, representing a significant challenge for a more complete worldwide understanding of active processes and seismic potential. Geologists commonly limit the search for active seismogenic faults along the major basinal depressions classically bound by associated normal faults. However, the strong 1980 Irpinia earthquake had already proven that faults capable of generating strong earthquakes can run for long distances in the highest portions of the massifs, generating small intramountain basins (*i.e.*, a few tens or hundreds of meters) elongated along fault-strike (*i.e.*, the Melara or the Piano di Pecore basins along the Irpinia fault^7,9,24^).

Considering the large extent of the investigated area (∼900 km^2^), our workflow started with an initial phase of satellite images interpretation, delineating an alignment of morphostructural elements, which we considered clues to guide a series of geological-structural field surveys. Many structural data were subsequently acquired in the field and represent the basis of the following interpretations. The ~65 km-long SSW-dipping trans-ridge fault is identified as an alignment of relatively continuous fresh scarps, bounding several en-échelon narrow intramountain basins filled by late Quaternary clastic deposits. These fault-controlled elongated basins cross the topographically highest sector of the Monti della Maddalena ridge, giving rise to a peculiar “*plain-in ridges*” landscape.

The CMF cross-cuts the Val d’Agri basin and disappears beneath anthropized continental deposits in its central portion. To constrain the structures in this key-area, we have operated a seismic interpretation of two commercial reflection profiles, whose quality and interpretability were previously improved using pre-conditioning filters and seismic attribute analysis^25,26^. Next, we defined the segmentation and parameterized the entire fault following globally applied segmentation criteria^27–29^ and distinguished three segmentation orders (*i.e.*, segments, sections, and sub-sections). Finally, we elaborated the structural data for a kinematics analysis and a formal inversion of the stress field to test the structure’s compatibility with the current stress field^9,30,31^. Only a minimal amount of structural data was available in the literature and almost exclusively limited to the northernmost portion of the structure^32–34^. Thanks to our new dataset, we improve and constrain the structures in their entire order of segmentation, highlighting a previously not considered direct connection among the WSW-dipping tectonic structures of the Auletta basin, Monti della Maddalena ridge and Val d’Agri basin.

## Background

### Geological setting

The study area is located in the southern Campania and Basilicata regions, in the axial sector of the southern Apennine fold-and-thrust belt. This mountain chain, which originated from a late Miocene—early Pliocene contractional deformation, was subsequently displaced by Quaternary extensional structures^10^. The latter are located within the extensional seismotectonic province (*sensu*^35^) of the Italian Apennines, which extends throughout the peninsula^36^, bounded by high-angle westward-dipping and low-angle eastward-dipping normal faults^10,36–40^. These faults dislocate the pre-existing late-Miocene early-Pliocene compressional structures and develop simultaneously with the eastward-verging compressional structures at the outer front of the Apennine thrust belt (*e.g.*,^41^ among others). Within the Adriatic foreland, near-vertical E-W striking faults are revealed by focal mechanisms (*e.g.*,^42^).

According to the most recent literature, the extensional strain field that generated these fault alignments has lasted since the very early Pleistocene^32,43–45^. Low-to-moderate slip rates (0.2–2 mm/year) are documented on most of the active faults during the late Pleistocene–Holocene^32,33,46,47^. However, other authors refer to early Pleistocene times the onset of the extensional activity currently dissecting the orogenic pile^37,48^. The latter was described in detail by the synthesis of Mostardini and Merlini^49^, which, based on the integration of geophysical and geological datasets, recognized several major superimposed tectonic units piled up according to a thin-tectonic style. From the uppermost to the lowermost, they are referred as: basinal Liguride and Sicilide (Middle Jurassic–Early Miocene), Apennine Carbonate Platform (Late Triassic—Middle Miocene), pelagic Lagonegro and Sannio-Molise (Middle Triassic—Early Cretaceous pelagites and Burdigalian–Messinian flysch deposits), and Apulian Carbonate Platform (Late Triassic—Early Pliocene). According to biostratigraphic revisions of several well-logs^50^, the orogenic deformation involved the internal paleogeographic domain during the Early Miocene, the Apennine Platform during the Serravallian–Tortonian interval, the Lagonegro–Sannio–Molise basin in the late Tortonian, and the Apulian Platform during the early Pliocene^10^.

In the study area, the major extensional structures are two large intramountain Quaternary basins, the Vallo di Diano and the Val d’Agri, NNW-SSE- and WNW-ESE-trending, respectively. They are separated by the Monti della Maddalena ridge (Fig. 1B). These basins are filled by syntectonic continental fluvio-lacustrine deposits, alluvial fans and slope debris, characterized by north-eastward thickening, tilted beds^51,52^. They belong to a regional-scale NW-SE-trending extensional belt including the Picentini, Irpinia, Alburni-Tanagro fault systems to the north, and the Mercure, Campotenese, Castrovillari and Crati fault systems to the south^7,9,10,24,31,33,34,43,44,53–56^ (Fig. 1A). The origin of the basins is debated: they may be the result of post-collisional extension, post-date an intermediate strike-slip phase^57–59^, or simply represent intra-continental extension^10,44^.

### Historical earthquakes

In historical times, the active faults of this portion of the southern Apennines released eight destructive earthquakes^6^ (Fig. 1A). Seven of them had M_w_ between 6.5 and 7.0, increasing macroseismic intensity of up to X-XI MCS. The Basilicata 1857 event had M_w_ 7.1 (XI MCS), and it is classified among the strongest earthquakes in peninsular Italy.

Moving southward along the main fault alignments of the area (see Fig. 1A), the 14 March 1702 (Sannio-Irpinia, M_w_ 6.5) and 29 November 1732 (Irpinia, M_w_ 6.6) events, located about 25 km east of Benevento, were both associated with a high-angle NE-dipping seismogenic structure^3^. In a more external position (*i.e.*, eastward), the 23 July 1930 earthquake (Irpinia, M_w_ 6.7) has been referred to a deep, blind WNW-ESE trending normal fault system^3^. The 14 August 1851 event (Basilicata, 6.3 ≤ M_w_ < 6.5) is poorly known and has been associated with a right-lateral strike-slip fault, similar to the structures activated during the San Giuliano (31 October–1 November 2002, M_w_ 6.0) and Potenza (5 May 1990–26 May 1991, M_w_ 5.8 and 5.2, respectively) seismic sequences^3,60^.

The 23 November 1980 Campania–Lucania multiple earthquake (M_w_ 6.9) was caused by the Irpinia east-dipping fault and its antithetic fault alignment^9^ (Fig. 1A). Three in-sequence sub-events were released in a time-lapse of 40 s, activating the fault system in a "graben-like" model^8,9^. The 8 September 1694 earthquake is considered a twin of the 1980 event; however, it may have activated either the west- or the east-dipping 1980 fault alignment^3,8,61^.

The 19 August 1561 (Vallo di Diano, 6.4 ≤ M_w_ < 6.5) earthquake was located at the northern tip of the Vallo di Diano basin. Despite the poorly constrained location of this seismic event, most of the authors associate it with the WSW-dipping Caggiano fault (Fig. 1A), which crops out in this area^46,62^.

The southernmost event is also the most significant in the area. The 16 December 1857 (Basilicata, M_w_ 7.1) earthquake was felt in the entire central and southern Italy, causing 10,000 to 19,000 casualties. In the only municipality of Montemurro (Fig. 1), it caused ~ 4000 deaths out of a population of ~ 7000 inhabitants^13,14^. In addition, two major sub-events were released in a time-lapse of ~ 3 min, either rupturing two east-dipping faults (Melandro-Pergola and Val d’Agri; Fig. 1A) or the west-dipping Val d’Agri fault alignment^3,15,16^. Whatever the dip-direction of the causative fault, its estimated magnitude suggests the activation of one or more seismogenic sources for several tens of km. Moreover, the seismogenic fault trend and extent must be suitable to justify the configuration of the macroseismic intensity field^5^ that includes the northern sector of the Monti della Maddalena ridge, and the reliefs located east of the Val d’Agri (Fig. 1).

## Methods

### Preliminary phases and field activities

For this study, we implemented a workflow initially based on the set-up of a GIS project (ESRI ArcMap/ArcGIS®). First, we reported the traces of the main faults from the literature and the geological formations as they are mapped in the most up-to-date available cartography (1:100,000 and 1:50,000 scale sheets of the Carta Geologica d’Italia; https://www.isprambiente.gov.it).

To enhance and constrain the geomorphological elements of possible tectonic meaning, we processed a 10 m-resolution DEM^12^ to obtain an hillshaded relief image artificially illuminated from the best direction and incidence angle. Moreover, for the areas falling within the Basilicata region, the availability of a 1:5.000 scale digital topography (https://rsdi.regione.basilicata.it/Catalogo/srv/ita/search?hl=ita#) allowed us to elaborate a 5 m-resolution DTM, that we further processed on ArcMap© to obtain a hillshade.

We traced all the observable alignments interpreted as structural-geological elements, supported by satellite images, and locally integrated with aerial frames. Our observations aimed to identify: (i) continuous linear scarps of possible tectonic origin, highlighted by abrupt variations in the steepness of the slopes; (ii) tectonic contacts showing fresh geomorphic signature displacing both bedrock and Quaternary stratigraphic units; (iii) depressions filled by late Quaternary deposits, in particular, those elongated nearly sub-parallel to the tectonic contacts and above-mentioned scarps; (iv) geometry of the fans spreading from the slopes towards the flat areas, mainly where any apparent topographic anomalies affect their topographic surfaces.

We obtained a map of the photo-interpreted elements from which we extracted the lineaments of Fig. 1B. Each of the rectangles contains alignments of structural elements shown in detail in the Supplementary Material (Fig. S1). The lineaments highlighted show substantial continuity and have guided the subsequent geological-structural survey campaigns. We imported these geo-referenced elements as lines in a project created using the FieldMove app (PetEx Ltd., version 2019.1) running on an iPad Pro tablet.

We made extensive field observations during four campaigns between 2020 and 2022, covering an area of ~900 km^2^ along fault segments prevailingly arranged in a right en-échelon pattern, for a total of about 90 linear kilometers (details in the Results section). Direct field observations were aimed to verify and validate previously mapped morpho-structural evidence of faulting and acquiring new fault/slip data. We focused on the structures outlined in the preliminary phase while enlarging the westward and eastward areas to constrain, with punctual data, the structures already known from the literature. We also aimed to understand the structural relationships between the traced elements and the faults outcropping in their surroundings. We acquired ~370 measurements, available in Supplementary Data 2. For quality control, during the field campaigns we assigned each outcrop and structural site (hereinafter referred to as SS) a quality parameter named OQR (*i.e.*, outcrop quality ranking, defined as in^9^). It was aimed to describe the preservation of the outcrop and attest the reliability of the data acquired on site. OQR is a 1-to-4 score with 1 the best and 4 the less reliable, reported in Supplementary Data 2. In addition, each outcrop is documented with georeferenced photographs to increase quality control and research reproducibility (Supplementary Data 1).

**Figure 2:**
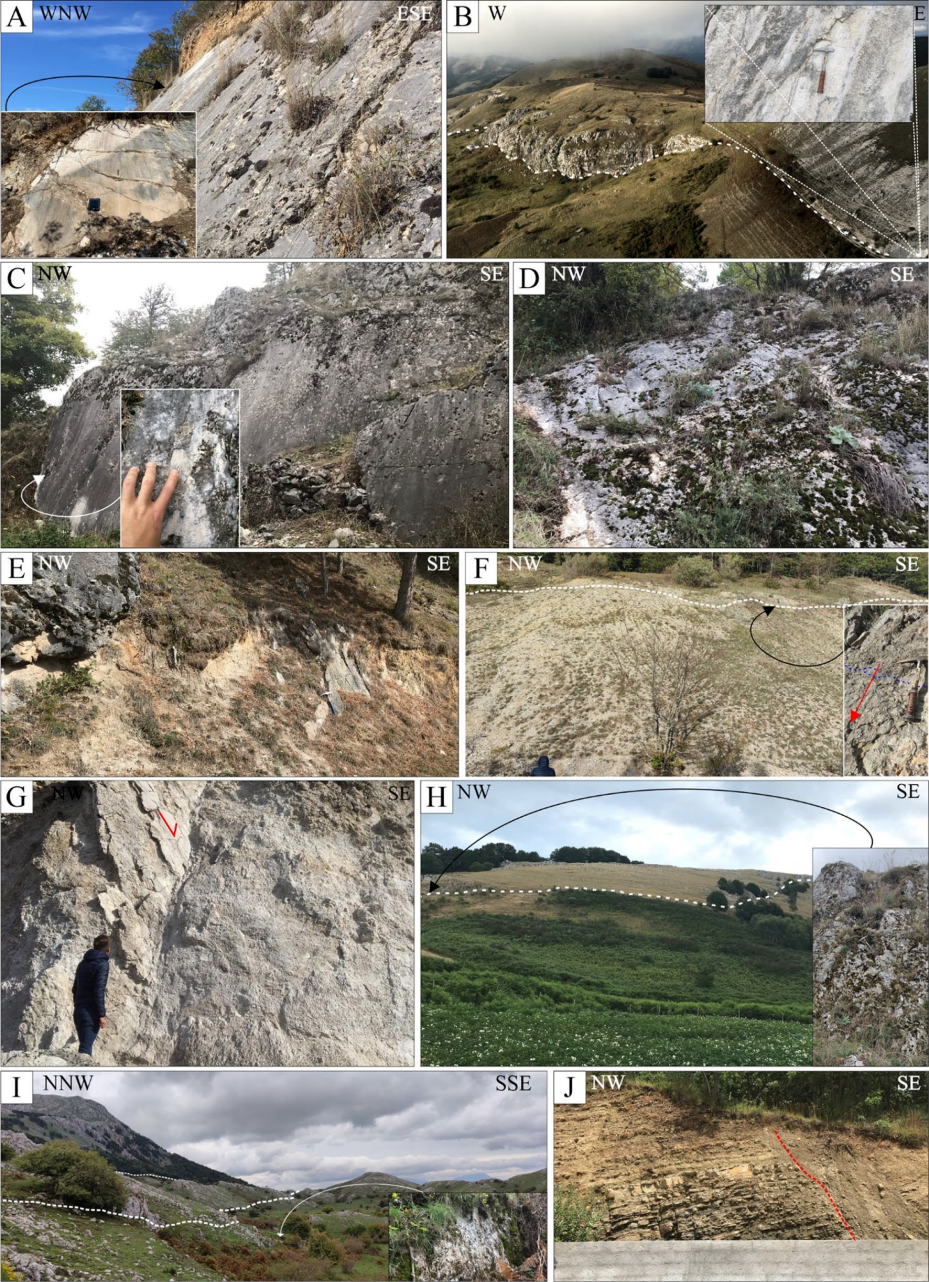
Photographic documentation of faulting along the fault alignments of Fig. 1A (see Supplementary Data 1 for a complete geo-referenced photographic documentation from the fieldwork). **(A)** Striated fault plane near Polla, looking NNE. **(B)** Panoramic view (looking north) of the Timpe fault^46^ with detail of slickenlines on the polished slip surface. **(C)**, **(D),** Striated fault planes along the southern prosecution of the Timpe fault, and (**E**) on the en-échelon strand developing SE of Atena Lucana. (**E)** Displaced deposits close to Marsicovetere **(F)** fault plane cropping out close to Marsicovetere and detail of normal striations, looking NE. **(G)** Striated normal fault plane close to Marsicovetere, looking north. **(H)** Fault scarp (white dotted line) and associated striated breccia cropping out at the edge of a small intramountain plane, NW of Paterno, looking NE. **(I)** boundary fault of a small basin, NE of Marsicovetere, looking WNW. **(J)** Displaced beds of the Gorgoglione Flysch along a SW-dipping normal fault between the Montemurro and Armento villages, looking NE.

We managed the acquired structural data in ArcGIS v.10.8 (ArcMap©), and we used both the FaultKin8.1 and Stereonet software^63,64^ to analyze their kinematics. We show in Figure 2 some photographic examples of SS (see Supplementary Data 1 for complete photographic documentation). During the fieldwork, we paid particular attention to identifying evidence of Quaternary activity, and in particular to finding faulting of recent deposits (*i.e.*, late Quaternary).

### Seismic reflection profiles

In the area where the CMF crosses the Val d’Agri basin, (Fig. 1B–area e; Fig. S1e), the pervasive agricultural anthropization and the sedimentation rates of the recent deposits (late Pleistocene–Holocene), higher than the faulting rates, hide any residual morphological evidence of past surface faulting. For this reason, we used two seismic lines to identify and constrain the tectonic structures affecting the geological substrate below the recent continental deposits. We interpreted two time-migrated seismic reflection profiles (hereinafter referred to as VA1 and VA2) collected during the ’80-’90 for hydrocarbon exploration by ENI (Italian Energy company) and kindly provided us for research purposes, under a confidential agreement. The seismic profiles have a moderate signal-to-noise ratio and vertical resolution, the latter estimated to be ca. 80 m (assuming an average velocity of 5000 m/s and a dominant frequency of 15 Hz). Profile VA1, WSW–ENE oriented, has an average spacing between the CDP of 12.5 m and a total length of 15 km. Profile VA2, NNW–SSE oriented, has an average spacing between the CDP of 20 m, and a total length of 14.8 km.

The quality of the profiles was improved to aid the detection of tectonic structures (*e.g.*,^26,65^). After a preliminary spectral analysis, they were pre-conditioned using OpendTtect Software (https://www.dgbes.com/) to reduce the high-frequency (random) noise, which is particularly visible in the first second (TWT). For this purpose, two different pre-conditioning strategies were used: (i) apply a conventional low-pass FFT filter (cut-off frequency of 35 Hz, 5% cosine taper up to 45 Hz) to remove the random noise, and (ii) apply a low-pass convolve filter (arithmetic averaging filter, size 5) which operated a further smoothing. In both cases, the overall quality of the profiles and imaging of the overall reflections were improved, with benefits in the display of the main sub-vertical discontinuities (*e.g.*, tectonic structures), except for a few high-frequency reflections occurring at shallow depth, which slightly reduced their original resolution (for comparison see the conventional migrated profiles in Supplementary Material Fig. S2).

However, this pre-conditioning strategy was fundamental to improve the computation of the seismic attributes^25,26,65–67^ which, on the contrary, are affected by noise-generated artifacts whether generated from original noisy data. Among several attributes tested, we decided to use the Pseudo Relief attribute (PR, see^25^), which generates an “outcrop-like” display^67^, extremely useful for structural interpretation. The final results (shown in the Results section) demonstrate how this workflow guaranteed a remarkable improvement in the quality and interpretability of the seismic profiles, particularly for faults detection.

### Stress field inversion

Following Delvaux and Sperner^68^ and Ferrarini et al.^30^, we inverted 137 structural-geological fault/striation pairs considering as model parameters the orientation of the three principal axes of the stress (σ1, σ2, σ3) and the stress ratio R = (σ2 − σ3)/(σ1 − σ3). The inversion highlights a normal faulting stress regime with a nearly horizontal NNE-SSW trending σ3-axis (212/10) and a sub-vertical σ1-axis attitude (041/80), as well as a shape factor of 0.39, indicating a near triaxial tensor. The quality rankings^69,70^ for the stress orientations solutions are QRw = C, QRt = B. The formal inversion shows that 86% (n/nt) of the fault/striation pairs are consistent with the computed stress tensor. The results of the stress field inversion are shown in the Results section.

### 1857 earthquake macroseismic field

The 16 December 1857 Basilicata earthquake (M_w_ 7.1) is one of the most destructive seismic events ever recorded in peninsular Italy which has been widely felt throughout the southern portion of the country. Most of the devastation and victims were in a large area between the Vallo di Diano (Campania) and the Val d’Agri (Basilicata).

Starting from 314 intensity points (Mercalli-Cancani Sieberg Scale) given by the Italian Macroseismic Database (DBMI15^71^) for the municipalities of central-southern Italy struck by the earthquake, and using the Natural Neighbor interpolation method in ArcMap©, we represented the macroseismic field as isoseismals lines (*i.e.*, lines of equal intensity)^72^. Averaging and smoothing the intensity pattern helps reduce the impact of local-scale effects, thus helping to highlight more regular distribution and making clearer dimensions and prevailing directions of the felt areas. It may be compared with the CMF trend and extent and used to speculate on the possible fault/earthquake relationship (see next sections). Further details regarding the analysis have been reported in the supplementary material reports further details regarding the macroseismic field analysis (see also Fig. S3).

## Results

### Kinematics and segmentation pattern of the fault system

**Figure 3:**
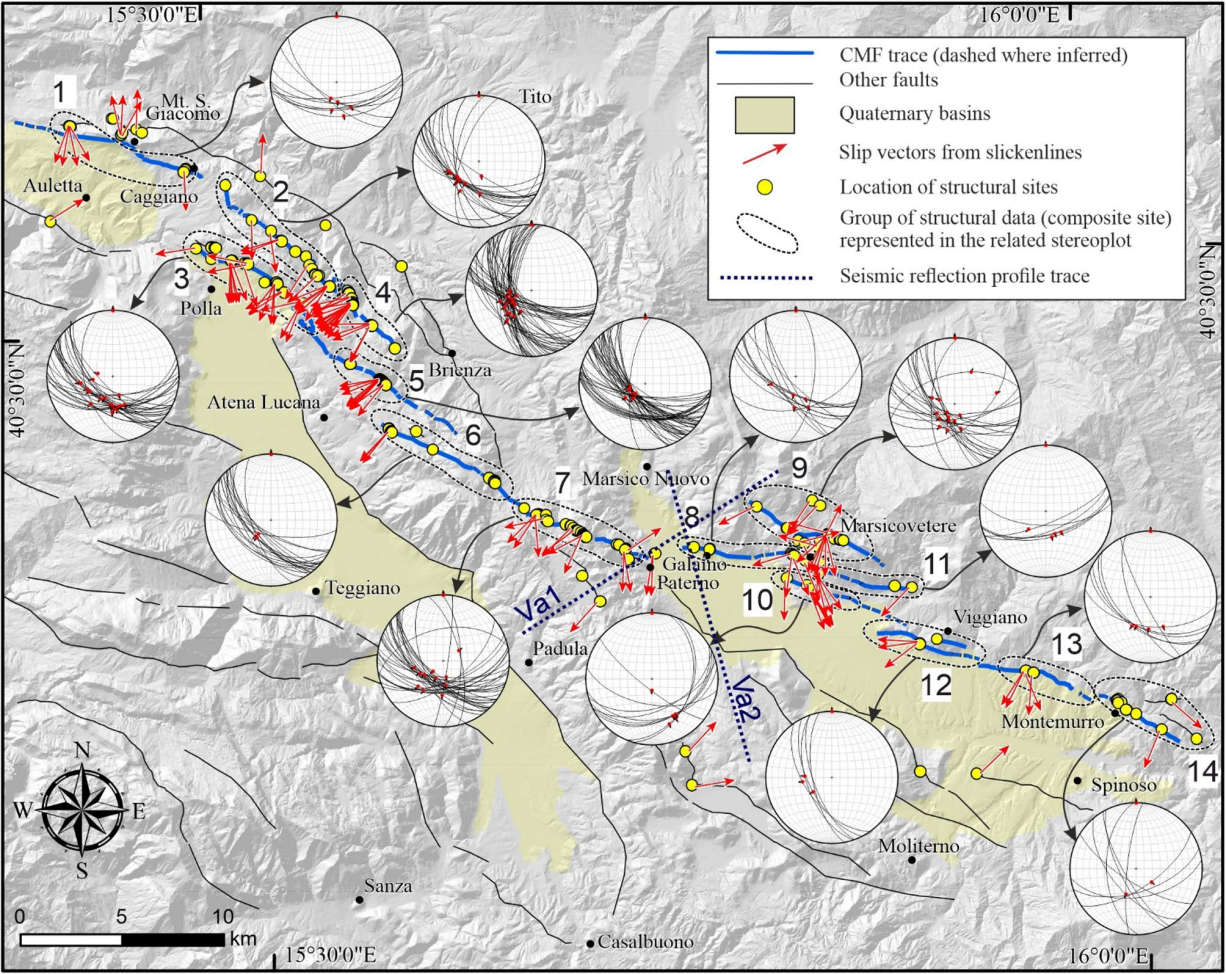
Structural map with survey sites (yellow circles) and slip vectors (red arrows). Location of the study area is in Fig. 1A. Stereoplots refer to groups of sites, delimited in the map by black dashed lines. Dark blue dotted lines are the trace of the two seismic reflection profiles interpreted in this paper.

We integrated the ~370 field data (fault/slip attitude and corresponding fault trace) acquired with fault traces from literature to produce a detailed structural map of the regional-scale CMF (Fig. 3). The data of each SS, including geographic coordinates and administrative location, planar and linear attitude (*i.e.*, strike and dip of the fault plane and trend and plunge of the slip vector) plus outcrop quality ranking (OQR) codes have been tabulated and are provided in the Supplementary Material as .txt (Supplementary Data 2).

We grouped the individual SS into fourteen composite sites lying on continuous and homogeneous CMF portions. For each composite site, we calculated the average fault plane (strike and dip), the average slip vector (trend and plunge), the rake in Aki-Richard's format, and the characteristics of the maximum tension axis (trend and plunge). The slickensides belonging to each composite site were plotted on lower hemisphere stereographic nets using FaultKin8.1 software^63,64^ and reported on the structural map of Fig. 3. For a more immediate representation of the average kinematics at each composite site, we generated the pseudo-focal mechanisms (PFM) shown in Table 1.

**Table 1:** Pseudo focal mechanisms.

Site n	PFM	Data n	F/S n	Sec	Mean plane strike* [deg.]	Mean plane dip* [deg.]	Mean slip vector trend* [deg.]	Mean slip vector plunge* [deg.]	Rake [A–R]	T-trend [deg.]	T-plunge [deg.]
1		14	5	CFS	095.5	60.6	174.4	60.1	−95.5	181.5	15.4
2		32	13	TFS	127	59.1	226.8	58.7	−84.9	220.7	13.9
3		49	20	PFS	114.3	57.7	202.3	57.7	−91	203.5	12.7
4		51	23	TFS	143.5	58	238.2	57.9	−87.5	235.4	12.9
5		48	11	PFS	126	69.6	231.7	68.8	−84.4	220.4	24.4
6		20	3	APFS	137.1	63.7	221.5	63.5	−92.5	225.3	18.6
7		35	11	APFS	105.4	59.8	226.5	55.8	−73.1	207.6	13.2
8		14	5	MVFS	129.2	64.5	190.1	61.4	−103.4	209.2	18.4
9		22	14	MVFS	138.2	68.4	217	68.1	−94.1	225	23.3
10		10	5	VIFS	098.4	48.5	159.6	44.8	−110	174.4	01.7
11		6	4	MVFS	087.3	65.4	159.8	64.3	−97.5	171.7	20
12		5	3	MOFS	148.7	58.2	255.8	57.0	−80.8	245.3	12.8
13		7	4	MOFS	116.6	50.4	192.4	49.5	−99.2	200.1	05.0
14		9	4	MOFS	099.1	60.4	190.9	60.4	−89.1	189.8	15.4

Site numbers correspond to the data group in Fig. 3. PFS = pseudo focal mechanisms computed from structural data. Data N = number of fault planes and slip vector measurements. F/S n = number of measurements used in the pseudo focal mechanism calculation (fault/striation pairs). Sec. = CMF sections (CFS: Caggiano fault section; TFS: Timpe fault section; PFS: Polla fault section; APFS: Atena-Paterno fault section; MVFS: Marsicovetere fault section; VIFS: Viggiano fault section; MOFS: Montemurro fault section). Seg. dip dir. = segment’s dip direction. Rake is in Aki-Richards’s format. T axes (trend/plunge) were computed with the dihedron angle method^73^. *Average attitude calculated from the main synthetic faults.

The SS, which are fairly evenly distributed along-strike, depict a fault with normal dip-slip kinematics, which, however, assumes a progressively higher dextral trans-tensive component towards the SE. The analysis of the T-axes of deformation^73^ which shows substantial consistency with geodetic data^74–76^ (Fig. S4) highlights that the CMF shows a kinematic partitioning which is strongly influenced by its segmentation. Overall, the fault strike is ~N120° between the municipalities of Caggiano and Montemurro, deviating from the ~N160° trend of the Vallo di Diano basin to follow a trend sub-parallel to the Val d’Agri basin.

The field mapping and the punctual structural data analysis allowed us to delineate a highly segmented but substantially continuous tectonic structure that connects the SSW-dipping border fault of the Auletta basin, close to the northern termination of the Vallo di Diano basin, with the eastern Val d’Agri fault system, crossing the Monti della Maddalena ridge (Figs. 1B, 3). The CMF structure shows a well-defined right en-échelon geometric pattern. The geometric and structural complexities identifiable between the fault strands (*e.g.*, gaps, overlaps and underlaps, sudden strike variations), are considered to study the segmentation of the CMF, as they are the most adopted criteria in the literature (see^9,27–29^ and references therein). By doing so, we were able to constrain four orders of fault surface segmentation. In particular, we consider the CMF as a first-order master fault which can be subdivided into two ~30 km-long second-order “segments”, seven ~10–15 km-long third order “sections” and fourteen ~5–7 km-long fourth order “sub-sections”. In addition, we also mapped further minor footwall or hanging wall spays. Based on the number of measurements on each sub-section and the OQR assigned in the field, we assigned a quality ranking to the fault sub-sections reported as FQR (*i.e.*, Fault Quality Ranking, defined as in^9^) in Table 2.

**Table 2:** Summary table of segmentation.

Type	Fault	Segm	Sec	Sub sec	FQR	X start	X end	Y start	Y end	Length	Strike	Dip dir	Dip	Rake
M/S	CMF	CPS	–	–	1	15,41,438	15,43,158	40,59,297	40,59,203	1.5	93	183	60.2	−92
Mo	CMF	CPS	CFS	1	2	15,49,284	15,45,977	40,56,480	40,57,620	10.0	106	196	–	−95.5
Mo	CMF	CPS	TFS	2	1	15,56,774	15,50,725	40,50,938	40,56,388	9.0	127	217	63.6	−84.9
Mo	CMF	CPS	TFS	3	1	15,59,731	15,57,722	40,48,268	40,50,635	5.4	128	218	70.7	−91
MS	CMF	CPS	–	–	2	15,49,971	15,48,693	40,53,213	40,53,797	1.3	115	205	67.2	–
Mo	CMF	CPS	PFS	4	1	15,54,874	15,54,634	40,49,376	40,50,634	11.1	116	206	59.5	−87.5
Mo	CMF	CPS	PFS	5	1	15,62,341	15,56,983	40,44,886	40,47,791	6.9	123	213	64.5	−84.4
MS	CMF	CPS	–	–	1	15,60,073	15,60,901	40,44,822	40,44,392	0.9	117	207	75	–
Mo	CMF	CPS	APFS	6	1	15,66,489	15,58,550	40,40,726	40,45,120	8.6	114	204	63.2	−92.5
Mo	CMF	CPS	APFS	7	1	15,73,201	15,66,410	40,38,312	40,40,666	6.3	112	202	62.8	−73.1
M/S	CMF	CPS	–	–	2	15,71,668	15,72,577	40,38,399	40,38,330	0.8	94	184	–	–
Mo	CMF	PMS	MVFS	8	2	15,79,264	15,75,174	40,37,958	40,38,637	5.1	100	190	68	−103.4
Mo	CMF	PMS	MVFS	9	1	15,85,443	15,83,560	40,36,377	40,37,068	7.5	115	205	66.5	−94.1
MS	CMF	PMS	MVFS	–	1	15,81,353	15,84,033	40,38,592	40,37,795	2.5	107	197	65.5	–
Mo	CMF	PMS	MVFS	10	1	15,86,004	15,79,218	40,37,602	40,40,426	7.5	114	204	69.6	−110
M/S	CMF	PMS	–	–	1	15,82,676	15,83,516	40,40,355	40,39,857	0.9	121	211	66.5	−84.3
Mo	CMF	PMS	VIFS	11	1	15,83,404	15,83,128	40,36,073	40,36,140	6.4	112	202	46.3	−97.5
MS	CMF	PMS	VIFS	–	3	15,89,236	15,90,962	40,33,870	40,33,301	1.7	108	198	66	–
Mo	CMF	PMS	MOFS	12	1	15,90,130	15,89,709	40,33,038	40,33,093	5.0	106	196	60.5	−80.8
Mo	CMF	PMS	MOFS	13	1	15,97,837	15,91,579	40,30,532	40,32,457	5.9	105	195	54.8	−99.2
Mo	CMF	PMS	MOFS	14	3	15,99,703	15,99,854	40,30,038	40,30,200	4.7	119	209	67.2	−89.1
M/S	CMF	PMS	–	–	3	16,01,688	16,03,989	40,29,632	40,28,264	2.6	121	211	73	–

Coordinates and measurements refer to the Sub-section. Type: Mo = Main outcropping; M/S = minor/splay. Segm. = segment. Sec. = section. Sections’ acronyms as in Table 1. Sub sec. = subsection, numbered as in Fig. 6. FQR = Fault quality ranking. X-start, X-end, Y-start, Y-end coordinates of the Sub-section. Dip dir. = dip direction. Rake is in Aki-Richards’ format.

In the northernmost CMF portion (Composite site 1 in Fig. 3) the fault crops out along the entire slope of Mt. S. Giacomo up to Caggiano village (Fig. 3). This fault section, known as the Caggiano fault (CFS), partially develops at the edge of the Auletta basin (Fig. 3). It is ~ESE-WNW-striking with a calculated rake of about −95° and it is already reported in the literature (*e.g.*,^51,77^). Despite this, its south prosecution is debated as some authors connected it with the border fault of the Vallo di Diano (*e.g.*,^33^), while, others, suggest a strike similar to the northern portion, linking it with the fault cropping out at the "Timpe del Vento" locality (Fig. 1B; Timpe fault section, TFS)^46,78^. The TFS was firstly mapped by Galli et al.^46^ after Cello et al.^78^. From our fieldwork, we hypothesize a connection of the CFS with the TFS, through a ~1.2 km gap. Moreover, our new mapping allowed us to re-locate the TFS southern tip 5 km further south, providing for the whole structure (*i.e.*, CFS plus TFS) a length of ~22 km. TFS shows dip-slip kinematics with a slight sinistral strike-slip component (Composite sites 2 and 4 in Fig. 3; Table 1).

The second main strand of the fault crops out just north of the Polla village (Fig. 3) for ~12 km, ~2 km SW to the TFS along the slope of the Monti della Maddalena ridge (Figs. 1, 3). In this area, the Polla fault section (PFS), and the synthetic TFS, are arranged approximately parallel with an extended ~11 km-long and ~2 km-wide overlap zone in a right en-échelon setting. The PFS we mapped in the field partly fits with a fault mapped by Spina et al.^77^, even if we detailed a more continuous trace characterized by a series of right en-échelon fragments in its northern and central portions, and a prosecution toward the south which makes the structure ~4.5 km longer overall. PFS presents various along-strike bends as summarized in the stereoplots of Fig. 3 (Composite sites 3 and 5; Table 1). The sense of motion of the structure displays a certain variability that confirms an almost pure dip-slip kinematics, locally associated with a slight sinistral strike-slip component (rake −85°).

Southward, the PFS overlaps for ~3 km at a distance of 1.5 km an additional SW-dipping branch arranged in a right en-échelon pattern. We named this segment the "Atena-Paterno fault section" (APFS) as it extends between these two villages, cutting across the Monti della Maddalena ridge. We mapped the APFS in the field for ~15 km, extending the previously mapped fault, recognized only in the westmost part, close to the Vallo di Diano, for a length lower than 2 km, by Cello et al.^78^. Due to its arrangement, the ~N135°-striking APFS connects the Auletta basin and the eastern Val d’Agri, showing short bends striking between ~N090° and ~N160°, with slickenlines recording approximately dip-slip kinematics (rake −85°; Composite sites 5 and 6 in Fig. 3; Table 1).

From the southern tip of APFS, which is the southernmost section of the Caggiano-Paterno segment (CPS) the CMF continues along the northern edge of the Val d’Agri basin, crossing the basin with a left en-échelon step, between the localities of Paterno and Galaino (Fig. 3). This area of surface data gap probably due to anthropic (agriculture) and geological (high sedimentation rate) factors.

On the east side of the Val d’Agri basin, the fault crops out with an approximately E–W trend, bordering the reliefs from Galaino to the slopes of the mount NE of Viggiano. This fault section, named "Marsicovetere" (hereinafter MVFS), is ~12 km long and the acquired data, grouped in the composite sites 8 and 11 (Fig. 3; Table 1), show normal-oblique kinematics with dextral strike-slip component (−98° <rake <−104°). A 2.2 km-long portion of the MVFS southern tip, partly fits with the fault mapped by Benedetti et al.^15^, considered a major active fault possibly involved in the 1857 earthquake. In the footwall of MVFS and parallel to it at a distance of ~2 km, a SW-facing splay show dip-slip kinematics with a slight sinistral strike-slip component, as shown by the structural data of composite site 9 (Fig. 3; Table 1). This splay fault runs along a narrow intramountain depression we identified through photo-geological analysis for a length of ~2 km.

Another SSW-dipping section arranged in a right en-échelon pattern with respect to the MVF, extends up to the valley east of the city of Viggiano (Fig. 3). This fault section, named “Viggiano” (VIFS), crops out over a length of 9 km displaying fault planes with normal kinematics and a discrete dextral strike-slip component (*i.e.*, rake −110°; composite site 10 in Fig. 3; Table 1). However, its trace presents some gaps, probably due to the poorly conservative rock types it crosses, and to the area’s sustained human activity.

The southernmost fault section "Montemurro" (from now on MOFS) extends in a ~ N110° direction from Viggiano to beyond the village of Montemurro (Fig. 3). The overall length of MOFS is almost 9 km and encompasses three en-échelon sub-sections separated by stepovers of a few hundred meters. The major evidence along the MOFS is deduced from satellite images. The tectonic structure affects the topography in many points; unfortunately, the poorly cemented flysch-type lithologies hamper the structure to crop out, as occurs within the carbonate bedrocks. Nevertheless, some outcrops, including striated fault planes, confirm the MOFS location highlighted using remote sensing techniques. From these outcrops, grouped in the composite sites 12, 13, and 14 (Fig. 3; Table 1), we obtain dip-slip kinematics with variable rake values between −81° and −99°. The set of MVFS, VIFS and MOFS represents the Paterno-Montemurro segment (PMS).

### Constraints from seismic reflection profiles across the Val d’Agri basin

**Figure 4:**
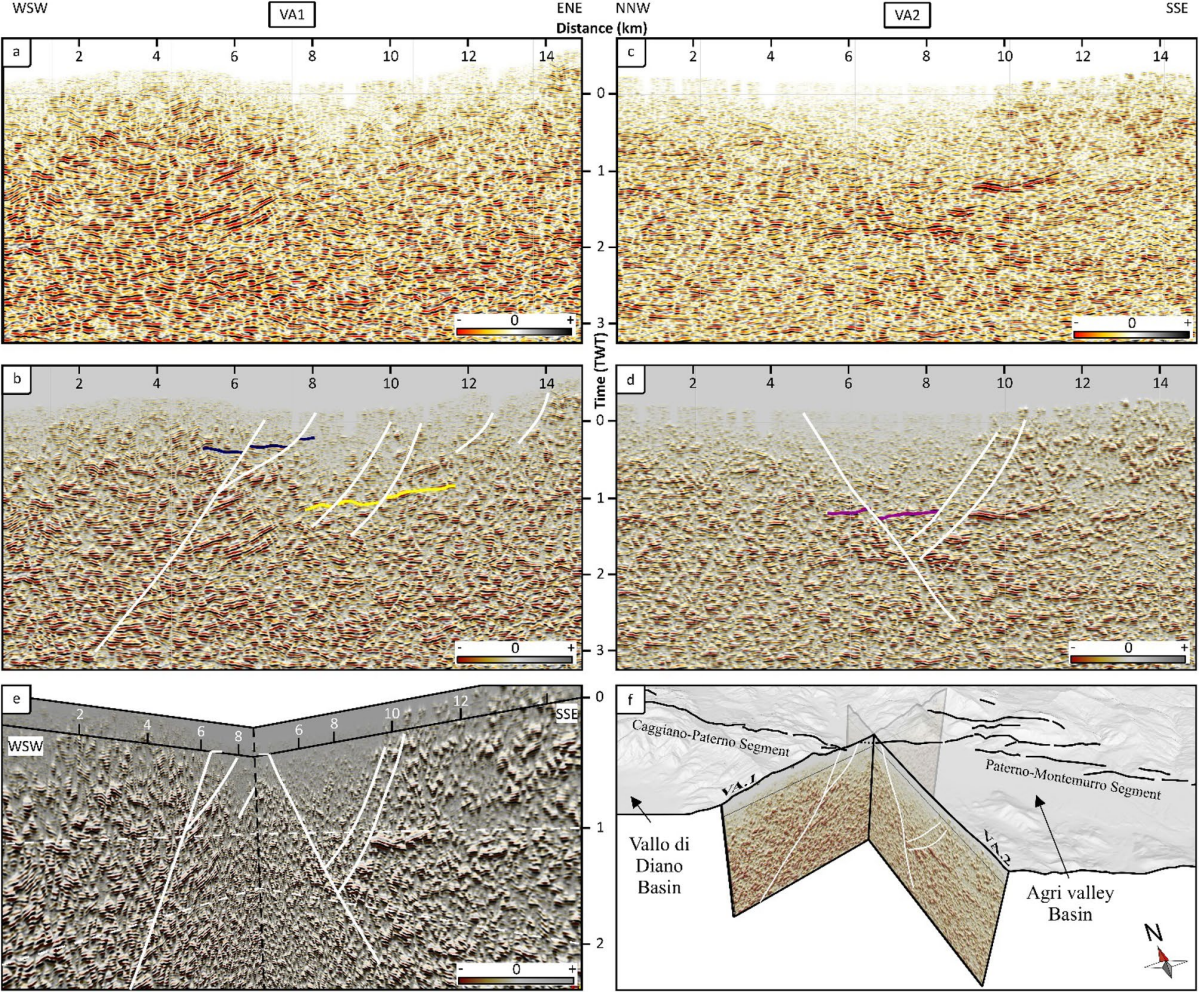
Seismic reflection profiles showing the subsurface seismic signature of the CMF; (**a**, **c)** conventional seismic reflection profiles in amplitude, after the application of pre-conditioning filters (PCf), showing a consistent reduction of high-frequency random noise; (**b**, **d)** co-rendering of PR attribute overlapped in transparency by PCf of panels (**a**–**c**), with line-drawing of the interpreted faults (white) and displaced reflections (yellow); (**e)** 3D view of the intersection between the two seismic lines (PR + PCf), focused in a key-sector of the study area; (**f)** a bird's eye view of the two profiles beneath an hillshaded 10 m-resolution topography in transparency (gray layer) including the CMF trace (black) mapped at surface.

Two seismic profiles (Fig. 4a, c) were interpreted by using a multi-attribute visualization by overlapping the pre-conditioned seismic profile (transparency variable between 65 and 75%) with the Pseudo Relief attribute (Fig. 4b, d). The profiles extend in NNW-SSE and WSW-ENE directions between the CMF northern and southern segments (CPS and PMS). Since interpretation was aimed to verify the presence or not beneath the Val d’Agri continental deposits of a normal fault connecting the two segments, we limited our interpretation to 3s TWT. To enhance the visualization of the associated displacement, in the absence of calibration wells, we used unnamed seismic reflections highlighted with colored lines (Fig. 4b, d).

The seismic profile VA1 (Fig. 4a, b) crosses the study area for a length of 15 km, from WSW to ENE (Fig. 3). In the shallower part of the profile, between distance 6 km and 12 km along the section trace, the presence of more chaotic and transparent reflections corresponds and underlies the Val d’Agri Basin (Figs. 1, 3). Below distance 6 km, between 0.5 and 1.5 s (TWT), a package of southwest-dipping discontinuous high-amplitude reflections does not continue to the south (blue lines, Fig. 4b). We interpreted their interruption as due to a southwest-dipping normal fault, which can be traced following the southern end of several other minor reflections and an alignment of a more transparent seismic facies. Below position 10 km, at ~1.1 s (TWT) we observe some displaced reflections (yellow lines, Fig. 4b) indicating the presence of two main southwest-dipping normal faults. Finally, in the northeastern part of the profile, a series of triangle-shaped areas, characterized by transparent seismic facies at ca. 0 s (TWT), and a group of interrupted low-amplitude reflections, suggest the presence of other minor structures dipping southwestwards.

The seismic profile VA2 (Fig. 4c, d) crosses the study area for 14.8 km, from NNW to SSE (Fig. 3). In the shallower part of the profile, between position 4 km and 10 km, the presence of more chaotic and transparent reflections corresponds to the deposits of the Val d’Agri Basin. Between position 8 km and 10 km, at 1s–2s (TWT), we observe a package of flat high-amplitude reflections whose northern termination suggests the presence of a north-dipping normal fault. Correspondingly, below position 6 km at ~1.5 s (TWT), there are discontinuous low-amplitude reflections (purple lines, Fig. 4d) which suggest the presence of a high-angle south-dipping normal fault.

Thanks to the interpretation of the VA1 and VA2 seismic reflection profiles, we constrain the lateral continuity of the northern and southern CMF segments, intercepted at position ~ 7 km on VA1 profile and ~ 5 km on profile VA2. A resultant N70° striking fault strand would connect the two segments, possibly as part of a single deep structure.

### Implications for the active tectonics

**Figure 5:**
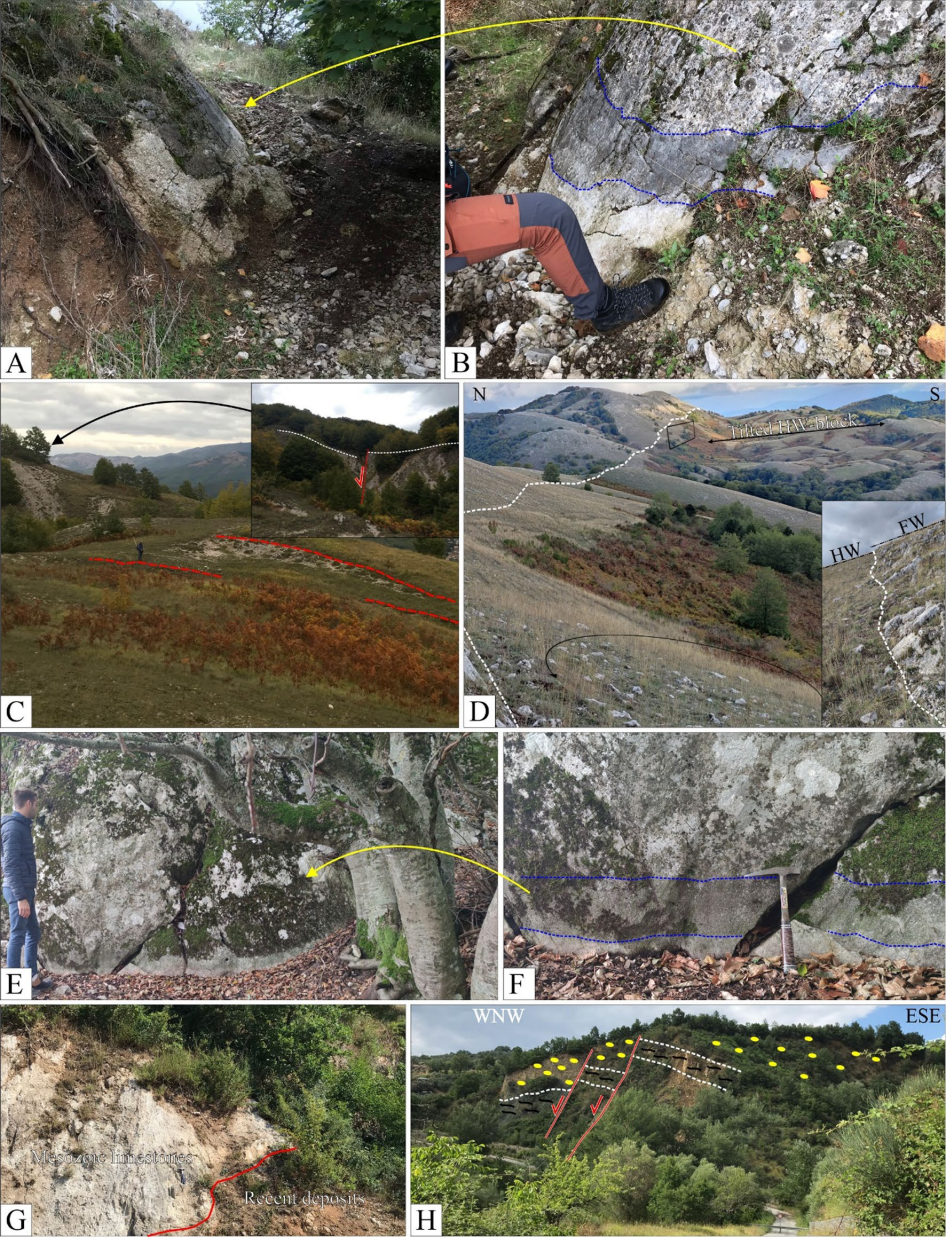
Evidence of Quaternary activity. **(A)** and **(B)** Weathered ribbons on limestone slickensides at the southern tip of the Timpe fault section. **(C)** Fault scarps in continuity at the southern tip of the Polla section. **(D)** Fault-controlled elongated basin in one of the topographically highest sectors of the Monti della Maddalena ridge, in the central portion of the Atena-Paterno section. **(E)** and **(F)** Weathered ribbons on limestone slickensides in the central portion of the Marsicovetere section. **(G)** Displaced recent deposits along the Marsicovetere section. **(H)** Displaced continental Quaternary fms along the Montemurro section.

The results of the survey campaigns, and in particular the presence of weathered ribbons and recent displaced soils and/or continental deposits (Fig. 5), indicate a late Pleistocene activity of the newly identified CMF alignment, as also shown from paleoseismic constraints^46^. Its ongoing activity is indeed testified by associate earthquake activity, but the timing of onset is questionable. We may preliminarily advance the hypothesis that the lack of an important basin associated and its development (transversal to the N–S Vallo di Diano basin), could mean likely onset during the later lower Pleistocene or even middle Pleistocene, post-onset of the Vallo di Diano boundary faults (Fig. 1). However, further analyses (*e.g.*, paleoseismology^24,47^ and GPR^79,80^) will be needed to better constrain the CMF evolution.

**Figure 6:**
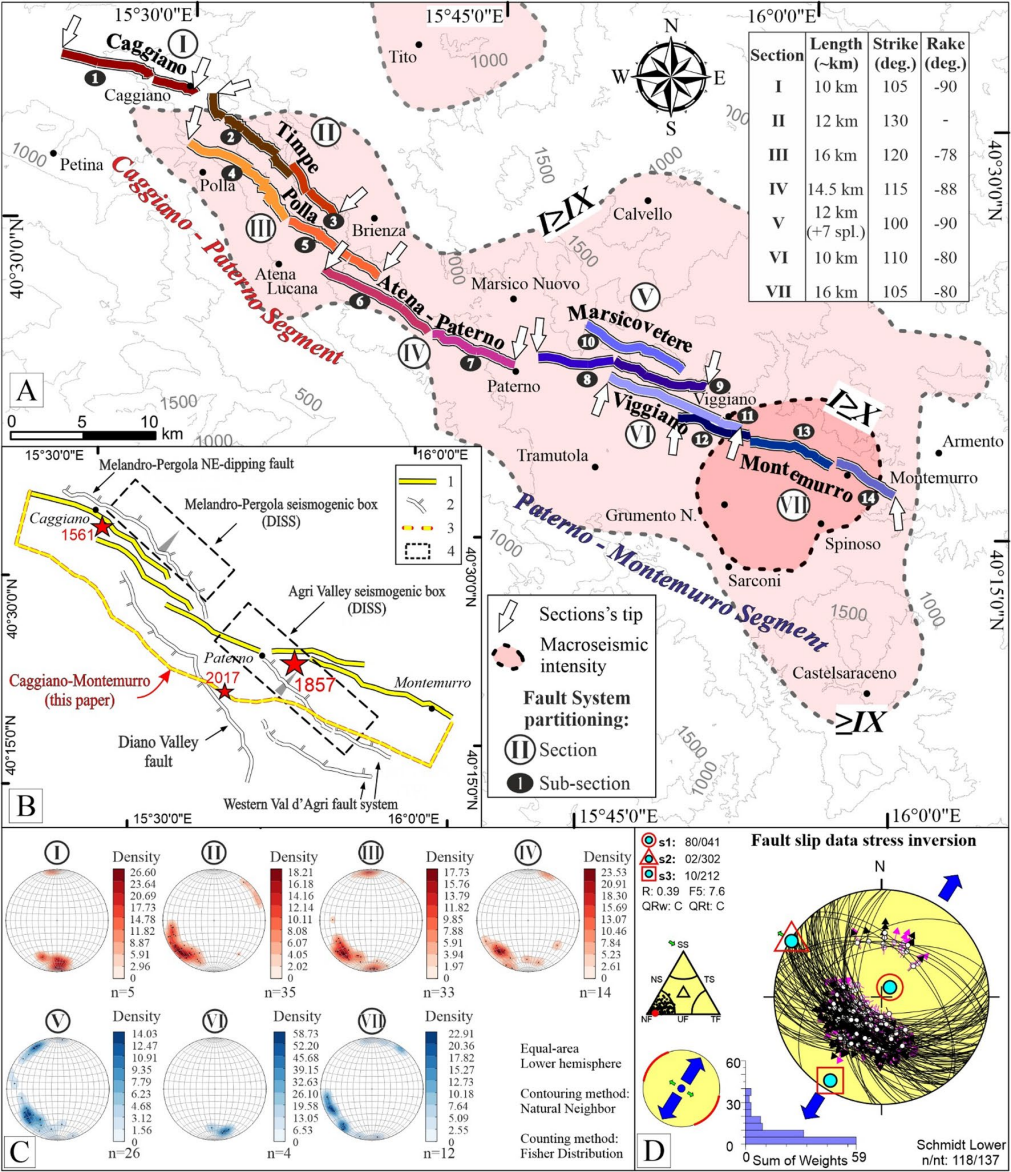
**(A)** Synthetic representation of the segmentation highlighted in this paper, in the context of the 1857 earthquake and Quaternary stress field. **(B)** (1) Caggiano-Montemurro Fault System (CMF); (2) Other fault systems from literature with ticks on downthrown side; (3) Surface projection of the inferred depth tip-line obtained enveloping the fault sections and extrapolating it with a constant 60° dip to 12 km depth; (4) Seismogenic boxes from DISS^3^. Red stars are the macroseismic epicenter of the 1857 and 1561^6^, and 2017^11^ earthquakes. **(C)** Results of the kinematic analysis represented in the contour of the T-axis of the deformation in stereoplots. **(D)** Stress inversion of the structural dataset. Inversion procedure as in Delvaux and Sperner^68^. Histogram = misfit angles *vs*. number of data points; nt = total number of data; n = number of successfully inverted data; σ1, σ2, σ3 = principal stress axes; R = stress ratio = (σ2 − σ3)/(σ1 − σ3); QR = quality factors as in^69,70^.

Our interpretation partially fit that of the previous authors^15,33,46,51,78^, who agreed in defining a late Quaternary activity, but did not recognize the entire length of the fault. While we reconstruct the CMF along-strike length for ~65 km, previous interpretations recognized only shorter segments, separated by important gaps. We note that these geometries and the fault length strongly limit the seismogenic potential and, therefore, the implications in terms of rupture propagation^27,81^. The kinematic analysis we carried out on the composite sites (Fig. 3; Table 1) and represented with the stereoplots in Fig. 6C, shows a general kinematic compatibility between the CMF and the stress field active in the intra-Apennine extensional belt^82^. In this regard, the inversion of the geological-structural dataset shows a minimum horizontal stress axis oriented N032, which slightly deviates from the ~ N045 axis previously defined in other areas of the Apennines (*e.g.*,^30,31,54,75,83,84^). An interesting observation derives from comparing our findings with the stress field obtained, using the same methodology, in the epicentral area of the 1980 Campania-Lucania earthquake^9^ (Table 3). Also in that case, the authors computed a NNE-SSW directed least principal stress, with a N031 minimum horizontal axis and considered it as a regional deviation from the classic SW–NE tensional direction across the Apennines of Italy. The inversions of the stress field of the two contiguous areas provide identical results (Table 3), confirming the hypothesis of a regional deviation and considerably enlarging to the whole Campanian-Lucanian Apennine, the area in which it occurs.

**Table 3:** Stress inversion parameters σ1, σ2, σ3 (trend/plunge) and R as computed in the inversion procedure of the geological datasets from this paper and from Bello et al.^9^ for the Irpinia earthquake epicentral area.

Geological dataset	No. of data (inverted/total)	σ1	σ2	σ3	R = (σ2−σ3)/(σ1−σ3)	QRw	QRt
CMF area—This paper	118/137 (86%)	041/80	302/02	032/10	0.39	C	B
Irpinia area—Bello et al.^9^	62/74 (84%)	235/83	121/03	031/07	0.31	B	B

The remarkable segmentation that characterizes the CMF (summarized in Table 2 and Fig. 6) is typical of many faults in extensional tectonic contexts (*e.g.*,^29,85,23^ and references therein). The fault we have highlighted develops with a series of closely spaced sections (~ 2 km), with large overlapping portions, along-strike bends, and numerous small gaps, especially between the small basins (Figs. S1, 2B, 2I, 5D). This arrangement could be interpreted as relating to a relatively young fault *sensu*^86,87^, along which the complete linkage of the contiguous segments, in delineating a continuous trace, has not been achieved. The low maturity of the structure could also explain the absence of a well-developed hanging wall basin associated with the fault.

In support of this interpretation, the field activities allowed us to map the sections and sub-sections of the CMF often at the edges of small “on the ridge” basins, frequent along the fault strike (*e.g.*, along the TF, APF, and MVF). The morphotectonic aspect of these small basins is surprisingly similar, with the outcropping fault being like a scarplet, a few tens of meters uphill from the cultivated plain, and running along the slope for the entire length of the basin. Where the depression gradually closes, generally against a low relief which acts as a threshold, also the scarplet disappears to then re-appear after a small gap (Fig. S1).

This segmented pattern could also suggest a possible diachronous reactivation of the fault segments and sections. As a result, the associated earthquakes may not correspond to the characteristic earthquake expected for the entire ~ 65 km-long structure, but smaller events may activate smaller portions, isolated or in cascade. In other words, this same high degree of segmentation could cause the release of multiple events, with segments triggering the adjoining structures^88^. The along-fault variability expressed by the segmentation of the structure has implications on the distribution of the slip during earthquakes, and for this reason, carefully studying its characteristics has evident implications for seismic hazard^28,29^.

One of the major seismotectonic implications of our new interpretation of the CMF, falls on its delimitation of the boundary fault system of the Vallo di Diano to the north, suggesting it also delimits its seismogenic potential, being a barrier to its northern development. Crossing the Monti della Maddalena ridge (Fig. 1, 3, 6B) and generating an offset in the stratigraphic sequences (Fig. S1c), the CMF sets a southern boundary to the Melandro–Pergola fault, also in this case, giving new implications on the seismogenic potential of the latter. However, the presence of the CMF does not deny the possible activity of the Melandro–Pergola fault or the Vallo di Diano boundary fault (Fig. 1, 3, 6B). In fact, we observe similar seismogenic characteristics in other areas such as the Vettore Fault System (central Italy), where structures 10–15 km apart, comparable with the CMF, are both active and seismogenic^28,89^.

## Discussion and conclusion

In light of our geological-structural analysis, derived from the complete integration of new data acquired remotely and from multiple survey campaigns, unedited seismic reflection profiles, and data from the literature, we highlight a more complex Quaternary extensional fault system than previously known. In particular, we describe a trans-ridge fault system that connects two basins (Auletta and Val d’Agri), crossing the Monti della Maddalena ridge (Fig. 6). The implications of this SSW-dipping structure, over 65 km long, are considerable in terms of local earthquake potential and of structural style of potentially seismogenic extensional sources. We summarize below the main implications of this research.

### Fault structure and segmentation

About 340 structural sites were surveyed along the various sections of the CMF, 140 of which include slip vectors obtained from kinematic indicators (*e.g.*, slickenlines and calcite steps) on the fault planes. We make available the structural data for future research in the Supplementary Material (Supplementary Data 2). The data highlights a fault with normal-to-oblique-normal kinematics (Fig. 2, 3; Table 1). In addition, the kinematic analysis indicates an increase, albeit with peaks that deviate from the general trend, of the dextral component along the fault strike moving southeastward from the northwest (Table 1, 2; Fig. 6C). This variation could be considered a kinematic partitioning due to the need for the faulted blocks to accommodate the same deformation field with differently-oriented tectonic structures.

The field activities, preceded by the analysis of satellite images, made it possible to highlight a complex fault pattern whose segmentation is defined according to generally recognized criteria (Table 2; Fig. 6A). We considered the presence of gaps, bends, overlaps, and underlaps, as well as the sudden variations of T-axis orientation, an index of kinematic partitioning^85,29,90^. In particular, we recognized two major segments with an along-strike length of ~ 30 km each (not including the splays and the overlaps between the sections; Fig. 6). We assume that, at shallow depths, the sections merge into a single fault due to their proximity at the surface (ca. 2 km). However, we are aware that to test this assumption, further studies, considering high-quality geological and geophysical data (*e.g.*, hypocentral depths and focal solutions, wells data and seismic profiles), are necessary to investigate the tectonic structures in-depth and in three dimensions.

In the portion of the study area where the CMF disappears beneath the Val d’Agri continental deposits (Fig. 1B, area e), we interpreted two unpublished seismic reflection profiles (Fig. 4, S2) to verify the lateral continuity or not between the northern and southern CMF segments. We observe that the fault zone intercepted at position ~ 7 km on VA1 profile and ~ 5 km on profile VA2, geometrically correlates well with both segments. Therefore, we propose a resultant N70° striking fault strand connecting the two left-laterally spaced segments. These reconstructions may suggest that the two segments are part of a single deep structure. However, at the surface, their linkage is obscured by the overlying anthropized continental deposits of the basin.

The Caggiano-Paterno Segment (CPS) is composed of the Caggiano (CFS), Timpe (TFS), Polla (PFS), and Atena-Paterno (APFS) sections, which are 10, 12, 16, and 14 km-long, respectively. The Paterno-Montemurro Segment (PMS) comprises Marsicovetere (MVFS), Viggiano (VIFS), and Montemurro (MOFS) sections, which are 12, 10, and 16 km-long, respectively (Fig. 6). Based on the geometric relationships between the sections, we divided them into 5- to 7 km-long sub-sections, generally arranged with a right-lateral en-échelon pattern. Locally, the sections of the CMF show minor E–W- or N–S-directed bends which, however, do not modify the overall geometry of the fault system. The northernmost (Caggiano, Timpe, and Polla) and the southern (Eastern Val d’Agri fault system) portions of the CMF were already known in the literature, but the overall along-strike extent of the trans-ridge fault highlighted in this work was unknown. Furthermore, structural data were missing for a large part of it. Thus, our new findings have a strong implication for the seismic hazard of the area.

### Implications for active structural setting

The CMF develops between the west-dipping Vallo di Diano border fault to the west, the east-dipping Melandro-Pergola fault system to the east (Fig. S1c), and the northeast-dipping Val d’Agri fault system to the south, partially limiting the extent of these structures. These observations neither confirm nor exclude the activity of these other faults, but certainly, constrain their size and seismogenic potential. The CMF may represent an Earthquake Gate (*sensu*^91^) exerting control over the maximum expected earthquakes of the faults surrounding it. For all these reasons, we believe that further research is necessary on this portion of the south-Apennine extensional belt and that these will have to take into account our results.

We consider the fault sections we mapped south of the Caggiano fault and the Timpe fault as their natural continuation and hypothesize that the whole CMF system may represent the main active fault of the area. According to paleoseismological investigations^46,92^, the Caggiano Section would have released the 1561 earthquake^62^ (Fig. 1a, 6B), and possibly one of the two shocks of the 1857 earthquake. From an instrumental point of view, at the upper crust level, the area is characterized by a low seismic release^93^ except for some microseismicity associated with shallow-depth east-dipping structures on the east-side Val d’Agri basin^94^. On 24 August 2017, a M_w_ 4.0 normal fault earthquake was released close to Montesano locality (Fig. 6), at a depth of about 14 km on a SW-dipping rupture plane^11^. This event may represent the down-dip continuation of the southern termination of the northern CMF segment.

Further constraints supporting the hypothesis that the CMF is the main active fault of the area derive from the results obtained in Zembo et al.^95,96^. These authors highlight two important observations: (i) the growth of the basin in the Val d’Agri with depocenter on the SW-dipping faults with the corresponding tilting of the dated strata and (ii) the substantial inactivity of the northernmost portion of the Val d’Agri (from Paterno towards Marsico Nuovo) where they place one of the three depocenters characterizing the Val d’Agri. From our observation of the morphological setting (*e.g.*, DEM in Fig. 1), the basin widens considerably only south of the CMF, in the portion crossing the Val d’Agri between Paterno and Galaino villages.

The inversion of the structural data (Fig. 6D) highlights a Quaternary NNE-SSW trending, near-horizontal, tensional principal stress co-axial with that calculated for the neighboring Campanian-Lucanian area, where the 1980 earthquake (M_w_ 6.9) was released^9^ (Table 3). The regional extent of such a tensional trend, which differs from the classic SW–NE one across the central Apennine Mountain belt, might suggest a regional crust-scale deviation relevant for seismotectonic investigations.

Furthermore, the CMF multi-scale segmentation pattern could represent a low degree of fault maturity (*sensu*^86^), the fault being nowadays in a stage of its growth characterized by fragments not linked yet, as expected for structures active for longer times^87^. Certainly, chronological constraints are needed, especially to date the more recent soils displaced by the fault. The fallout from this finding should be considered in future stress field analyses in this portion of Italy.

### Implications for seismic potential

The previous literature proposes two different source models for the nucleation of the 1857 earthquake: either two east-dipping faults (Melandro-Pergola and the western border fault of the Val d’Agri basin) for a total length of 40 km^16^, or a southwest-dipping alignment running for ~ 30 km from Marsico Nuovo to Montemurro along the western Val d’Agri fault system (Fig. 3)^15^. Differently from both, Galli et al.^46^ shows as the SW-dipping Caggiano, Timpe, and Polla faults were involved during the 1857 earthquake and possibly, entirely released a previous and relatively smaller event (1561, M_w_ 6.3)^77^, as well as a later minor one (1895, M_w_ 5.2)^62^.

In the light of our new data, we speculate on the possibility that the overall CMF, with a total length of 65 km, was activated and released the two in-sequence events, in a time-lapse of ~ 3 minutes^3,15,16^. The good fitting between the macroseismic field (Fig. 6a) and the CMF dimension, as well as the paleoseismological data for the northern portion of the CMF^46,62,92^ (*i.e.*, Caggiano, Timpe), would support this hypothesis.

The implications in terms of seismic hazard concern the possibility that, depending on the energy released by the earthquakes, events of different magnitude could be generated in case of partial or total activation of the fault, thus releasing complex source earthquakes or even cascade earthquakes^97^. Assuming that the CMF can rupture entirely during any possible earthquake (even a complex one with several sub-events), and based on the empirical relationships for normal faults by Wells and Coppersmith^98^, Wesnousky^1^, Leonard^99^, Galli et al.^92^ and Schirripa Spagnolo et al.^100^ (magnitude/fault length correlation), we obtain the magnitudes reported in Table 4.

**Table 4:** Maximum expected earthquake magnitudes (M_w_) from the length of the fault segments described in this paper, obtained from empirical equations.

Segm	Length (Km)	M_w_ (1)	M_w_ (2)	M_w_ (3)	M_w_ (4)	M_w_ (5)
CPS	35	6.90	6.85	6.85	5.82	6.75
PMS	30	6.81	6.81	6.74	6.71	6.66
TOT	65	7.25	6.97	7.31	7.27	7.14

CPS = Caggiano–Paterno Segment; PMS = Paterno–Montemurro Segment. Key: M_w_ (1) = Wells and Coppersmith^98^; M_w_ (2) = Wesnousky^1^; M_w_ (3) = Galli et al.^92^; M_w_ (4) = Leonard^99^; M_w_ (5) = Schirripa Spagnolo et al.^100^.

From these relationships, we can state that the complete rupture of CMF is correlated to a maximum expected magnitude between 7.0 and 7.3. The equation by Schirripa Spagnolo et al.^100^ give the result closest to the magnitude reported by the main catalogs^5^. Apart from Wesnousky’s^1^, all equations return a magnitude greater than 7.0, matching the estimated magnitude of the 1857 Basilicata earthquake (M_w_ 7.1). In the CMF we find the simplest possible explanation is supported both by literature data^46,95,96^ and by the new constraints introduced by this work. The two sub-events released in a time lapse of two minutes might result from the progressive activation of the northern segment first and the southern segments soon after. Our results can be used for guiding future high-resolution geophysical surveys (*e.g.*, GPR^79,80^) and paleoseismological investigations, to give further insights. For this reason, we applied the empirical equations also to the two segments (CPS and PMS), obtaining the seismic potential of each of them (Table 4). As suggested in Trippetta et al.^101^, the further characterization of the fault dimension and segmentation helped us to assess the area’s seismic potential.

### Broad interest implications

Ridge-bounding fault systems worldwide characterize intermountain seismic belts and related seismotectonic provinces, such as the Basin and Range in the USA and Italy‘s Apennines (*e.g.*,^20–22,44^). As the CMF described in this paper, trans-ridge faults are rarer. Their identification can represent a significant challenge for a complete understanding of active processes and seismic potential. In Italy, the first clue of the trans-ridge geometry was given by the fault system responsible for the 1980 Irpinia earthquake (M_w_ 6.9), whose fault trace, associated coseismic ruptures, and minor basinal depressions (size from few meters to tens and hundreds of meters) were surveyed at high altitudes along the main massifs^7,9,24^.

This innovative interpretative thinking implies that trans-ridge faults may control major earthquakes and can re-evaluate the seismotectonic interpretation of many areas at high seismic risk.

In the specific case of Italy, in the previous literature, attention as a potential source of major Campanian-Lucanian earthquakes, was focused on the ENE-dipping Melandro-Pergola fault system^16^, on the eastern^15^ or the western^16^ Val d’Agri fault system, on the WSW- dipping Caggiano-Timpe fault segments^46,62,92^, and even on WSW-dipping border fault of the Vallo di Diano basin^3^. The here field-identified trans-ridge CMF interlinks portion of the above faults (Caggiano-Timpe and the eastern Val d’Agri) with newly identified active fault sections (Atena–Paterno). So-doing builds a ~ 65 km-long WSW-dipping high-angle structure, articulated in two major segments (Fig. 6), which may represent the source of the two sub-events of the 1857 earthquake.

The cascade nature of the 1857 earthquake, with the release of two subevents in the time-lapse of a few minutes, is another reason of general interest. In the proposed interpretation, based on the integration of field data on fault segmentation and size with scale-law analysis, the above event might have entirely activated the two major CMF segments, for about 30 km each. Following Burrato and Valensise^16^ we might hypothesize a unilateral southward-directed rupture, activating first the northernmost segment and subsequently the southern one. However, we cannot exclude a bilateral propagation, first northward and soon after southward, from a central hypocentral position located more or less in correspondence with the interconnection area between the northern and southern CMF segments. An example of bilateral propagation is the 24 August 2016 Accumoli earthquake (M_w_ 6.2) of central Italy^102^. In that case, the nucleation of the two events was near-instantaneous. In this paper, we advance a new hypothesis on earthquake/fault association in the case of the 1857 event. However, although we are confident of the field structural constraints and the newly-defined trans-ridge structural style, we are conscious that further investigations are necessary to extrapolate the fault to hypocentral depths and to constrain seismotectonic interpretation, among which deeper seismic line interpretation and, for example, the building of alternative static Coulomb stress scenarios^103^.

A general seismotectonic interest arises from placing the 1857 event and the related fault system within the scenarios of some strong Italian earthquakes, such as Reggio Calabria-Messina 1908 (M_w_ 7.1), Irpinia 1980 (M_w_ 6.9), Southern Calabria 1783 (M_w_ 7.1) and Central Italy 2016–2017 (M_w_ 6.6)^6^. By focusing on the timing of the earthquake sub-events, these range from nearly instantaneous (1908), to some seconds (1980), few minutes (1857), few weeks (1783) and few months (2016), but released similar cumulate magnitudes. These earthquakes have been largely studied, but a robust and commonly accepted seismotectonic model is still debated. Future research could test if fault complexities also control the timing of activation of multi-events ruptures as it happens for the rupture propagation^20,29,104^.

The workflow adopted in this paper is based on structural fieldwork (also through modern digital tools), hierarchization of the outcropping Quaternary fault planes and kinematic indicators, fault/slip data inversion, seismic attribute analysis, and scale-law based inferences on earthquake/fault associations. It may represent a methodological approach for seismotectonic investigations, especially in areas with strong earthquakes historical records and modest instrumental seismicity. The lack of instrumental seismicity implies a lack of direct information on the fault geometry and seismogenic state of stress. However, high-detailed analysis of fault segmentation and kinematics may help infer hypocentral mechanisms and evaluate individual or combined segments' seismic potential when integrated with empirical equations.

Detailed structural investigation of faults potentially capable of rupturing the topographic surface in the occurrence of a strong earthquake can help not only geoscientists for scientific purposes worldwide but also institutions that deal with spatial planning or, for example, seismic microzonation studies, especially in fragile territories such as the one here studied, in which the social and economic fabric is integrated into the largest oilfield on land in Europe.

## Supplementary Information

Supplementary Information 1.

Supplementary Information 2.

Supplementary Information 3.

## Data Availability

The original contributions presented in the study are included in the article and in the Supplementary Material.
