# Supplementary material for: Complex trans-ridge normal faults controlling large earthquakes
Source: Sci Rep. 2022 Jun 23;12:10676. doi: 10.1038/s41598-022-14406-4 (PMC9226052; doi:10.1038/s41598-022-14406-4)
Supplement: Supplementary file 3 — Supplementary Information 3. [file 41598_2022_14406_MOESM3_ESM.docx]

# Supplementary Material for:

# Complex trans-ridge normal faults controlling large earthquakes

# Simone Bello^1,2*^, Giusy Lavecchia^1,2^, Carlo Andrenacci^1,2^, Maurizio Ercoli^2,3^, Daniele Cirillo^1,2^, Filippo Carboni^2,3^, Massimiliano R. Barchi^2,3^, Francesco Brozzetti^1,2^

^1^DiSPUTer, University G. d’Annunzio, via dei Vestini 31, Chieti 66100, Italy

^2^CRUST - Centro InteRUniversitario per l’analisi Sismotettonica Tridimensionale, Chieti, Italy

^3^Dipartimento di Fisica e Geologia, Università degli Studi di Perugia, Perugia, Italy

Corresponding author: [simone.bello@unich.it](mailto:simone.bello@unich.it)

The supplementary material of this work contains supplementary data (Supp Data 1 = a geo-referenced photographic documentation of the structural sites of this work; Supp Data 2 = a .txt file of the structural data), four supplementary figures (Supplementary Figure S1, S2, S3, S4) and the supplementary text referring to the macroseismic field of the 1857 earthquake (reported below). Supplementary Data are in separate files.


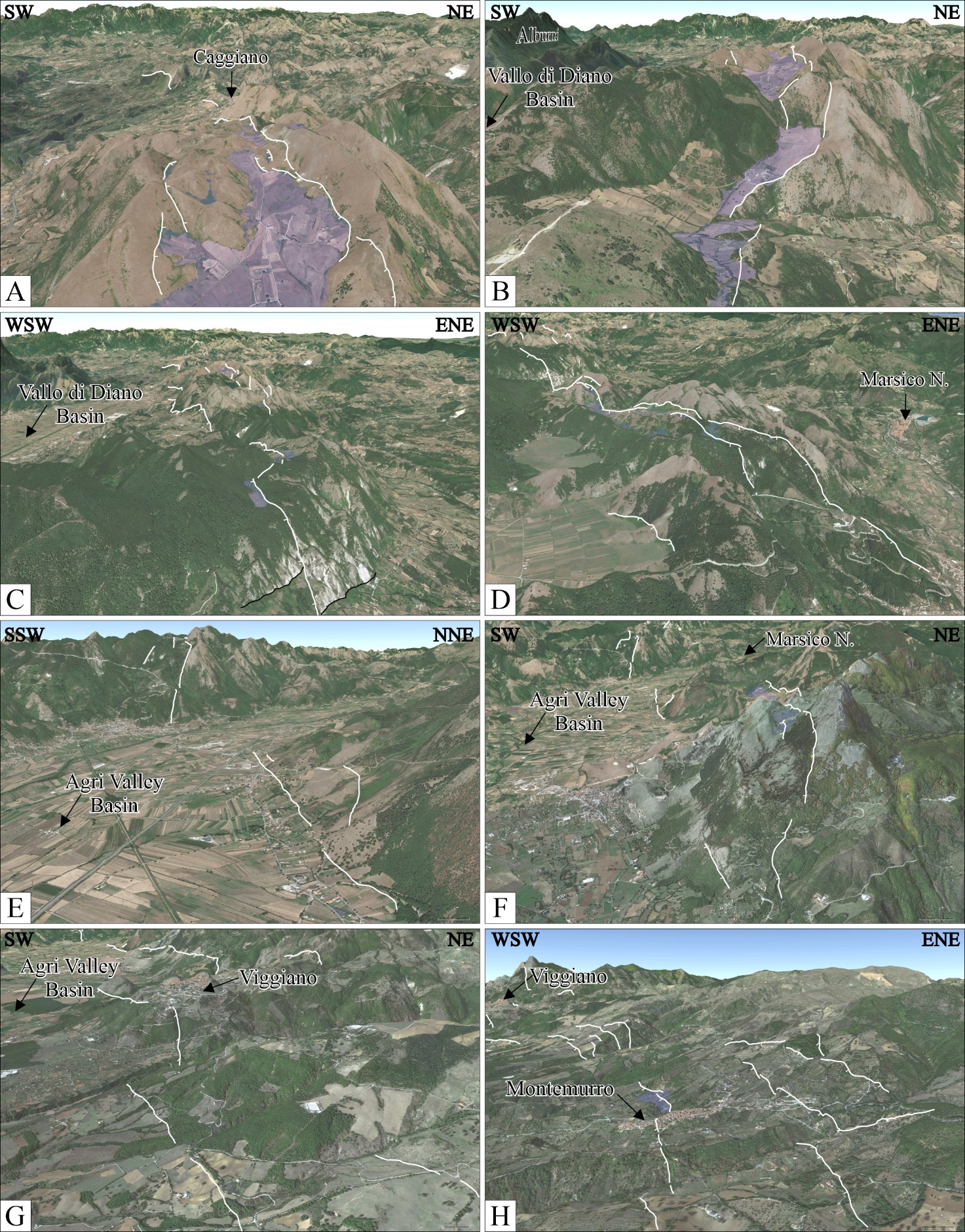


**Figure S1.** Key areas from photo-interpretation. Location in Figure 2 of the main text. White lines represent the photo-interpreted elements while blue polygons are the small intramountain Quaternary basins.


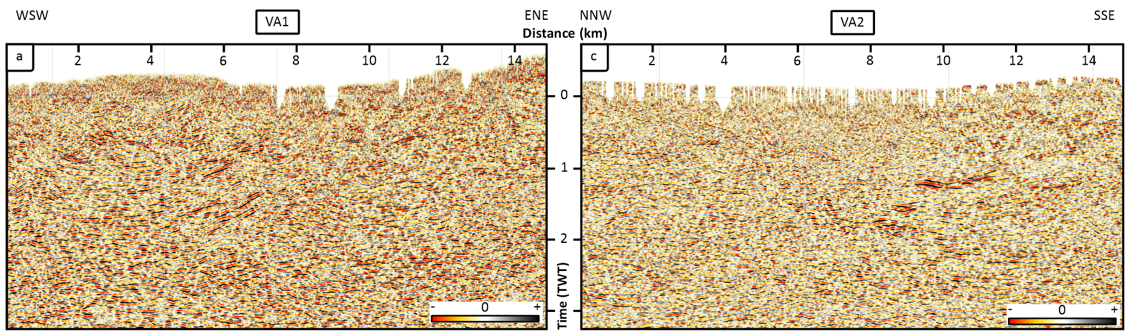


**Figure S2.** Conventional stack seismic reflection profiles (courtesy of Eni S.p.a.): the high level of random noise tends to obscure the reflections and the signal discontinuities, hampering the seismic interpretation.

**1857 earthquake macroseismic field**

The 16 December 1857 earthquake, occurred in Basilicata (Italy), is one of the most destructive seismic events recorded in Italy, the effects of which have been widely felt throughout the southern portion of the country. According to various authors (Mallet, 1862, Baratta 1901, Branno et al., 1985) the two main shocks, the second strongest occurring a few minutes after the first, generated most of the devastation and victims in a large area between the Diano (Campania) and the upper Agri Valleys (Basilicata). Figure S3a shows the macroseismic field of the Italian Macroseismic Database (DBMI15, Locati et al. 2022) composed of 314 macroseismic MSC intensities of the municipalities of central-southern Italy and the relative isoseismals obtained by the Natural Neighbor interpolation method (Sibson, 1981). The maximum intensities (XI° MSC) are recorded in Montemurro and Grumento Nova while 27 locations have intensity between X° and IX° mainly located between the Vallo di Diano and the upper Val d’Agri (Basilicata). Based on the macroseismic areas obtained from the interpolation, two areas with maximum intensity are identified: one elliptical between Montemurro, Viggiano and Grumento Nova and the other, less extensive between Polla, Atena Lucana and Brienza, this can be associated with the two distinct seismic events involving in the 1857 earthquake (Branno et al., 1985). The first was more resentful in the Vallo di Diano area and the second, stronger, concerned the High Agri Valley (Fig. S3b). The isosime of X° is elongated in the NW-SE direction for about 70 km, identifying a large area of damage of more than 1000 km extending towards the SE in the area of the macroseismic epicenter. The analysis of the macroseismic field suggests a more extensive seismic source than the previous interpretations (Benetti et al., 1998; Cello et al., 2000) extending between Polla and Montemurro involving the two SW dipping fault systems of the Vallo di Diano and the high Val d'Agri.


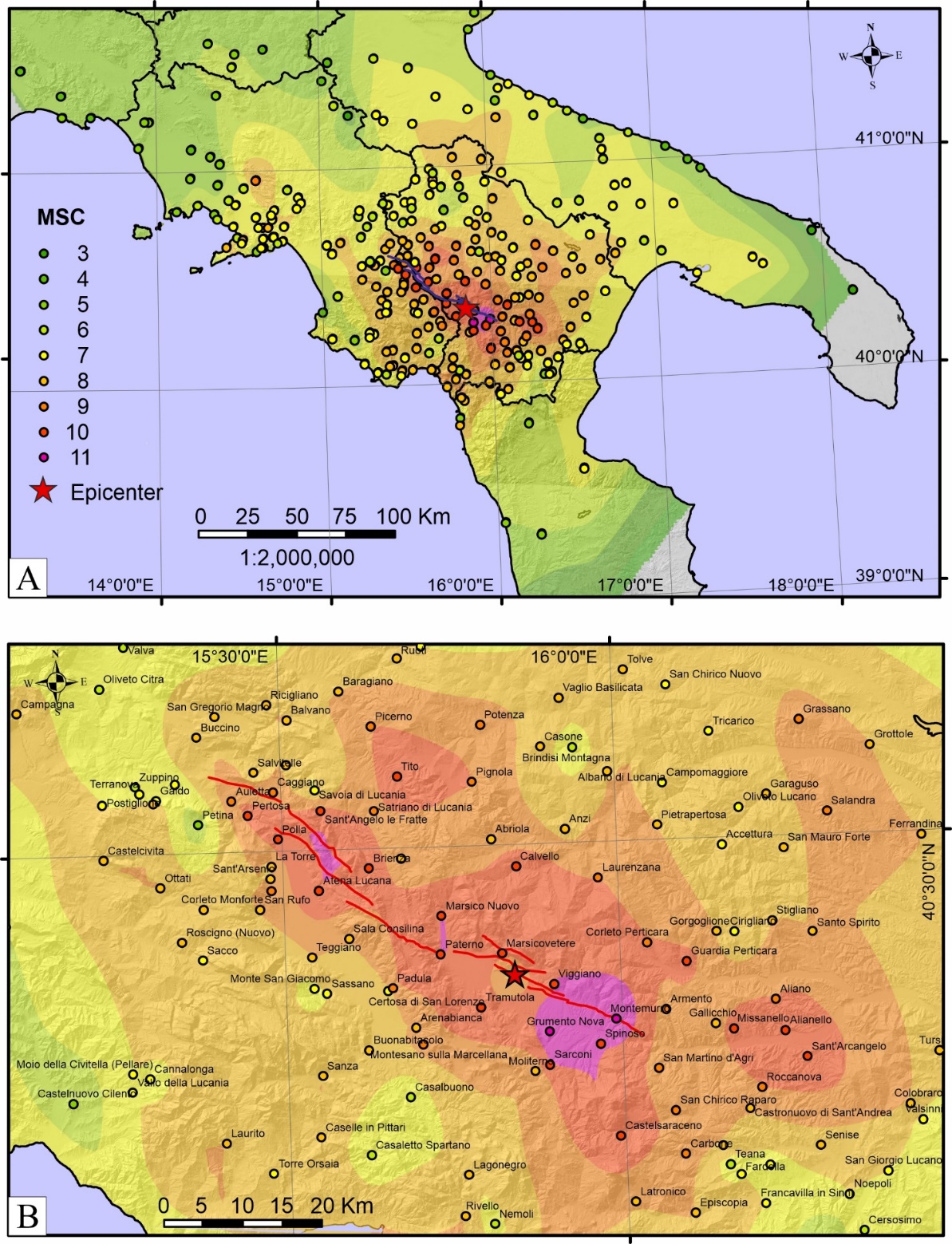


**Figure S3. (a)** Macroseismic field of the Italian Macroseismic Database (DBMI15, Locati et al. 2022) composed of 314 macroseismic MSC intensities of the municipalities of central-southern Italy and the relative isoseismals obtained by the Natural Neighbor interpolation method (Sibson, 1981). **(b)** Focus on the macroseismic epicentral area of the 1857 earthquake.

**
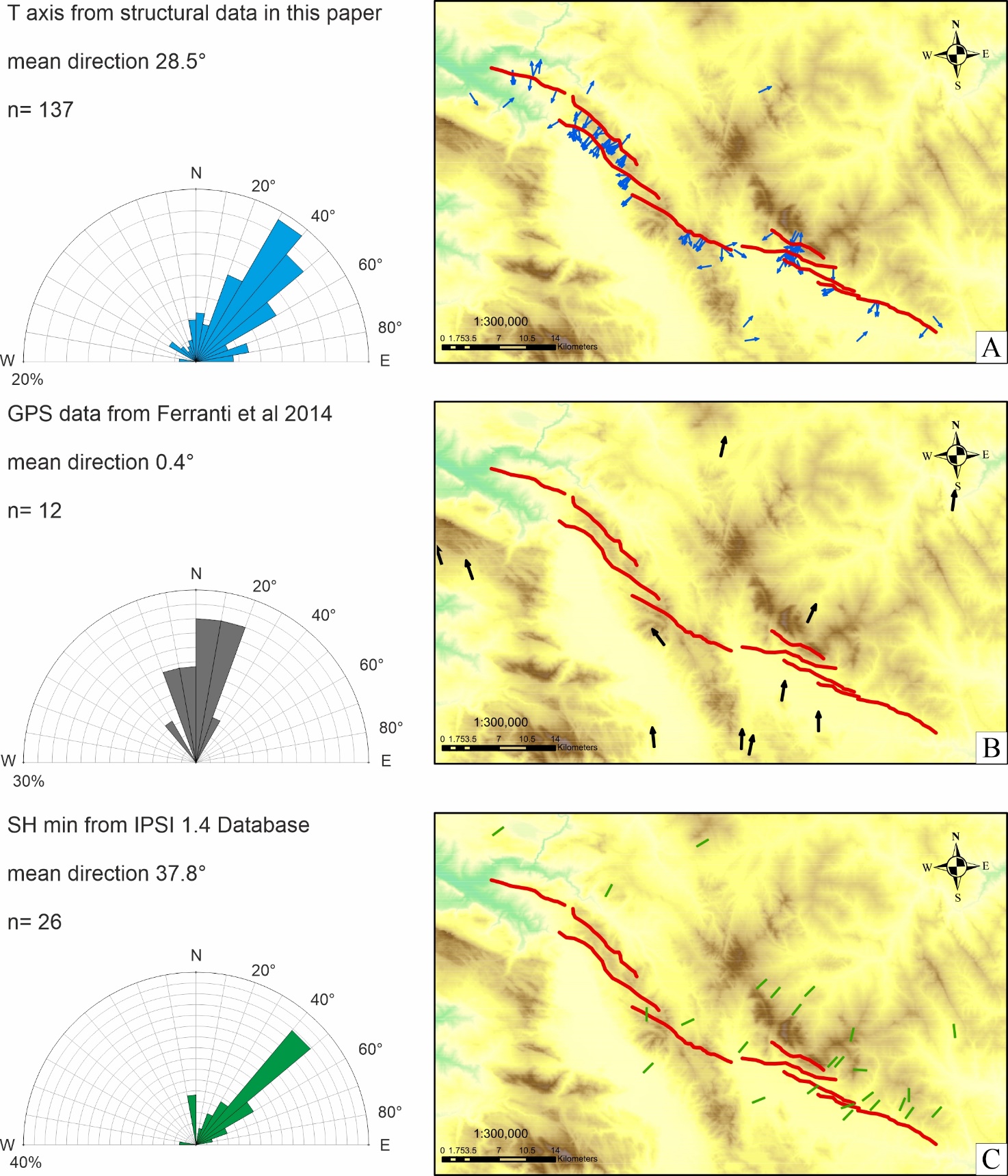
**

**Figure S4. (a)** T-axes from structural data of this paper and rose diagram of the directions. **(b)** GPS data from Ferranti et al., 2014 and rose diagram of the directions. **(c)** Shmin from the IPSI Database and rose diagram of the directions.

**Supp. material - References**

Baratta, M., 1901. I terremoti d’Italia, Arnoldo Forni Editore.

Benedetti, L., P. Tapponnier, G. C. P. King and L. Piccardi (1998), Surface faulting of the 1857 southern italian earthquake? Terra Nova, 10, 4, 206-210.

Branno, A., Esposito, E., Maturano, A., Porfido, S., Rinaldis, V., 1983. Studio, su base macrosismica, del terremoto della Basilicata del 16 dicembre 1857. Bollettino della Societa` dei Naturalisti di Napoli.

Cello, G., Martini, N., Paltrinieri, W., Tortorici, L., 1989. Structural styles in the frontal zones of the southern Apennines, Italy: an example from the Molise district. Tectonics 8 (4), 753–768.

Ferranti, L. et al. (2014). Rates of geodetic deformation across active faults in southern Italy. Tectonophysics 621, 101-122, doi:10.1016/j.tecto.2014.02.007.

G. Cello, E. Tondi, L. Micarelli, L. Mattioni (2003) Active tectonics and earthquake sources in the epicentral area of the 1857 Basilicata earthquake (southern Italy). Journal of Geodynamics 36 (2003) 37–50.

Locati M., Camassi R., Rovida A., Ercolani E., Bernardini F., Castelli V., Caracciolo C.H., Tertulliani A., Rossi A., Azzaro R., D'Amico S., Conte S., Rocchetti E., Antonucci A. (2022). Database Macrosismico Italiano (DBMI15), versione 4.0. Istituto Nazionale di Geofisica e Vulcanologia (INGV). <https://doi.org/10.13127/dbmi/dbmi15.4>.

Mallet, R., 1862. The Great Neapolitan Earthquake of 1857. The First Principles of Observational Seismology. London, vol. I, pp. 431, vol. II, pp. 399.

Mariucci, M. T. & Montone, P. (2020). Database of Italian present-day stress indicators, IPSI 1.4. Sci Data 7, 298, doi:10.1038/s41597-020-00640-w.

R. Sibson. A brief description of natural neighbor interpolation. Interpreting Multivariate Data, pages 21–36, 1981.
