# Supplementary material for: Complex trans-ridge normal faults controlling large earthquakes
Source: Sci Rep. 2022 Jun 23;12:10676. doi: 10.1038/s41598-022-14406-4 (PMC9226052; doi:10.1038/s41598-022-14406-4)
Supplement: Supplementary file 1 — Supplementary Information 1. [file 41598_2022_14406_MOESM1_ESM.pdf]

Supplementary

Complex trans-ridge normal faults controlling large earthquakes

Simone Bello[1,2*], Giusy Lavecchia[1,2], Carlo Andrenacci[1,2], Maurizio Ercoli[2,3], Daniele Cirillo[1,2], Filippo Carboni[2,3], Massimiliano R. Barchi[2,3], Francesco Brozzetti[1,2]

[1]DiSPUTer, University G. d'Annunzio, via dei Vestini 31, Chieti 66100, Italy
[2]CRUST - Centro InteRUniversitario per l'analisi Sismotettonica Tridimensionale, Chieti, Italy
[3]Dipartimento di Fisica e Geologia, Università degli Studi di Perugia, Perugia, Italy
Corresponding author: simone.bello@unich.it

Image_name	**latitude**	**longitude**	**altitude**	**time_date**
Figure 1	40.319148	15.946943	678.346523	21/07/2021 12:03:42
Figure 2	40.363731	15.823866	715.98819	23/07/2021 10:18:02
Figure 3	40.36993	15.828644	817.11795	23/07/2021 10:33:15
Figure 4	40.374686	15.820233	871.62442	23/07/2021 11:19:39
Figure 5	40.365788	15.702531	1077.080776	23/07/2021 13:44:53
Figure 6	40.399773	15.689233	1206.716396	23/07/2021 17:33:53
Figure 7	40.504016	15.574385	876.790039	12/10/2021 10:29:59
Figure 8	40.505087	15.574494	896.036377	12/10/2021 11:21:28
Figure 9	40.506978	15.574057	925.122559	12/10/2021 11:33:55
Figure 10	40.509412	15.572648	958.908203	12/10/2021 12:07:28
Figure 11	40.510418	15.572116	972.845459	12/10/2021 12:28:43
Figure 12	40.511437	15.56937	998.870293	12/10/2021 12:58:04
Figure 13	40.512946	15.561782	1028.509087	12/10/2021 13:30:57
Figure 14	40.494613	15.583565	930.567558	12/10/2021 16:32:44
Figure 15	40.494373	15.584236	943.327263	12/10/2021 16:36:40
Figure 16	40.486564	15.594242	1188.042107	12/10/2021 17:23:08
Figure 17	40.45689	15.607452	865.157707	13/10/2021 10:15:17
Figure 18	40.478194	15.570337	858.082634	13/10/2021 12:40:28
Figure 19	40.47813	15.570333	856.605339	13/10/2021 12:42:26
Figure 20	40.421573	15.647223	1123.199707	13/10/2021 16:35:11
Figure 21	40.419412	15.650533	1007.206753	13/10/2021 17:17:43
Figure 22	40.411216	15.656676	1087.660522	13/10/2021 18:08:39
Figure 23	40.443768	15.602587	1061.676235	14/10/2021 09:17:18
Figure 25	40.43801	15.613841	1093.672817	14/10/2021 13:05:52
Figure 26	40.448634	15.589431	964	14/10/2021 13:22:28
Figure 27	40.447263	15.590744	961	14/10/2021 13:33:44
Figure 28	40.470374	15.586565	1030.764397	14/10/2021 15:21:57
Figure 29	40.470348	15.586431	1023.392815	14/10/2021 15:26:37
Figure 30	40.470226	15.586777	1031.812004	14/10/2021 15:42:09
Figure 31	40.4701	15.587021	1029.452507	14/10/2021 15:48:10
Figure 32	40.469866	15.5874	1025.987419	14/10/2021 16:02:27

Figure 33	40.469376	15.588177	1026.431999	14/10/2021 16:22:35
Figure 34	40.468772	15.588566	1006.673943	14/10/2021 16:34:18
Figure 36	40.467792	15.589924	993.861443	14/10/2021 16:44:10
Figure 37	40.387539	15.719588	841.962734	15/10/2021 12:07:52
Figure 38	40.517848	15.5556	1008.949638	12/10/2020 17:36:04
Figure 39	40.527406	15.507326	635.463799	09/10/2020 12:16:04
Figure 40	40.568829	15.356424	308.633389	24/07/2021 16:14:32
Figure 41	40.39856	15.687862	1153.735123	23/07/2021 17:43:33
Figure 42	40.399414	15.686094	1147.038408	11/10/2021 14:51:46
Figure 43	40.431641	15.651517	943.201748	13/10/2020 12:10:33
Figure 44	40.365716	15.70251	1068.562954	23/07/2021 13:46:56
Figure 45	40.527429	15.507114	626.634819	09/10/2020 12:57:35
Figure 46	40.280207	15.877821	658.036537	22/07/2021 12:02:07
Figure 47	40.399773	15.689233	1206.716396	23/07/2021 17:33:57
Figure 49	40.494204	15.584898	963.153069	12/10/2021 16:47:58
Figure 52	40.446589	15.604952	1064.398403	14/10/2021 12:25:59
Figure 53	40.504039	15.574441	862.269402	12/10/2021 10:42:38
Figure 55	40.421394	15.647341	1110.196915	13/10/2021 16:42:11
Figure 56	40.470094	15.587081	1032.712517	14/10/2021 15:47:58
Figure 57	40.47016	15.586812	1028.406975	14/10/2021 15:42:06
Figure 58	40.526898	15.50786	617.791786	09/10/2020 12:00:59
Figure 59	40.527523	15.506305	620.495049	09/10/2020 13:06:14
Figure 60	40.592562	15.420644	450.009487	12/10/2020 10:25:51
Figure 61	40.420936	15.647818	1101.246181	13/10/2021 16:53:11
Figure 63	40.504166	15.574316	879.858887	12/10/2021 10:46:59
Figure 64	40.469798	15.587397	1021.119743	14/10/2021 16:02:47
Figure 65	40.374677	15.820286	872.160675	23/07/2021 11:17:43
Figure 66	40.50388	15.574473	878.178711	12/10/2021 10:51:45
Figure 67	40.585239	15.447597	874.819857	10/10/2020 17:22:44
Figure 68	40.527308	15.507285	631.096663	09/10/2020 12:13:50
Figure 69	40.503887	15.574427	875.220634	12/10/2021 10:50:50
Figure 70	40.504313	15.574414	886.870117	12/10/2021 11:03:50
Figure 71	40.519968	15.604094	515.383904	13/10/2020 14:20:20
Figure 72	40.280259	15.877786	652.103188	22/07/2021 12:04:57
Figure 73	40.504001	15.574378	867.622795	12/10/2021 10:33:02
Figure 74	40.422184	15.647732	1093.996948	13/10/2021 16:14:17
Figure 75	40.411234	15.656702	1073.373257	13/10/2021 18:09:08
Figure 78	40.470351	15.58652	1029.756096	14/10/2021 15:32:43
Figure 79	40.469349	15.58815	1031.829704	14/10/2021 16:22:45
Figure 80	40.369885	15.82851	811.498932	23/07/2021 10:37:51
Figure 81	40.504297	15.574384	875.805882	12/10/2021 11:05:15
Figure 82	40.503787	15.574331	870.599663	12/10/2021 11:11:46
Figure 83	40.503778	15.574367	876.550049	12/10/2021 11:12:21
Figure 84	40.503778	15.574367	876.550049	12/10/2021 11:12:25
Figure 85	40.468624	15.588726	1006.692497	14/10/2021 16:33:35
Figure 86	40.391423	15.812715	1059.015778	13/10/2021 11:04:07
Figure 87	40.592297	15.419557	446.945217	12/10/2020 10:15:32
Figure 88	40.384018	15.822537	967.970734	13/10/2021 11:25:35
Figure 89	40.399699	15.689412	1211.679565	11/10/2021 12:35:18

Figure 90	40.551485	15.403071	360.630947	24/07/2021 15:46:40
Figure 91	40.394721	15.696884	1267.342688	11/10/2021 14:16:27
Figure 92	40.527459	15.506519	640.352837	09/10/2020 13:06:32
Figure 93	40.467816	15.589886	994.810417	14/10/2021 16:44:16
Figure 95	40.512913	15.561731	1028.950127	12/10/2021 13:32:55
Figure 96	40.335936	15.883409	701.468012	21/07/2021 17:14:51
Figure 97	40.389666	15.717147	1070.493397	12/10/2021 11:26:35
Figure 98	40.399699	15.689412	1211.679565	11/10/2021 12:35:34
Figure 99	40.3986	15.687837	1155.908251	23/07/2021 17:44:36
Figure 100	40.509383	15.572724	941.351074	12/10/2021 12:08:51
Figure 101	40.512945	15.56185	1028.009331	12/10/2021 13:32:47
Figure 102	40.422207	15.647778	1099.335415	13/10/2021 16:12:49
Figure 103	40.421575	15.647171	1110.320923	13/10/2021 16:40:48
Figure 104	40.448625	15.589515	957.02404	14/10/2021 13:27:19
Figure 105	40.447068	15.591093	971	14/10/2021 13:37:00
Figure 106	40.470328	15.586538	1024.217278	14/10/2021 15:23:58
Figure 107	40.46988	15.587372	1025.760246	14/10/2021 16:03:38
Figure 108	40.512913	15.561731	1028.950127	12/10/2021 13:32:58
Figure 109	40.51683	15.525147	654.90922	10/10/2020 10:36:27
Figure 110	40.533354	15.498431	712.601167	09/10/2020 11:13:22
Figure 111	40.527331	15.507394	638.037773	09/10/2020 12:22:37
Figure 112	40.369904	15.828673	812.829477	23/07/2021 10:32:24
Figure 113	40.516322	15.531632	808.887871	09/10/2020 17:04:39
Figure 114	40.524538	15.516267	656.852348	09/10/2020 14:28:11
Figure 115	40.58524	15.447597	875.314053	10/10/2020 17:22:08
Figure 116	40.494337	15.5844	959.915031	12/10/2021 16:44:17
Figure 117	40.478211	15.57031	860.885368	13/10/2021 12:42:07
Figure 118	40.469812	15.587425	1027.82592	14/10/2021 16:01:38
Figure 119	40.494282	15.584465	957.692741	12/10/2021 16:41:38
Figure 120	40.551527	15.402532	344.77792	24/07/2021 15:35:54
Figure 121	40.399772	15.689168	1197.021938	23/07/2021 17:33:45
Figure 122	40.377574	15.693418	1043.598965	23/07/2021 15:44:51
Figure 123	40.563657	15.526131	589.522044	13/10/2020 18:08:05
Figure 124	40.395359	15.696213	1273.121073	11/10/2021 13:35:28
Figure 125	40.546877	15.788429	1139.321655	23/05/2017 13:58:08
Figure 126	40.398832	15.690727	1228.078945	11/10/2021 12:43:11
Figure 127	40.398842	15.690723	1228.79855	11/10/2021 12:42:51
Figure 128	40.505242	15.574362	898.874512	12/10/2021 11:28:29
Figure 129	40.421165	15.646922	1122.647515	13/10/2021 16:32:48
Figure 130	40.47037	15.586692	1038.247661	14/10/2021 15:36:38
Figure 131	40.515742	15.532186	803.103691	09/10/2020 17:16:50
Figure 132	40.320259	15.942741	689.281826	21/07/2021 12:24:09
Figure 133	40.592531	15.420603	448.910122	12/10/2020 10:23:31
Figure 134	40.289791	16.017608	768.576565	20/07/2021 15:47:51
Figure 135	40.297356	15.744722	843.173409	24/07/2021 10:10:20
Figure 136	40.504141	15.574488	862.833488	12/10/2021 10:39:37
Figure 137	40.447258	15.590647	953.749626	14/10/2021 13:43:40
Figure 138	40.524516	15.516495	669.352715	09/10/2020 15:07:59
Figure 139	40.284262	16.036979	813.519503	20/07/2021 15:07:42

Figure 140	40.527362	15.507361	634.425224	09/10/2020 12:18:54
Figure 141	40.39463	15.697049	1265.557463	11/10/2021 13:39:56
Figure 142	40.585215	15.447659	875.65292	10/10/2020 17:26:22
Figure 143	40.527405	15.506465	643.745781	09/10/2020 12:48:29
Figure 144	40.527308	15.507285	631.096663	09/10/2020 12:14:02
Figure 145	40.421571	15.647147	1116.184536	13/10/2021 16:36:38
Figure 146	40.446896	15.591159	394.227529	14/10/2021 13:39:53
Figure 147	40.447257	15.590646	954.140984	14/10/2021 13:43:44
Figure 148	40.27717	15.909768	607.065498	22/07/2021 17:26:53
Figure 149	40.369977	15.828756	812.937868	23/07/2021 10:34:44
Figure 150	40.519802	15.604877	508.193718	13/10/2020 14:16:07
Figure 151	40.302616	16.024546	919.868382	20/07/2021 13:26:25
Figure 152	40.394541	15.698522	1269.693206	11/10/2021 13:54:47
Figure 153	40.401364	15.676731	1138.35	11/10/2021 15:44:32
Figure 154	40.365797	15.70266	1068.874355	23/07/2021 13:45:01
Figure 156	40.302606	16.024557	919.191013	20/07/2021 13:26:39
Figure 157	40.51174	15.535618	838.124687	09/10/2020 17:31:40
Figure 158	40.568829	15.356424	308.633389	24/07/2021 16:14:42
Figure 159	40.506438	15.545666	927.578628	09/10/2020 18:39:21
Figure 160	40.526875	15.507823	616.779715	09/10/2020 12:01:10
Figure 161	40.527307	15.507079	642.268852	09/10/2020 12:48:48
Figure 162	40.548794	15.784024	1116.131937	23/05/2017 11:46:57
Figure 163	40.302887	15.993988	801.606045	22/07/2021 09:18:52
Figure 164	40.319116	15.946941	677.310434	21/07/2021 12:04:25
Figure 165	40.421394	15.647341	1110.196915	13/10/2021 16:41:24
Figure 166	40.470384	15.586643	1036.211418	14/10/2021 15:32:37
Figure 167	40.39856	15.687862	1153.735123	23/07/2021 17:43:26
Figure 168	40.290321	15.914287	528.808662	22/07/2021 15:36:18
Figure 169	40.282293	15.746921	870.035469	24/07/2021 09:39:52
Figure 170	40.592344	15.420927	453.985622	12/10/2020 10:27:38
Figure 171	40.289797	16.017616	767.987209	20/07/2021 15:48:07
Figure 172	40.534426	15.536307	1159.548033	11/10/2020 16:01:36
Figure 174	40.527363	15.506818	646.286822	09/10/2020 13:01:40
Figure 175	40.519277	15.604491	511.236198	13/10/2020 14:13:19
Figure 176	40.398958	15.690535	1228.789805	11/10/2021 14:38:32
Figure 177	40.362102	15.827933	685.574141	23/07/2021 09:47:38
Figure 178	40.335984	15.883352	706.918976	21/07/2021 17:12:22
Figure 179	40.369898	15.82867	805.867584	23/07/2021 10:32:08
Figure 180	40.533913	15.495682	704.96703	09/10/2020 10:38:35
Figure 181	40.280204	15.877834	665.538368	22/07/2021 12:04:59
Figure 182	40.592542	15.420237	441.144741	12/10/2020 10:41:35
Figure 183	40.534439	15.536199	1161.485771	11/10/2020 16:11:39
Figure 184	40.296282	16.002681	721.800076	20/07/2021 16:21:01
Figure 185	40.39444	15.697788	1268.513356	11/10/2021 13:51:01
Figure 186	40.515731	15.532231	809.915947	09/10/2020 17:18:24
Figure 187	40.592572	15.420633	451.293972	12/10/2020 10:25:40
Figure 188	40.524808	15.514671	611.424004	09/10/2020 15:33:39
Figure 189	40.551527	15.402532	344.77792	24/07/2021 15:36:02
Figure 190	40.396647	15.694814	1286.748379	11/10/2021 13:09:28

Figure 191	40.524516	15.516495	669.352715	09/10/2020 15:07:47
Figure 192	40.362014	15.827872	684.410892	23/07/2021 09:48:54
Figure 193	40.319064	15.946988	675.9087	21/07/2021 12:03:07
Figure 194	40.568767	15.356402	312.206579	24/07/2021 16:15:19
Figure 195	40.395792	15.69604	1284.634204	11/10/2021 13:27:56
Figure 196	40.516245	15.531526	804.363945	09/10/2020 17:04:12
Figure 197	40.551607	15.402626	356.006924	24/07/2021 15:44:47
Figure 199	40.320269	15.942903	680.980068	21/07/2021 12:26:06
Figure 201	40.394723	15.696866	1266.76183	11/10/2021 14:16:39
Figure 202	40.395231	15.696373	1274.578127	11/10/2021 13:34:09
Figure 203	40.515734	15.532331	813.937675	09/10/2020 17:21:35
Figure 204	40.399783	15.689163	1196.871792	23/07/2021 17:33:31
Figure 205	40.304627	15.994275	826.505459	22/07/2021 09:57:54
Figure 206	40.302652	16.024524	918.021336	20/07/2021 13:20:40
Figure 207	40.51173	15.535542	831.637383	09/10/2020 17:28:38
Figure 208	40.53444	15.536317	1160.022149	11/10/2020 16:01:24
Figure 209	40.541892	15.55935	595.872734	13/10/2020 15:28:15
Figure 210	40.52484	15.514872	625.577446	09/10/2020 15:32:51
Figure 211	40.506434	15.545339	915.421806	09/10/2020 18:27:40
Figure 212	40.397676	15.692039	1228.567715	11/10/2021 12:52:38
Figure 213	40.363825	15.823959	720.939362	23/07/2021 10:16:23
Figure 214	40.592582	15.420586	452.526699	12/10/2020 10:22:55
Figure 215	40.516813	15.524879	632.257091	10/10/2020 10:18:28
Figure 216	40.551546	15.402525	346.790371	24/07/2021 15:35:37
Figure 217	40.525039	15.577185	623.912744	13/10/2020 15:06:16
Figure 218	40.528828	15.495462	557.043602	09/10/2020 11:33:17
Figure 219	40.394468	15.697351	1256.192431	11/10/2021 13:42:37
Figure 220	40.519544	15.604171	513.311272	13/10/2020 14:11:00
Figure 221	40.320259	15.942741	689.281826	21/07/2021 12:24:02
Figure 222	40.516164	15.531666	802.881279	09/10/2020 16:57:29
Figure 223	40.522993	15.551176	1047.343437	12/10/2020 17:06:46
Figure 224	40.363757	15.82391	718.93073	23/07/2021 10:15:20
Figure 225	40.569179	15.484732	732.420535	12/10/2020 14:16:55
Figure 226	40.39444	15.697767	1268.256394	11/10/2021 13:50:54
Figure 227	40.551456	15.403104	355.789639	24/07/2021 15:46:32
Figure 228	40.397609	15.692167	1234.069428	11/10/2021 12:53:35
Figure 229	40.551522	15.402542	349.373989	24/07/2021 15:35:21
Figure 231	40.2954	16.002934	702.223293	20/07/2021 16:12:03
Figure 232	40.405951	15.671942	1292.696365	11/10/2021 16:10:41
Figure 233	40.31676	15.965582	665.991299	20/07/2021 19:15:58
Figure 234	40.38723	15.835956	1260.792511	13/10/2021 14:25:29
Figure 235	40.424927	15.64109	1252.495815	14/10/2021 10:12:32
Figure 236	40.374602	15.830084	1017.656281	13/10/2021 15:10:18
Figure 238	40.36951	15.810201	695.197418	14/10/2021 10:01:52
Figure 239	40.385681	15.768013	673.454433	15/10/2021 15:18:13
Figure 240	40.365536	15.821499	755.927765	14/10/2021 11:03:41
Figure 241	40.363551	15.871588	948.80589	14/10/2021 11:45:59
Figure 242	40.402646	15.828791	1422.726837	14/10/2021 16:49:13
Figure 244	40.387565	15.719615	845.450039	15/10/2021 12:09:17

Figure 245	40.383373	15.720645	851.83166	12/10/2021 10:55:40
Figure 246	40.299823	15.965418	613.734707	21/07/2021 11:10:55
Figure 247	40.320263	15.942881	683.483852	21/07/2021 12:27:32
Figure 248	40.384923	15.767911	671.554287	12/10/2021 16:45:22
Figure 249	40.369971	15.828605	809.022369	23/07/2021 10:38:31
Figure 250	40.378016	15.824965	1020.233381	13/10/2021 11:36:05
Figure 251	40.369525	15.809026	659.789215	23/07/2021 11:53:58
Figure 252	40.381401	15.829919	1201.170807	13/10/2021 11:56:20
Figure 253	40.483434	15.596578	1085.475017	12/10/2021 18:08:41
Figure 256	40.403198	15.675598	1221.184048	11/10/2021 15:44:32
Figure 257	40.302716	16.024549	902.906834	20/07/2021 13:16:17
Figure 258	40.405935	15.671841	1296.464363	11/10/2021 16:12:26
Figure 259	40.362282	15.871634	903.889557	14/10/2021 12:52:45
Figure 260	40.399914	15.832823	1372.333171	14/10/2021 17:16:29
Figure 261	40.387532	15.719486	840.08102	15/10/2021 11:54:31
Figure 262	40.387572	15.719607	843.346767	15/10/2021 12:09:24
Figure 263	40.384923	15.767911	671.554287	12/10/2021 16:45:33
Figure 264	40.385402	15.818778	984.794098	13/10/2021 10:16:44
Figure 265	40.387087	15.835955	1263.708038	13/10/2021 12:20:27
Figure 266	40.374543	15.830073	1016.989777	13/10/2021 15:04:05
Figure 267	40.374581	15.830073	1021.175079	13/10/2021 15:10:47
Figure 268	40.36951	15.810201	695.197418	14/10/2021 10:02:01
Figure 270	40.299431	15.99824	787.571602	21/07/2021 19:29:45
Figure 271	40.405957	15.671897	1289.672106	11/10/2021 16:15:24
Figure 272	40.399887	15.832835	1374.900383	14/10/2021 17:17:57
Figure 273	40.387699	15.719637	844.246914	15/10/2021 12:18:31
Figure 274	40.385424	15.818733	984.640852	13/10/2021 10:19:57
Figure 275	40.539853	15.562315	609.336723	13/10/2020 15:49:06
Figure 276	40.374673	15.83006	999.686676	13/10/2021 15:11:34
Figure 277	40.36948	15.810343	693.469391	14/10/2021 10:04:22
Figure 278	40.559494	15.778651	863.701624	23/05/2017 15:00:04
Figure 279	40.403415	15.675519	1221.278088	11/10/2021 15:46:07
Figure 282	40.389899	15.716231	1076.563442	12/10/2021 11:30:39
Figure 285	40.554977	15.783468	1060.758057	23/05/2017 11:09:43
Figure 286	40.387053	15.835997	1268.974152	13/10/2021 12:25:09
Figure 287	40.376546	15.821897	944.781036	13/10/2021 15:27:13
Figure 288	40.369441	15.810334	694.973663	14/10/2021 10:05:50
Figure 289	40.360912	15.88162	901.827057	14/10/2021 13:08:52
Figure 290	40.40537	15.6756	1266.688918	11/10/2021 15:59:22
Figure 291	40.405942	15.671467	1295.152187	11/10/2021 16:18:35
Figure 294	40.386736	15.836115	1282.824738	13/10/2021 12:31:11
Figure 295	40.38019	15.814226	898.497955	13/10/2021 16:10:11
Figure 296	40.369441	15.810334	694.973663	14/10/2021 10:06:01
Figure 297	40.360904	15.881678	903.396918	14/10/2021 13:09:34
Figure 298	40.40586	15.673533	1290.204751	11/10/2021 16:04:07
Figure 299	40.40599	15.67134	1300.123257	11/10/2021 16:21:56
Figure 301	40.391201	15.812981	1055.154456	13/10/2021 10:54:50
Figure 302	40.386501	15.836274	1293.868439	13/10/2021 12:34:14
Figure 303	40.379626	15.814641	902.621124	13/10/2021 16:14:19

Figure 305	40.405969	15.670955	1298.784038	11/10/2021 16:24:16
Figure 306	40.38478	15.767693	677.244375	12/10/2021 16:30:44
Figure 307	40.391163	15.813115	1052.964659	13/10/2021 11:07:32
Figure 308	40.38497	15.839545	1334.795949	13/10/2021 12:55:52
Figure 309	40.379101	15.815396	906.298004	13/10/2021 16:19:32
Figure 310	40.337382	15.893628	896.668121	14/10/2021 15:45:59
Figure 312	40.405864	15.670358	1301.078457	11/10/2021 16:26:02
Figure 313	40.384783	15.767717	677.78284	12/10/2021 16:30:58
Figure 314	40.384002	15.822514	965.891266	13/10/2021 11:22:54
Figure 315	40.384242	15.842544	1355.923859	13/10/2021 13:06:55
Figure 318	40.3842	15.843203	1346.01414	13/10/2021 13:09:10
Figure 319	40.378789	15.815654	904.907623	13/10/2021 16:32:22
Figure 320	40.40576	15.670189	1299.80126	11/10/2021 16:28:57
Figure 322	40.378558	15.815746	869.03421	13/10/2021 16:43:14
Figure 324	40.383839	15.844608	1367.450348	13/10/2021 13:15:07
Figure 325	40.40568	15.670068	1288.827991	11/10/2021 16:30:54
Figure 326	40.386828	15.836113	1281.43495	13/10/2021 14:19:29
Figure 327	40.405568	15.669942	1284.218716	11/10/2021 16:34:16
Figure 328	40.405568	15.669942	1284.218716	11/10/2021 16:34:25

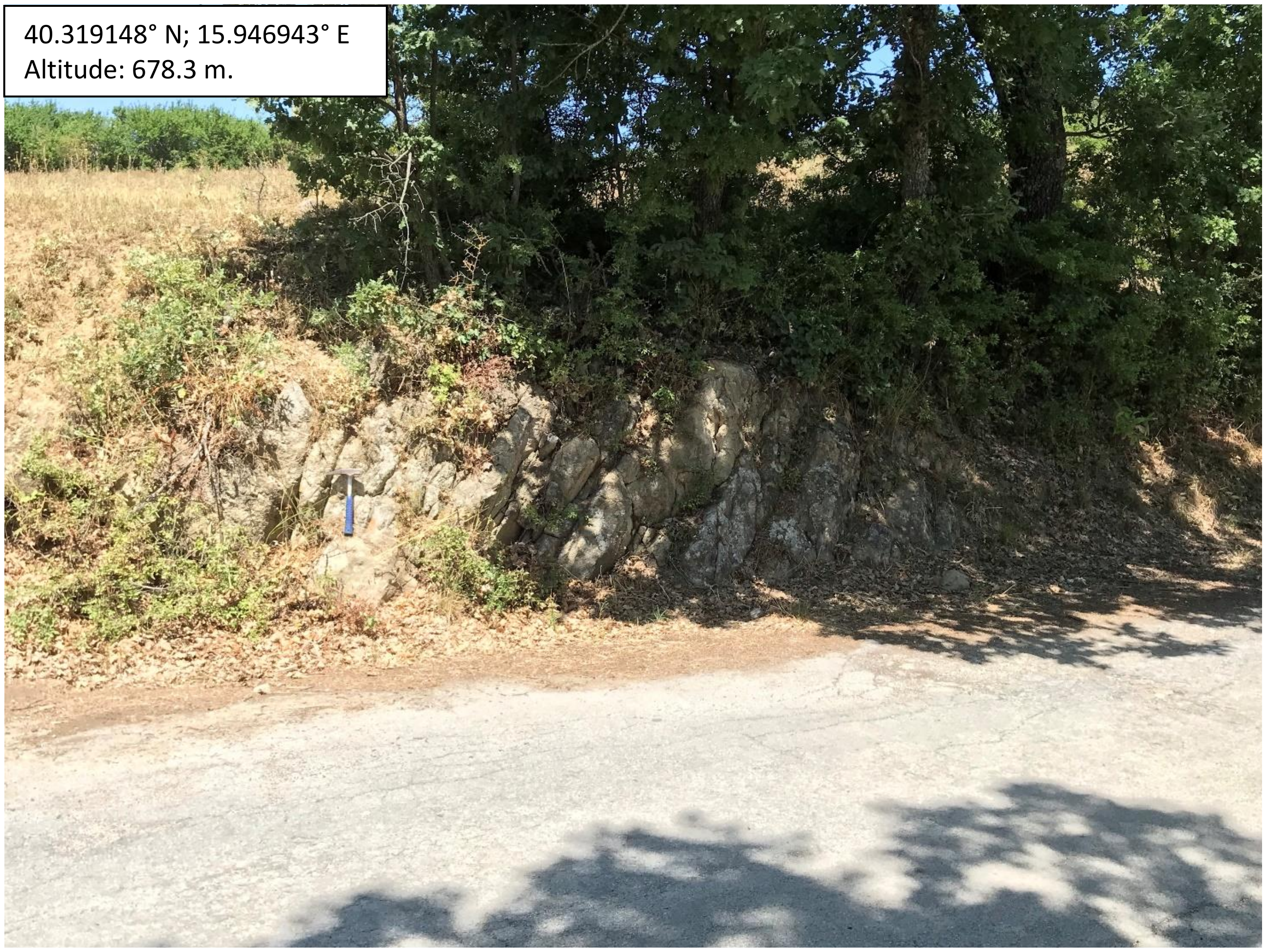

Figure 1.

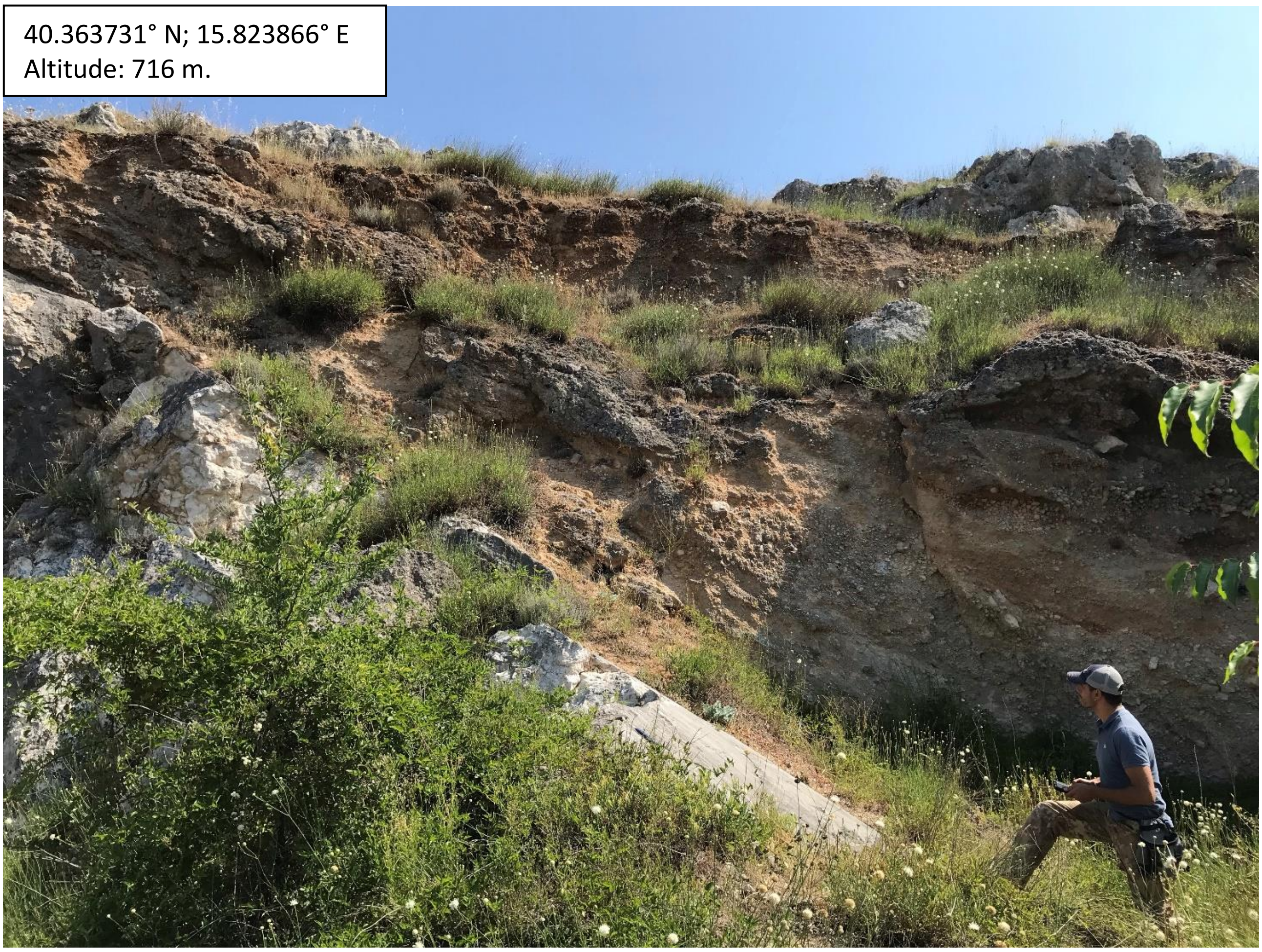

Figure 1.

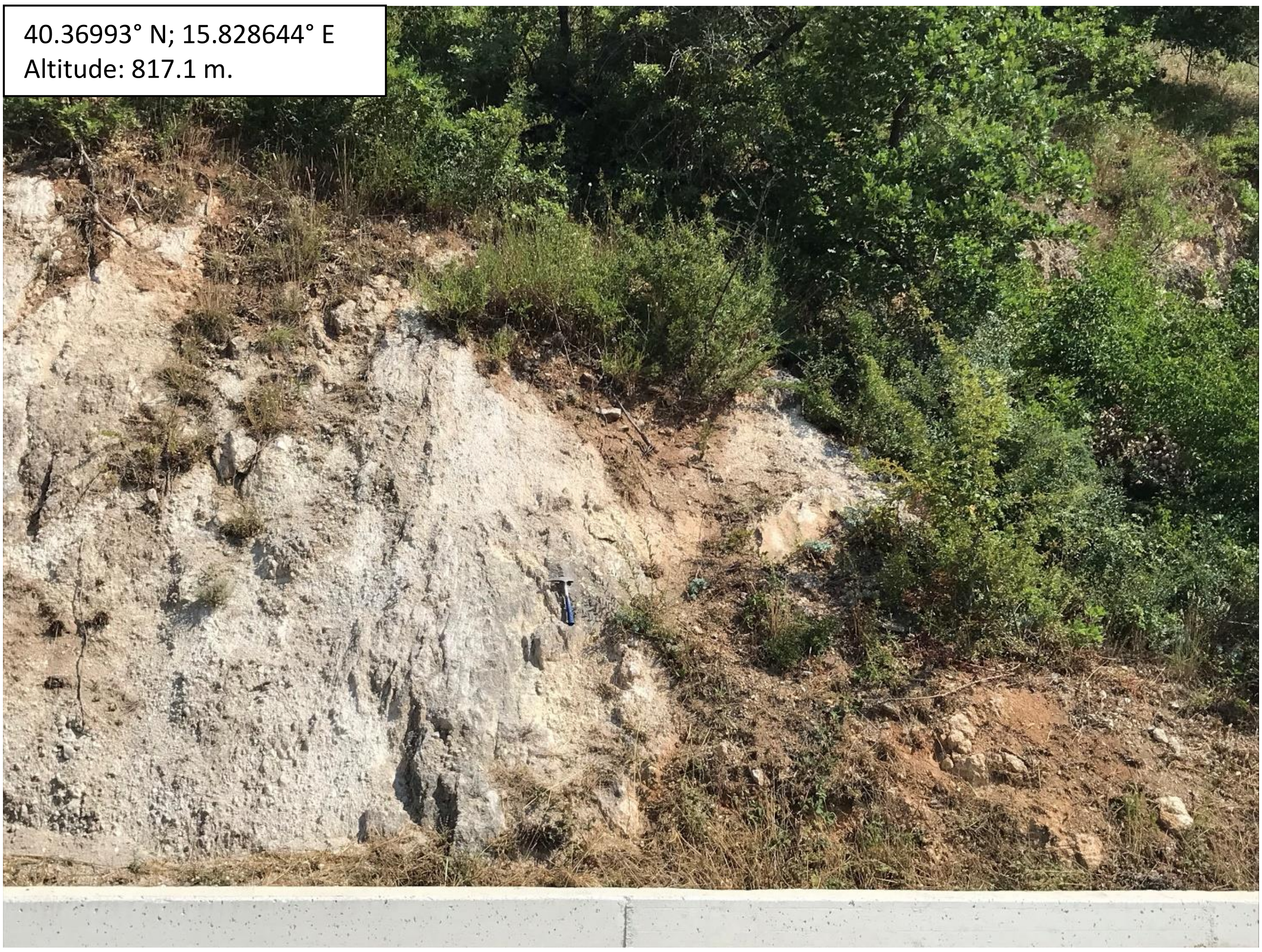

Figure 2.

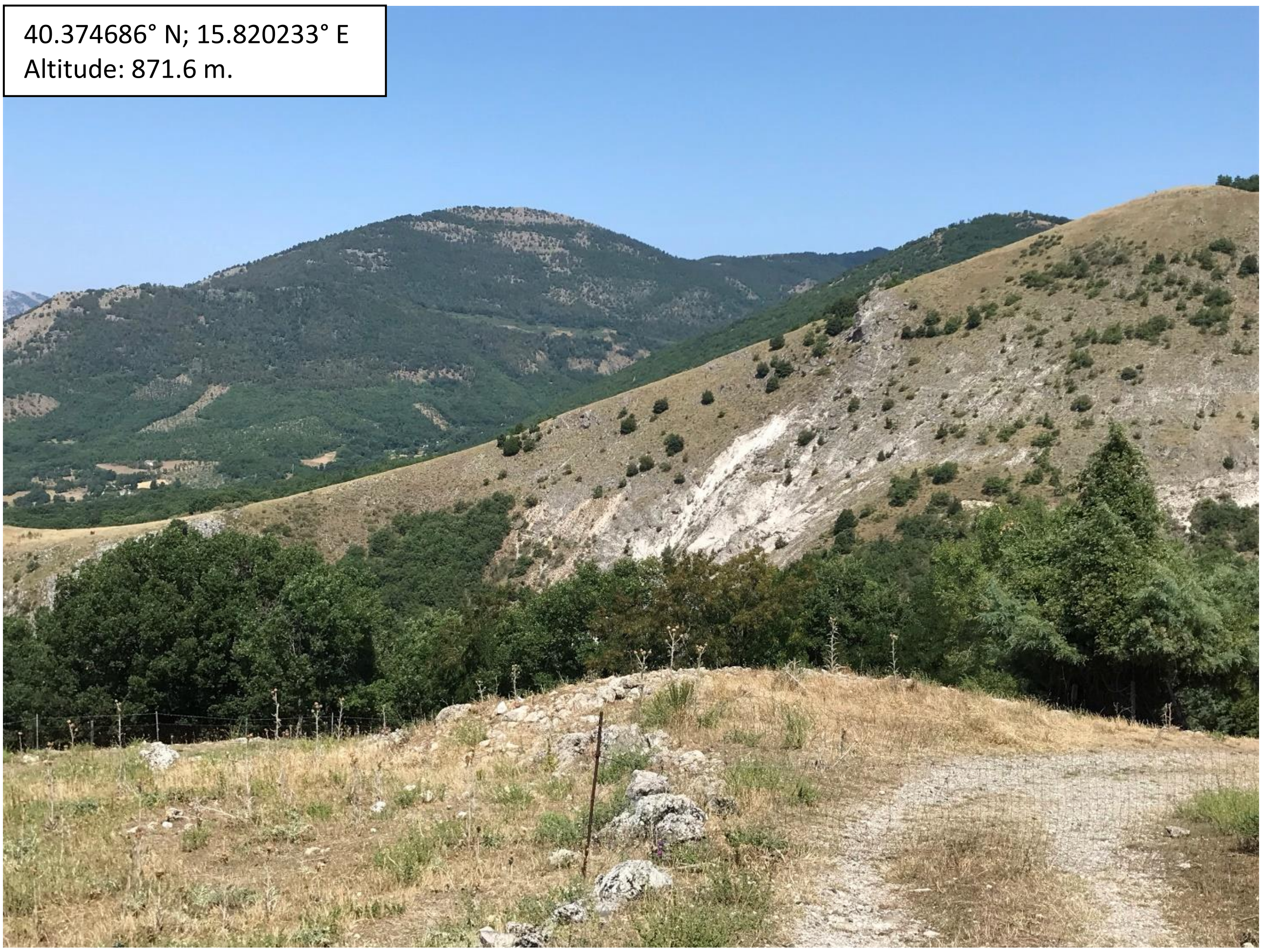

Figure 3.

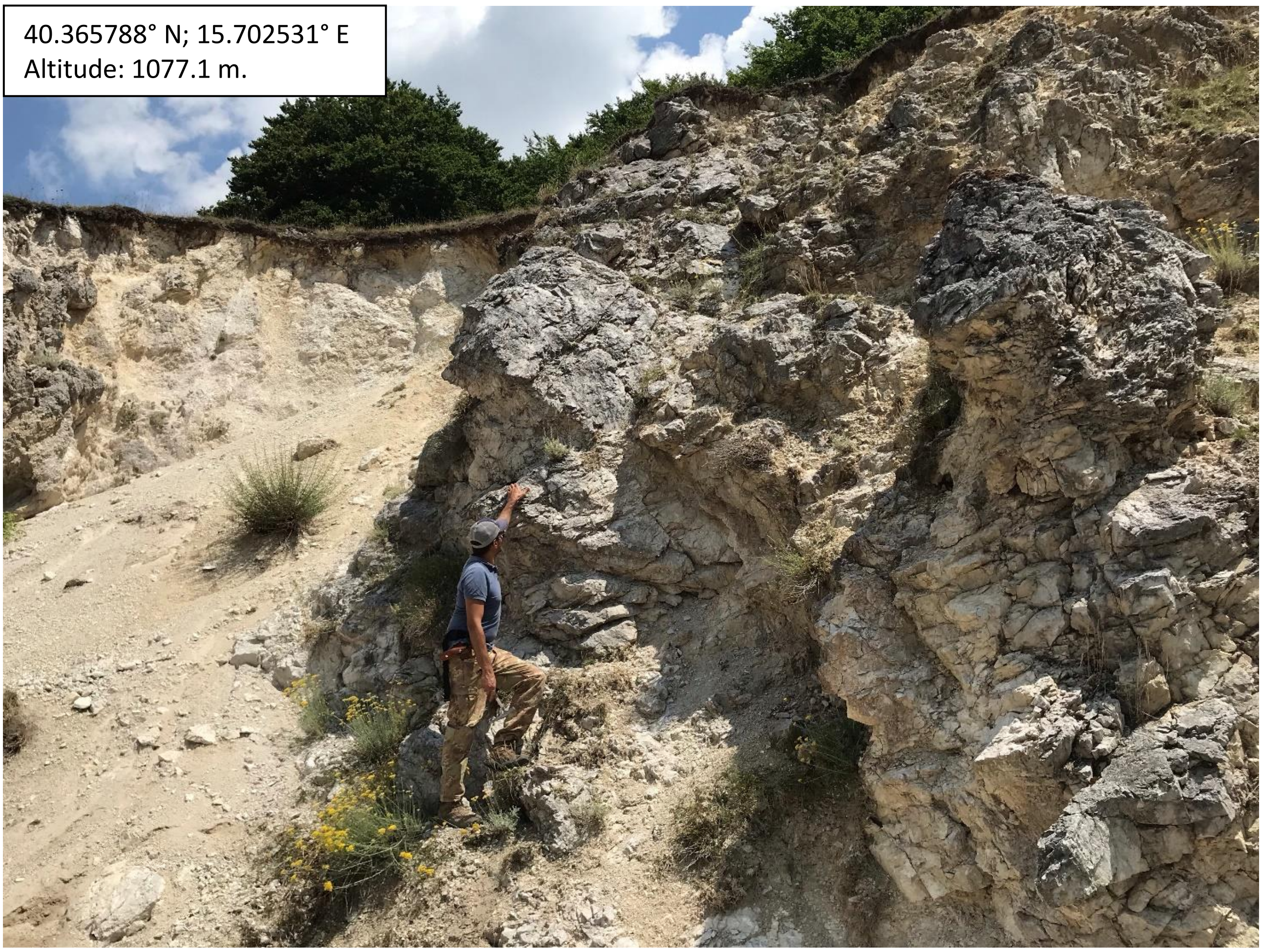

Figure 4.

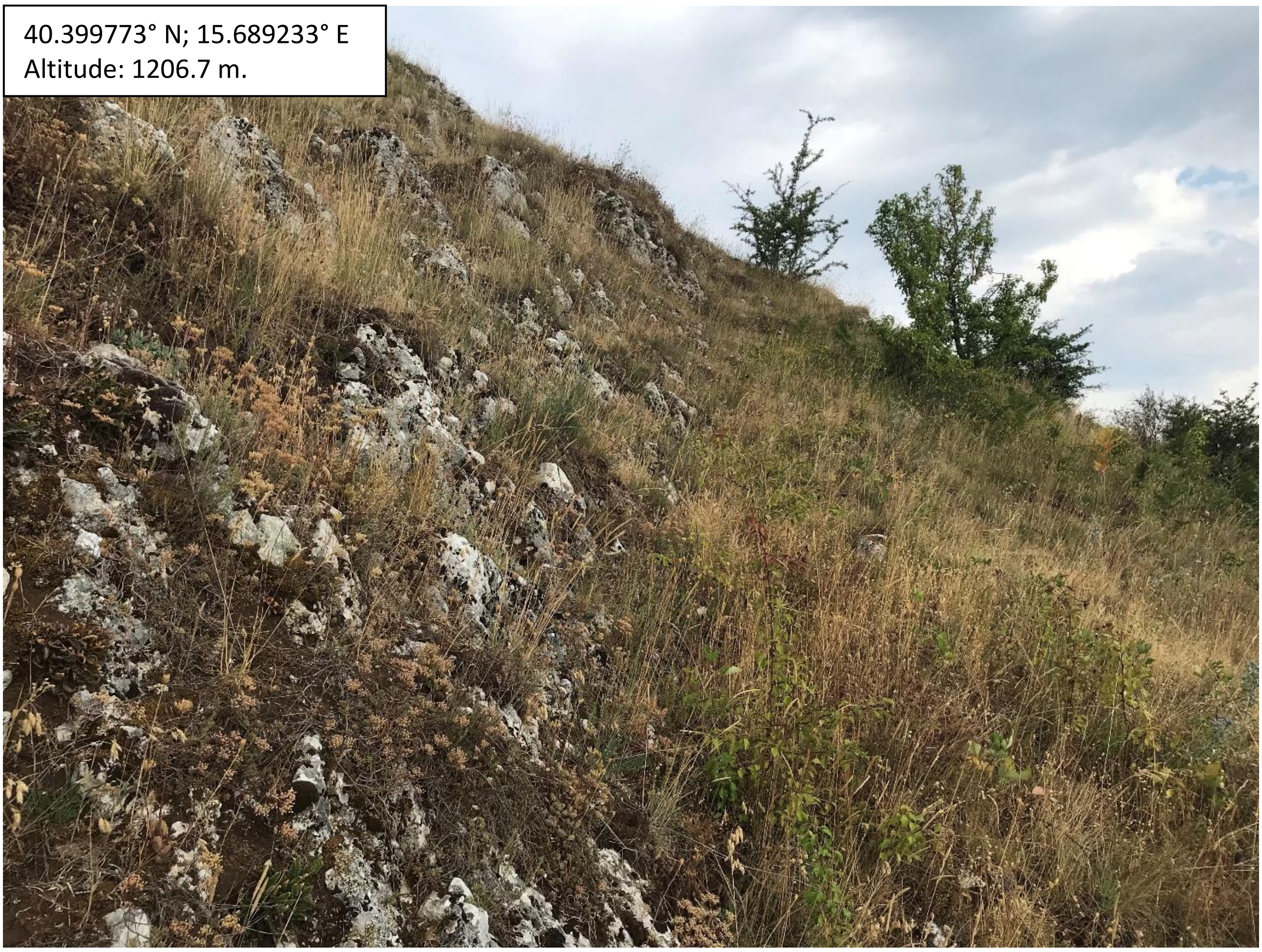

Figure 5.

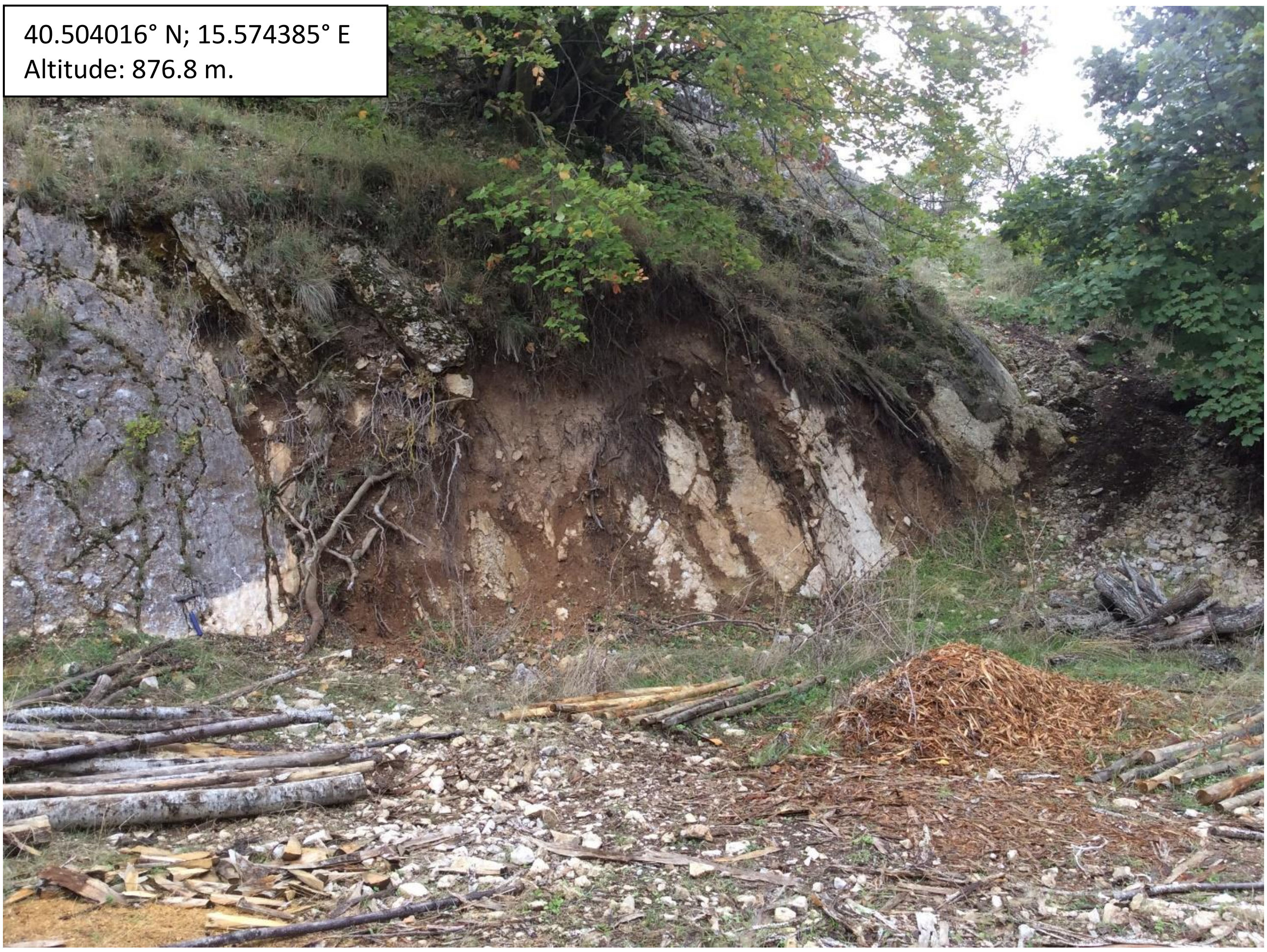

Figure 6.

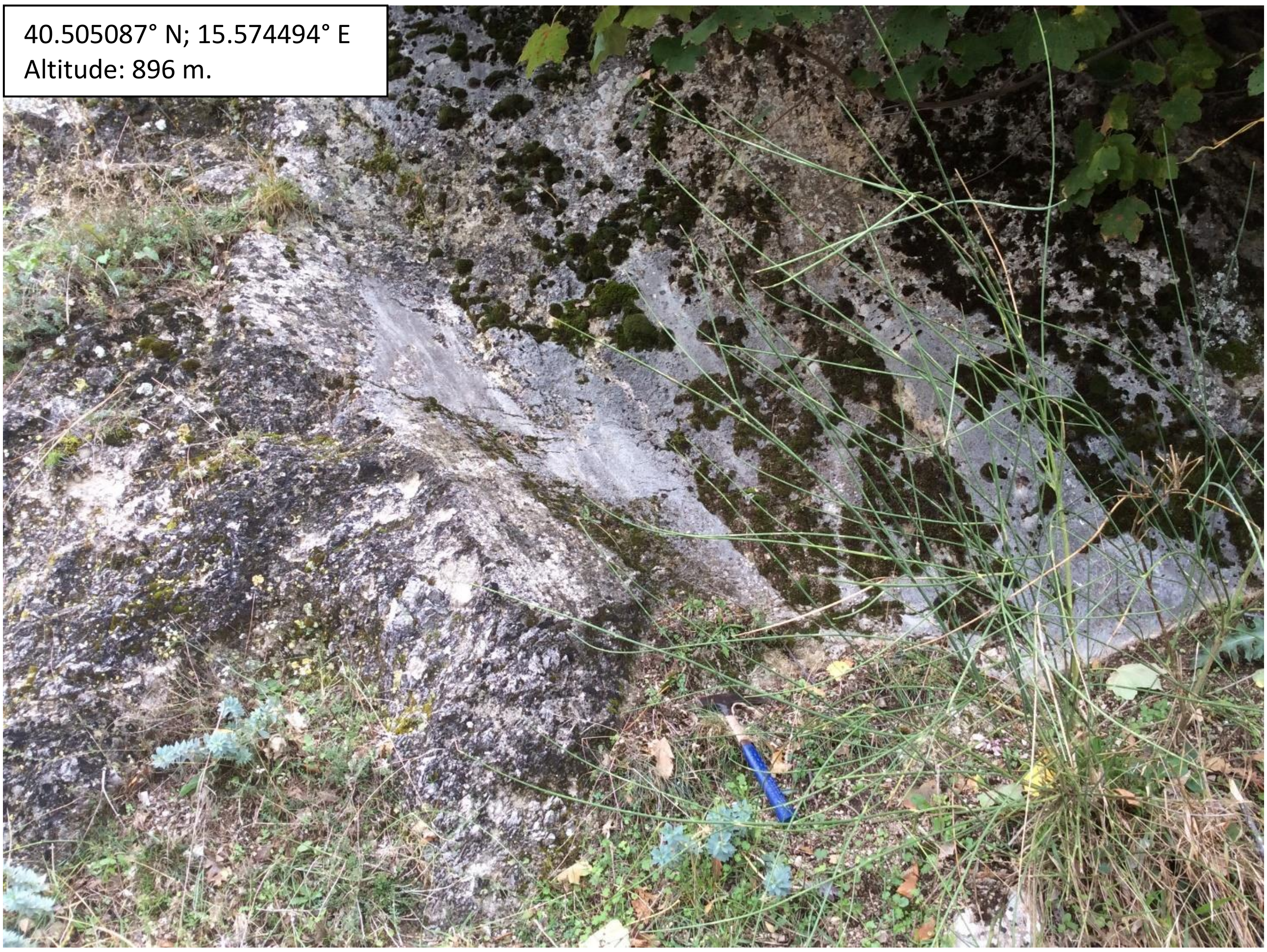

Figure 7.

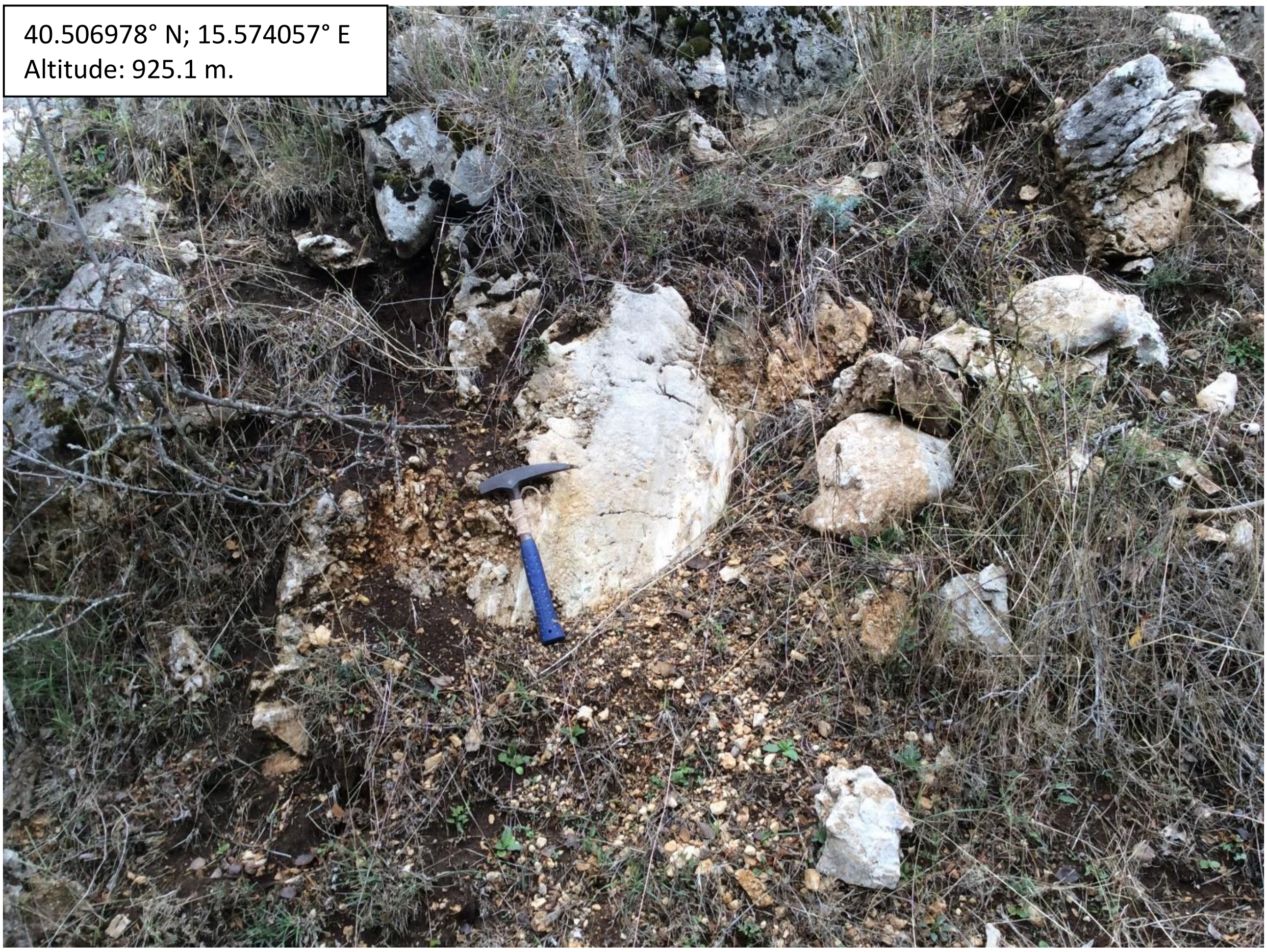

Figure 8.

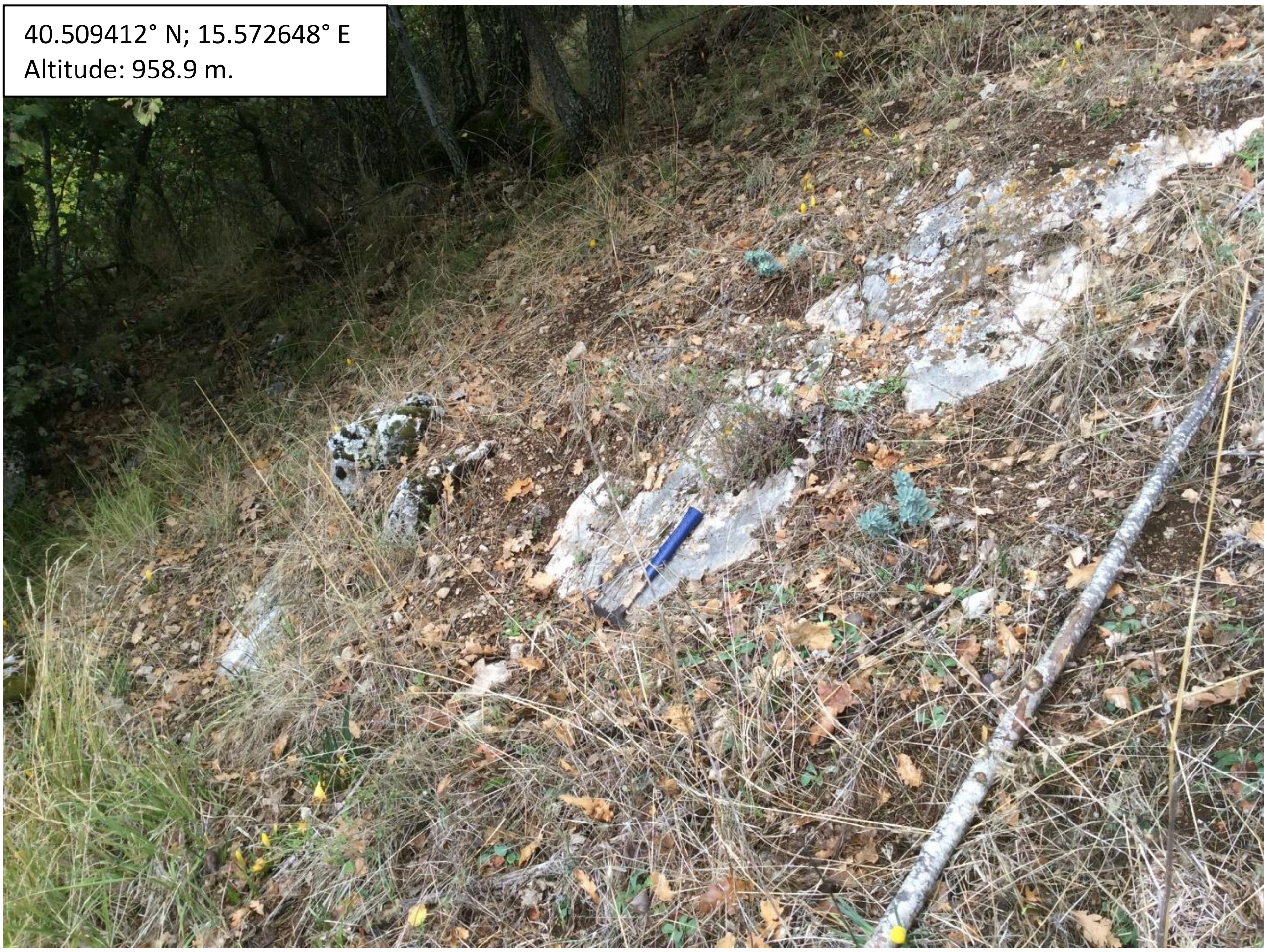

Figure 9.

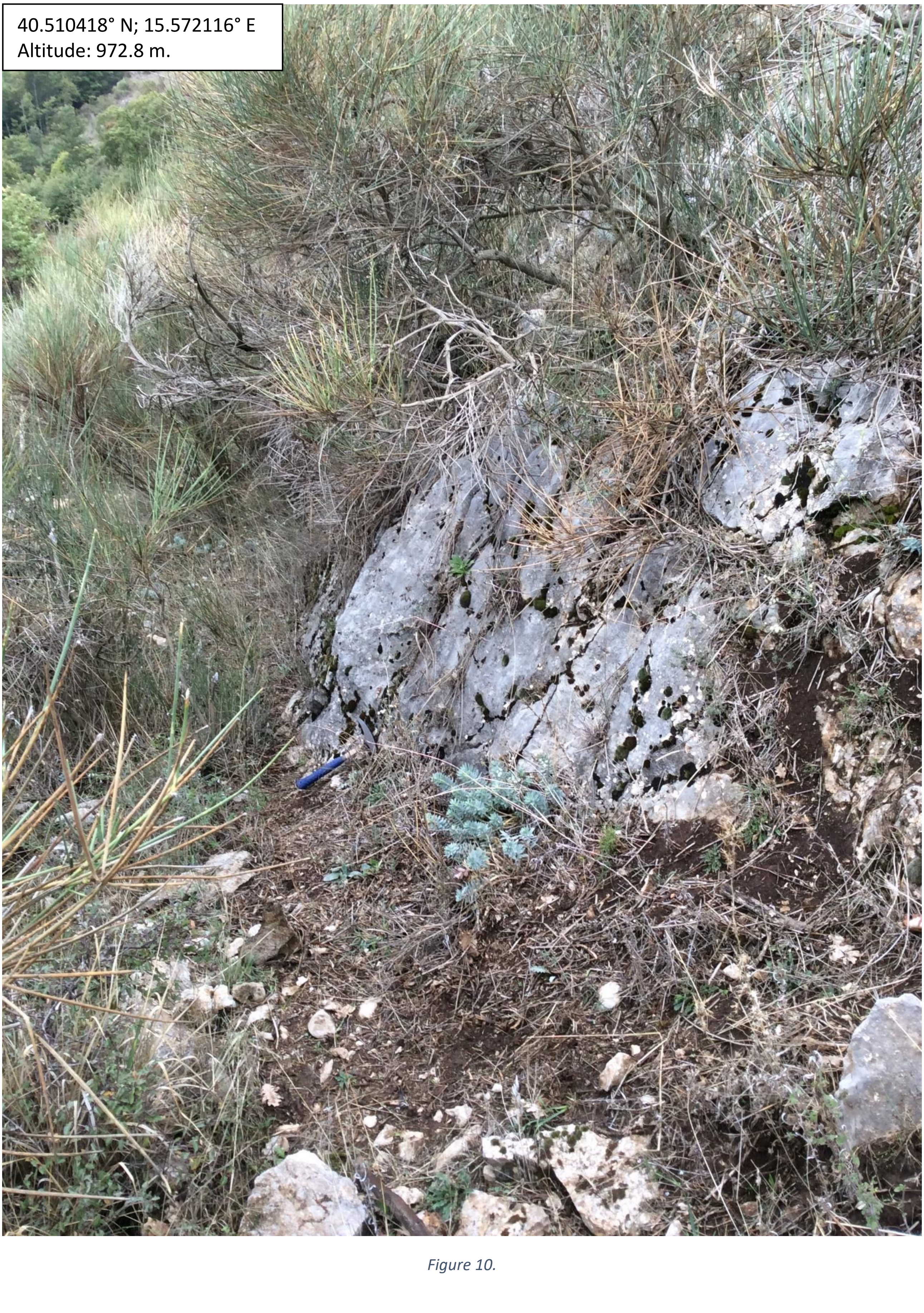

Figure 10.

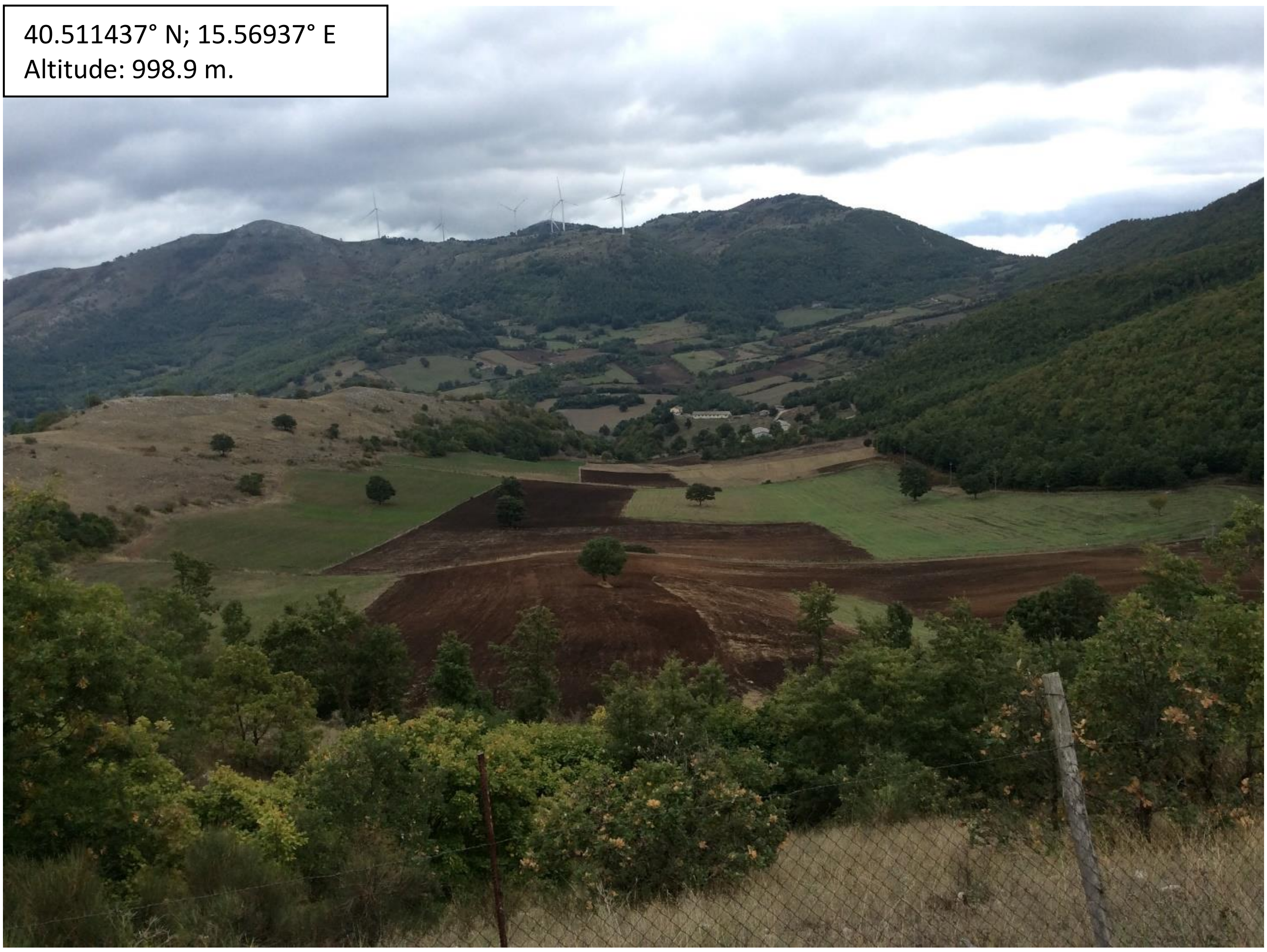

Figure 11.

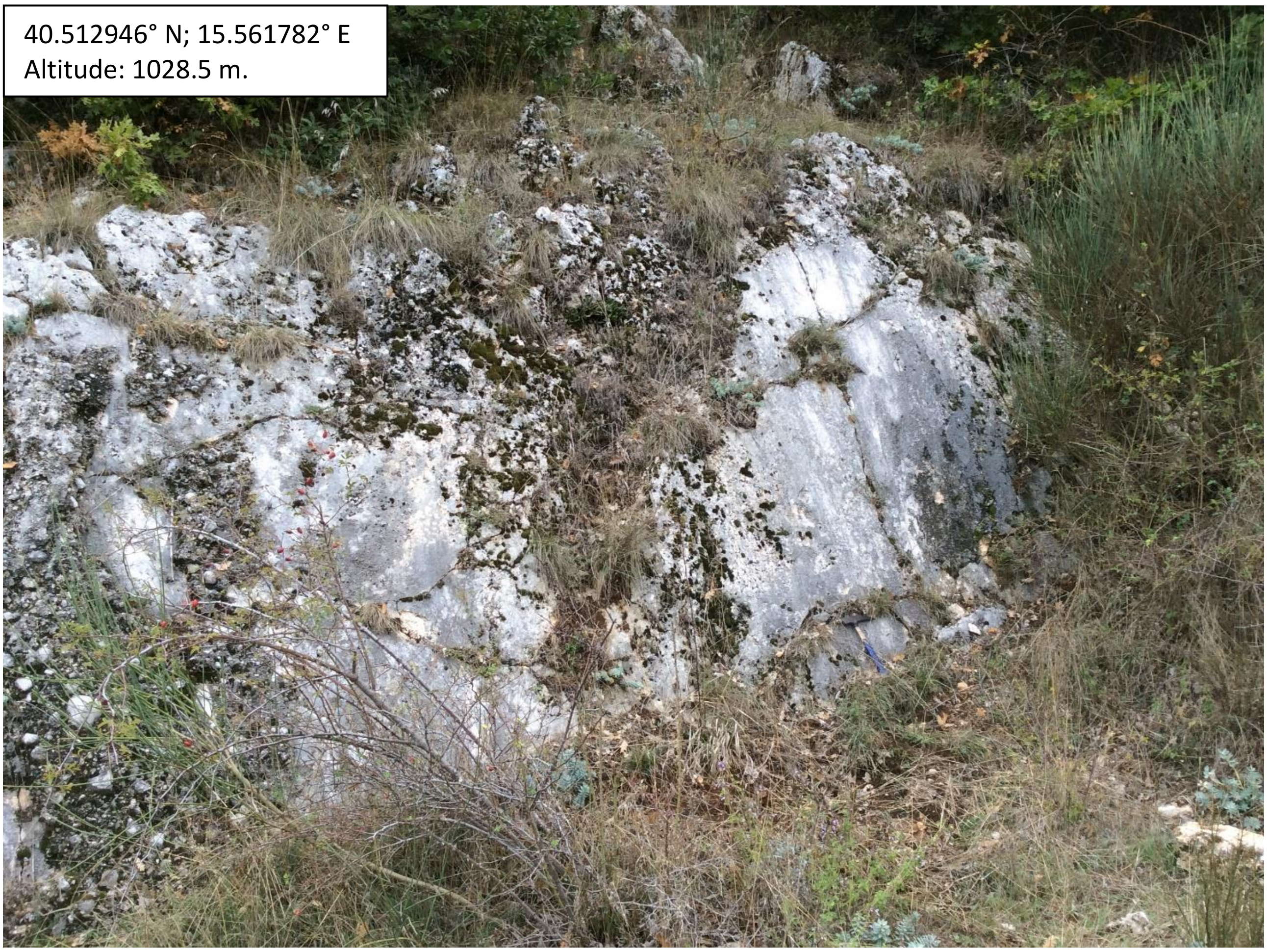

Figure 12.

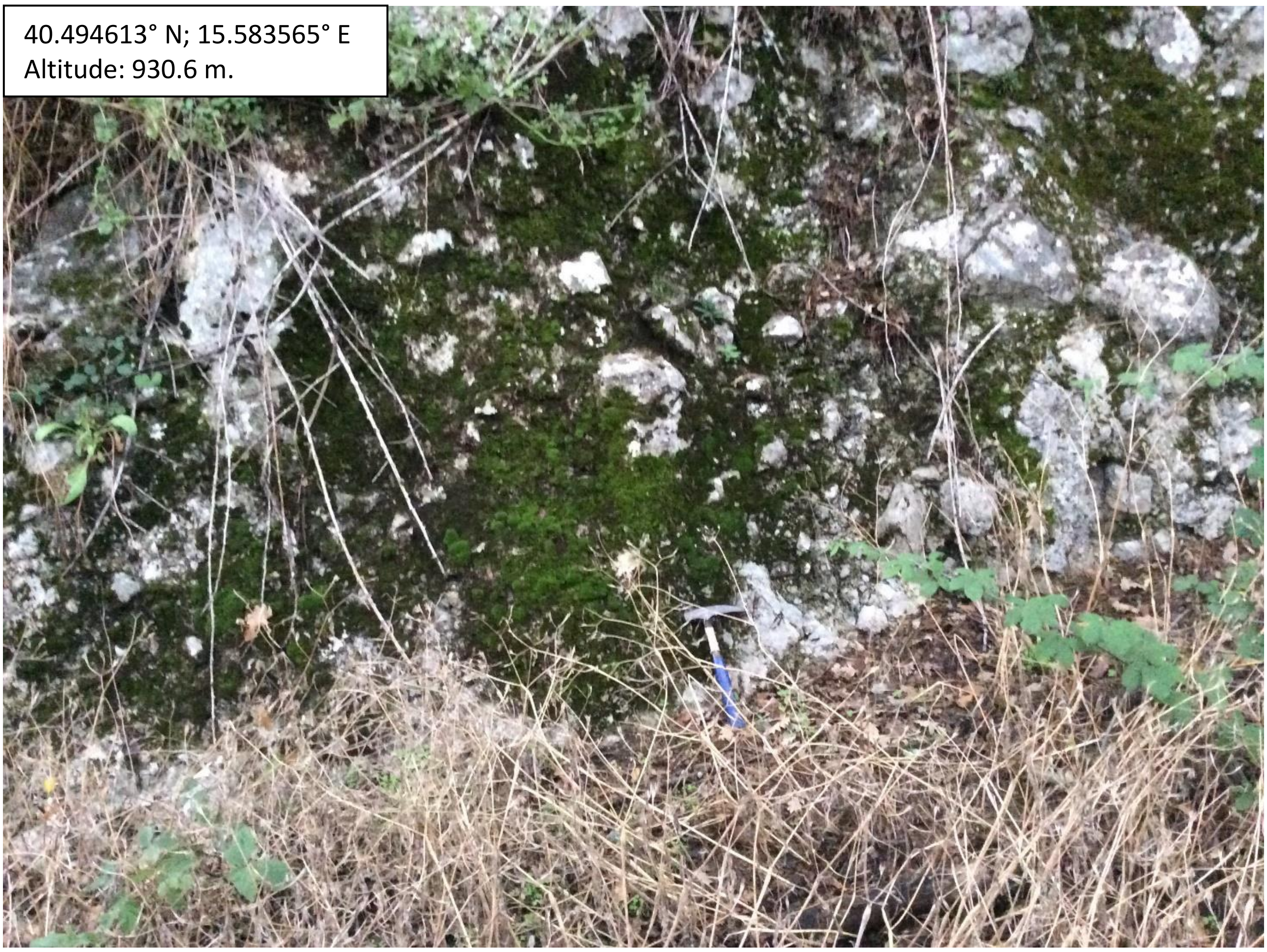

Figure 13.

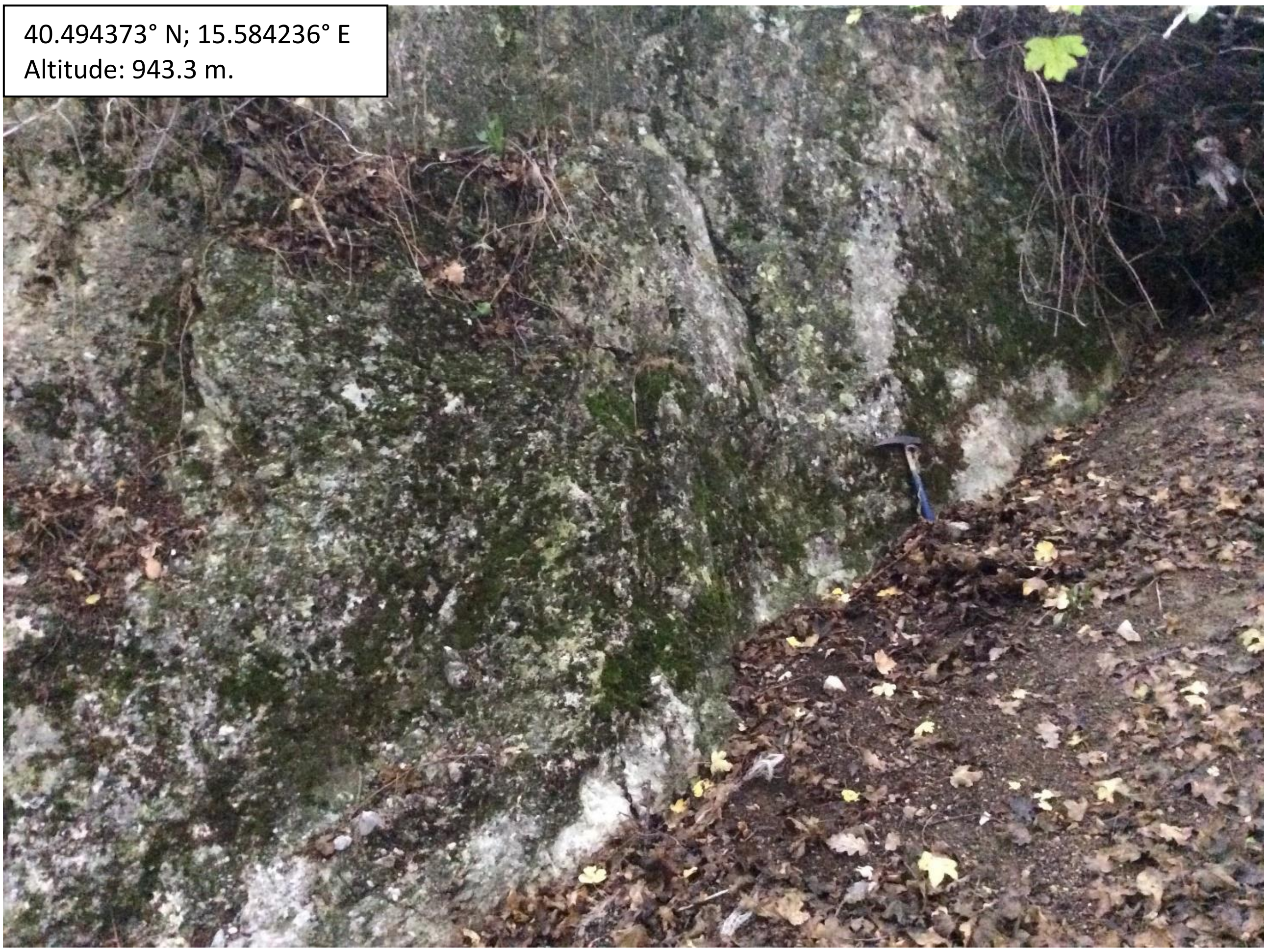

Figure 14.

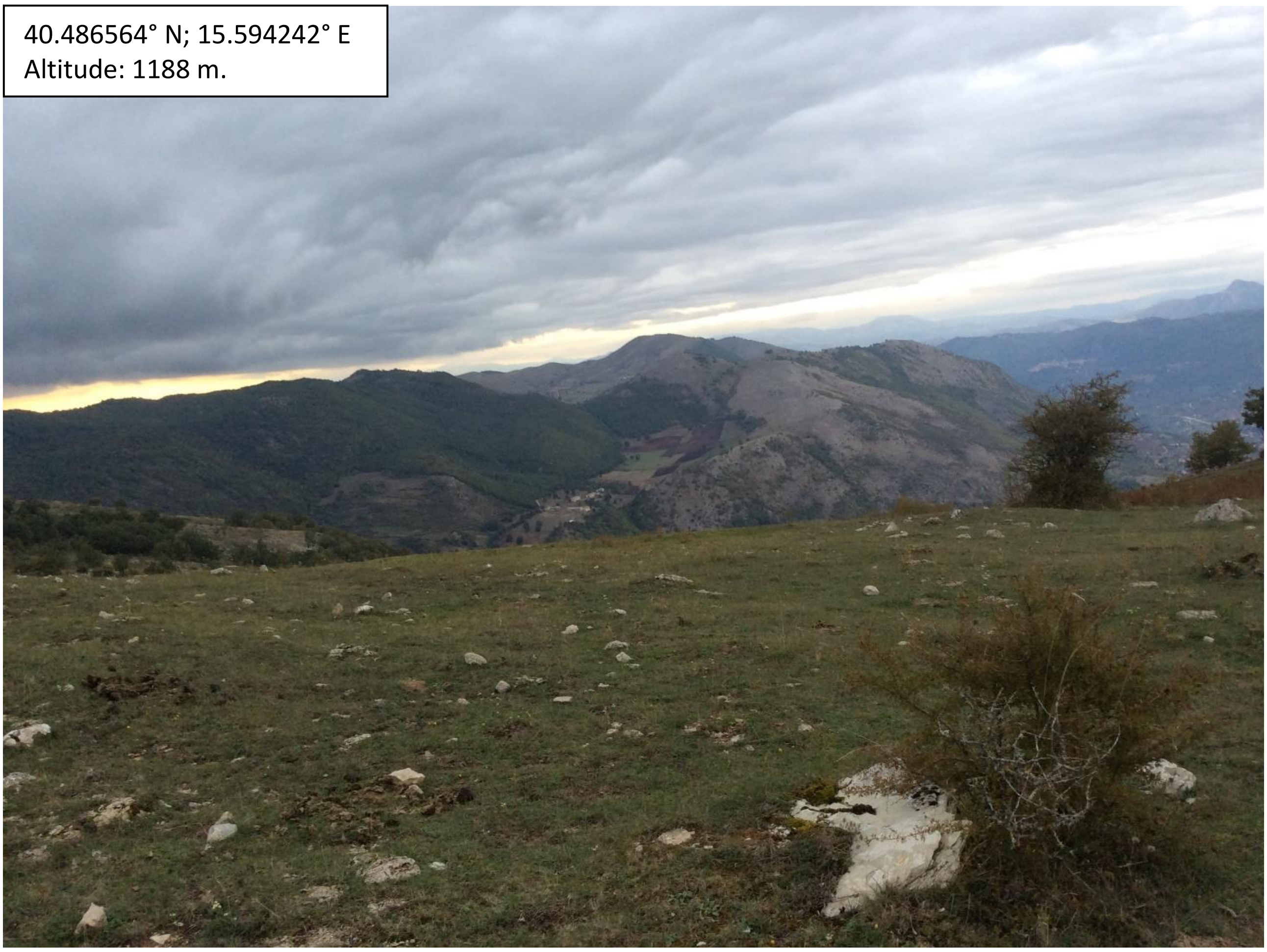

Figure 15.

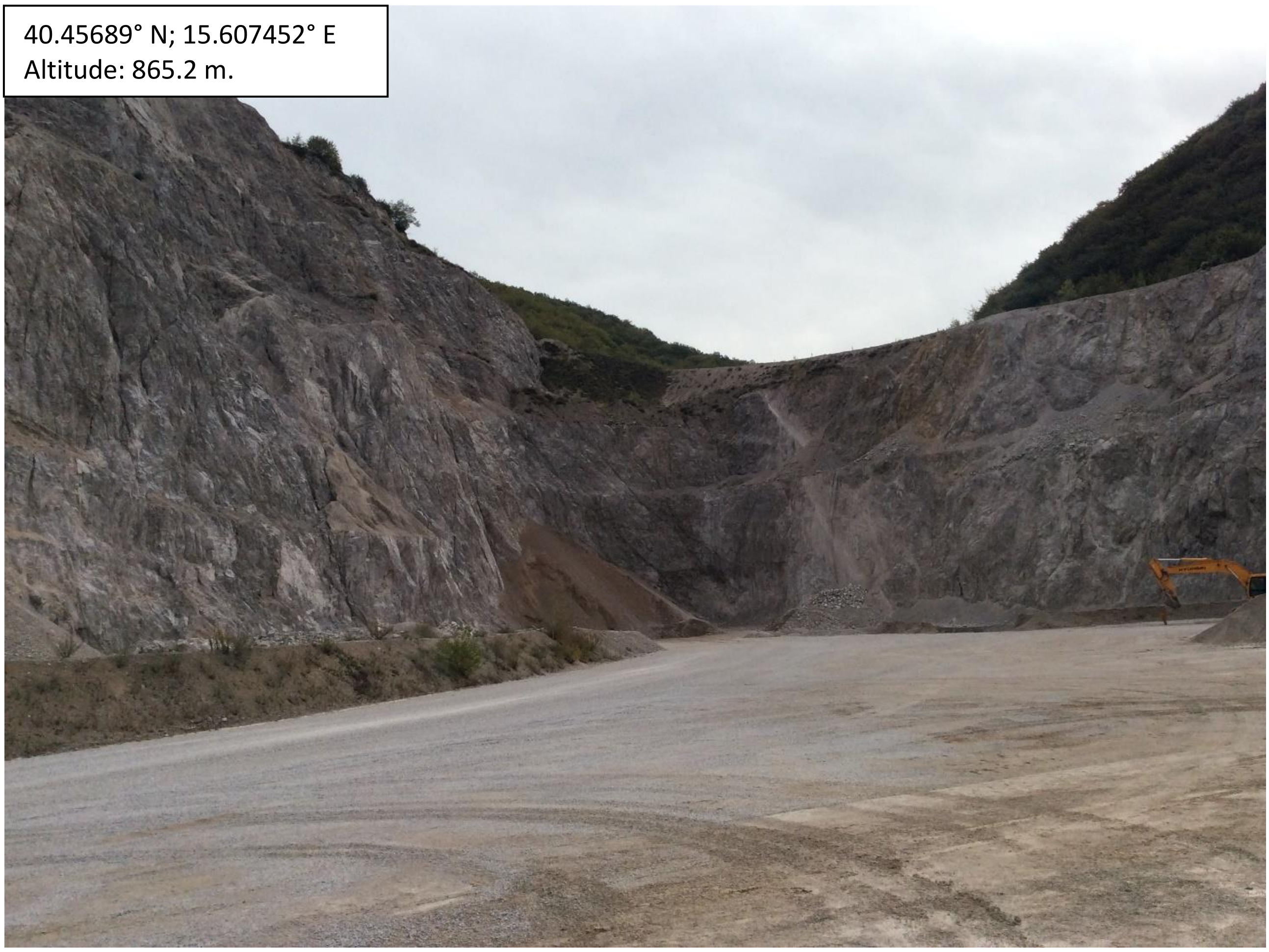

Figure 16.

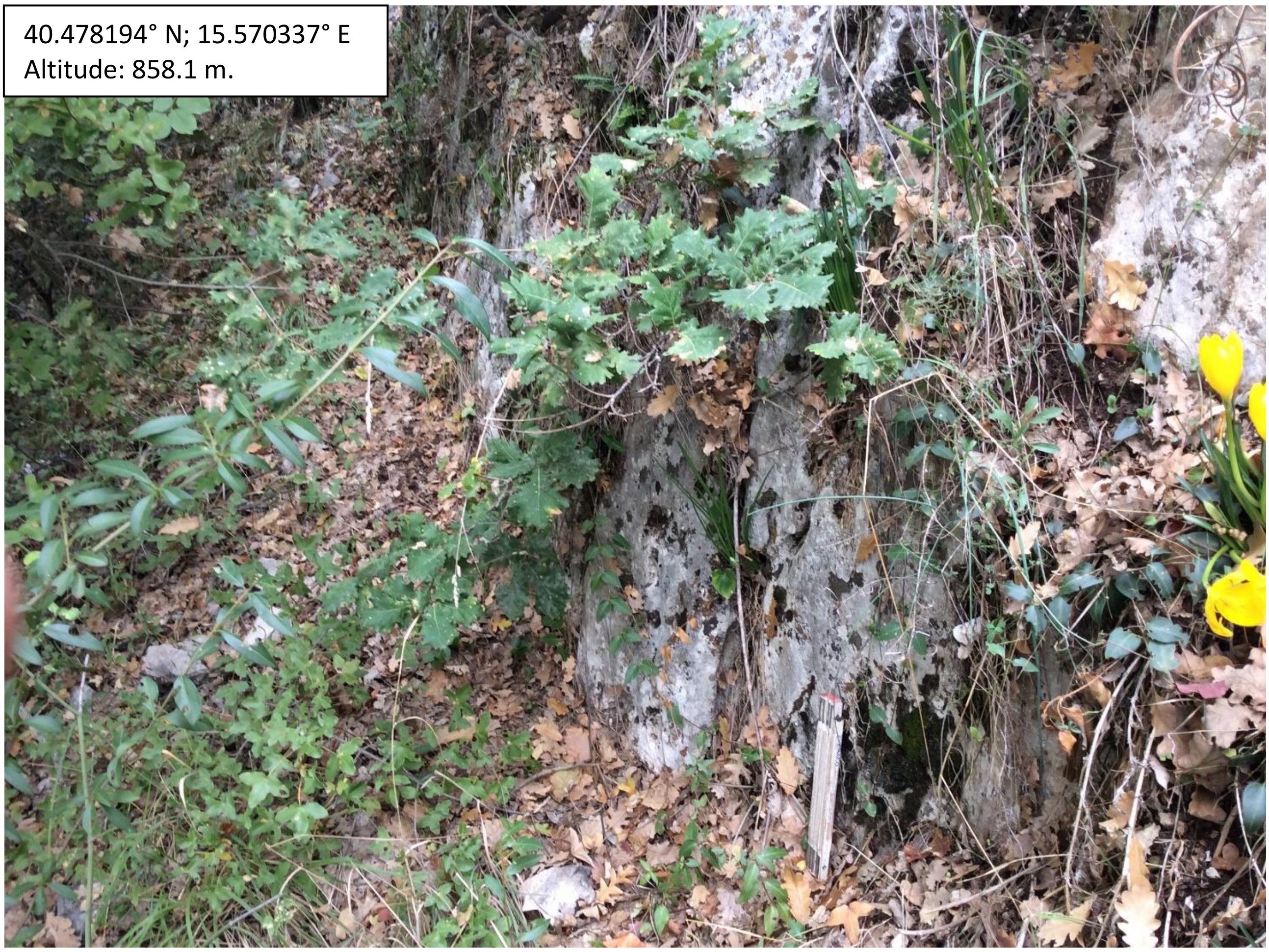

Figure 17.

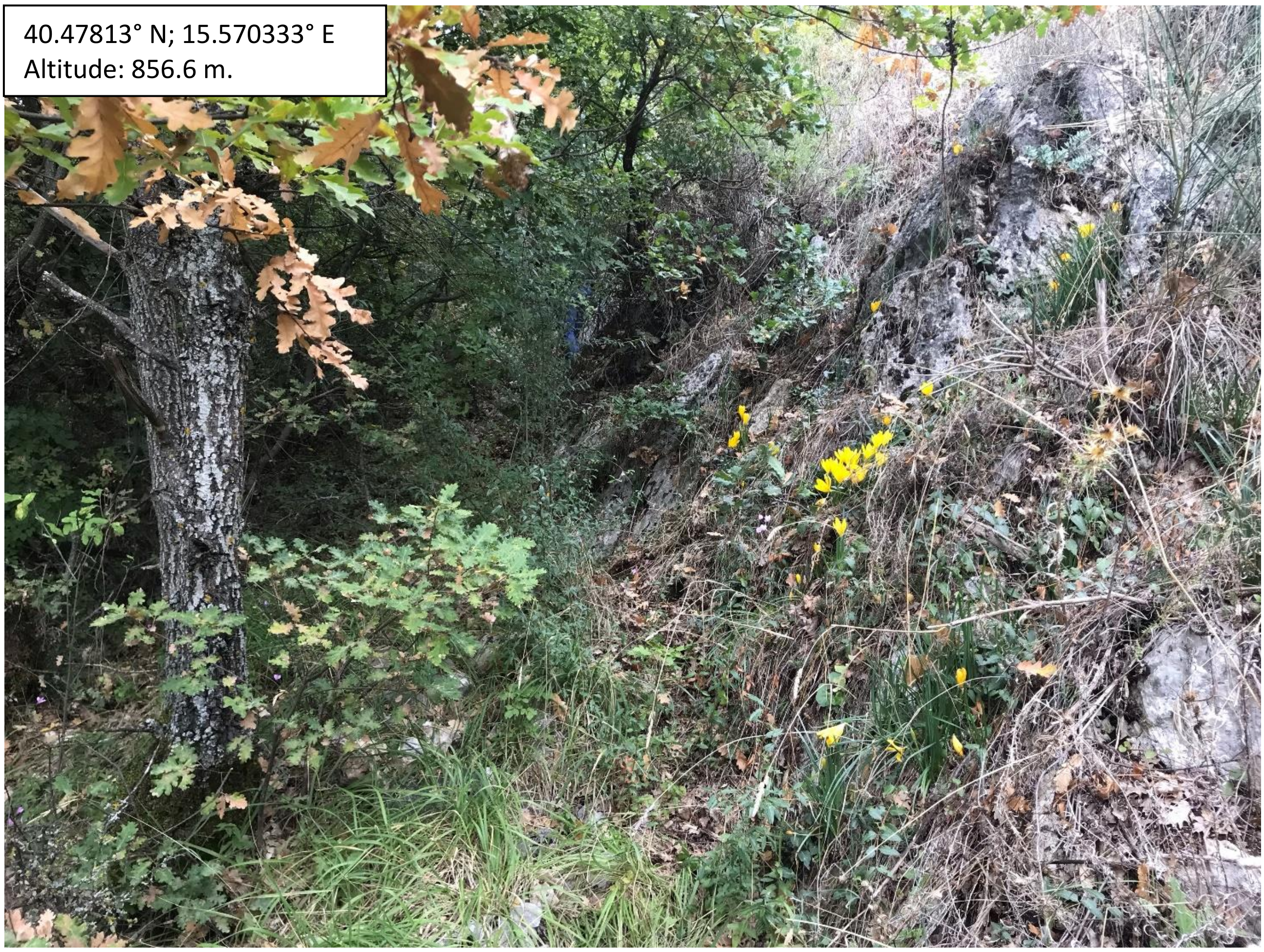

Figure 18.

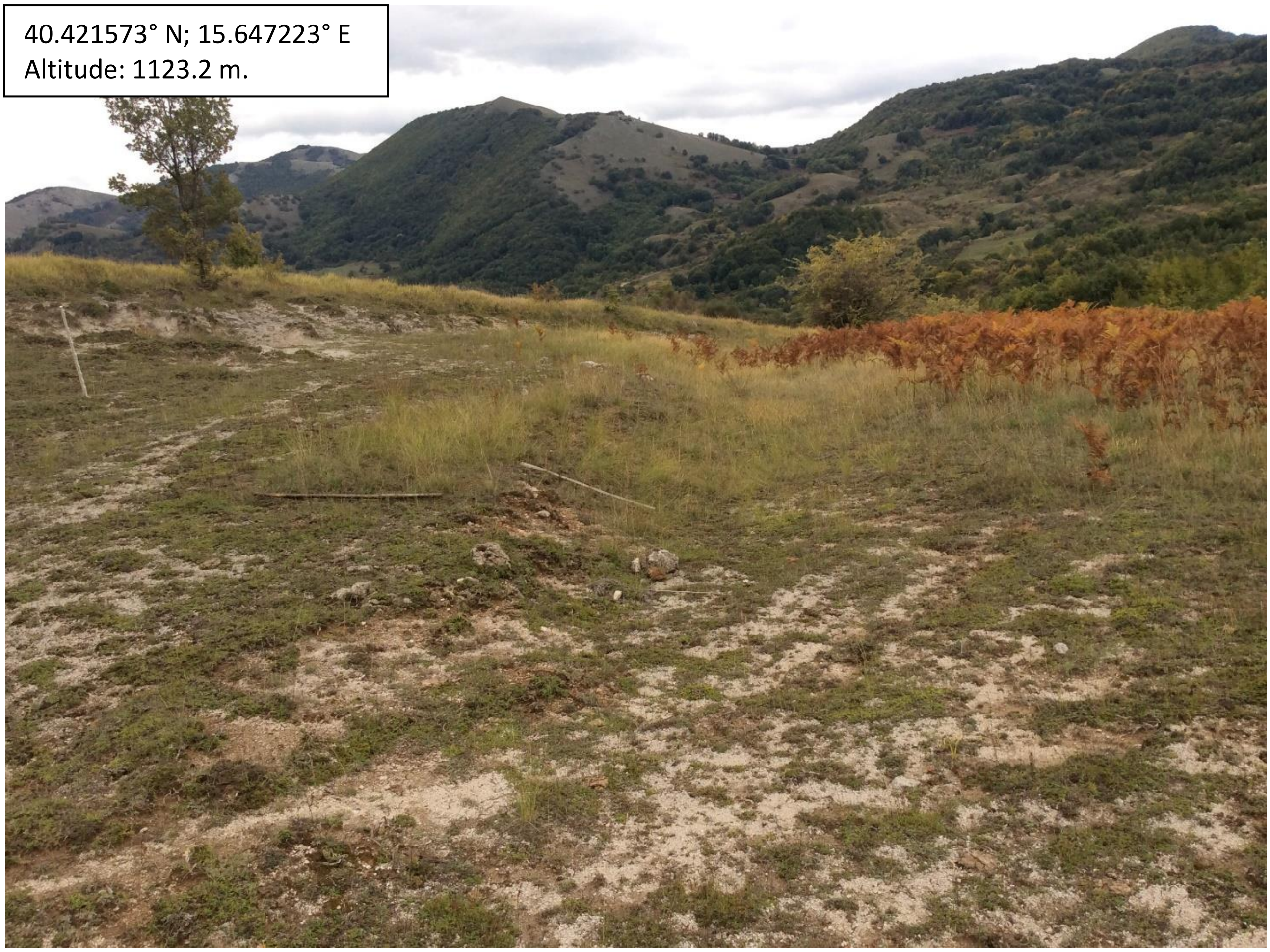

Figure 19.

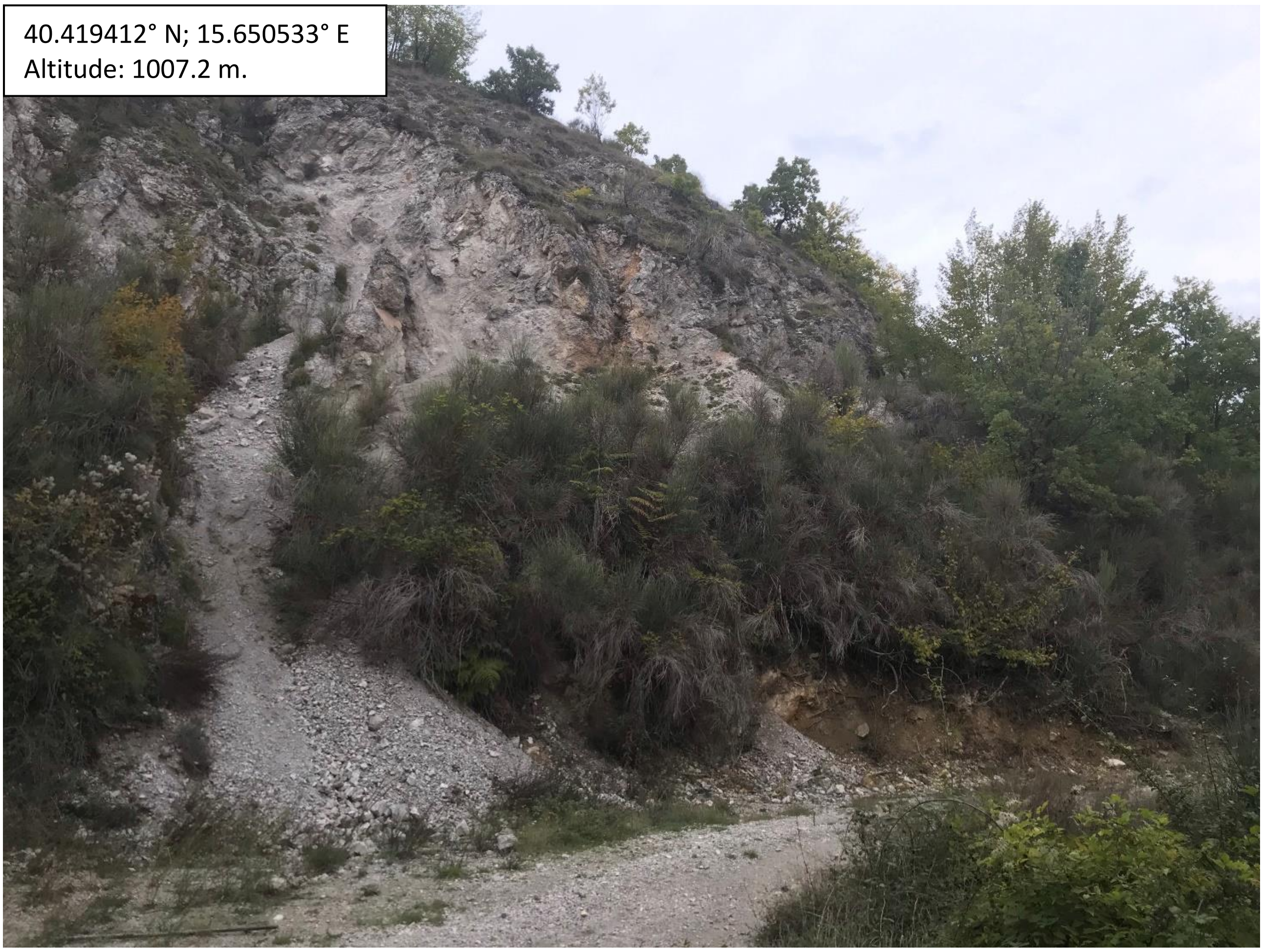

Figure 20.

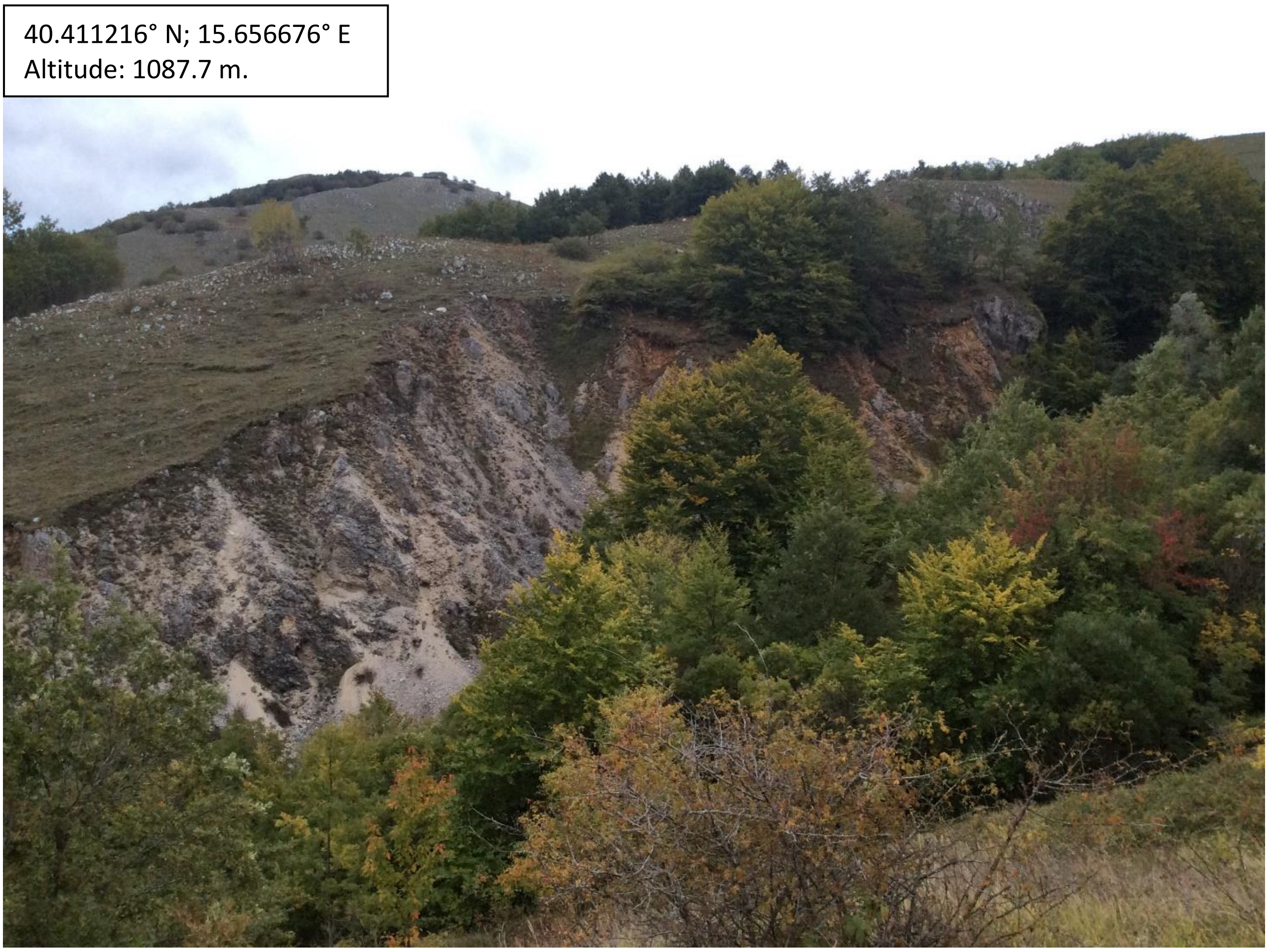

Figure 21.

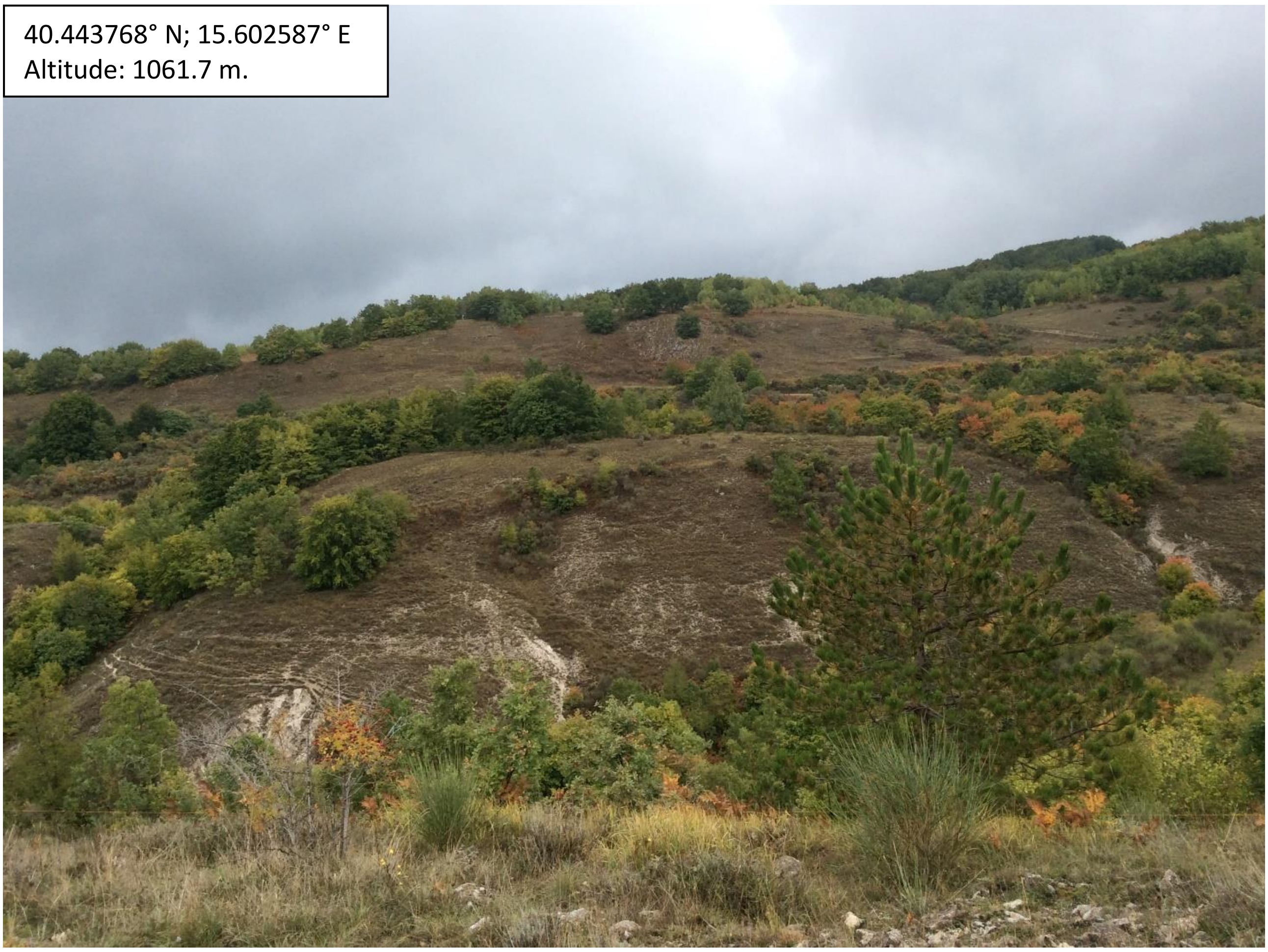

Figure 22.

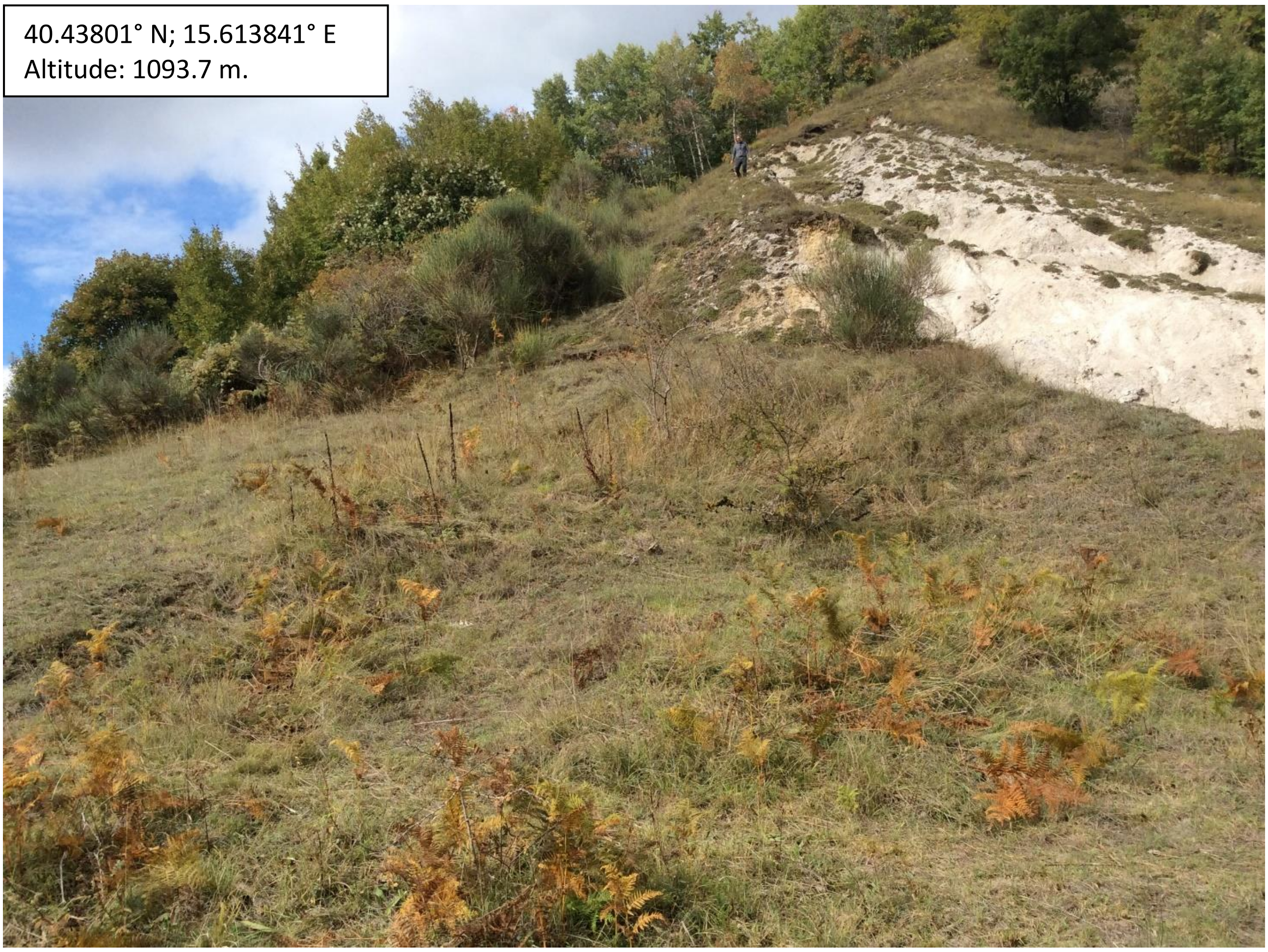

Figure 23.

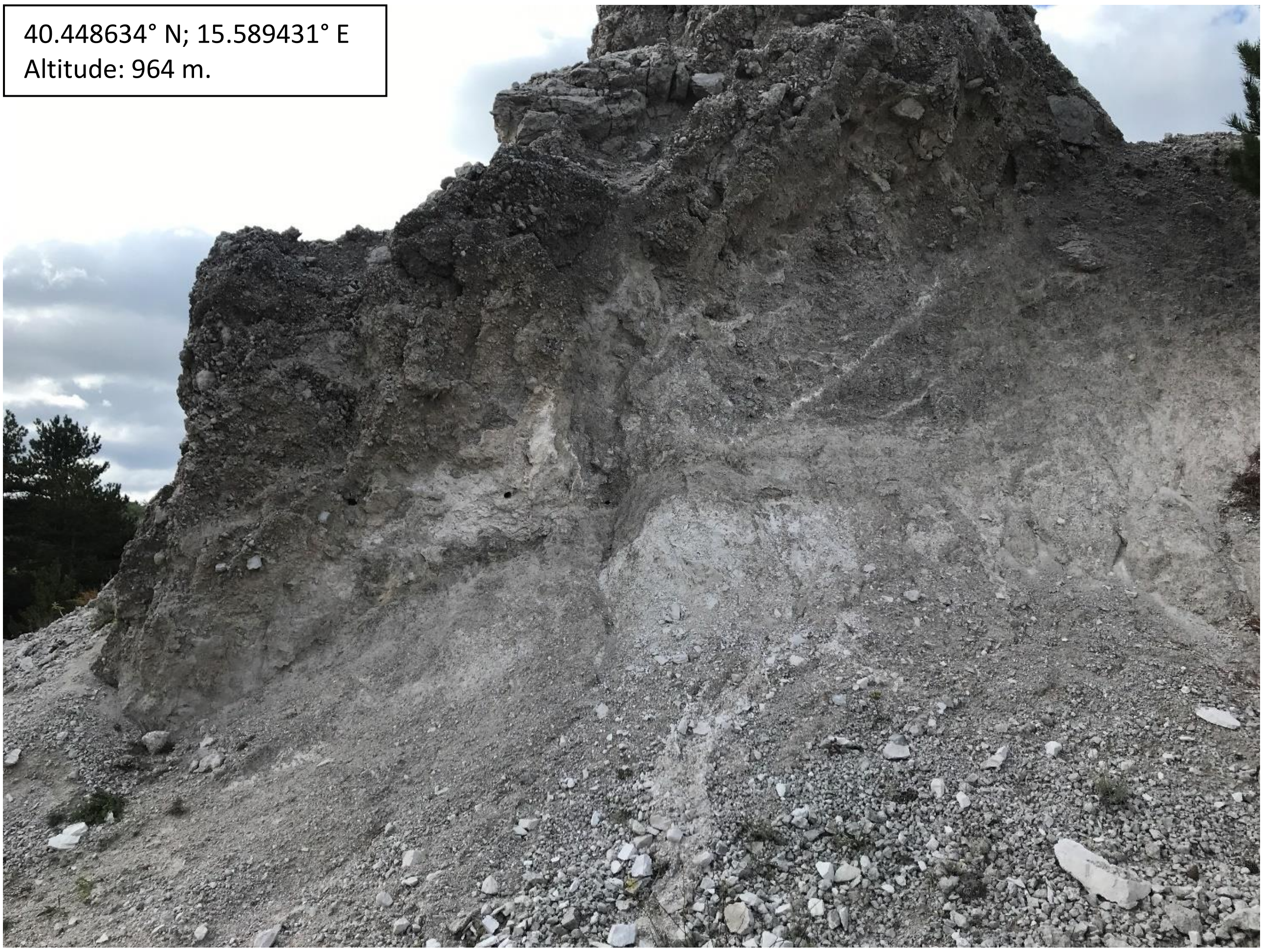

Figure 24.

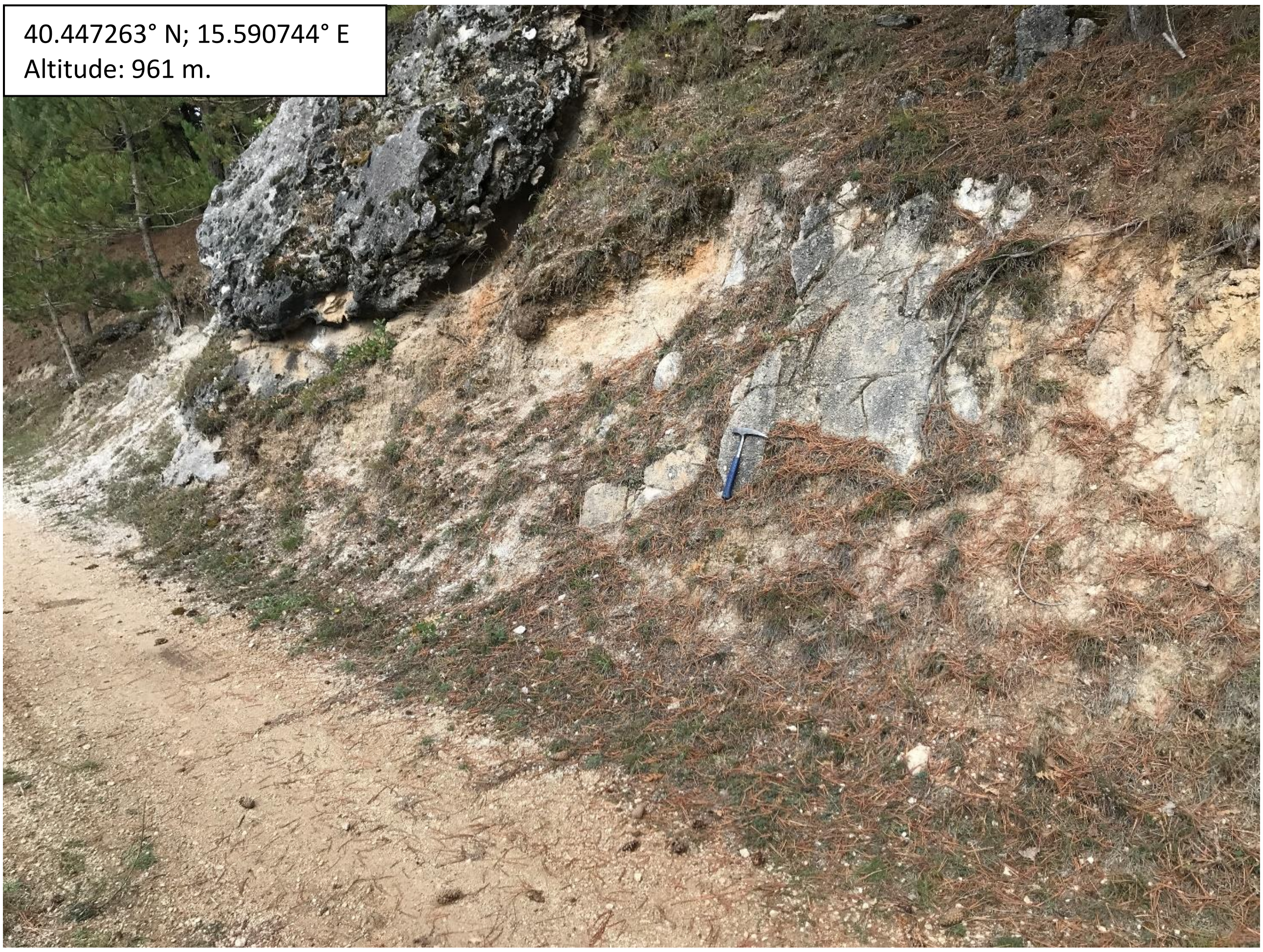

Figure 25.

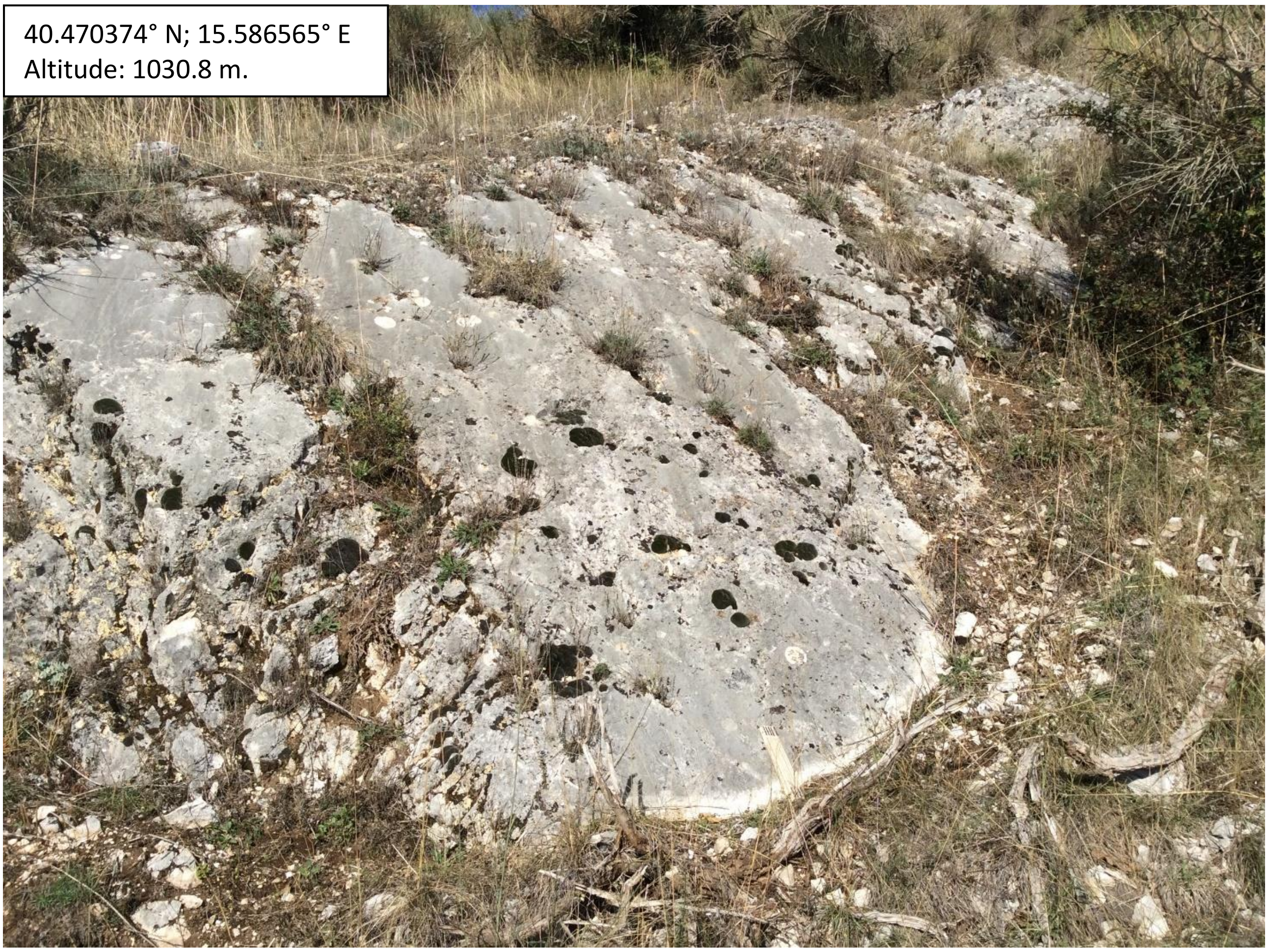

Figure 26.

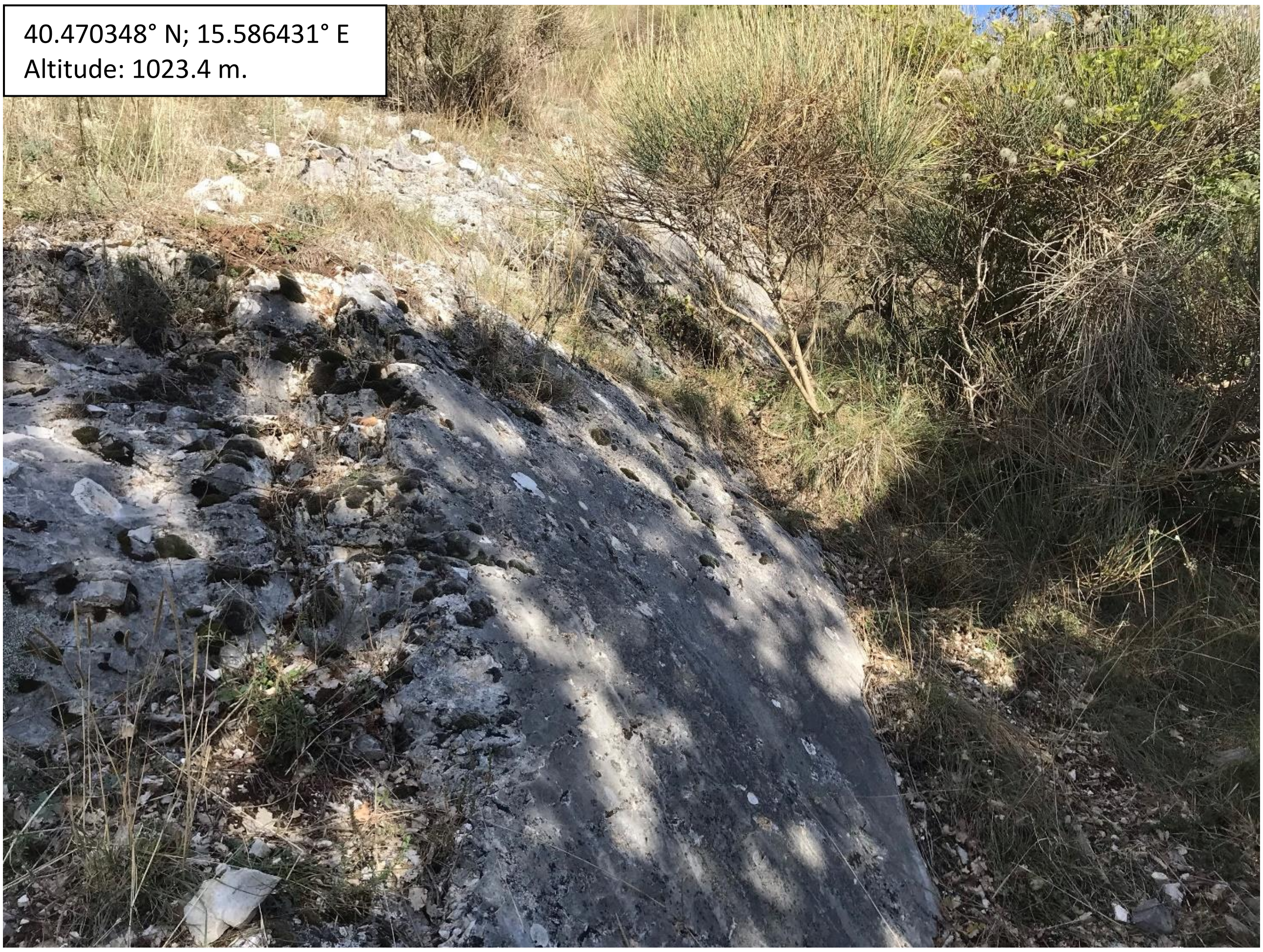

Figure 27.

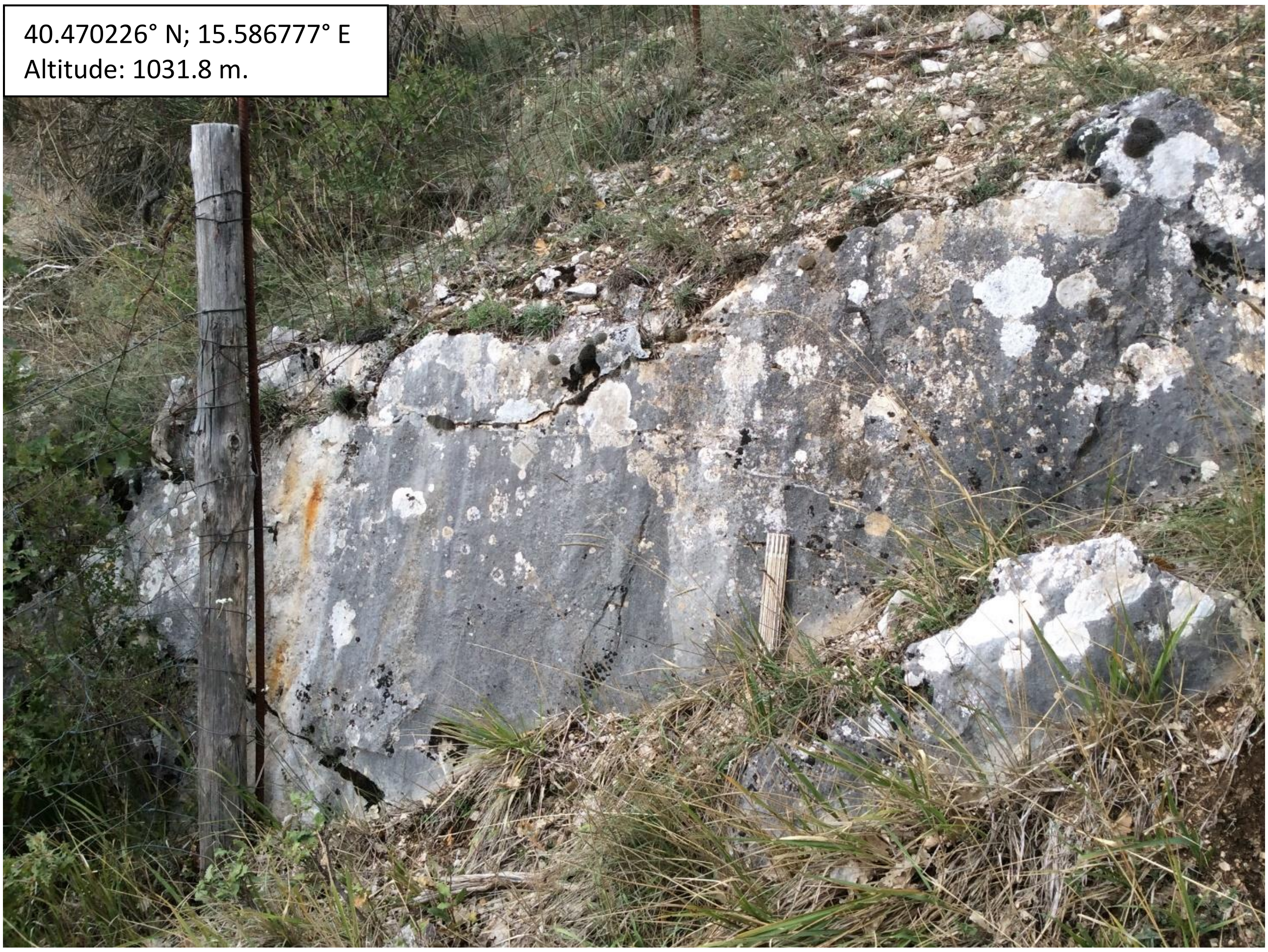

Figure 28.

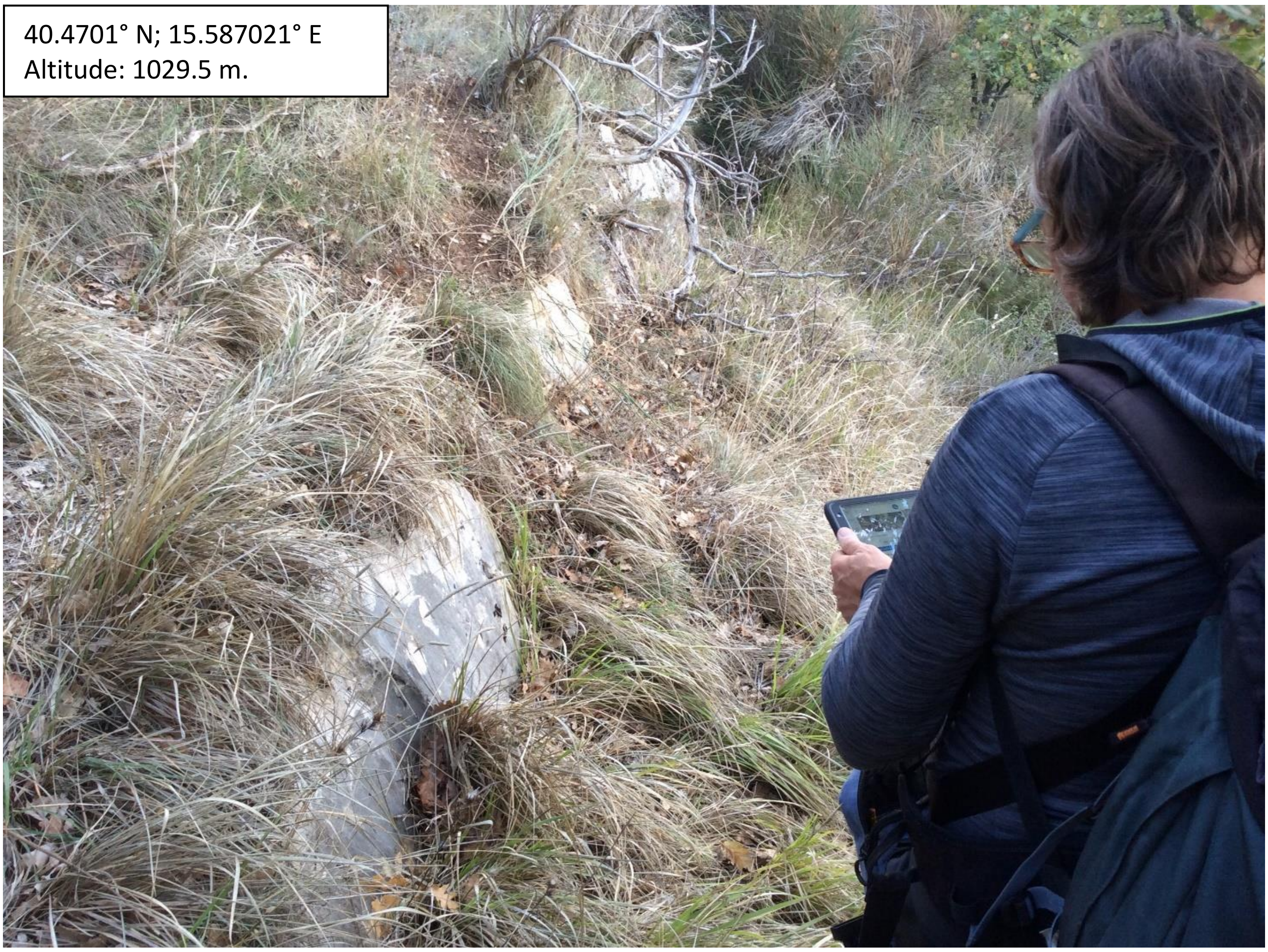

Figure 29.

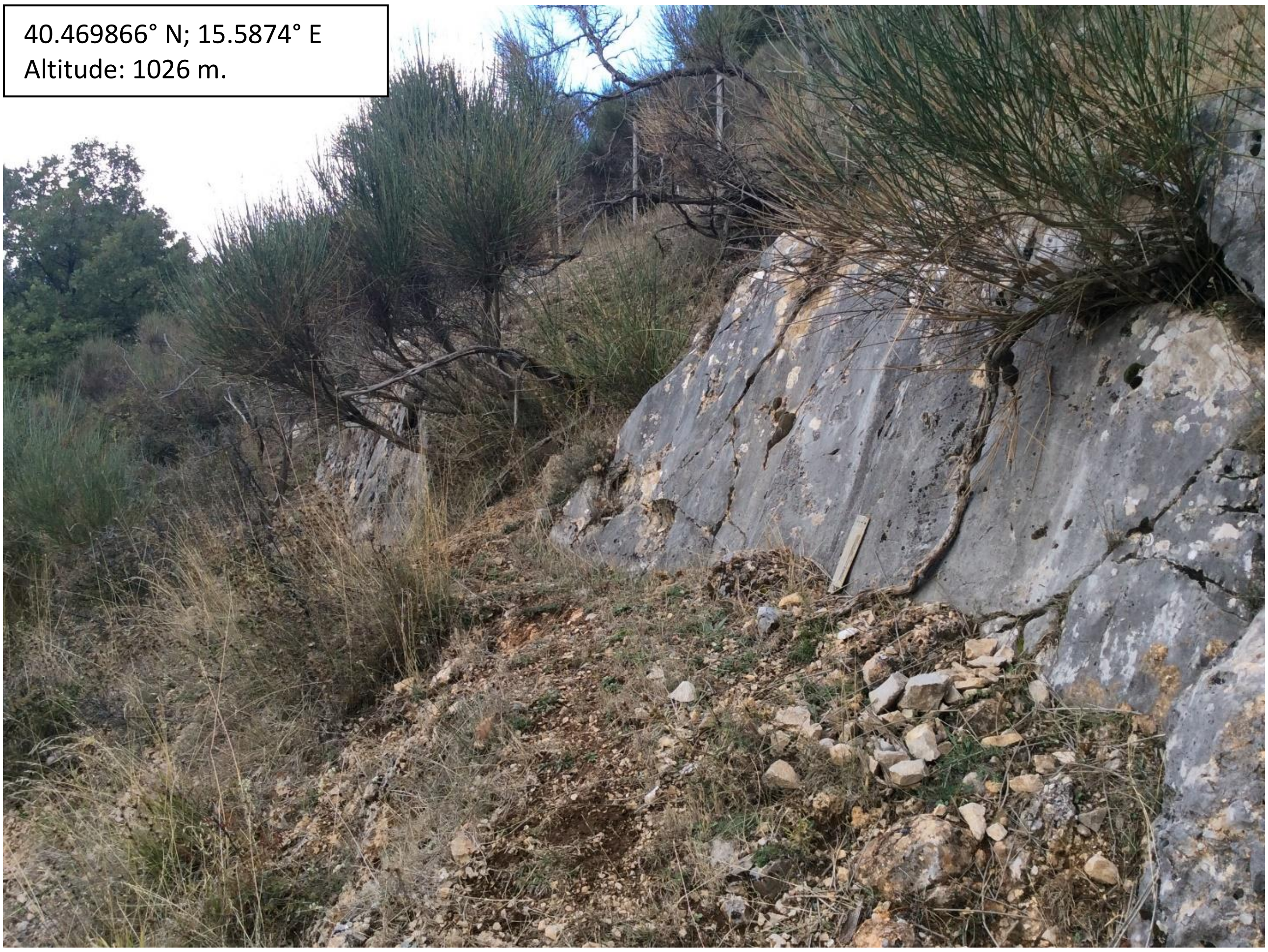

Figure 30.

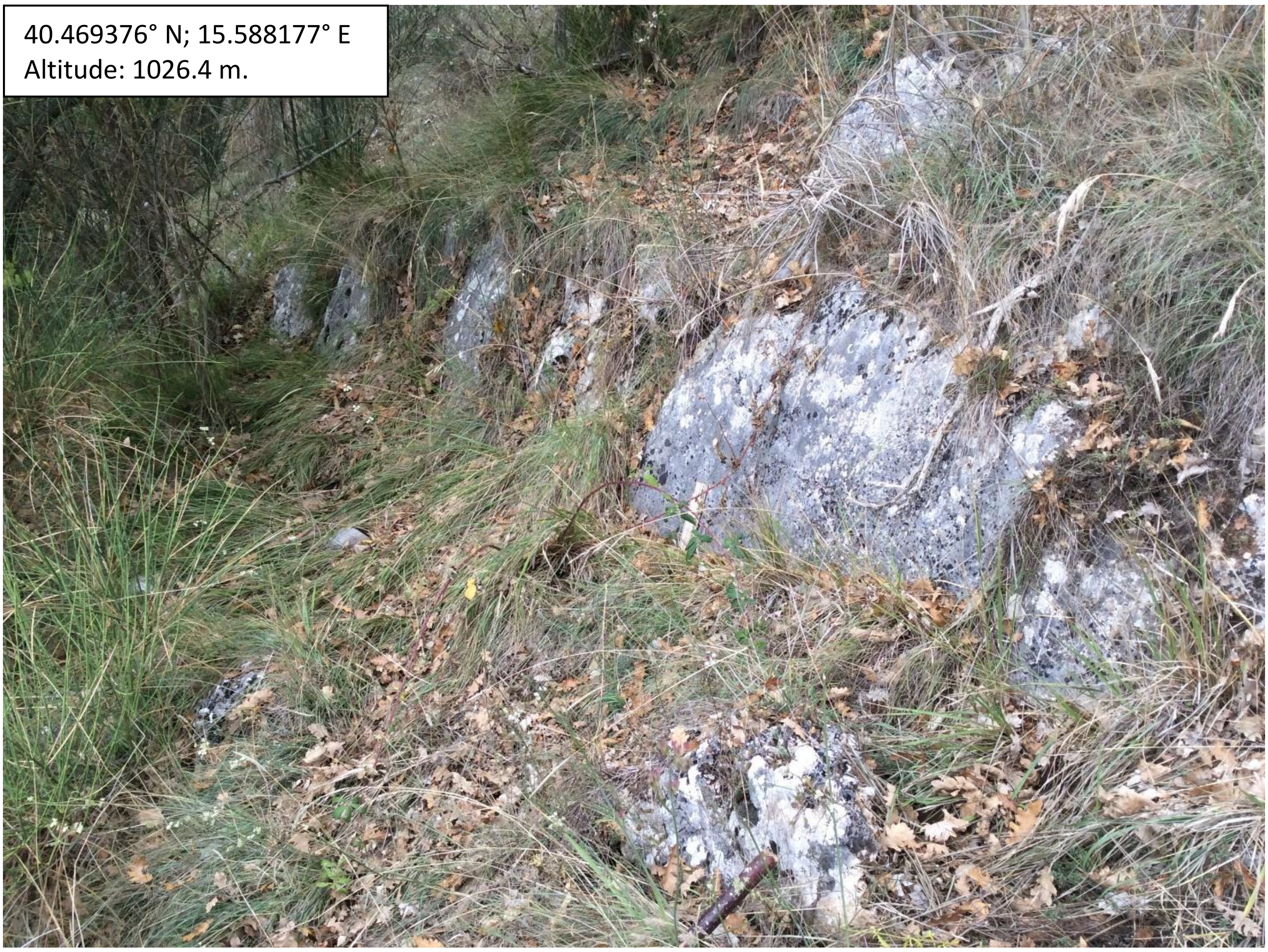

Figure 31.

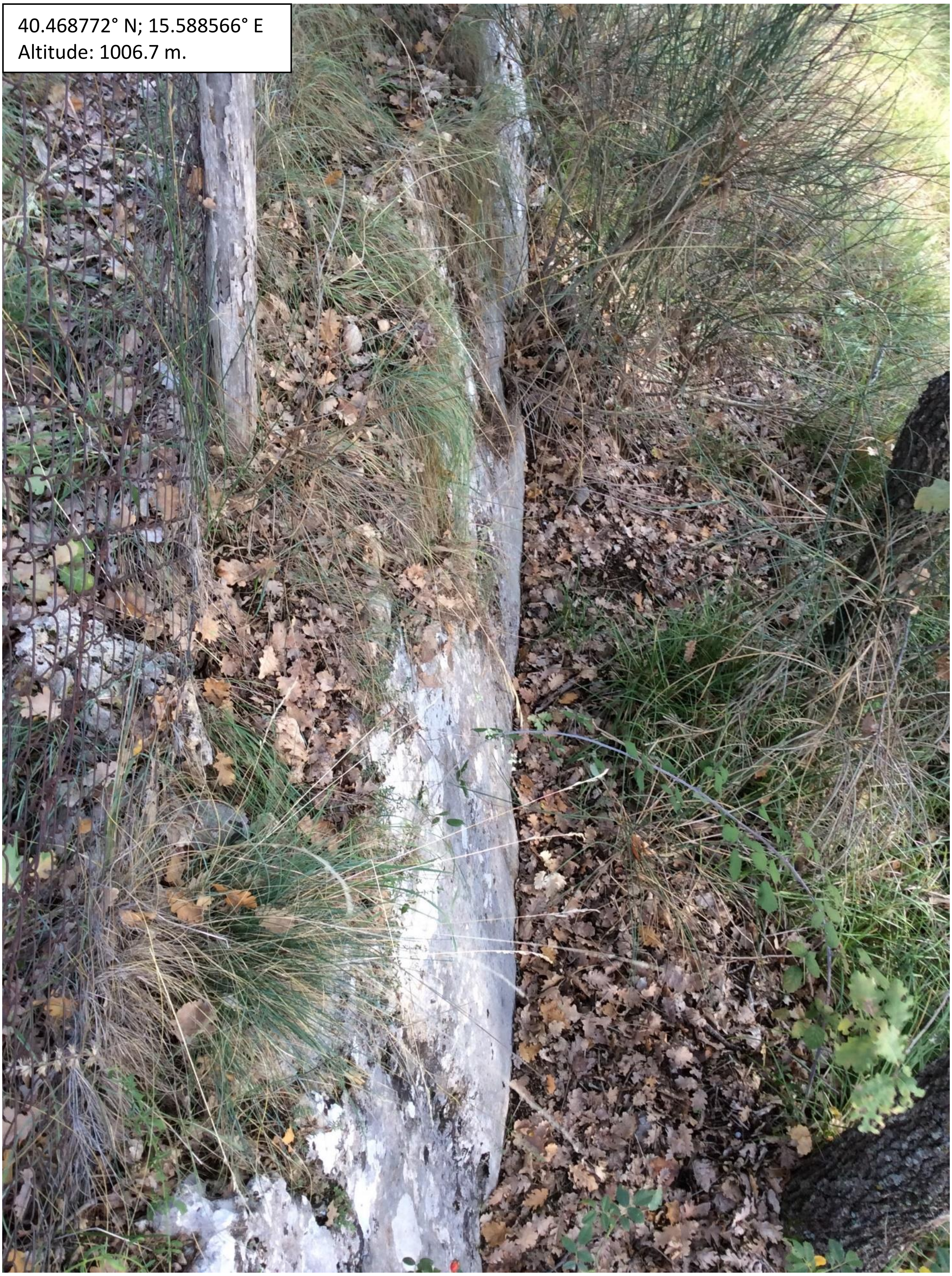

Figure 32.

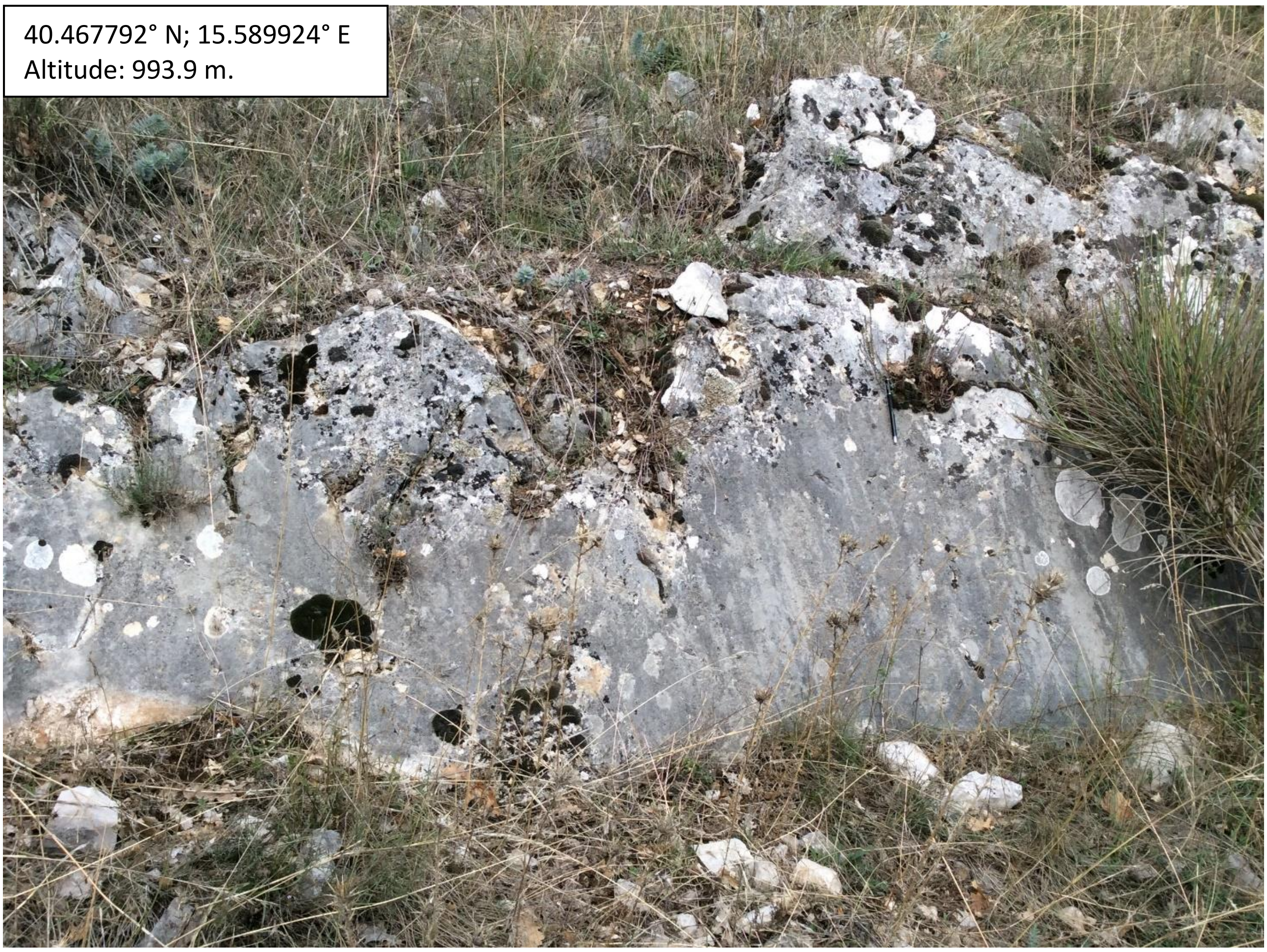

Figure 33.

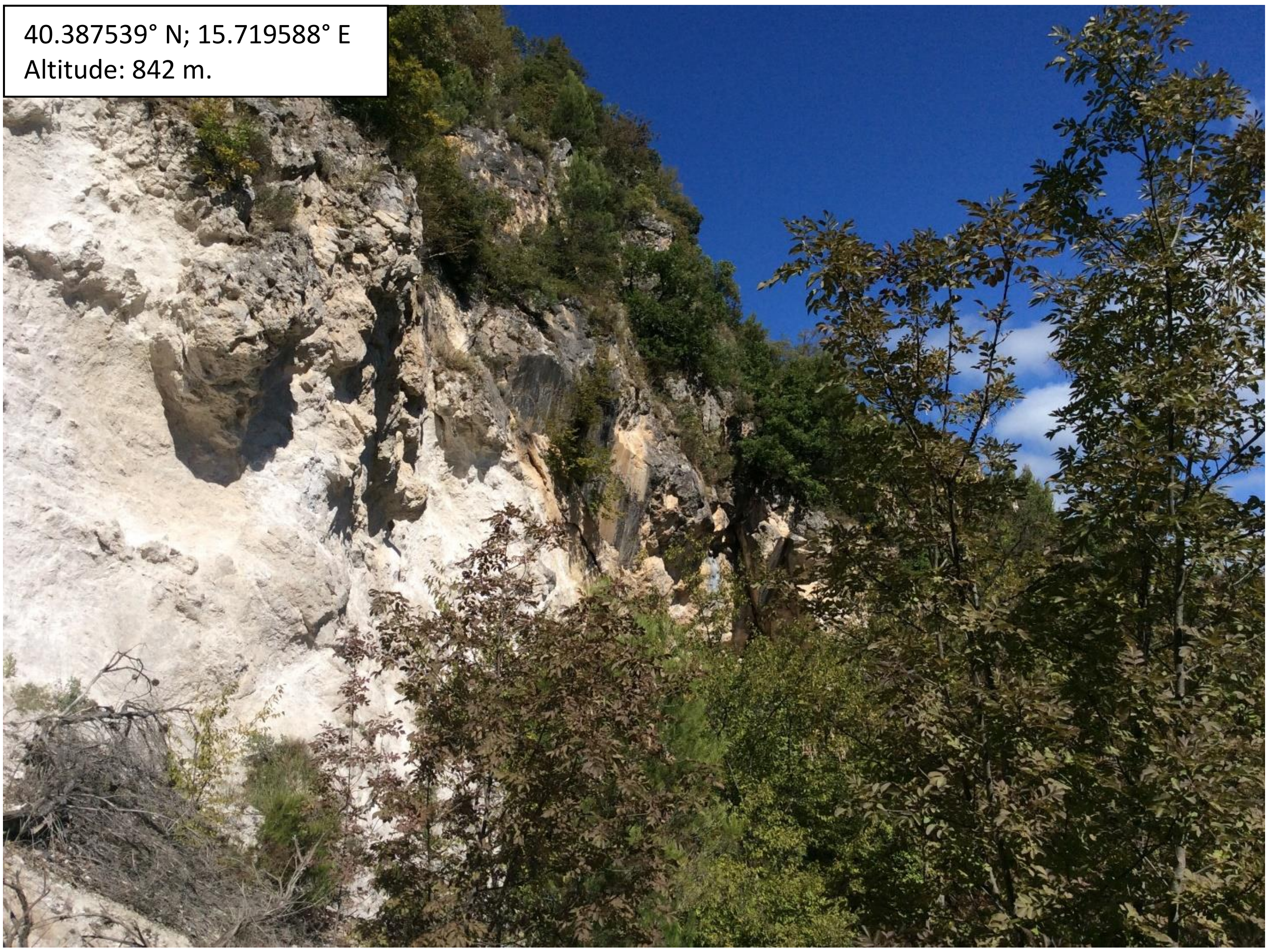

Figure 37.

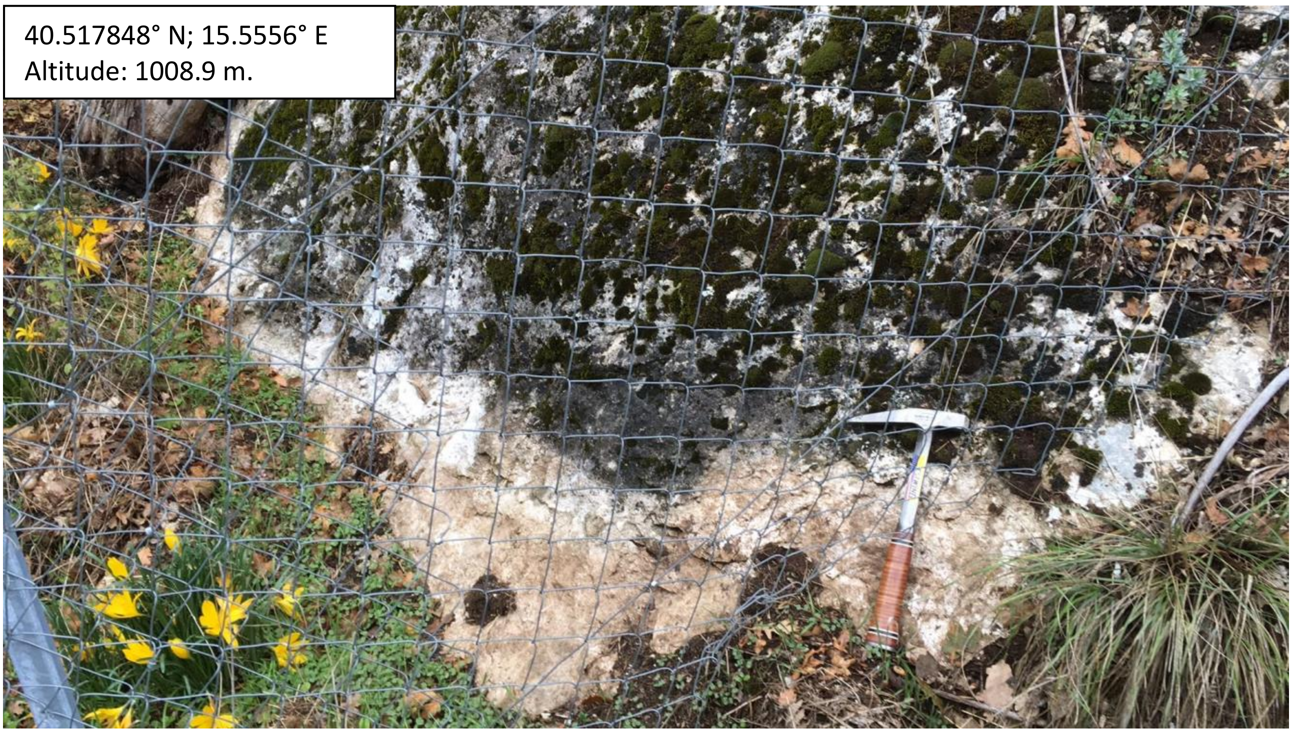

Figure 38.

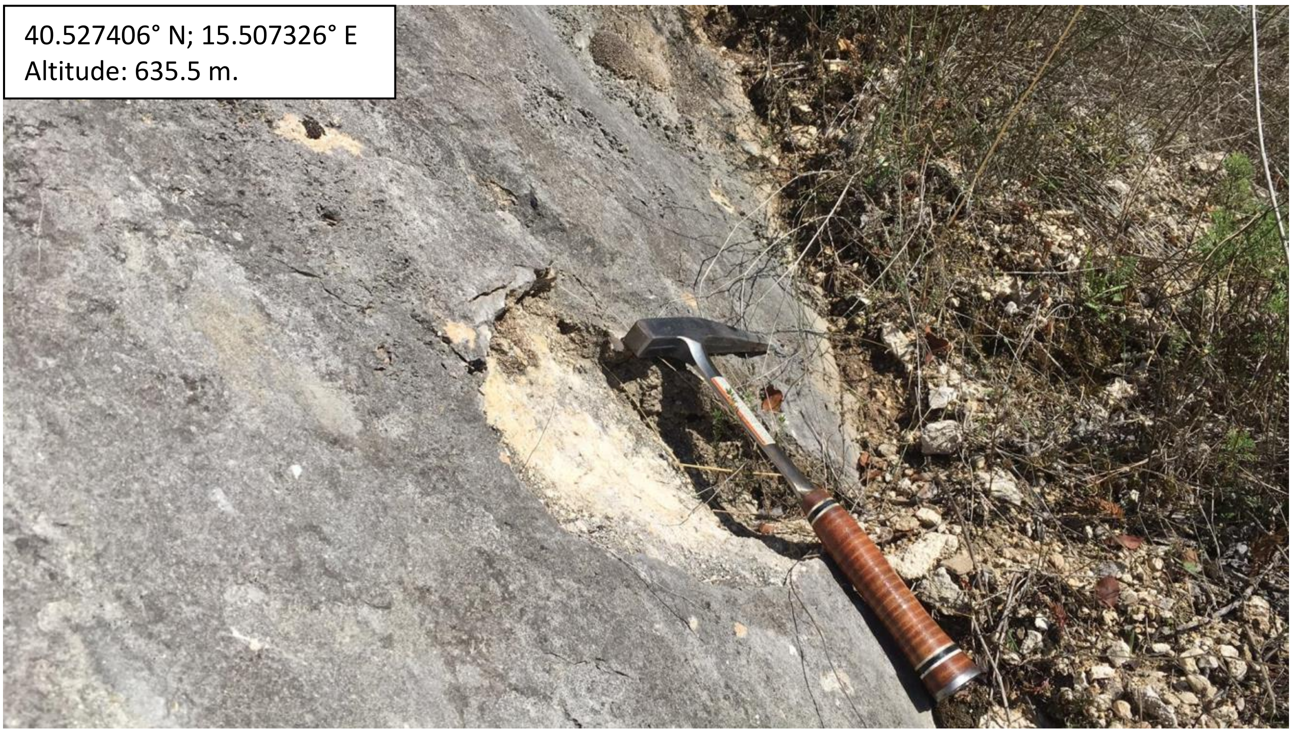

Figure 34.

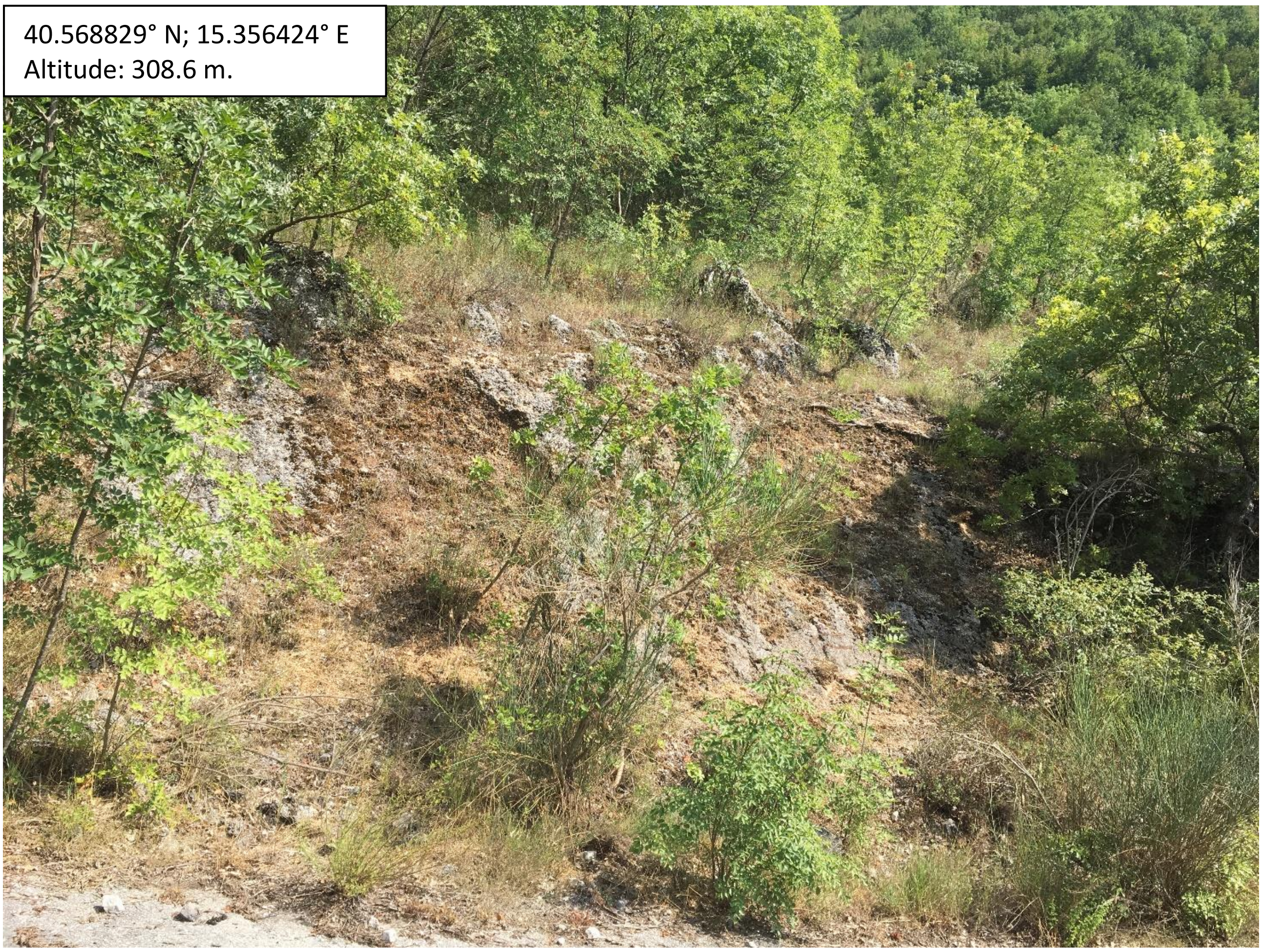

Figure 35.

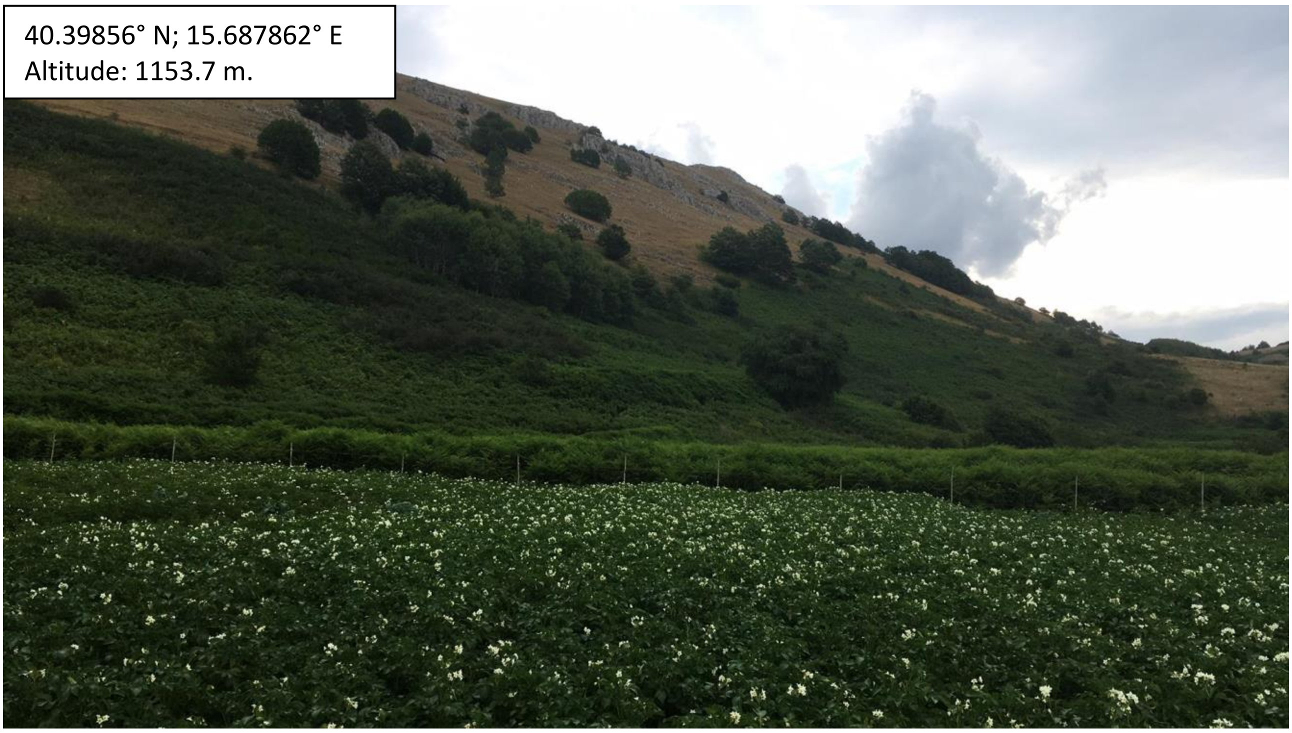

Figure 36.

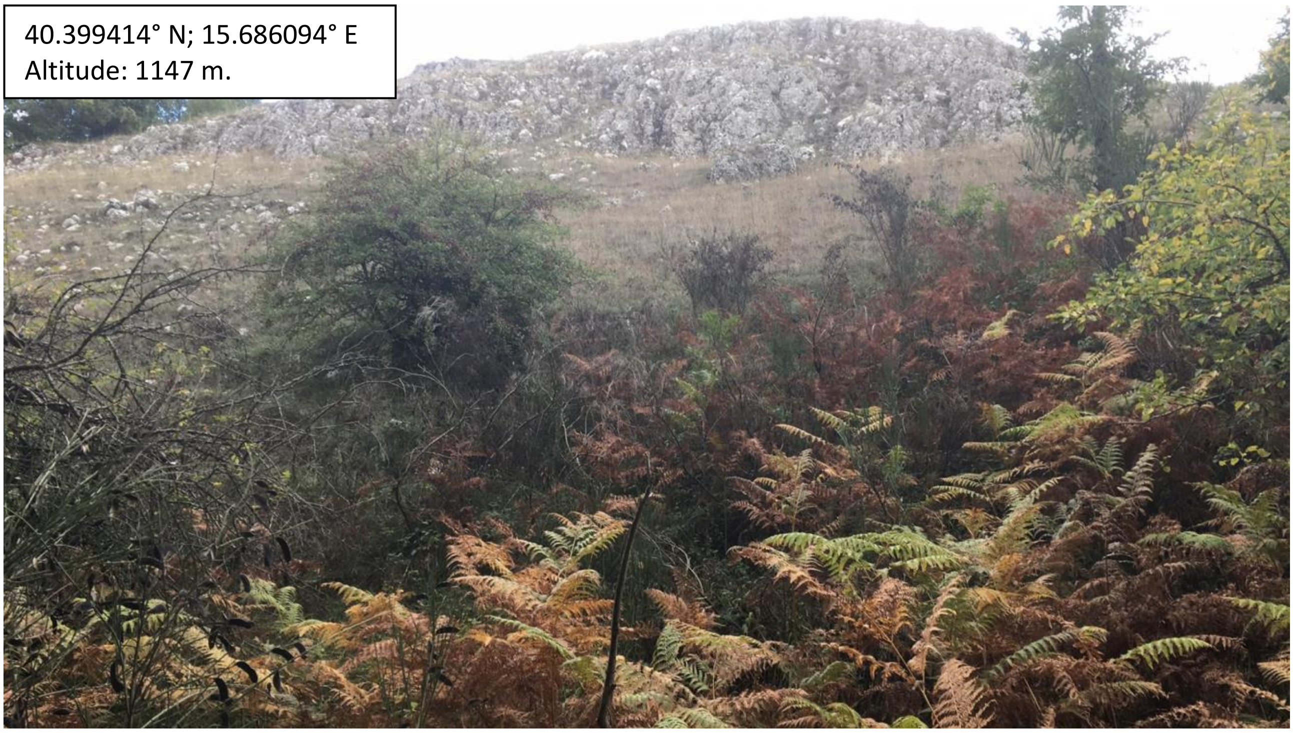

Figure 37.

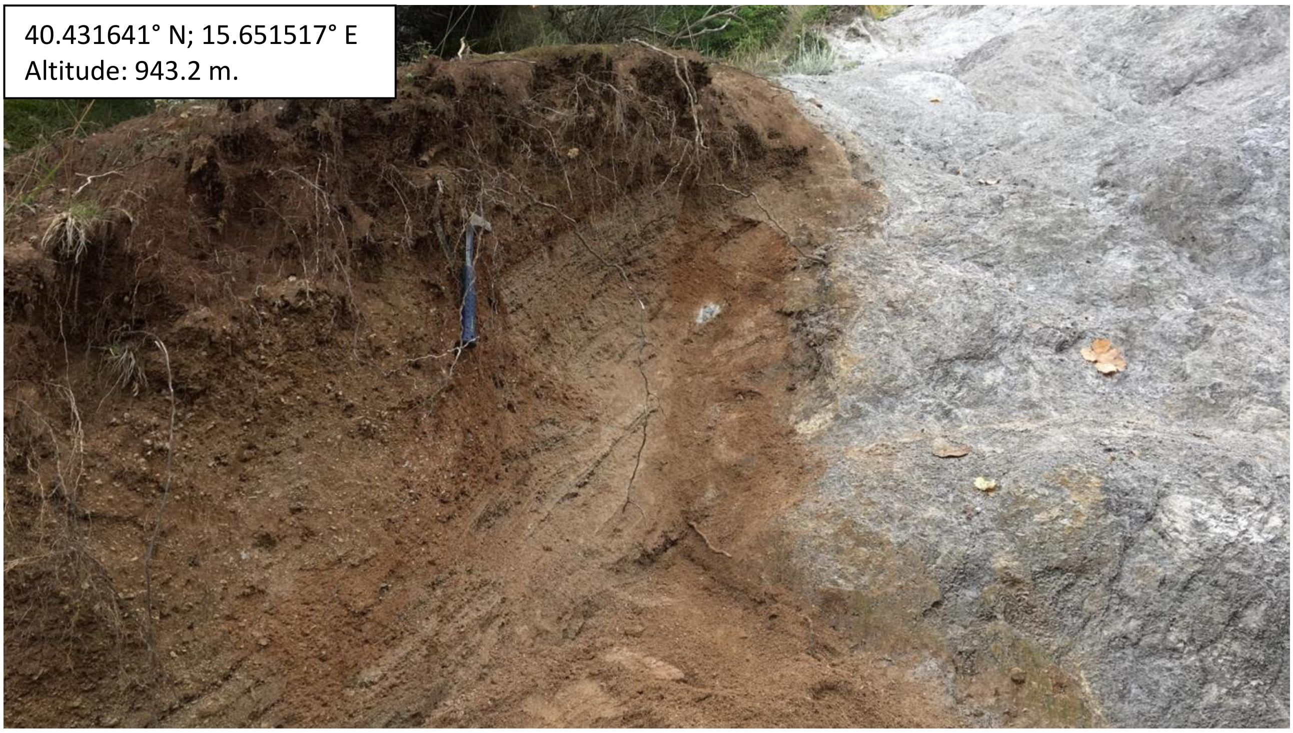

Figure 38.

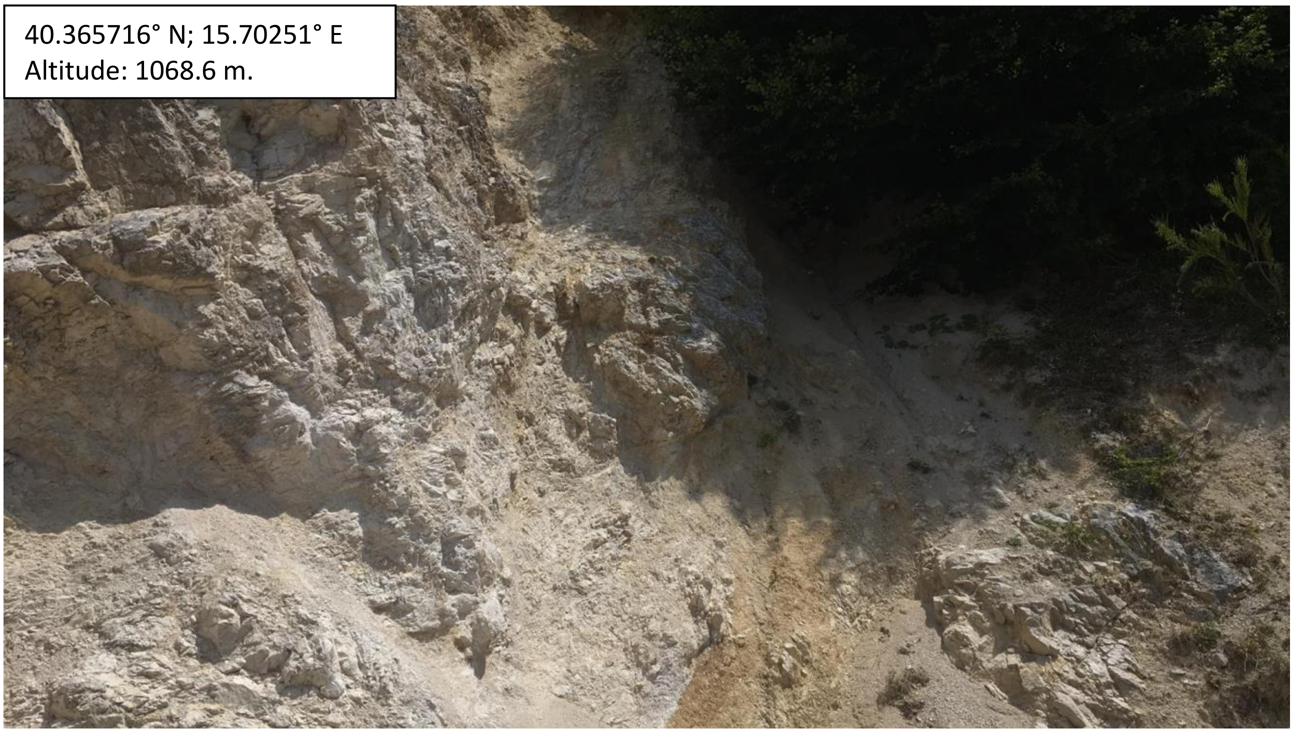

Figure 39.

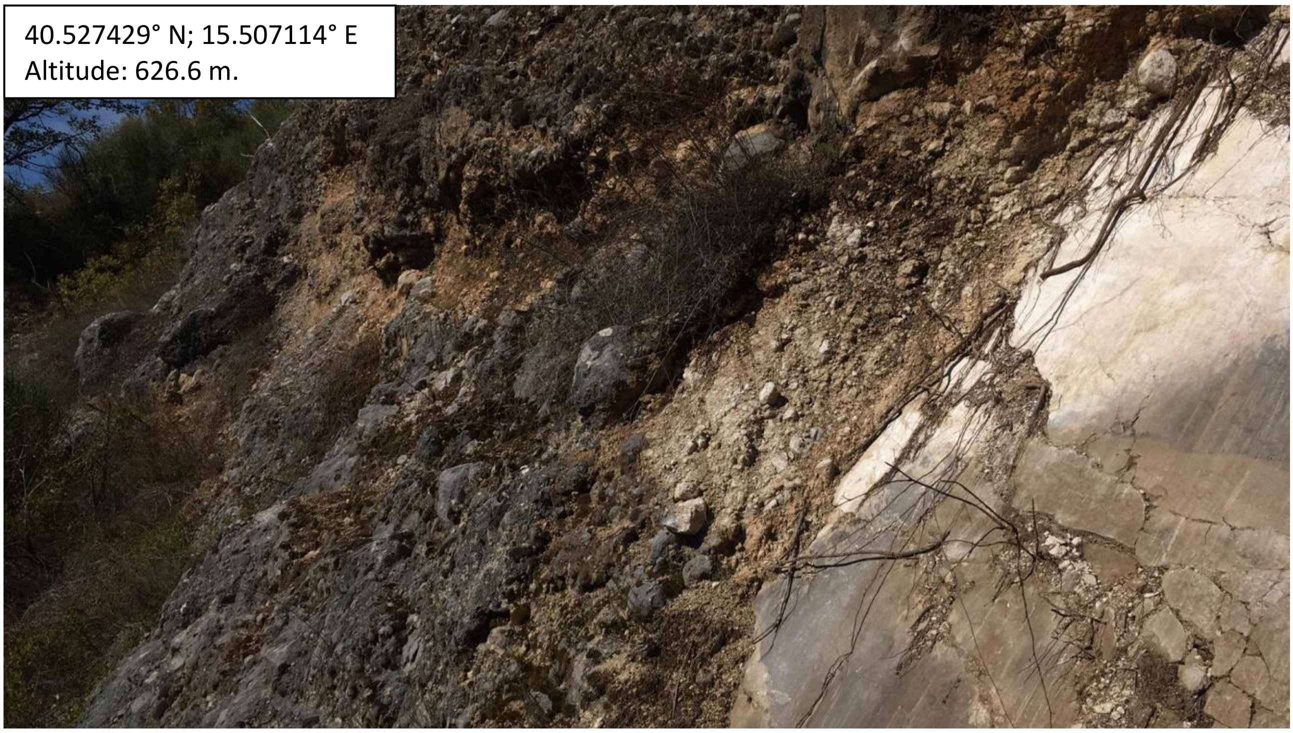

Figure 40.

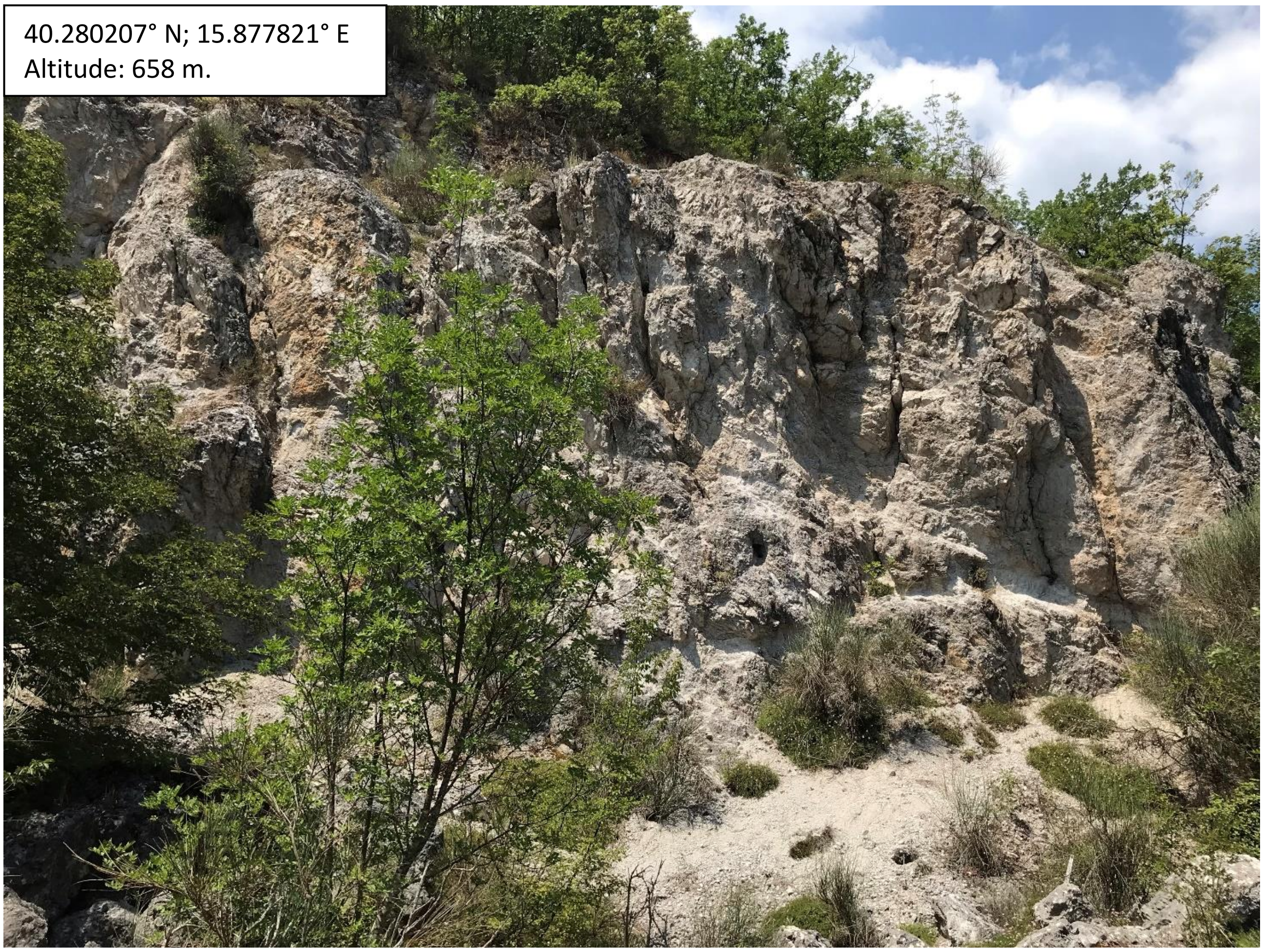

Figure 41.

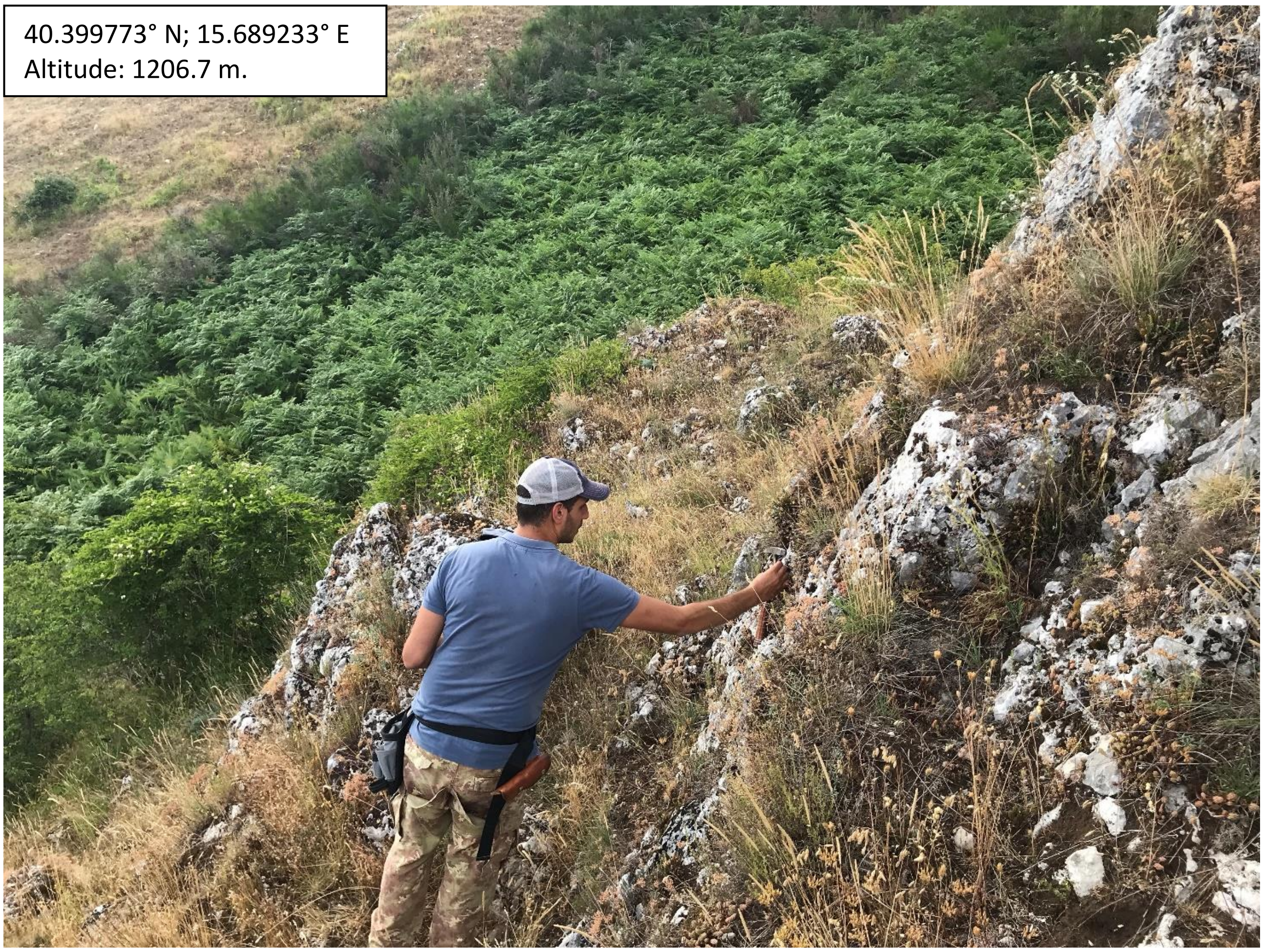

Figure 42.

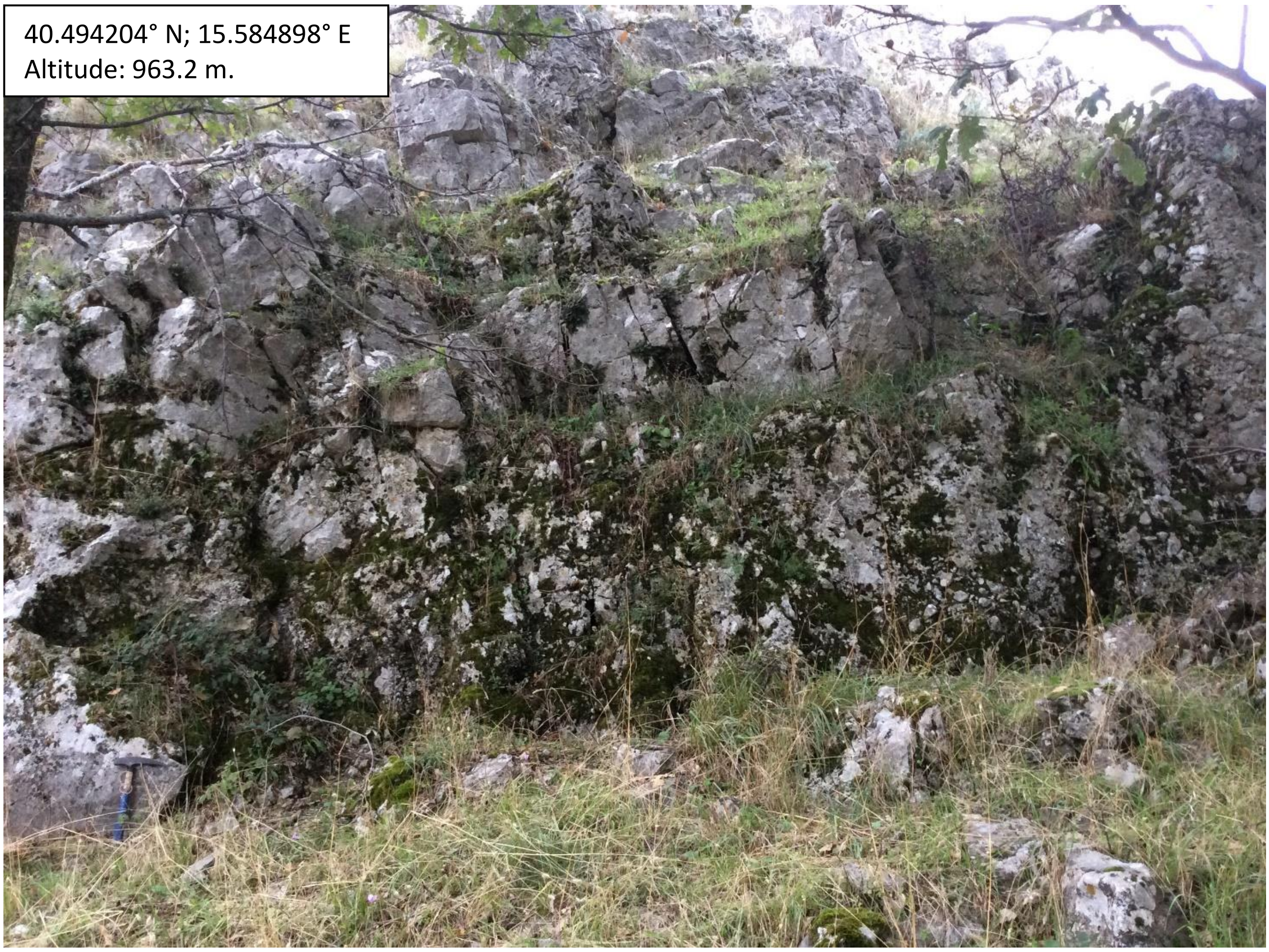

Figure 43.

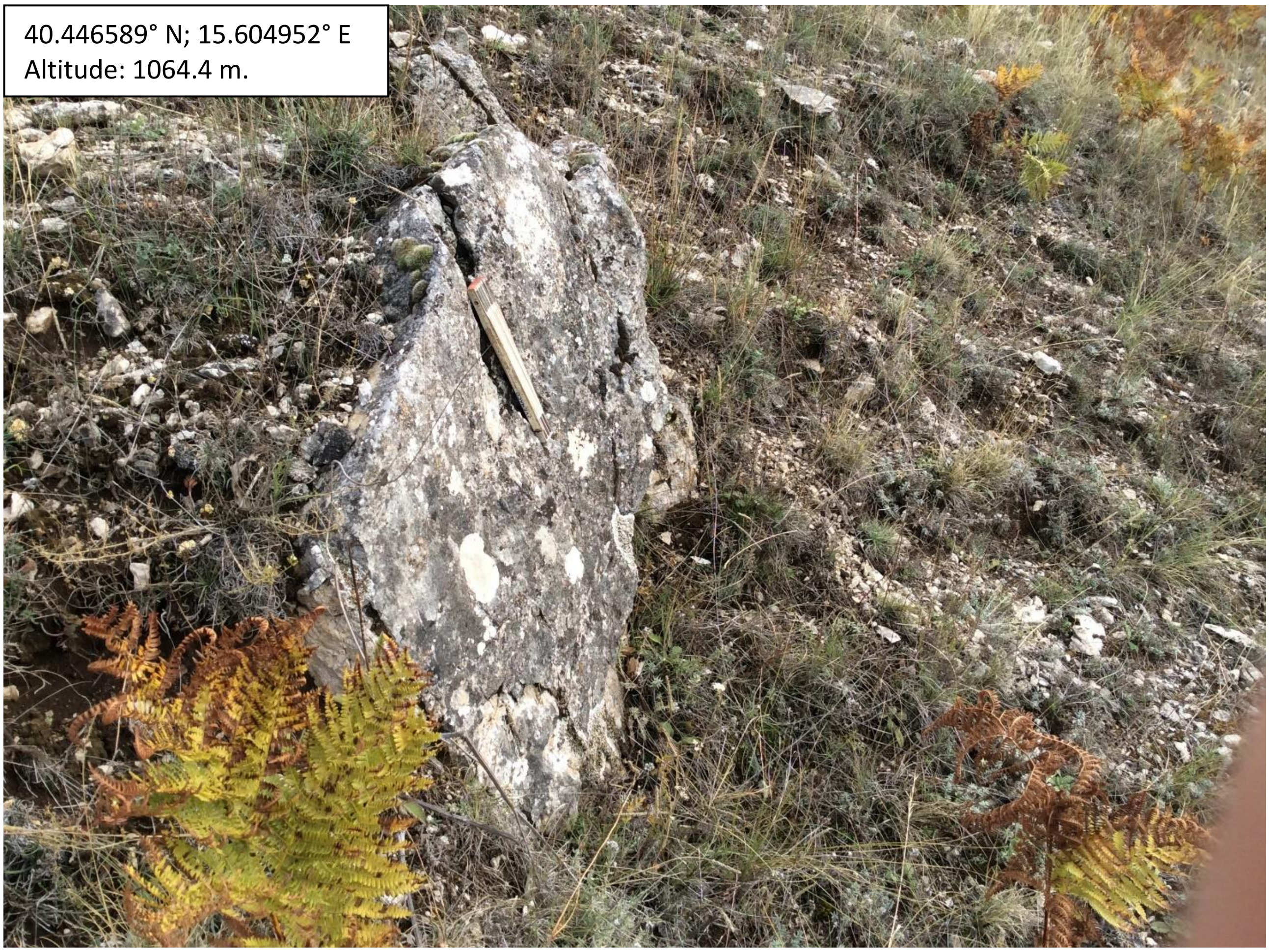

Figure 44.

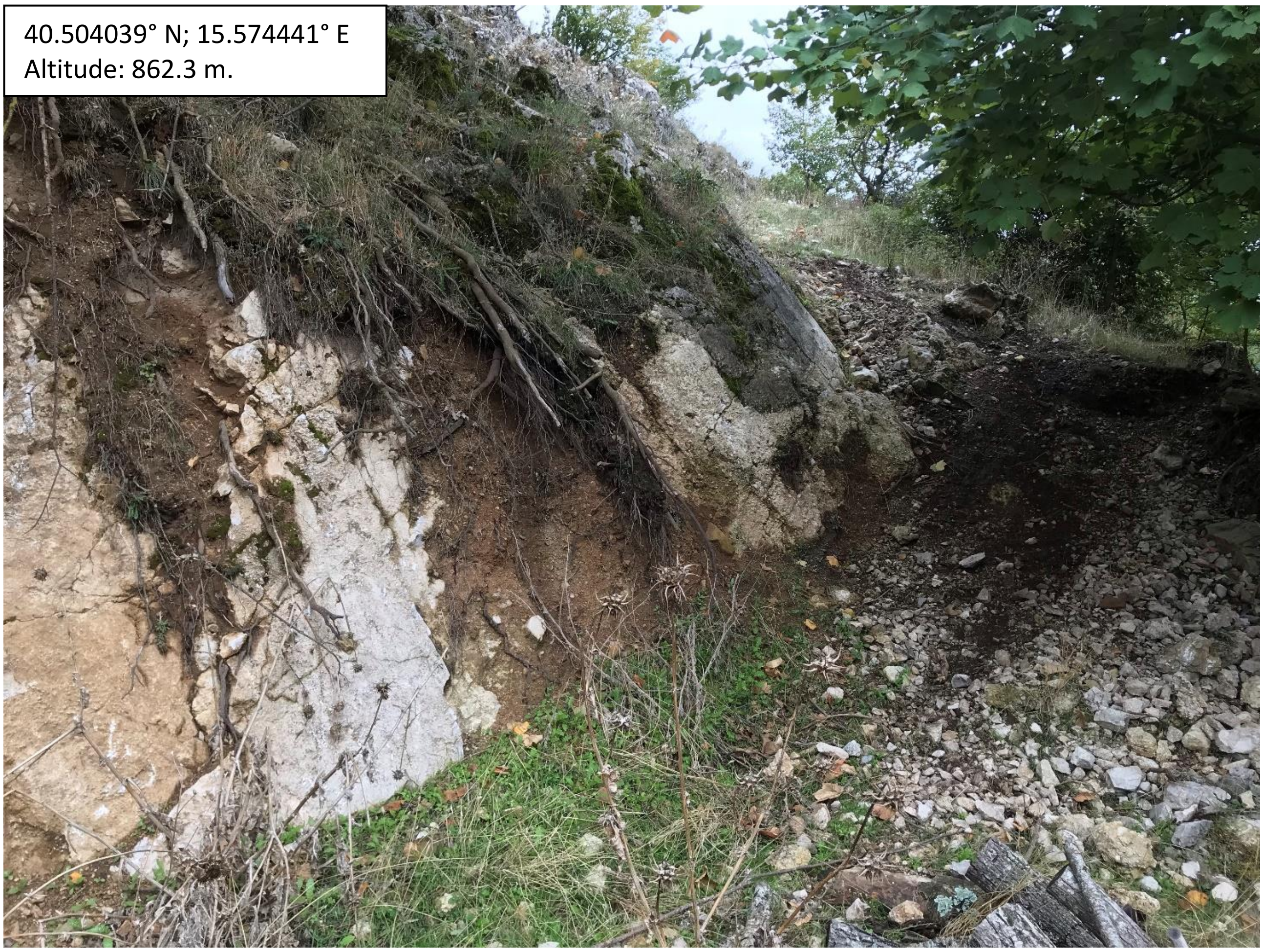

Figure 45.

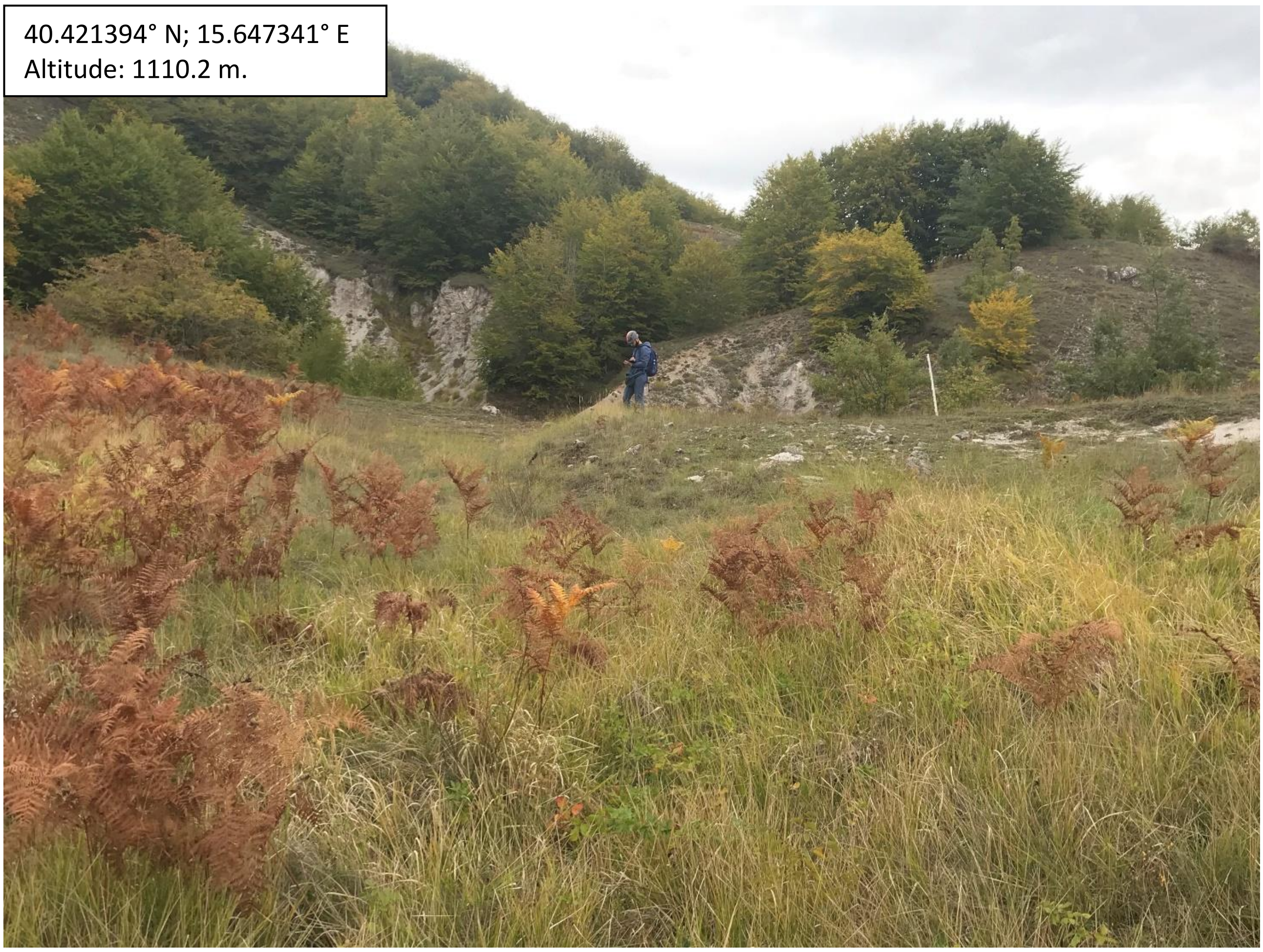

Figure 46.

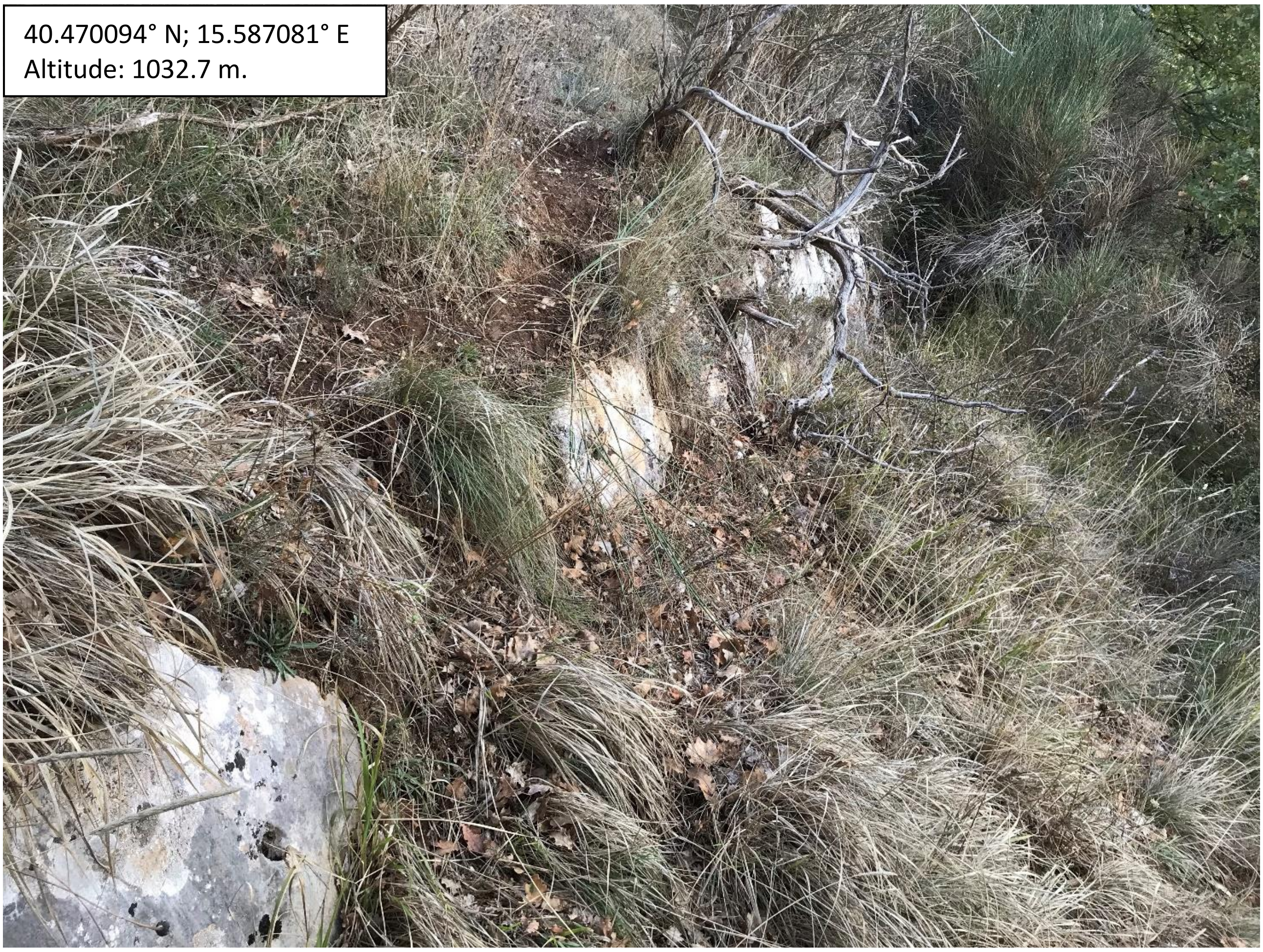

Figure 47.

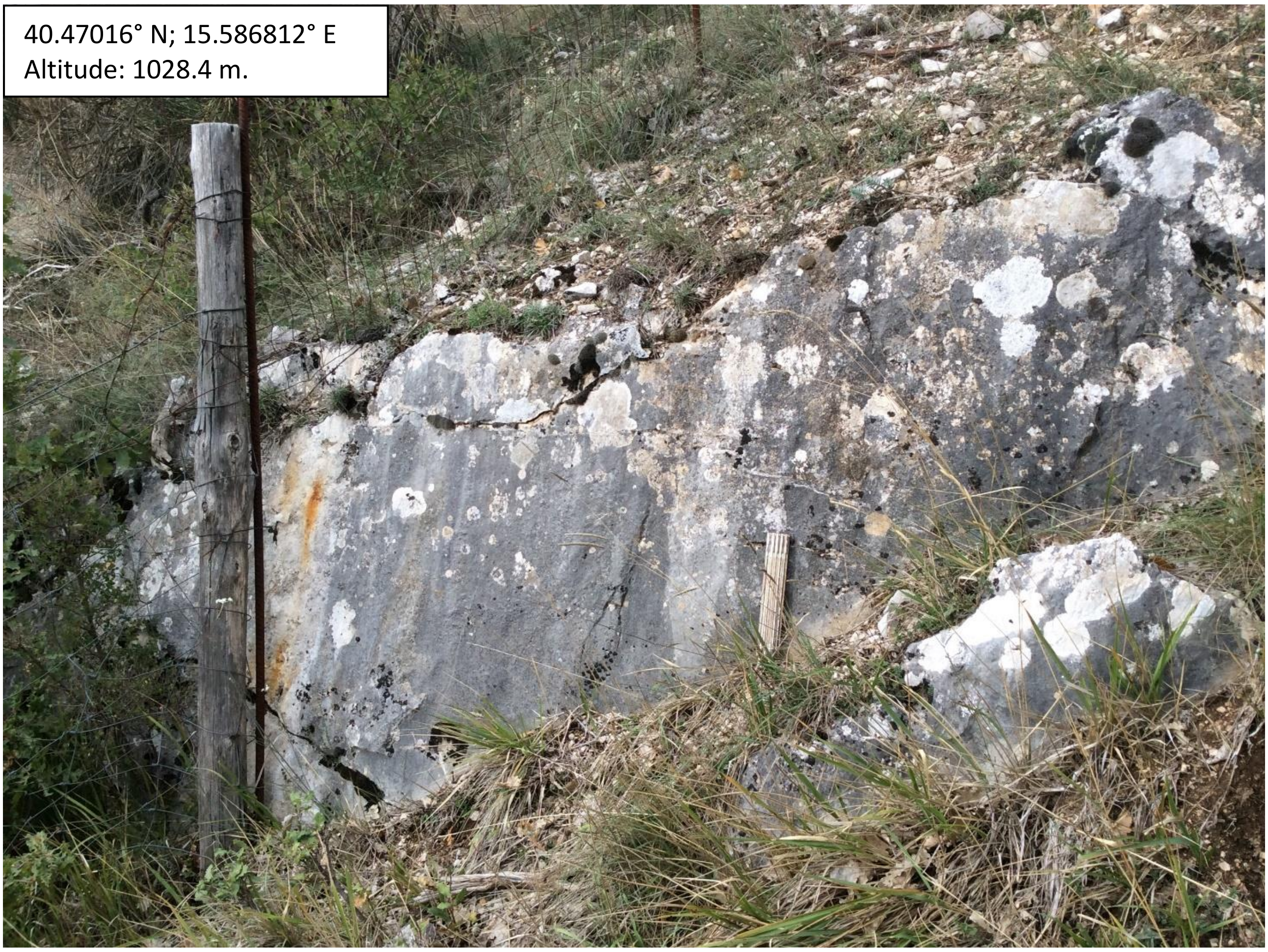

Figure 48.

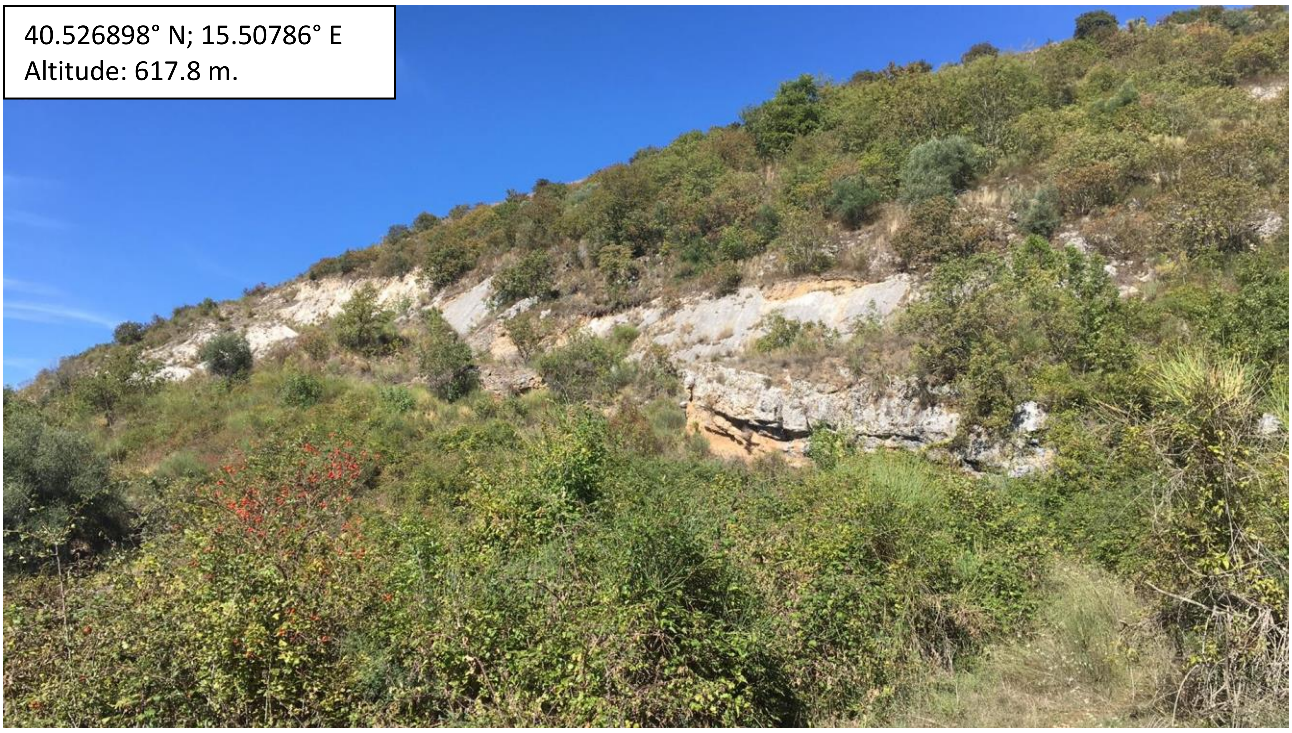

Figure 49.

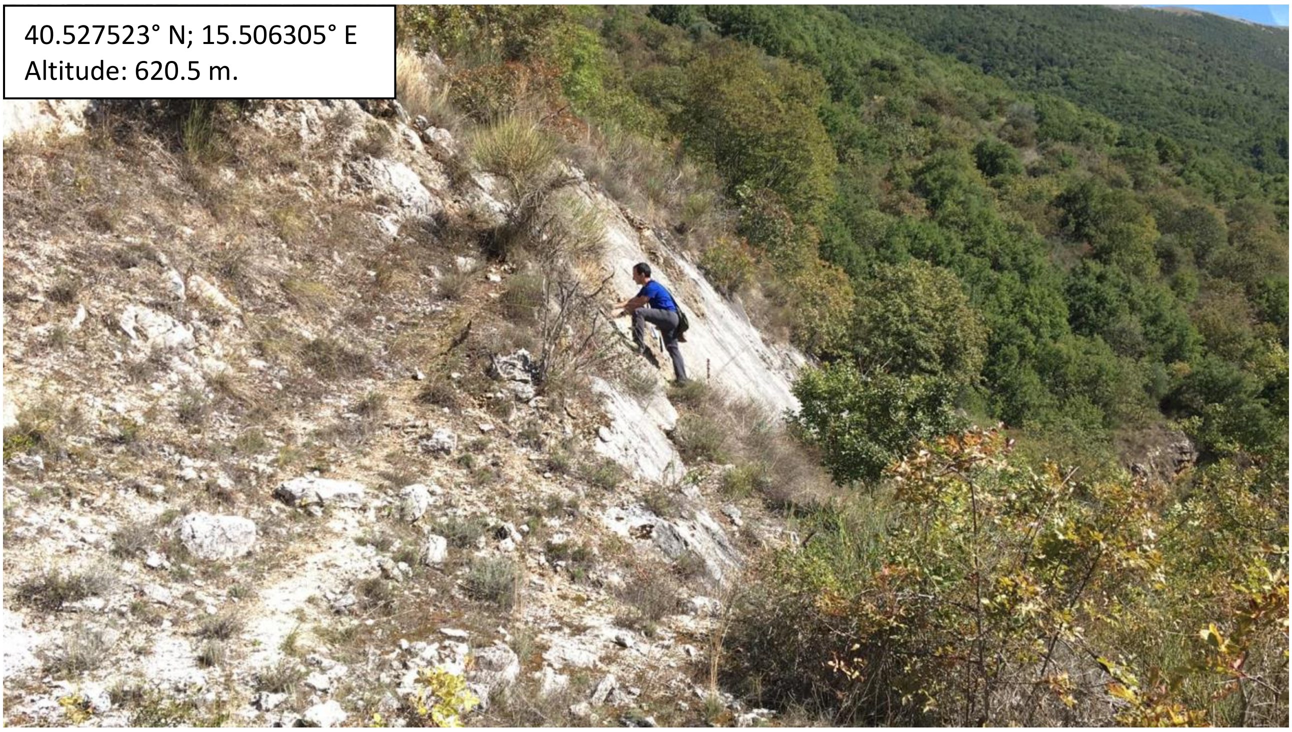

Figure 50.

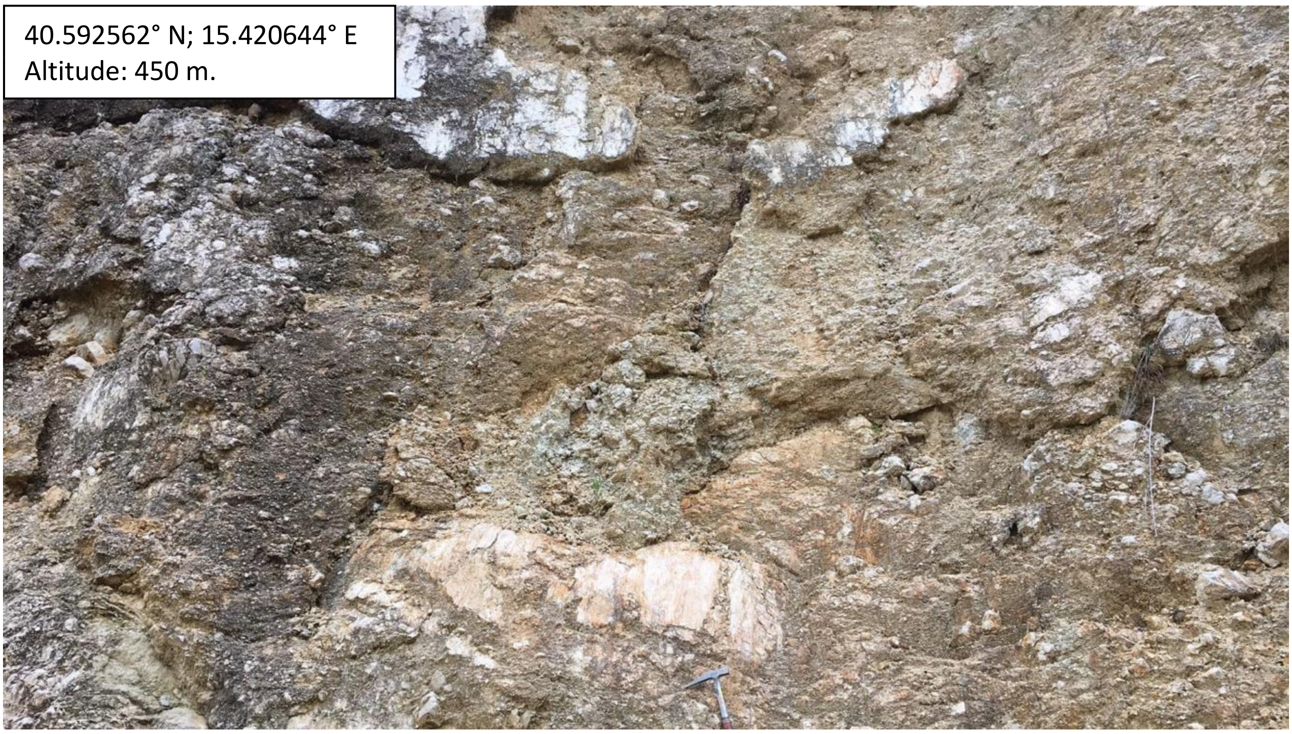

Figure 51.

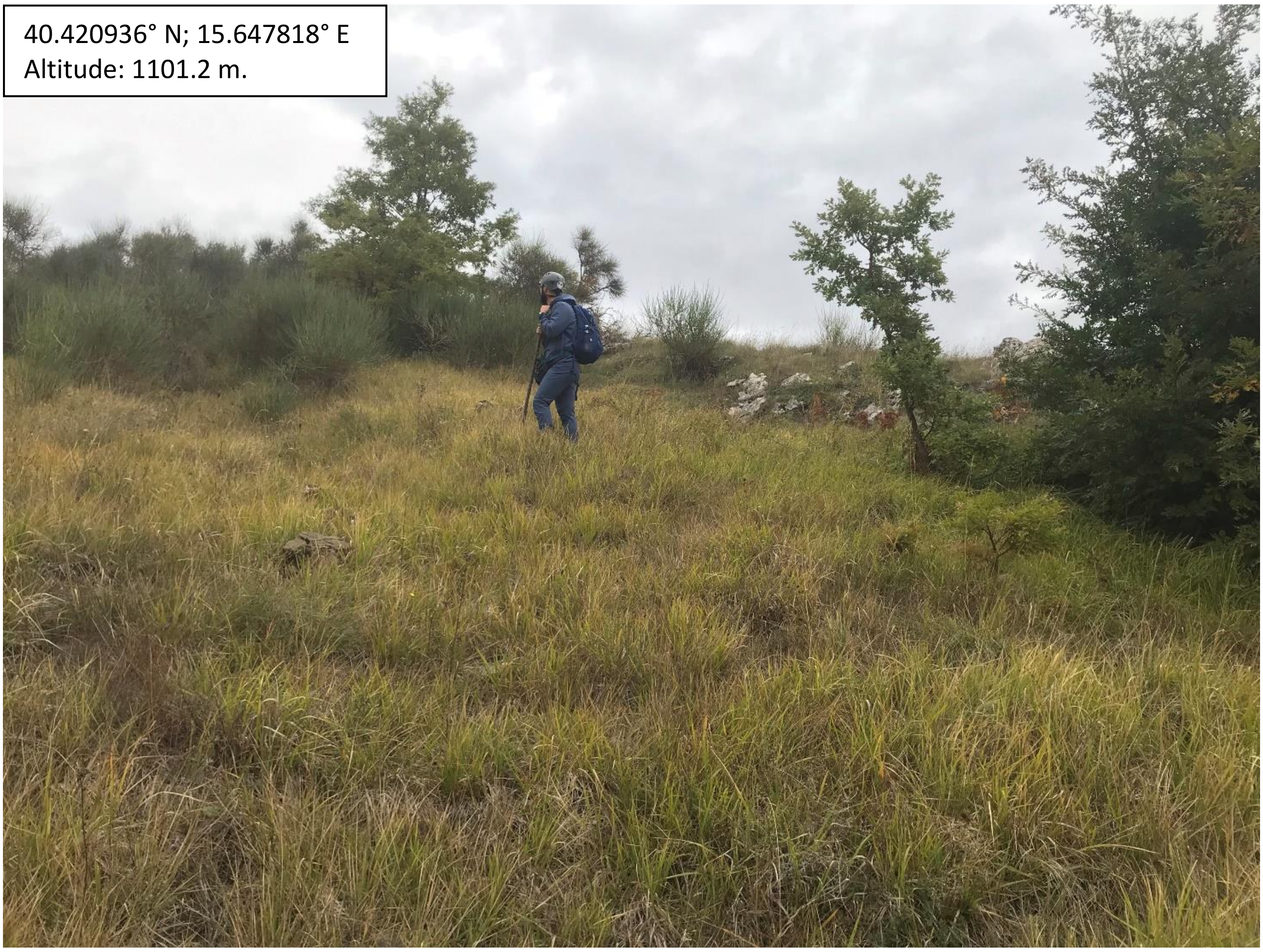

Figure 52.

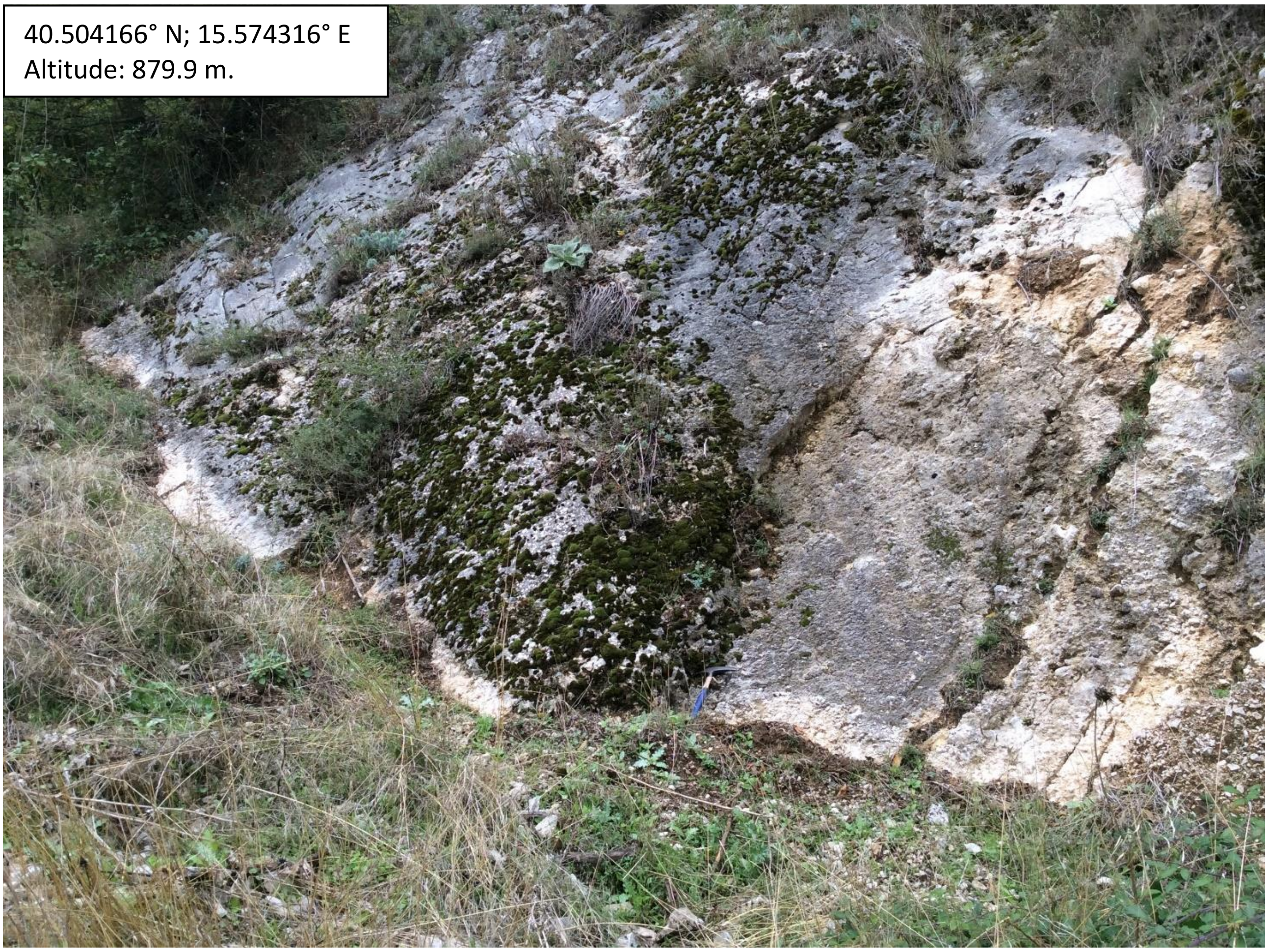

Figure 53.

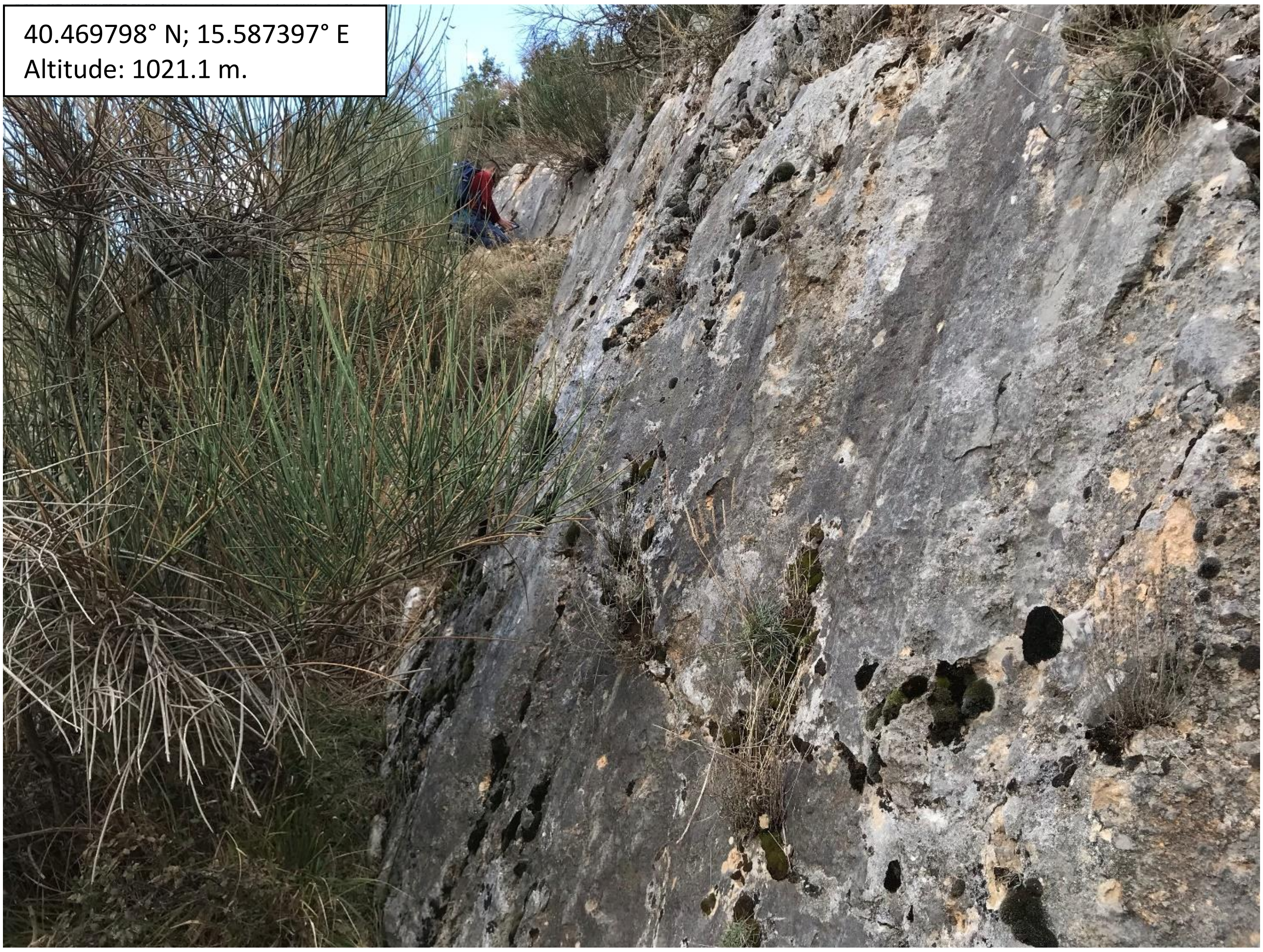

Figure 54.

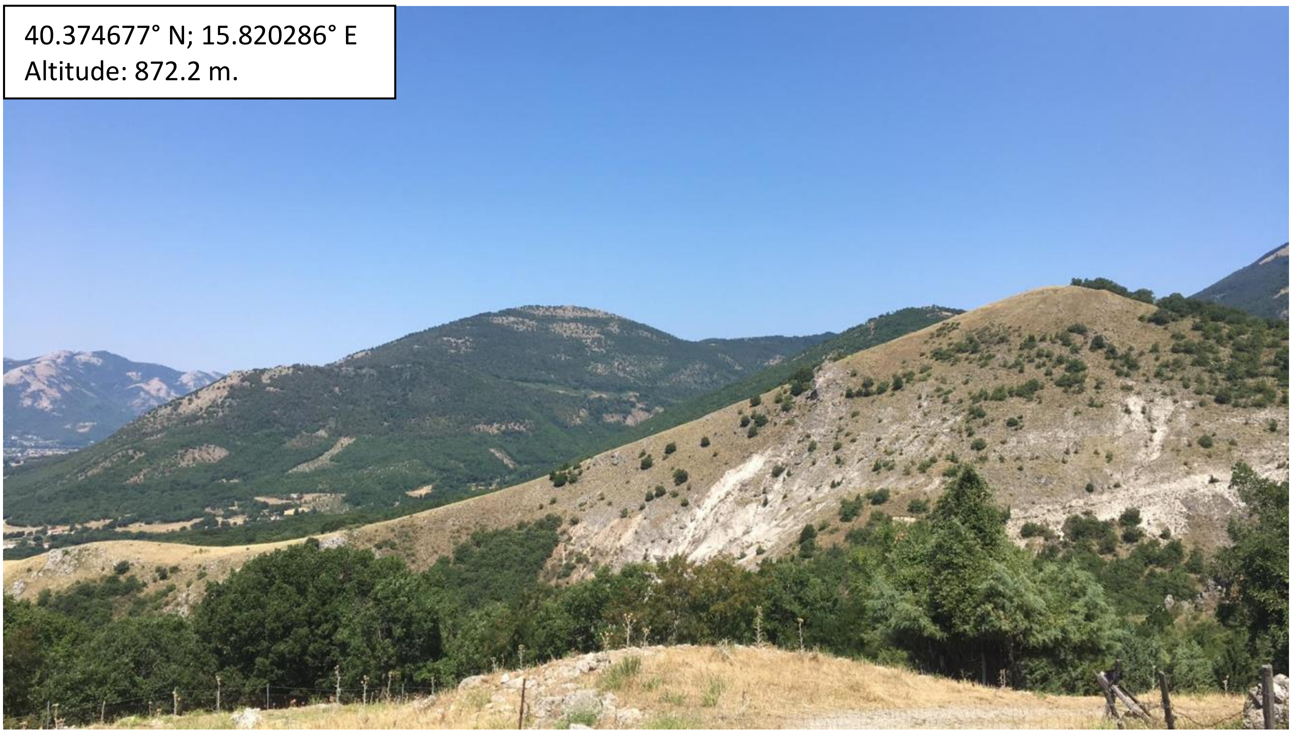

Figure 55.

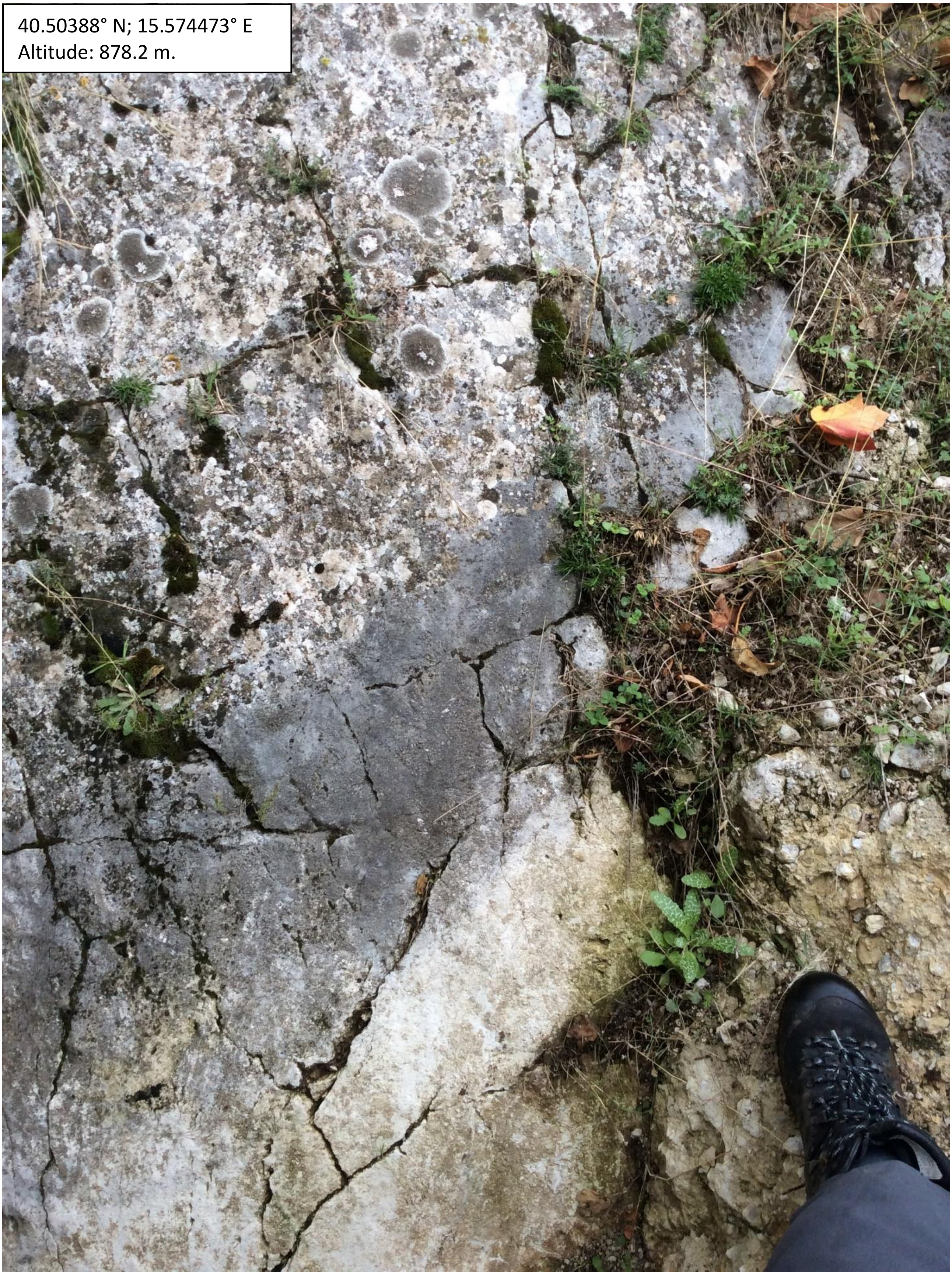

Figure 56.

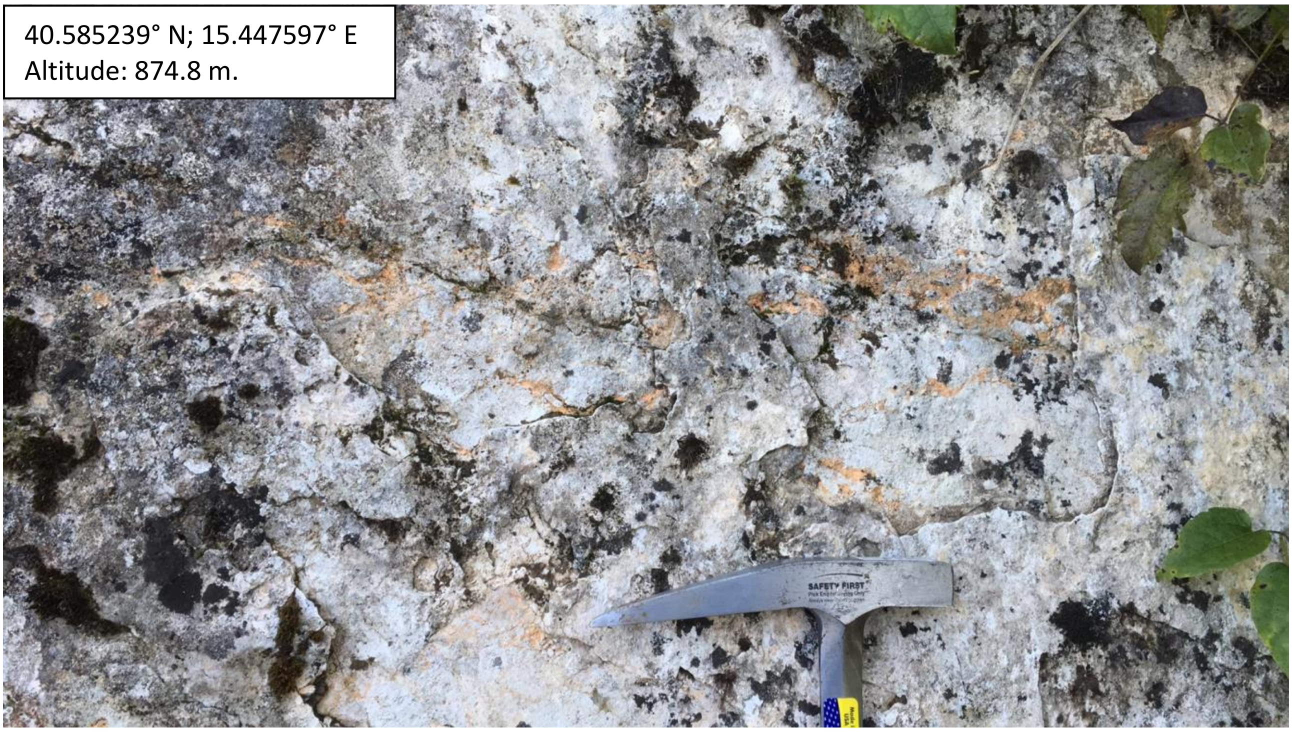

Figure 57.

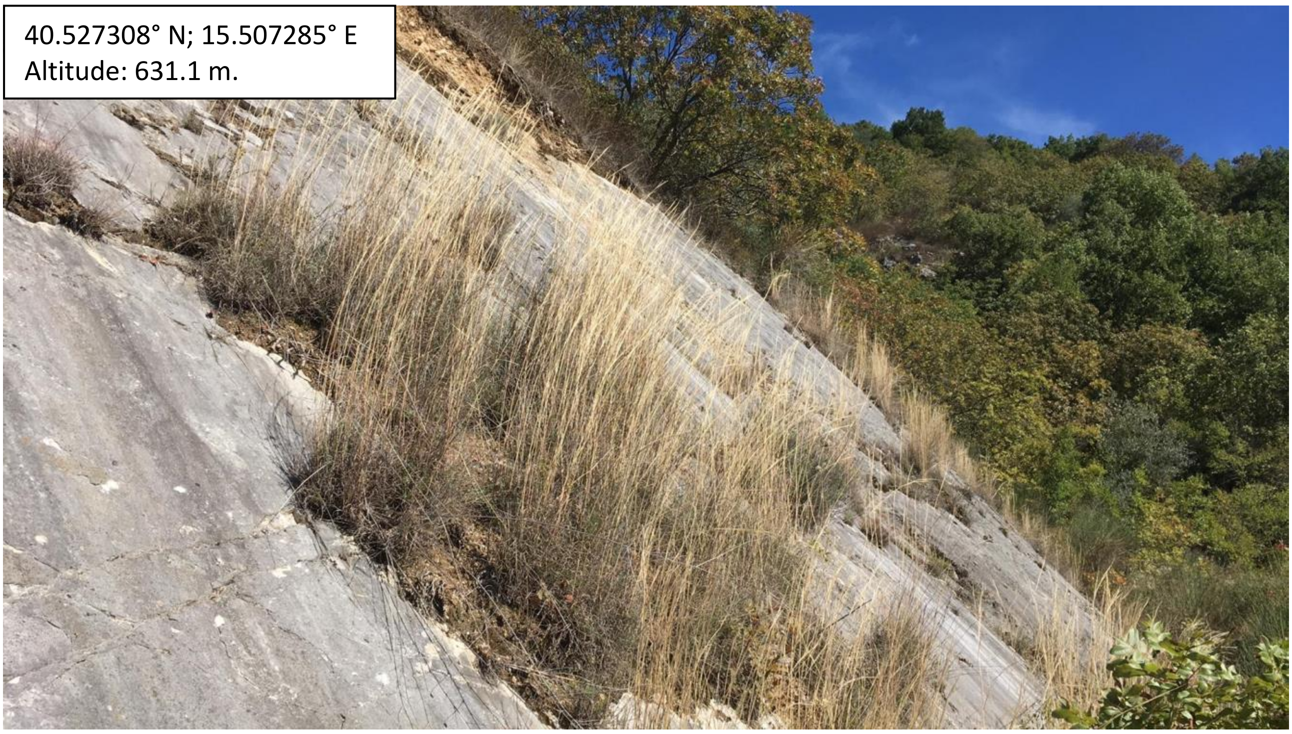

Figure 68.

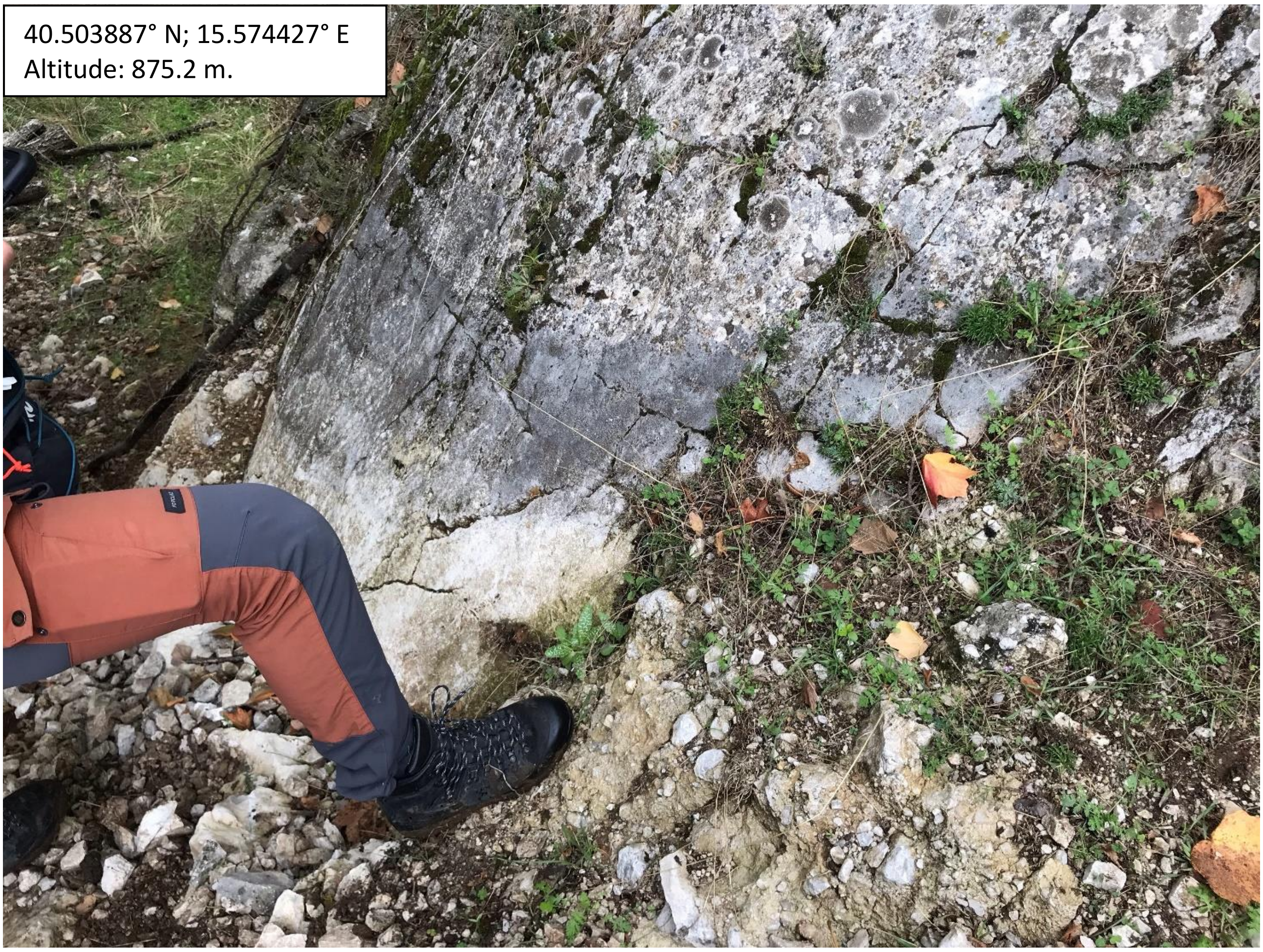

Figure 58.

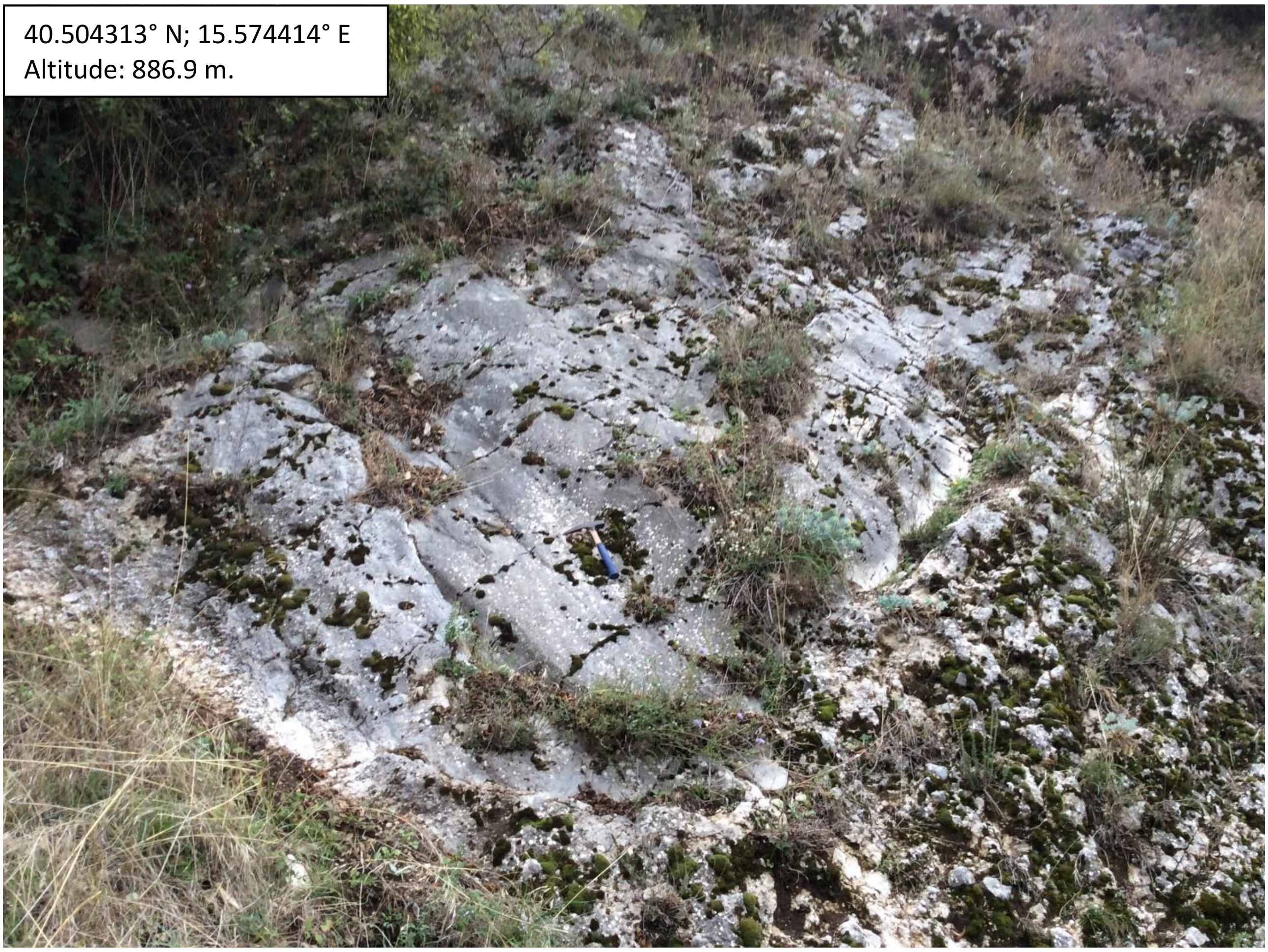

Figure 59.

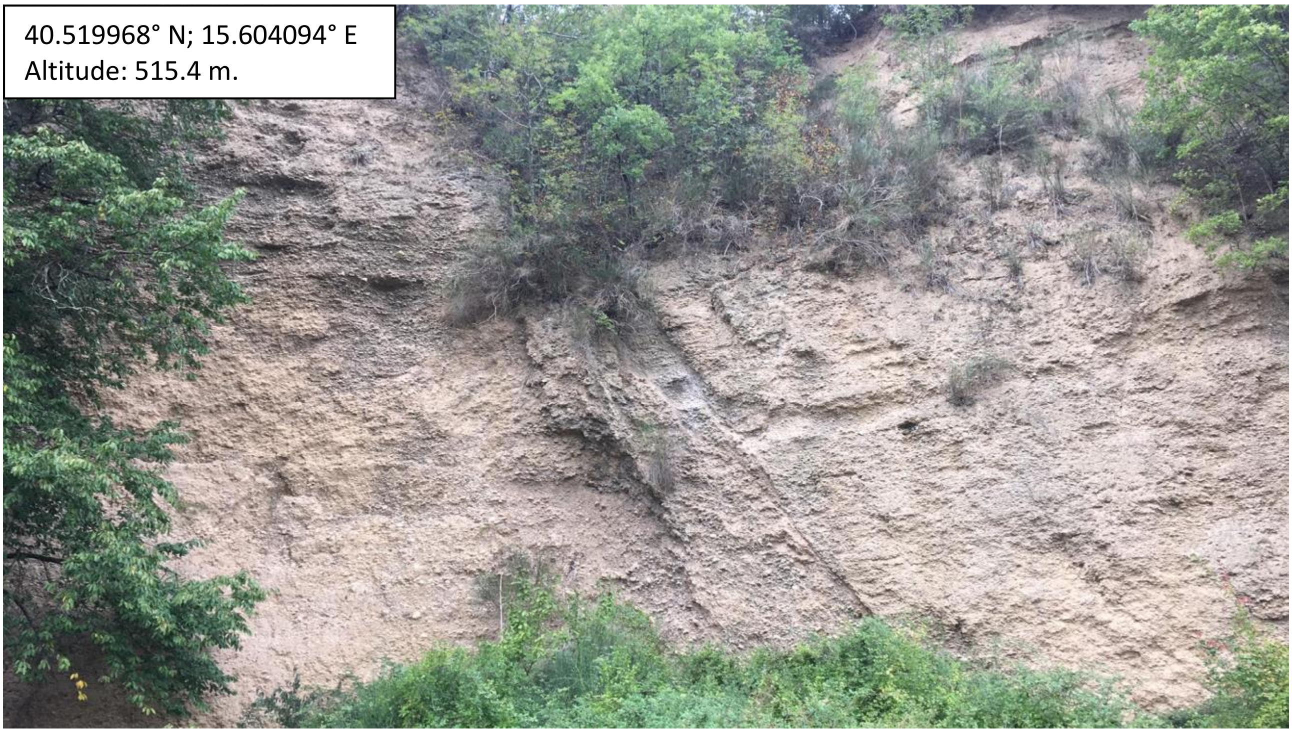

Figure 60.

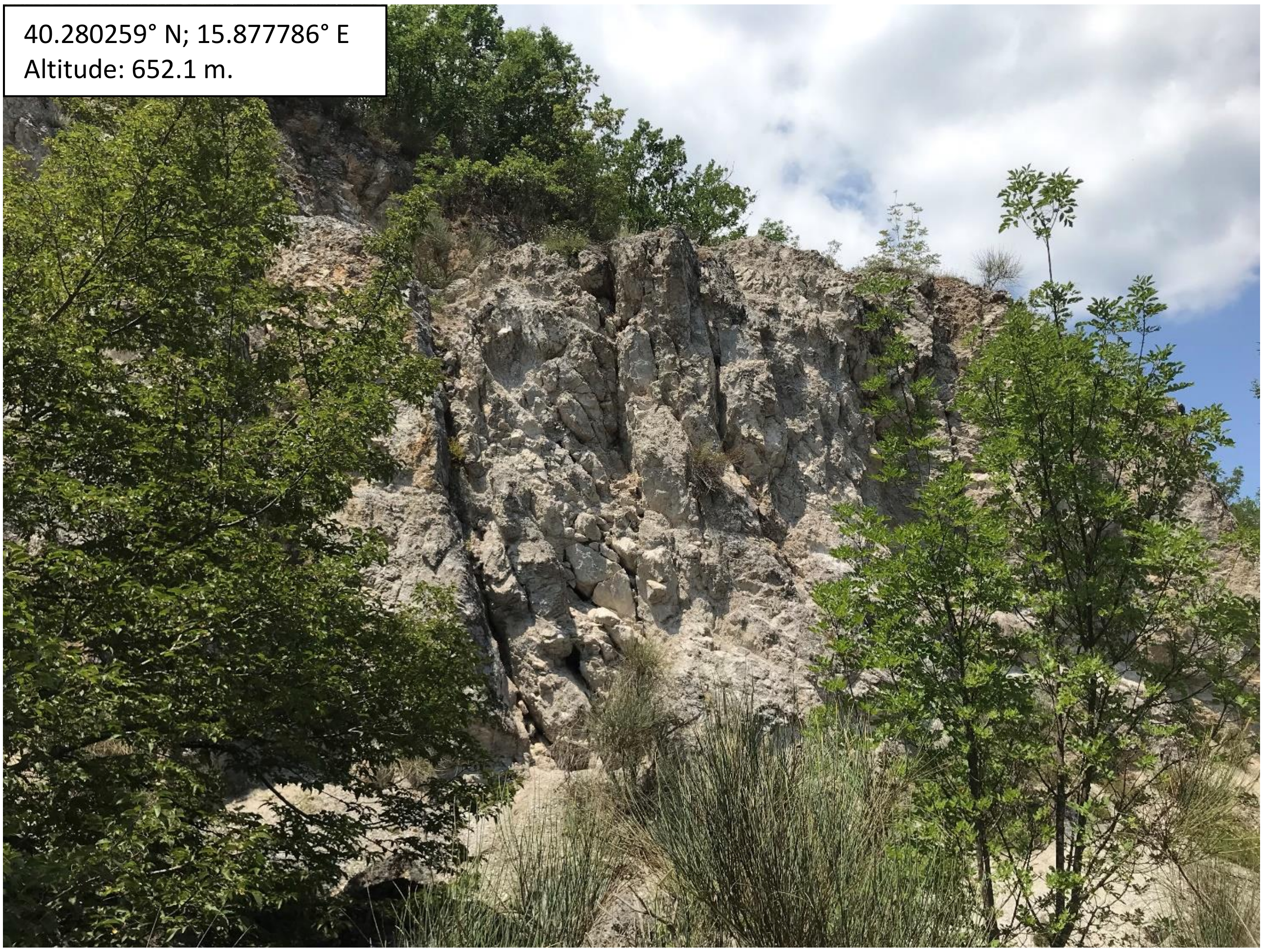

Figure 61.

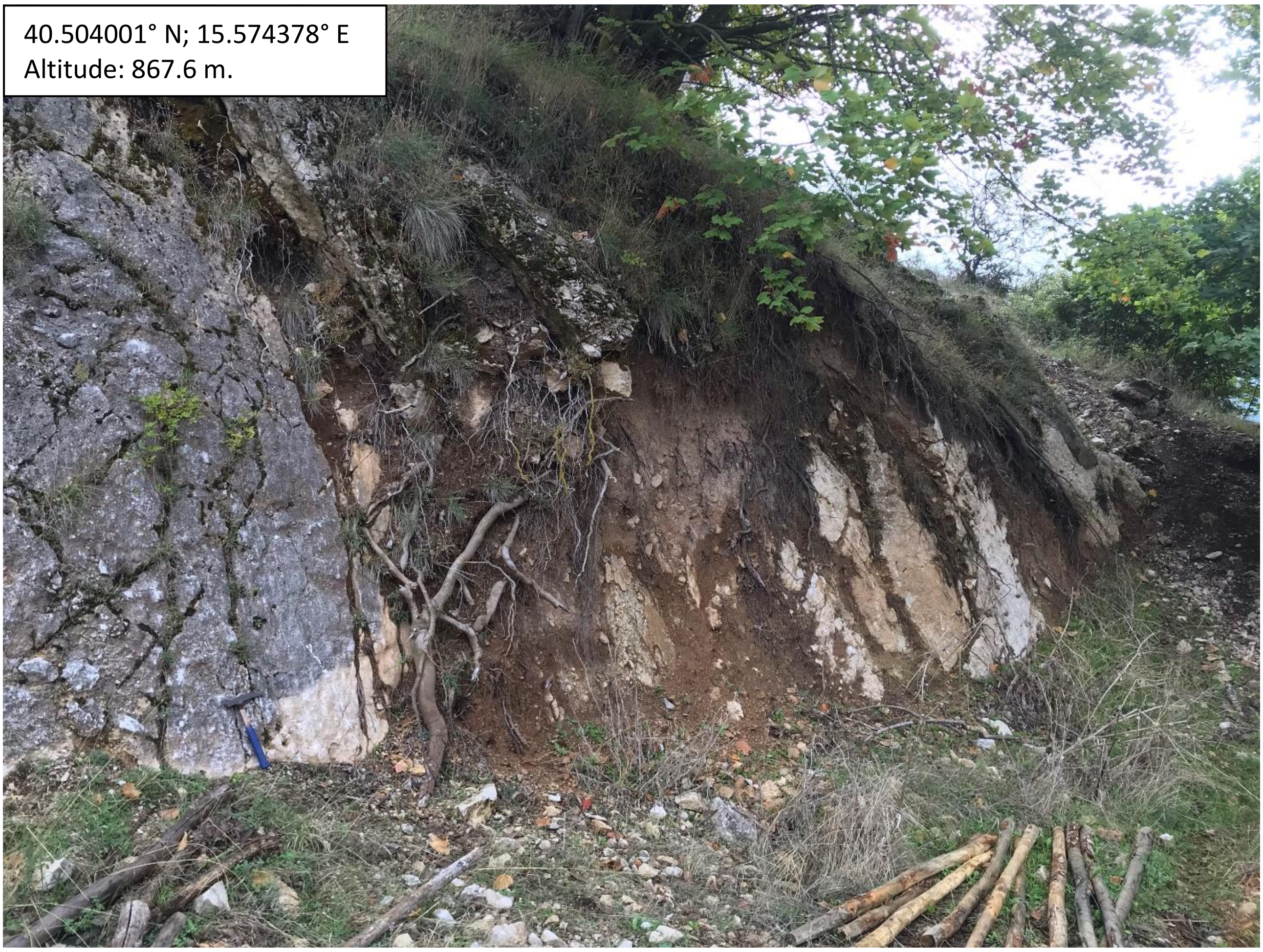

Figure 62.

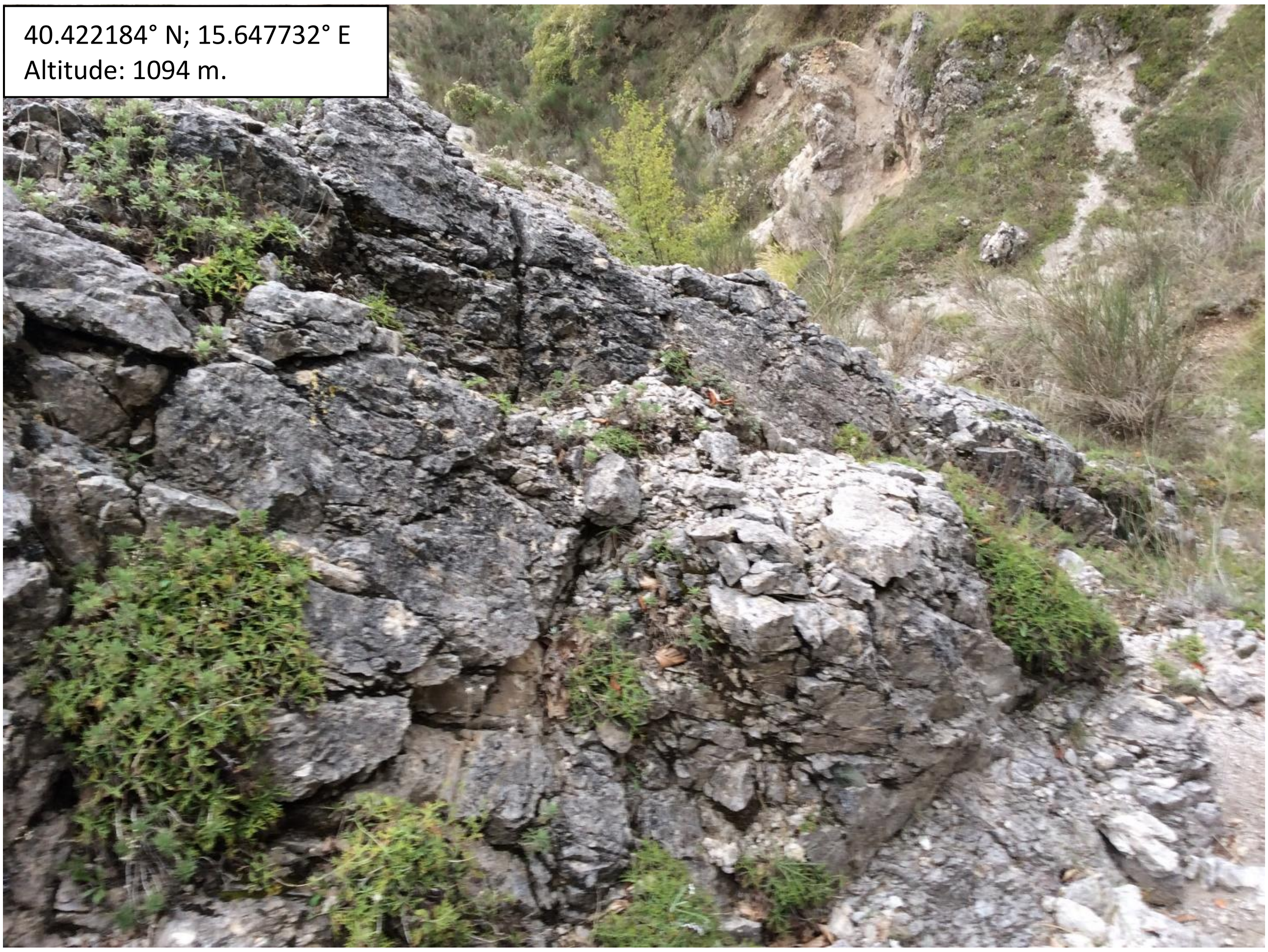

Figure 74.

40.411234° N; 15.656702° E
Altitude: 1073.4 m.

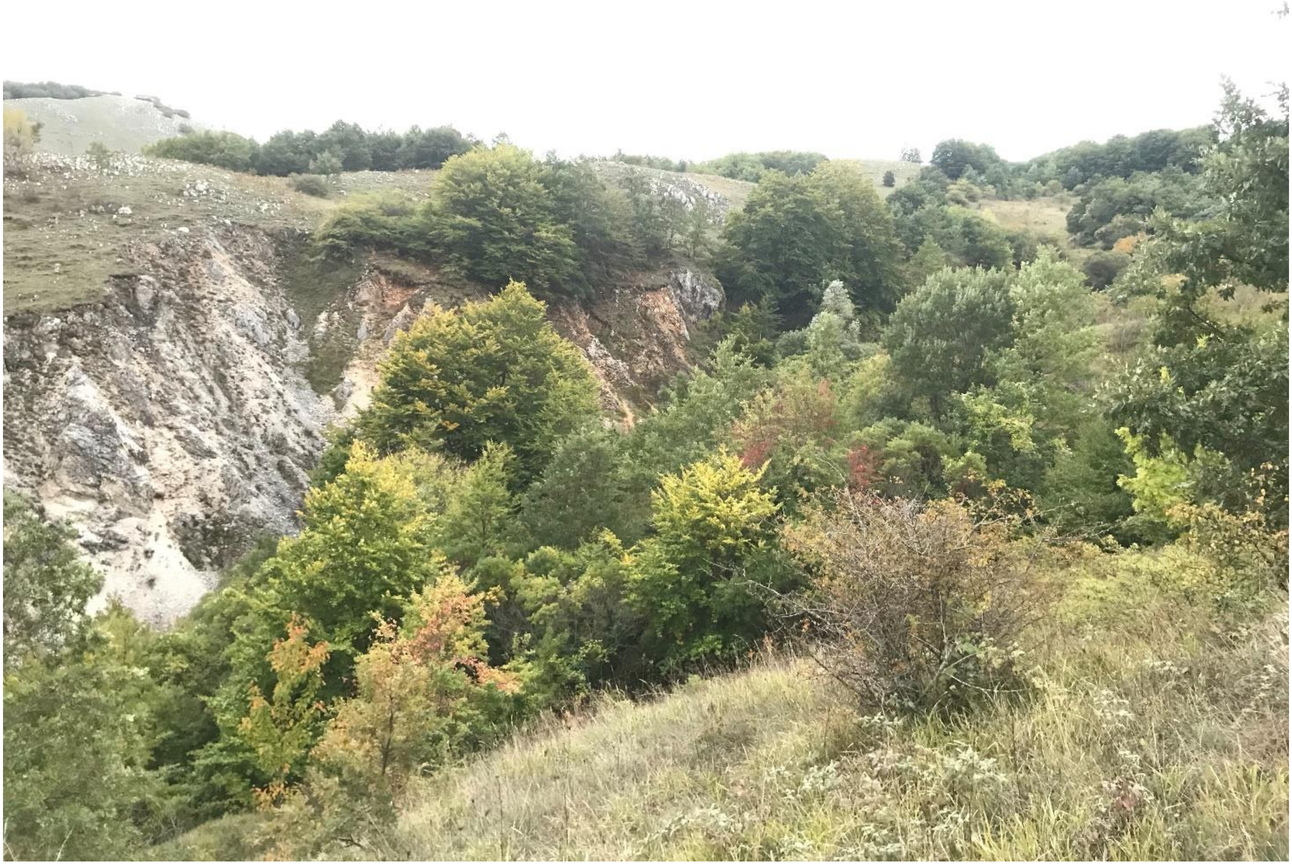

Figure 75.

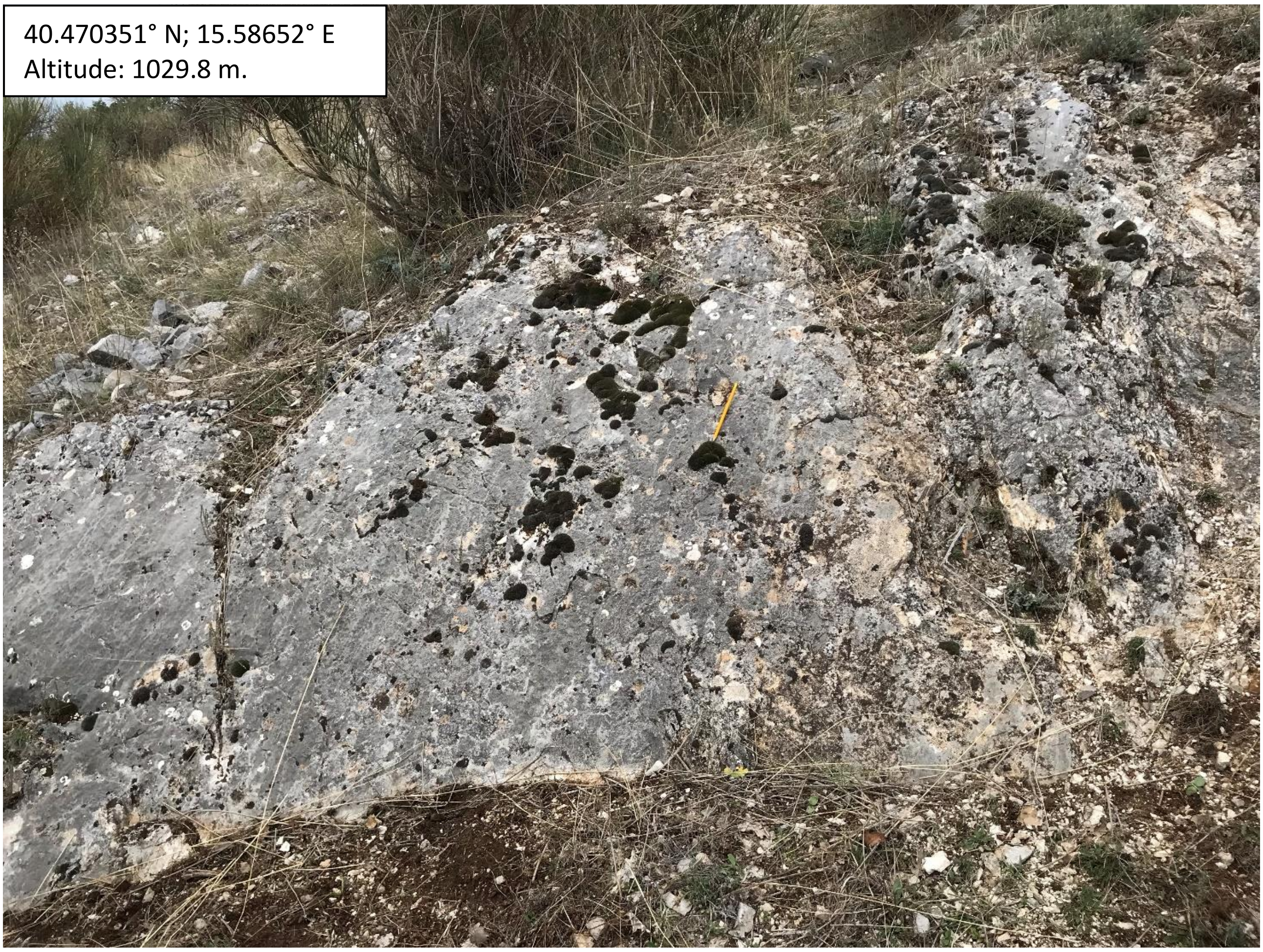

Figure 78.

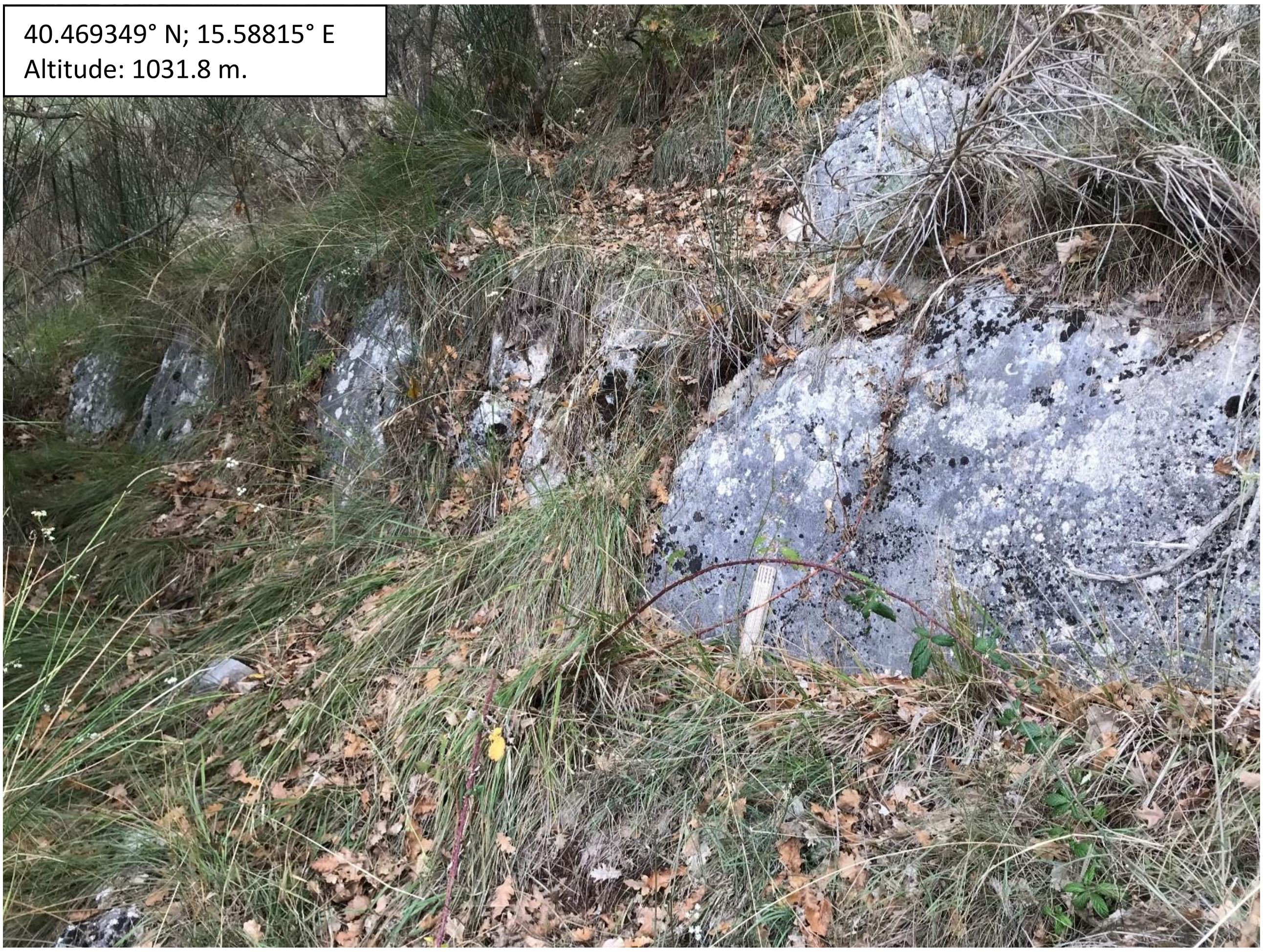

Figure 79.

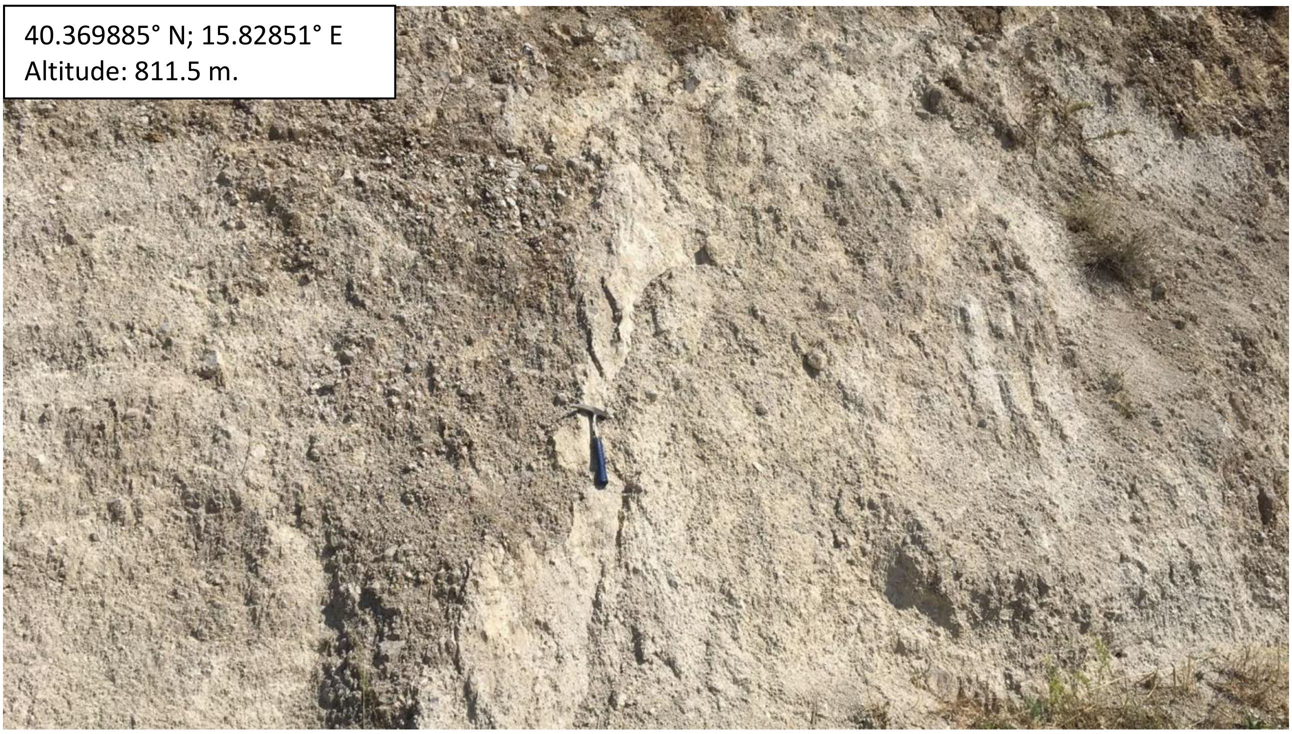

Figure 80.

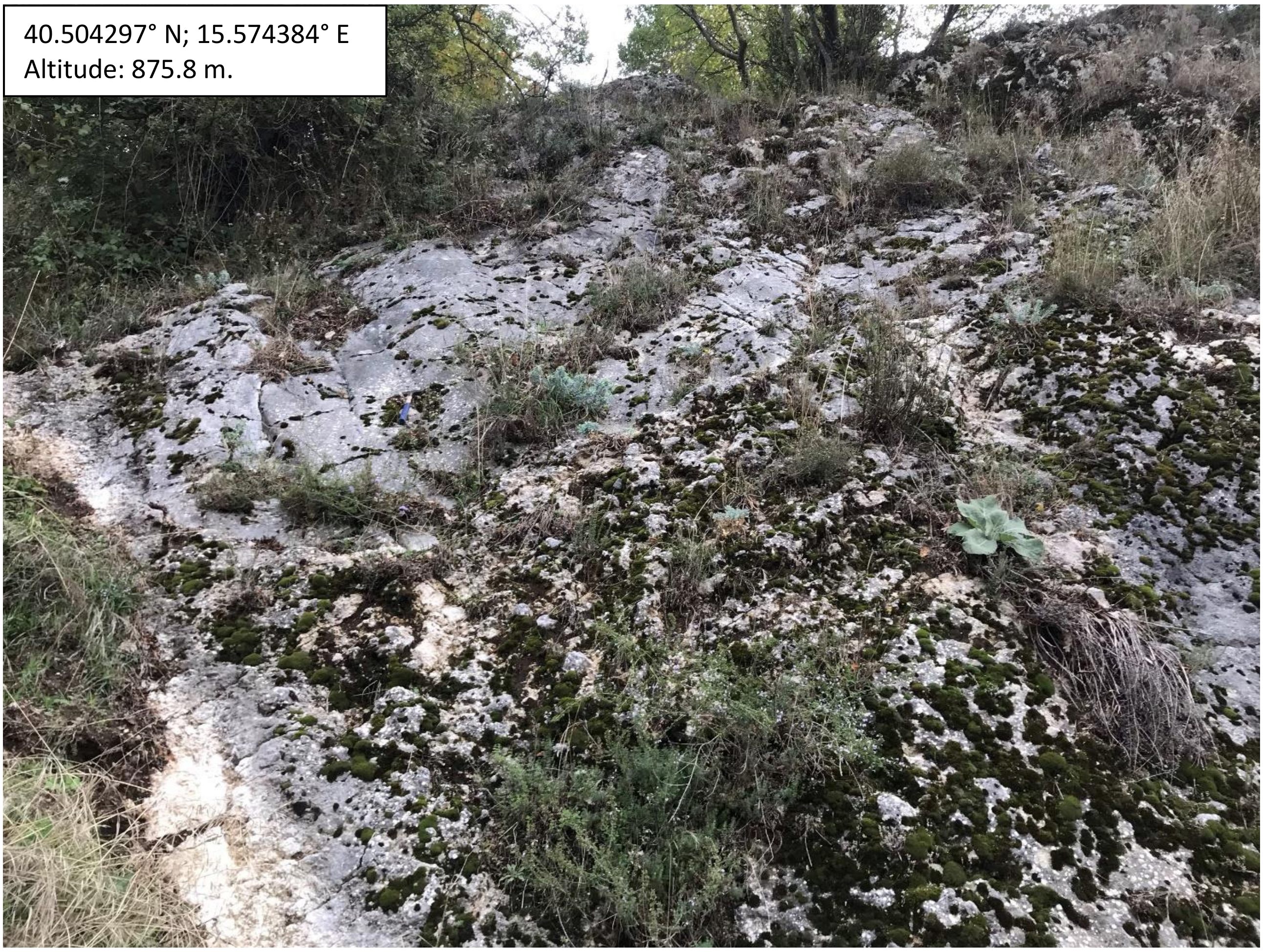

Figure 81.

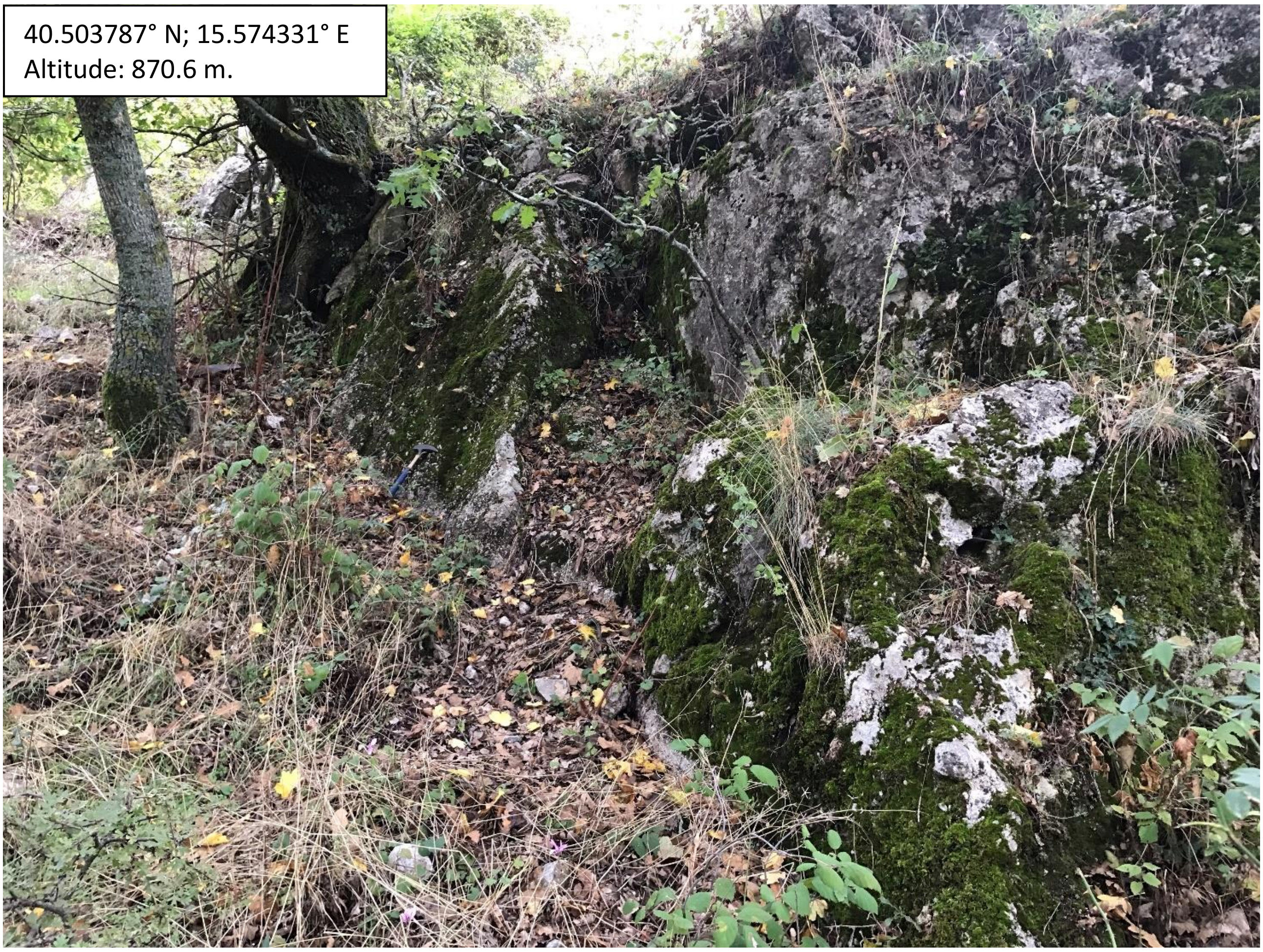

Figure 82.

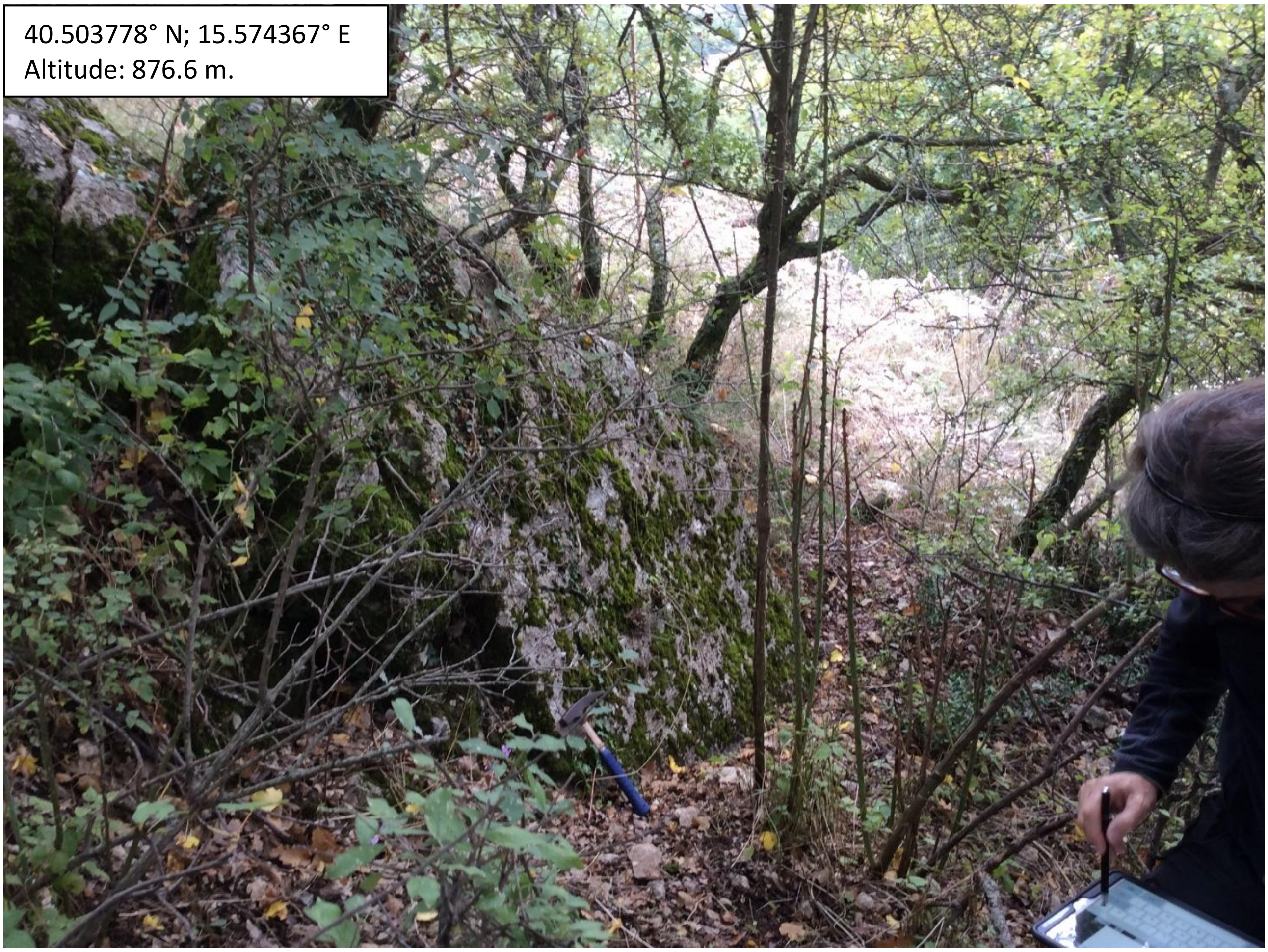

Figure 83.

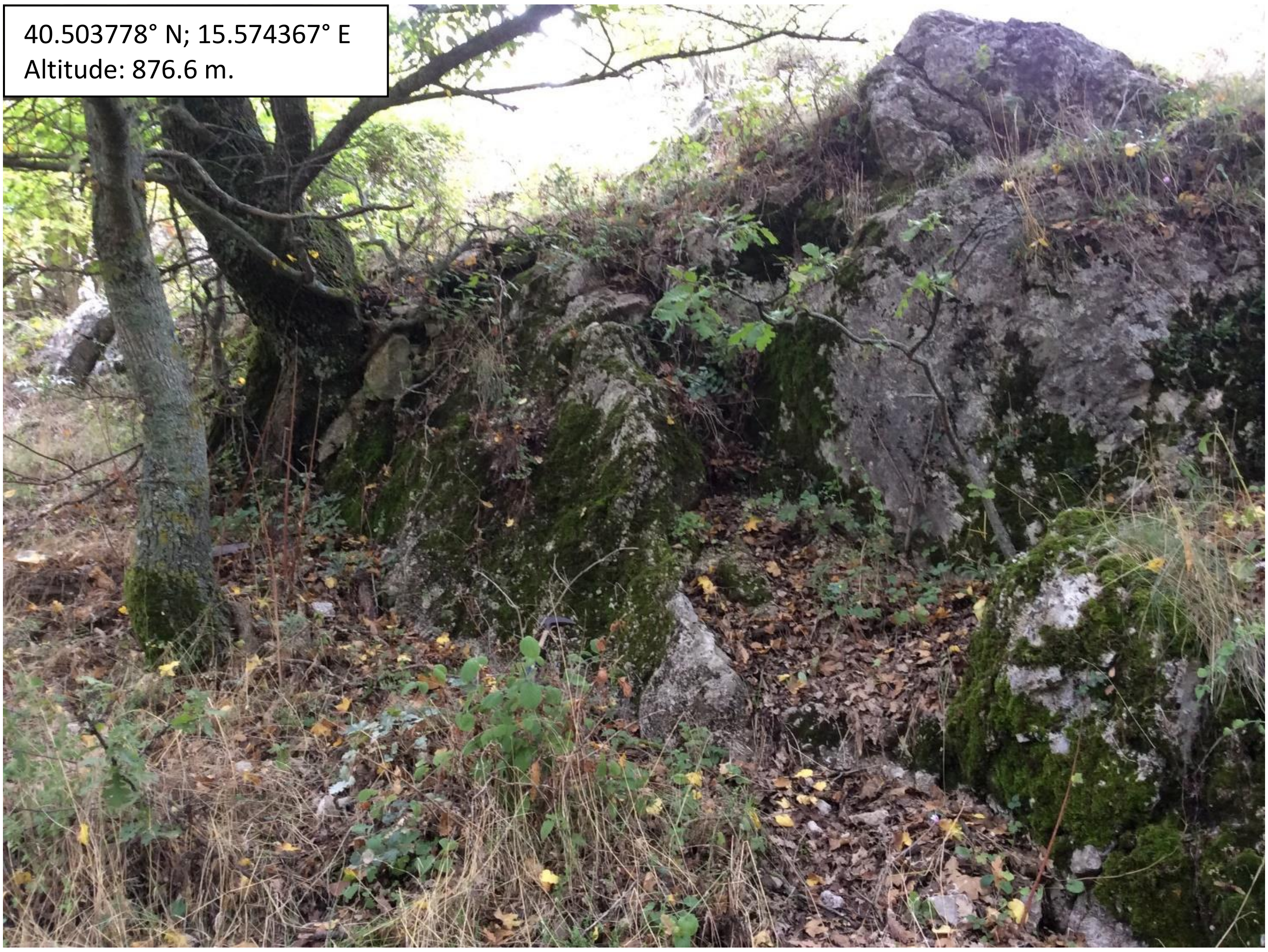

Figure 84.

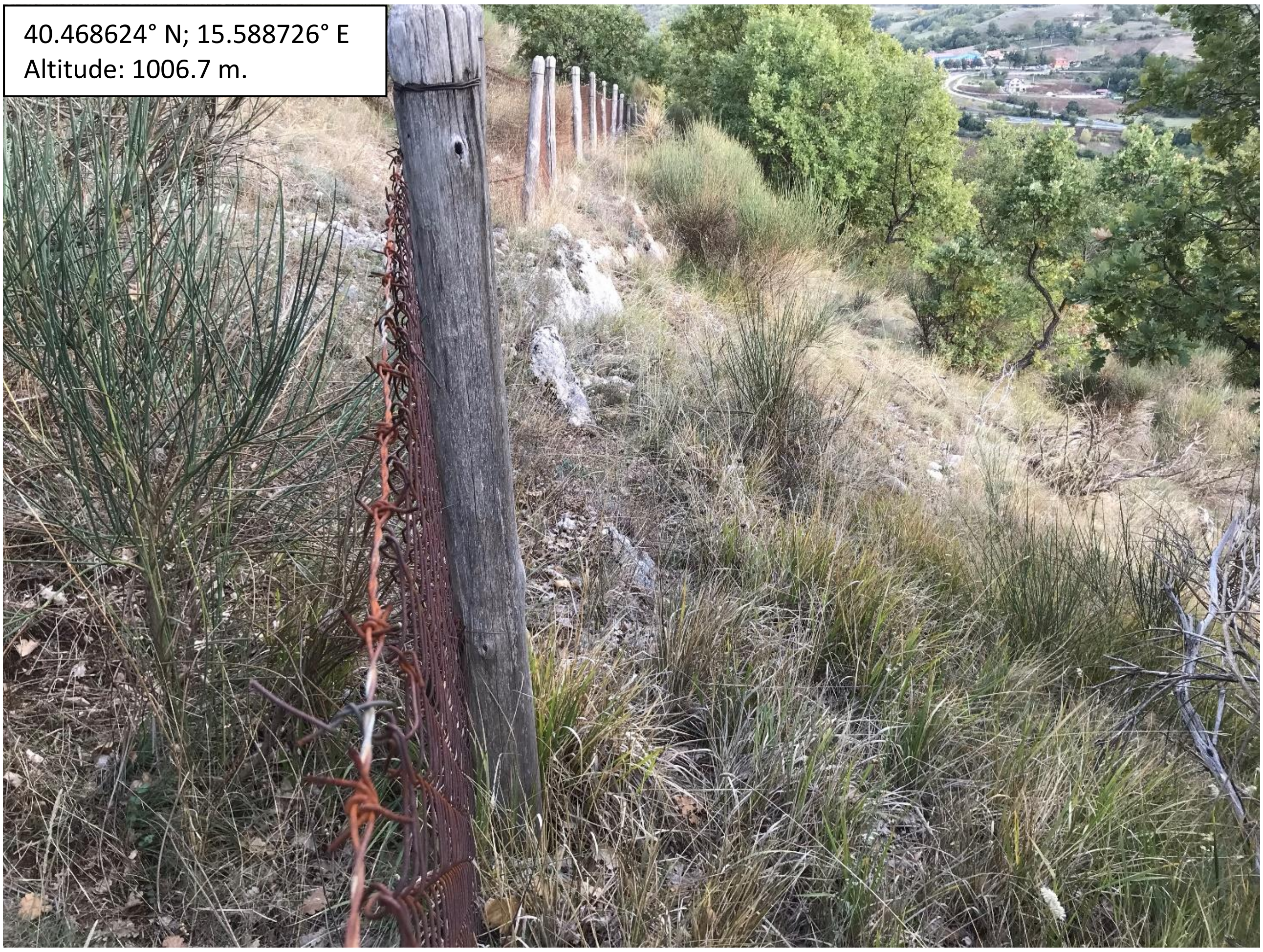

Figure 85.

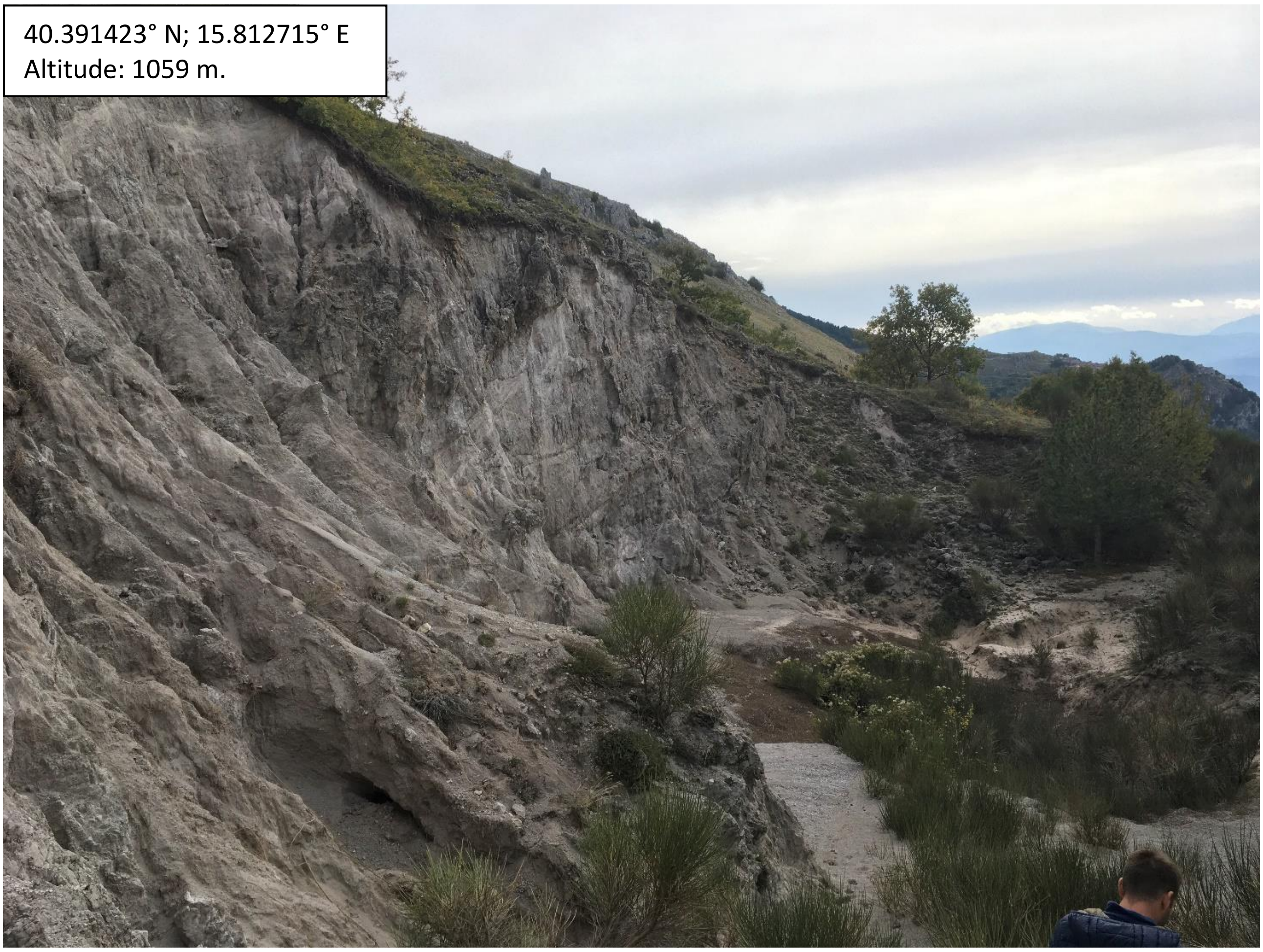

Figure 86.

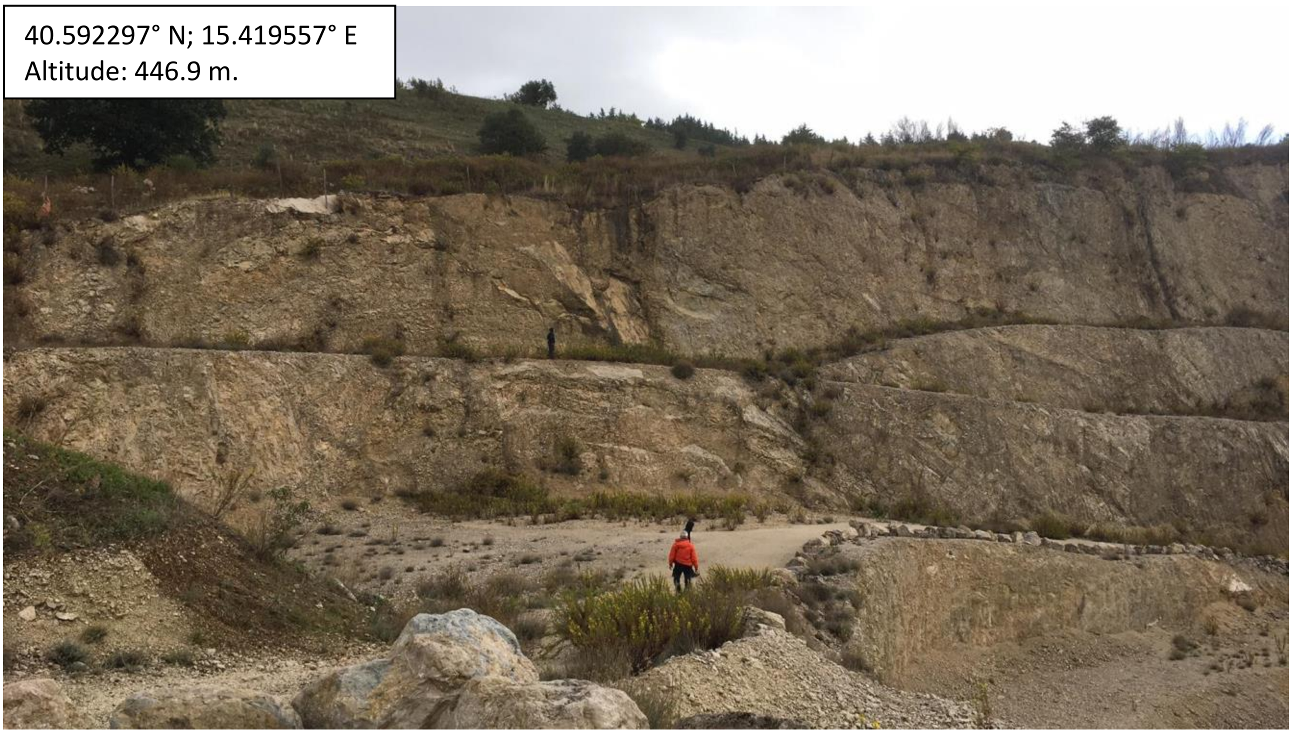

Figure 87.

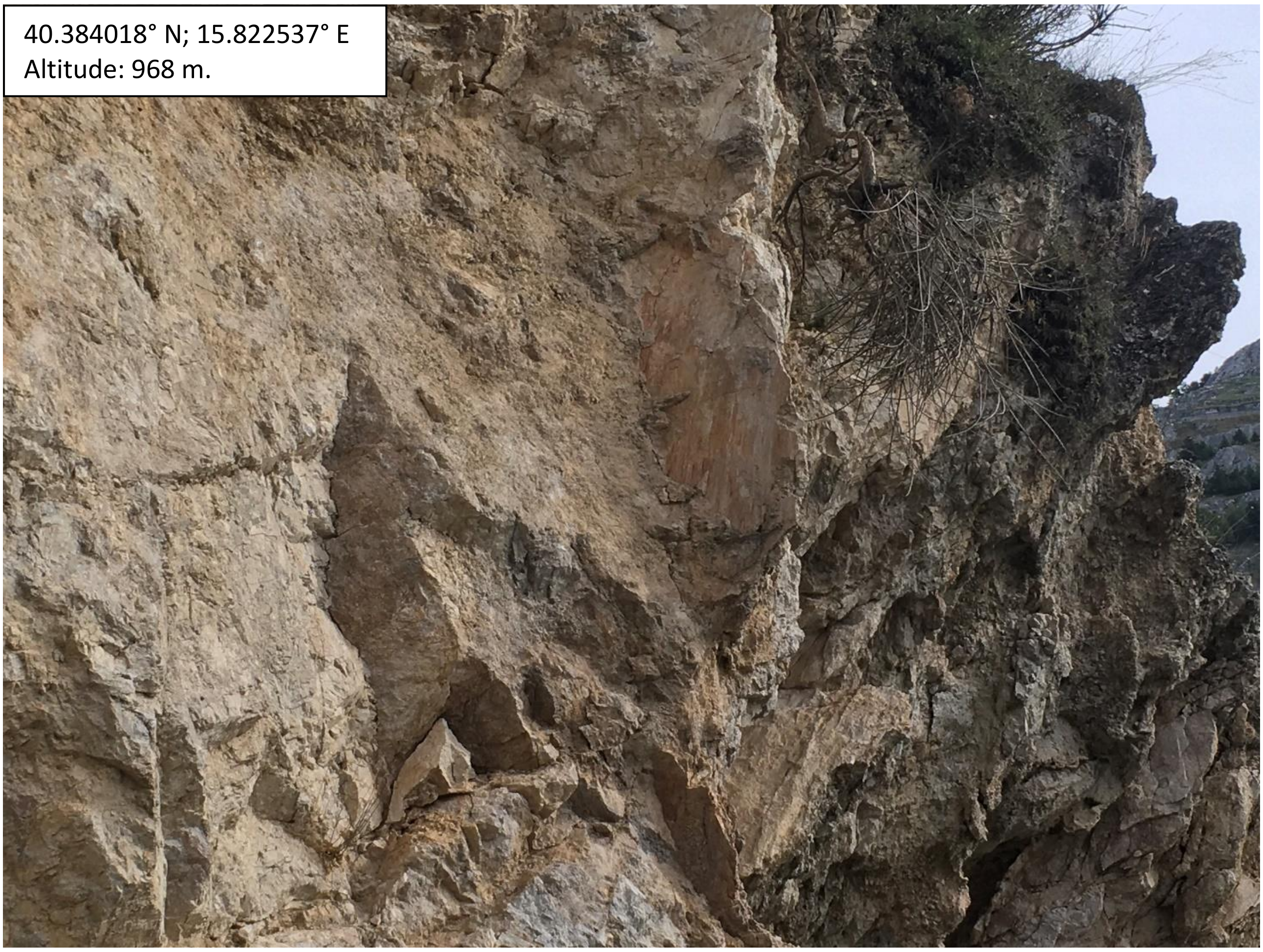

Figure 88

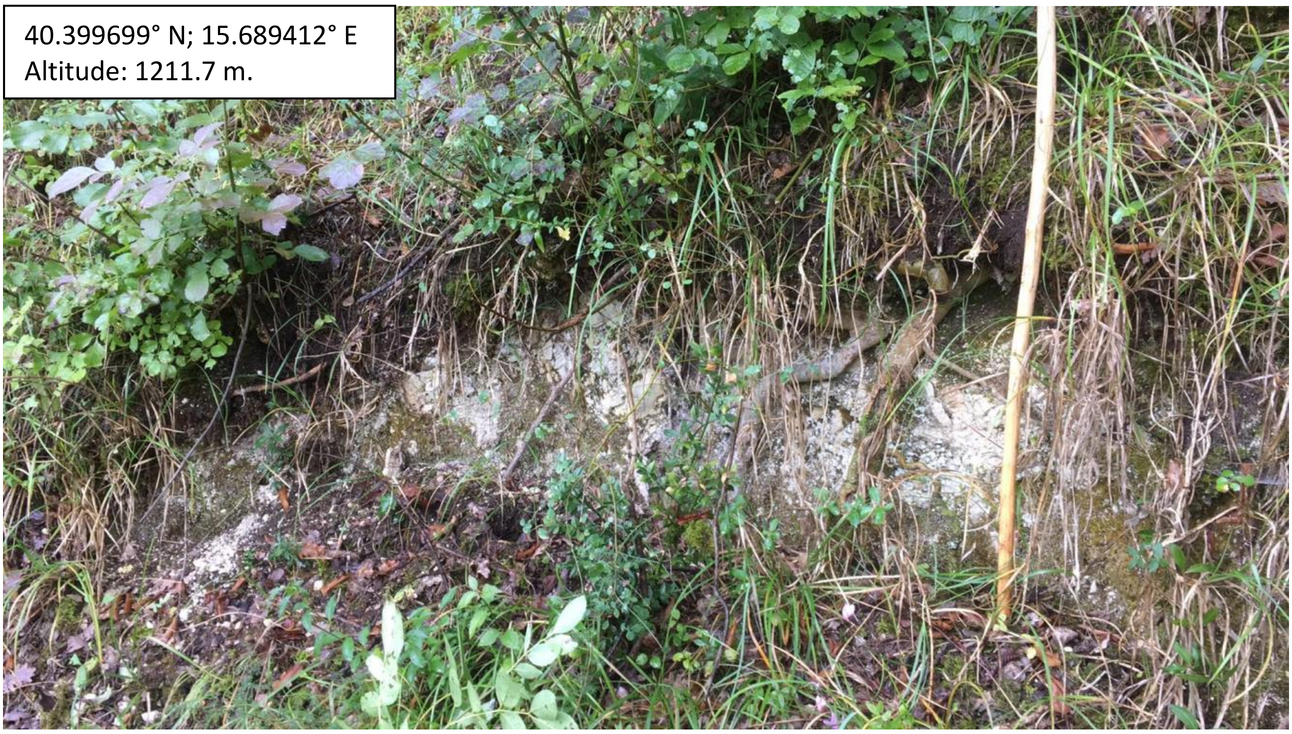

Figure 89.

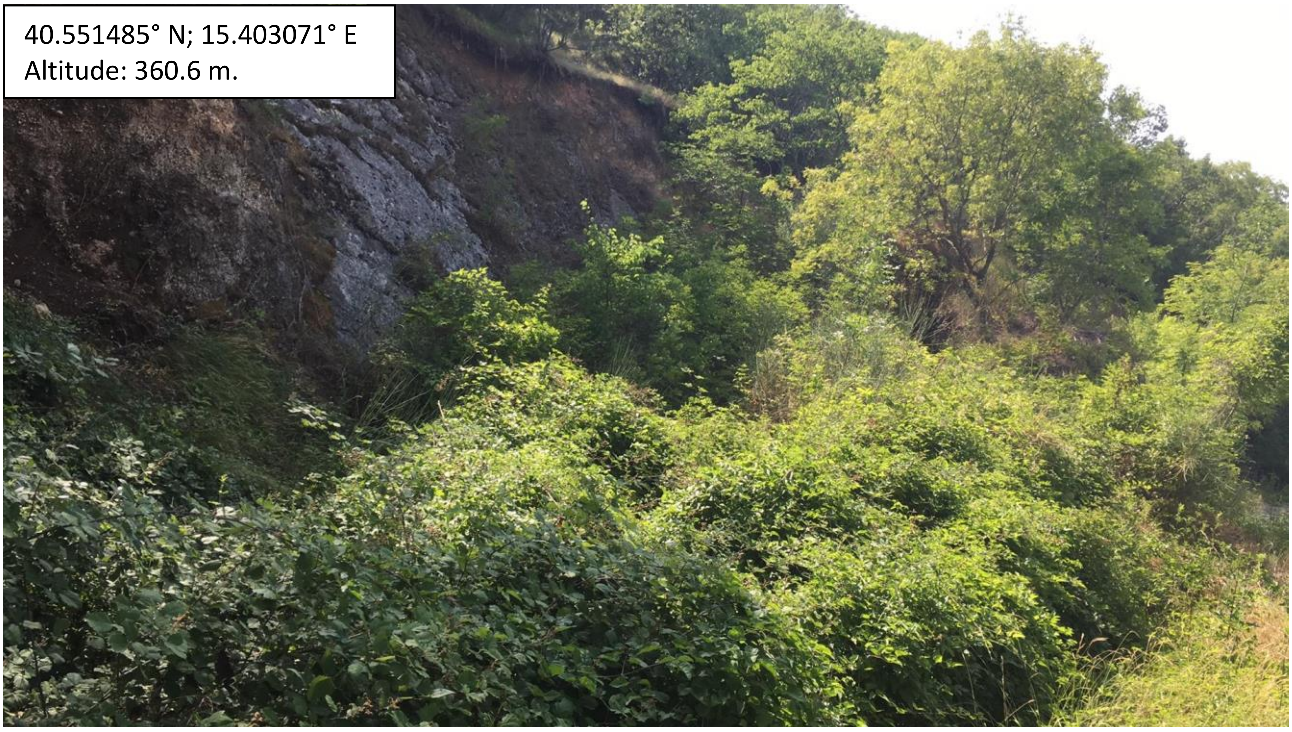

Figure 90.

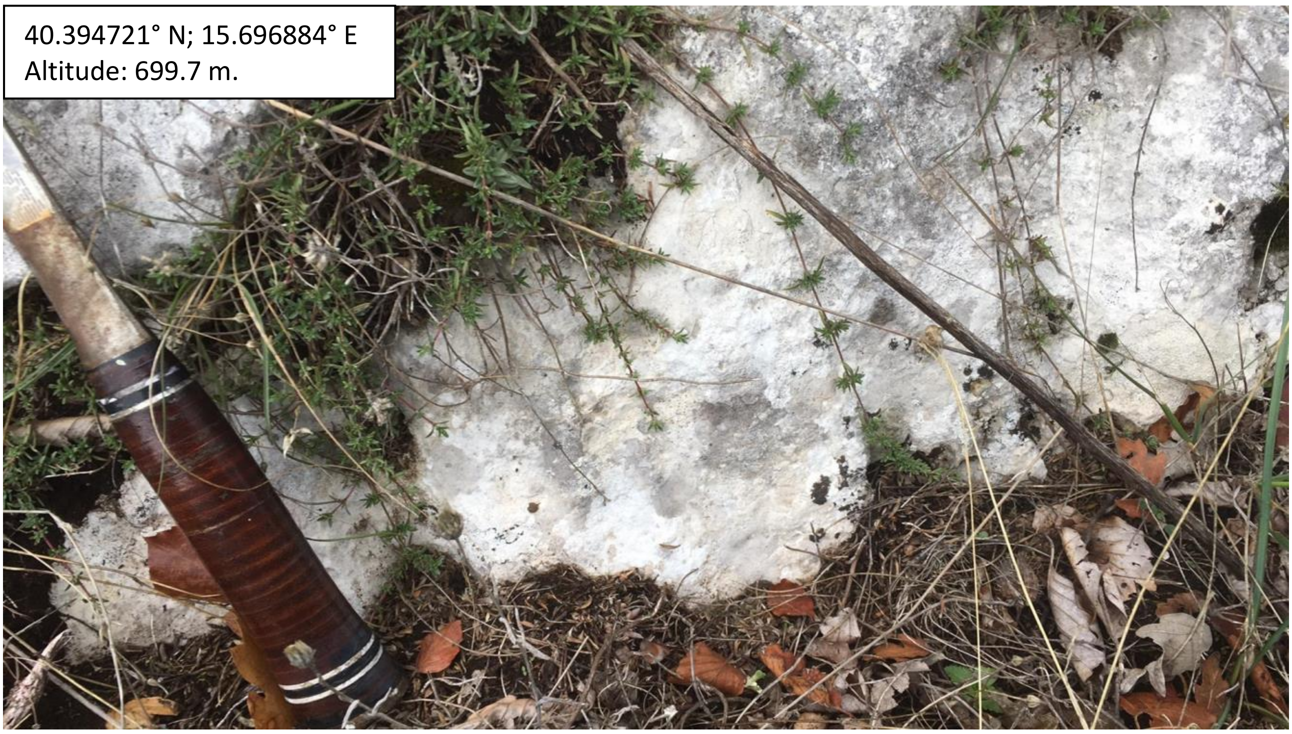

Figure 91.

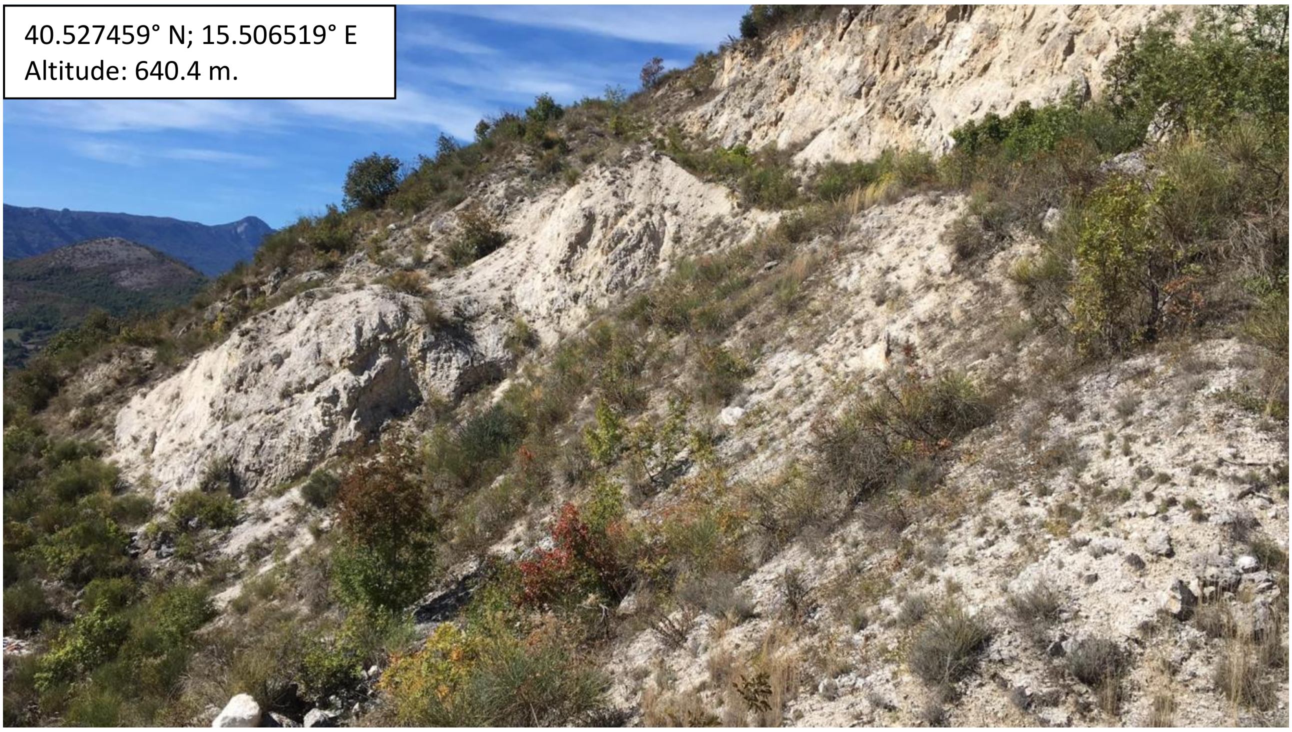

Figure 92.

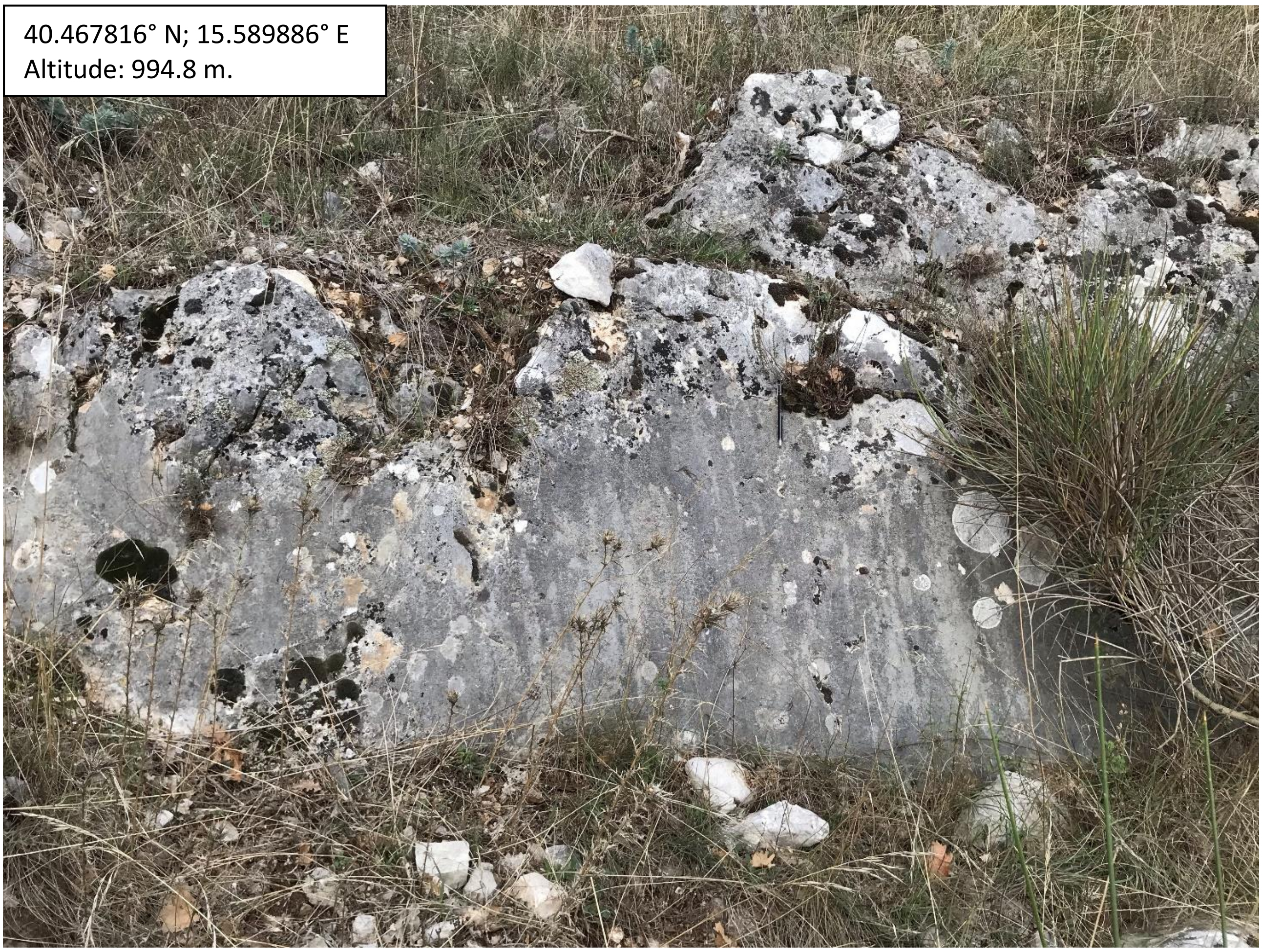

Figure 93.

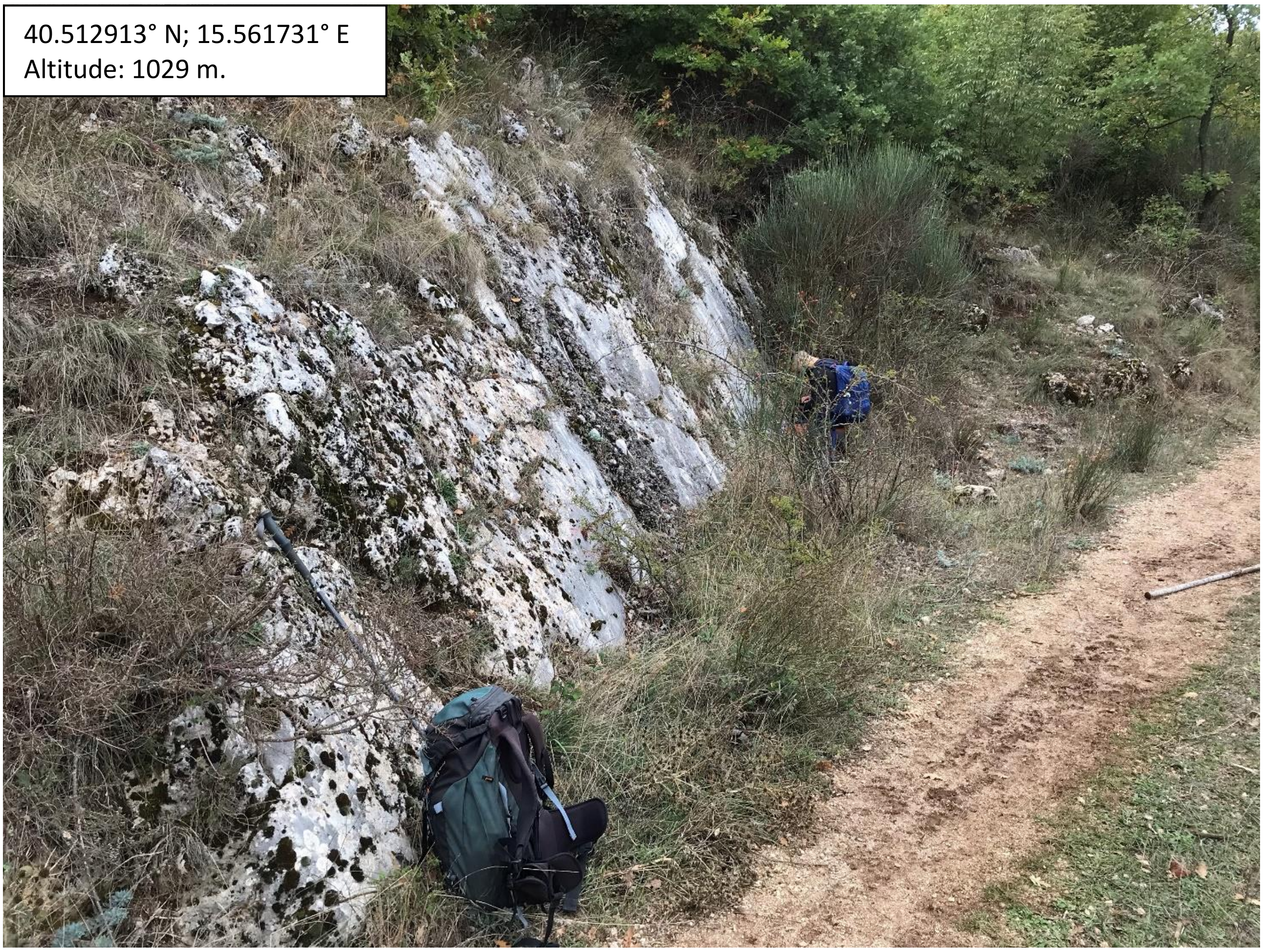

Figure 95.

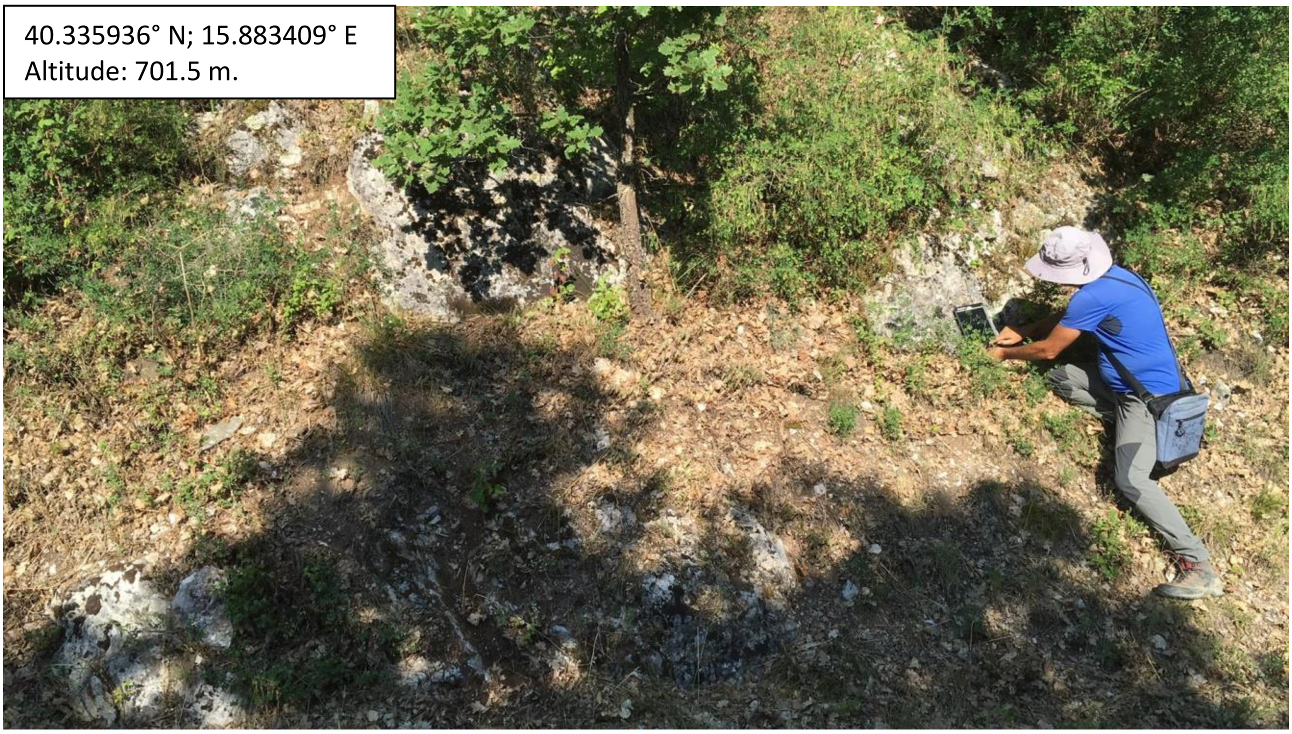

Figure 96.

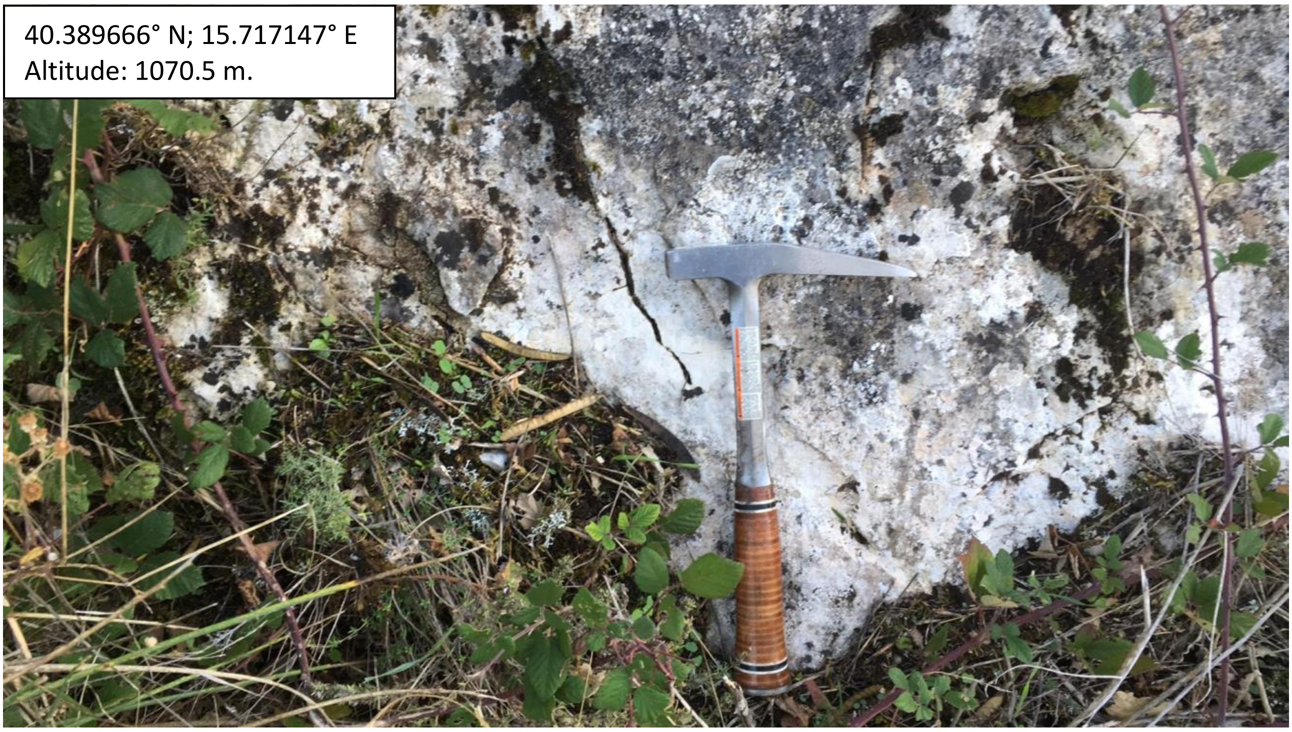

Figure 97.

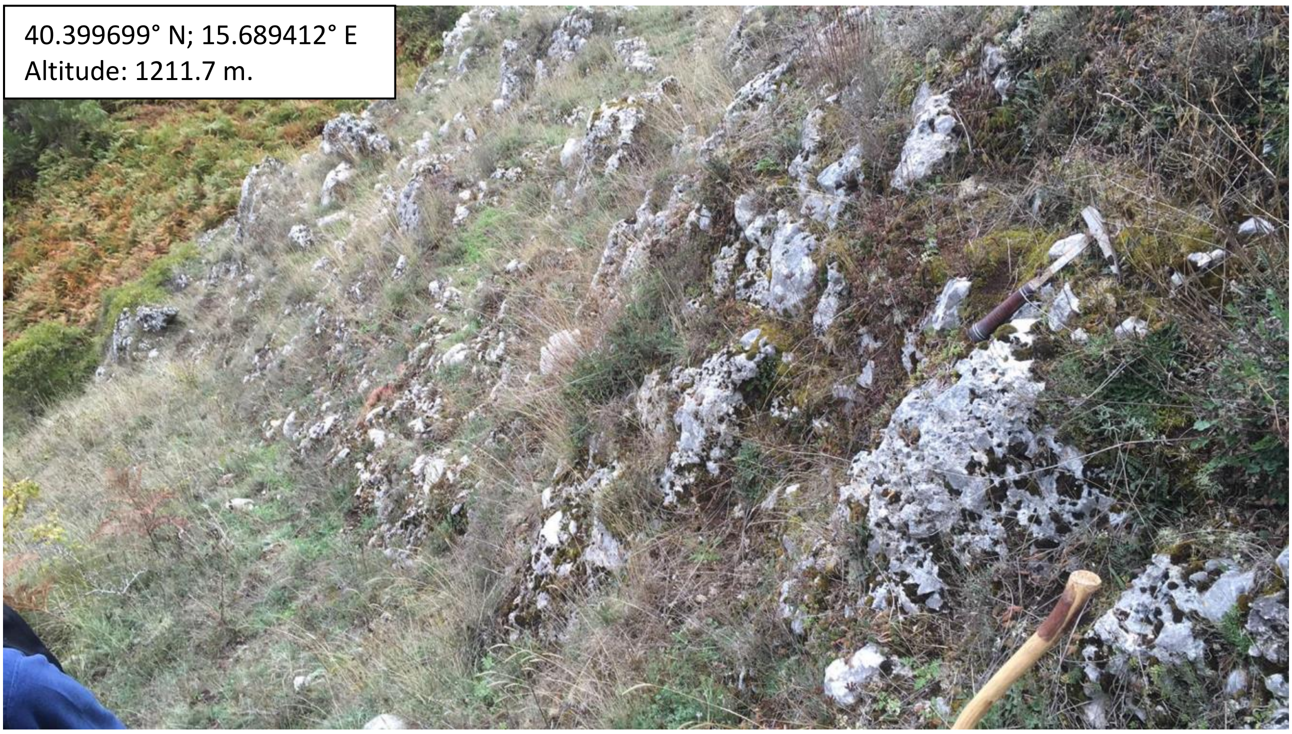

Figure 98.

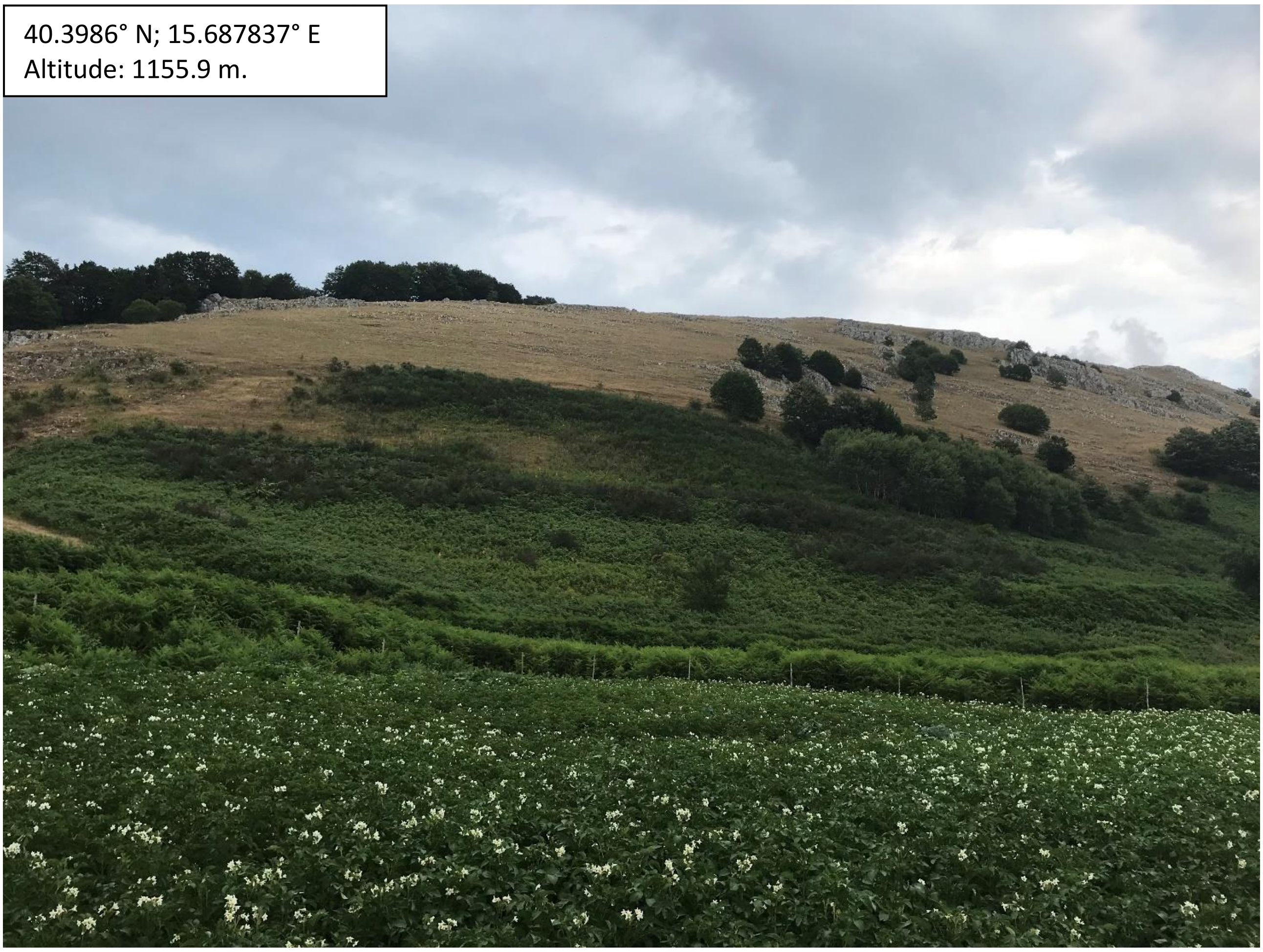

Figure 99.

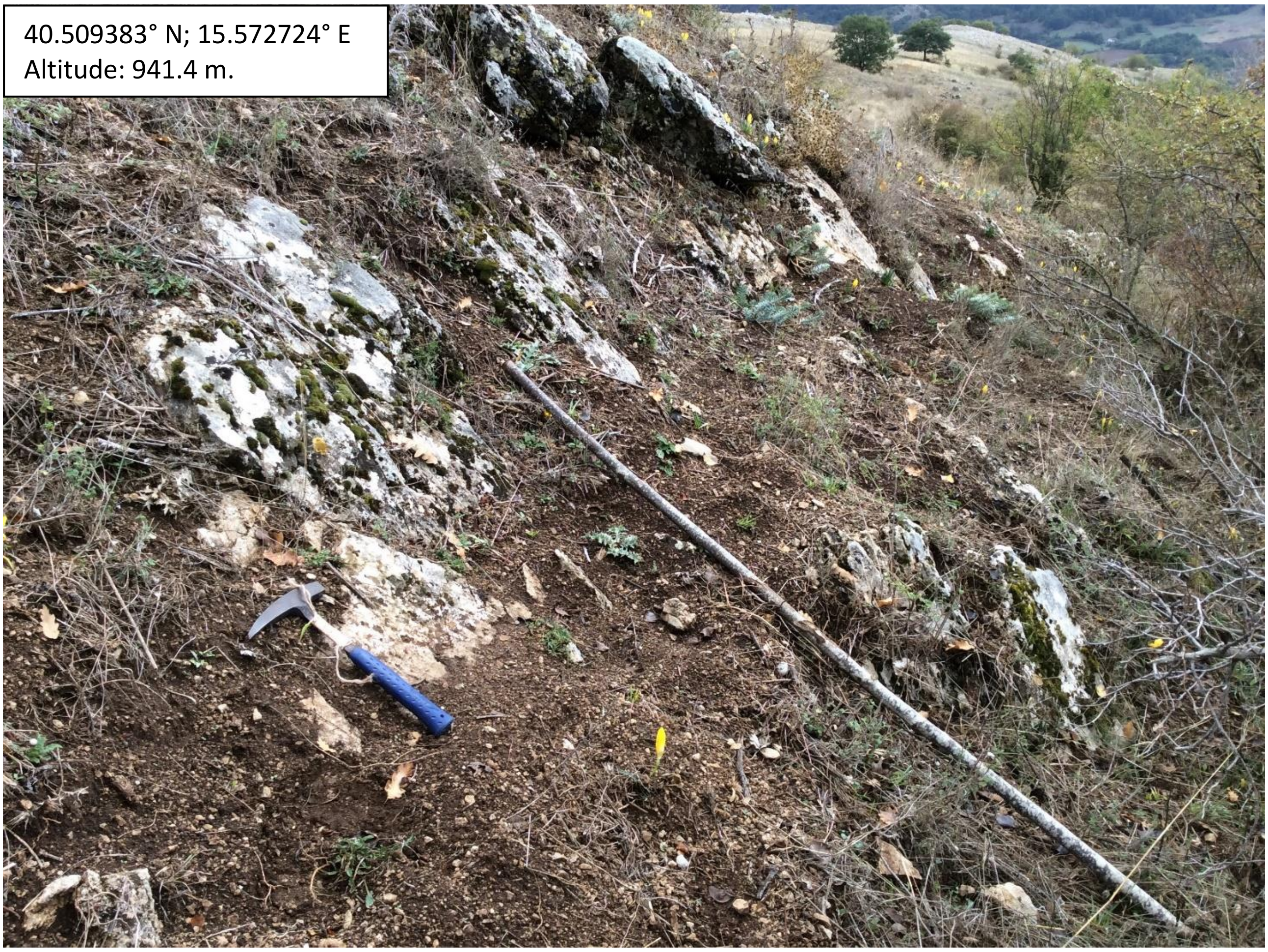

Figure 100.

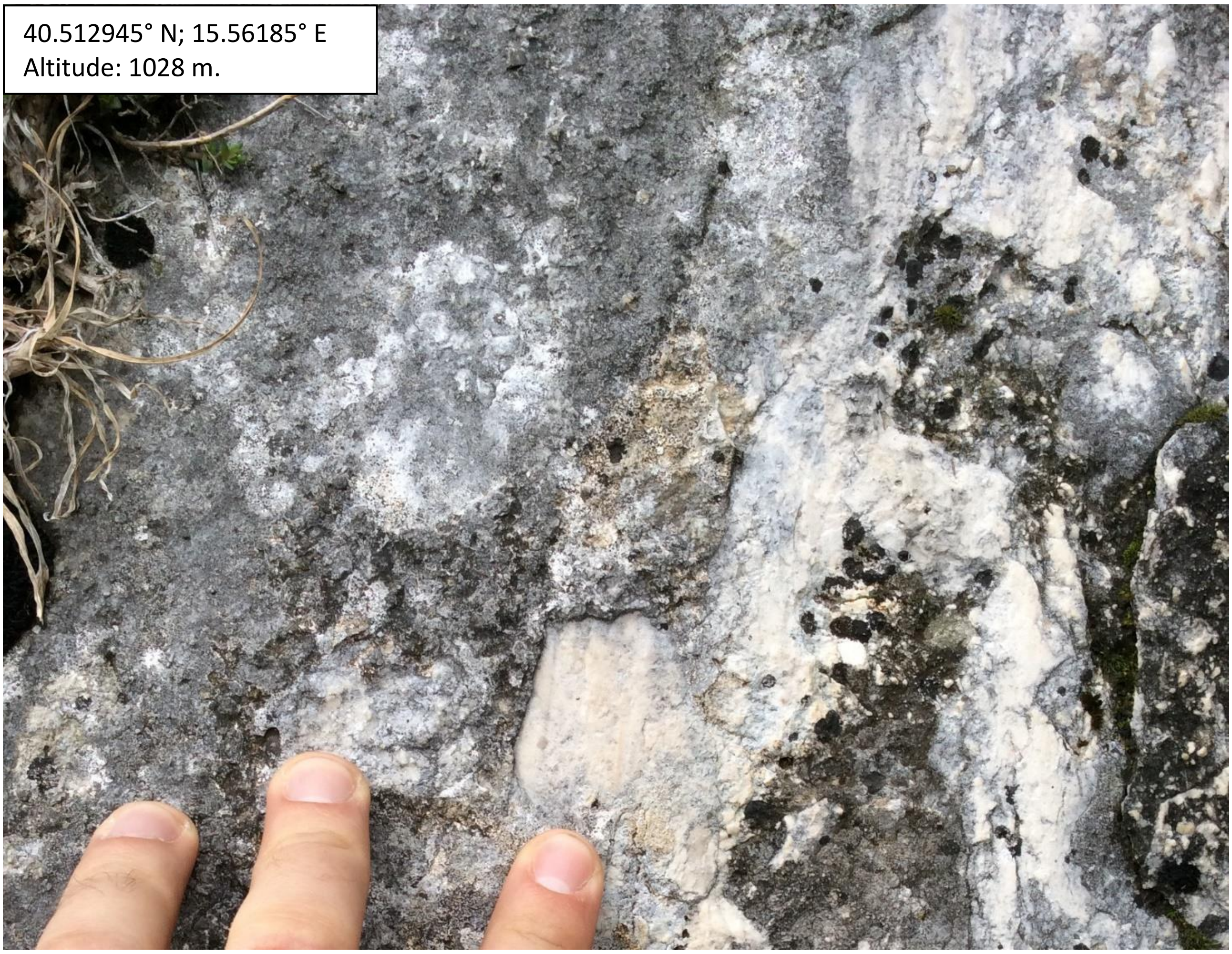

Figure 101.

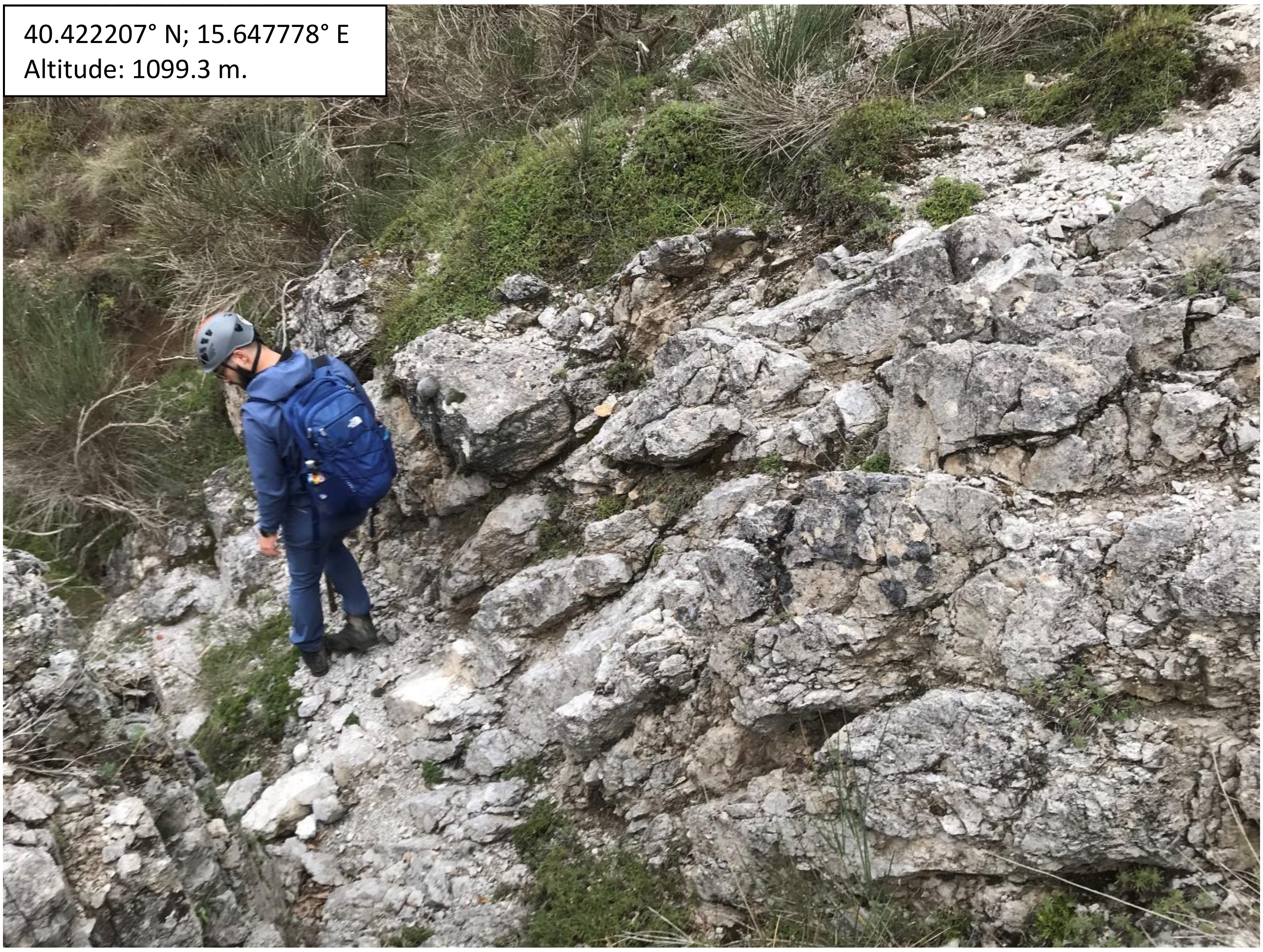

Figure 102.

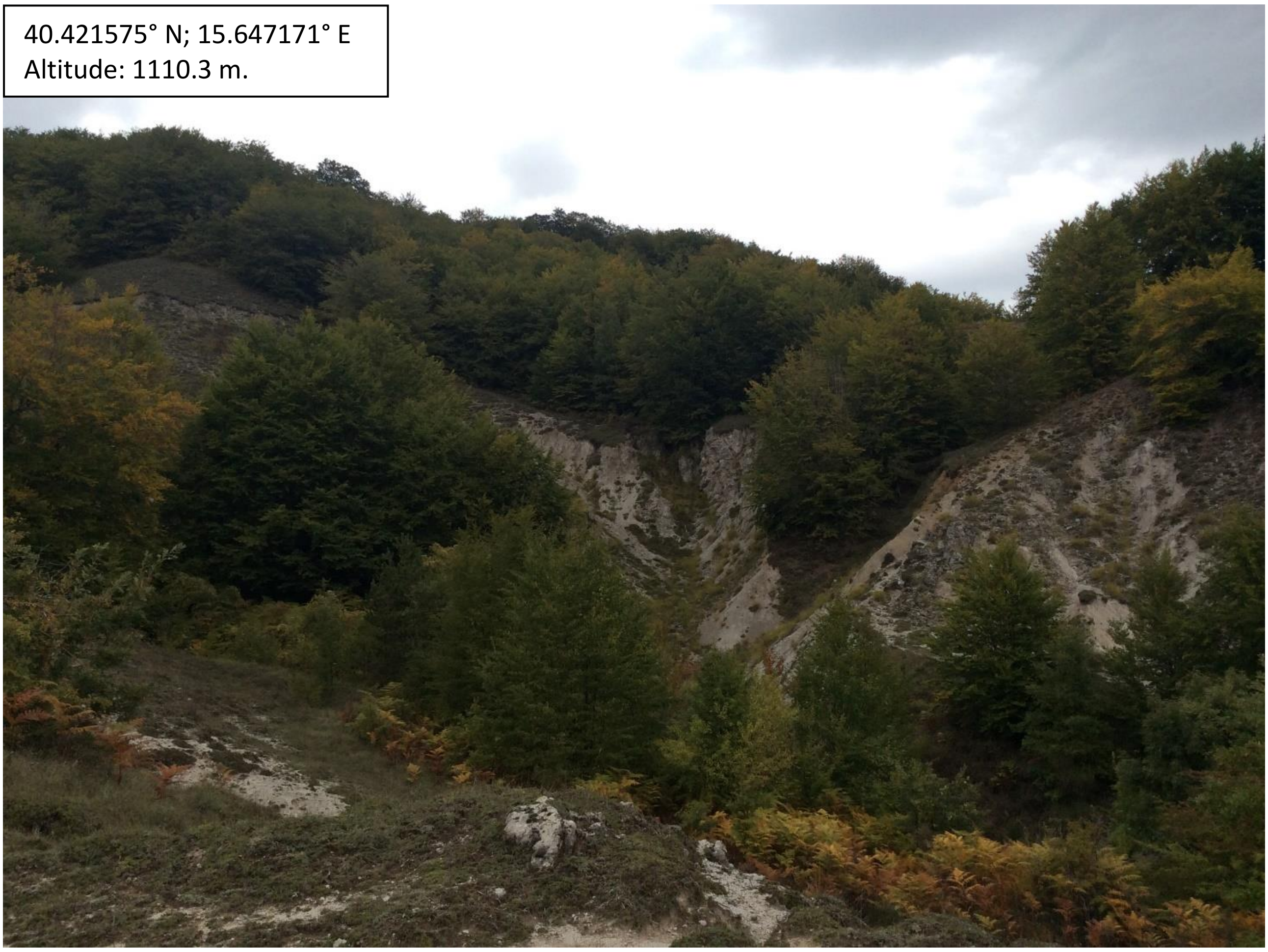

Figure 103.

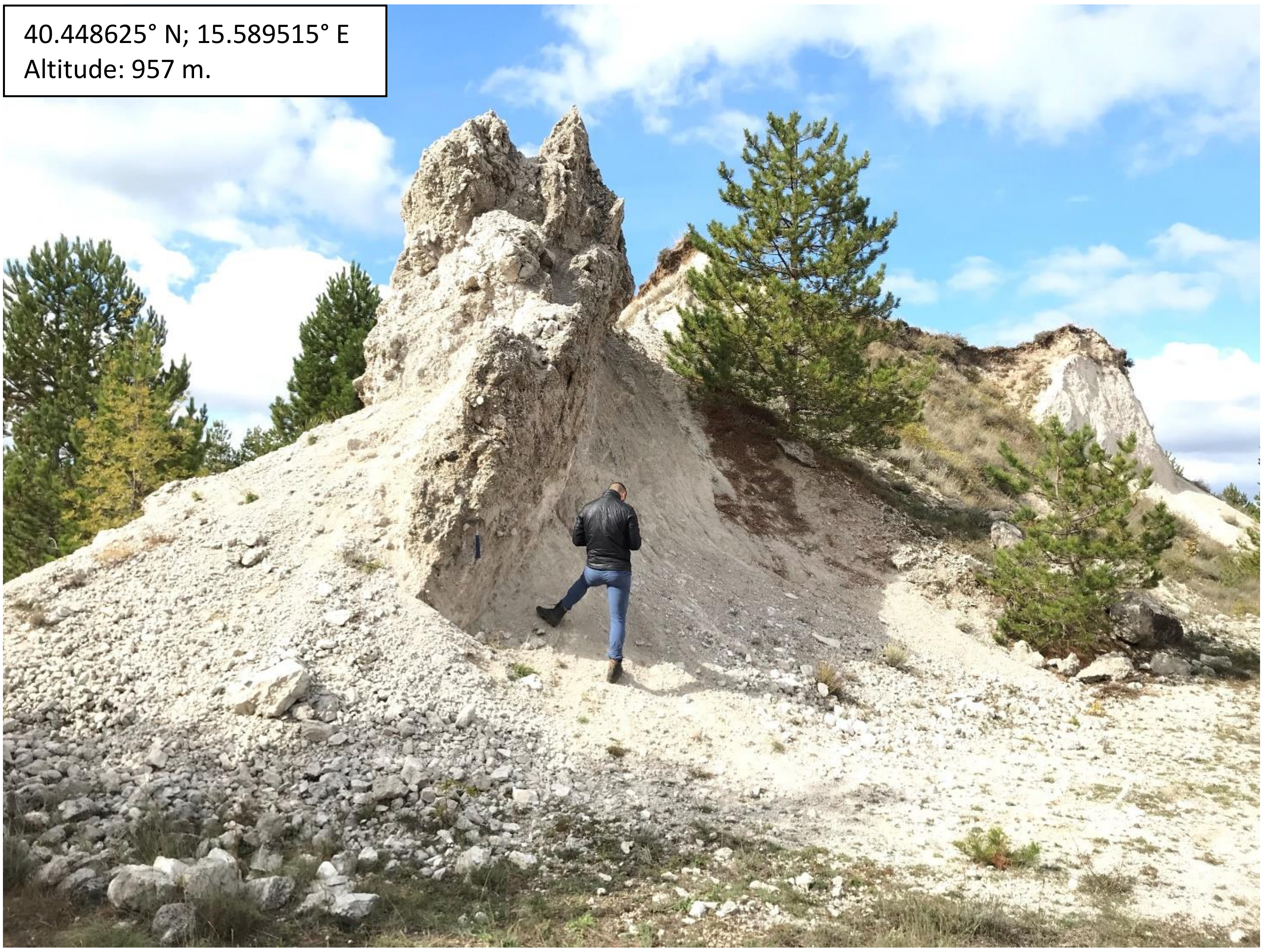

Figure 104.

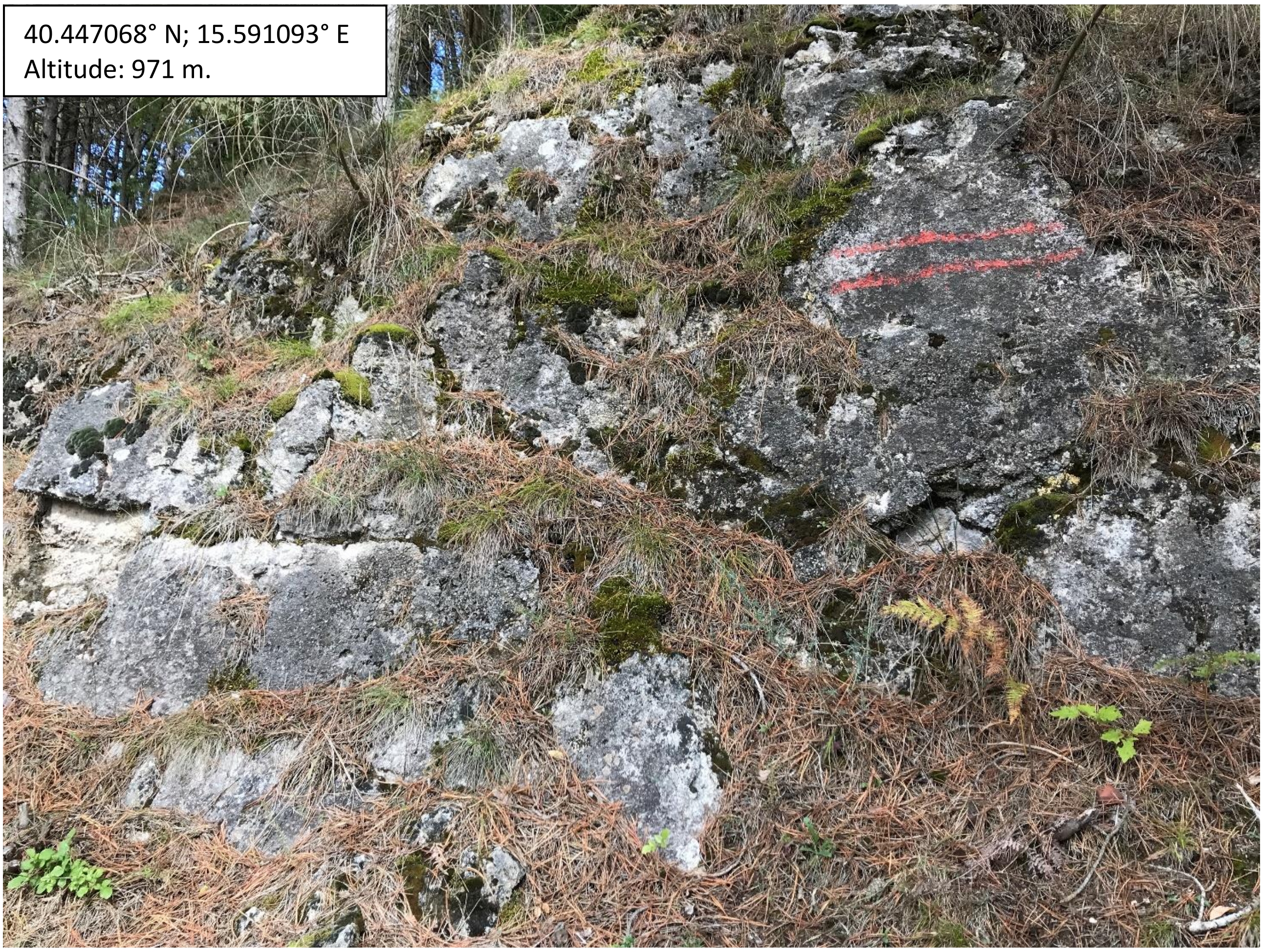

Figure 105.

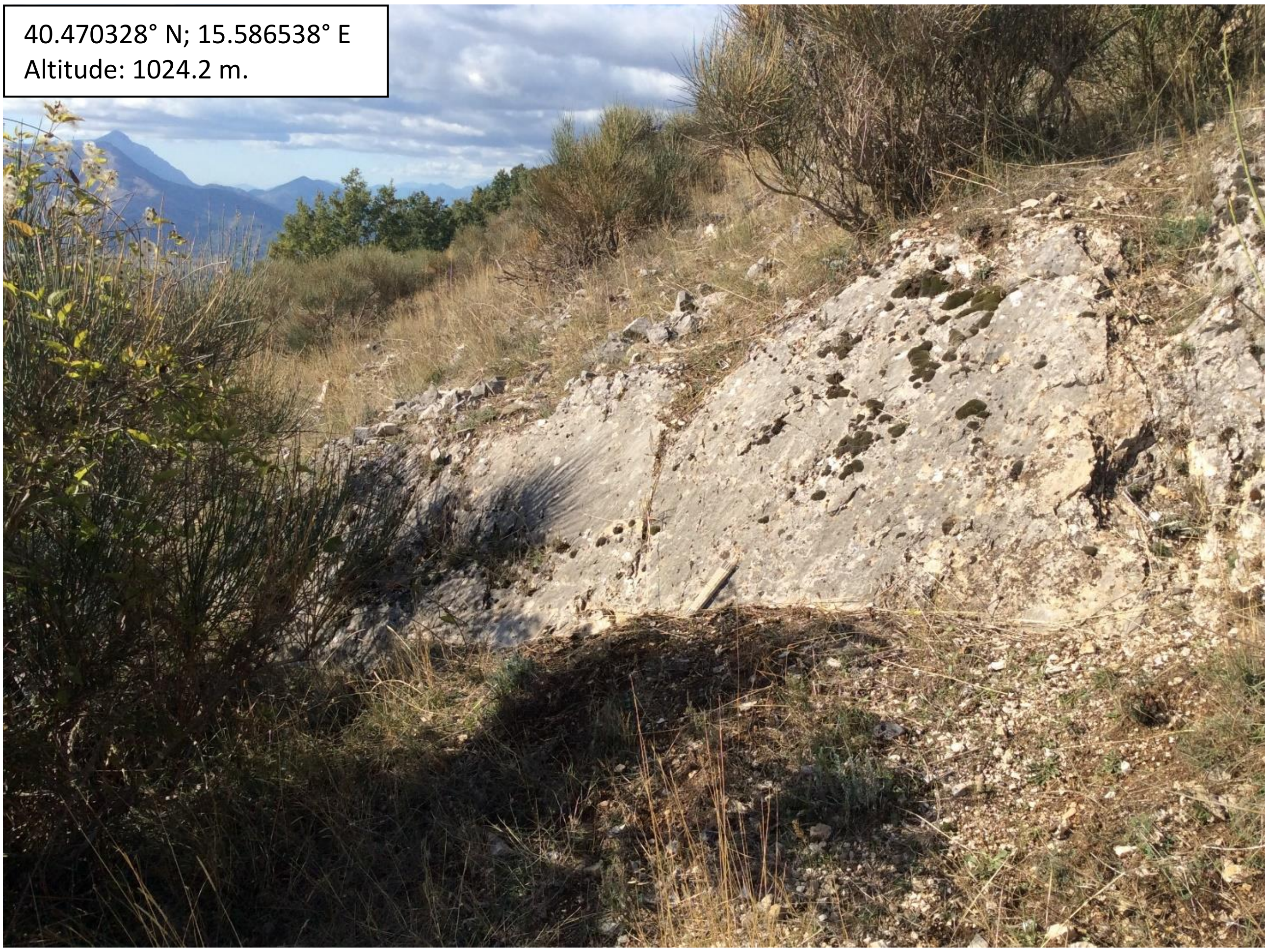

Figure 106.

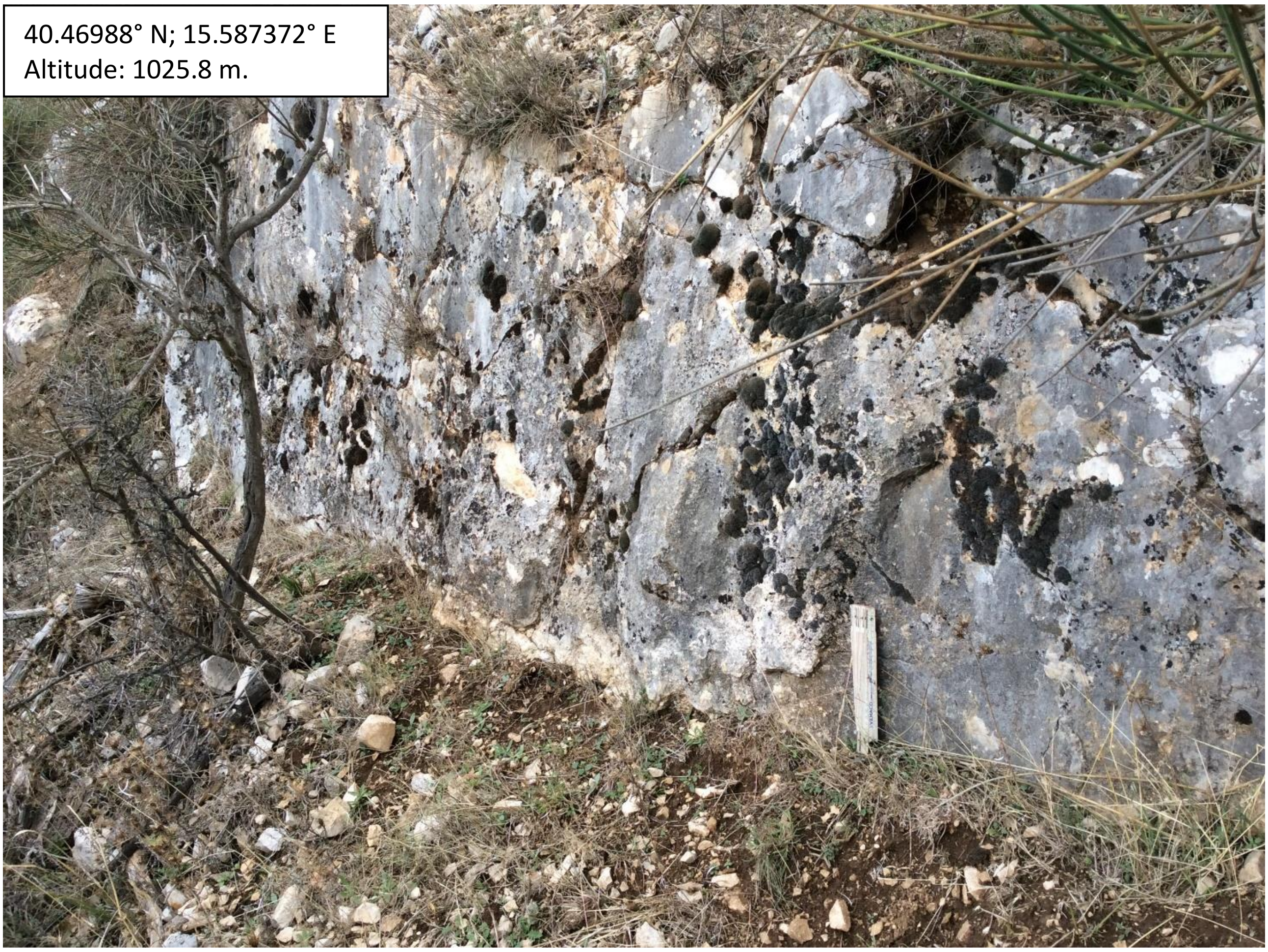

Figure 107.

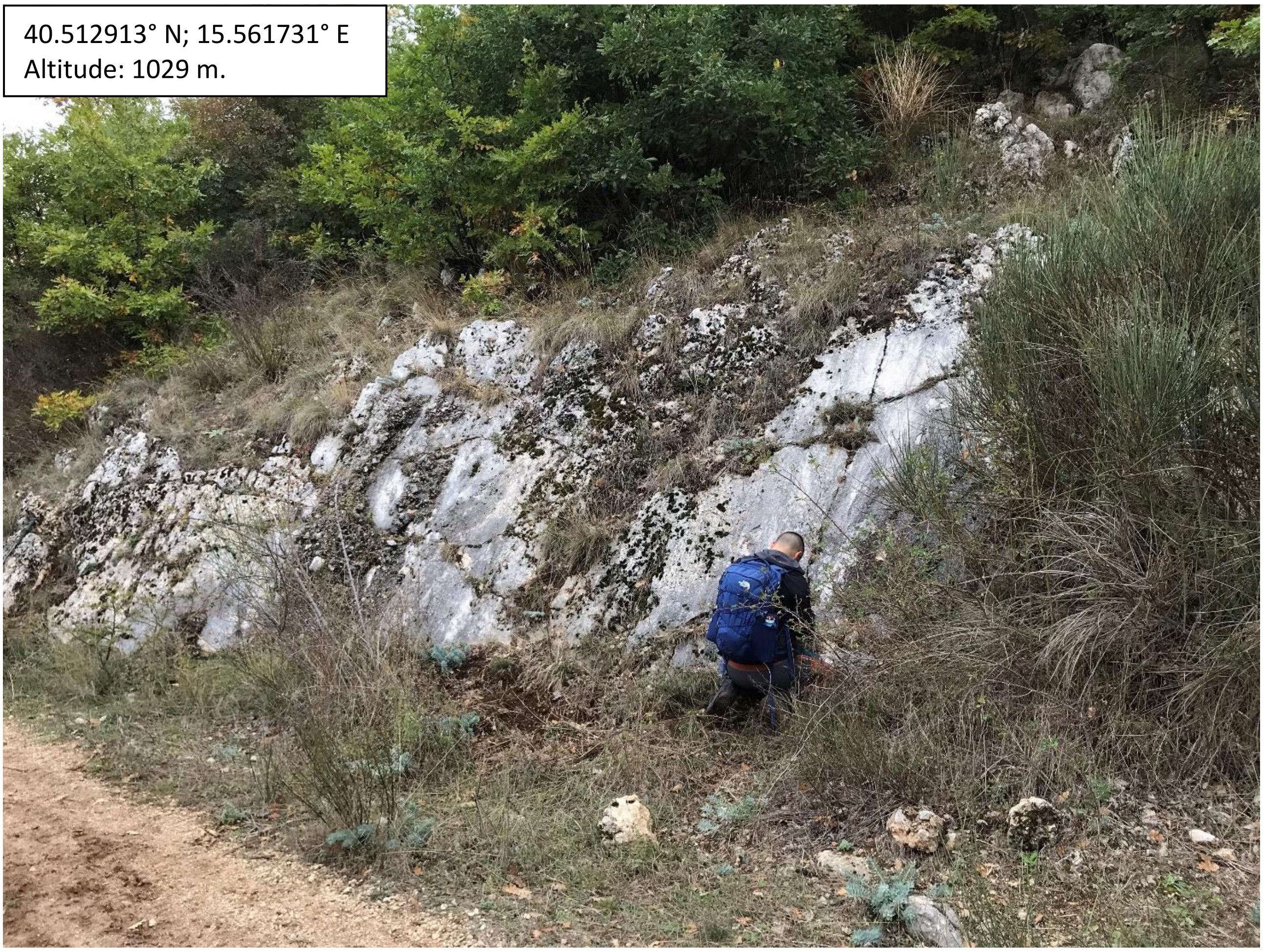

Figure 108.

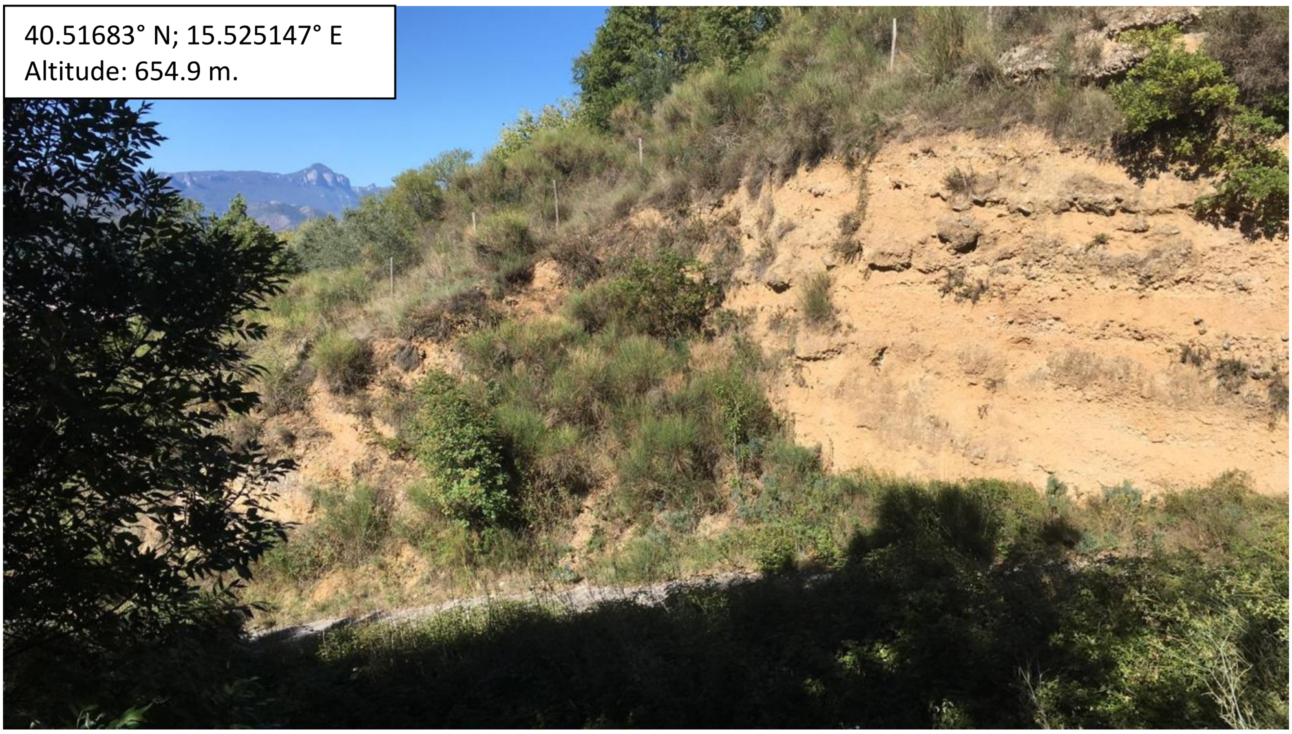

Figure 109.

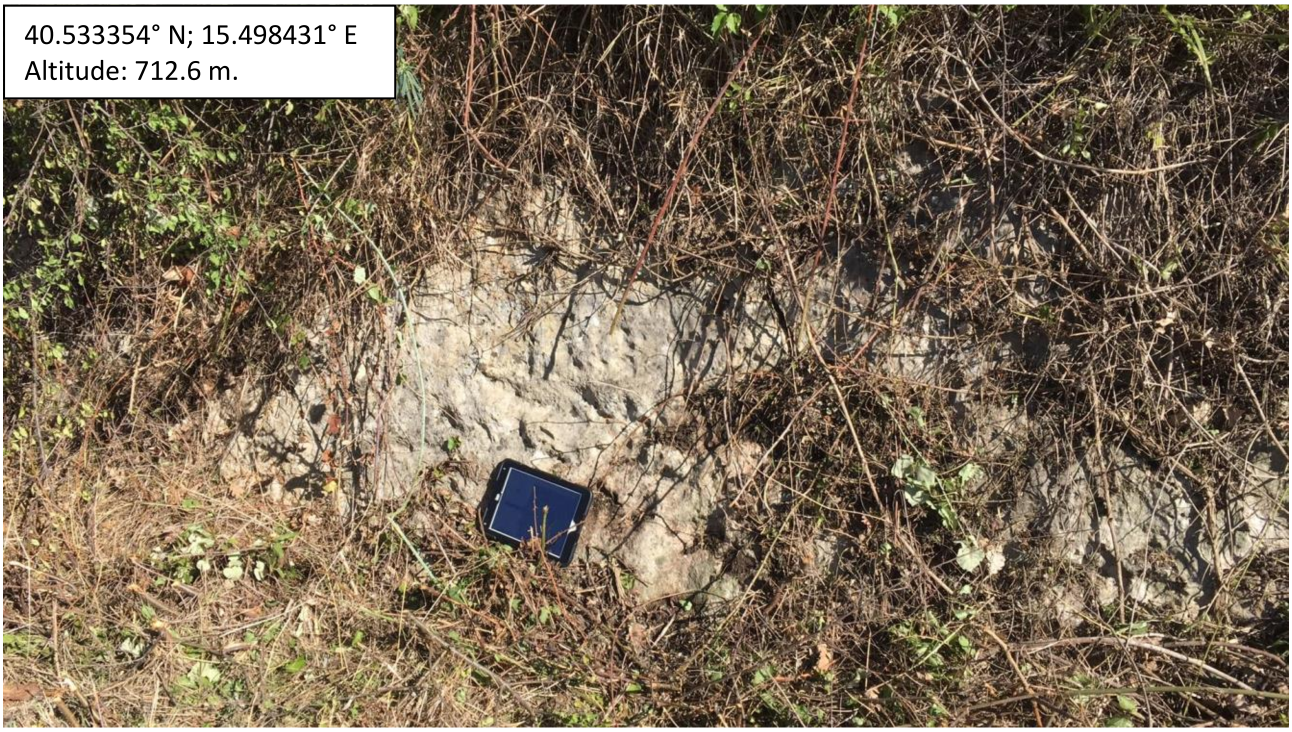

Figure 110.

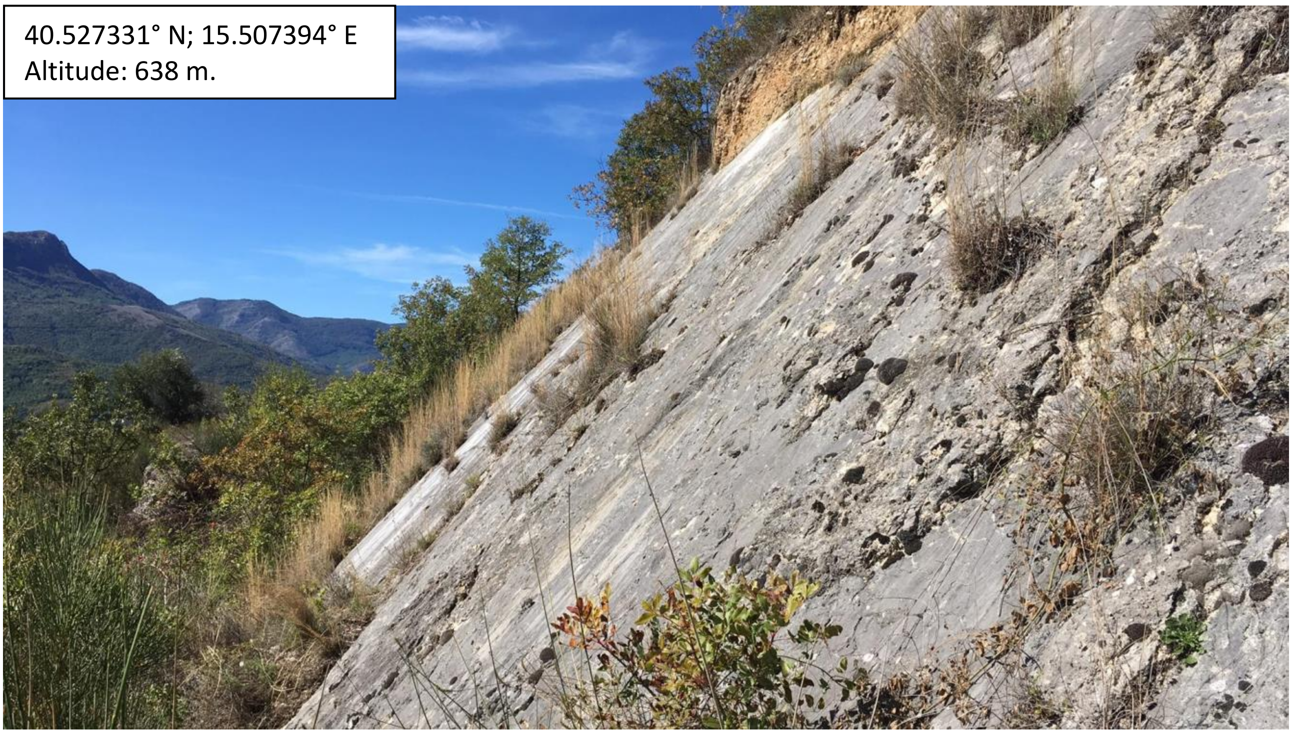

Figure 111.

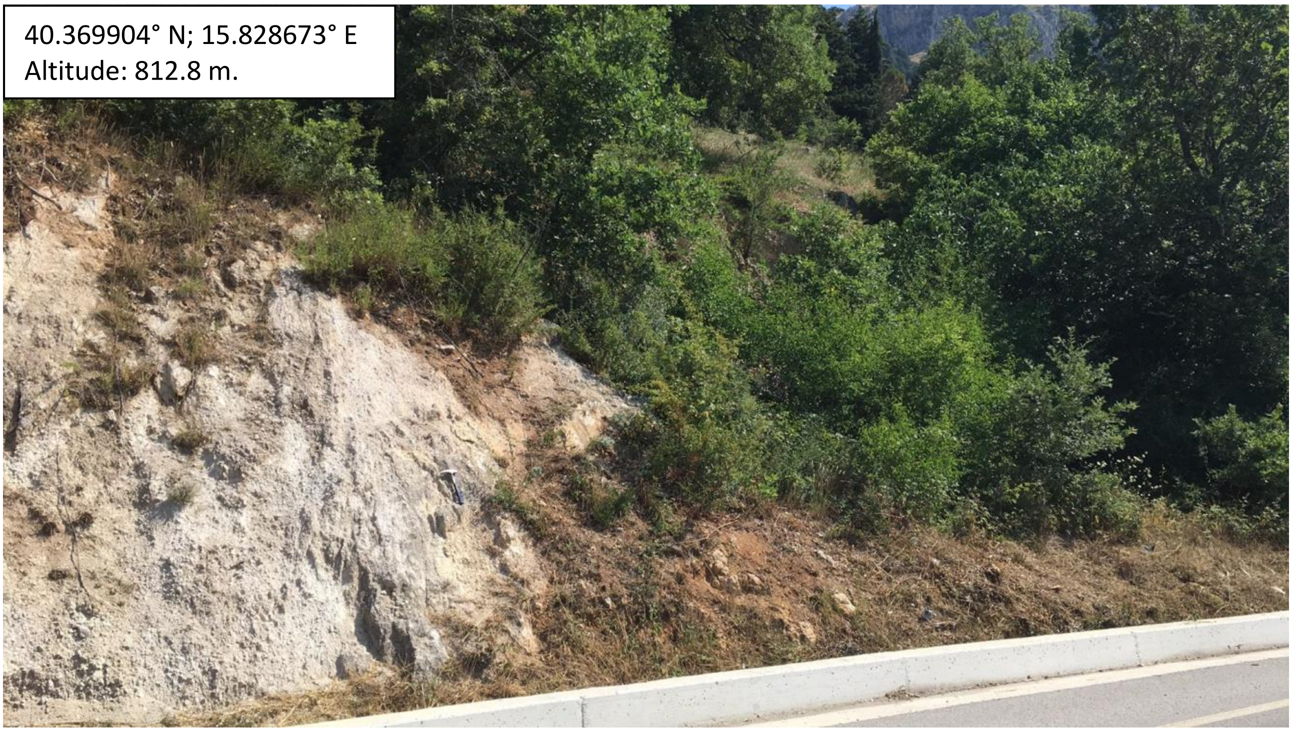

Figure 112.

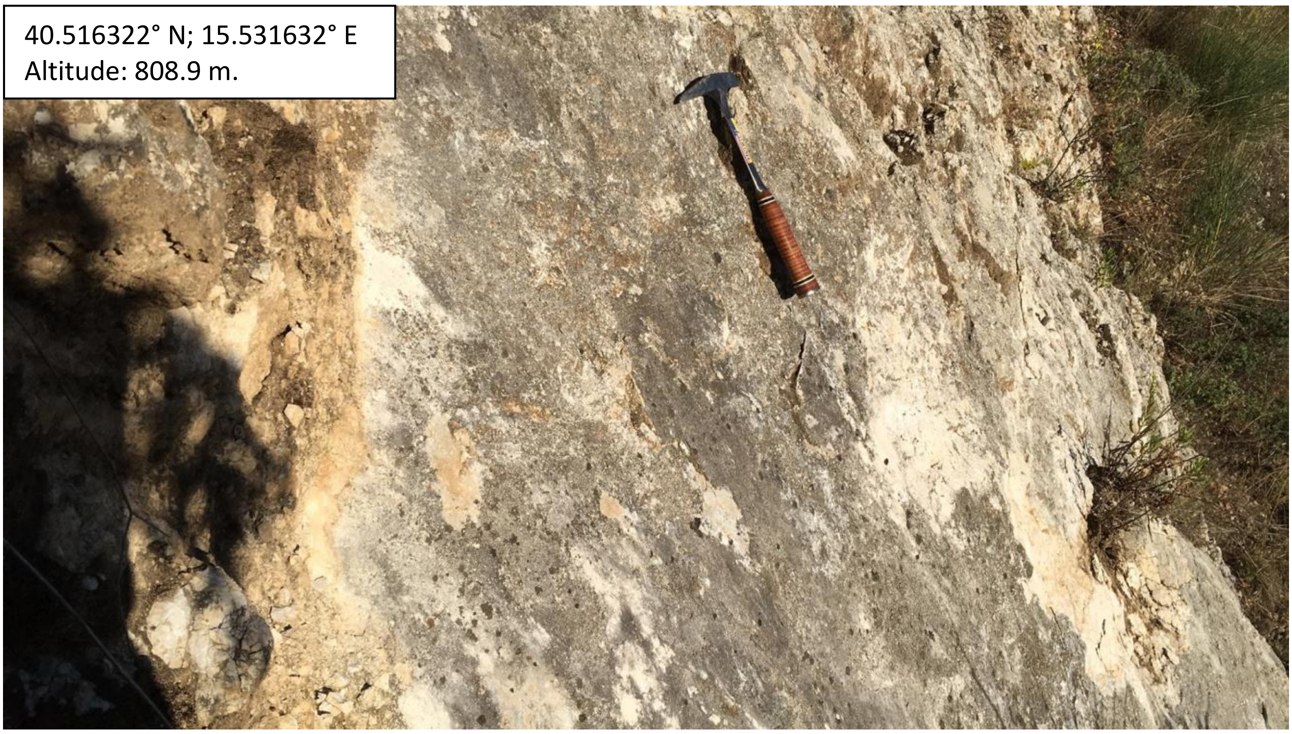

Figure 113.

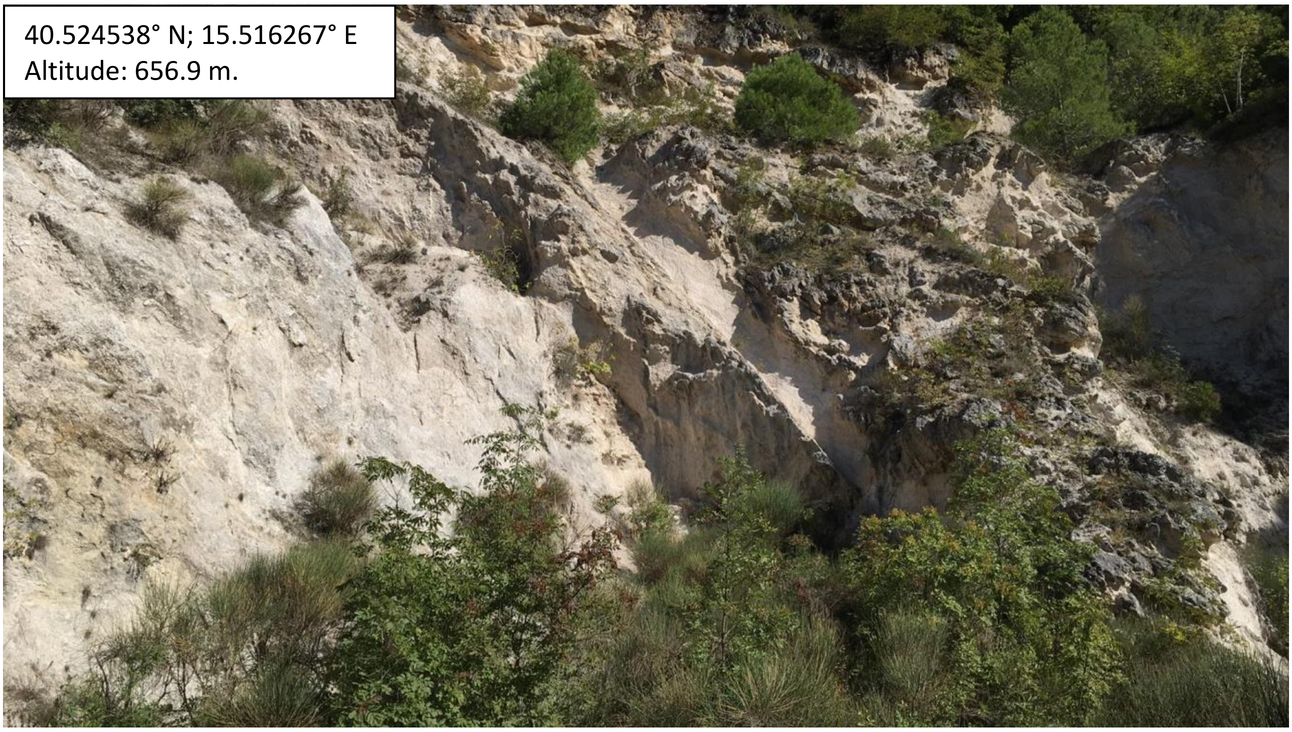

Figure 114.

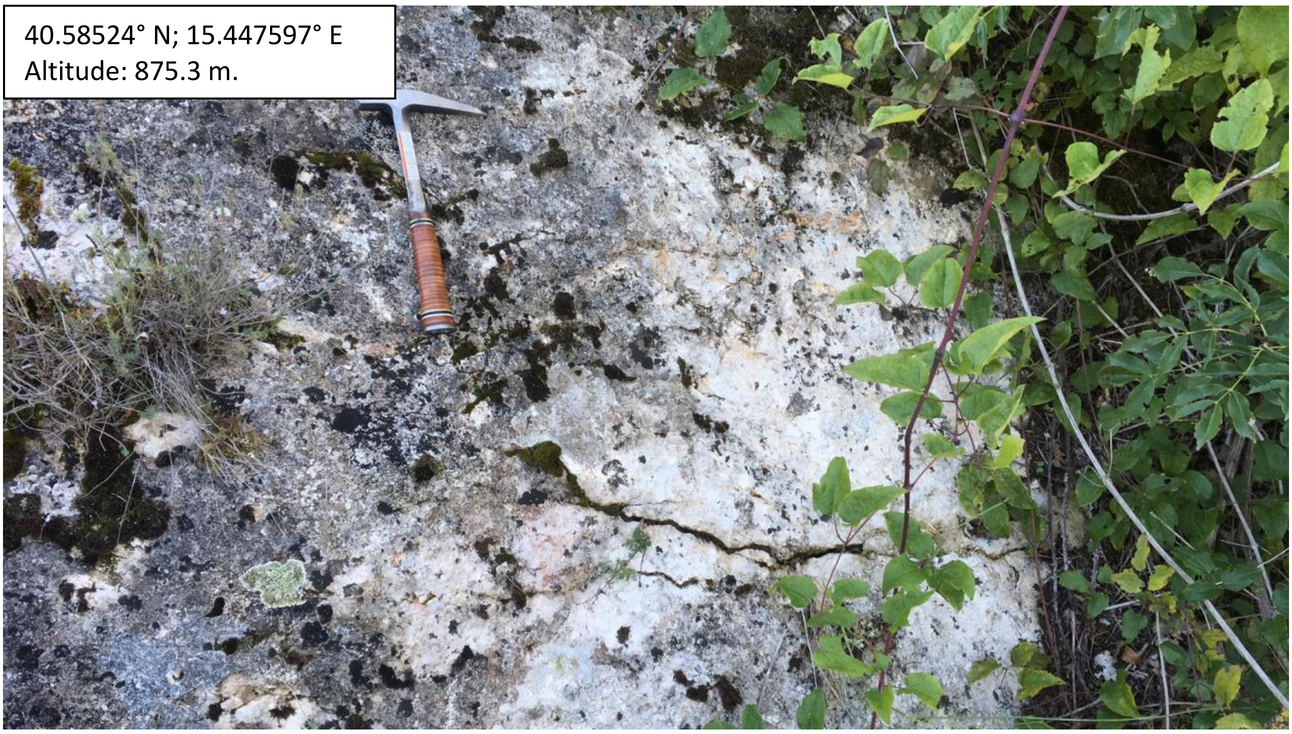

Figure 115.

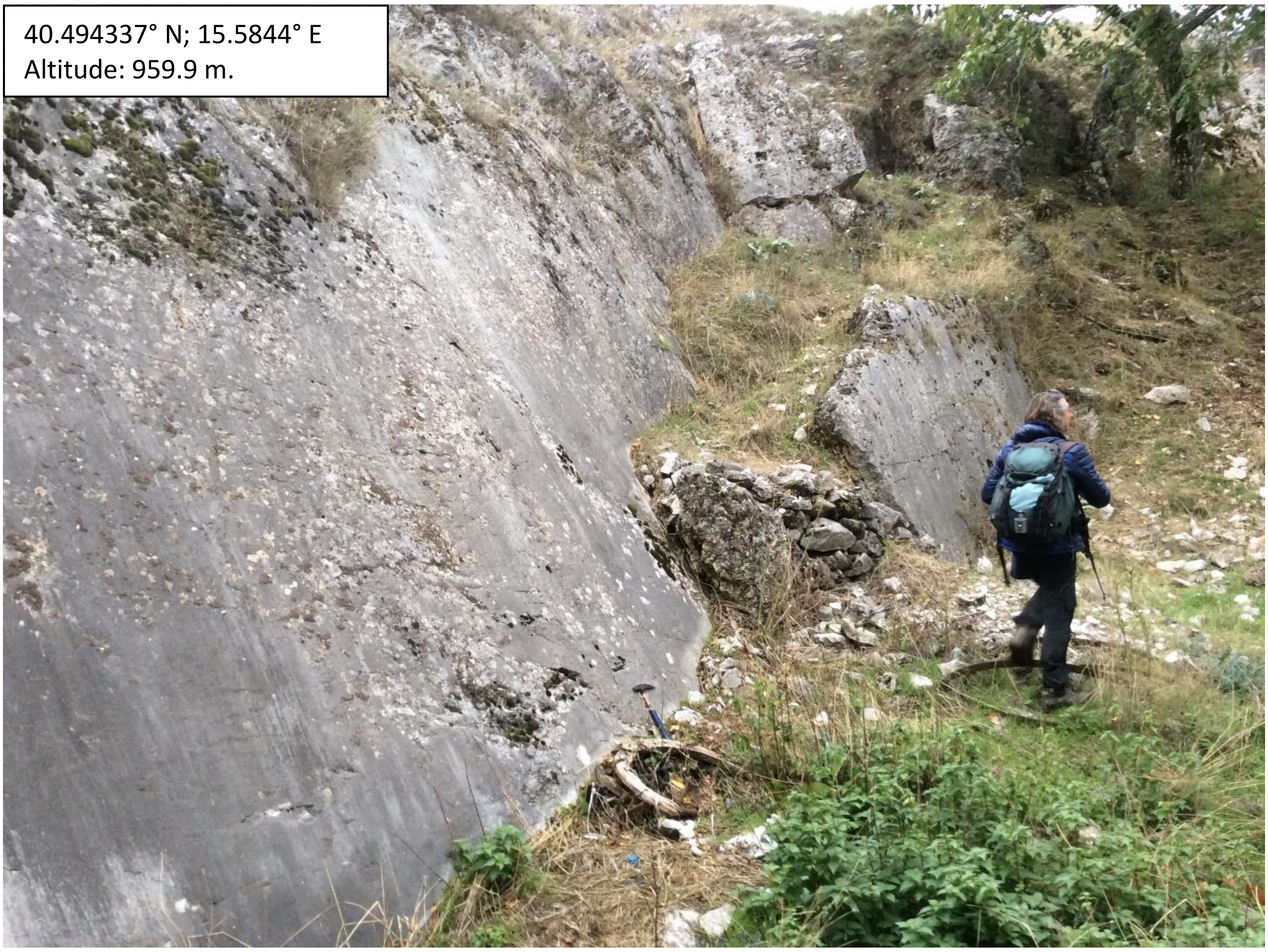

Figure 116.

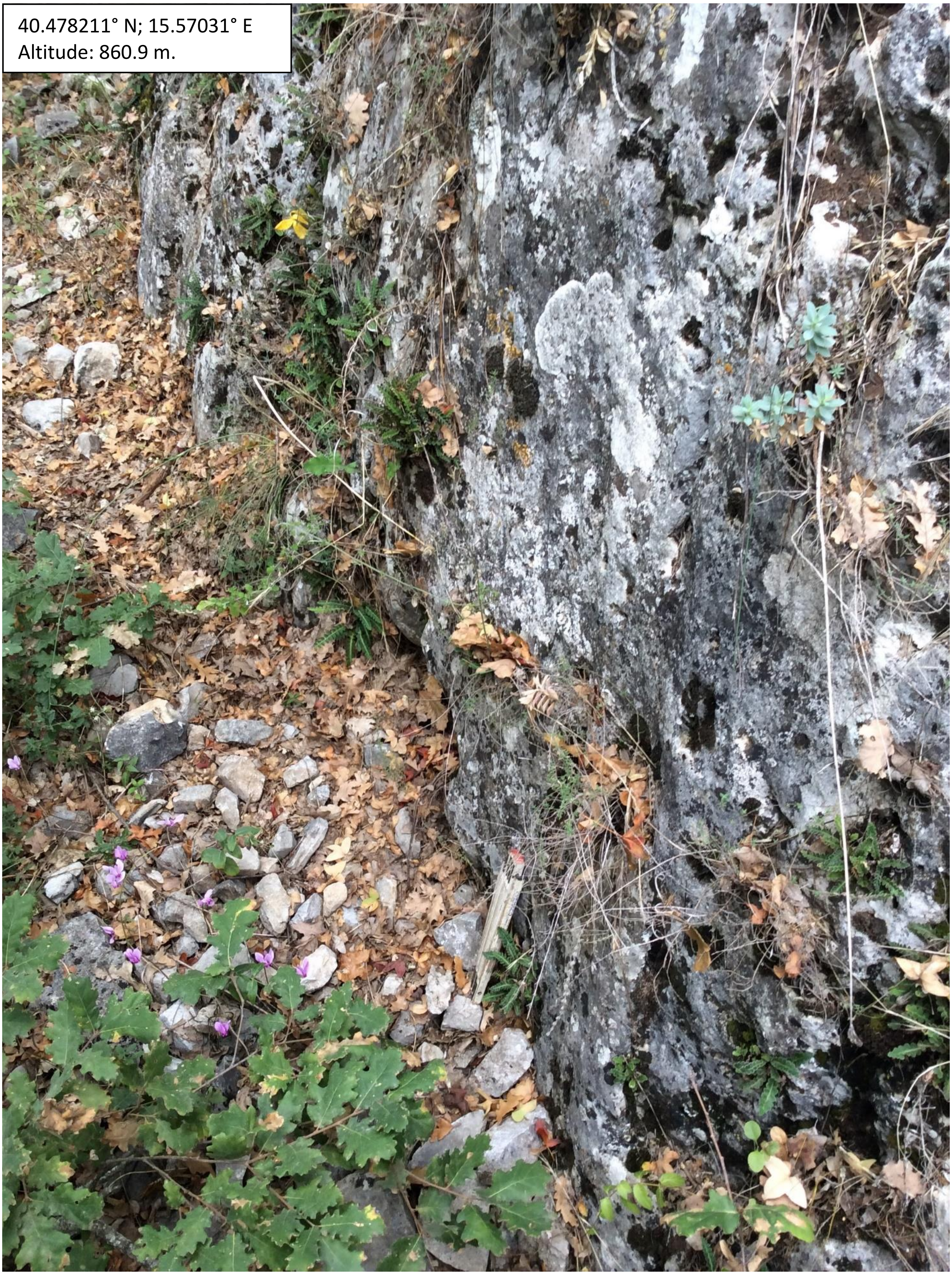

Figure 117.

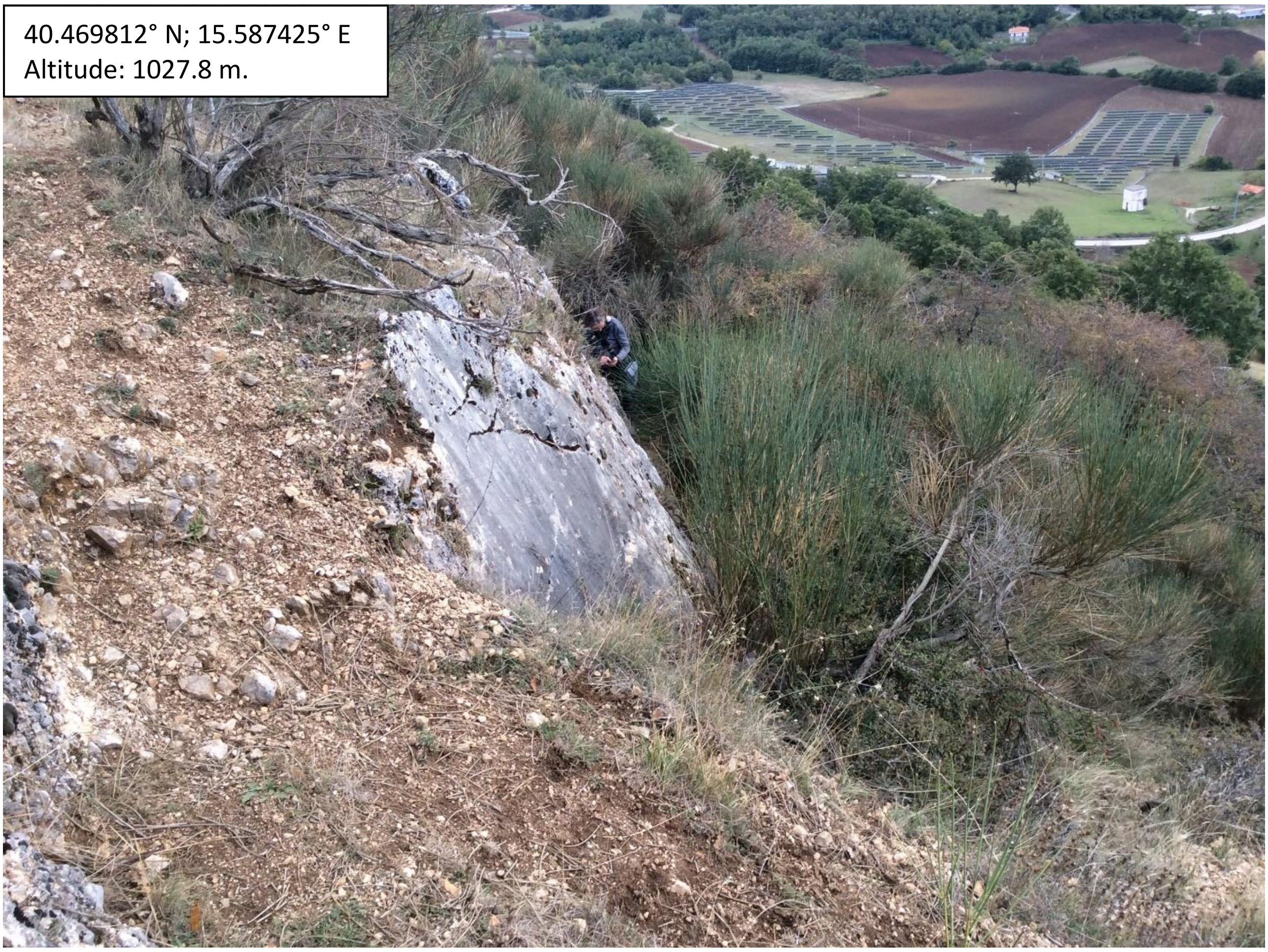

Figure 118.

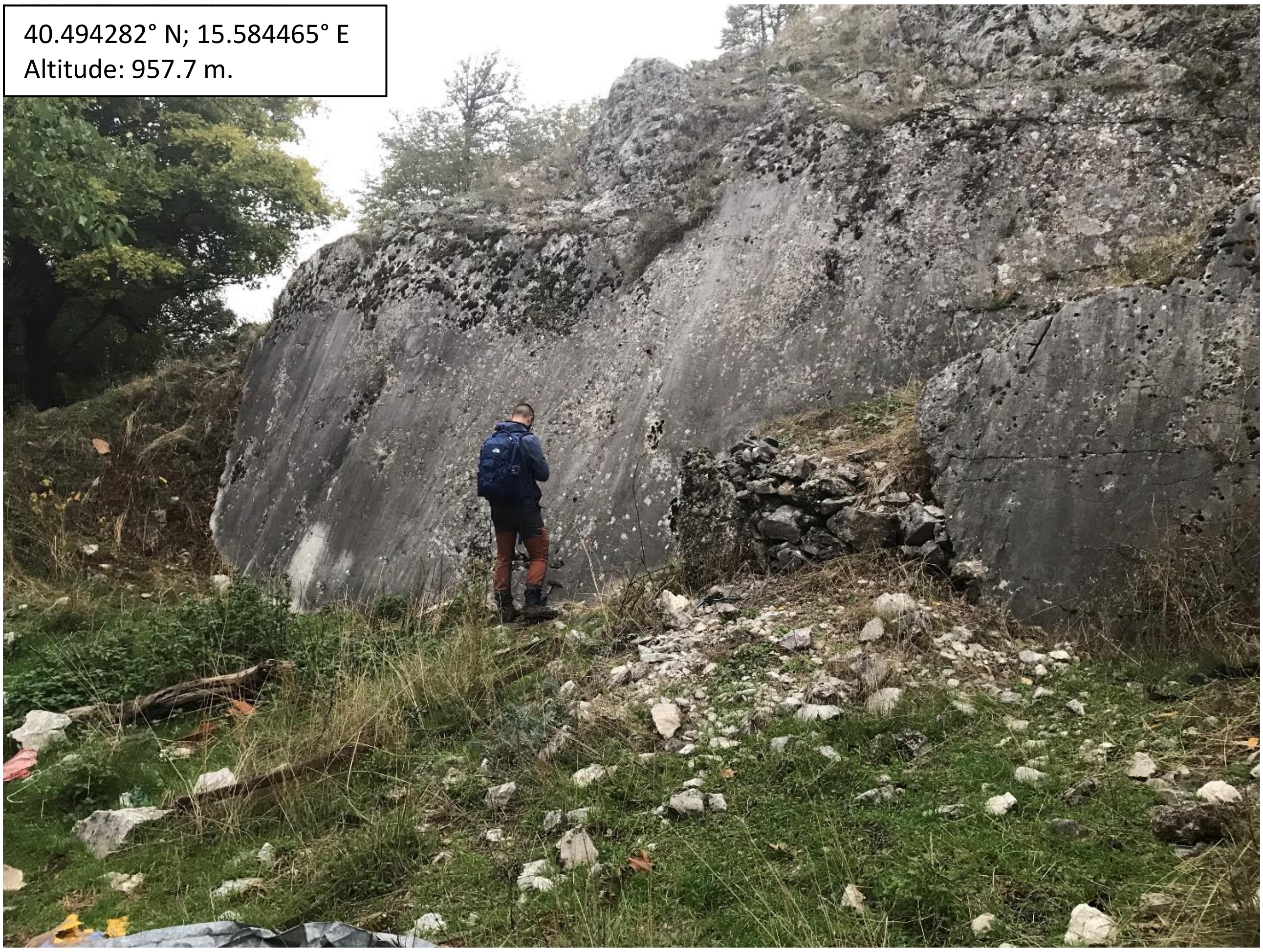

Figure 119.

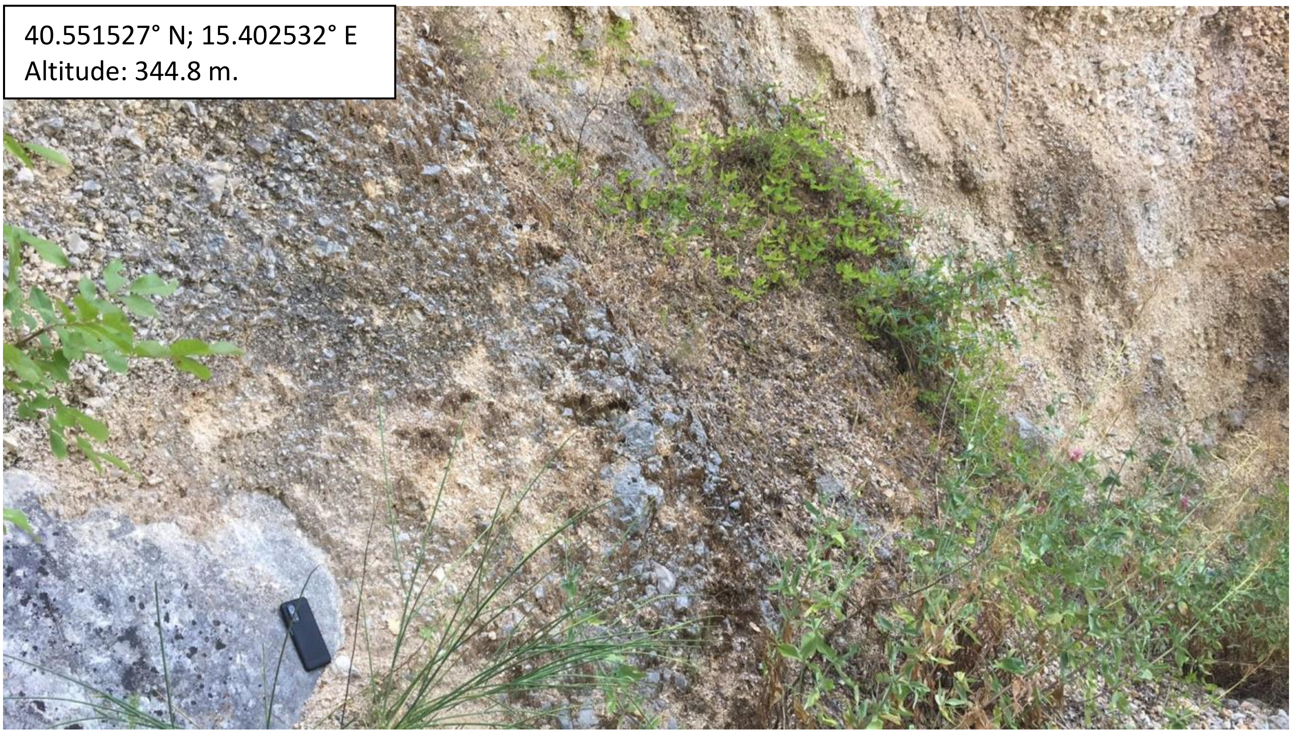

Figure 120.

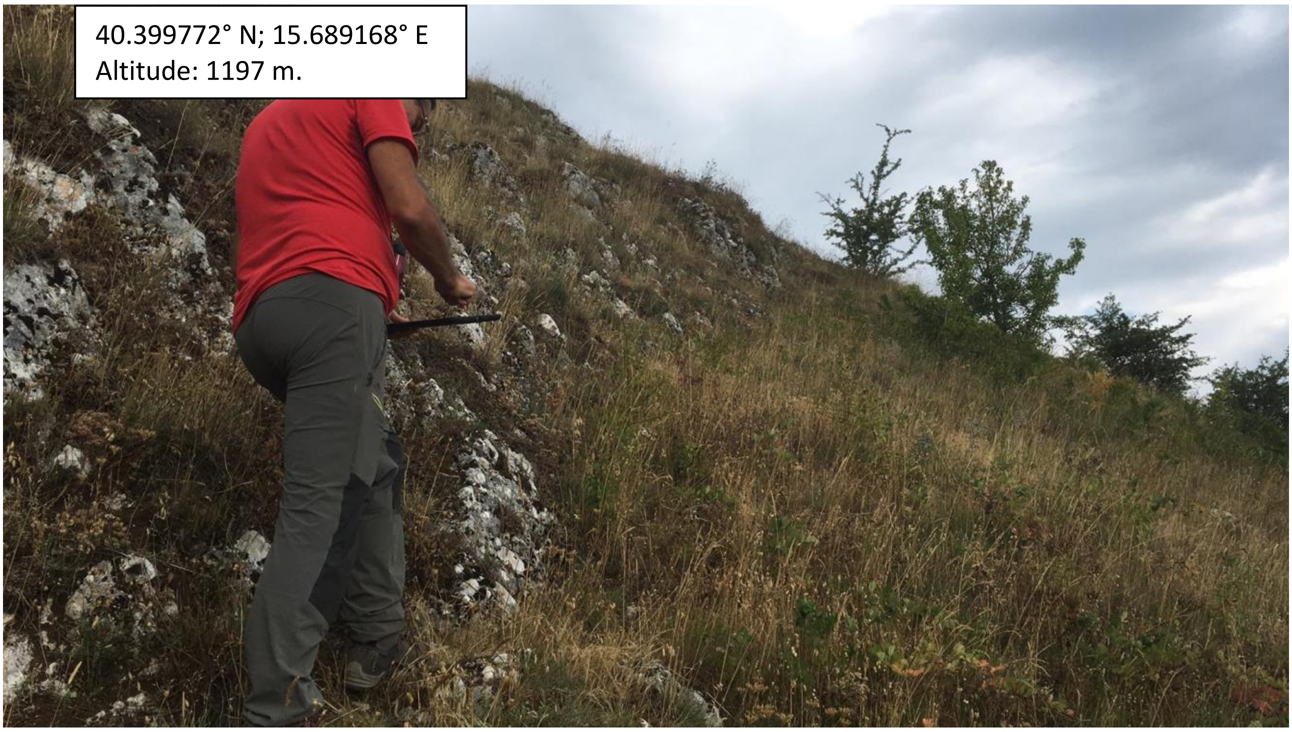

Figure 121.

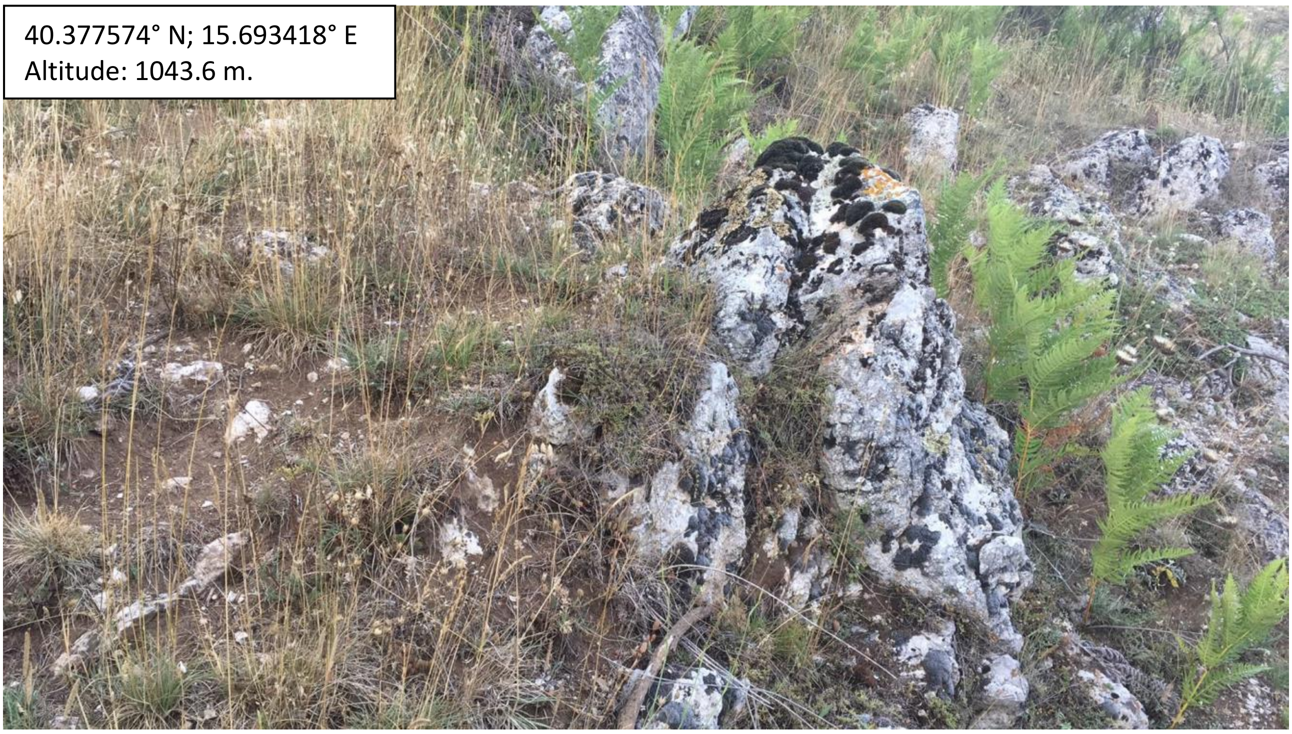

Figure 122.

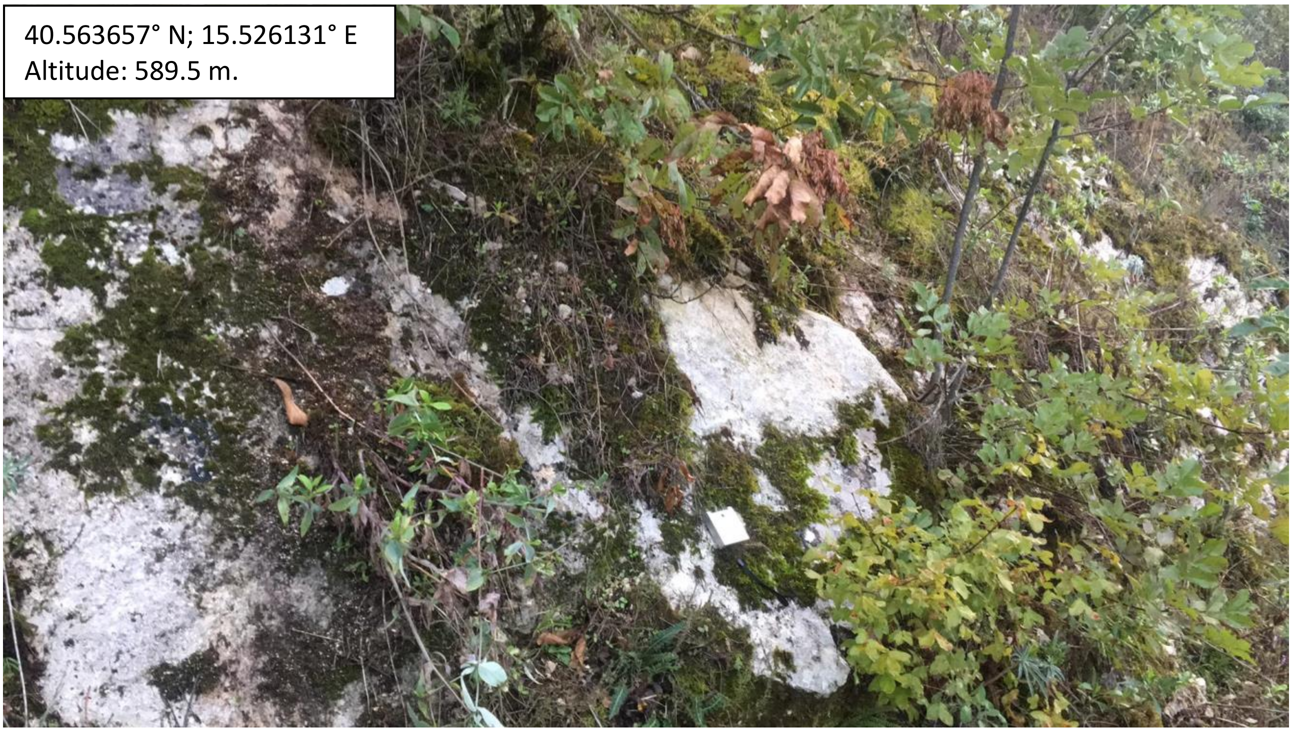

Figure 123.

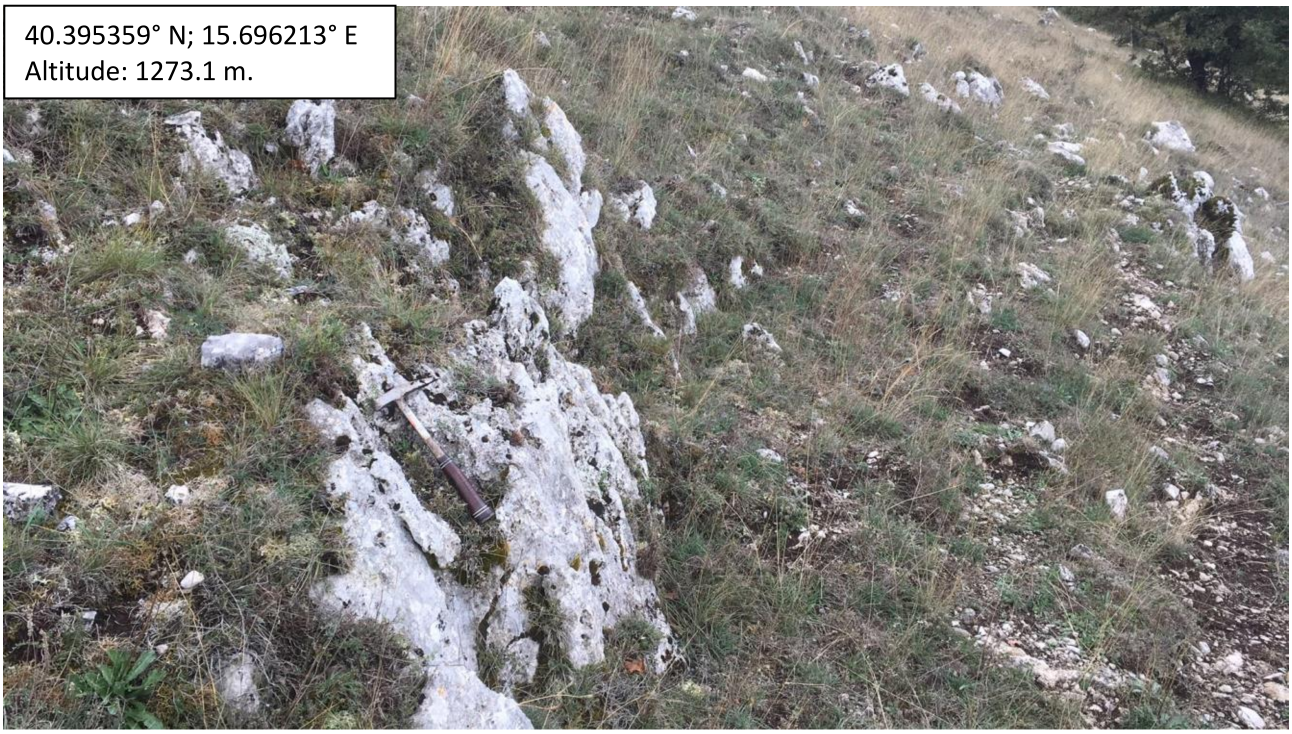

Figure 124.

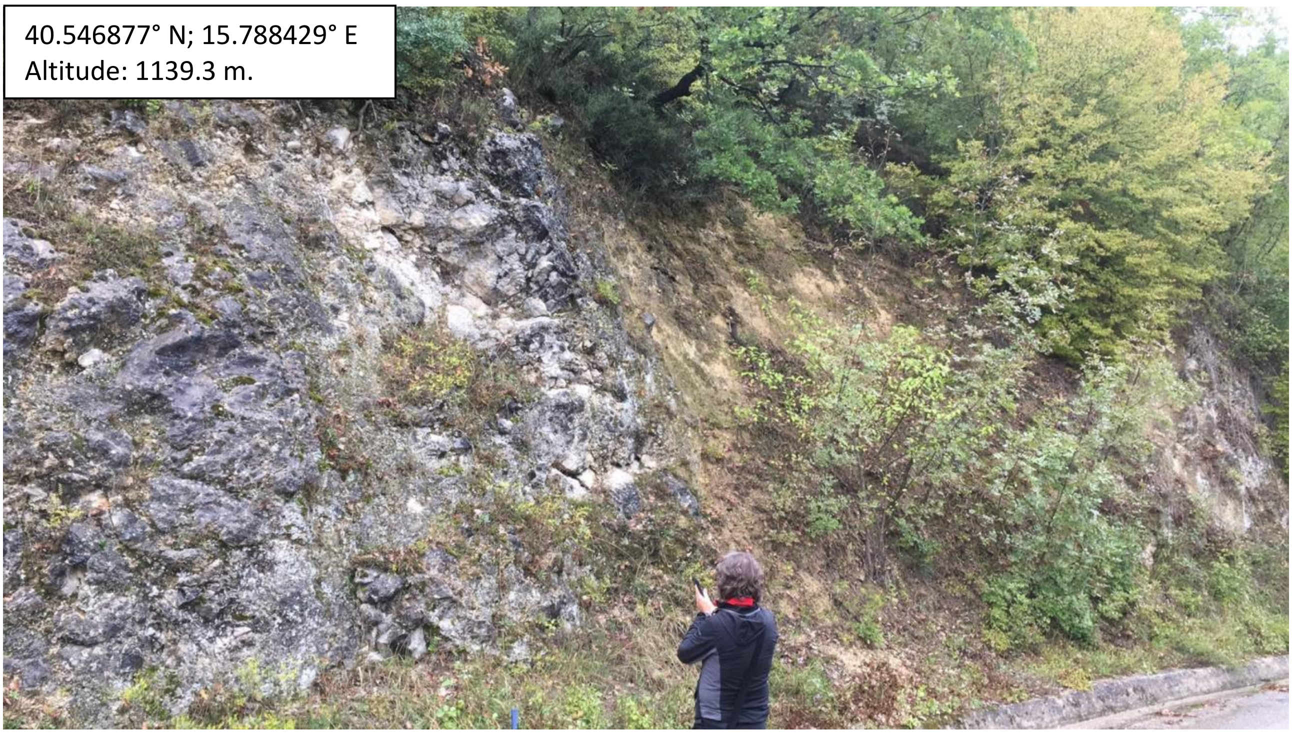

Figure 125

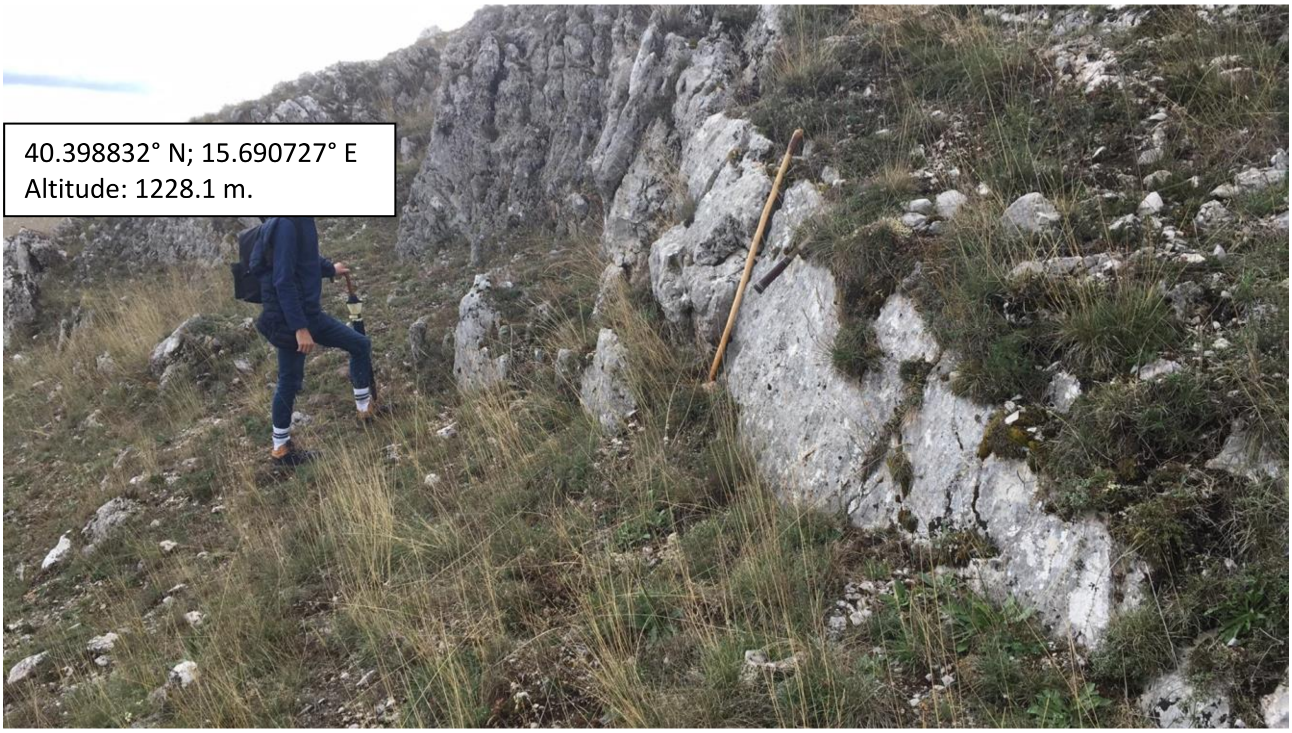

Figure 126.

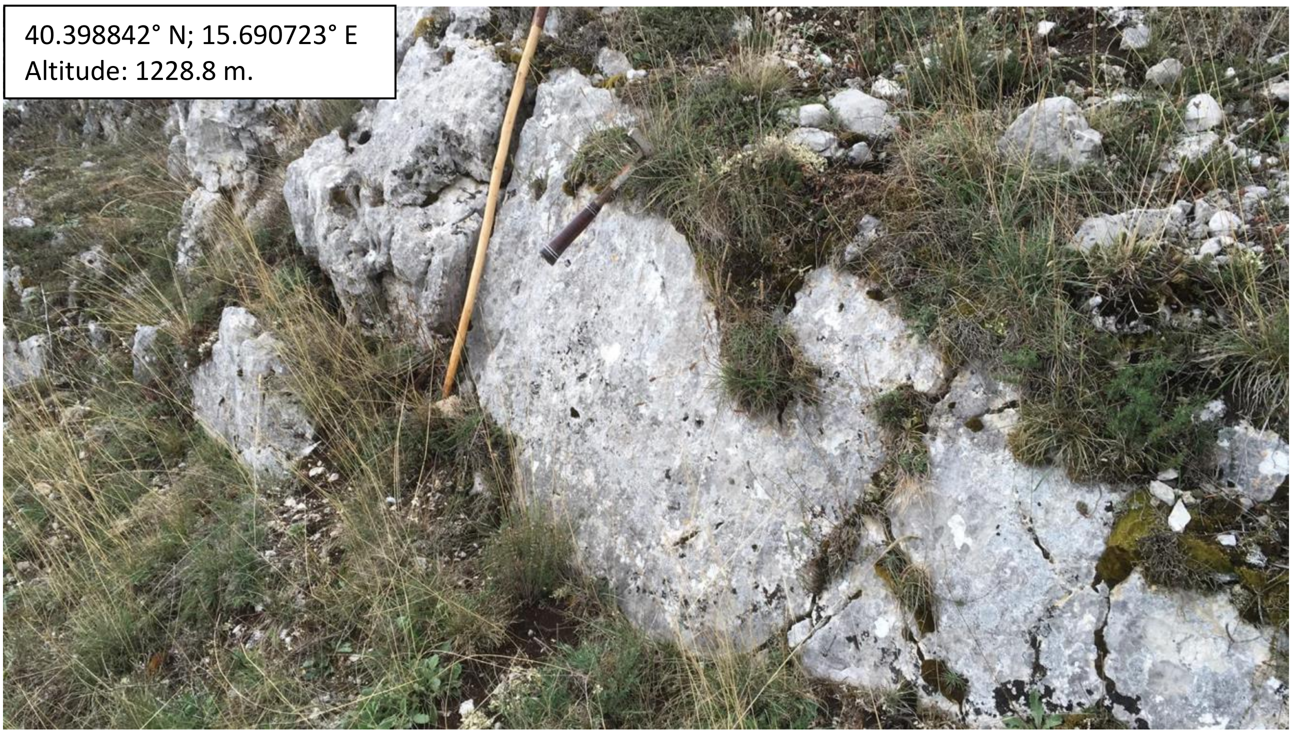

Figure 127.

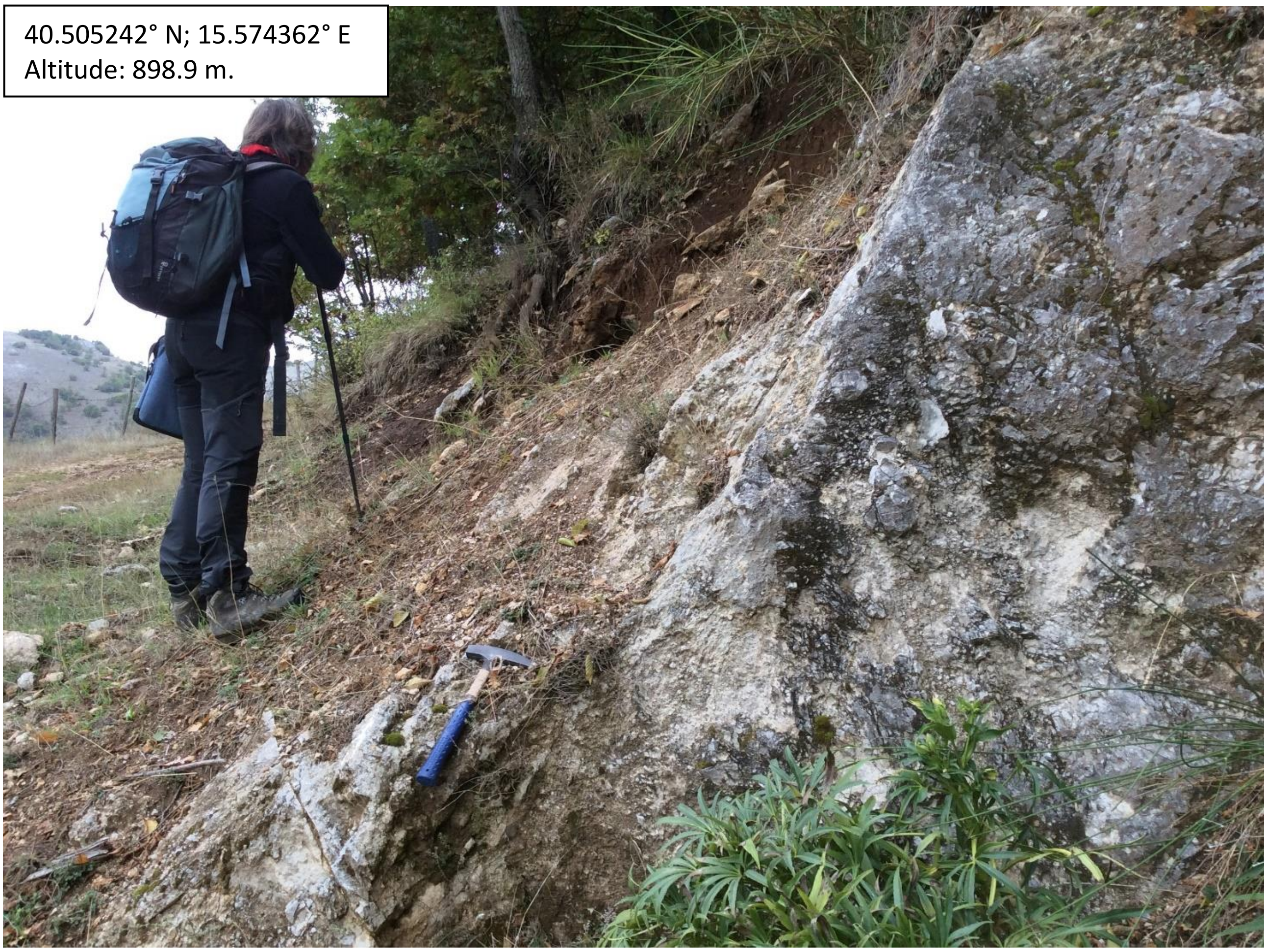

Figure 128.

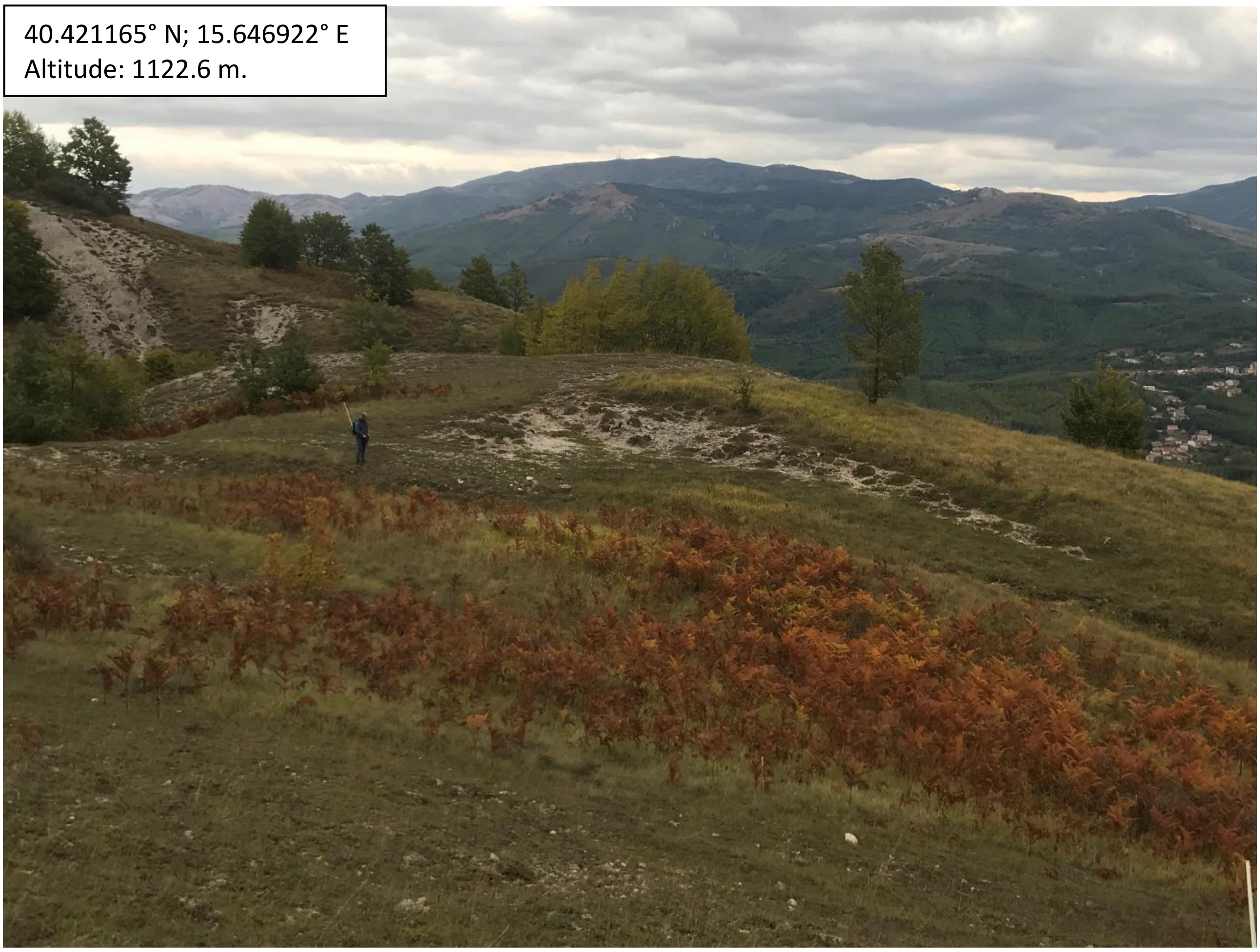

Figure 129.

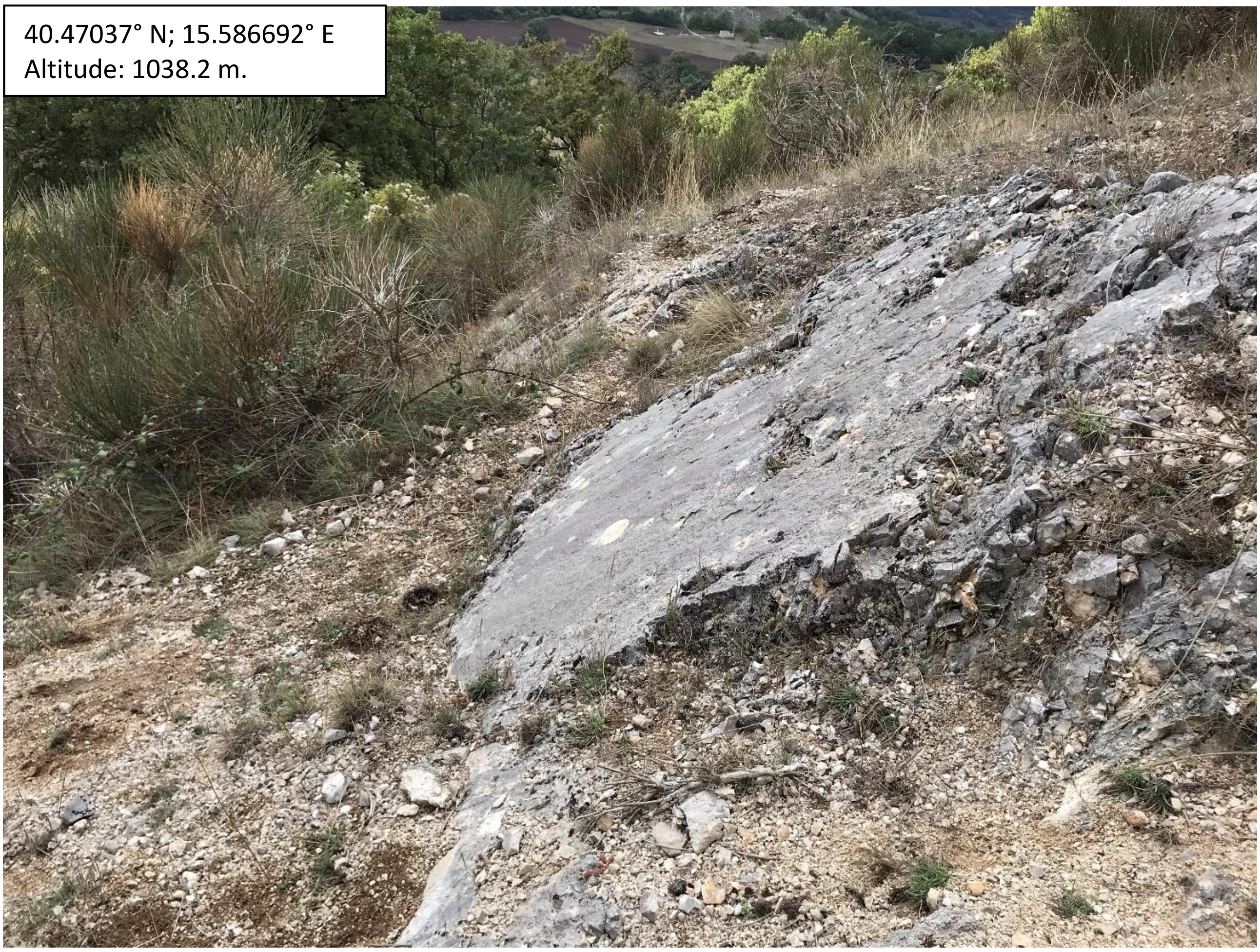

Figure 130.

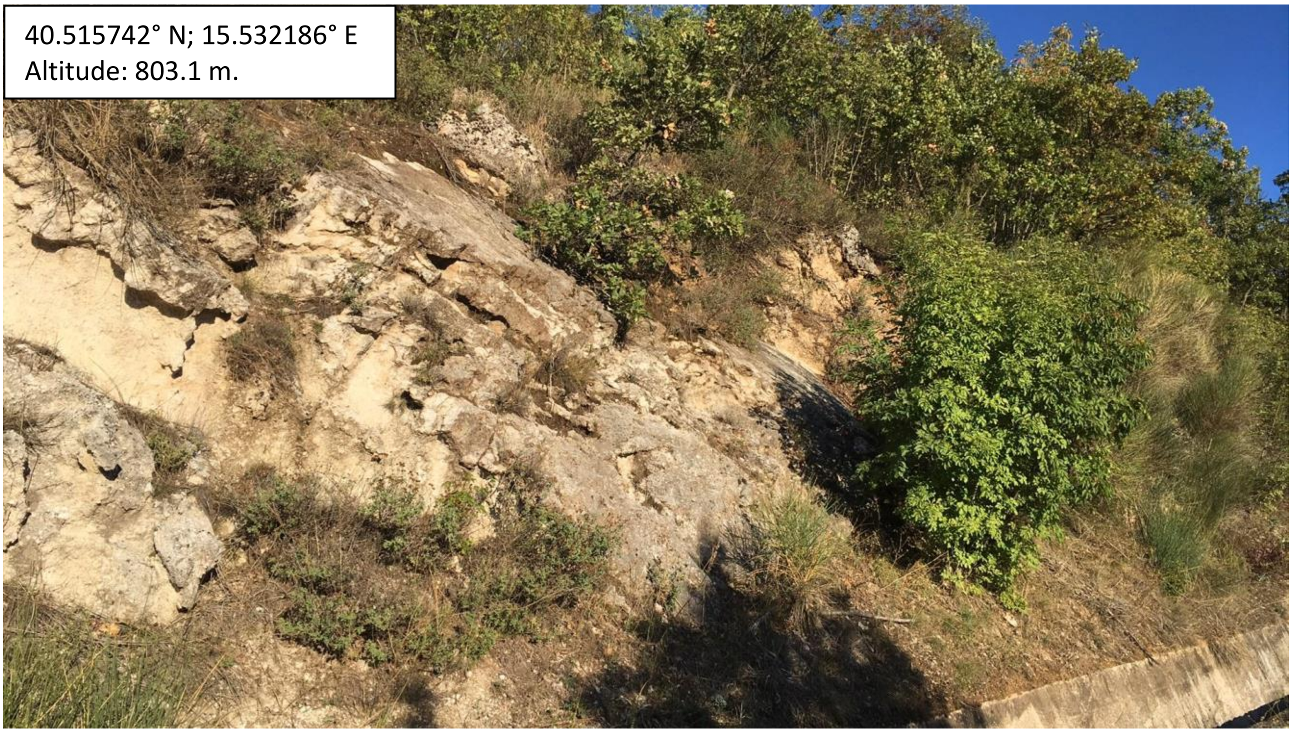

Figure 131.

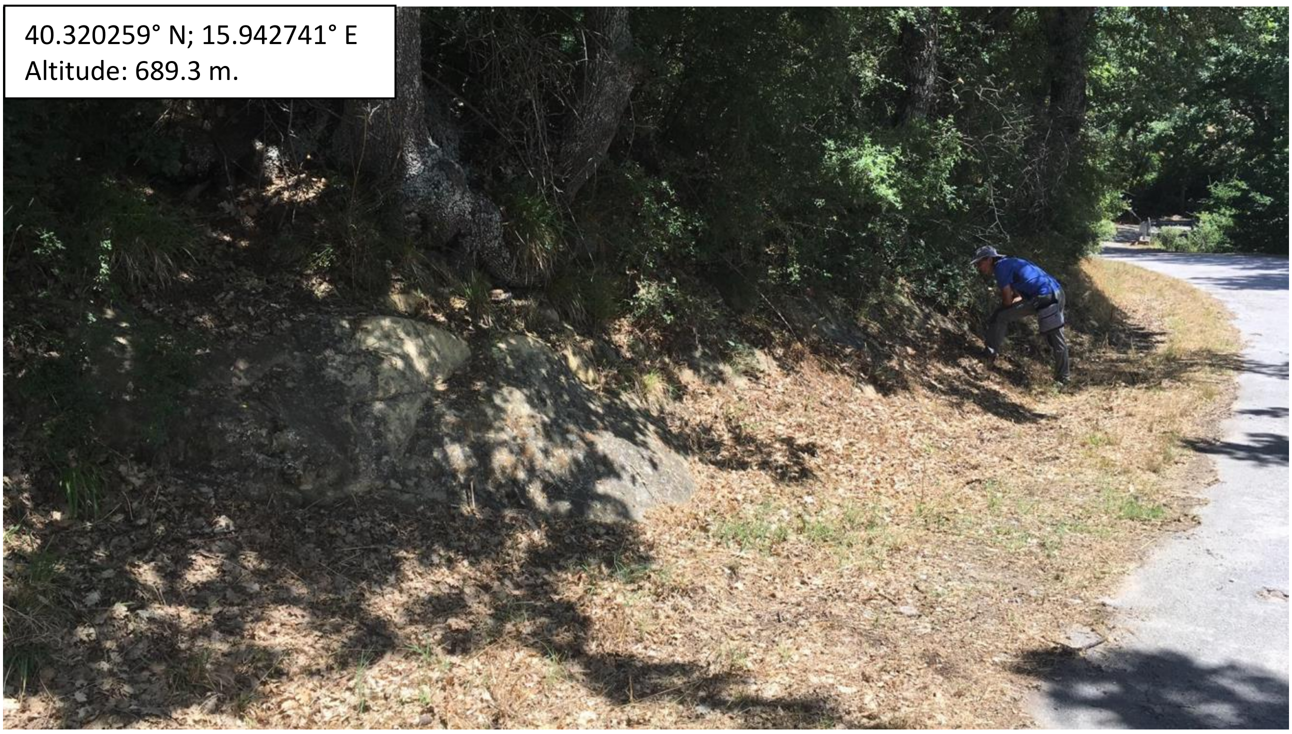

Figure 132.

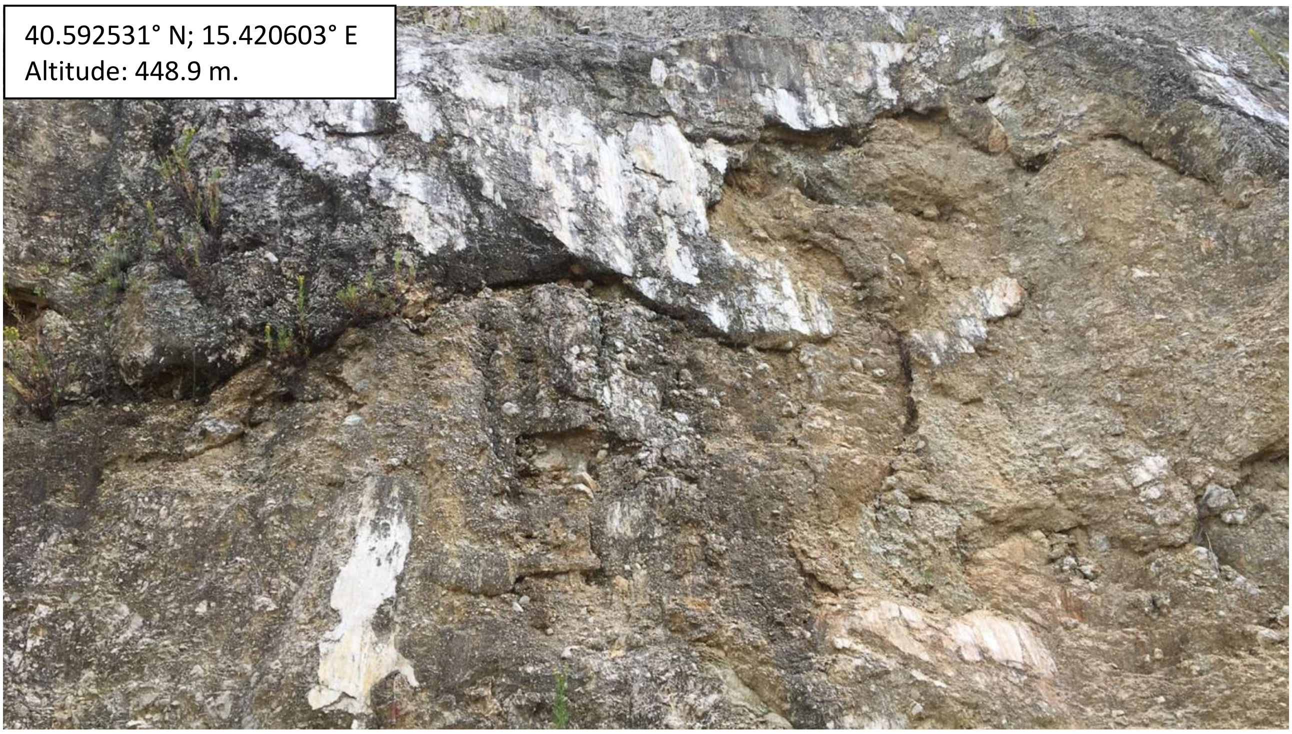

Figure 133.

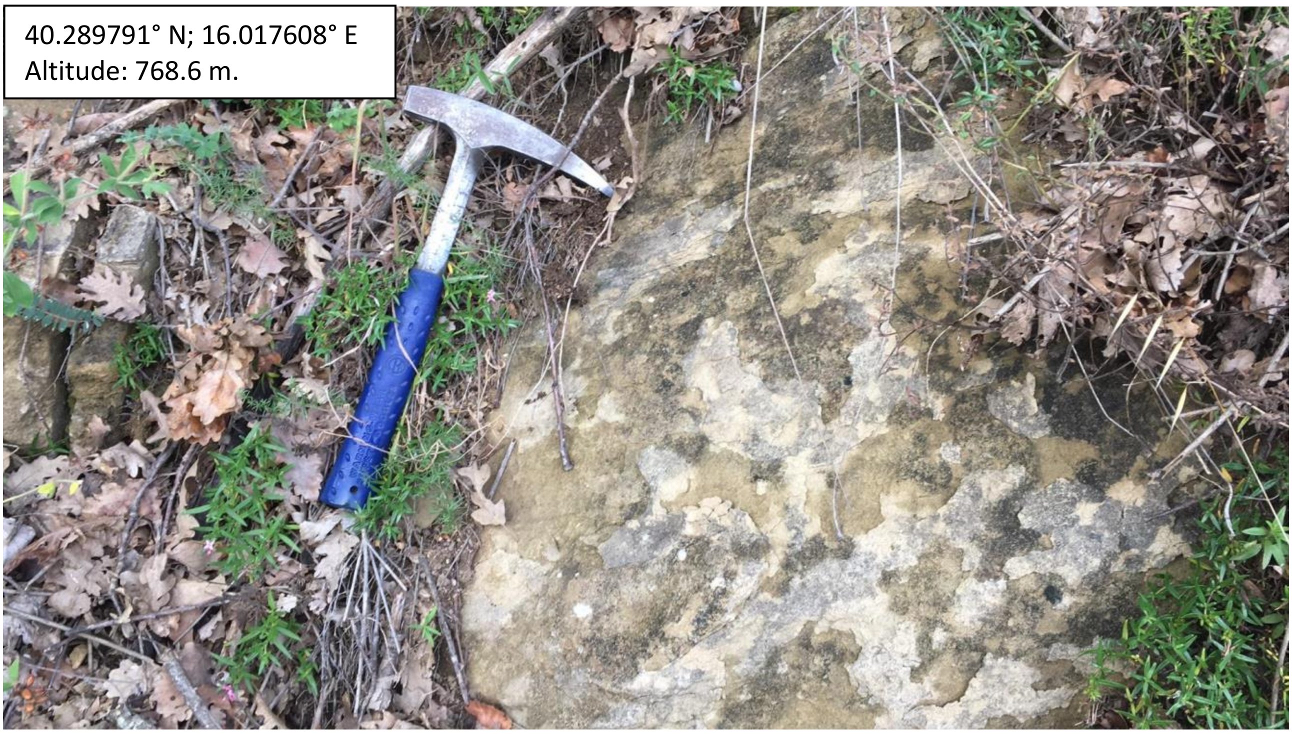

Figure 134.

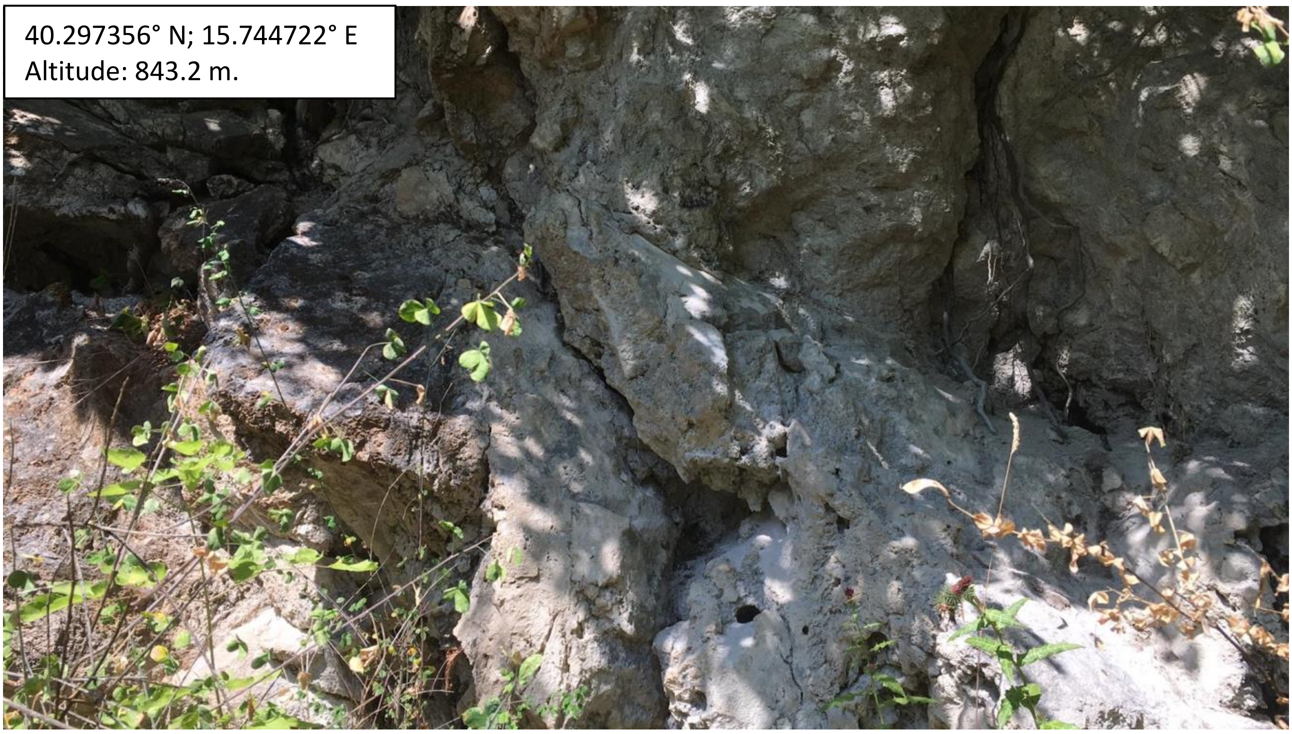

Figure 135.

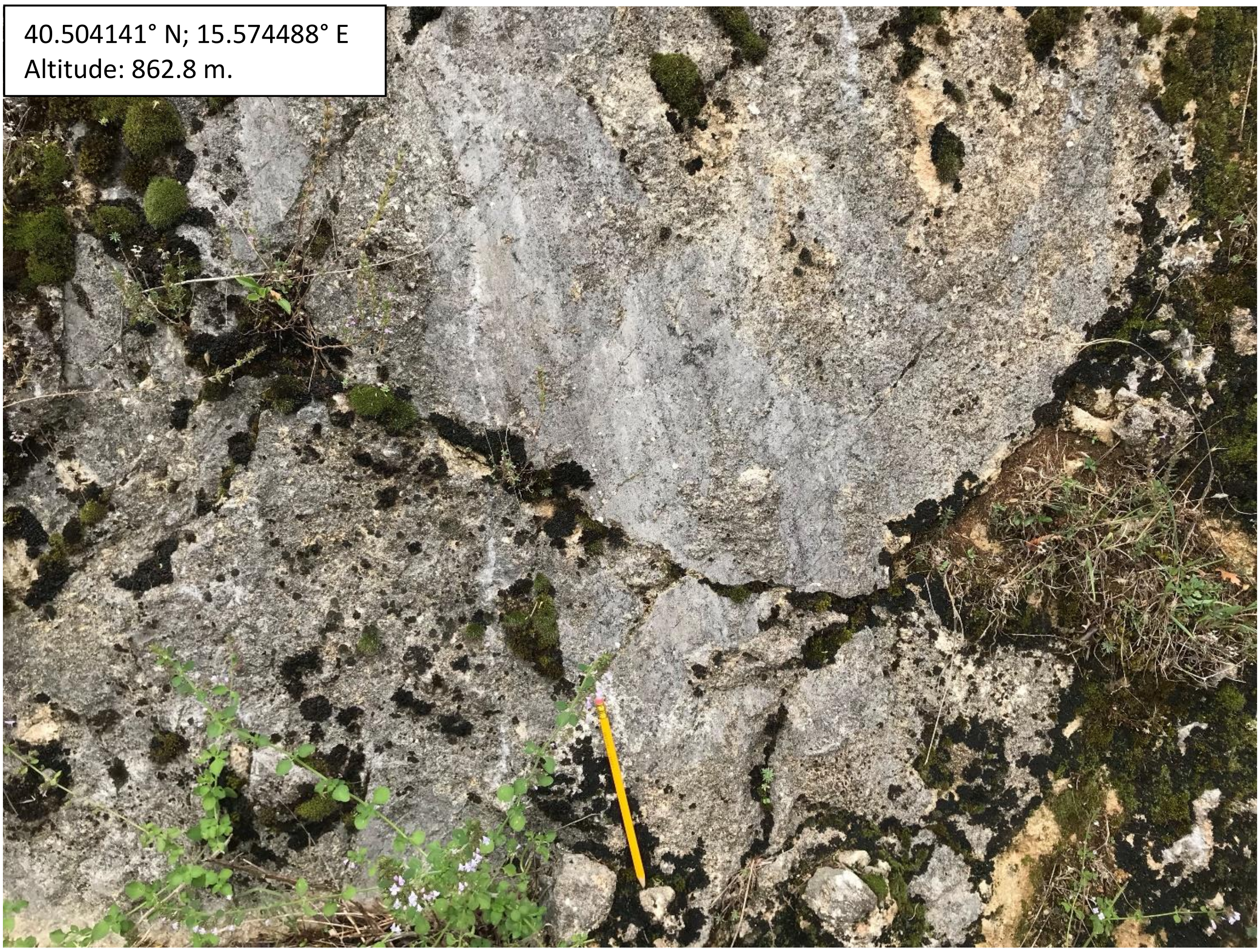

Figure 136.

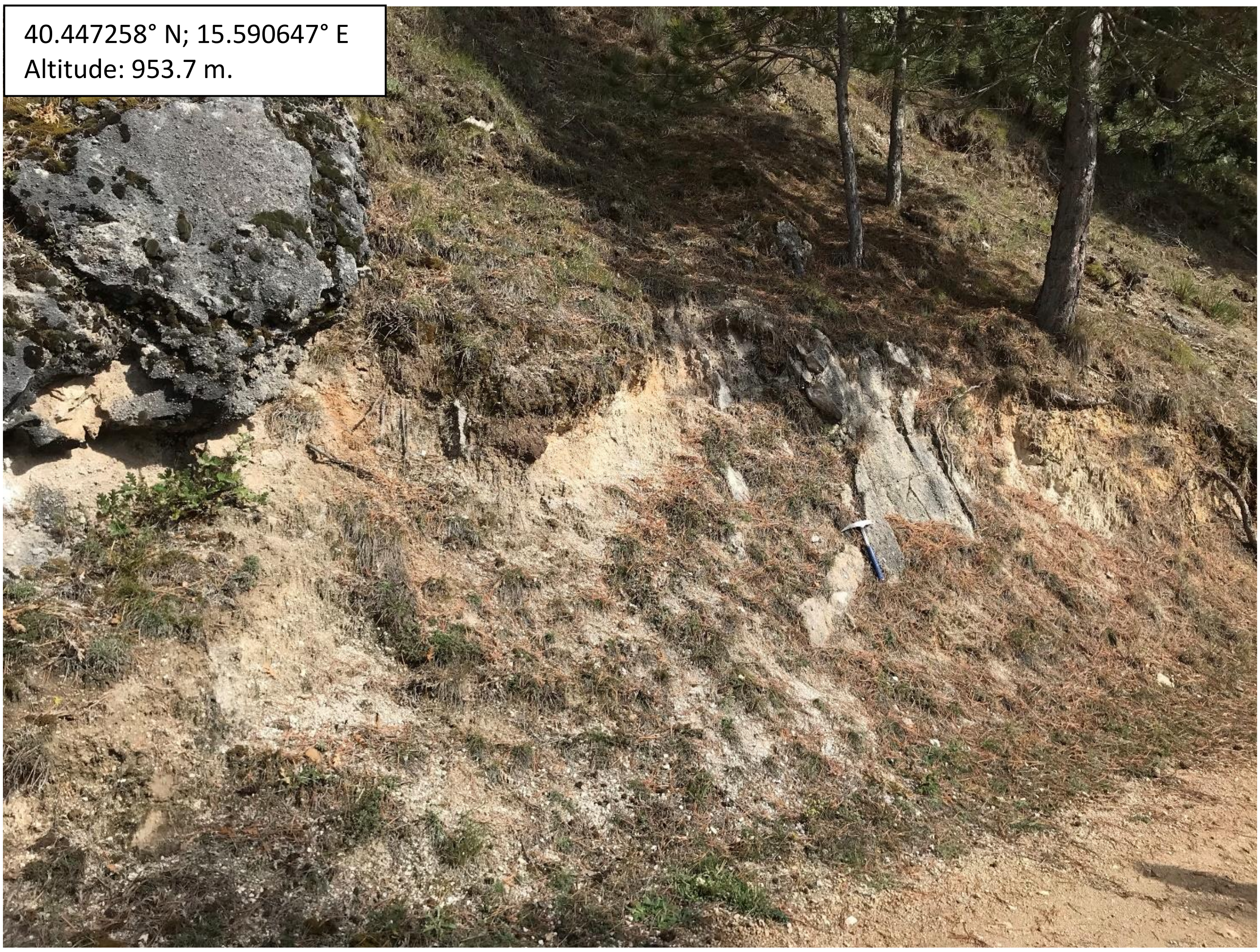

Figure 137.

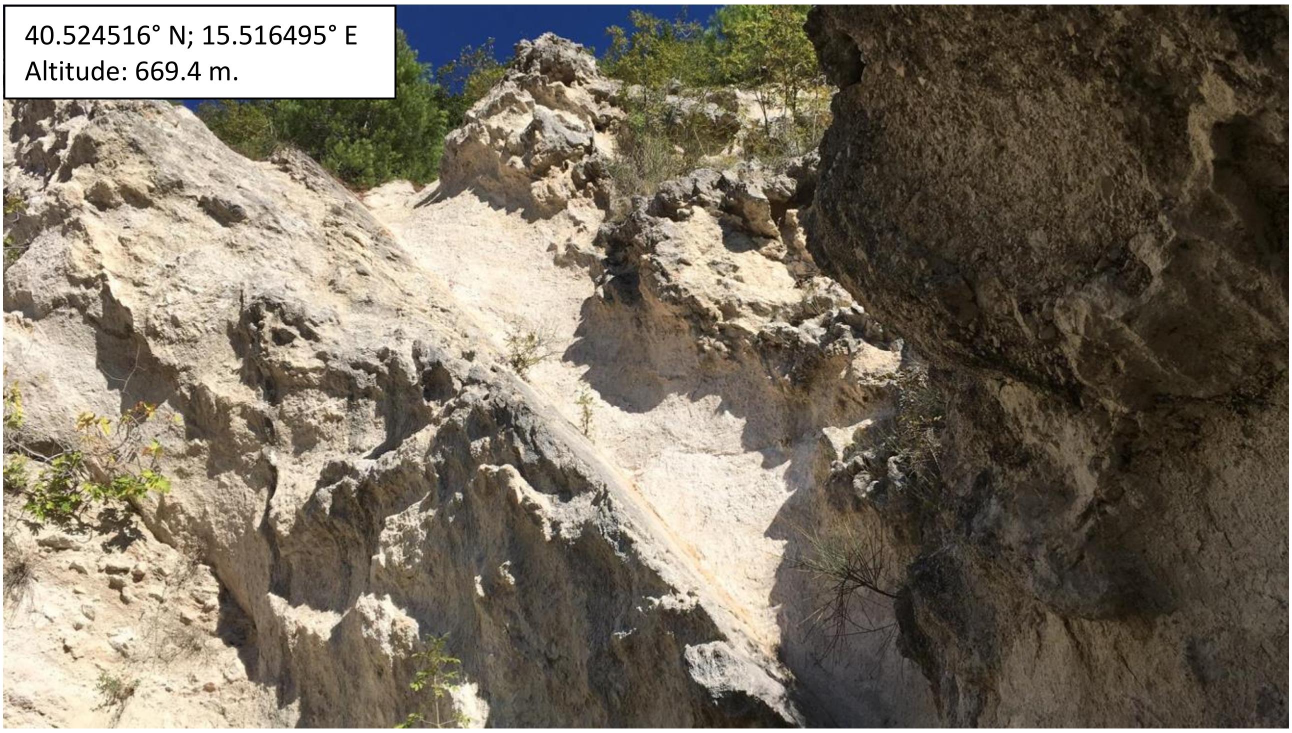

Figure 138.

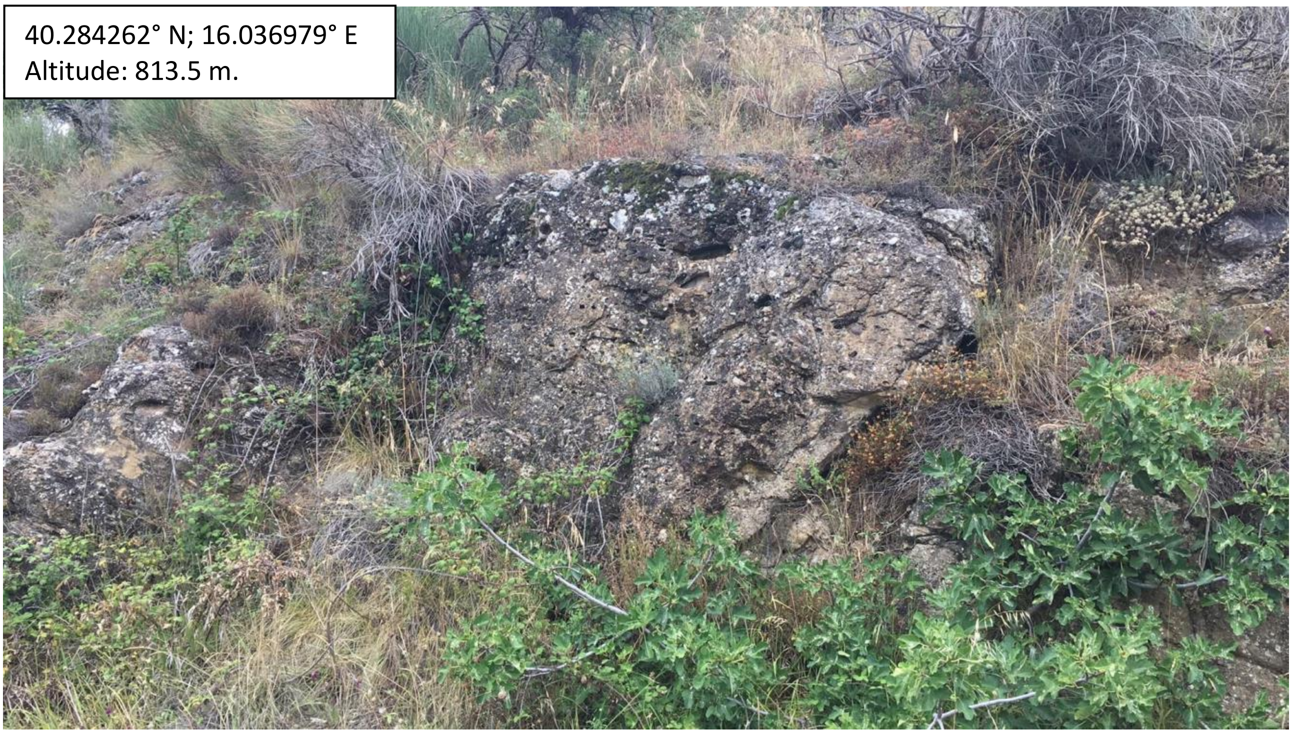

Figure 139.

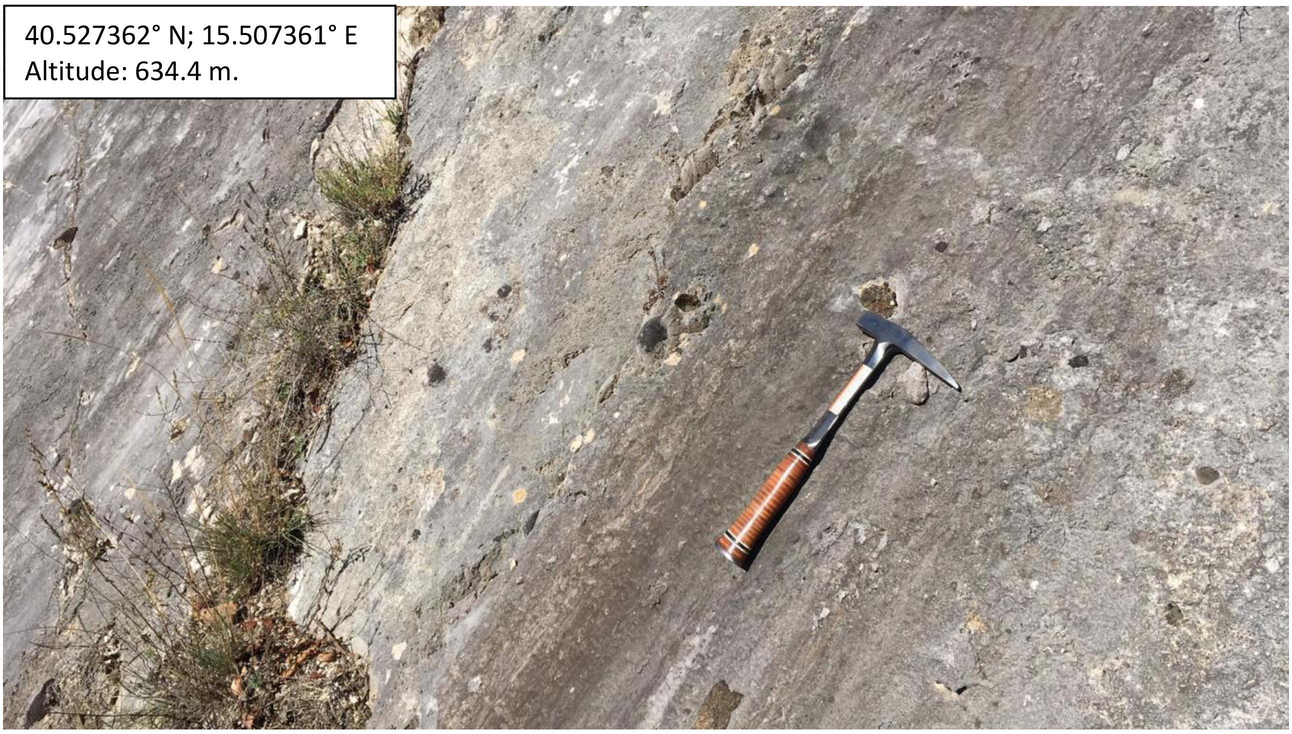

Figure 140.

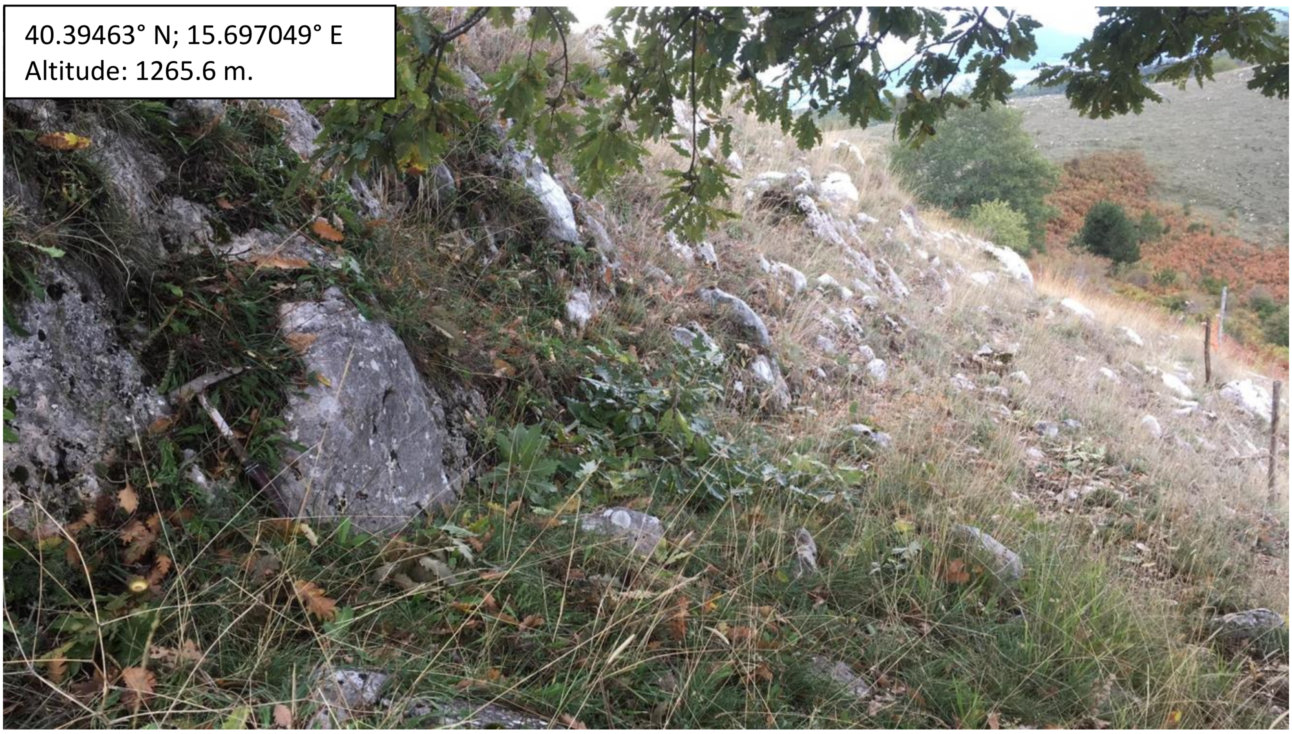

Figure 141.

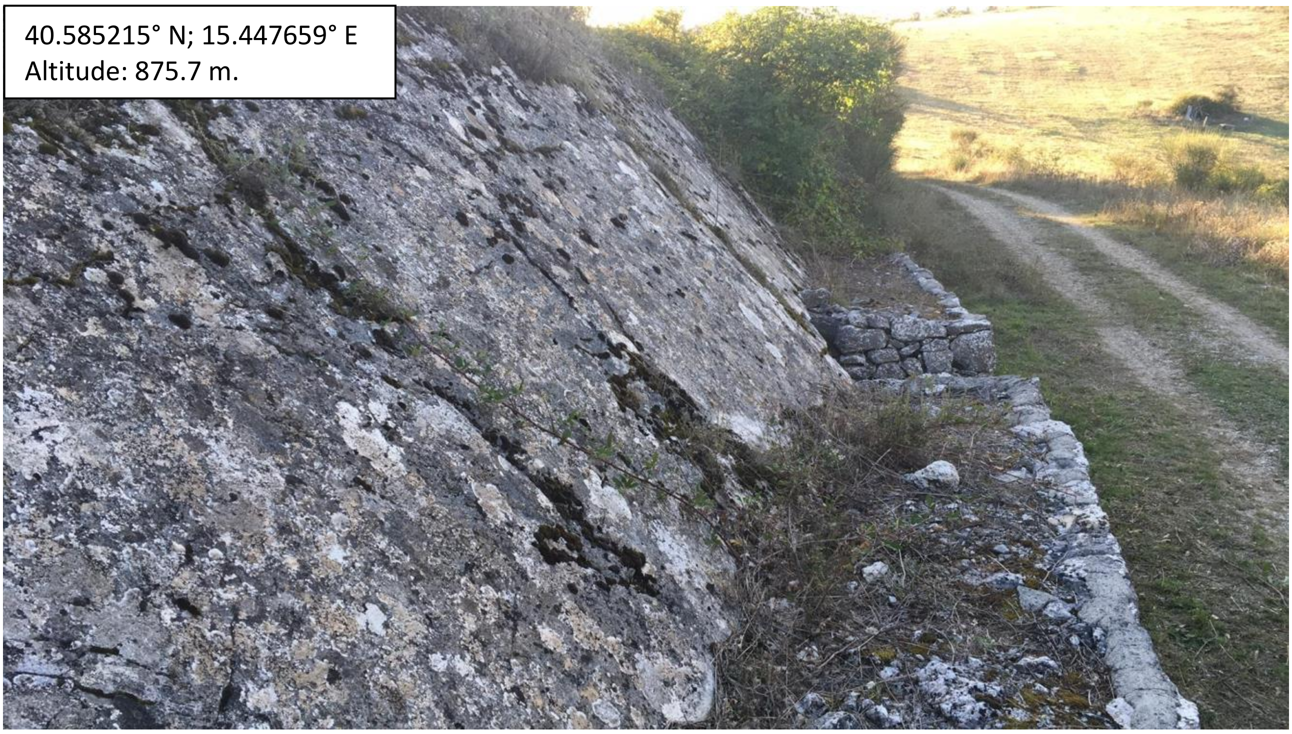

Figure 142.

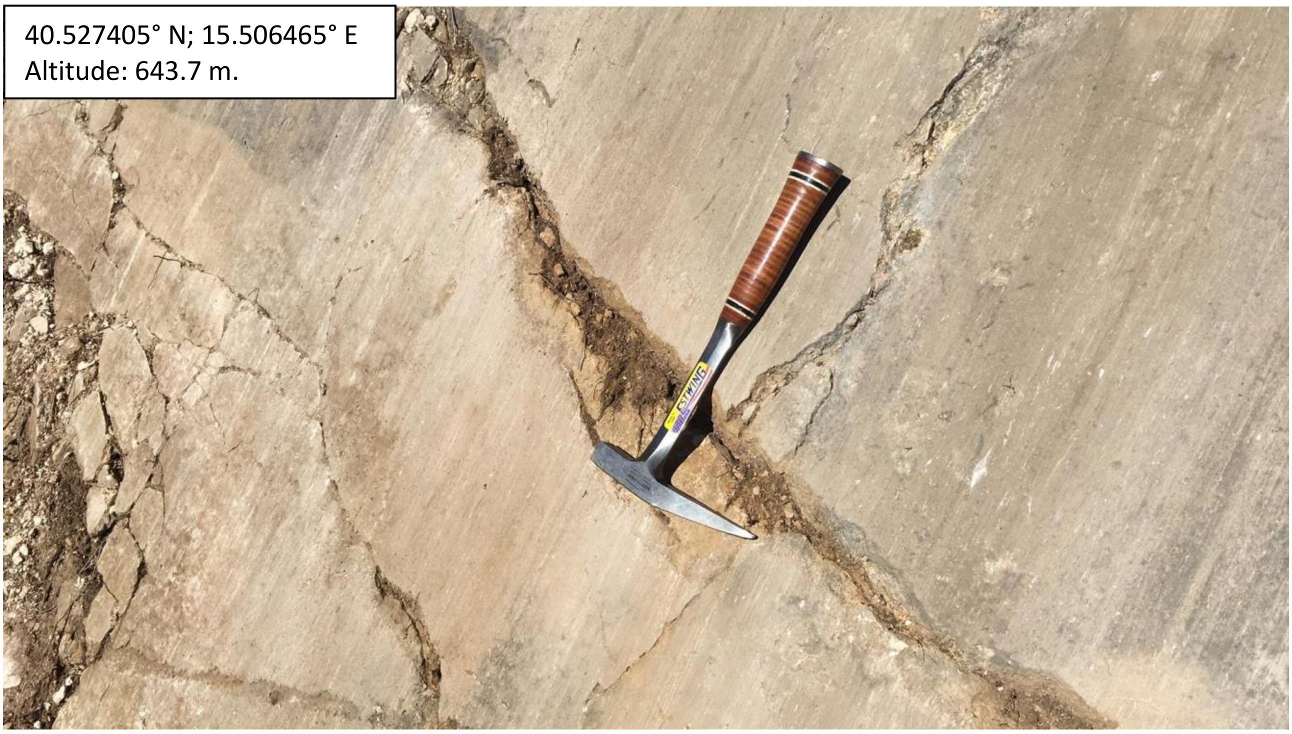

Figure 143.

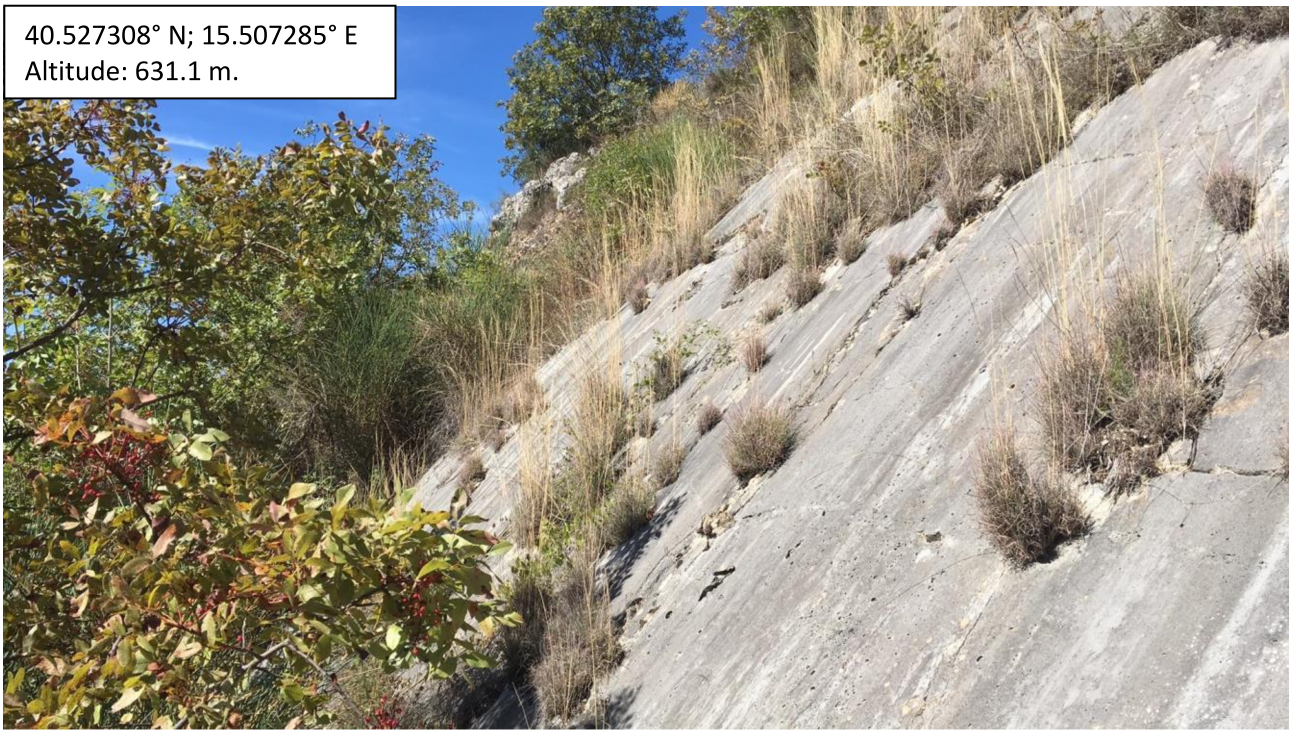

Figure 144.

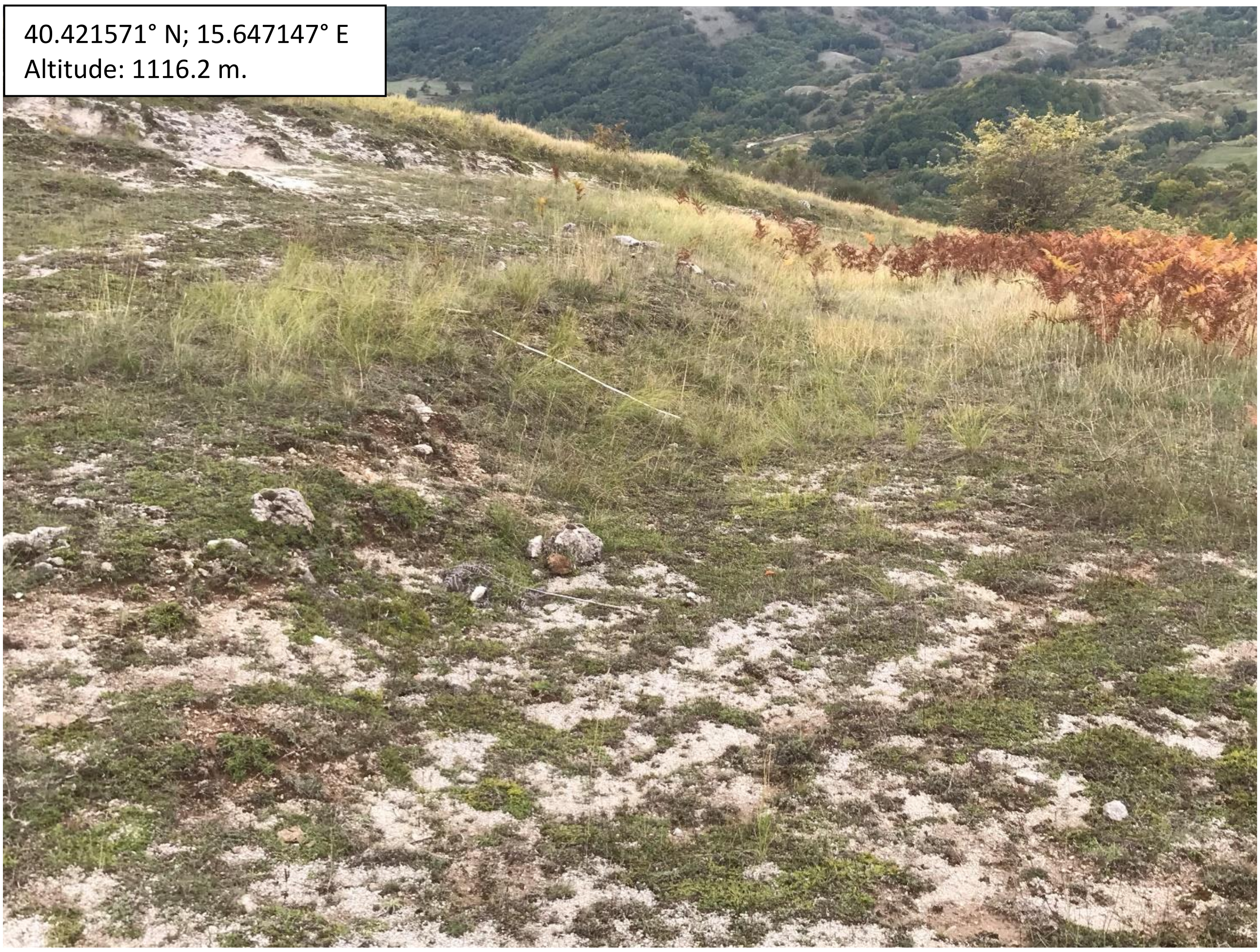

Figure 145.

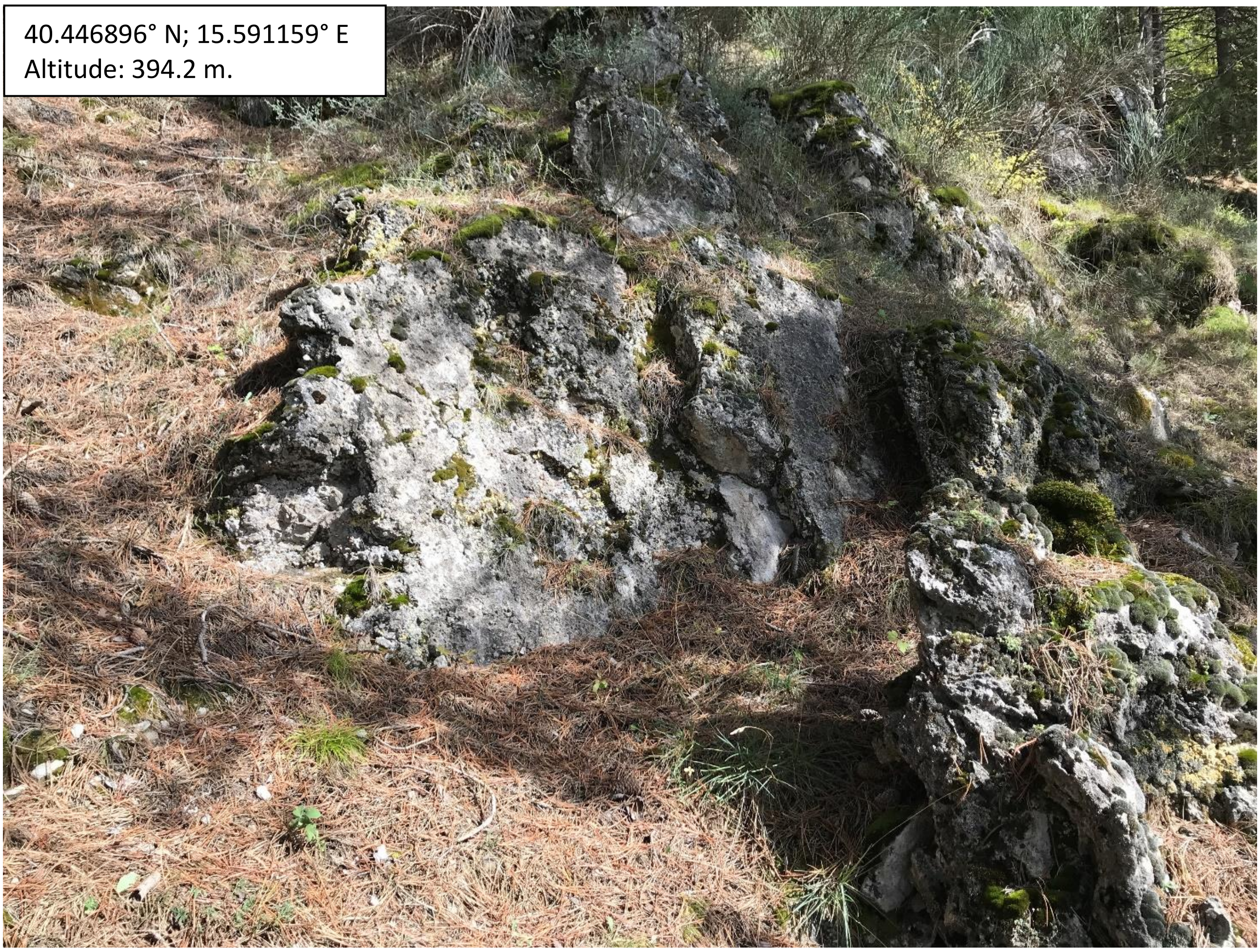

Figure 146.

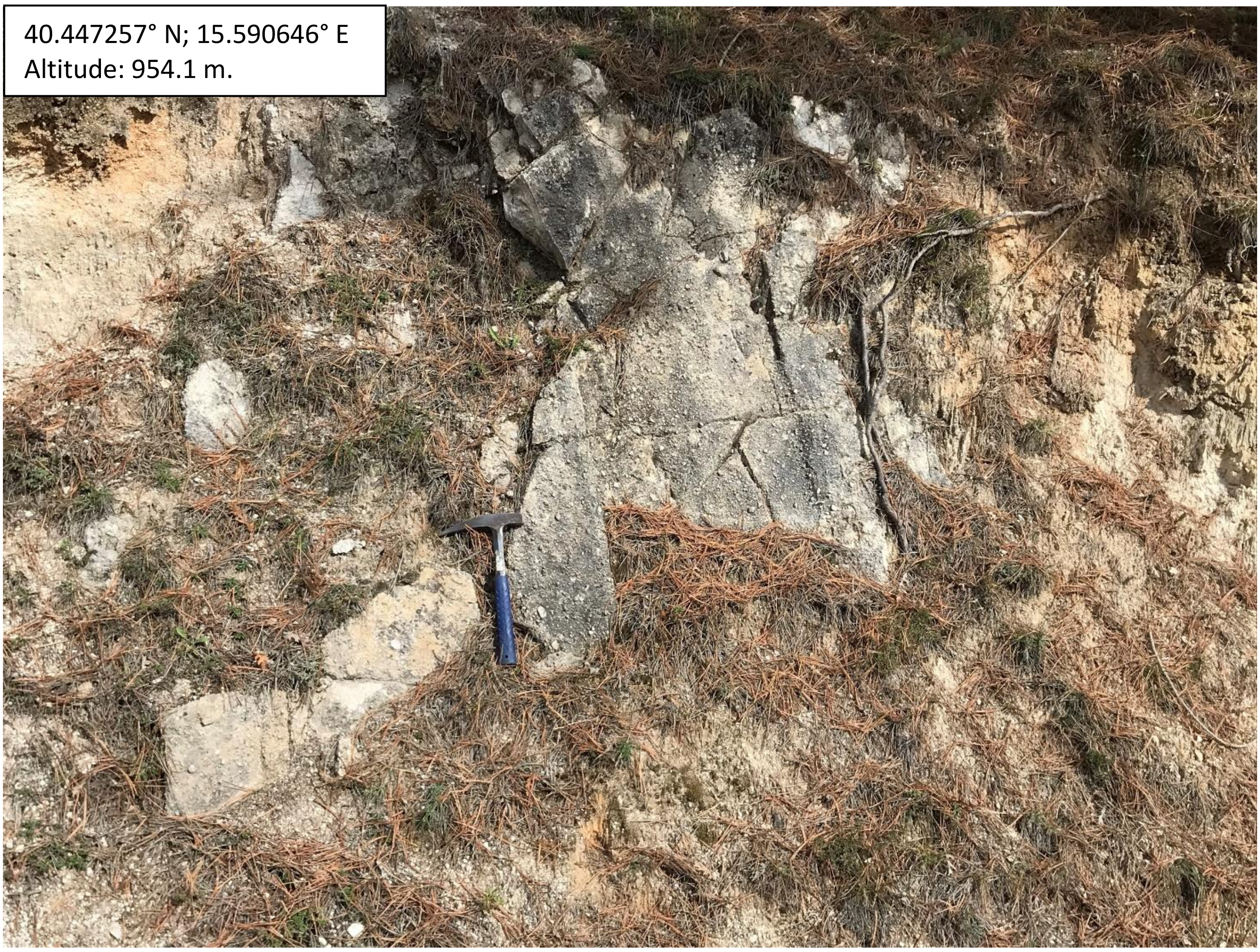

Figure 147.

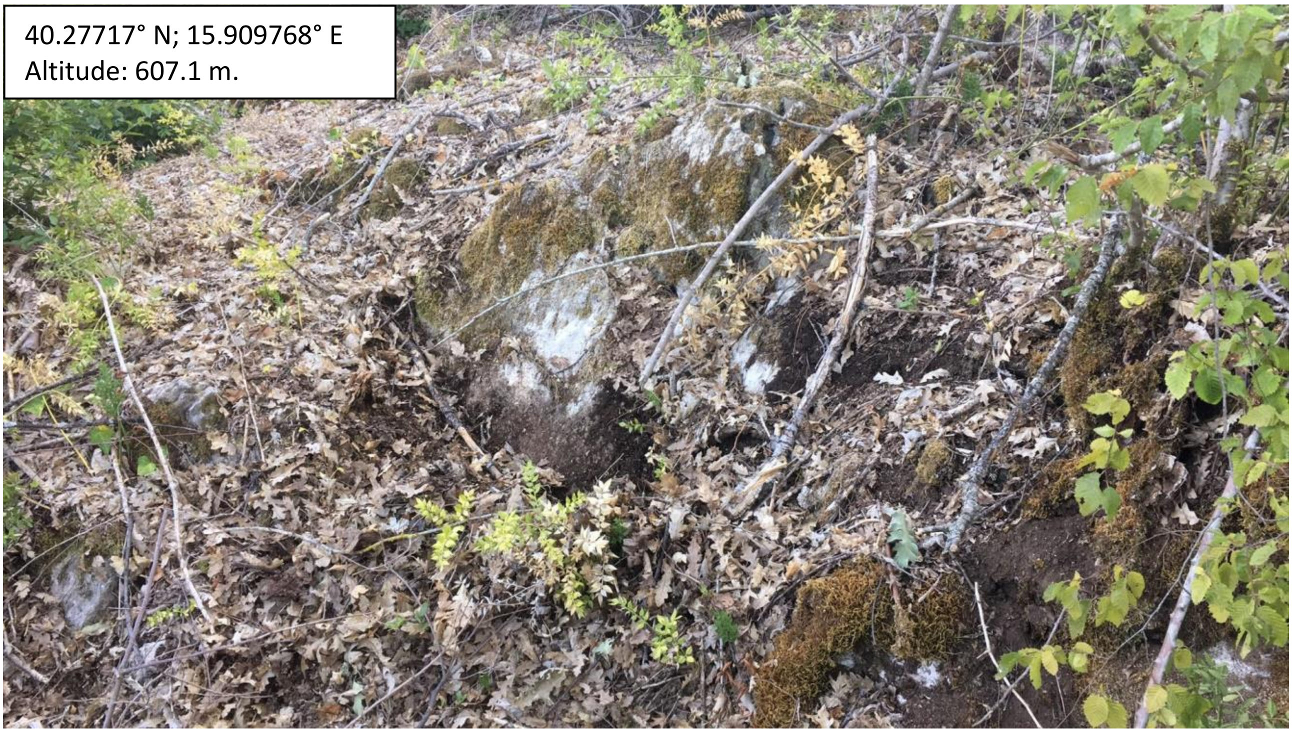

Figure 148.

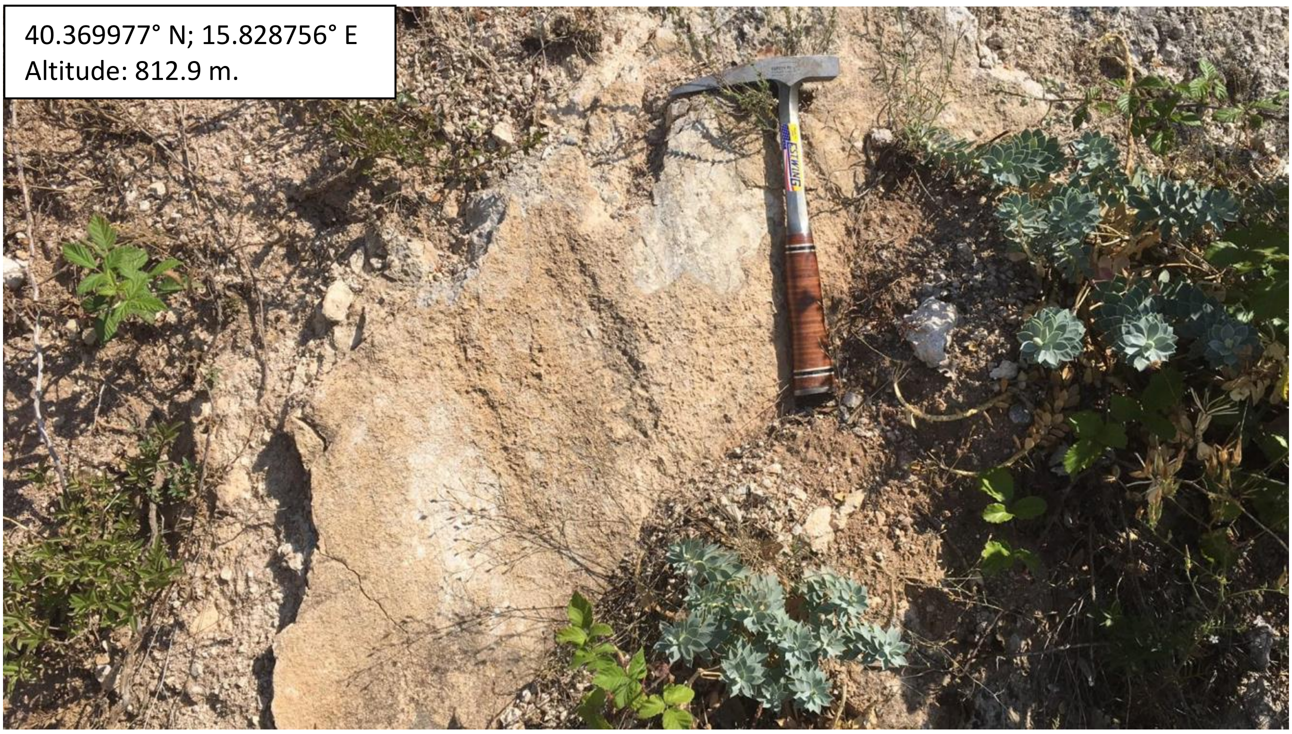

Figure 149.

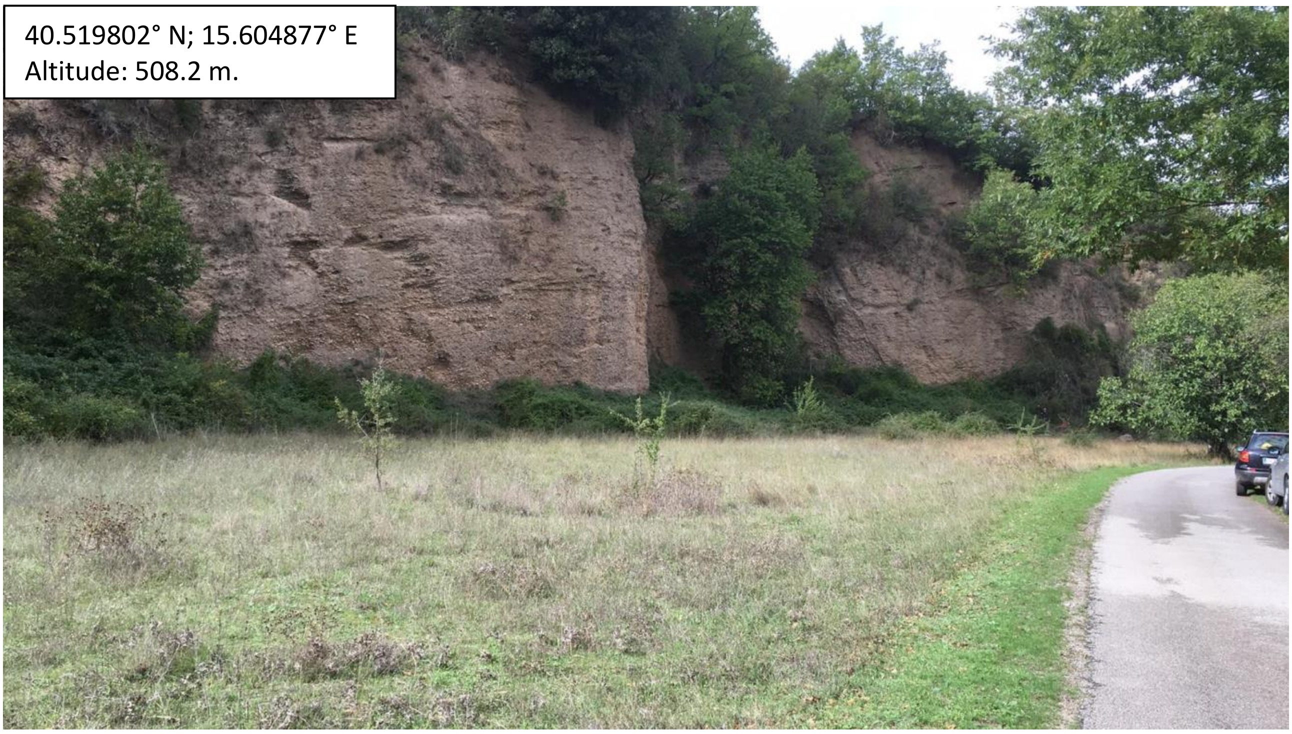

Figure 150.

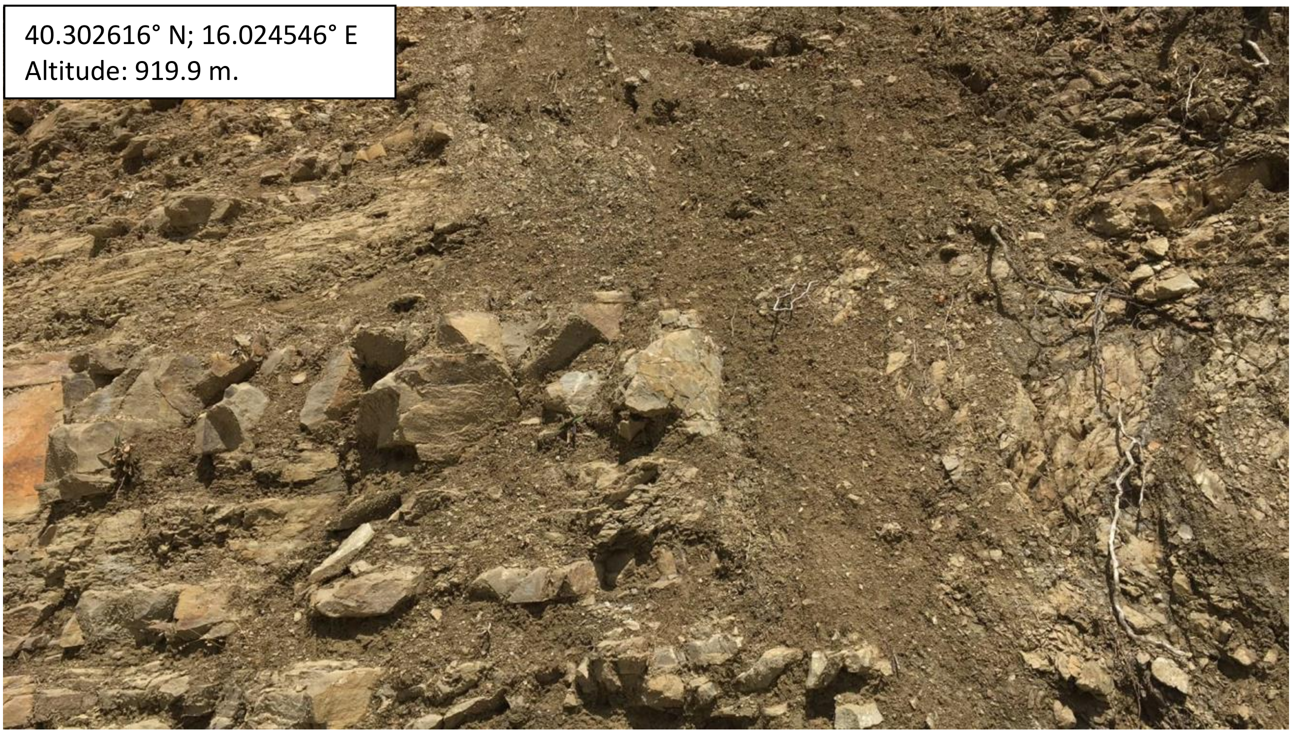

Figure 151.

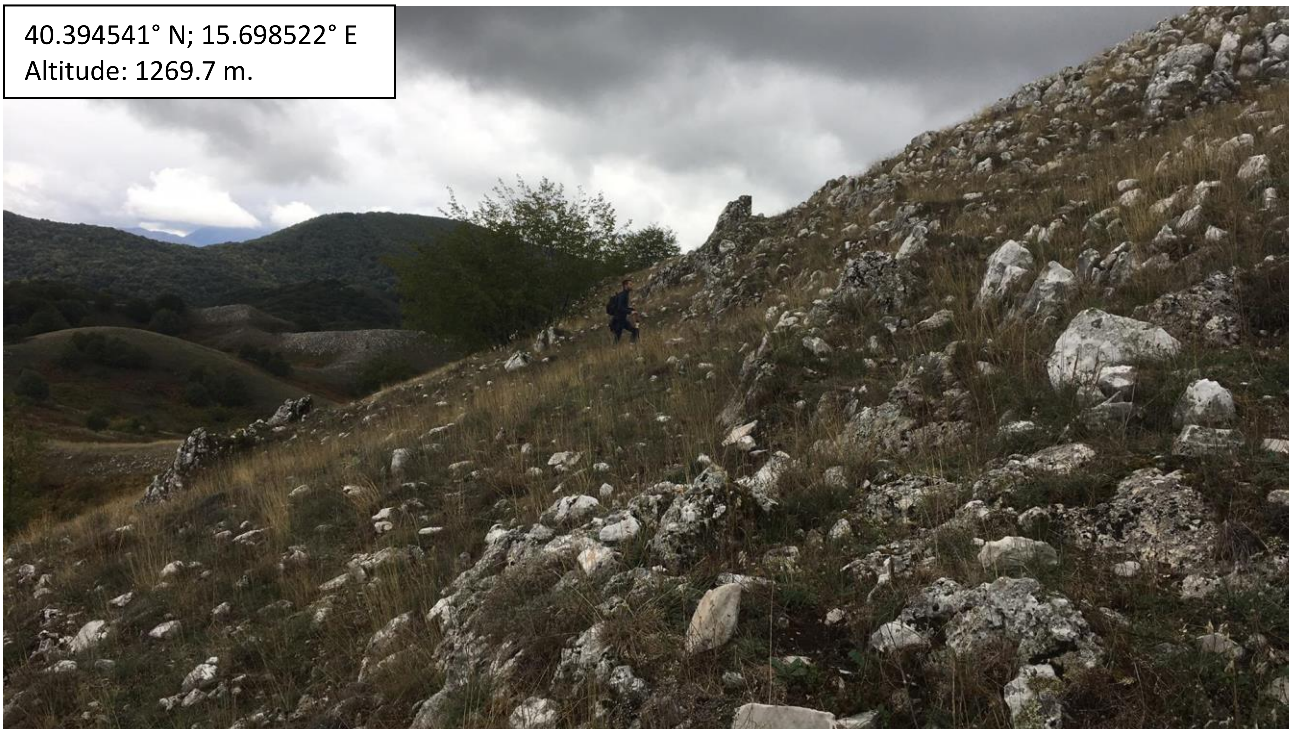

Figure 152.

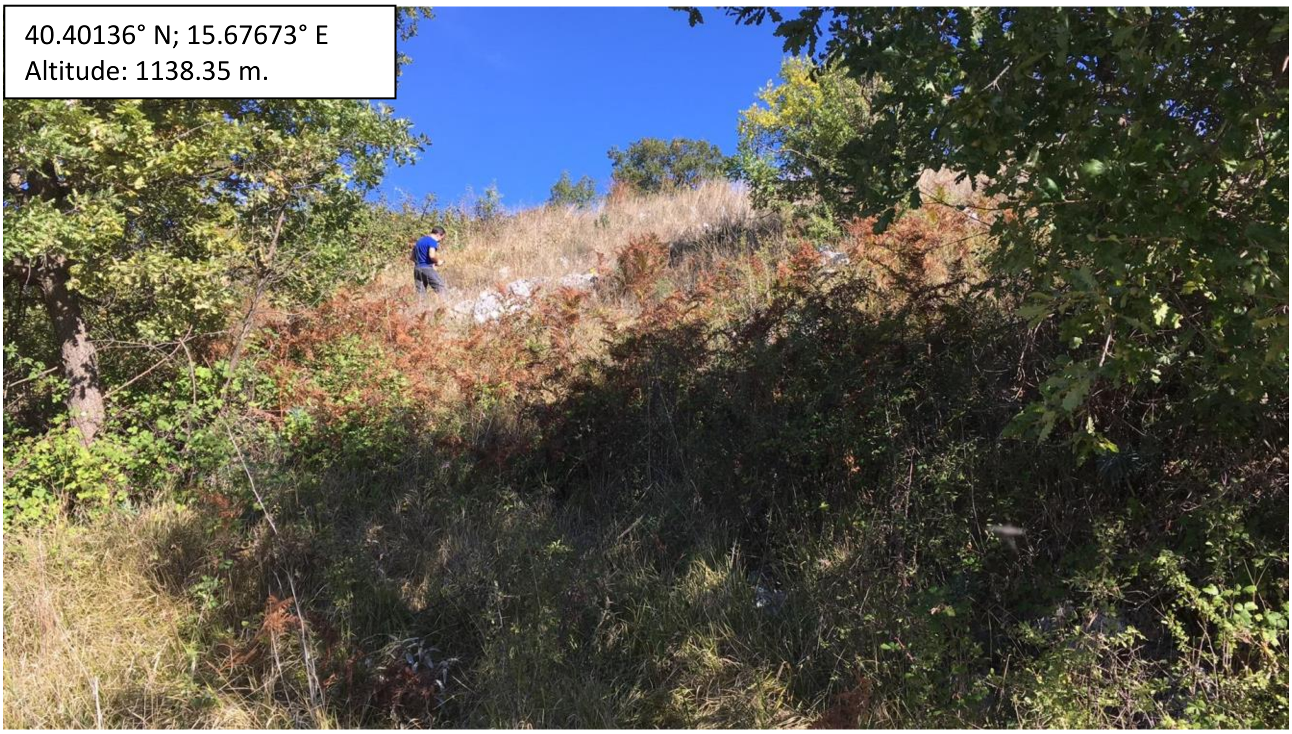

Figure 153.

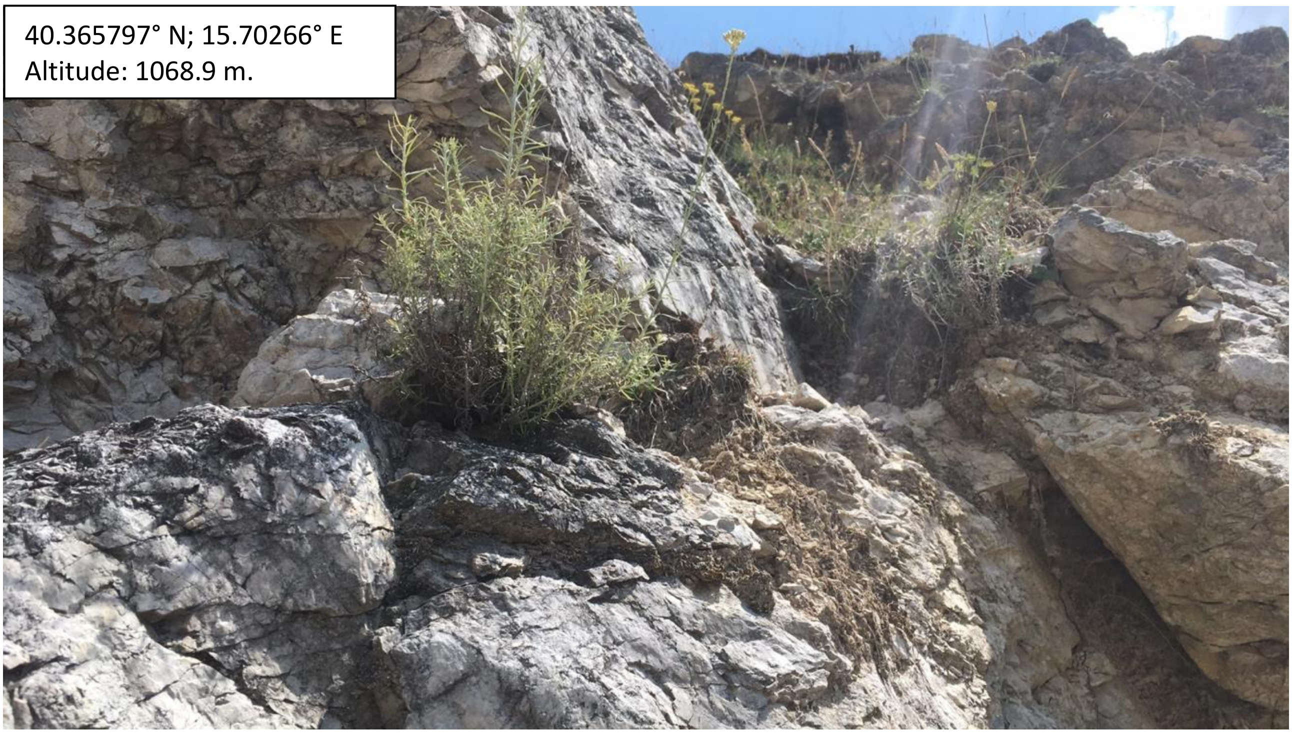

Figure 154.

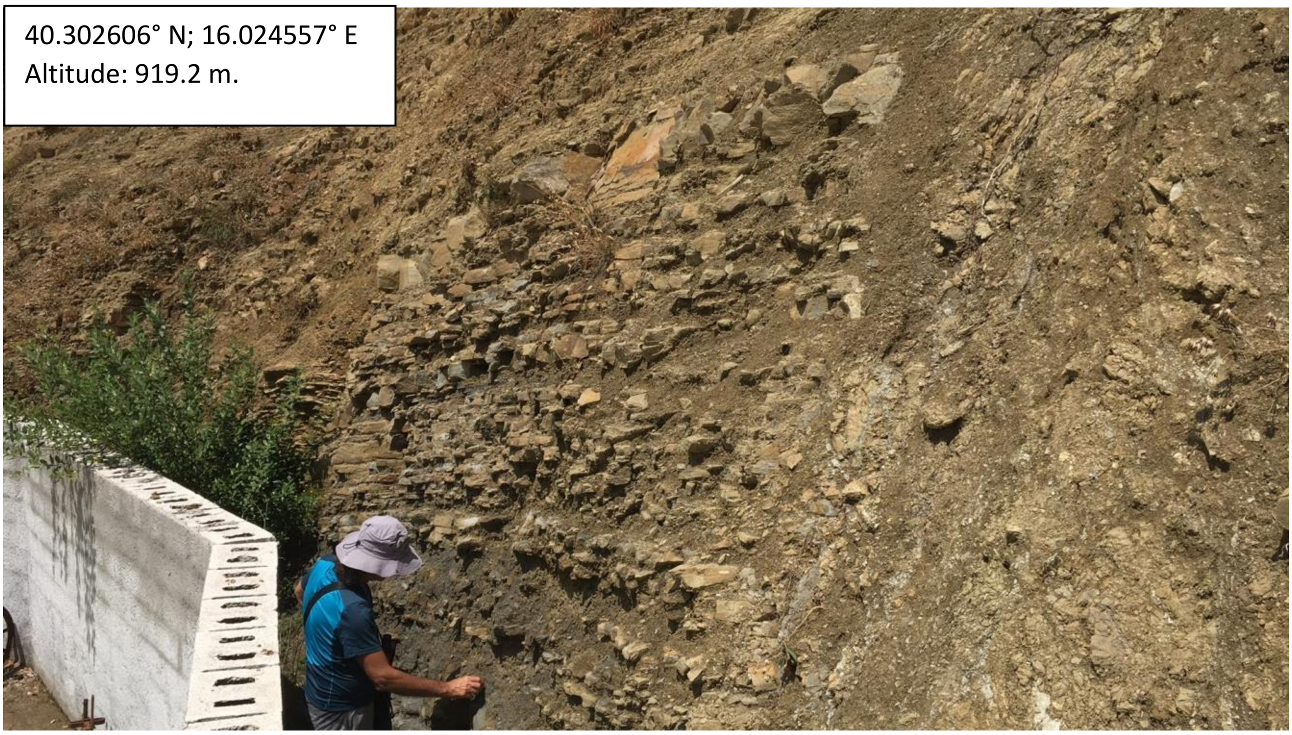

Figure 156.

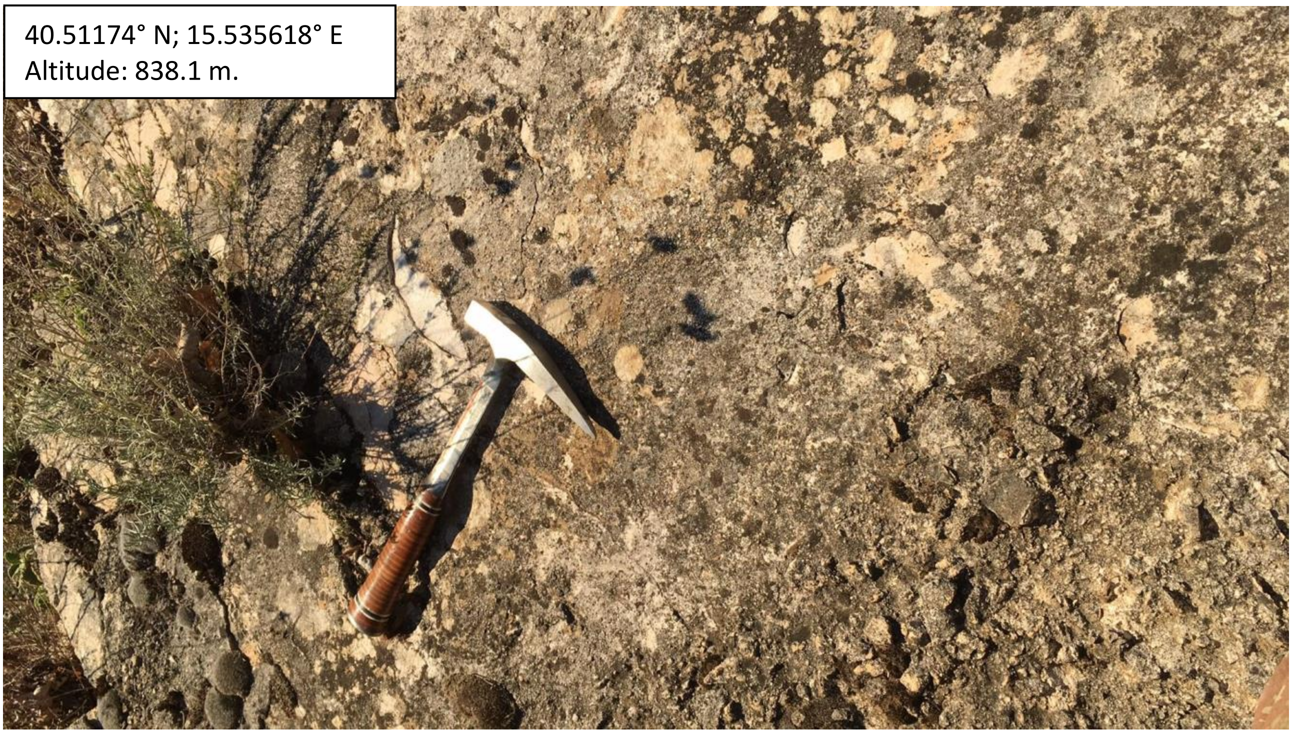

Figure 157.

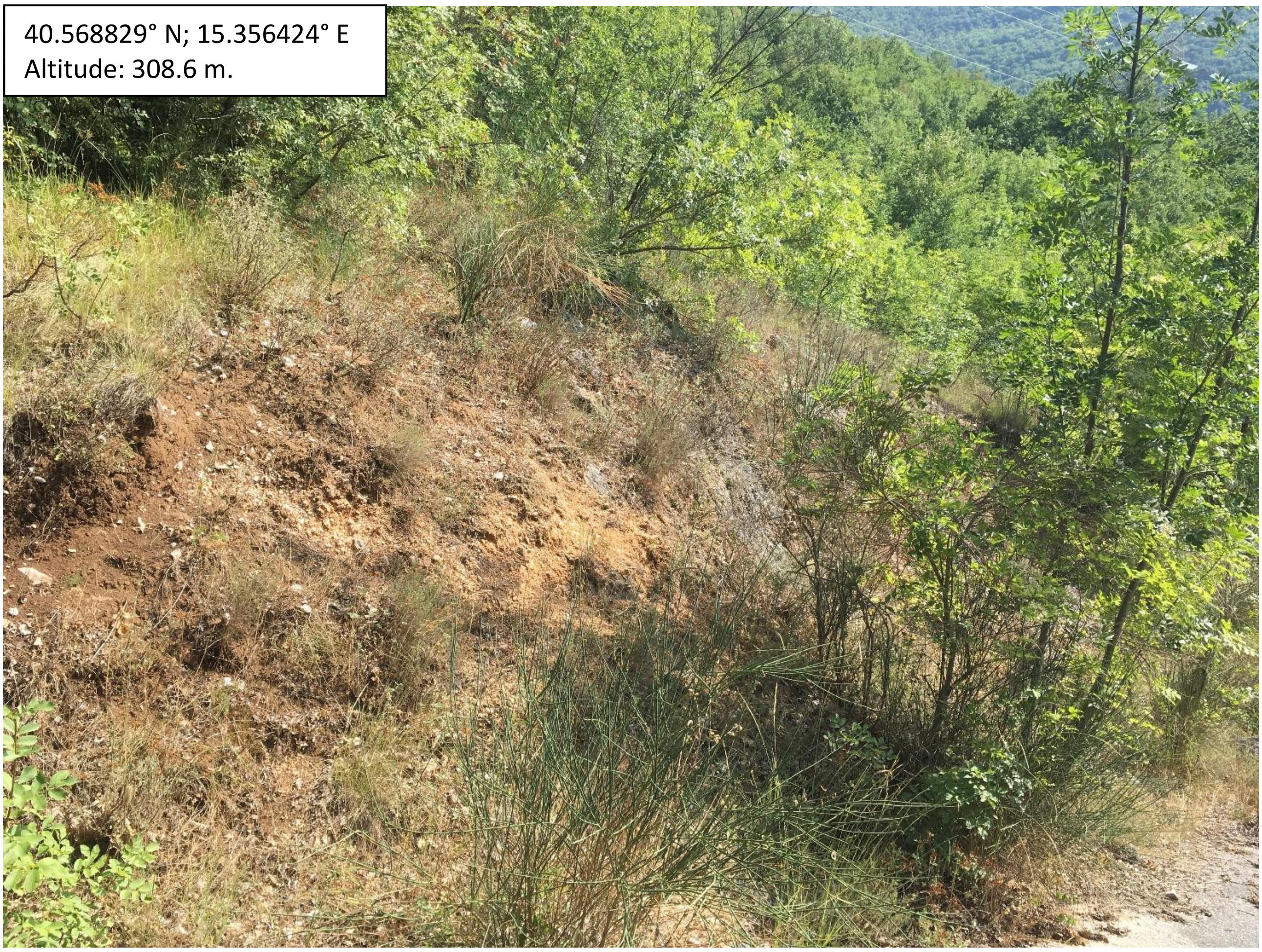

Figure 158.

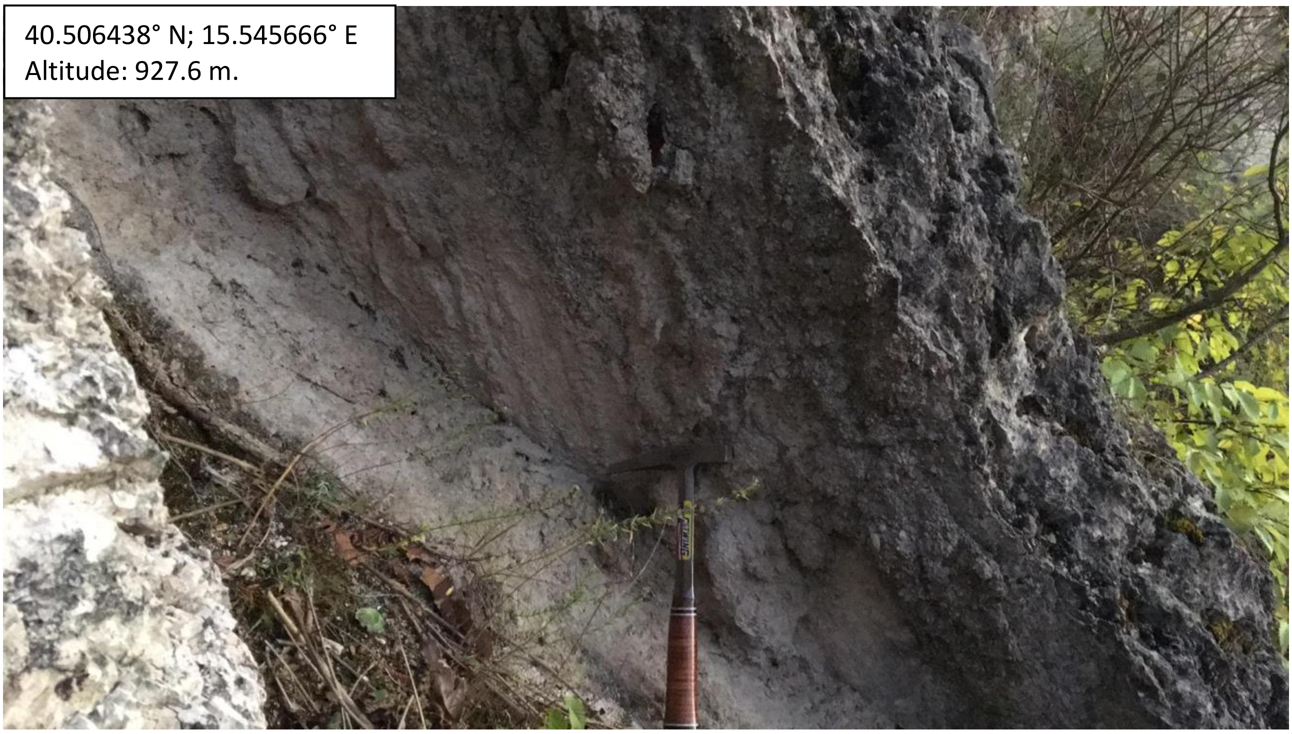

Figure 159.

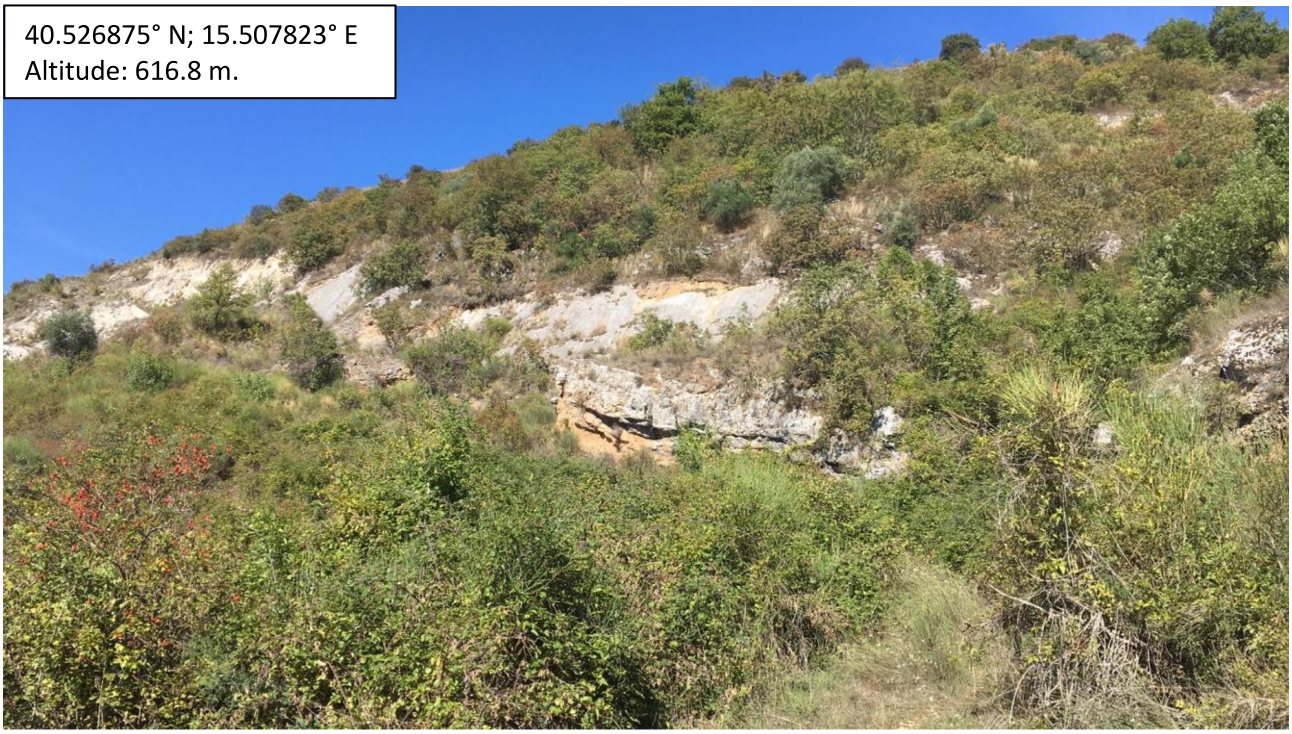

Figure 160.

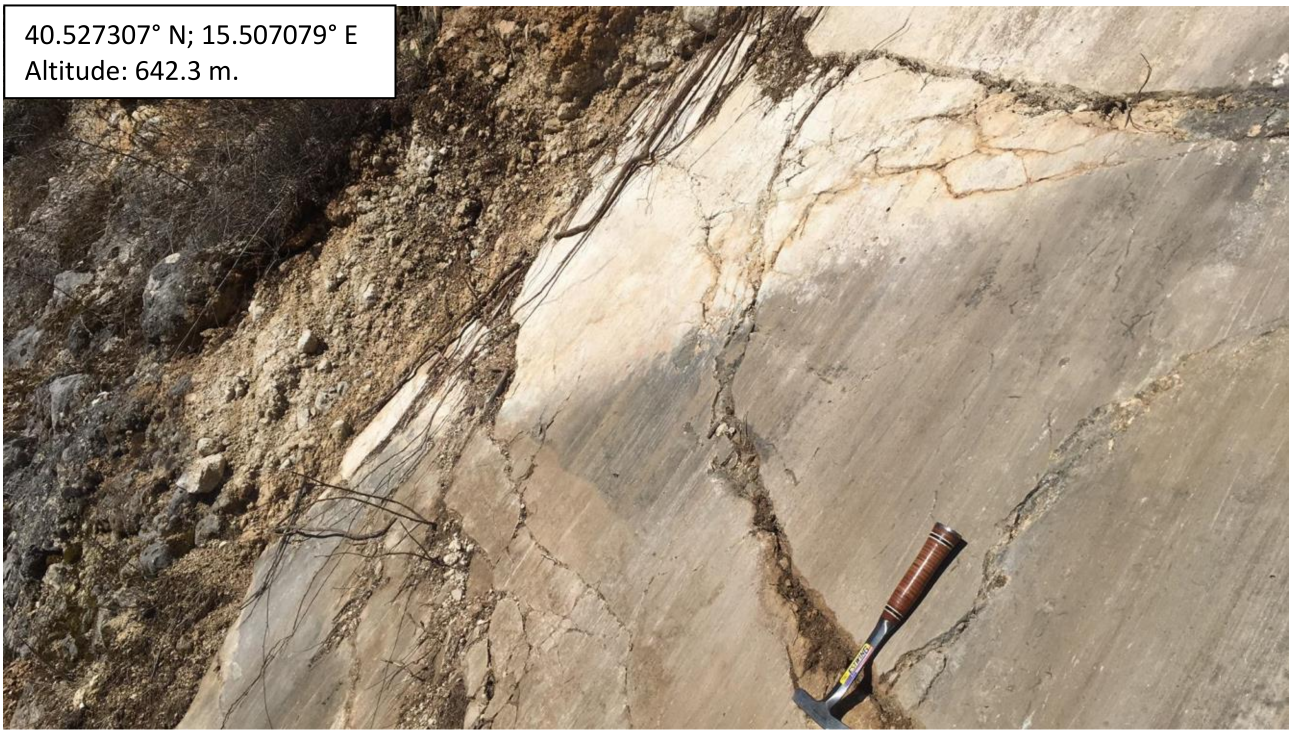

Figure 161.

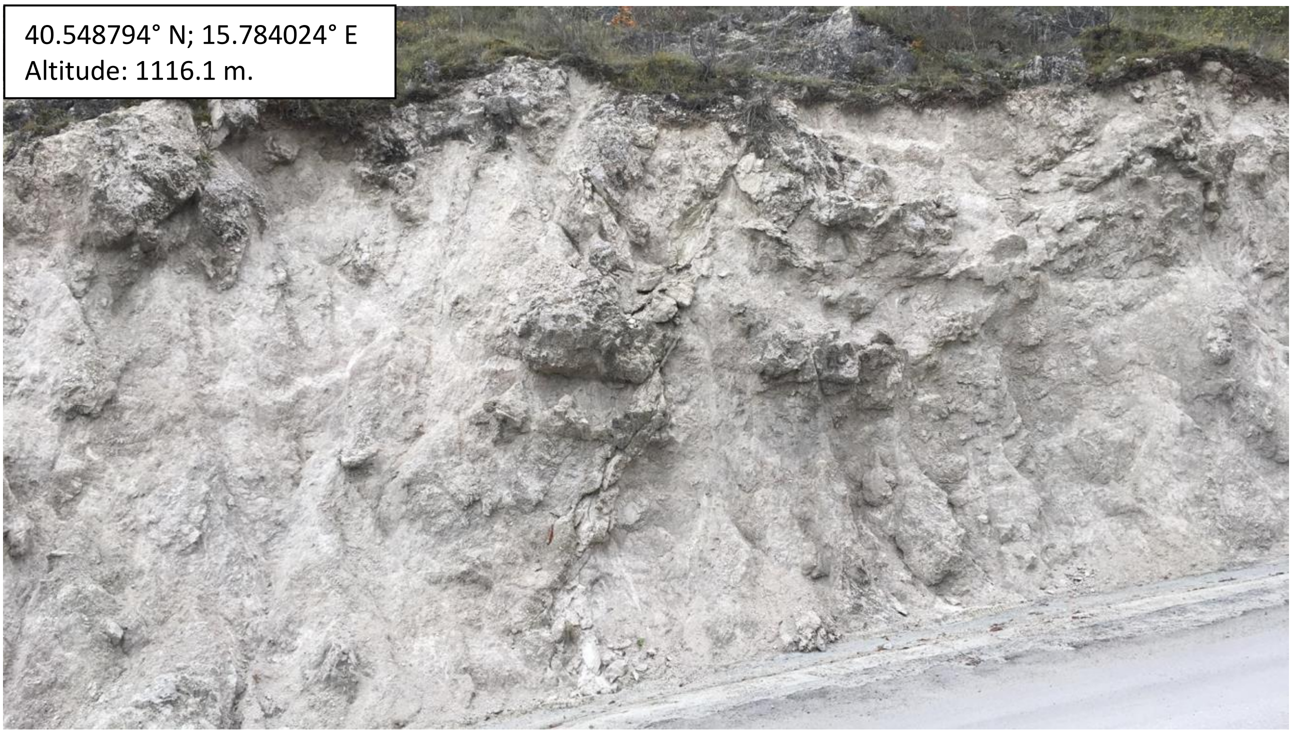

Figure 162.

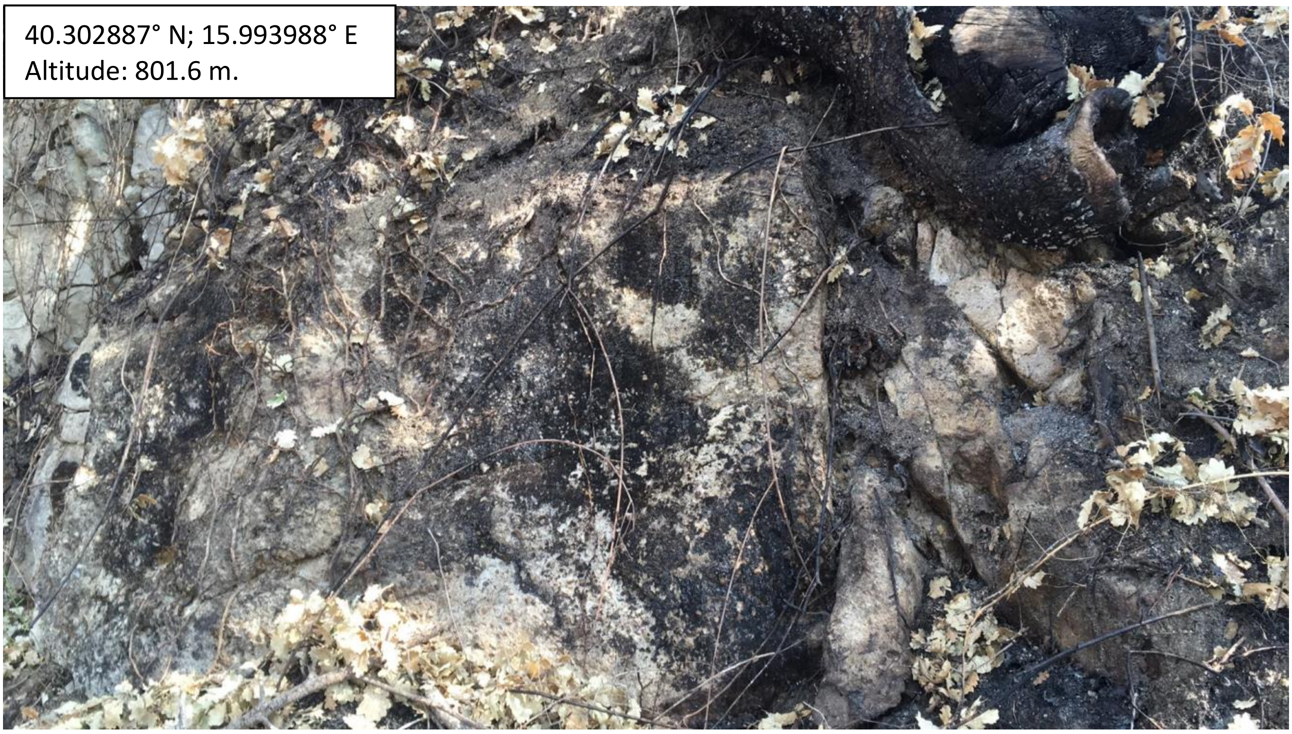

Figure 163.

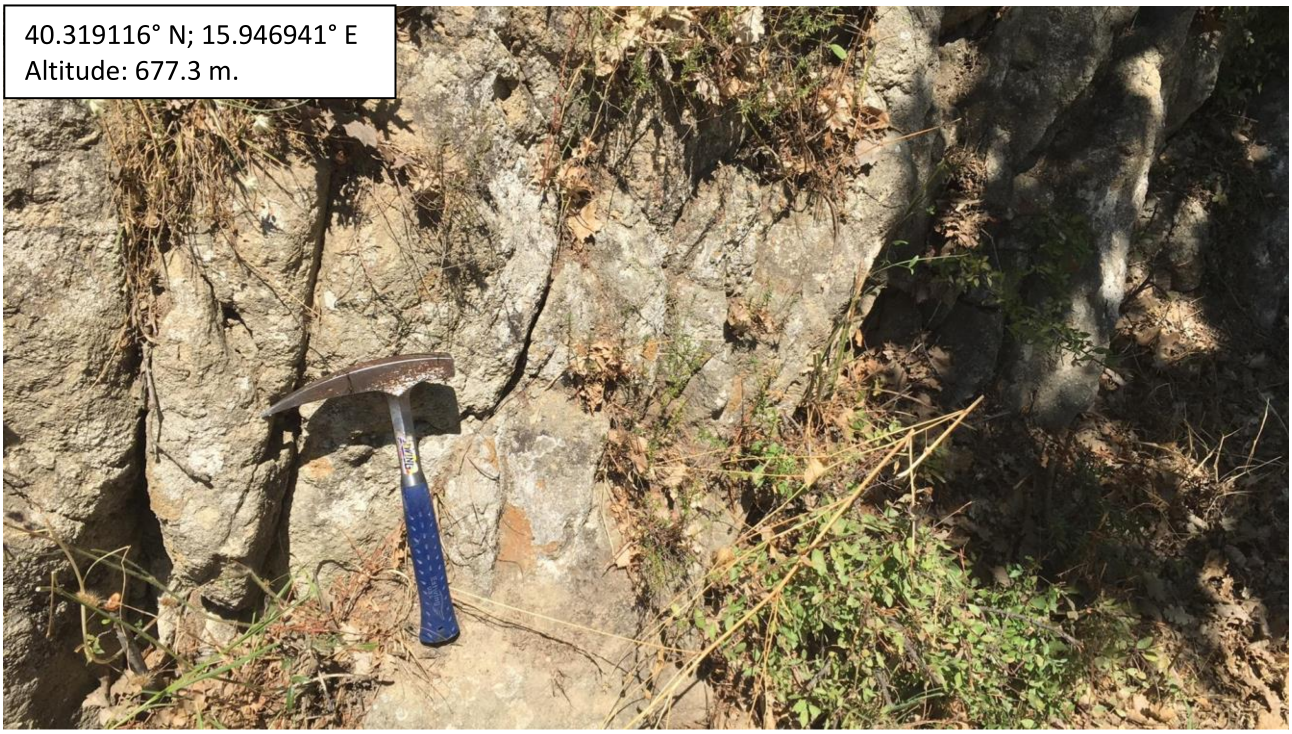

Figure 164.

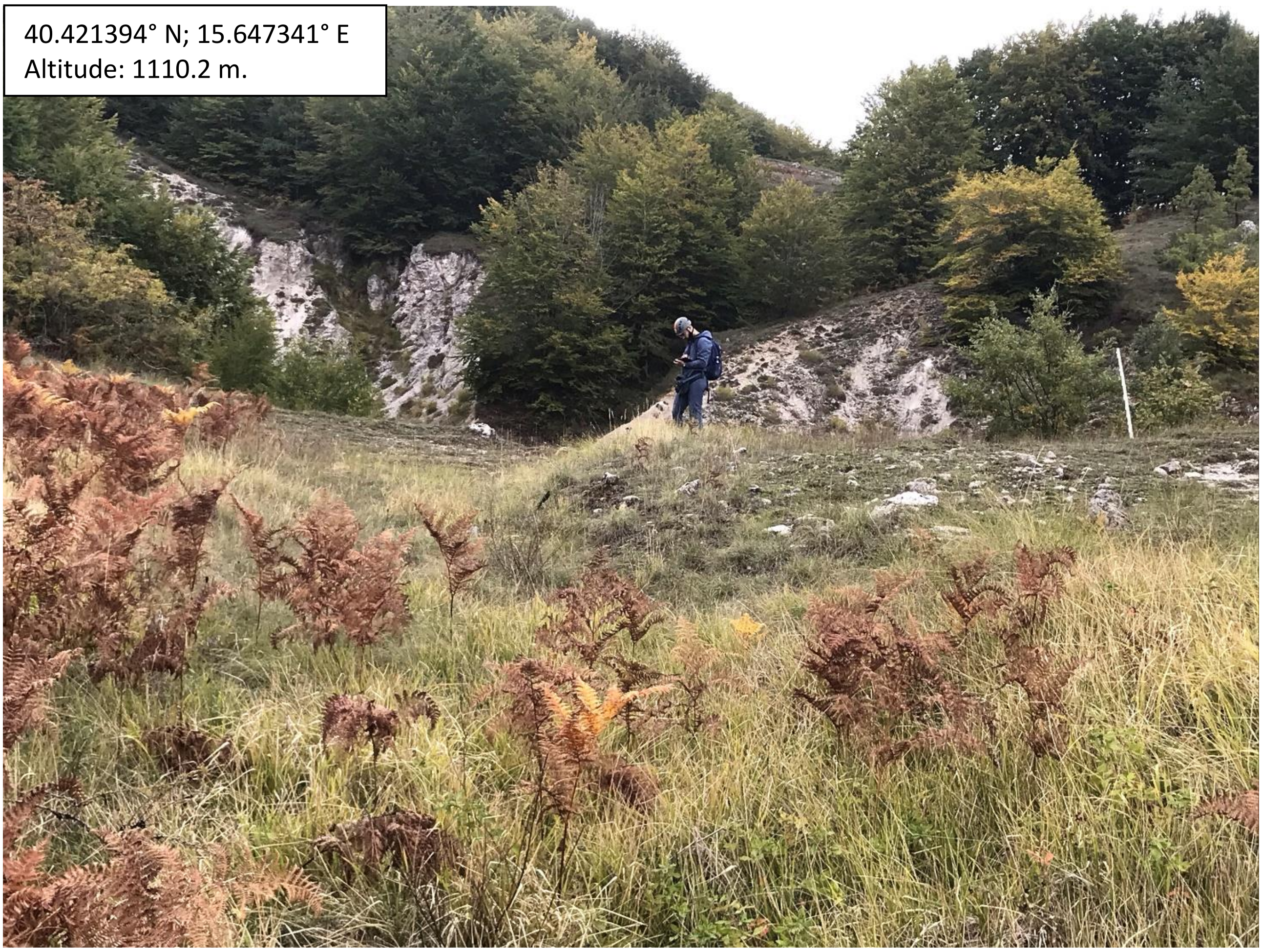

Figure 165.

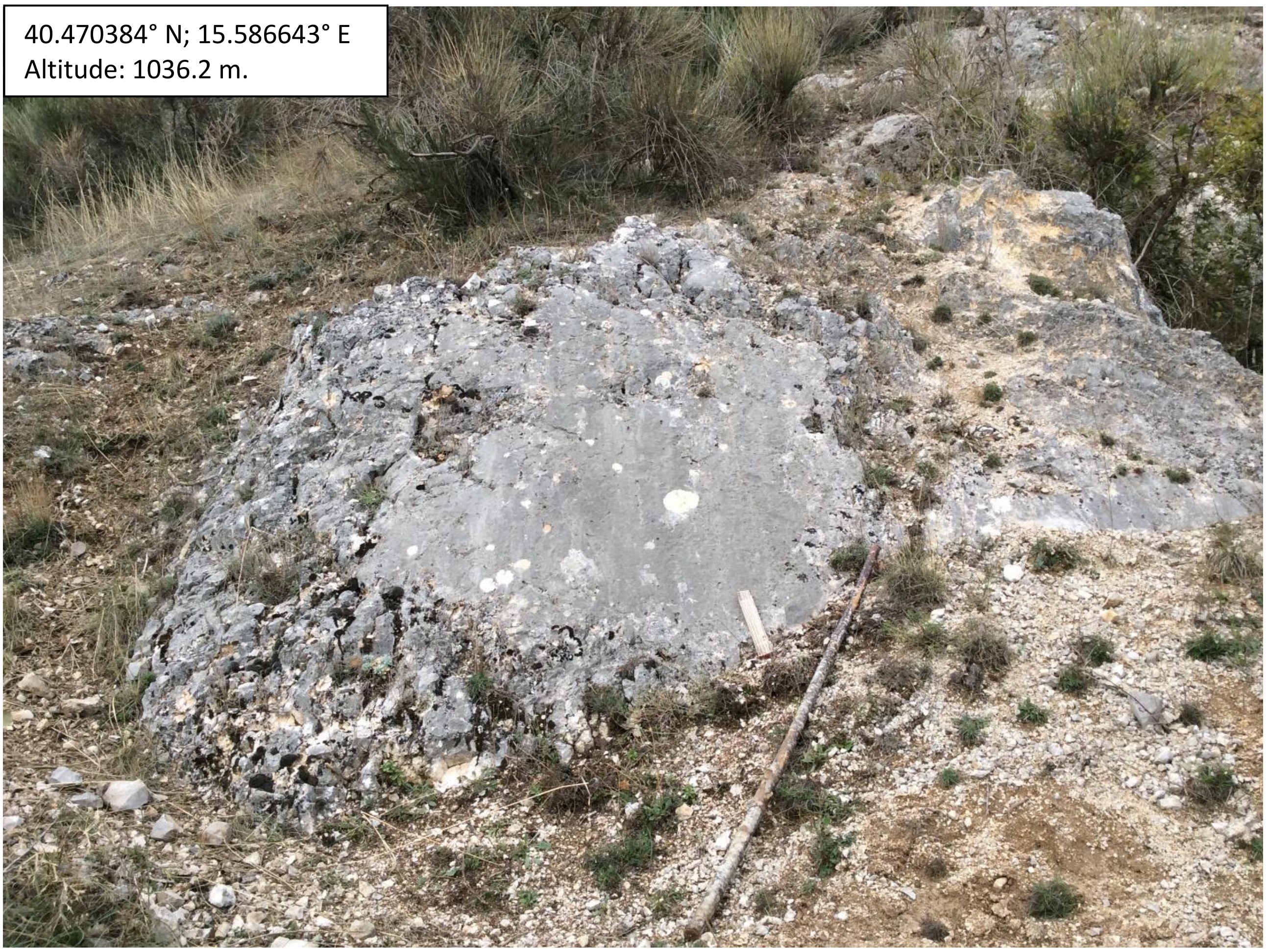

Figure 166

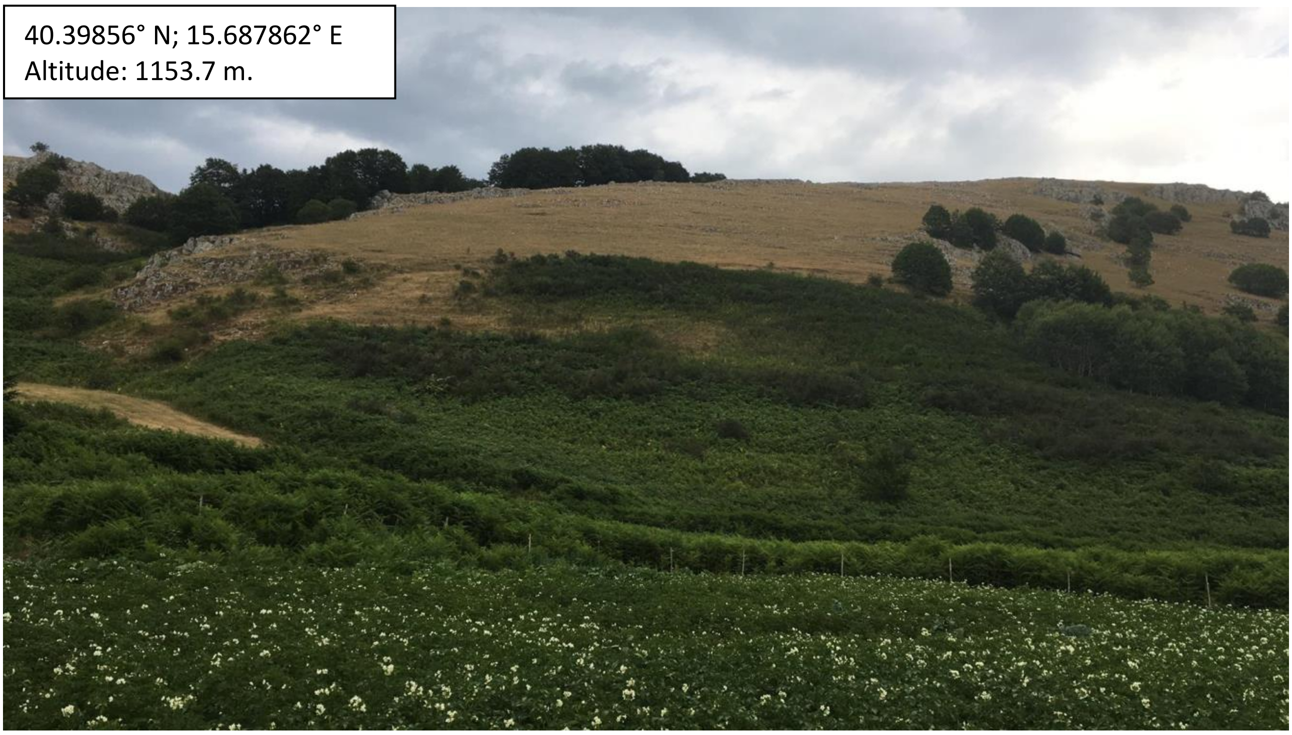

Figure 167.

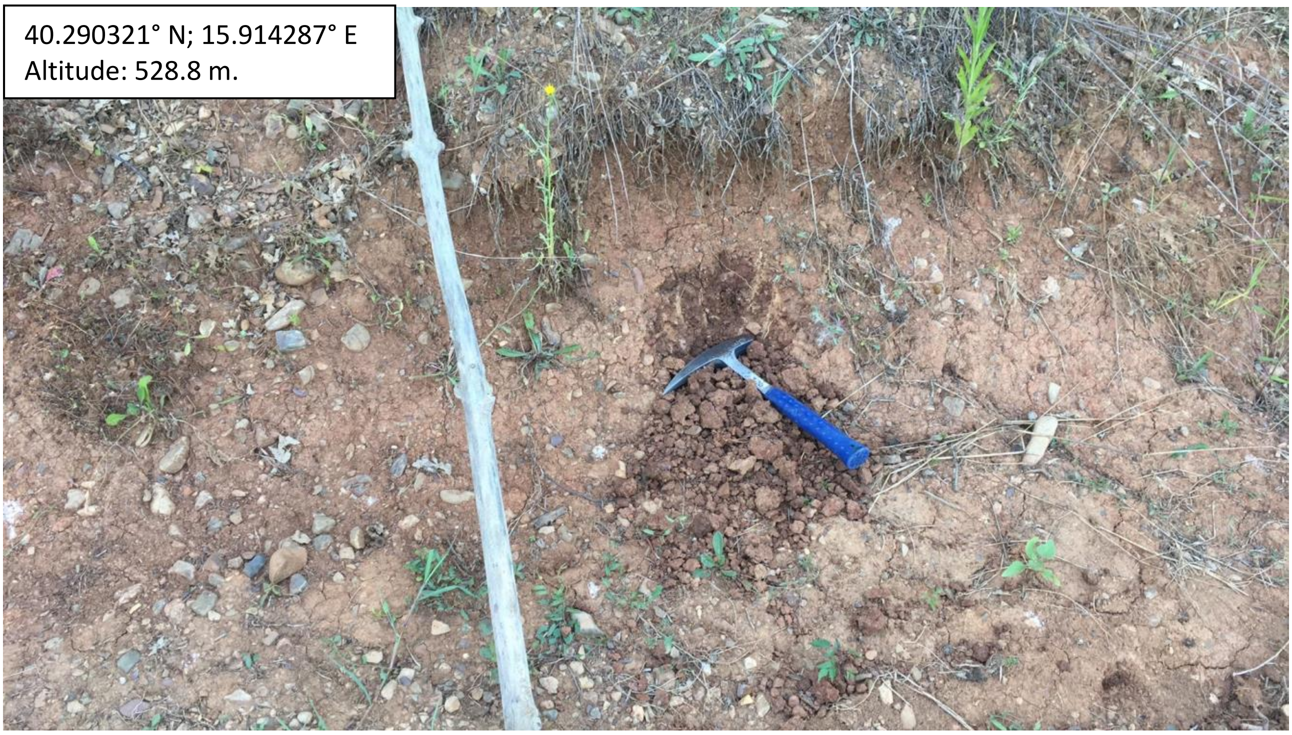

Figure 168.

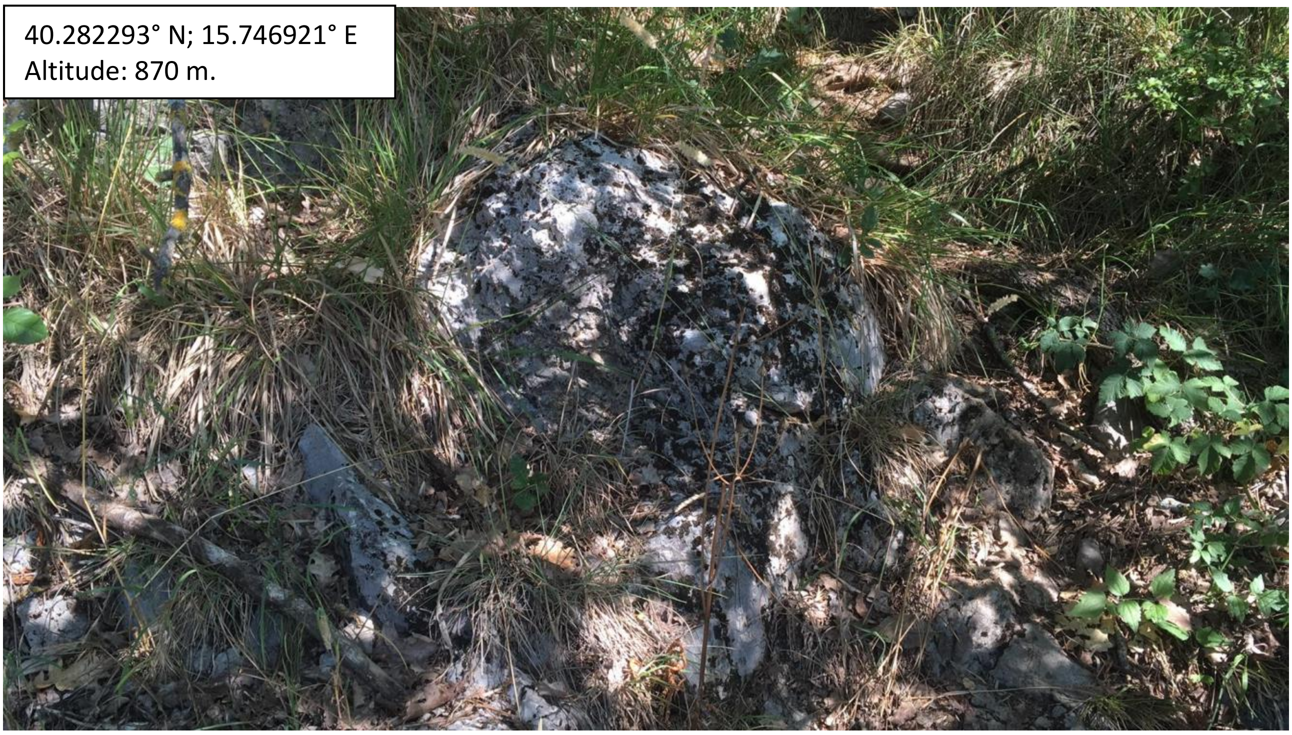

Figure 169.

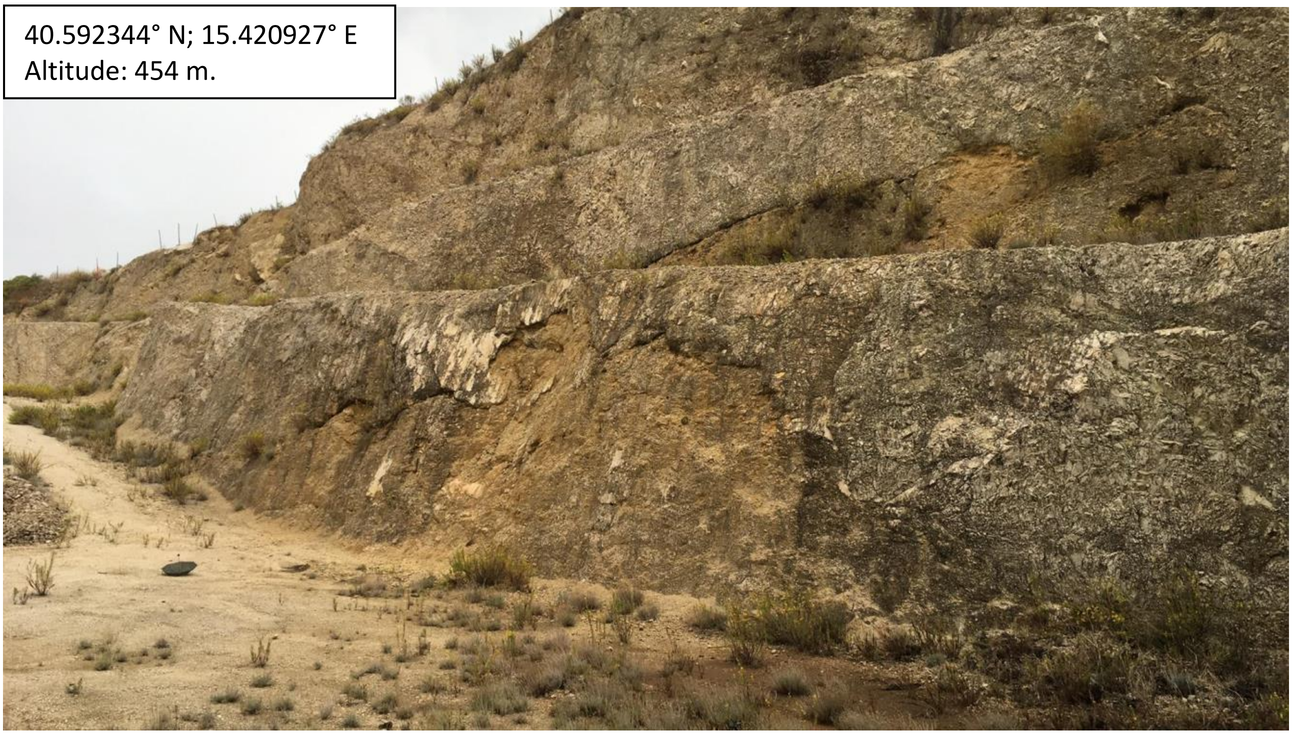

Figure 170.

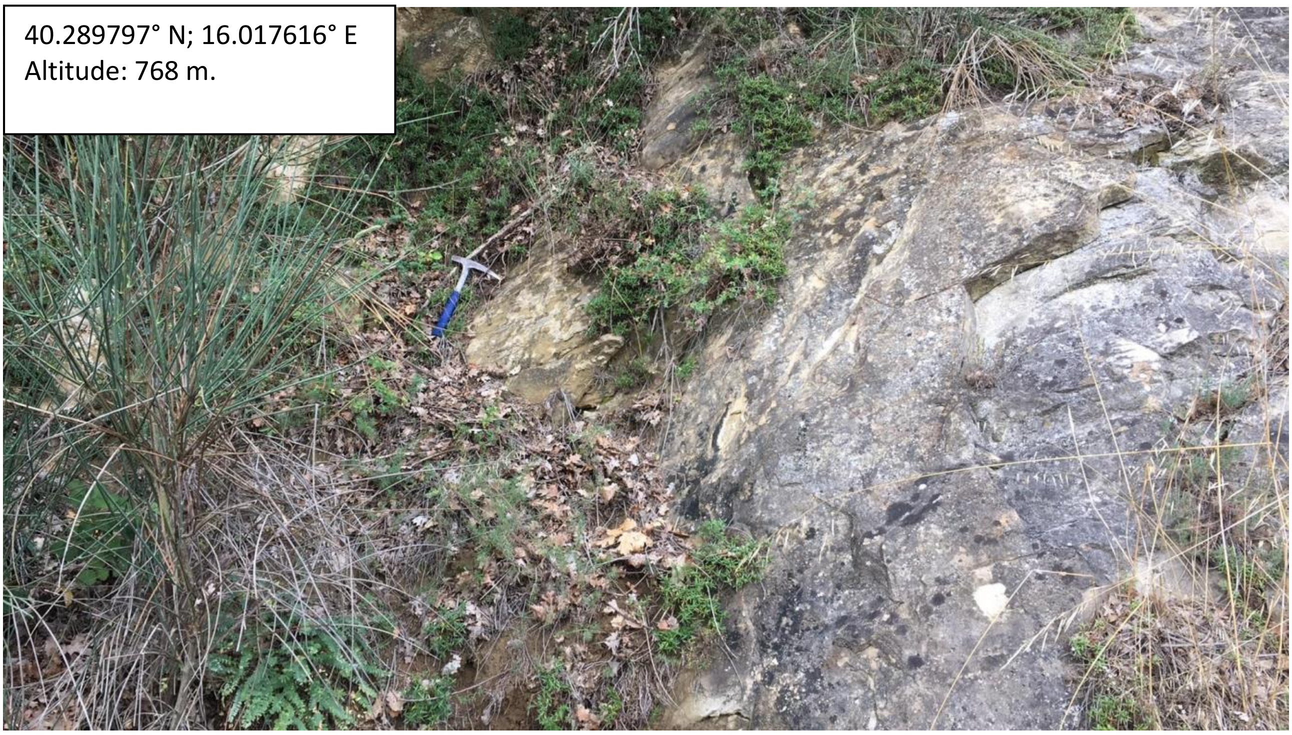

Figure 171.

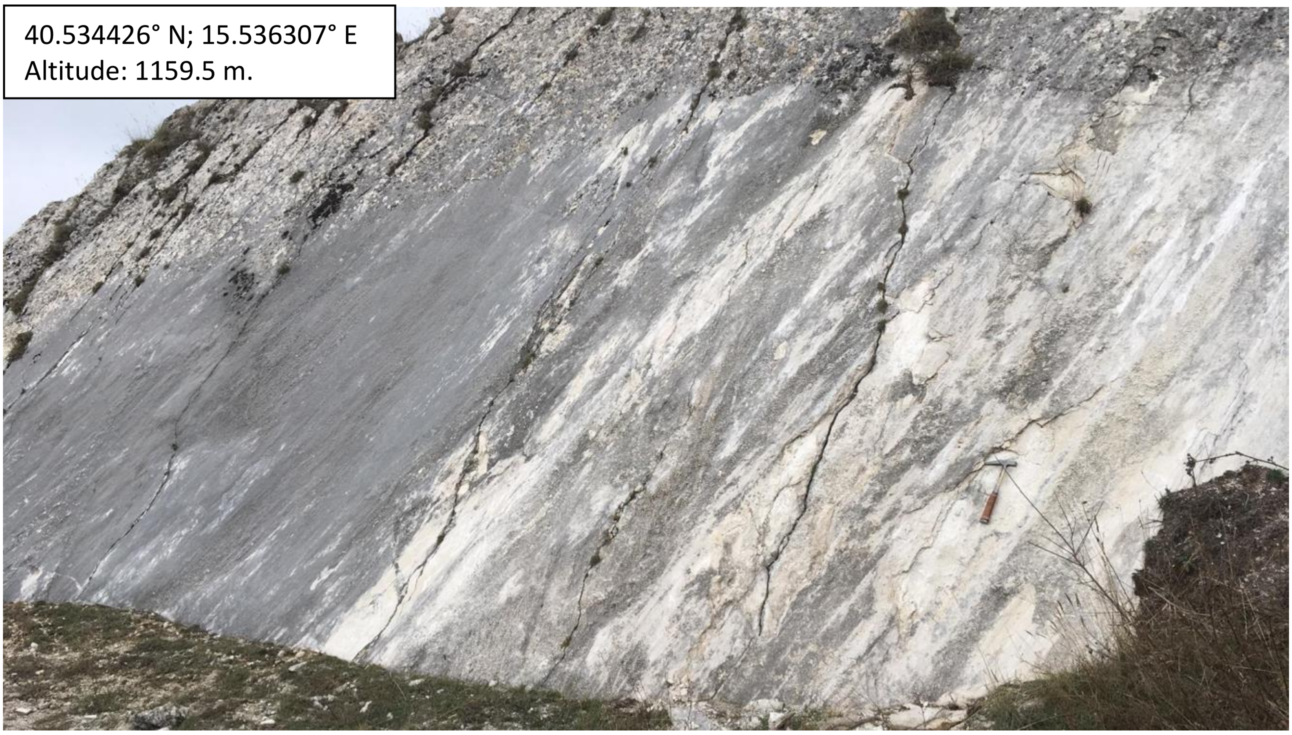

Figure 172.

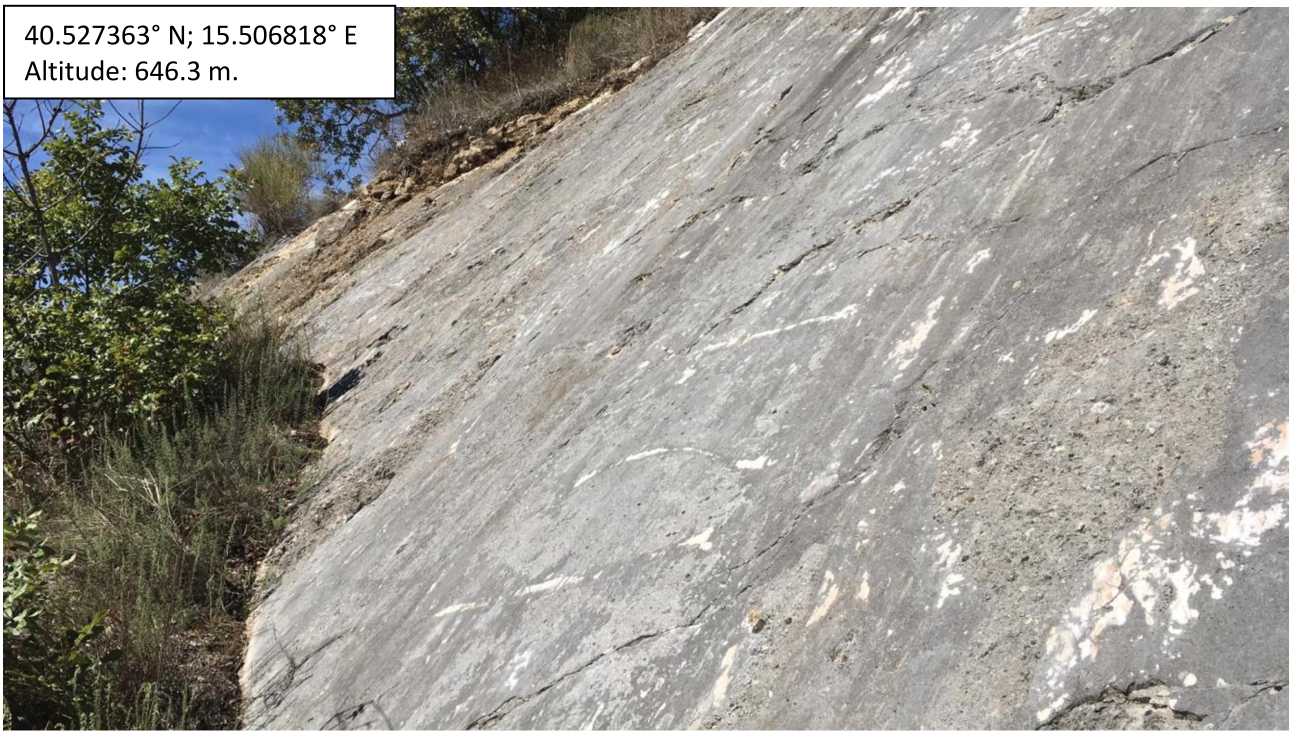

Figure 174.

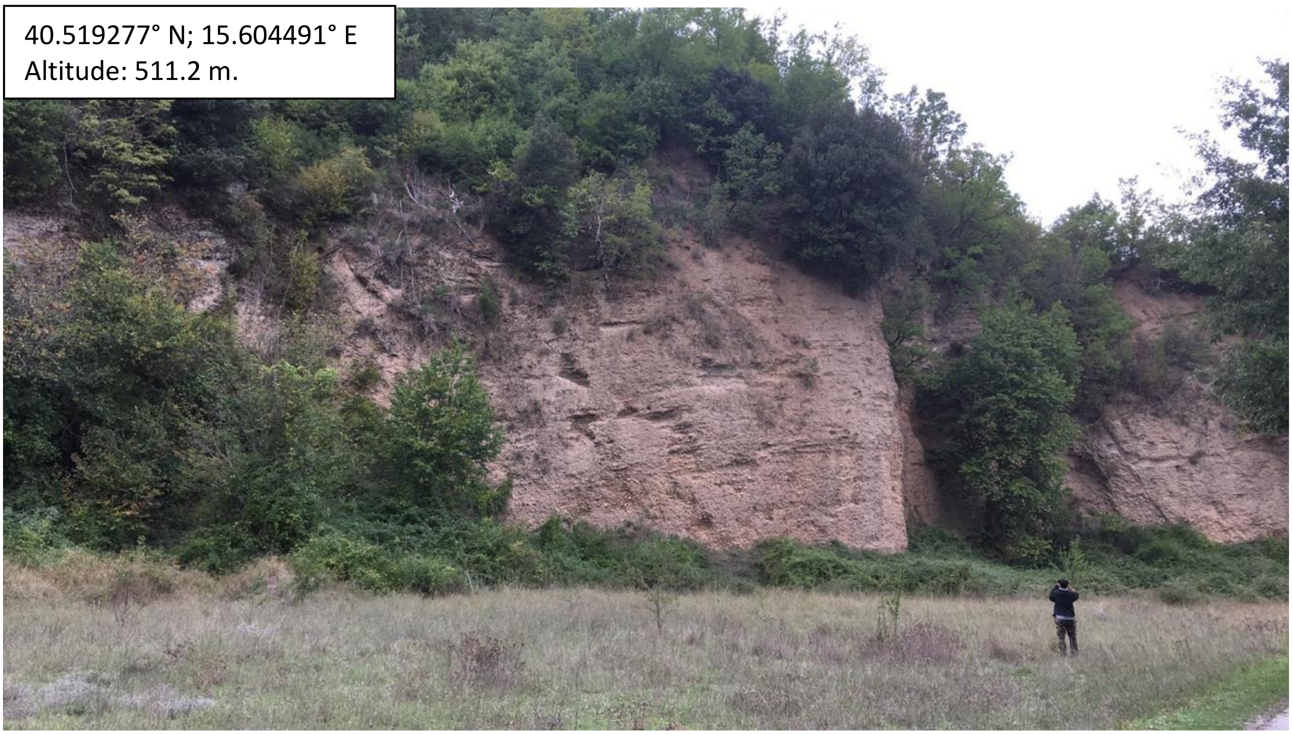

Figure 175.

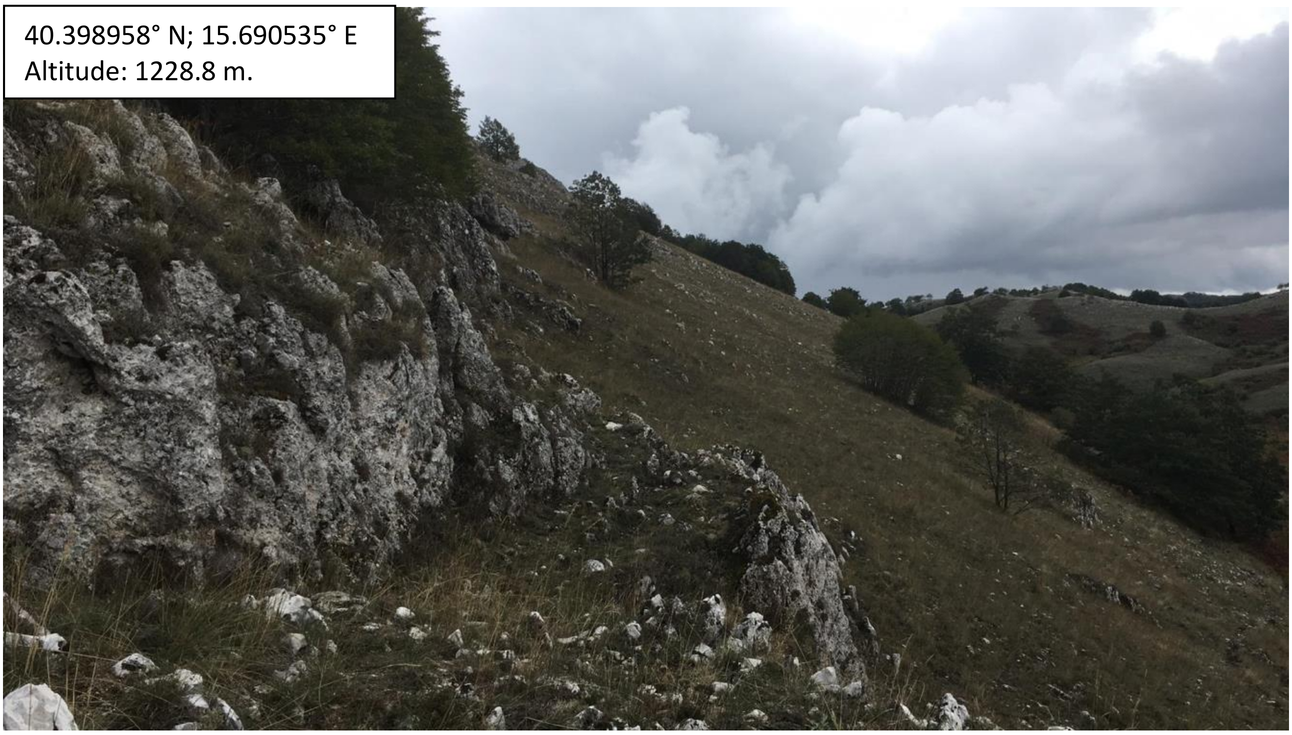

Figure 176.

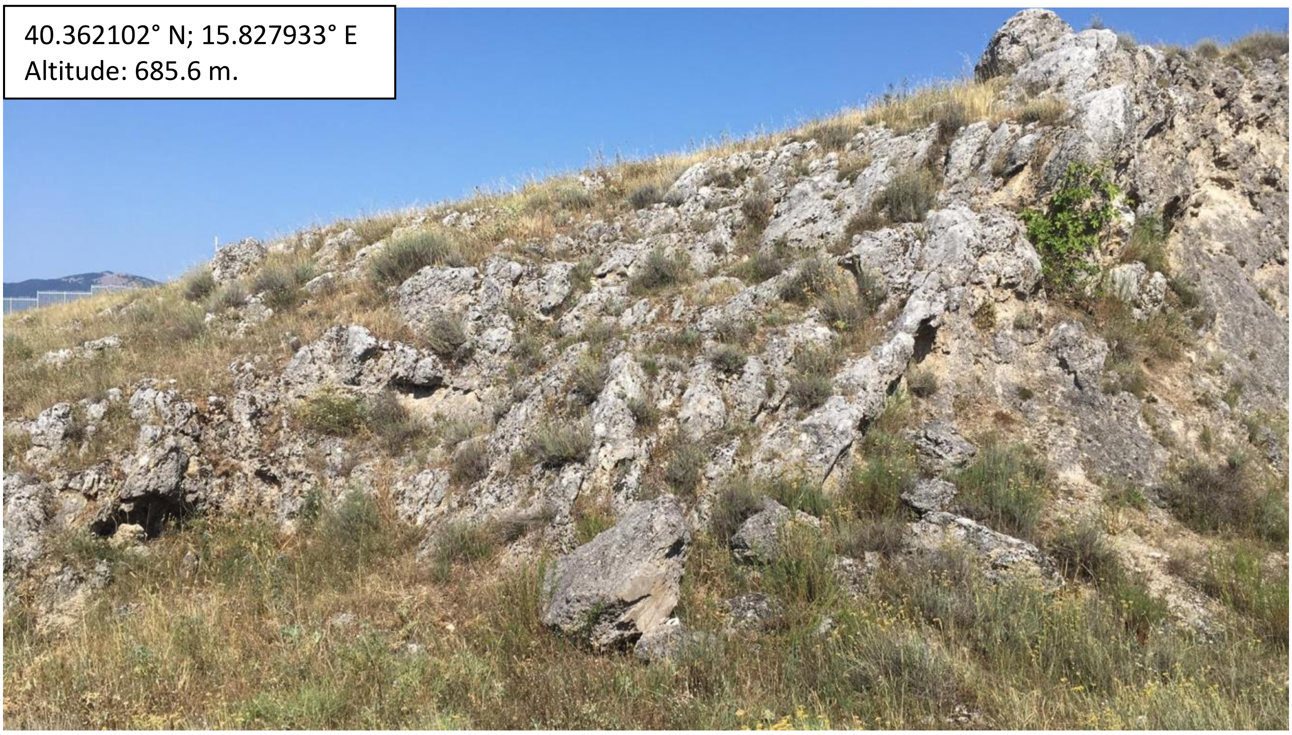

Figure 177.

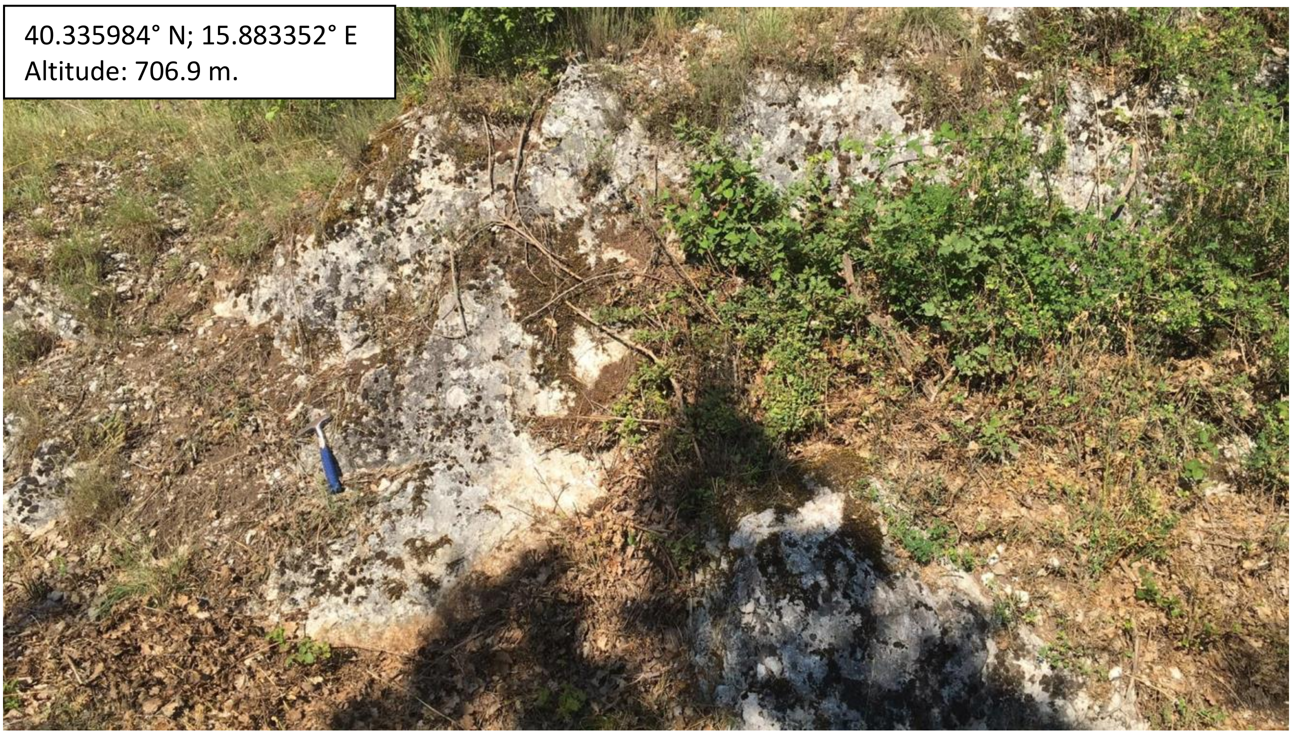

Figure 178.

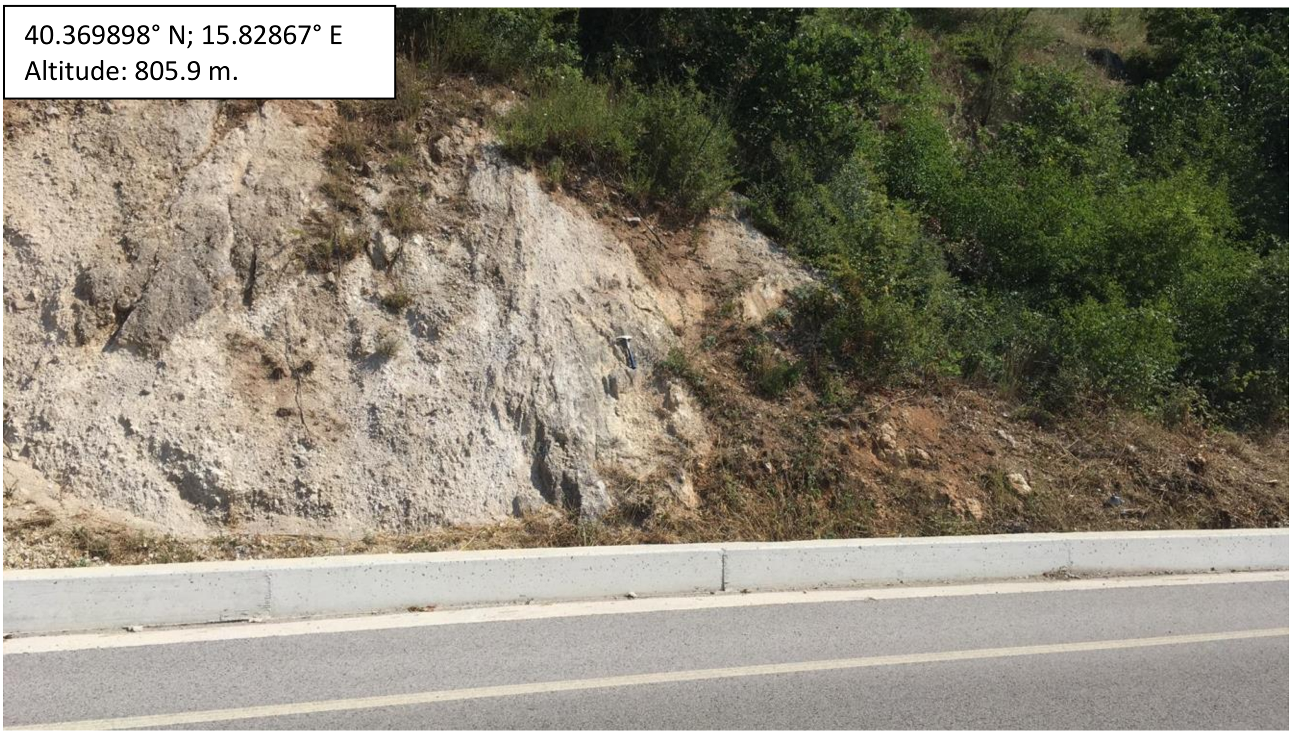

Figure 179.

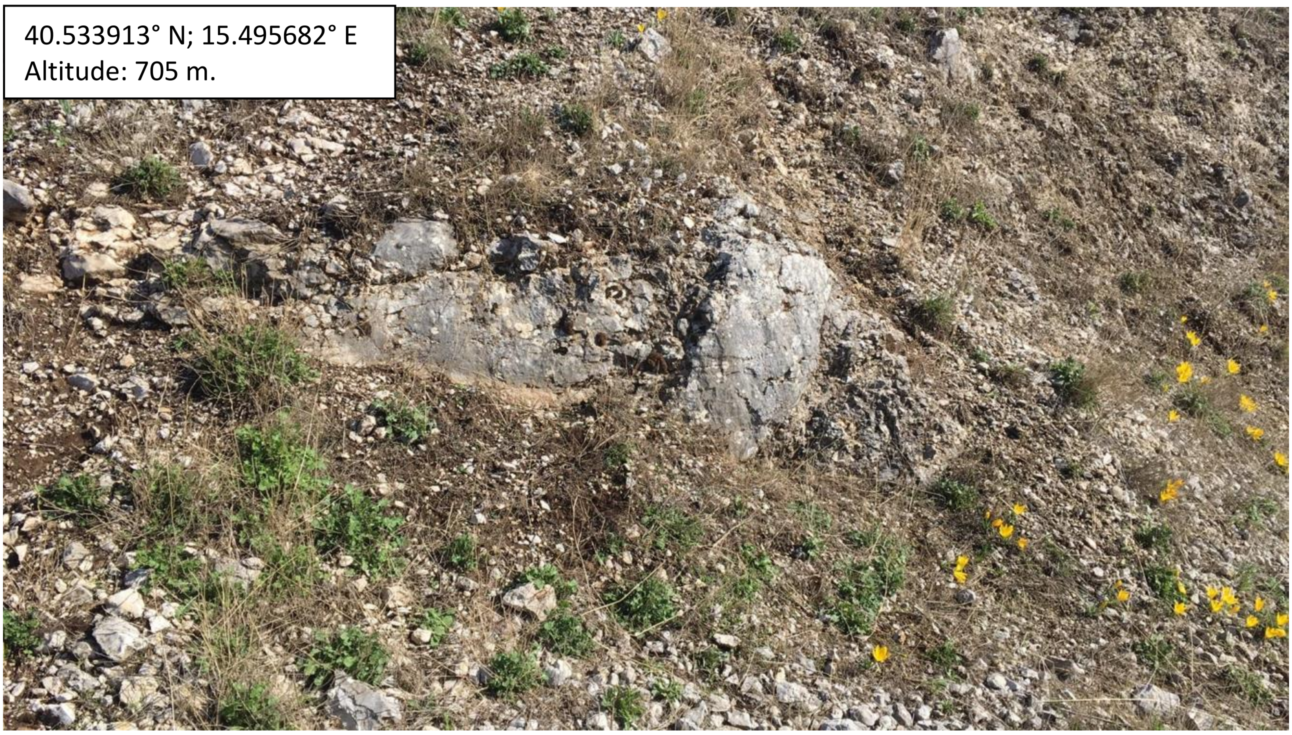

Figure 180.

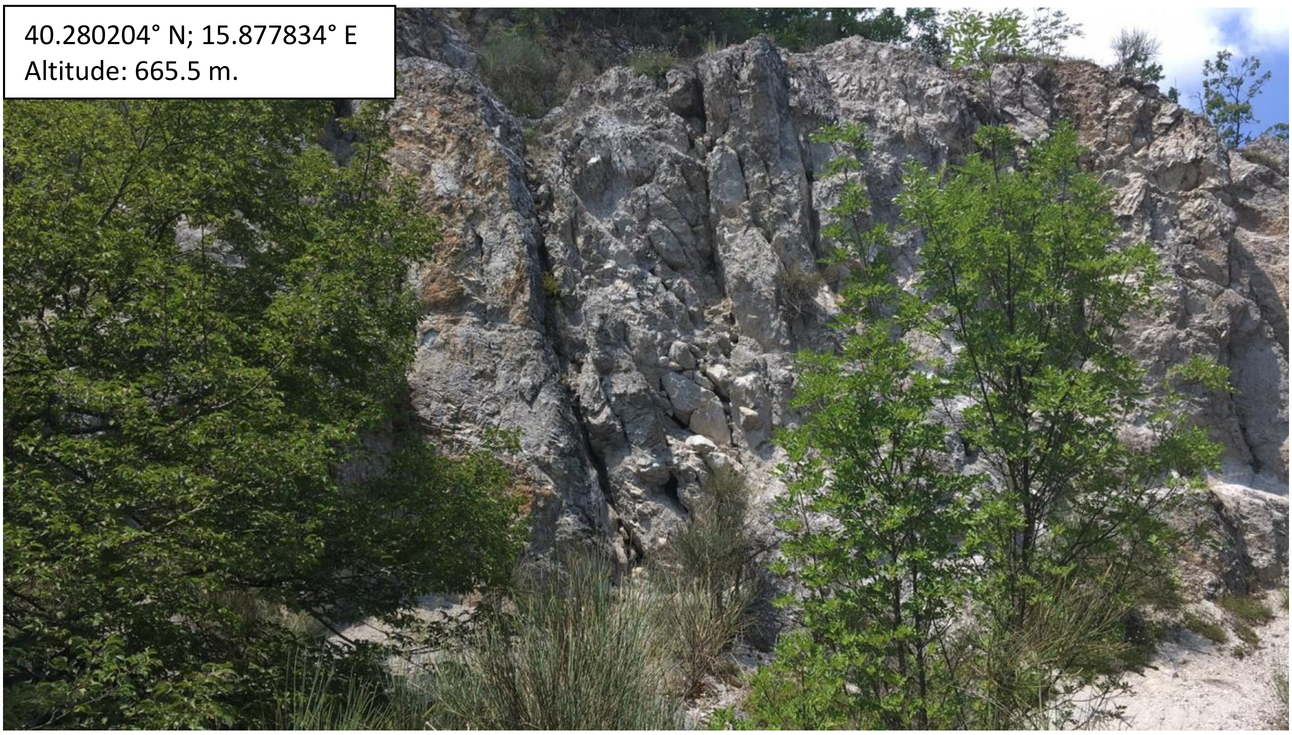

Figure 181.

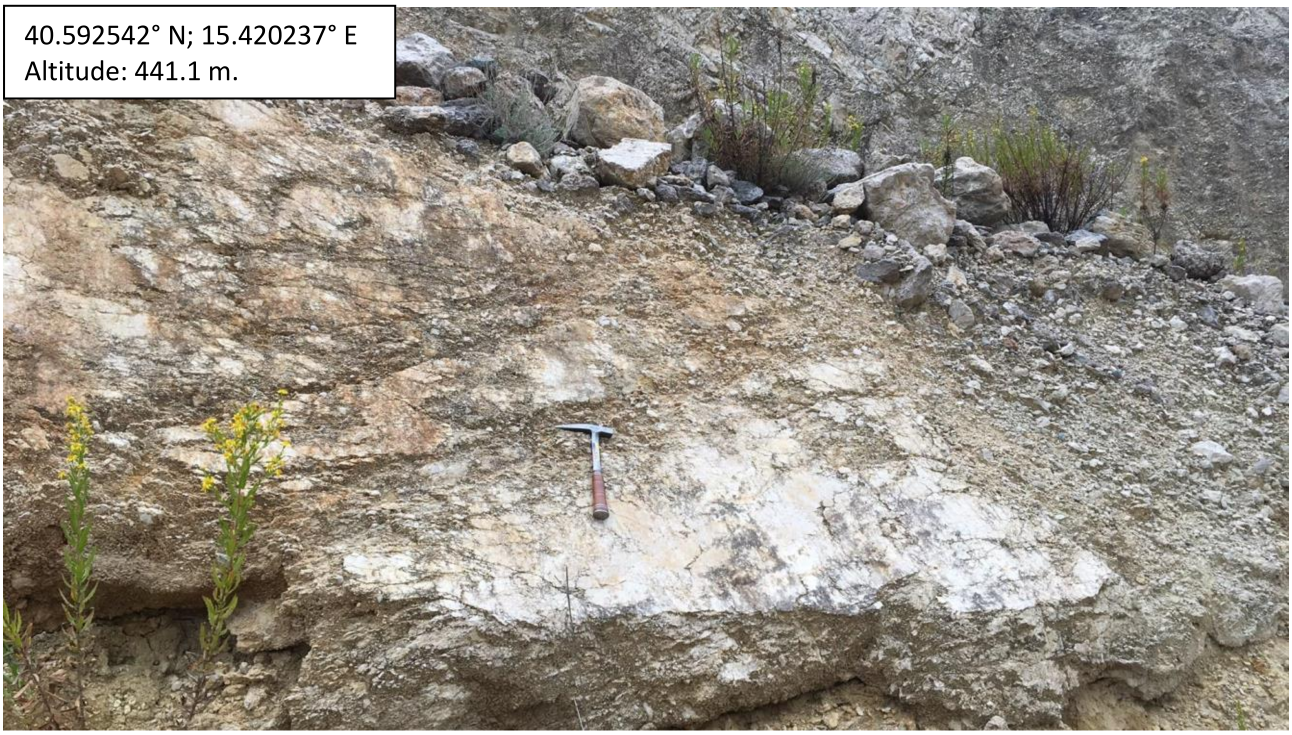

Figure 182.

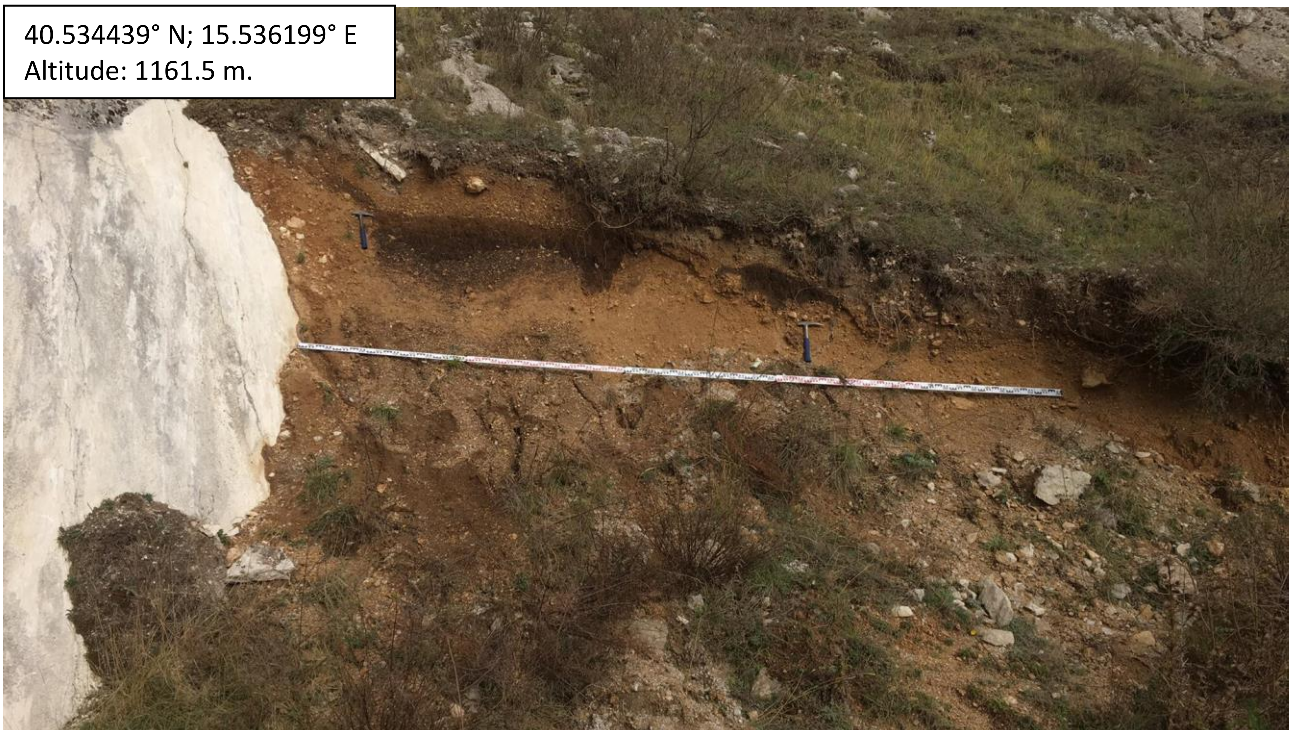

Figure 183.

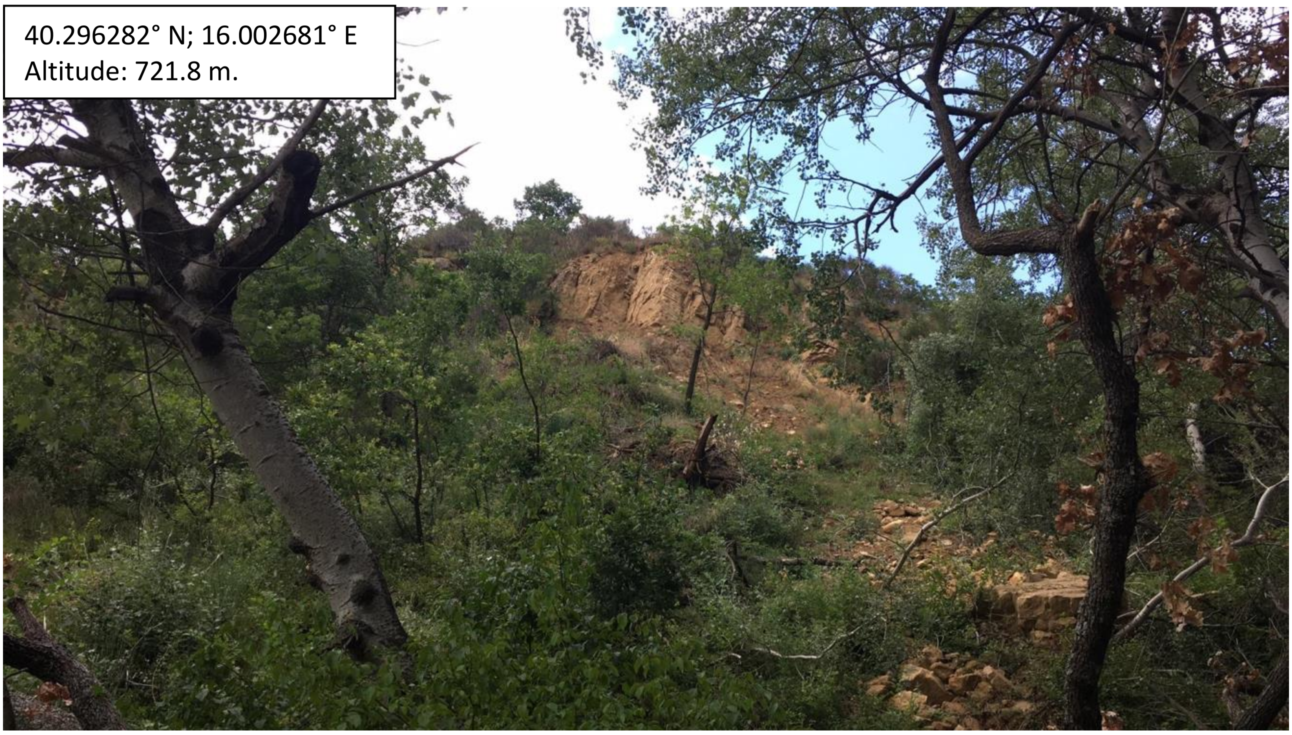

Figure 184.

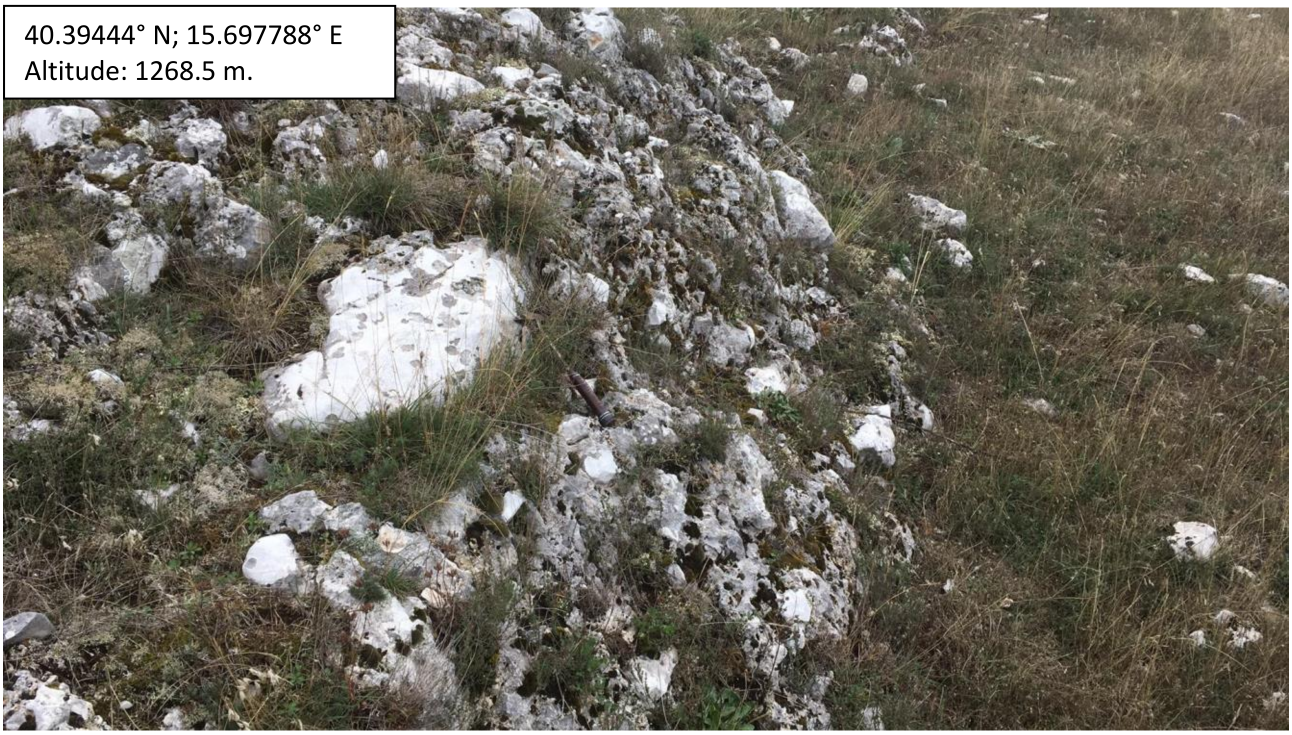

Figure 185.

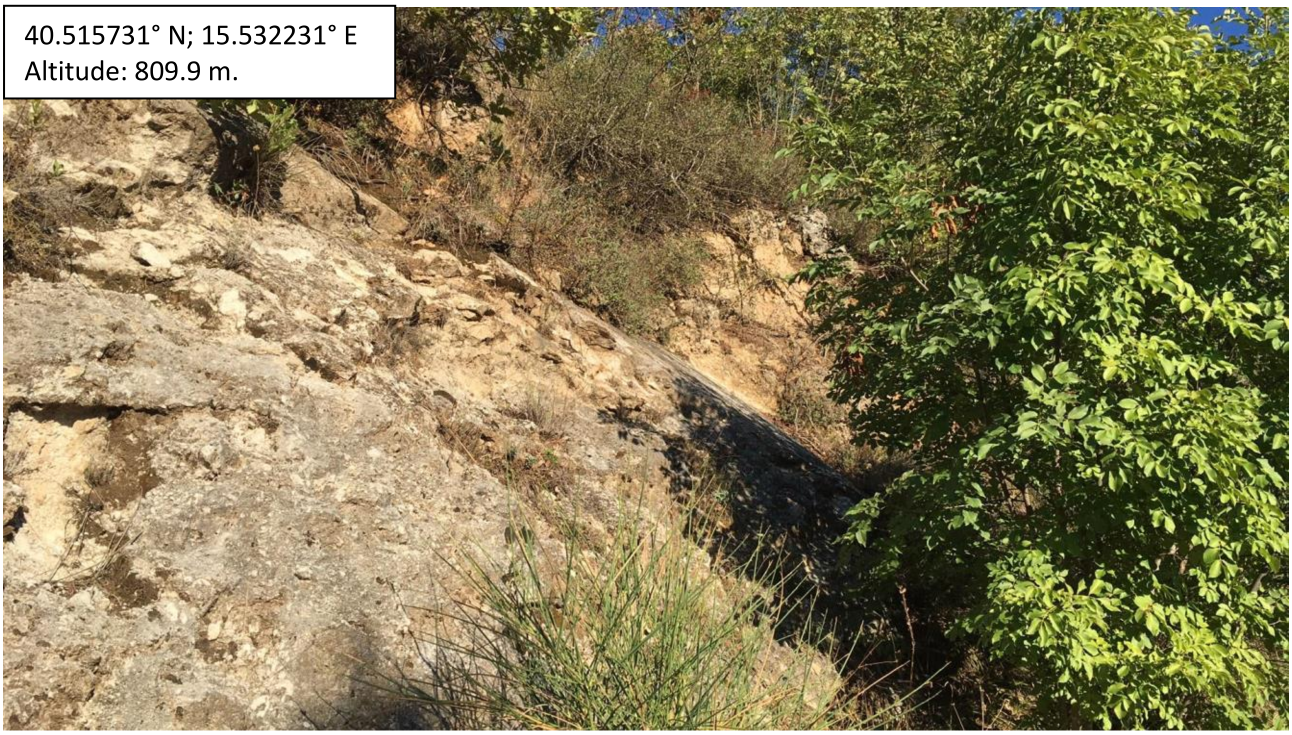

Figure 186.

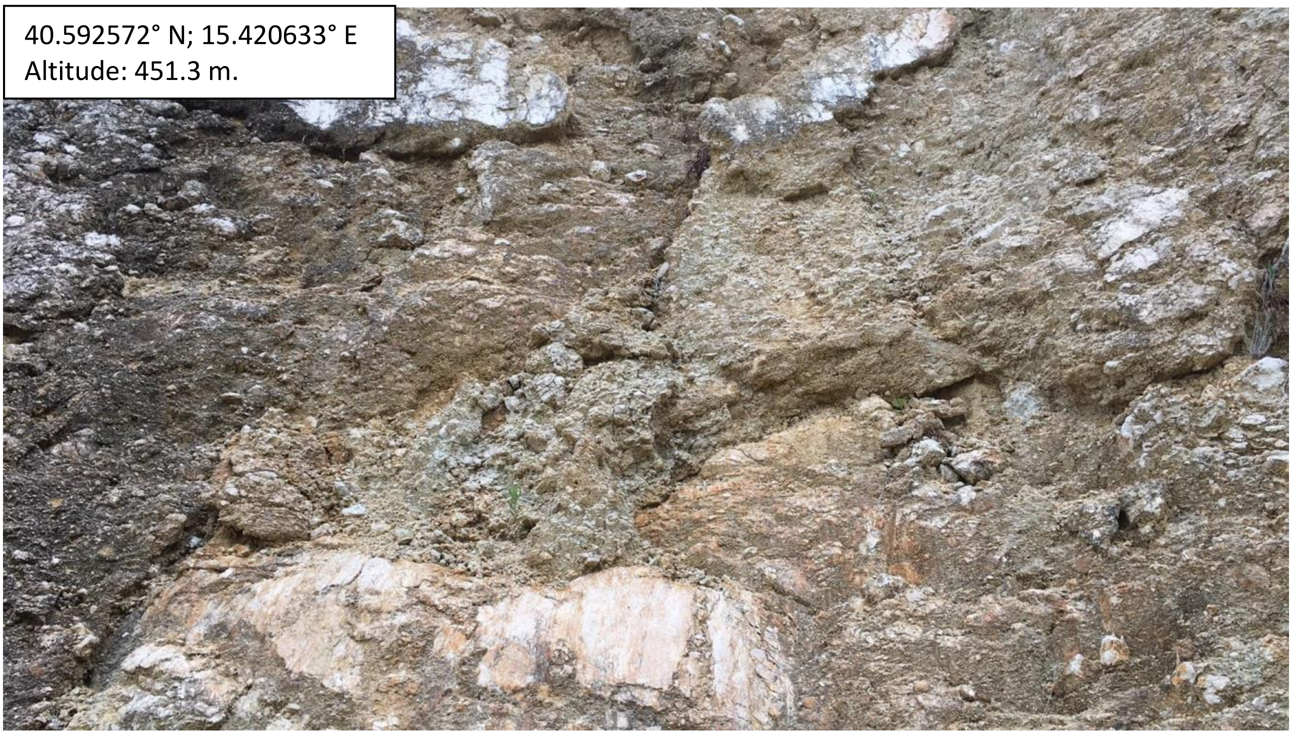

Figure 187.

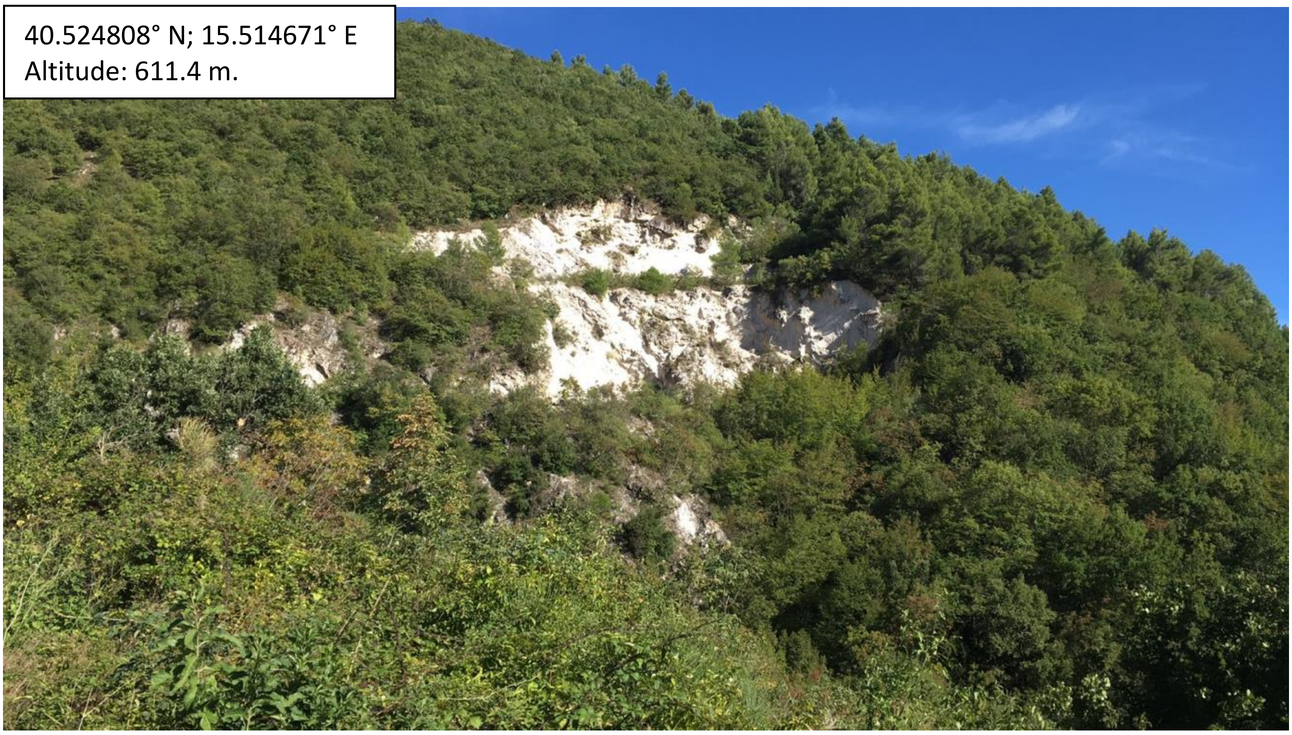

Figure 188.

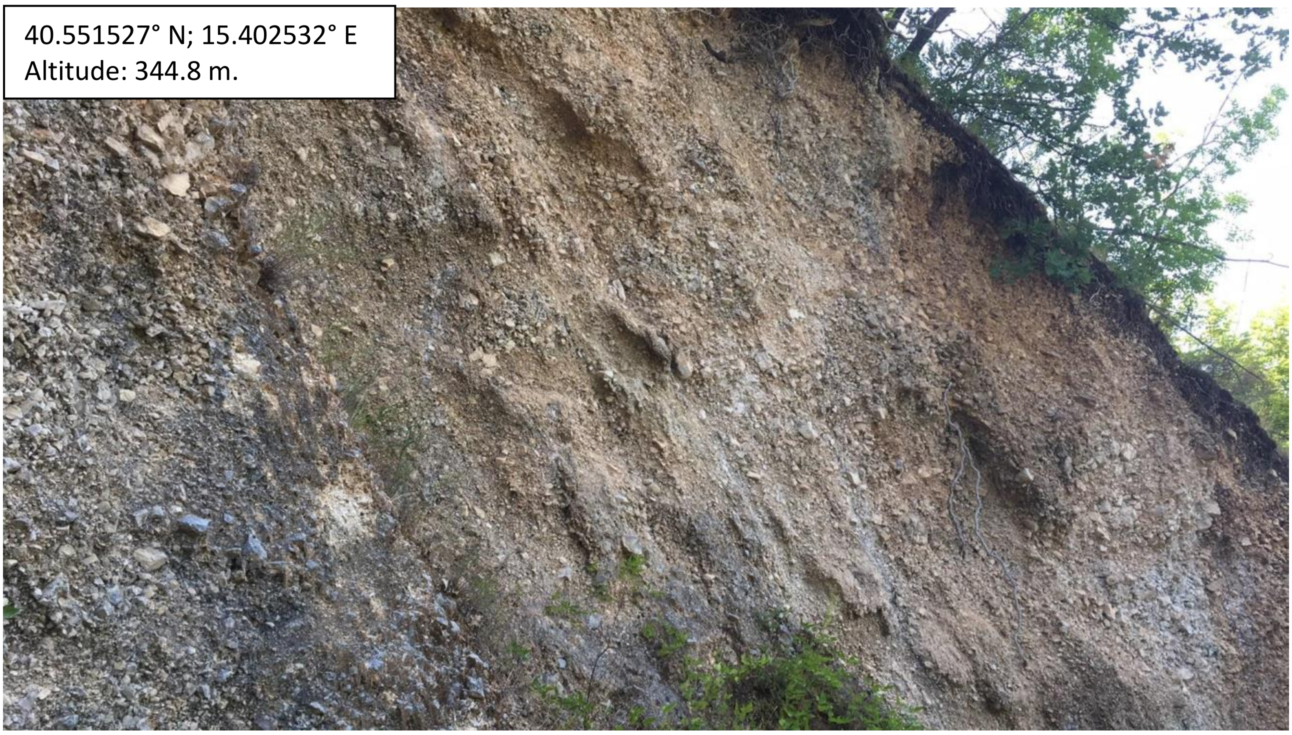

Figure 189.

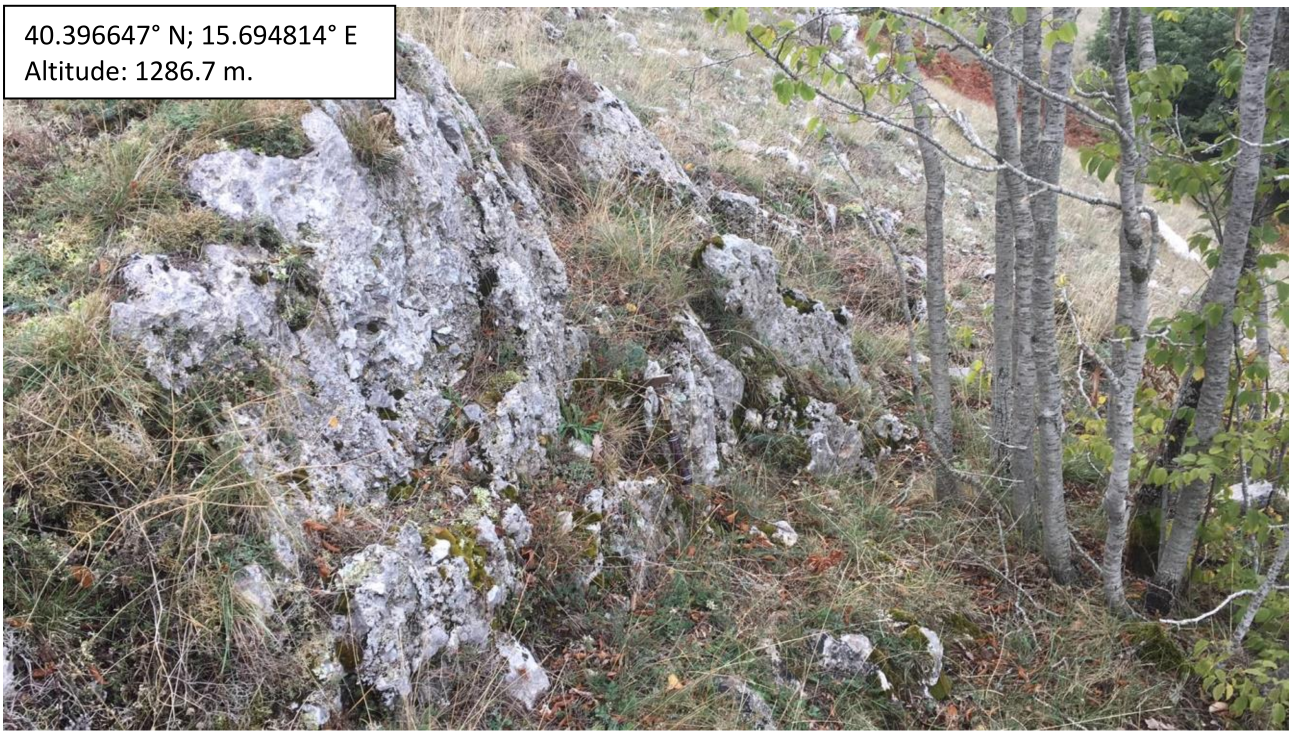

Figure 190.

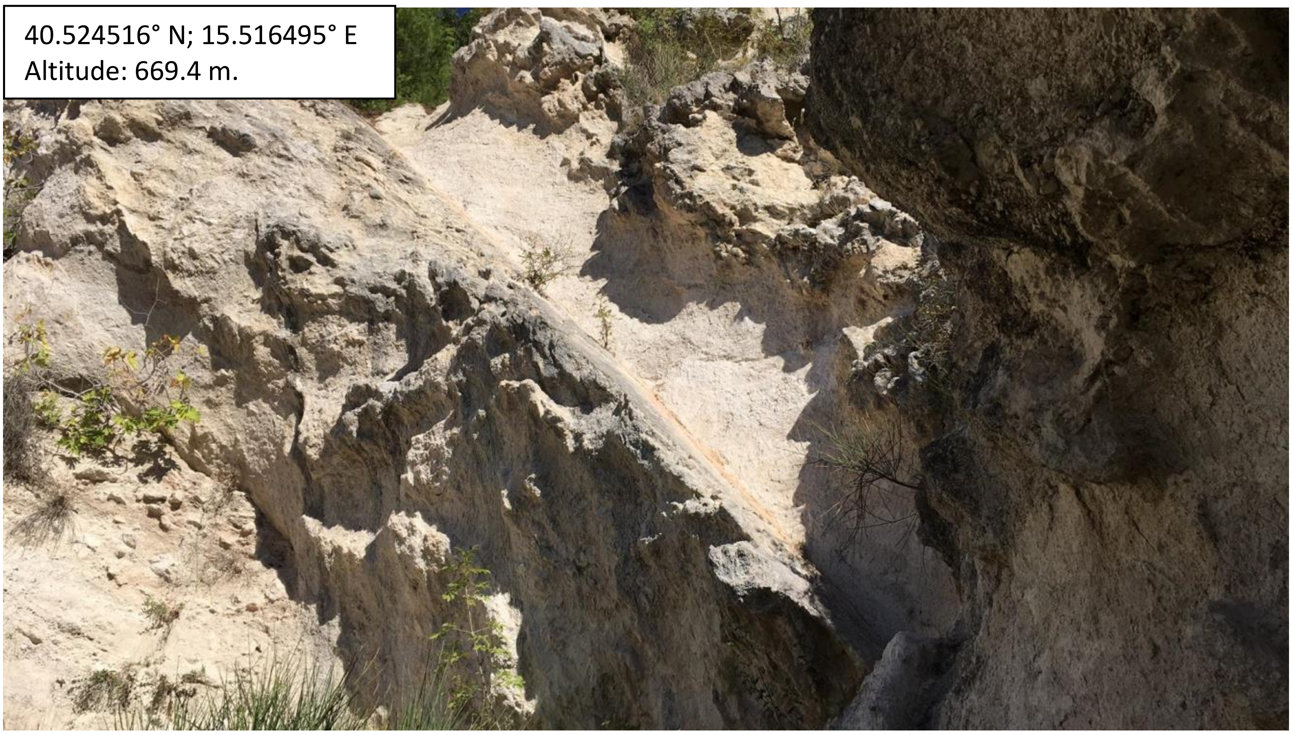

Figure 191.

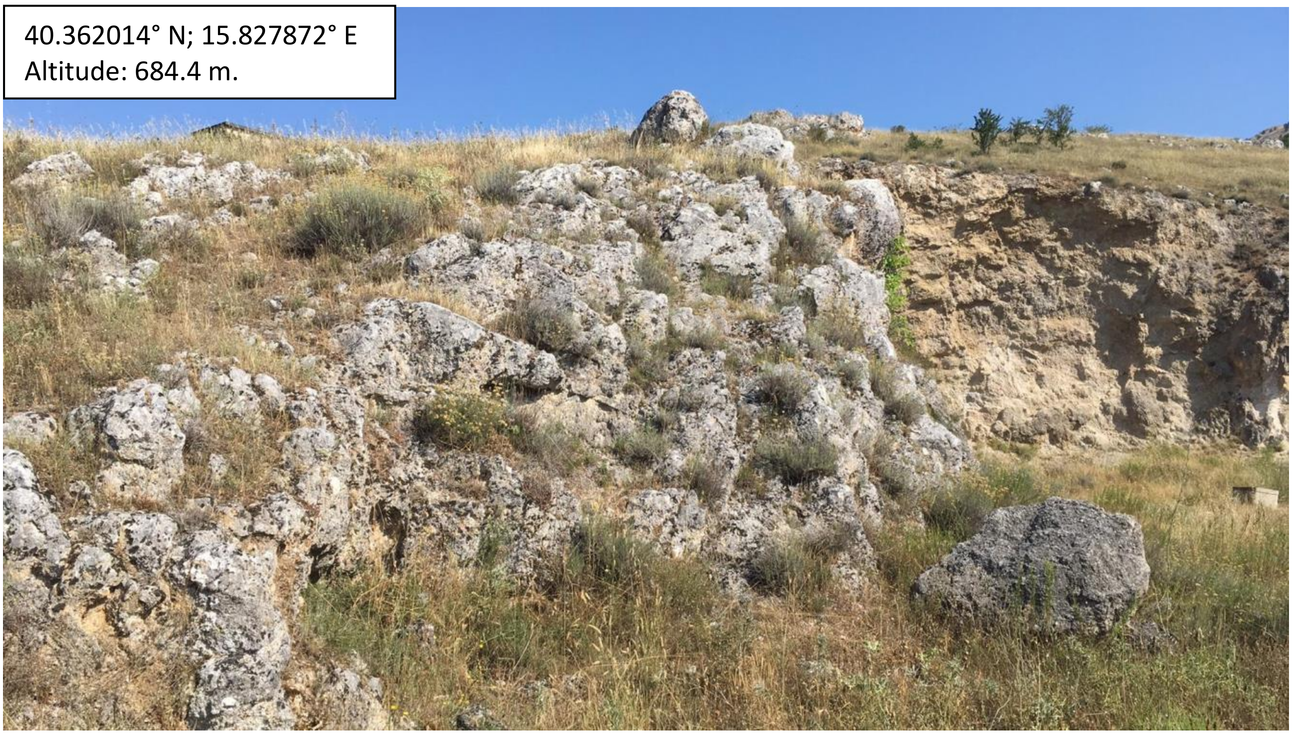

Figure 192.

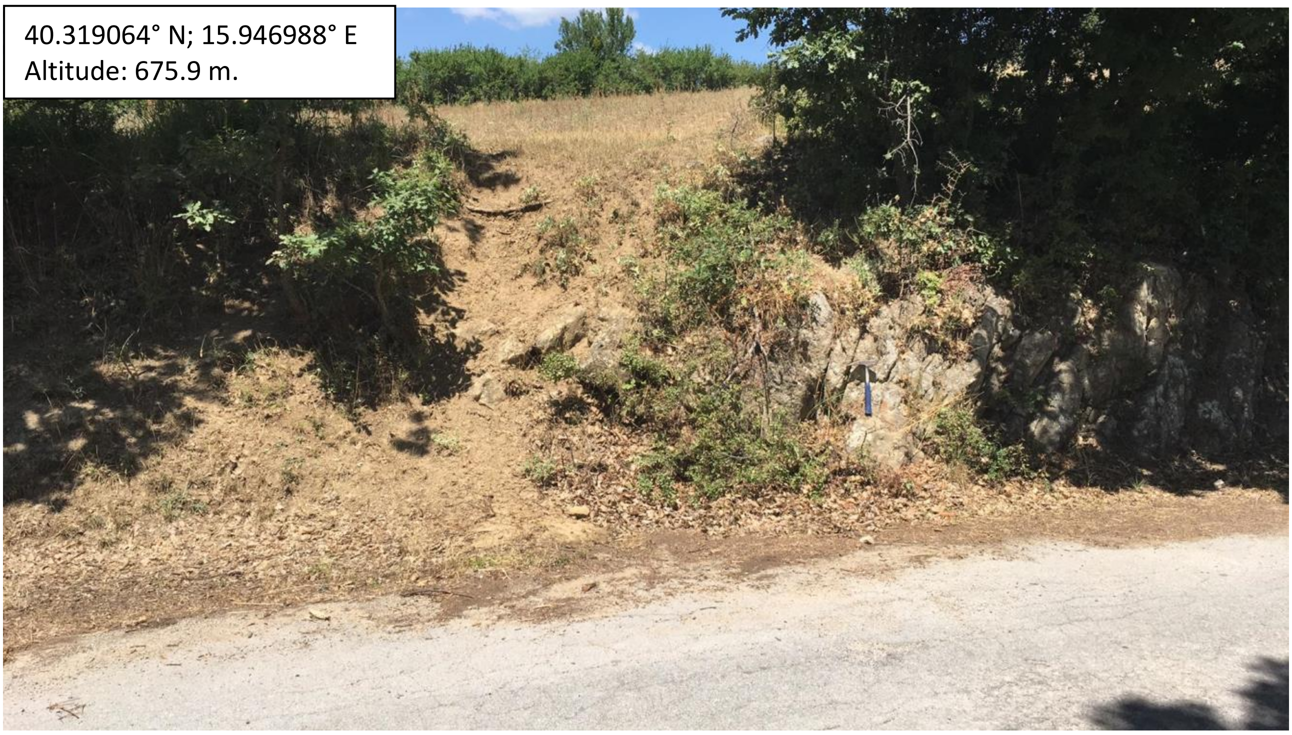

Figure 193.

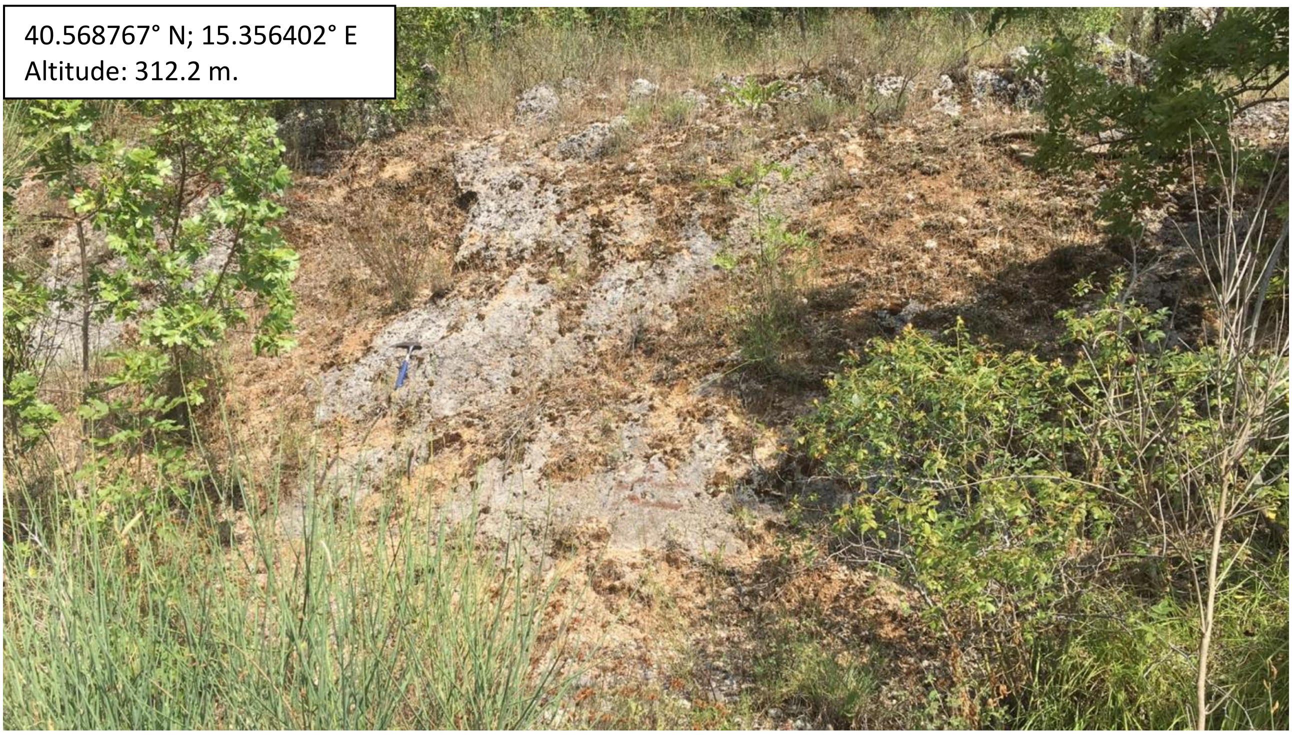

Figure 194.

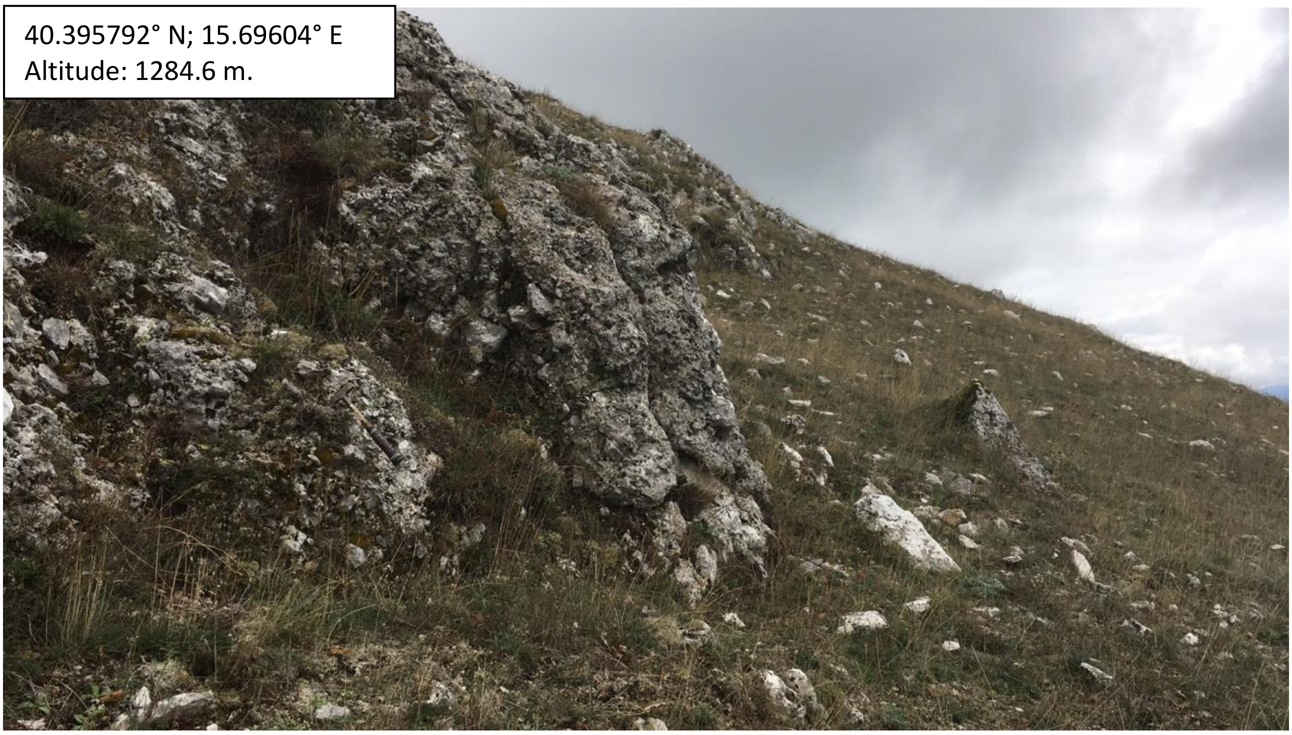

Figure 195.

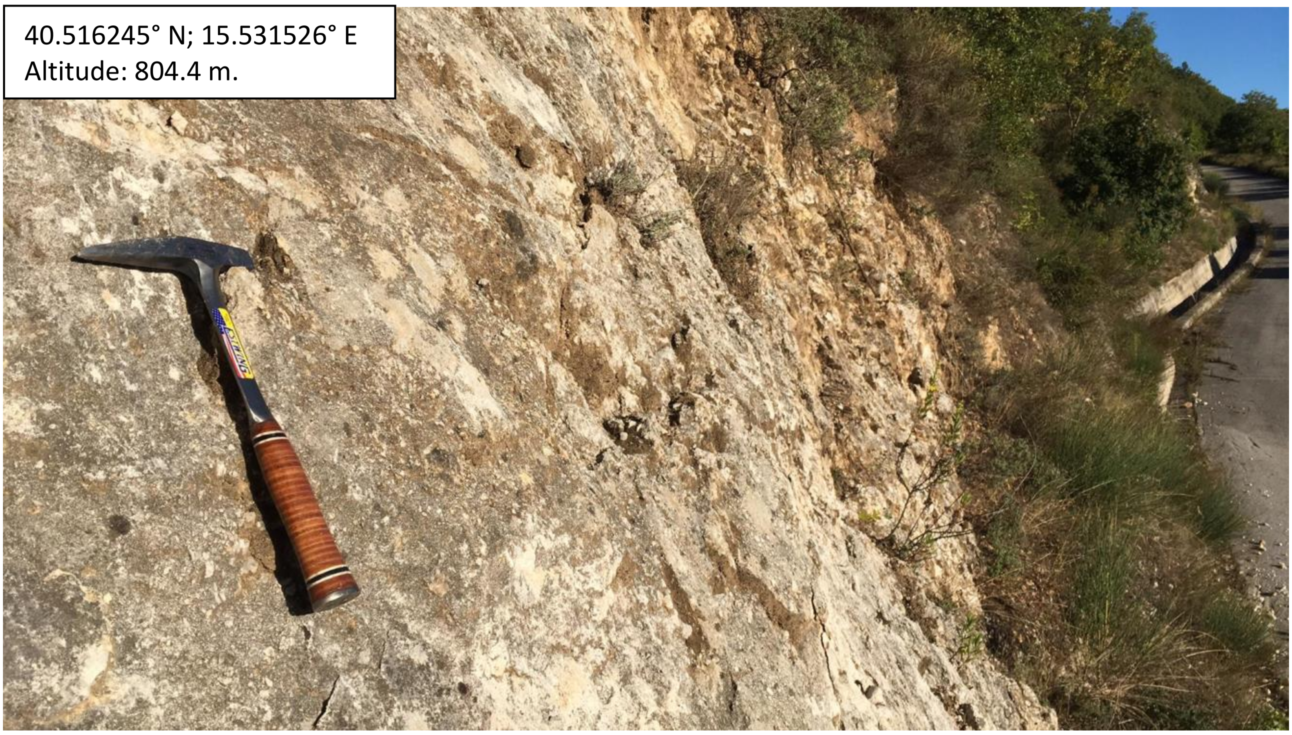

Figure 196.

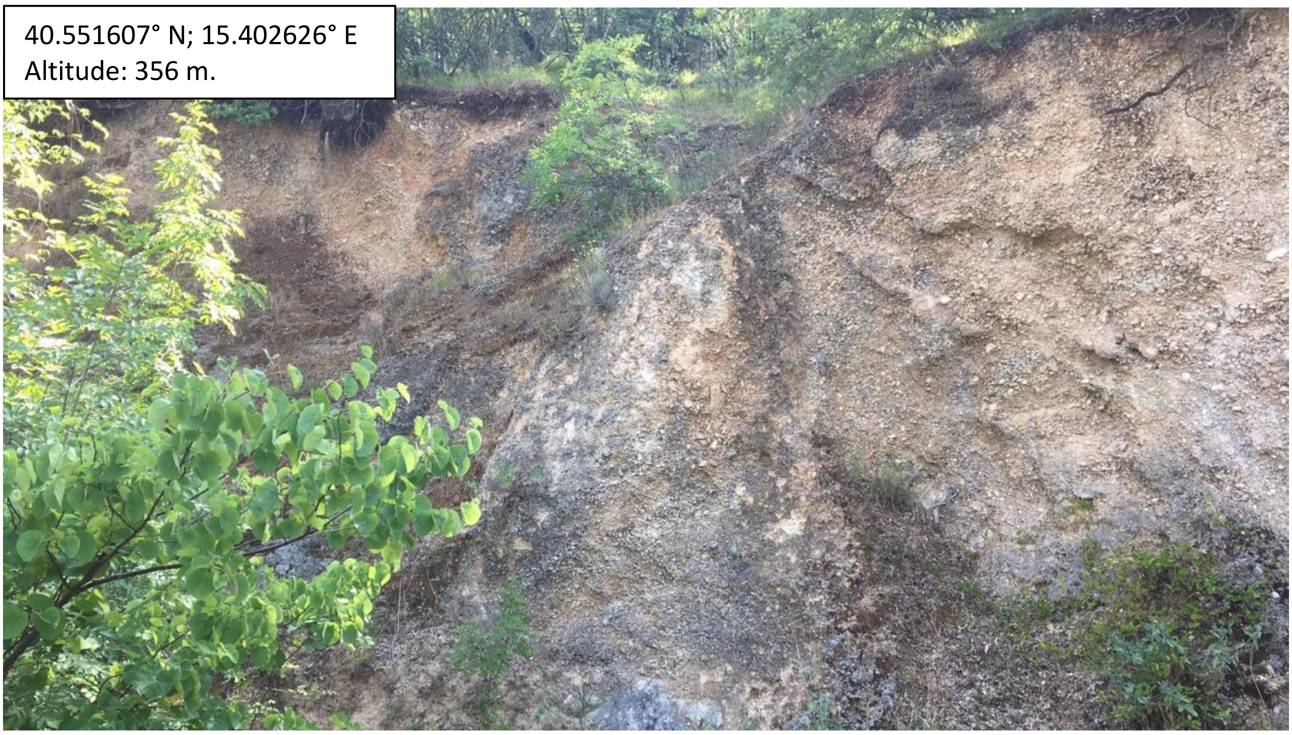

Figure 197.

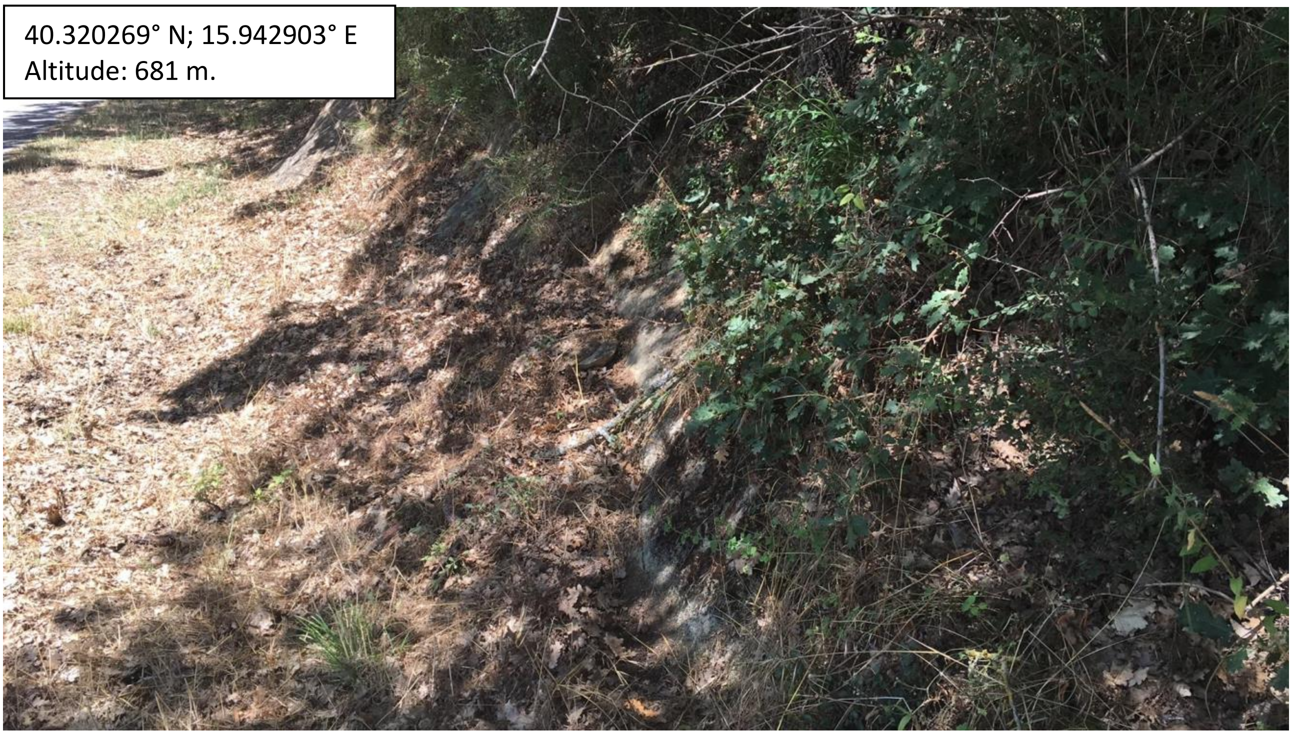

Figure 199.

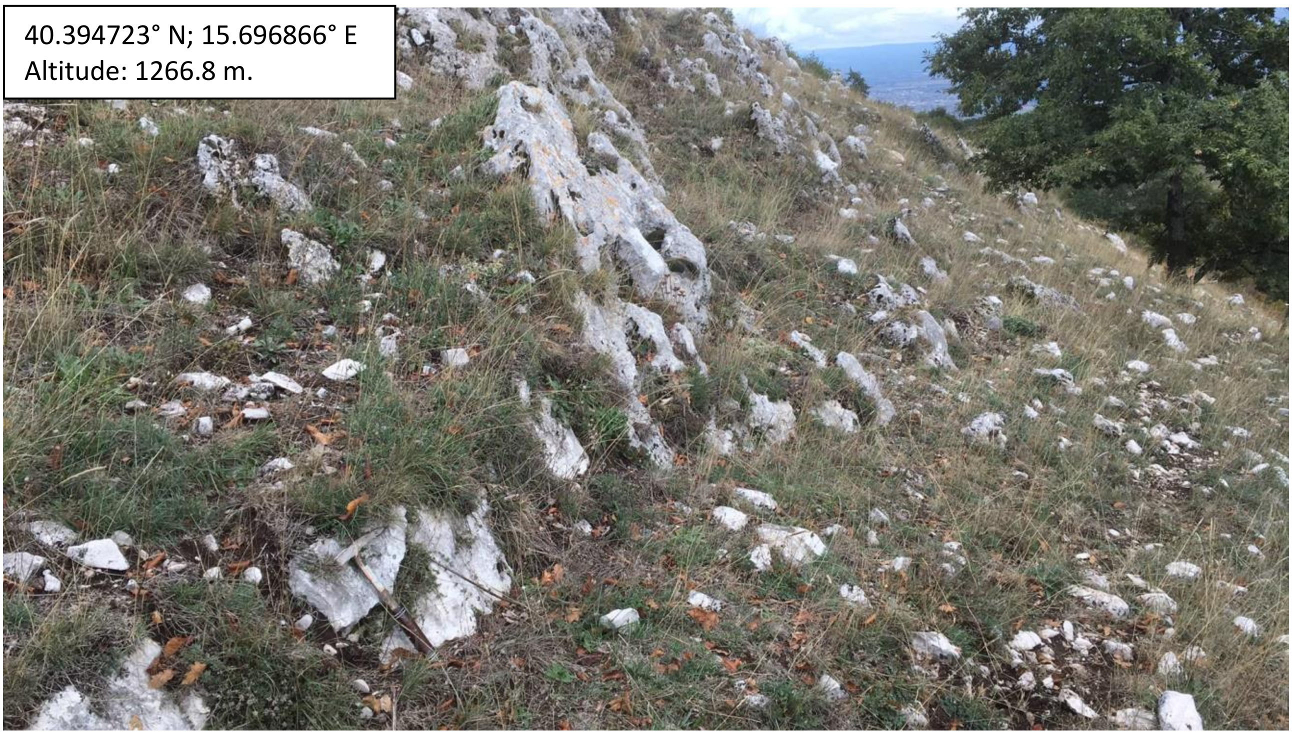

Figure 201.

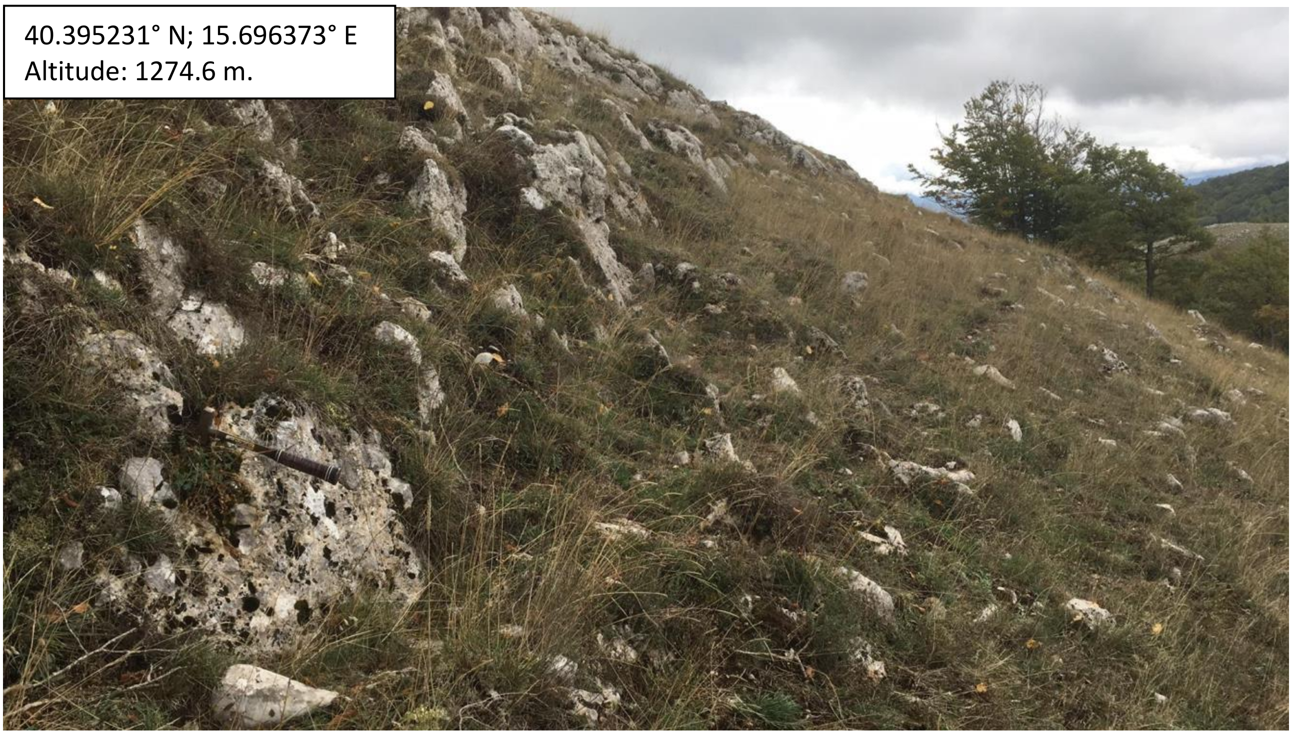

Figure 202.

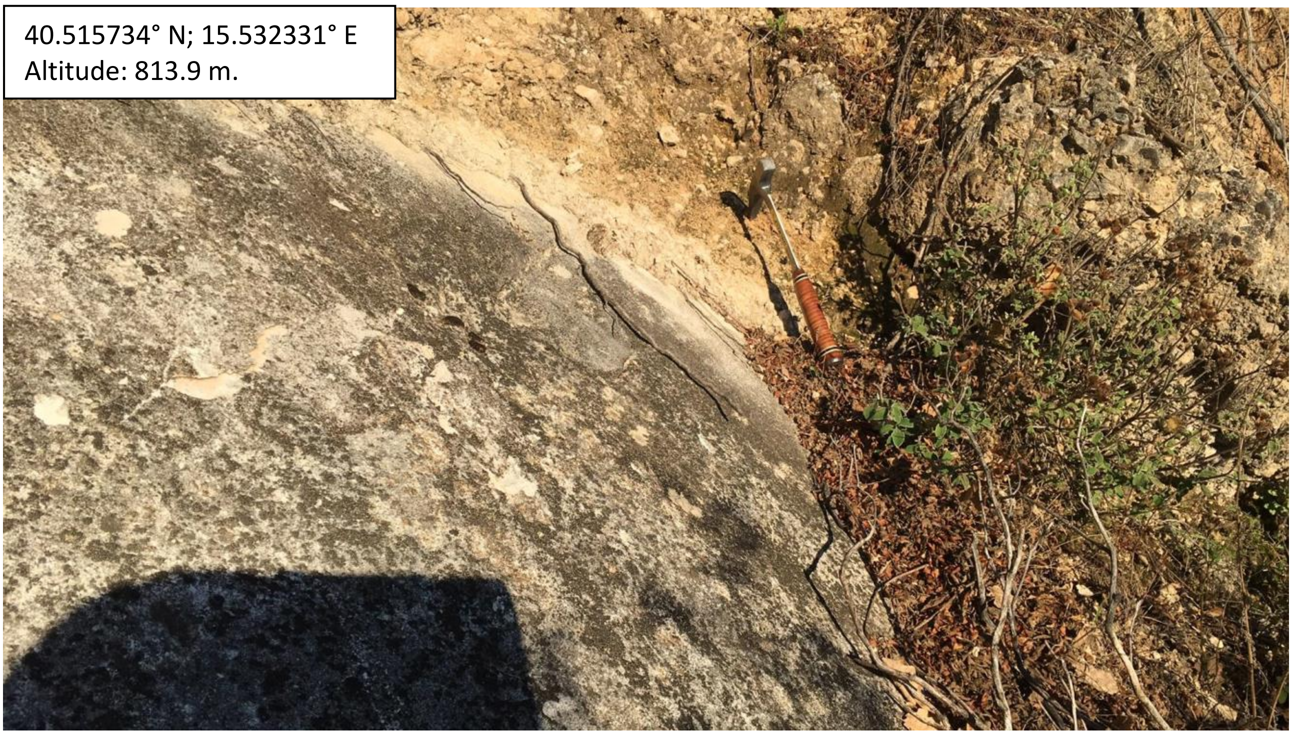

Figure 203.

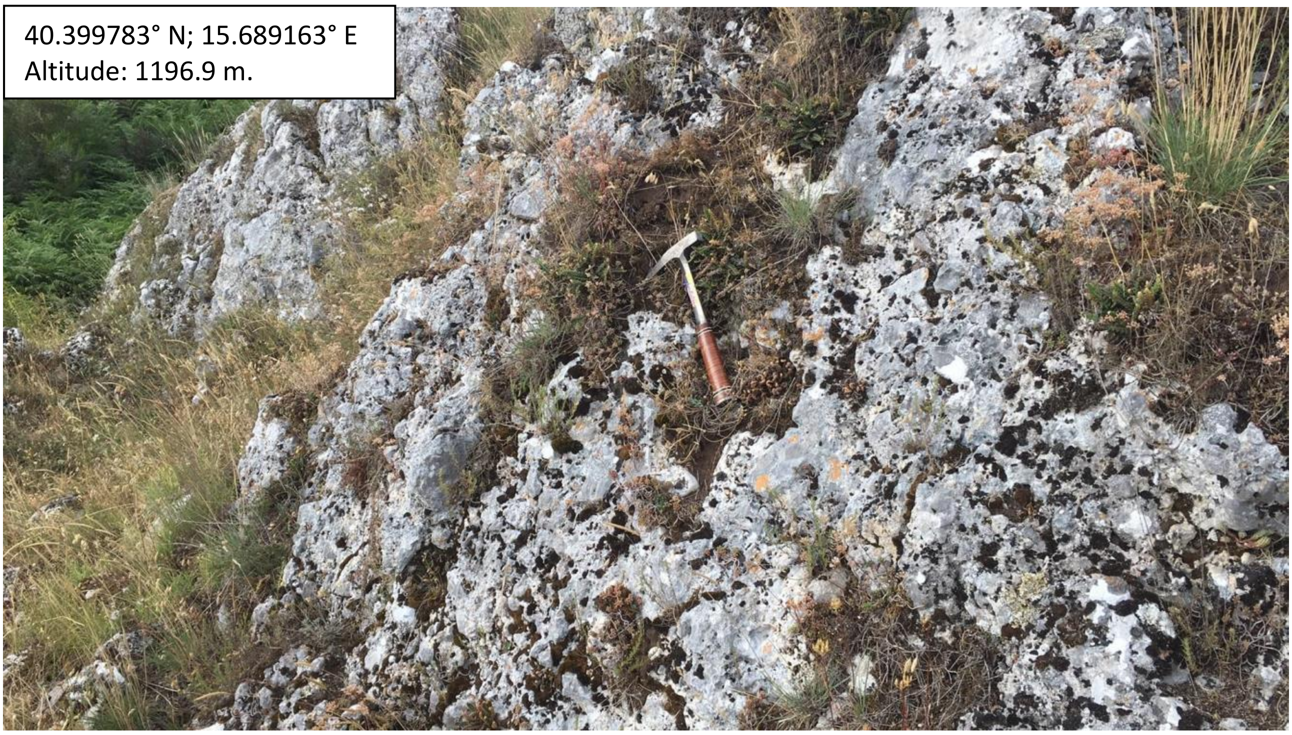

Figure 204.

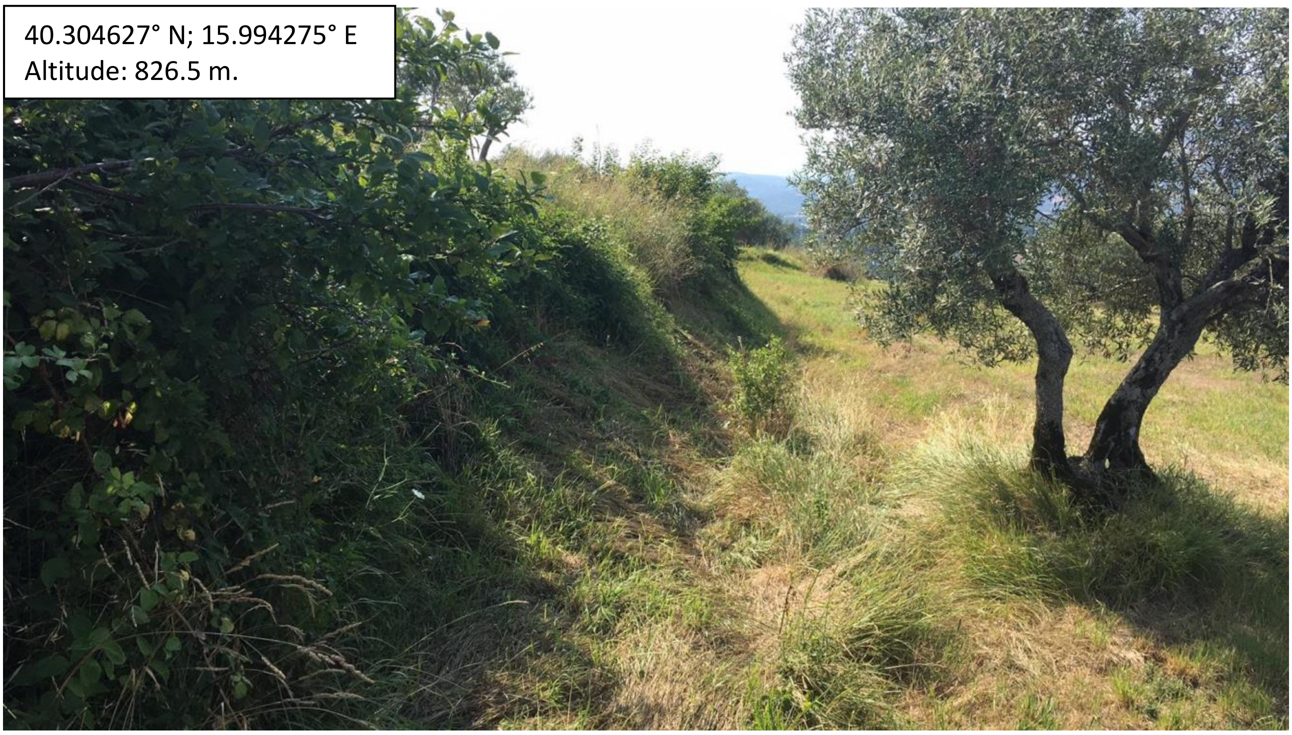

Figure 205.

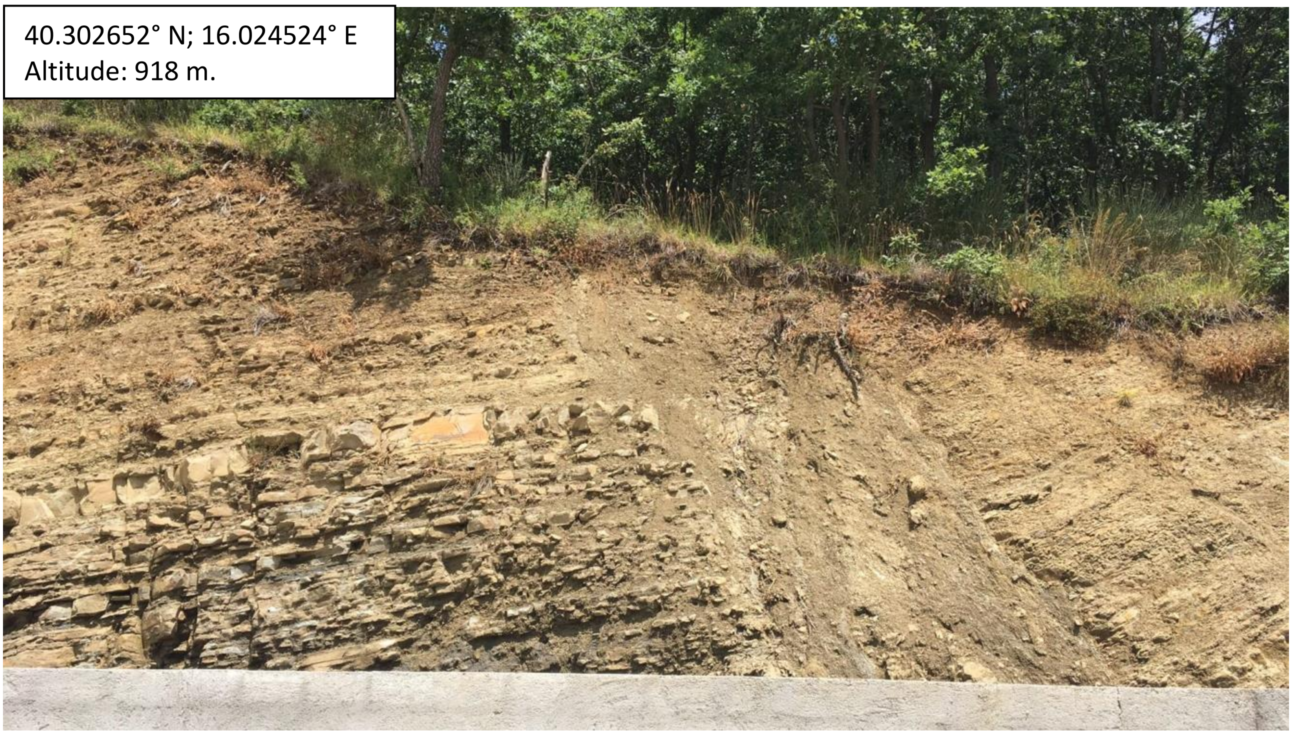

Figure 206.

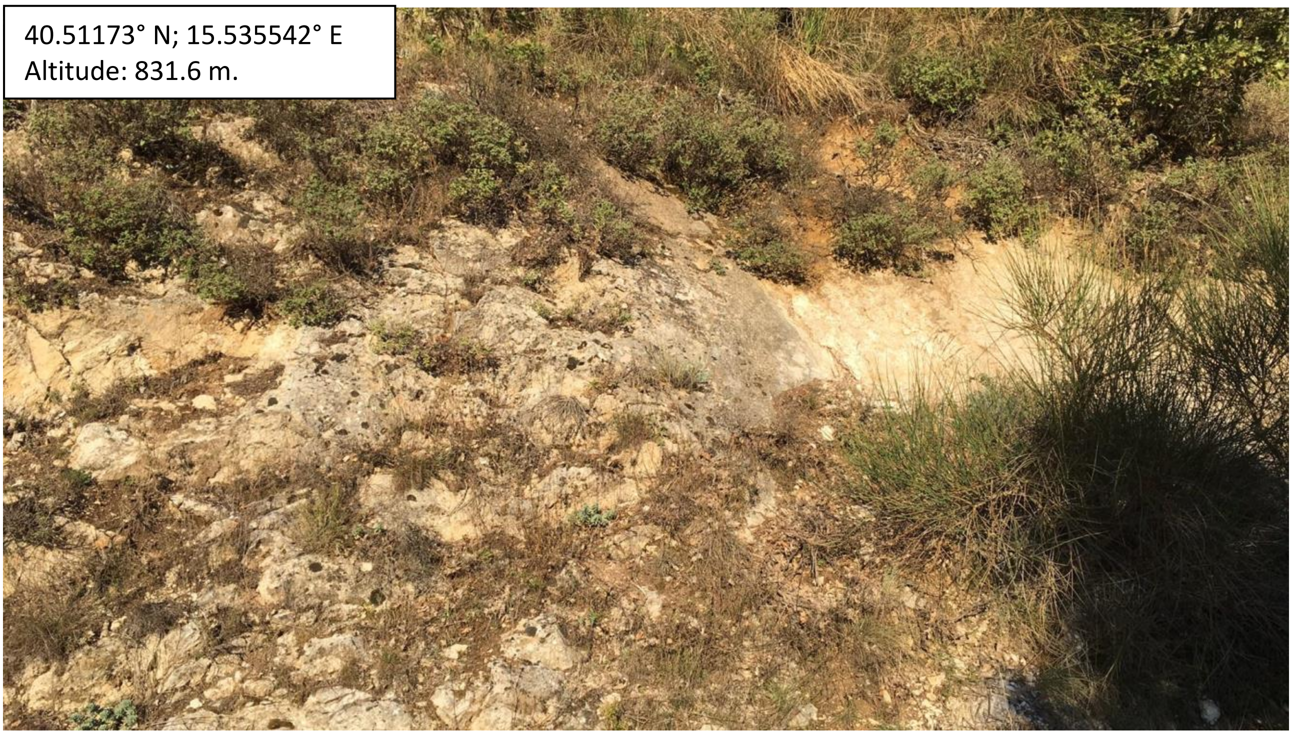

Figure 207.

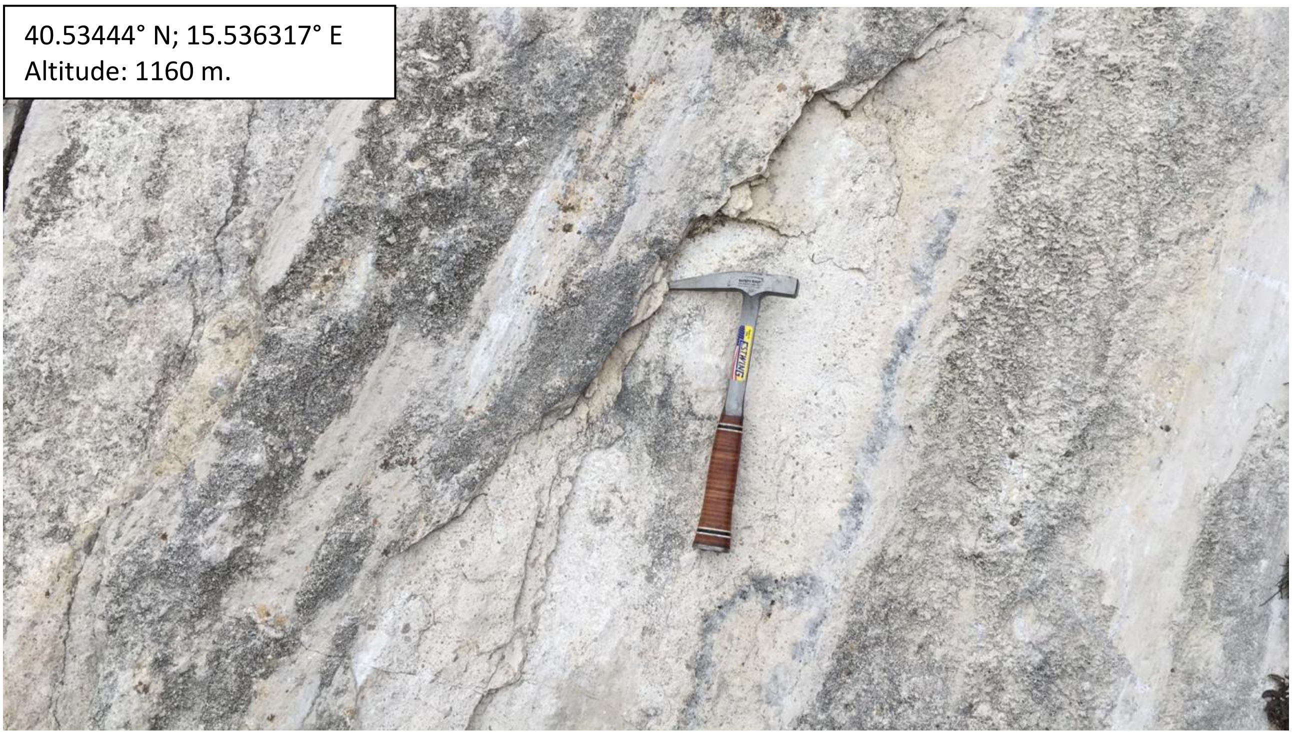

Figure 208.

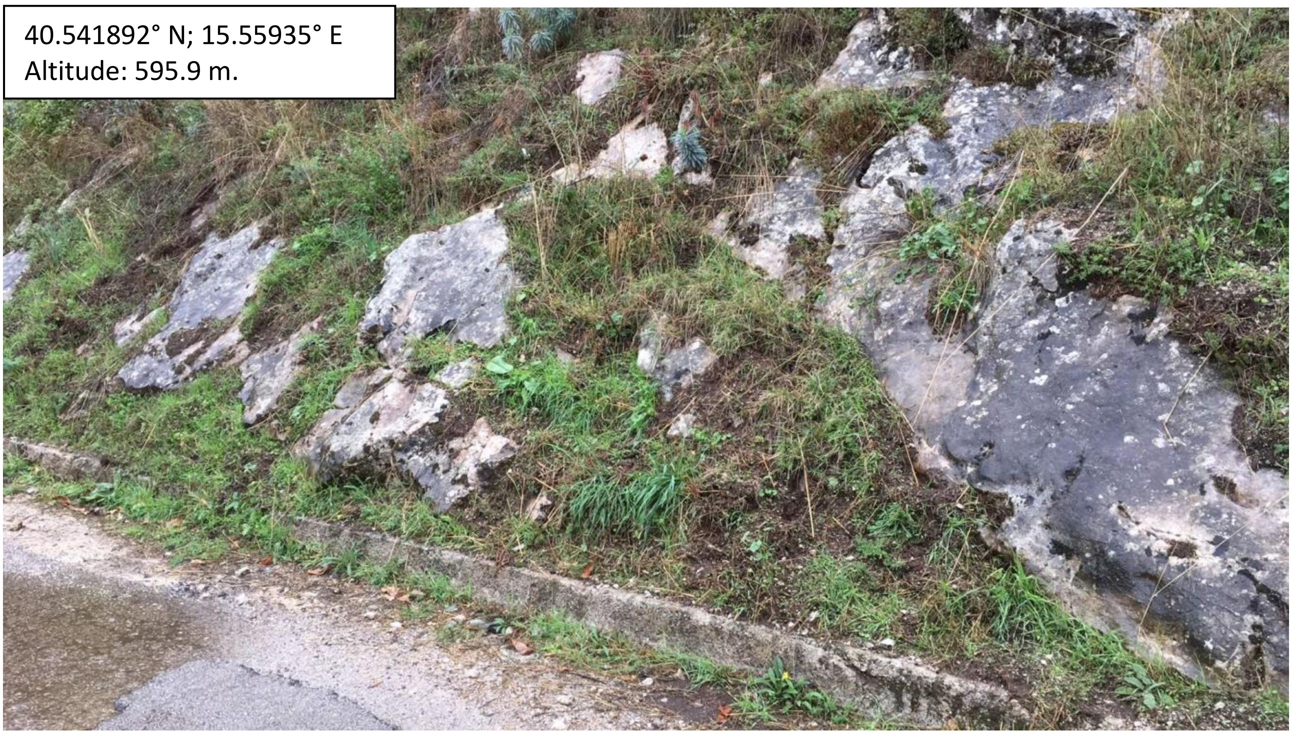

Figure 209.

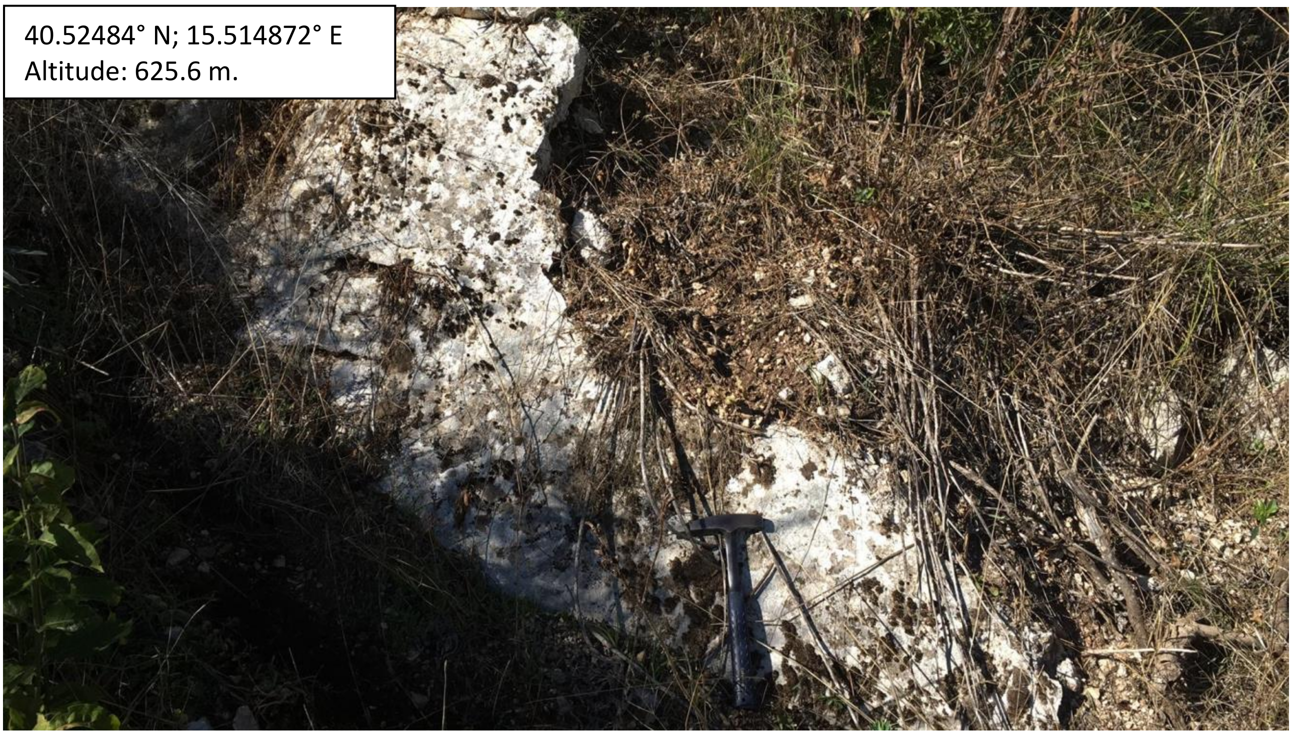

Figure 210.

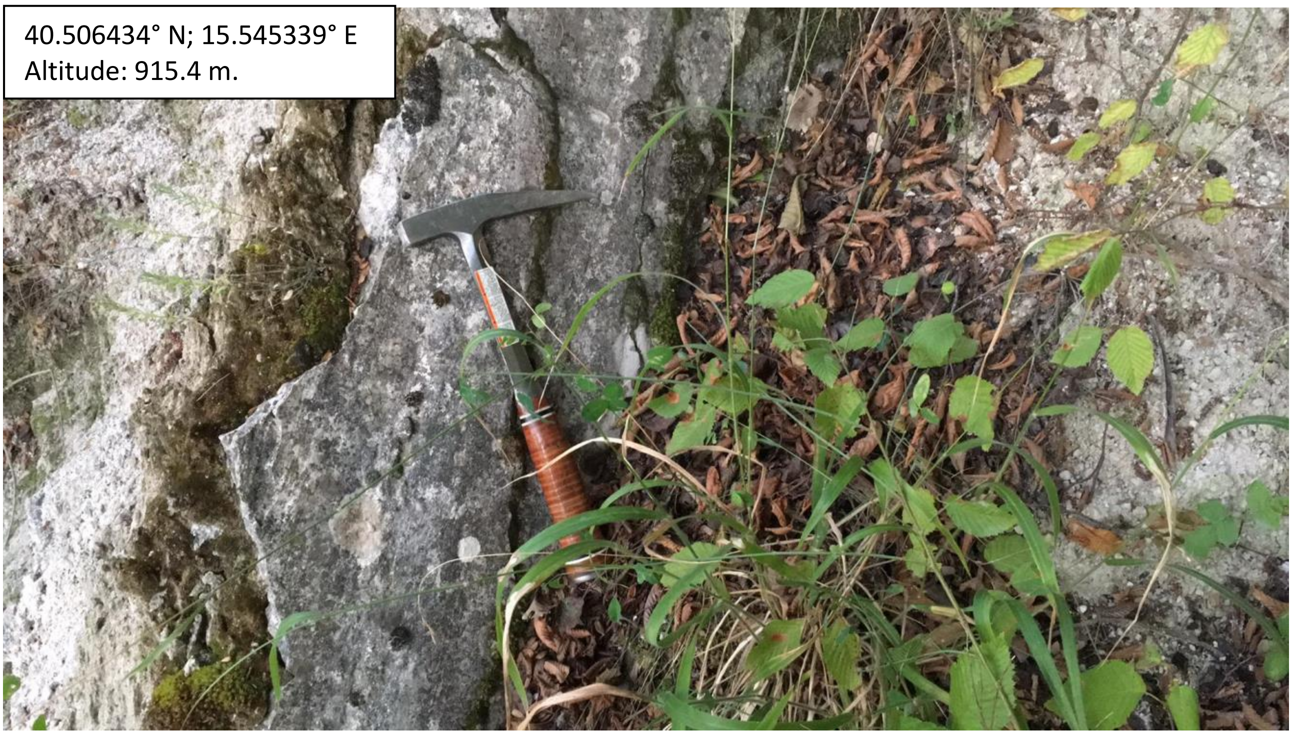

Figure 211.

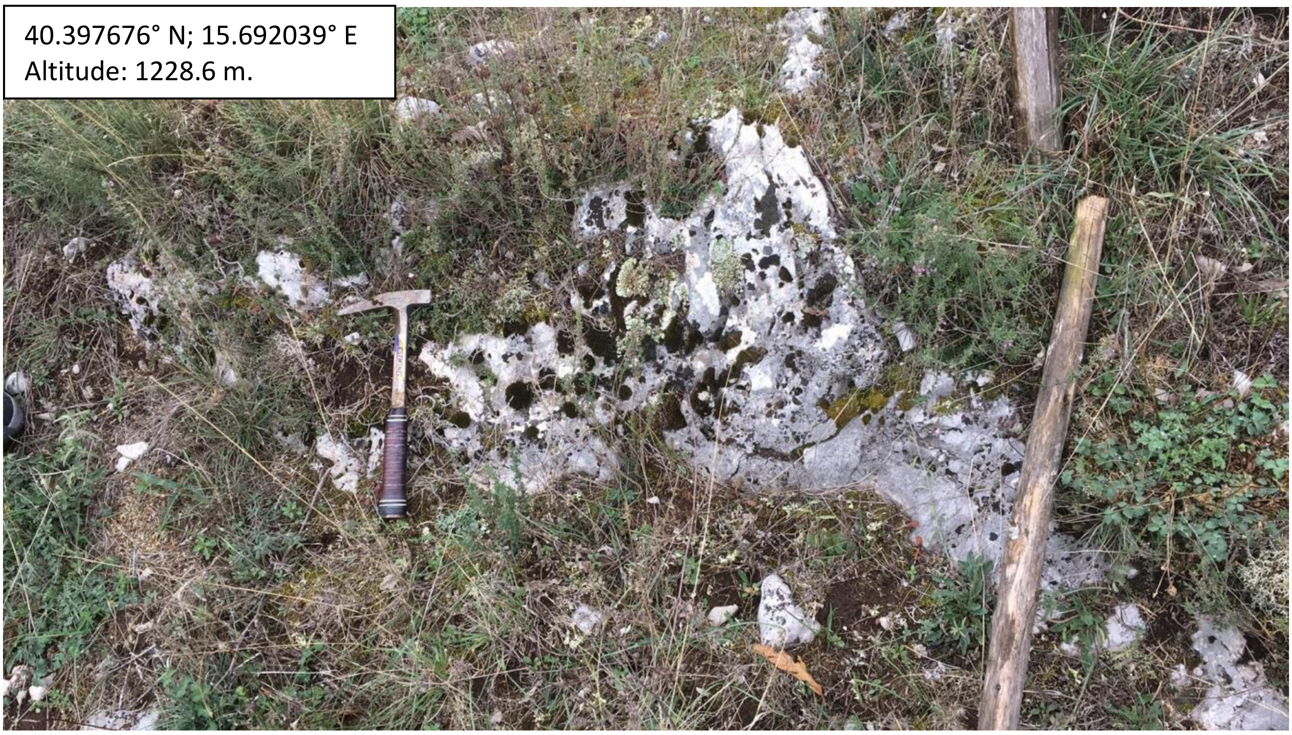

Figure 212.

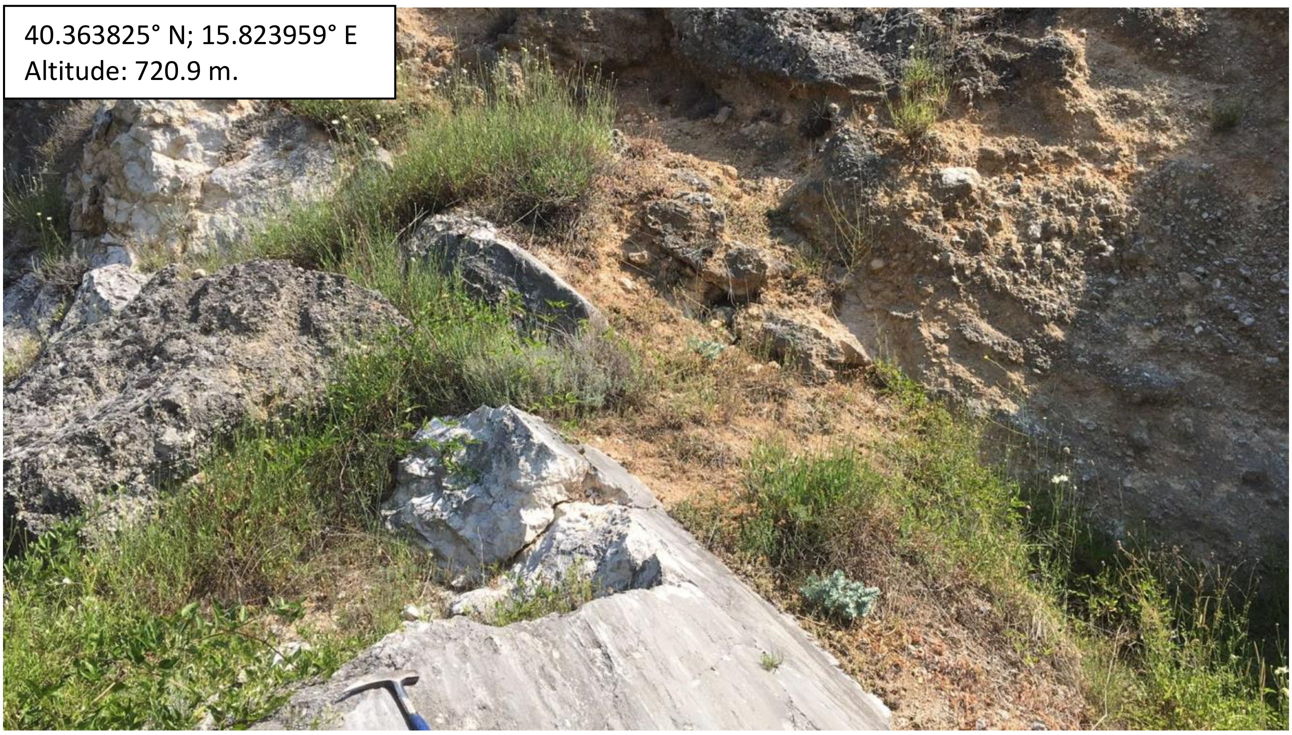

Figure 213.

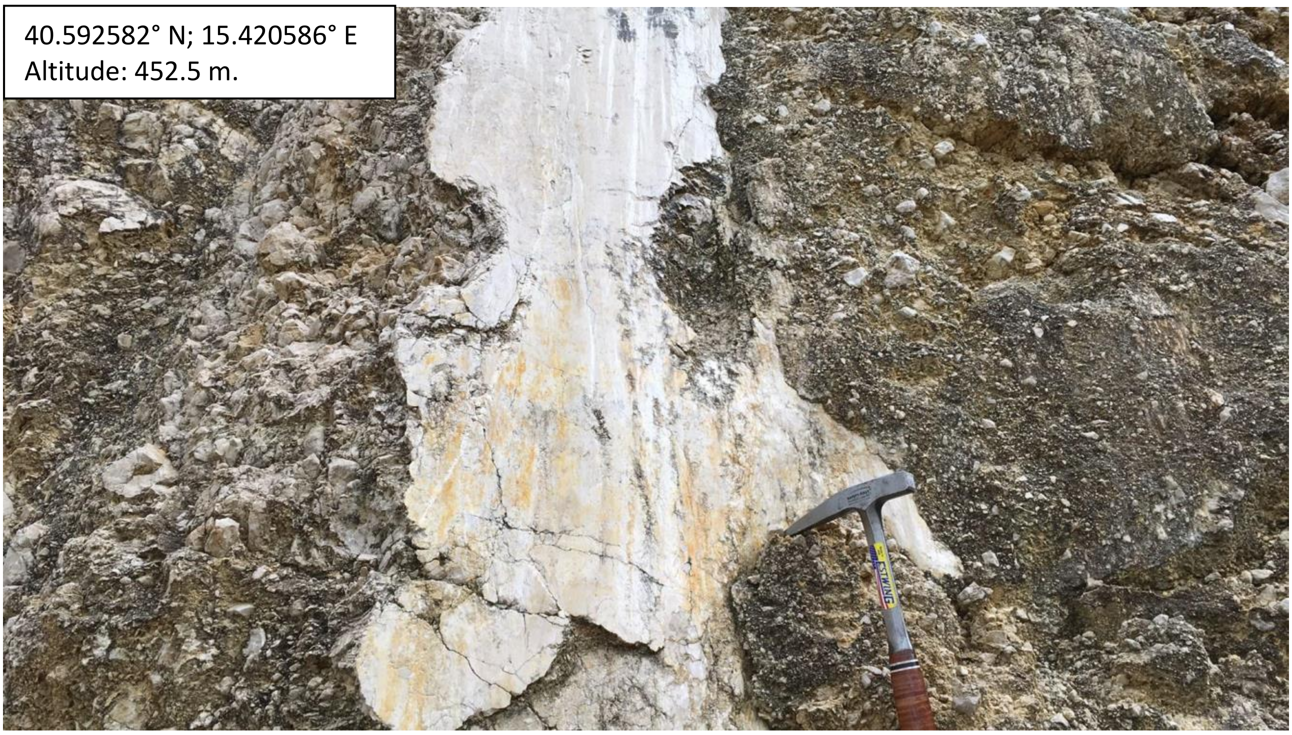

Figure 214.

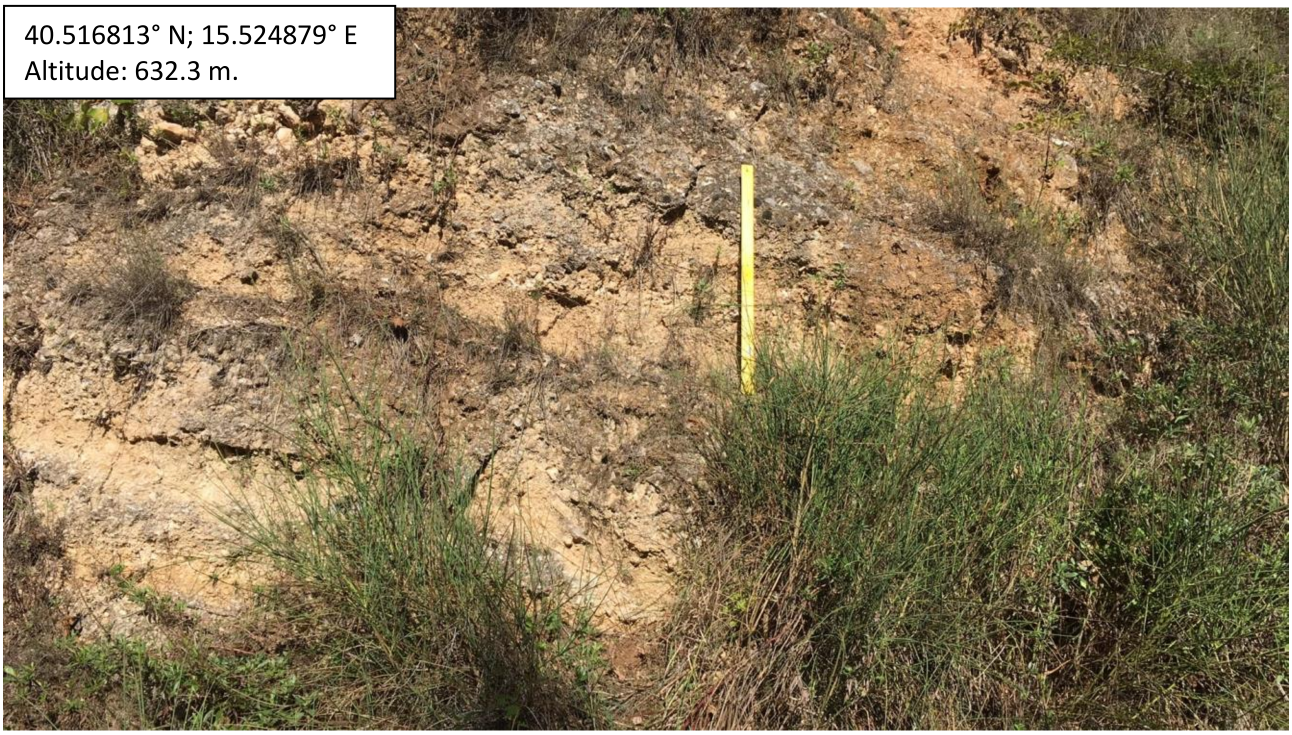

Figure 215.

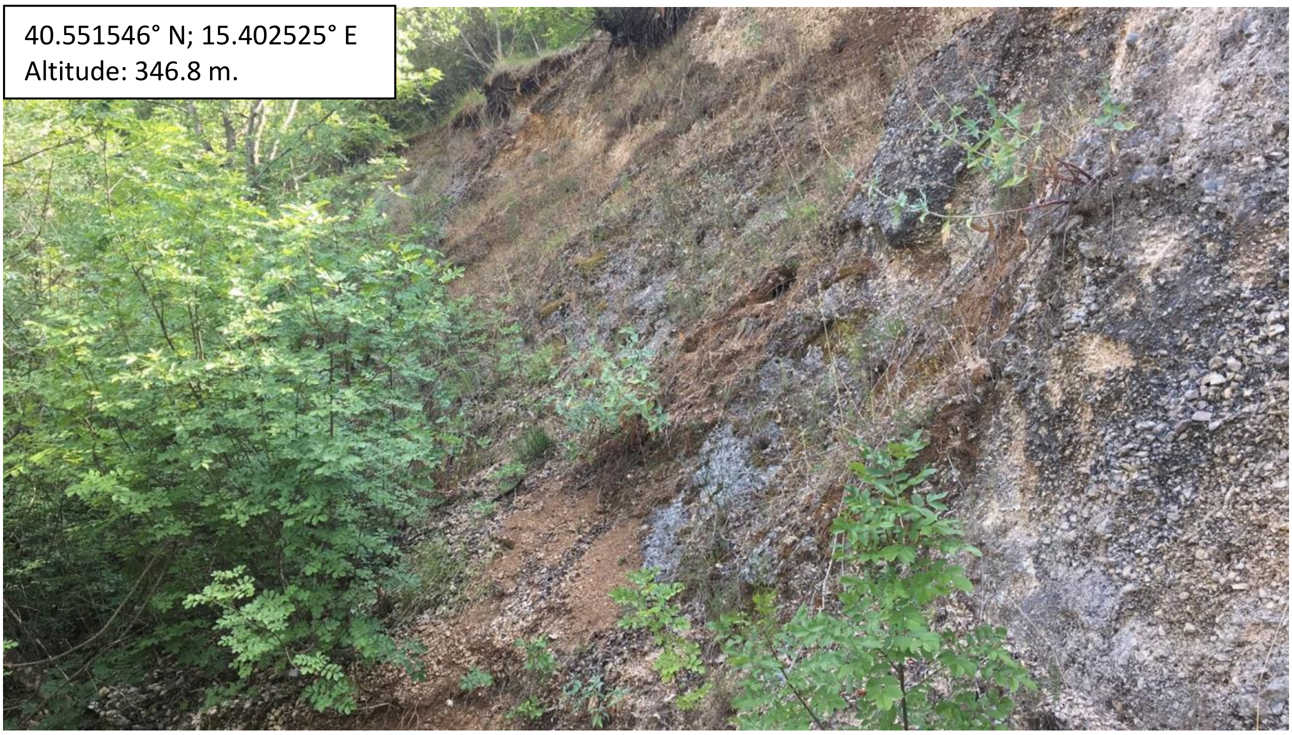

Figure 216.

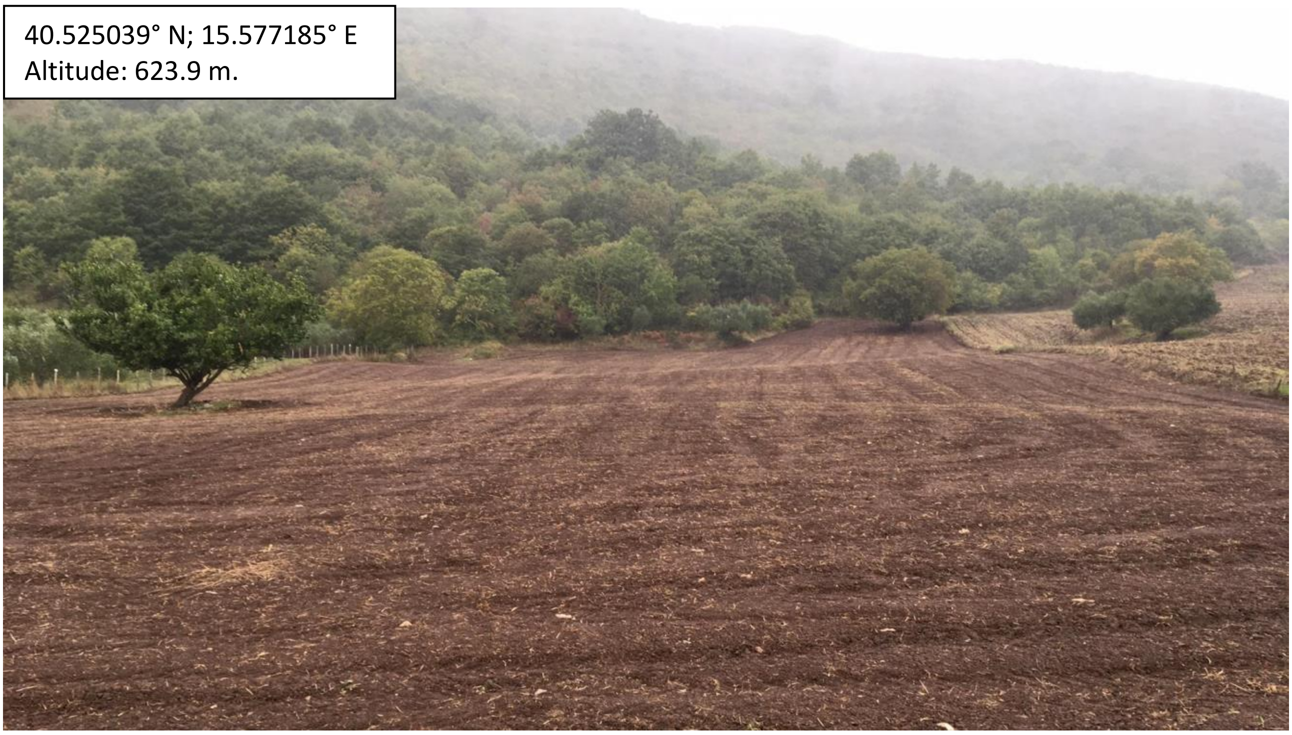

Figure 217.

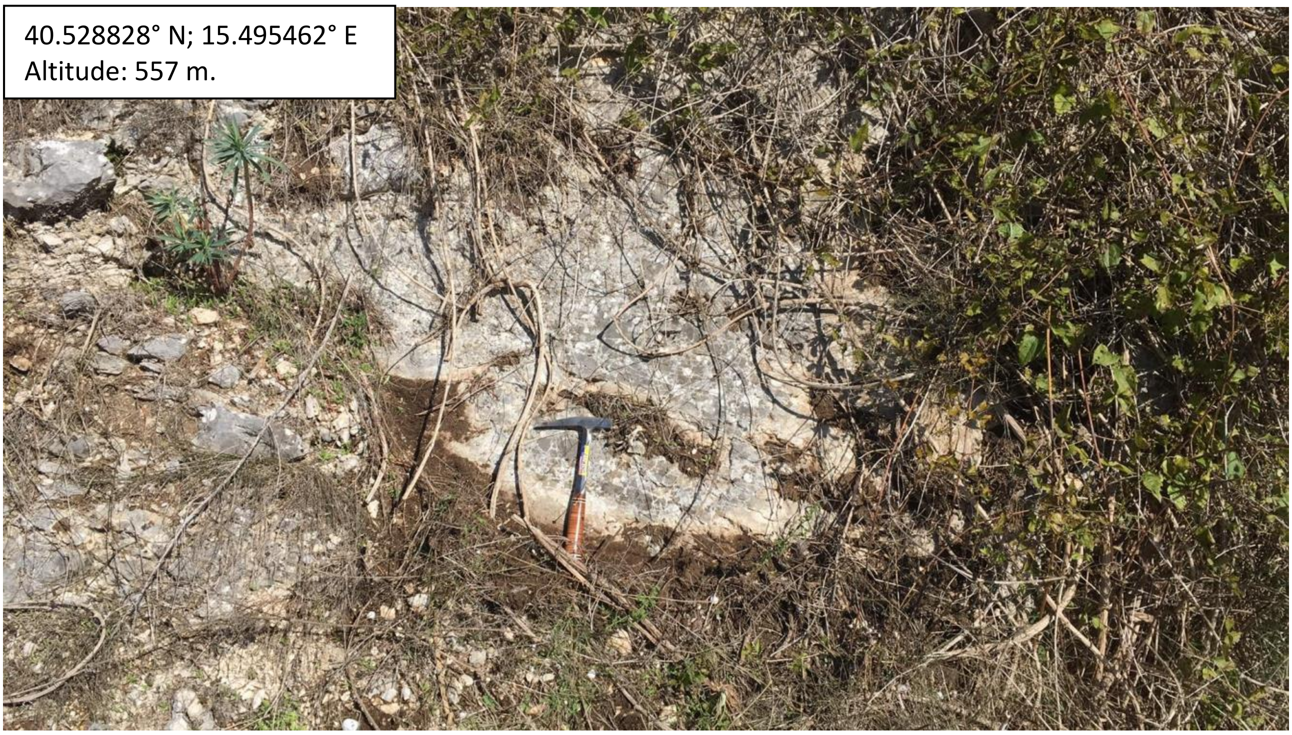

Figure 218.

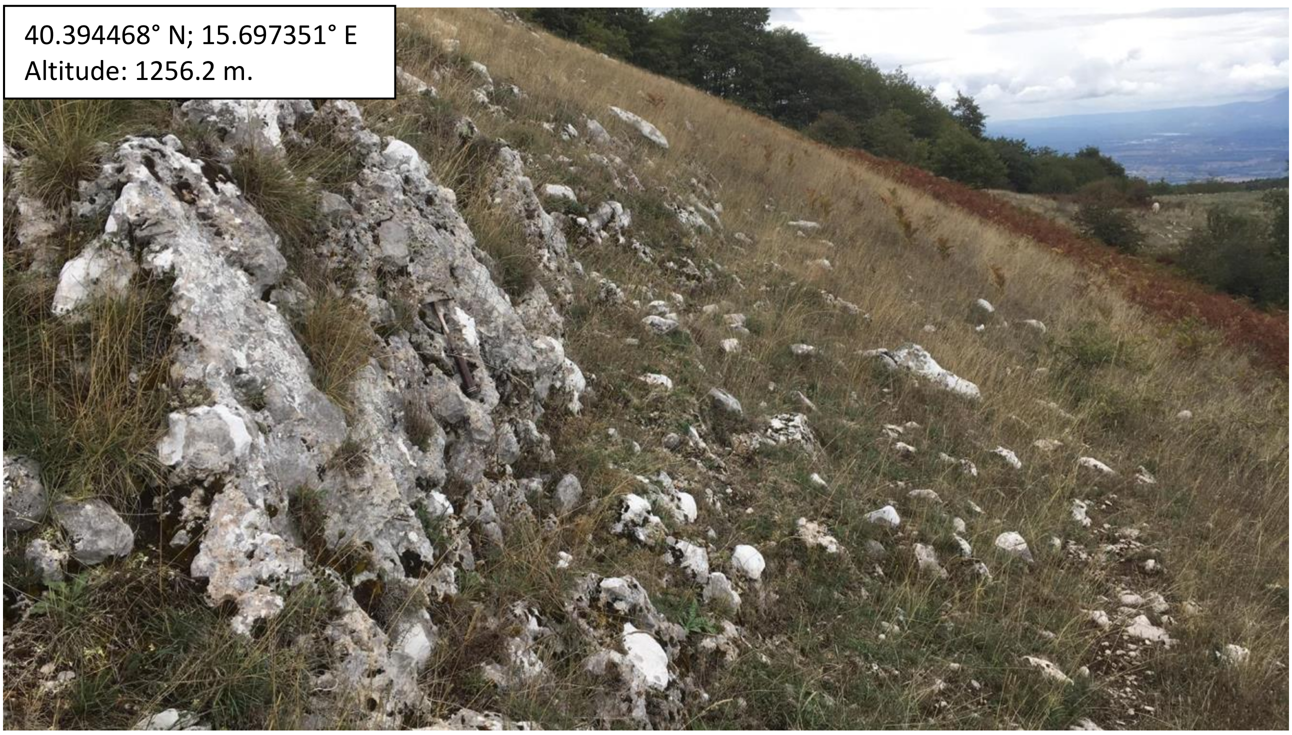

Figure 219.

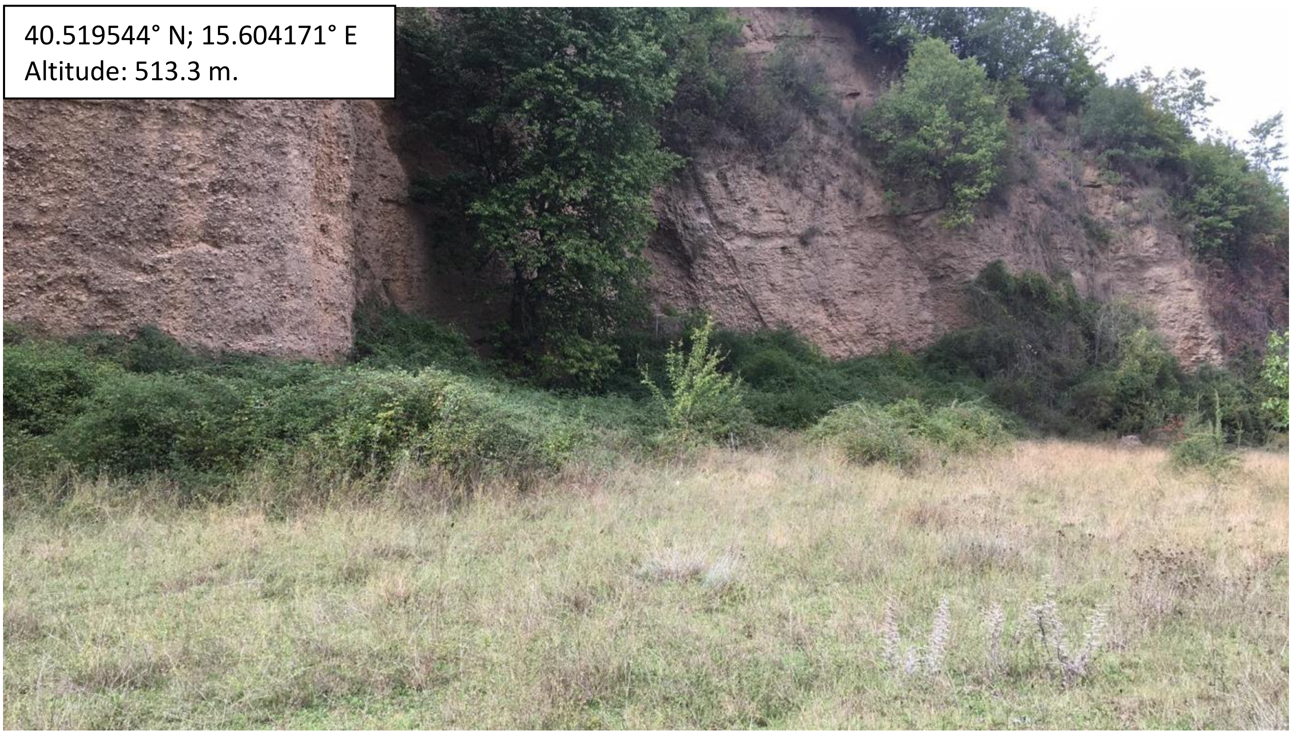

Figure 220.

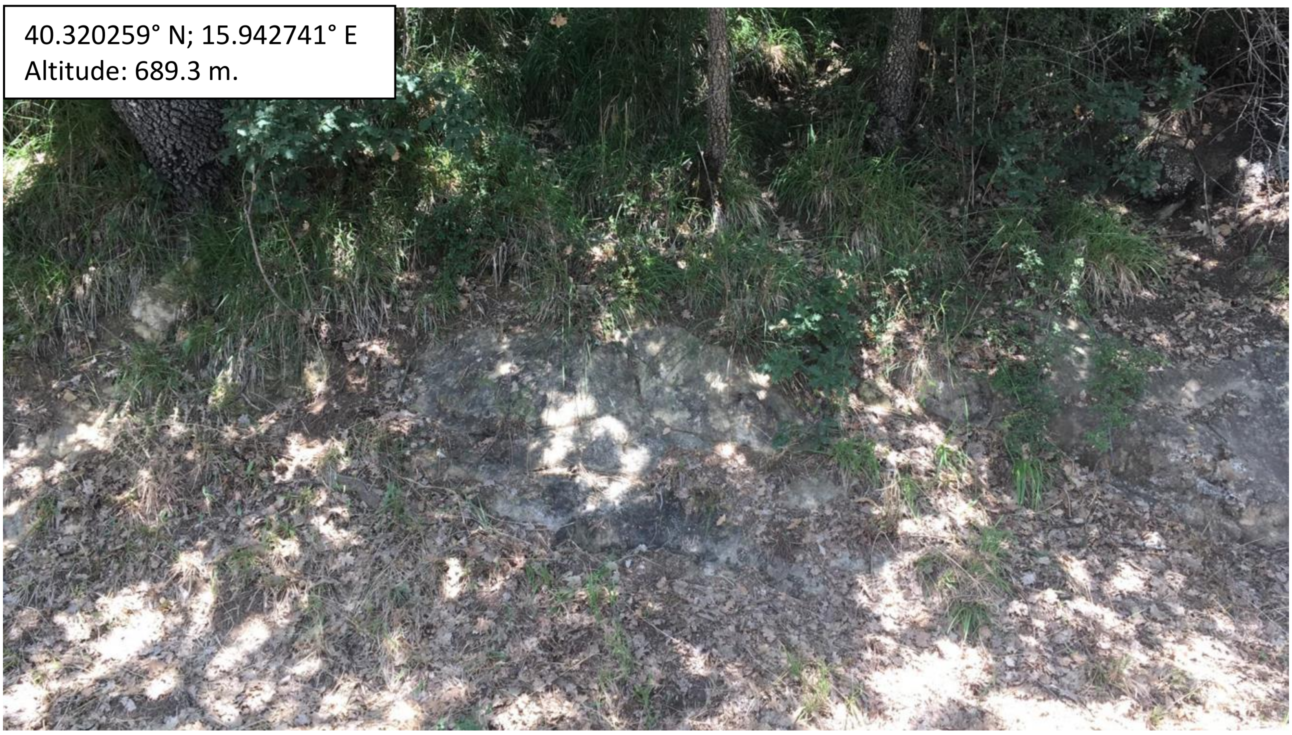

Figure 221.

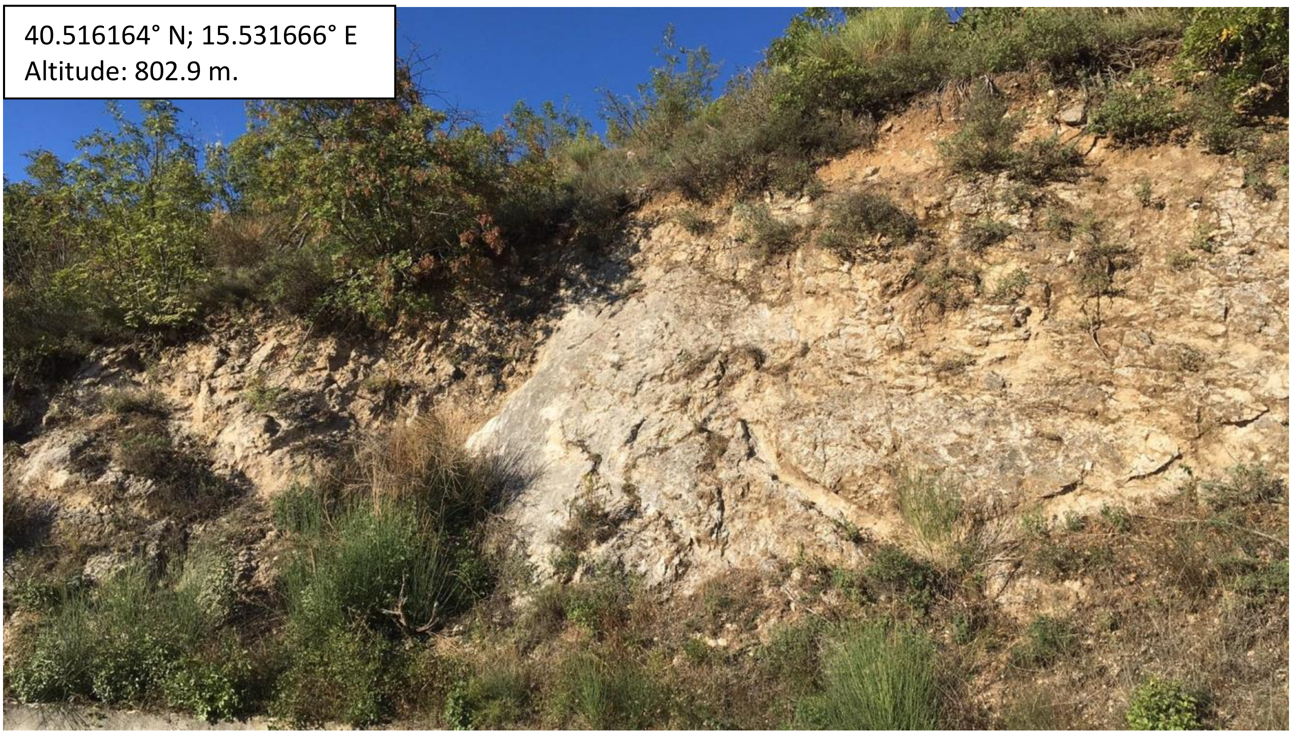

Figure 222.

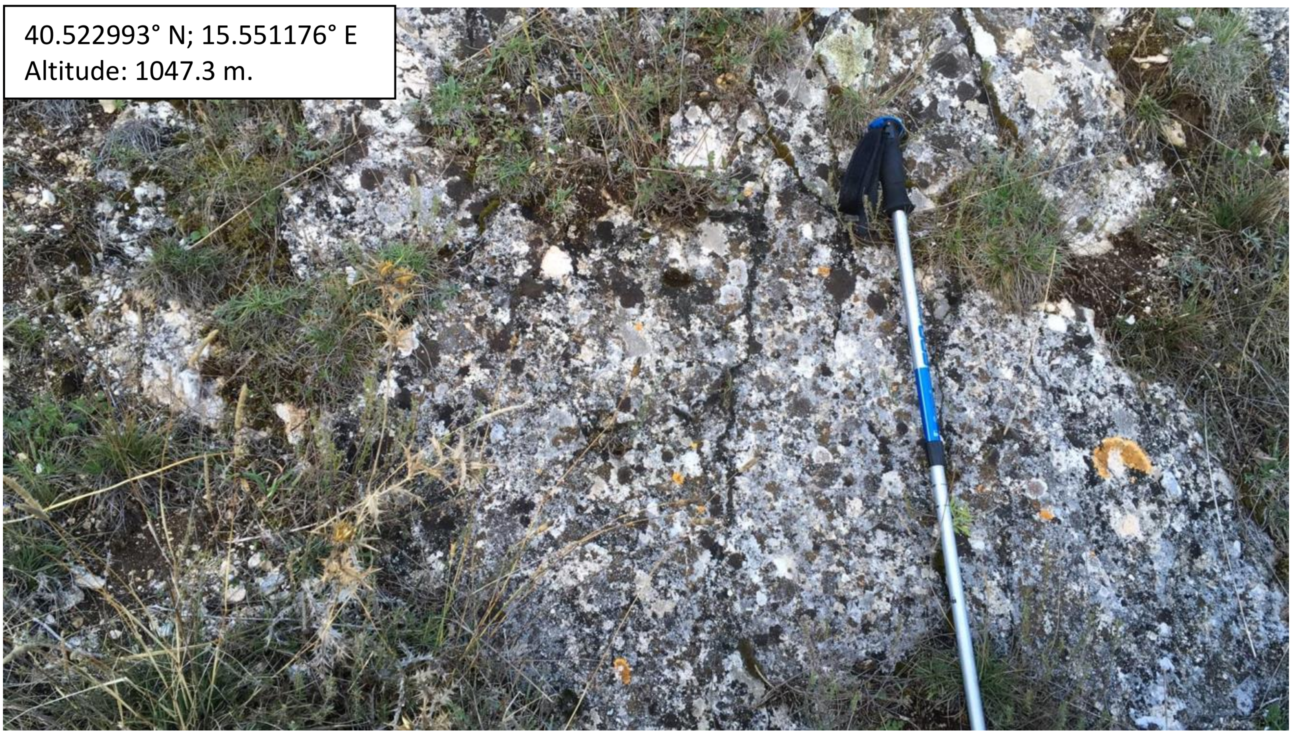

Figure 223.

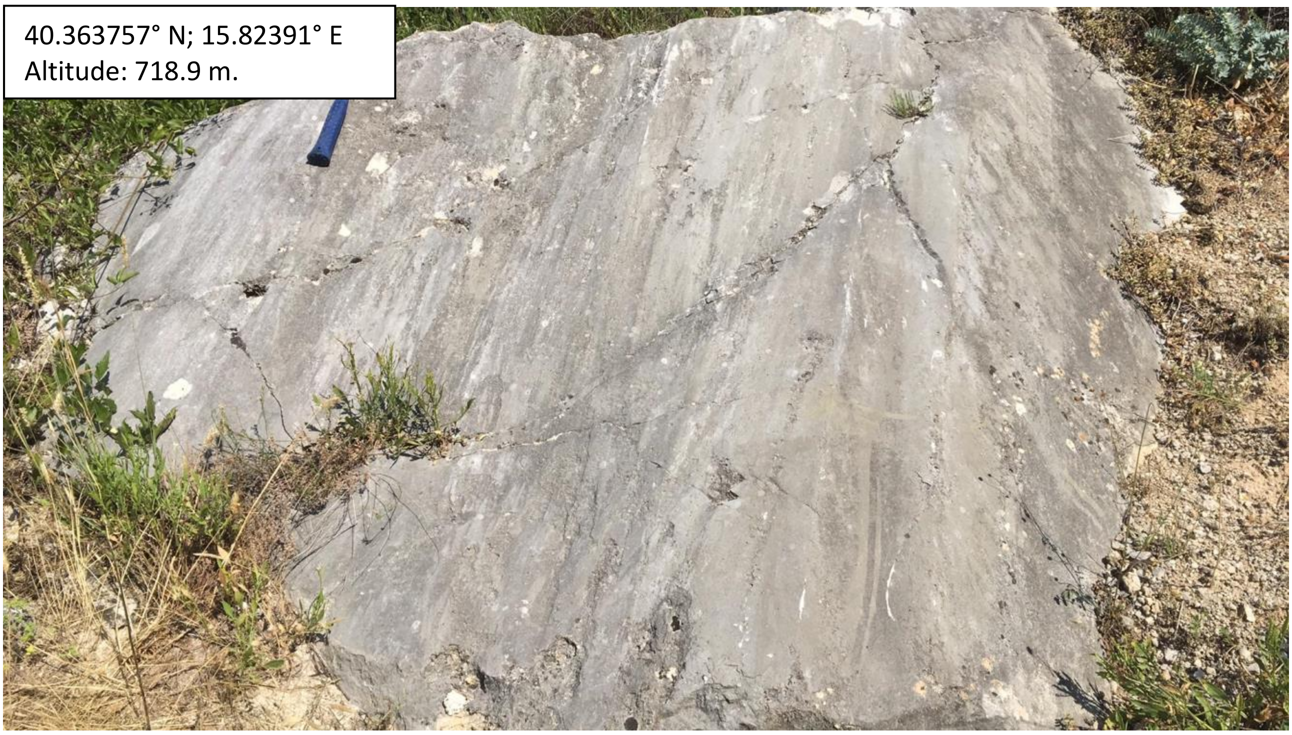

Figure 224.

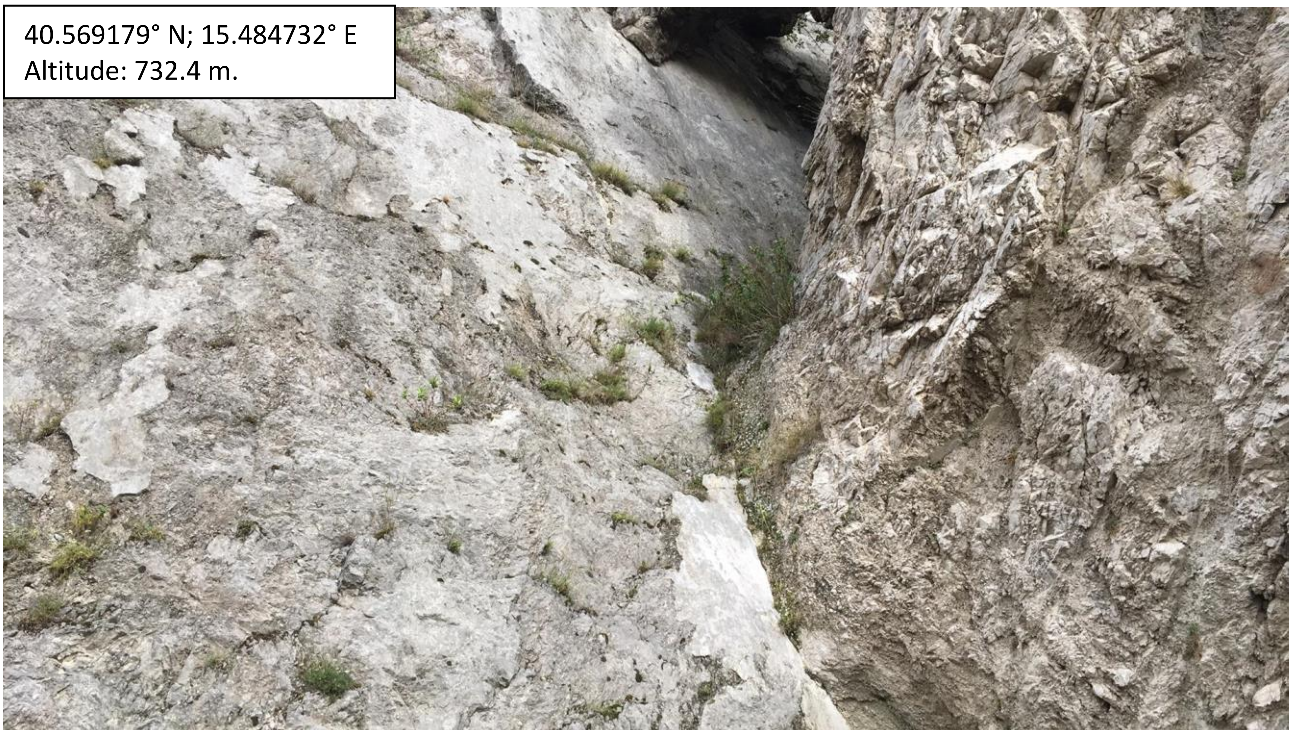

Figure 225.

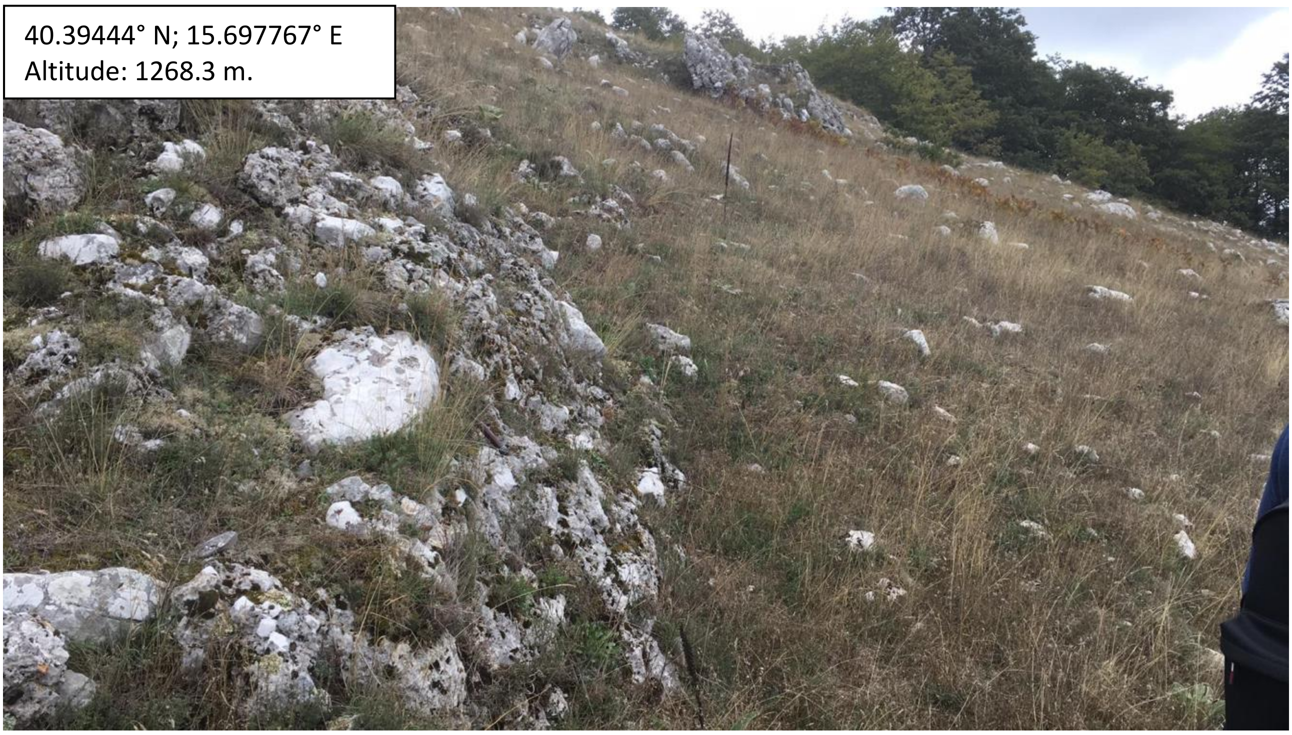

Figure 226.

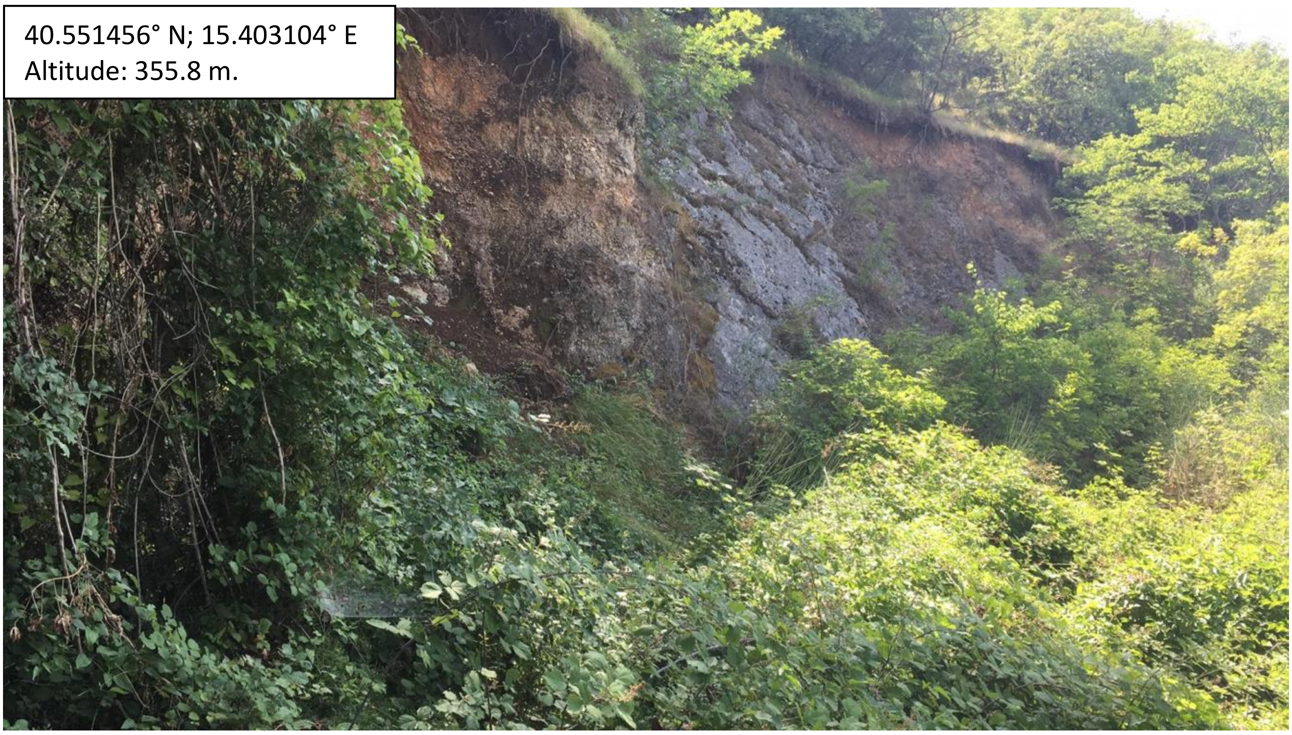

Figure 227.

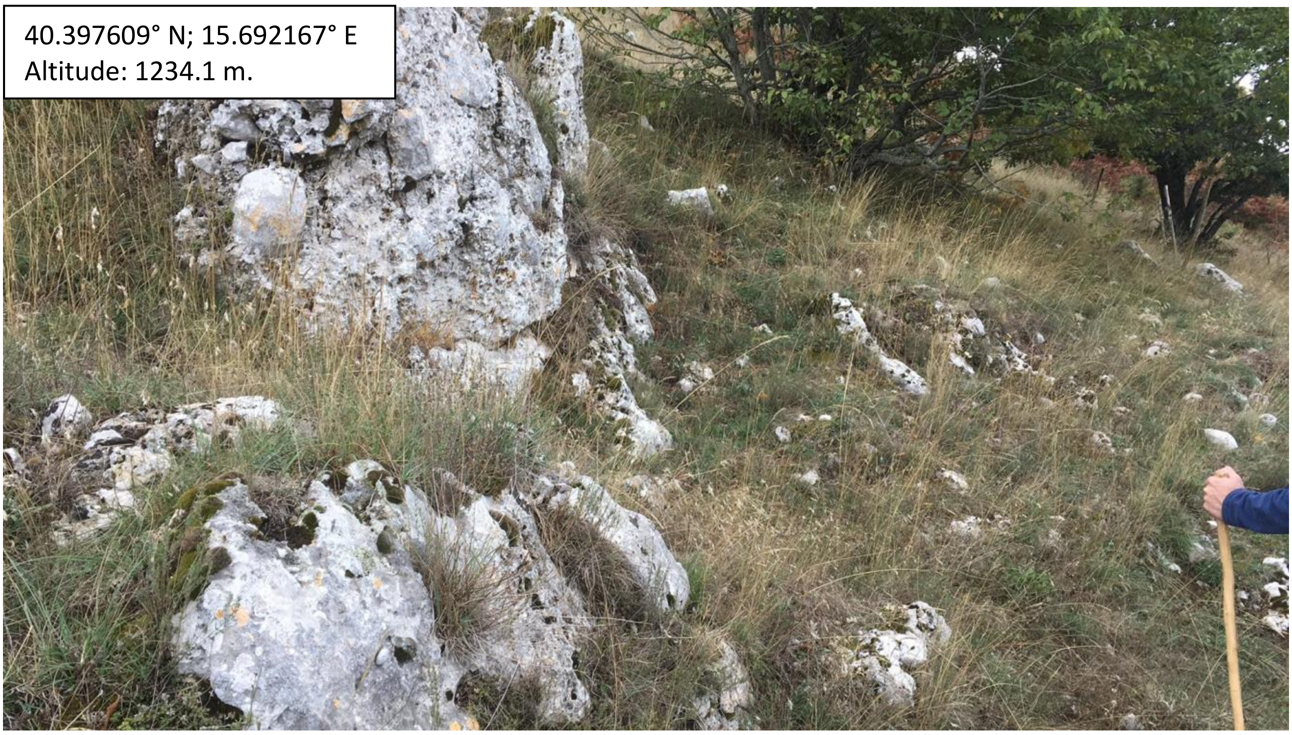

Figure 228.

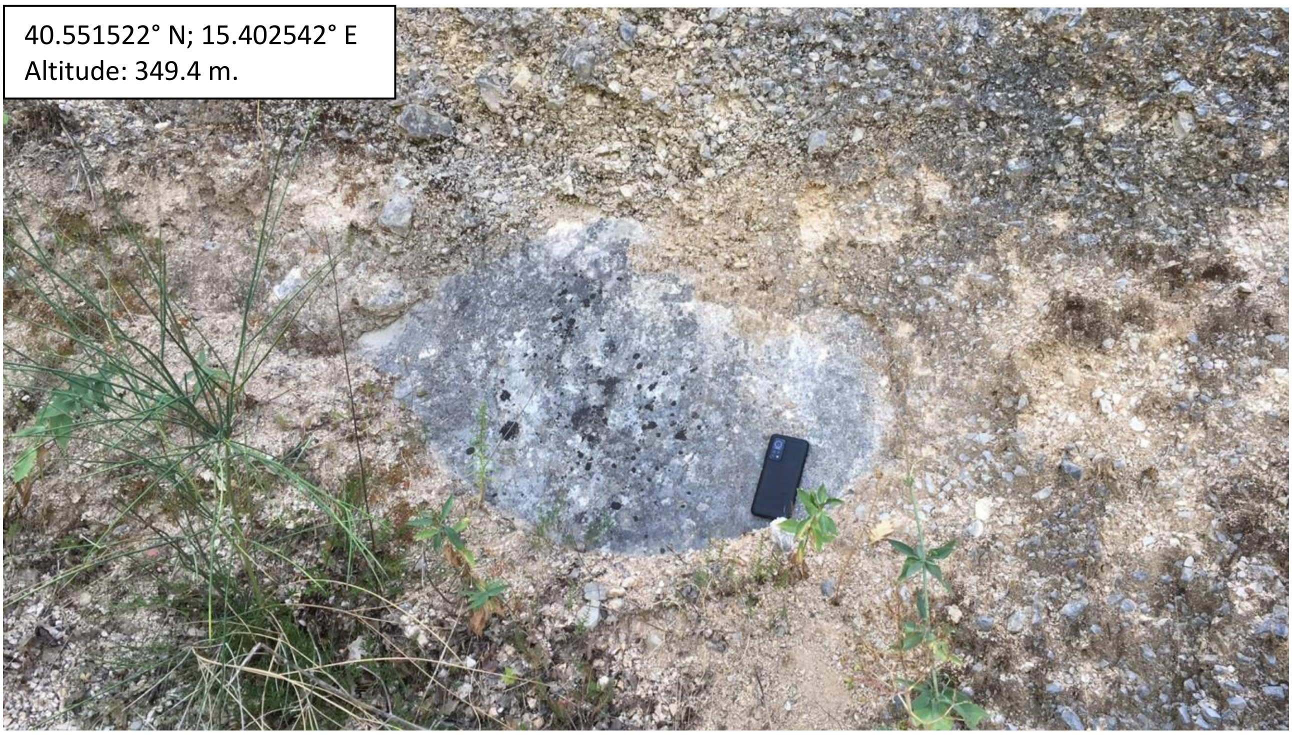

Figure 229.

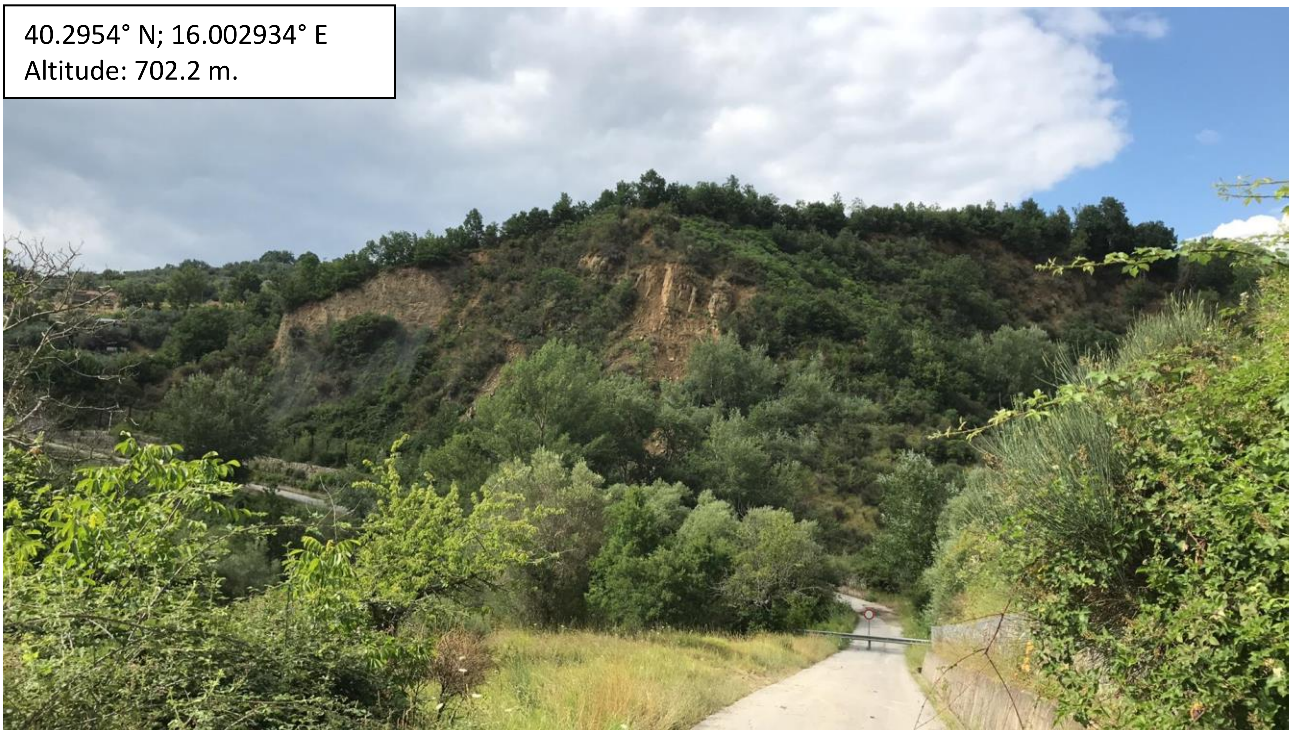

Figure 231.

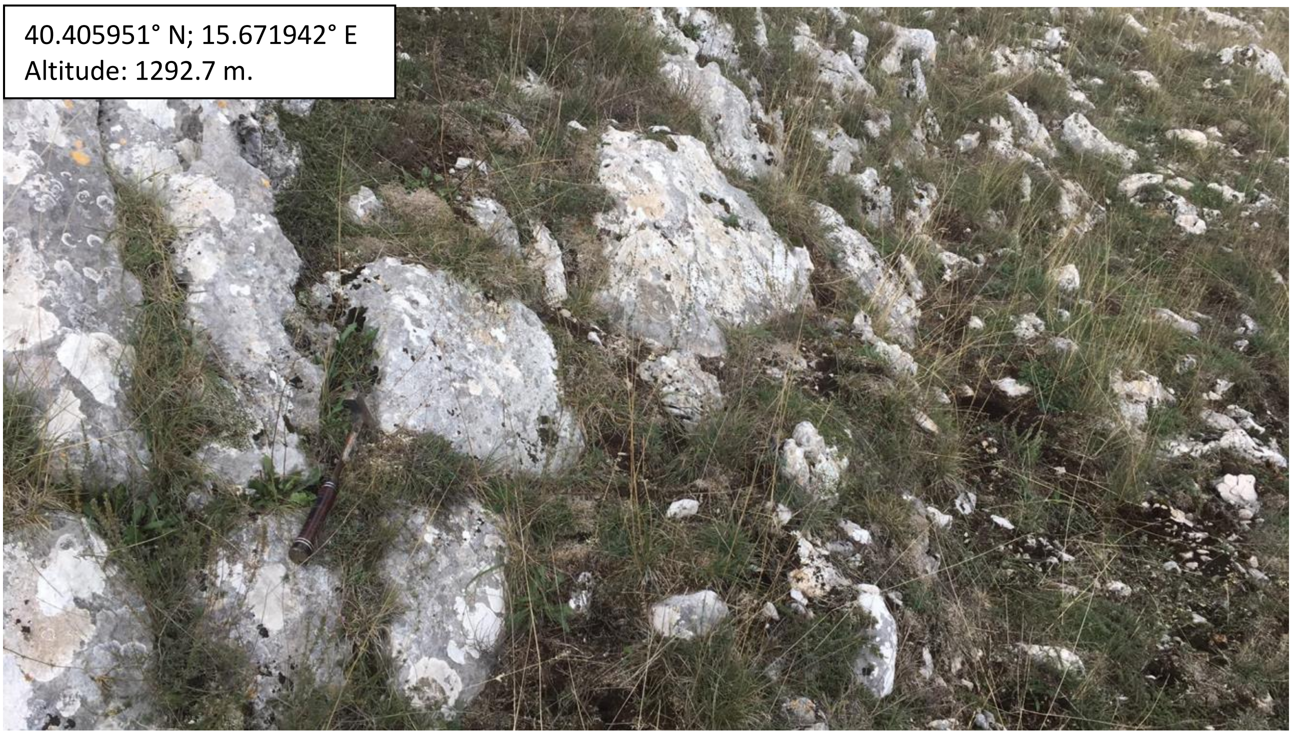

Figure 232

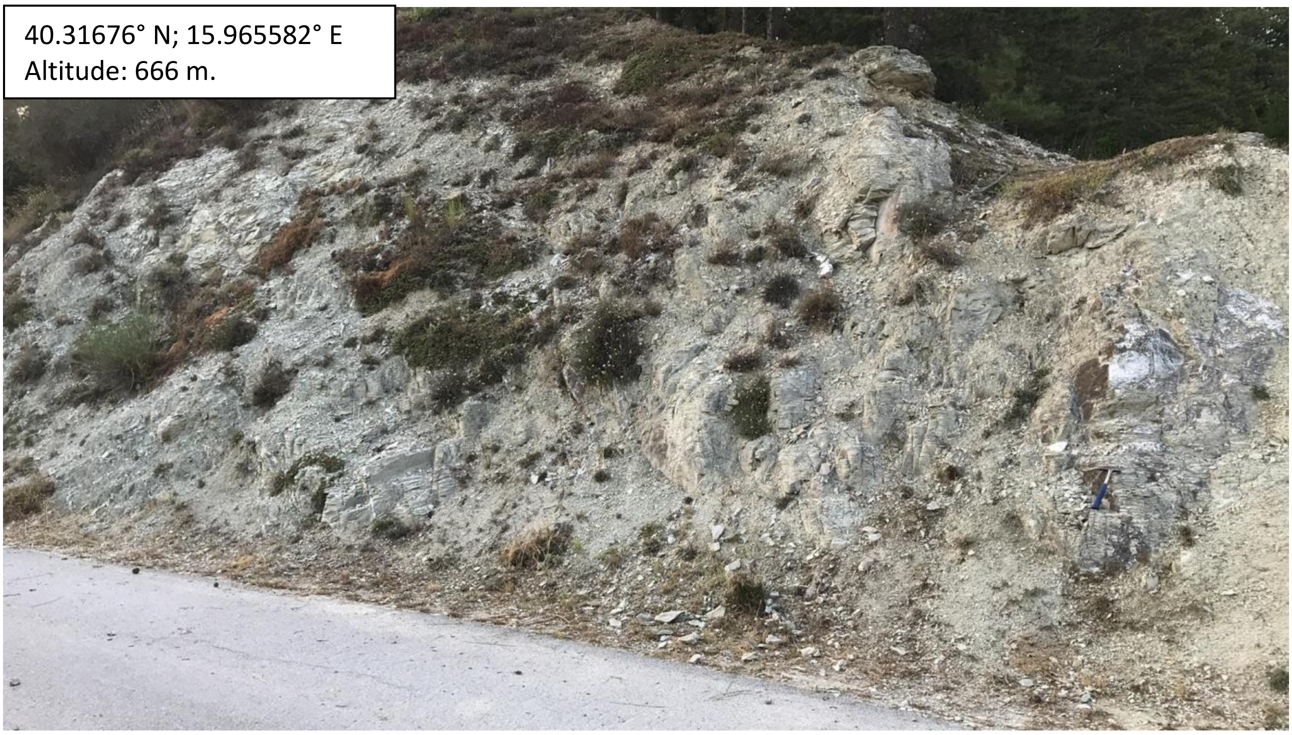

Figure 233.

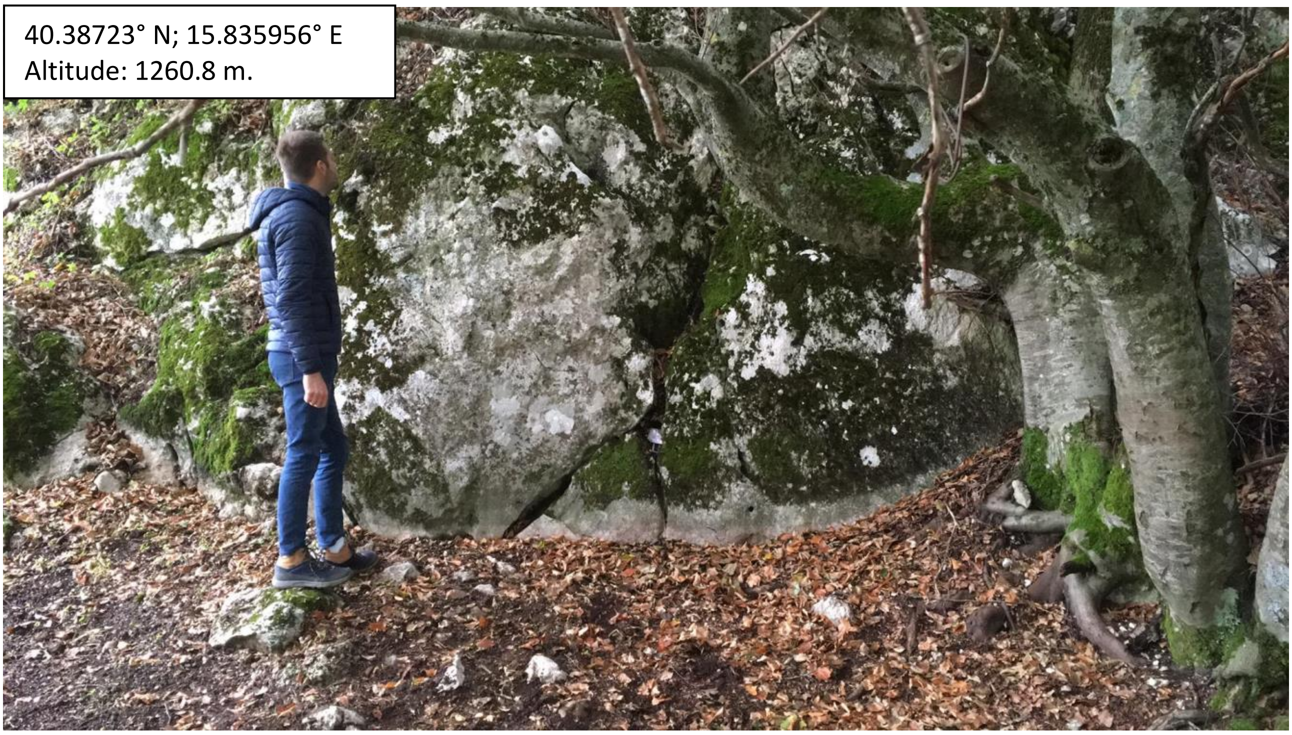

Figure 234.

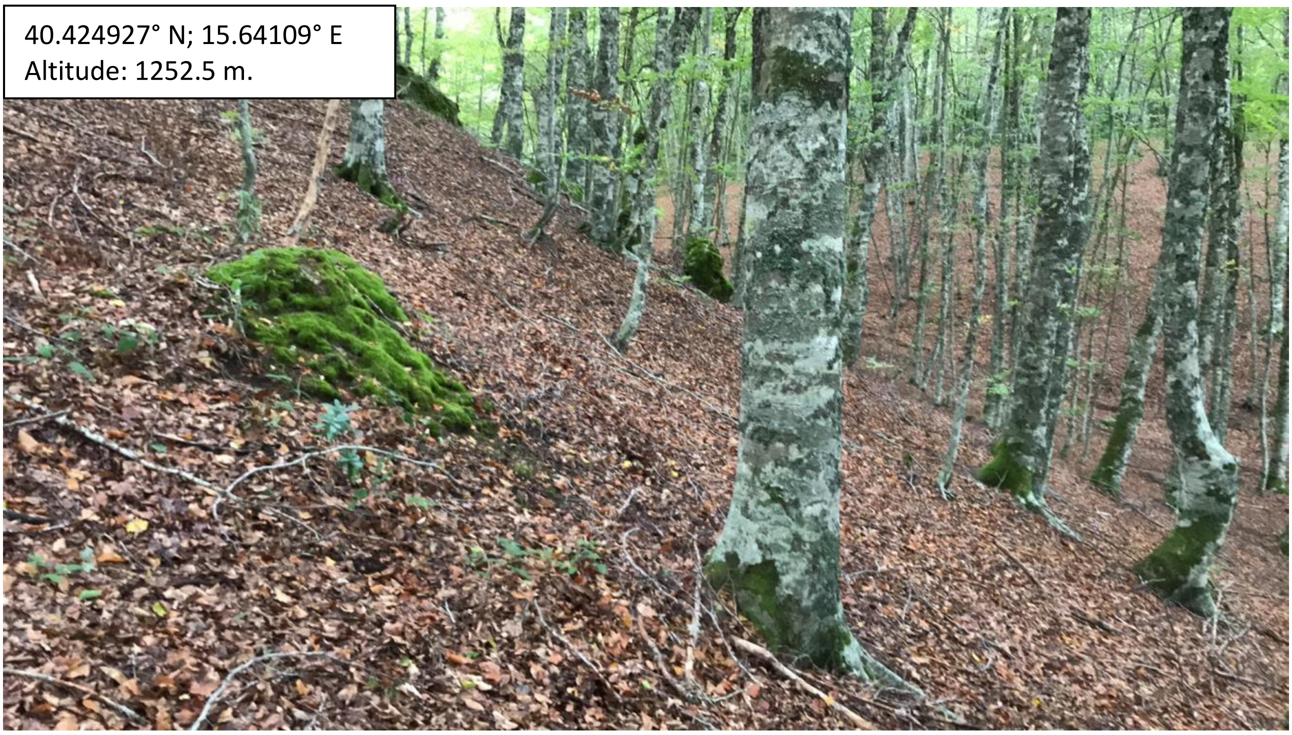

Figure 235.

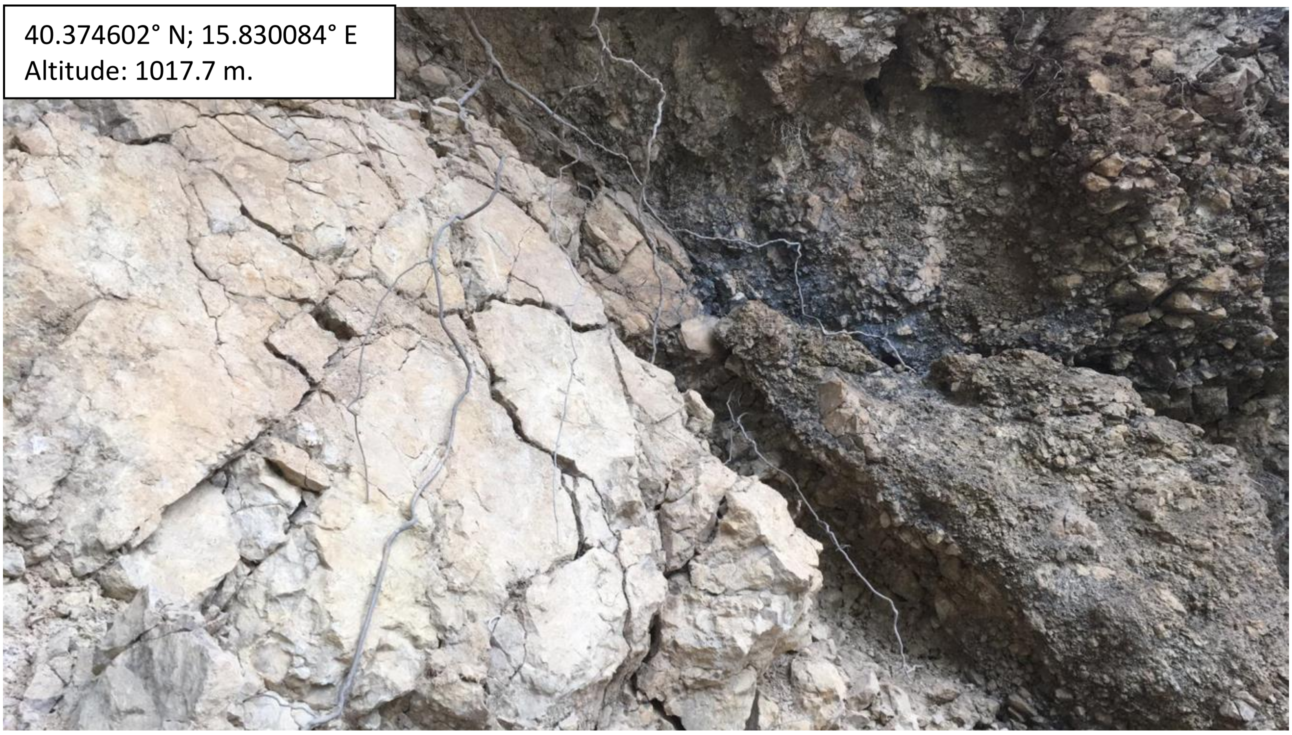

Figure 236.

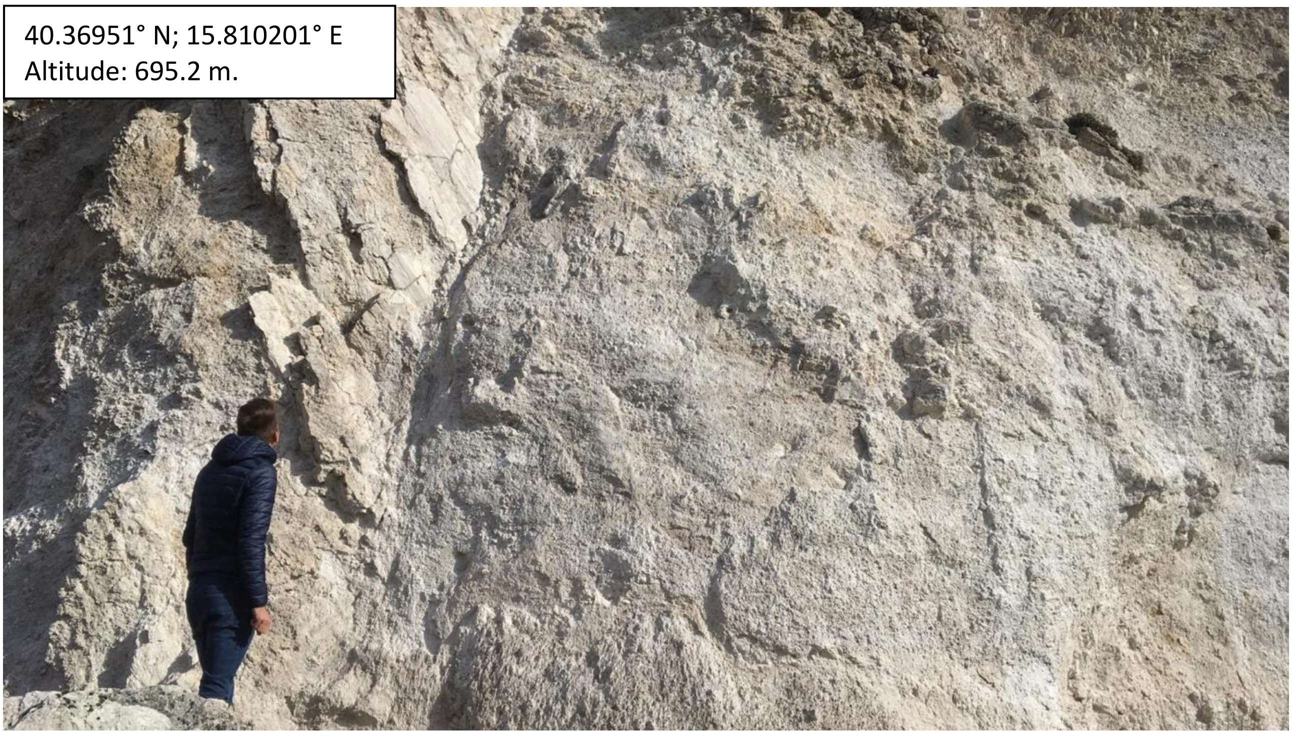

Figure 238.

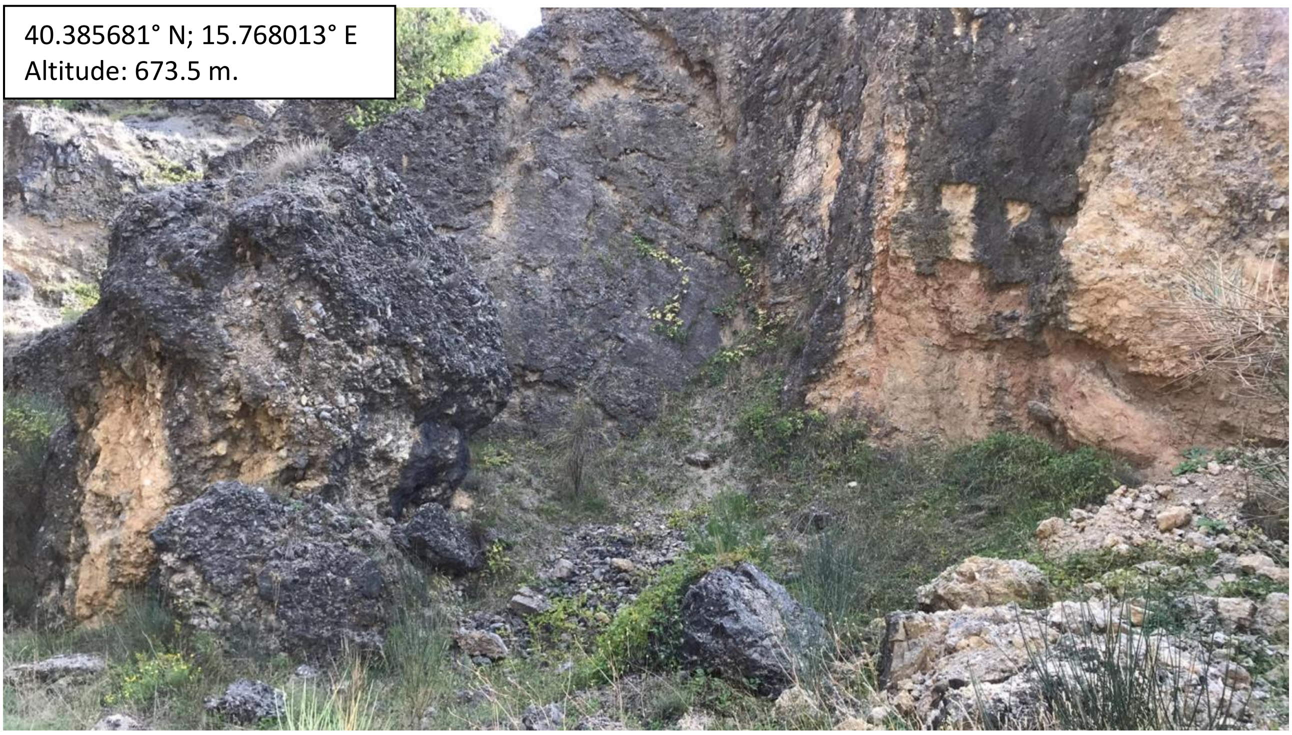

Figure 239.

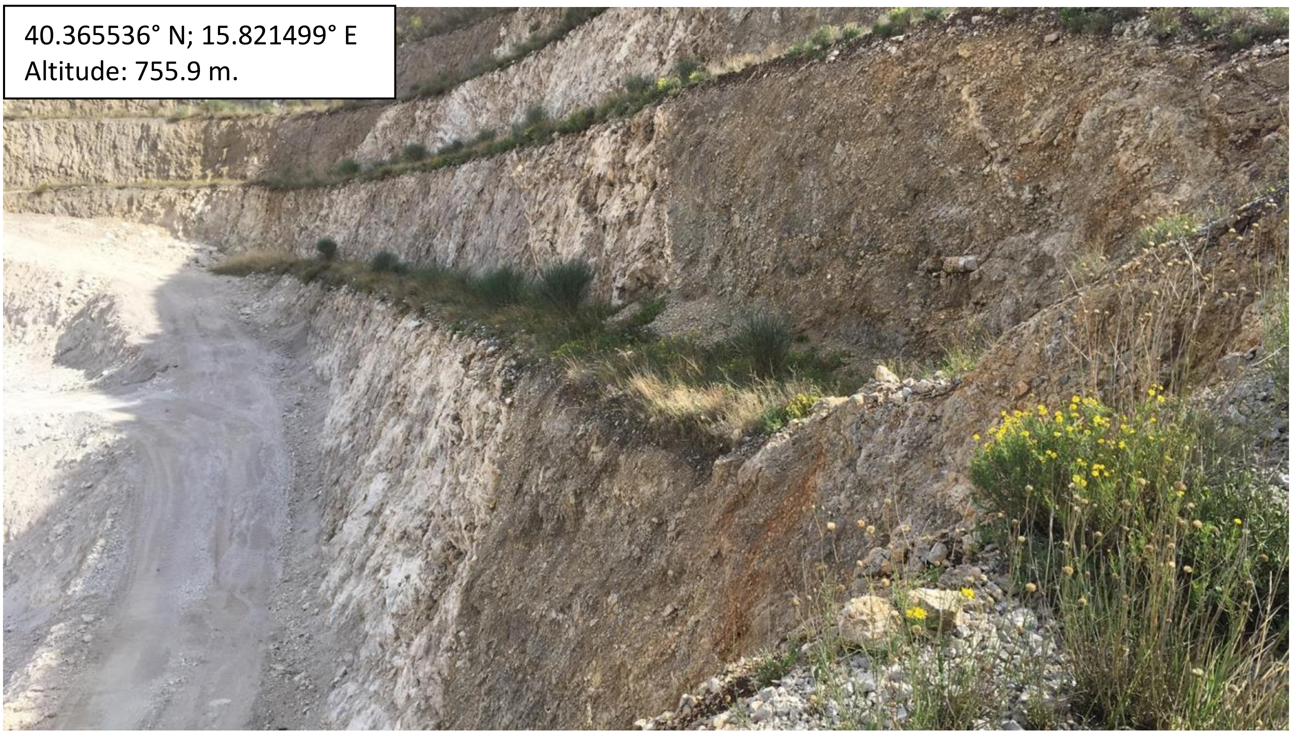

Figure 240.

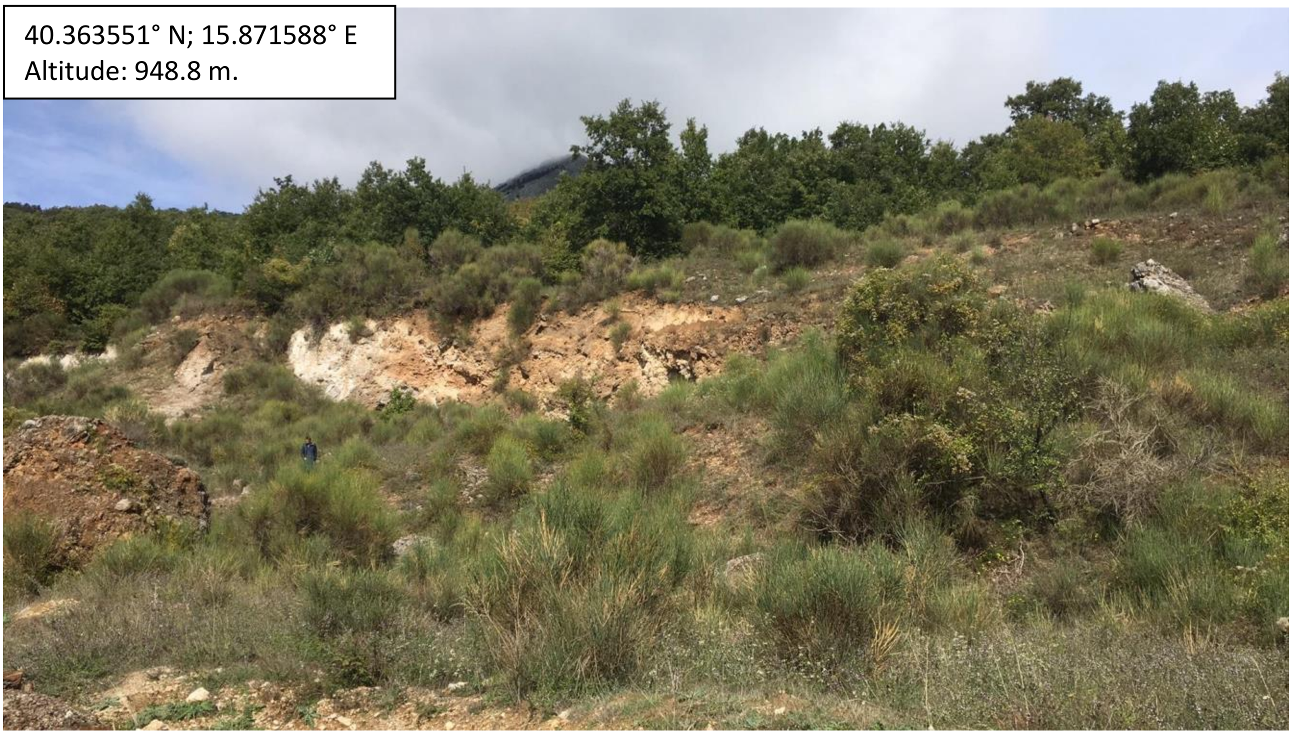

Figure 241.

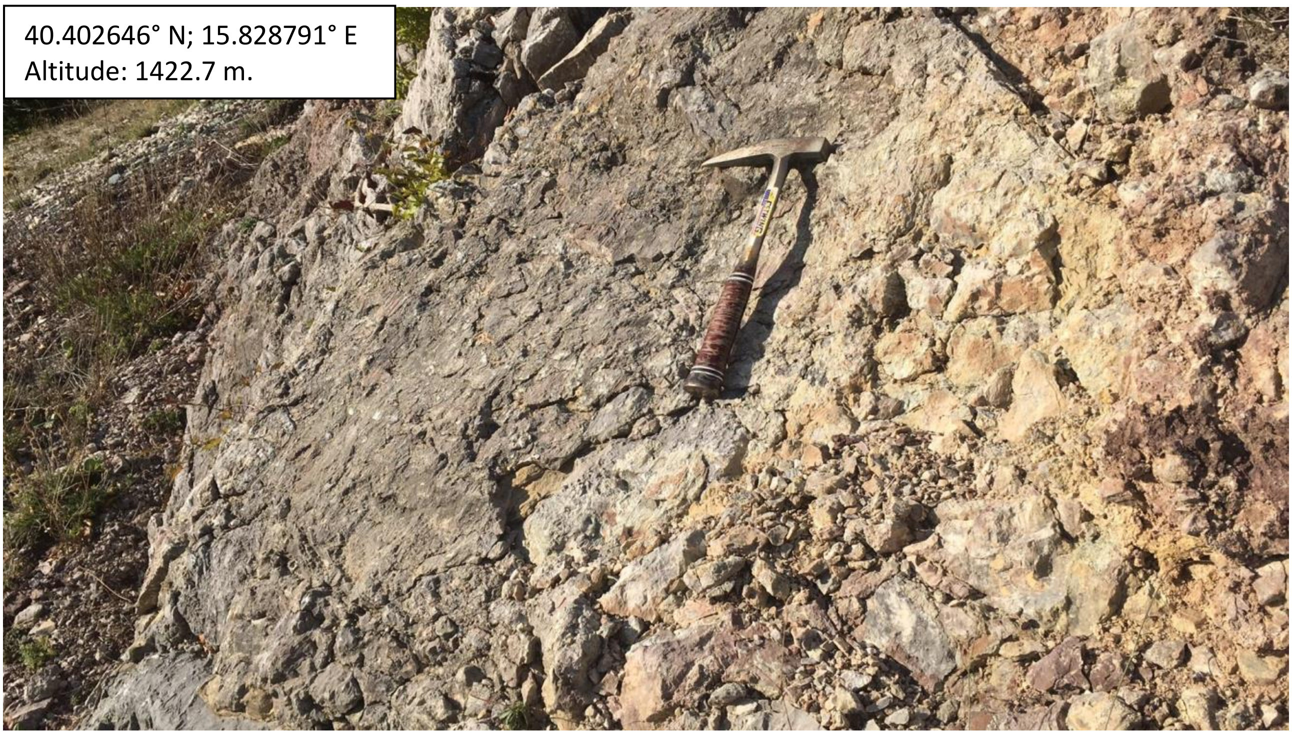

Figure 242.

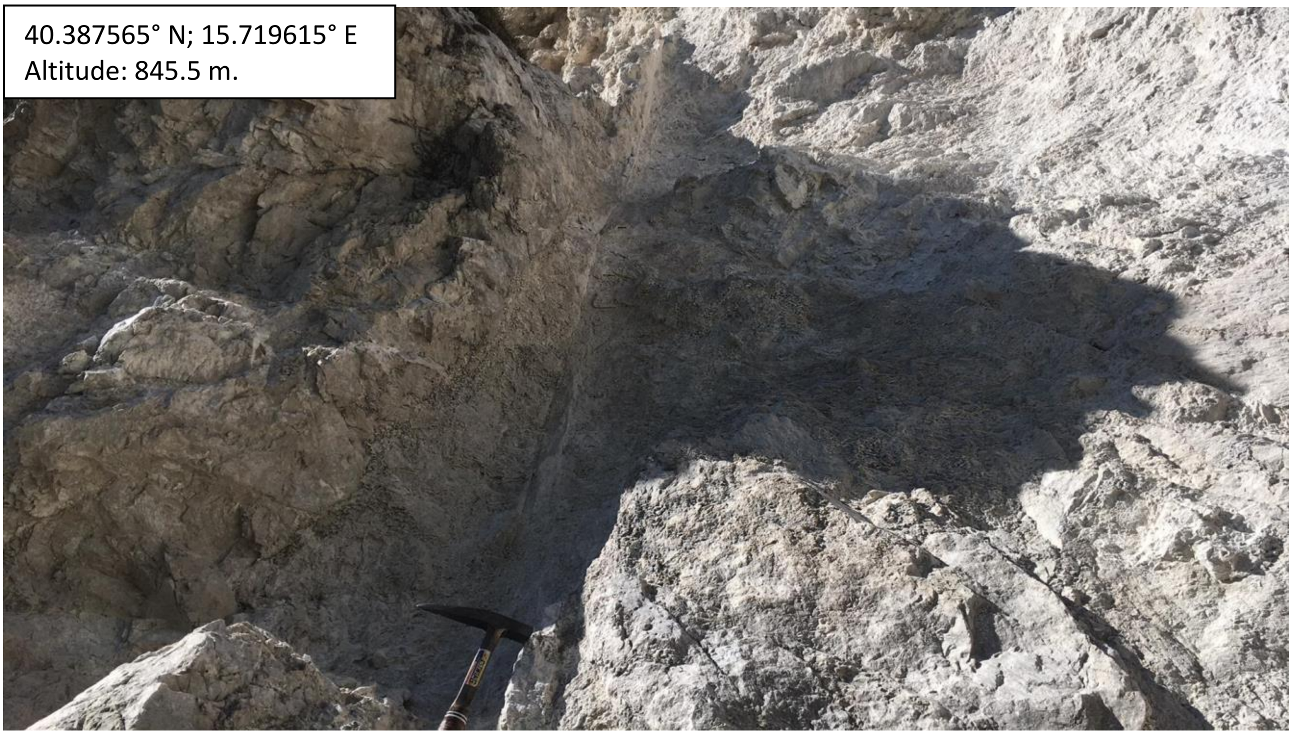

Figure 244.

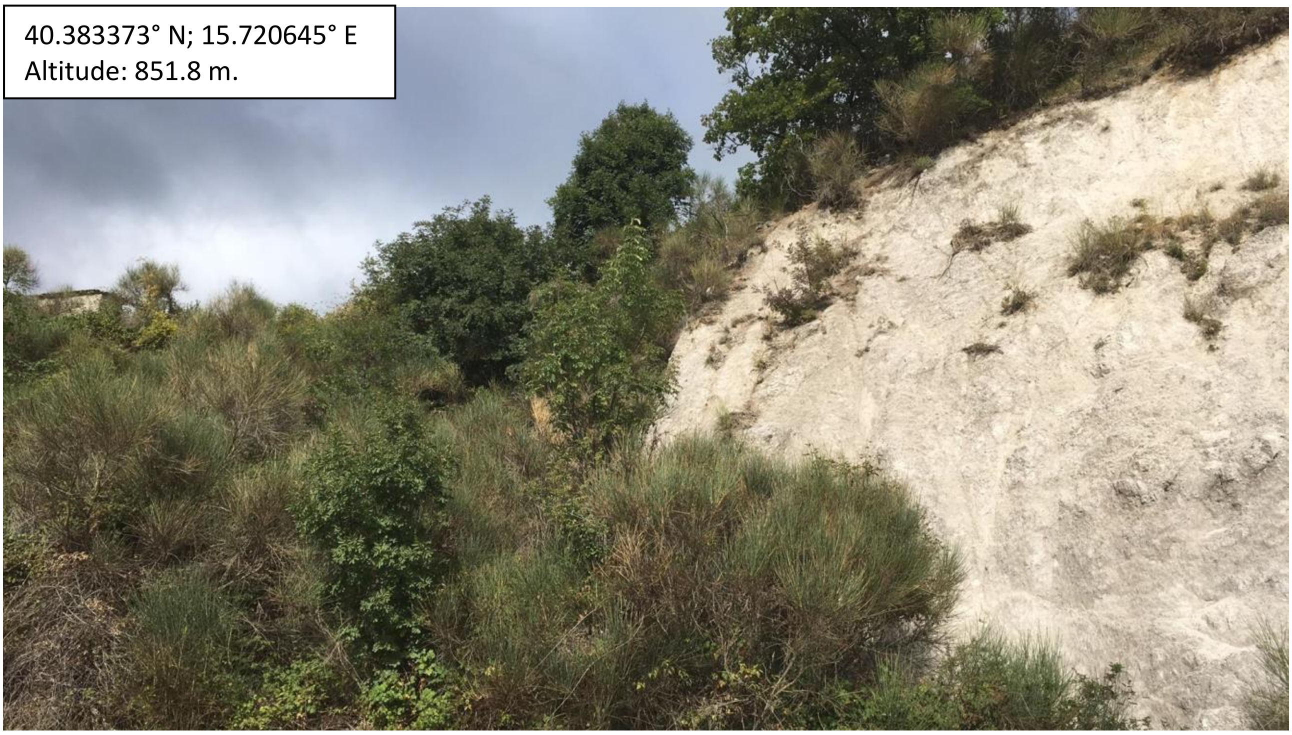

Figure 245.

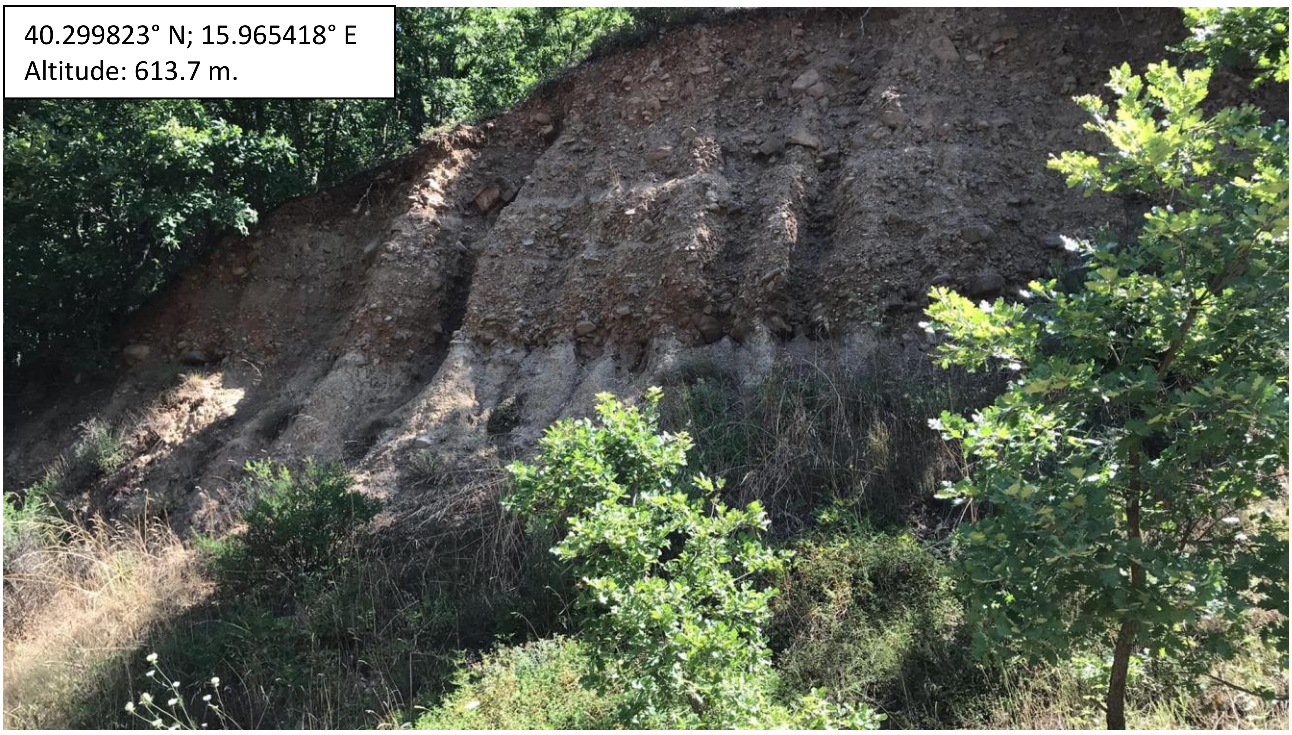

Figure 246.

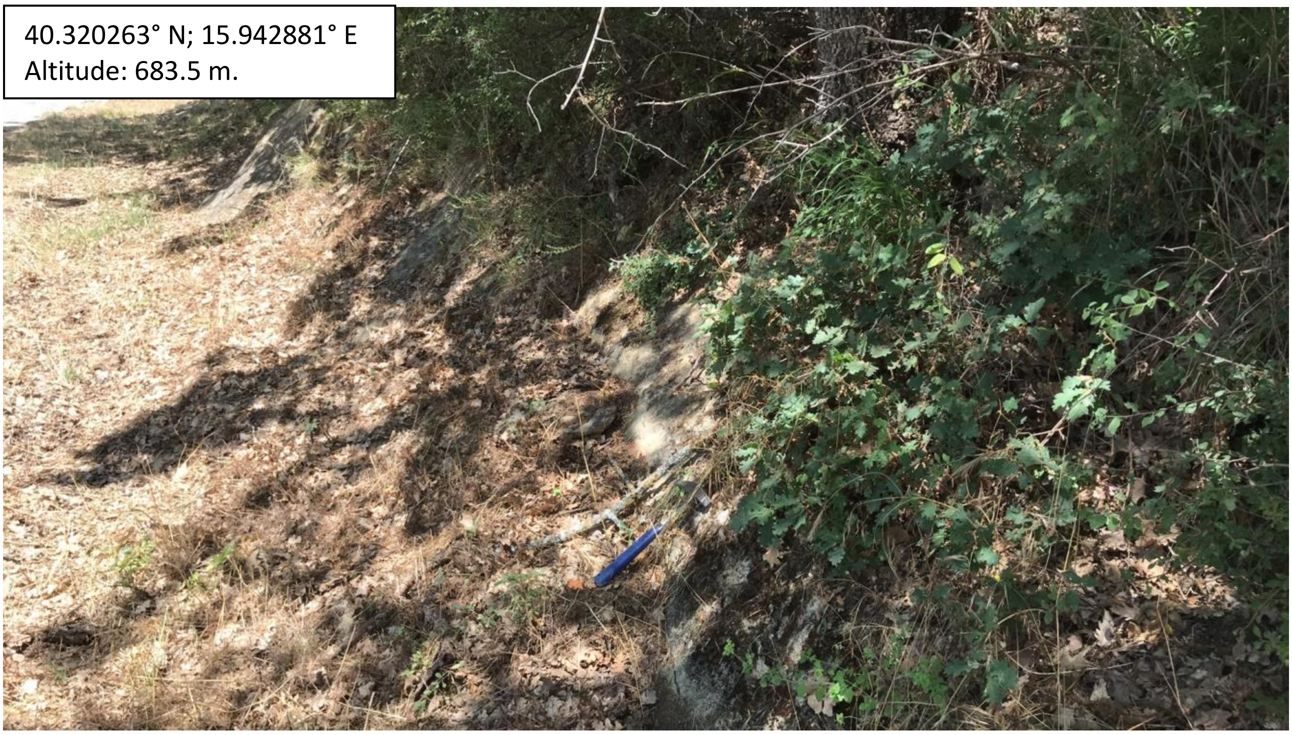

Figure 247.

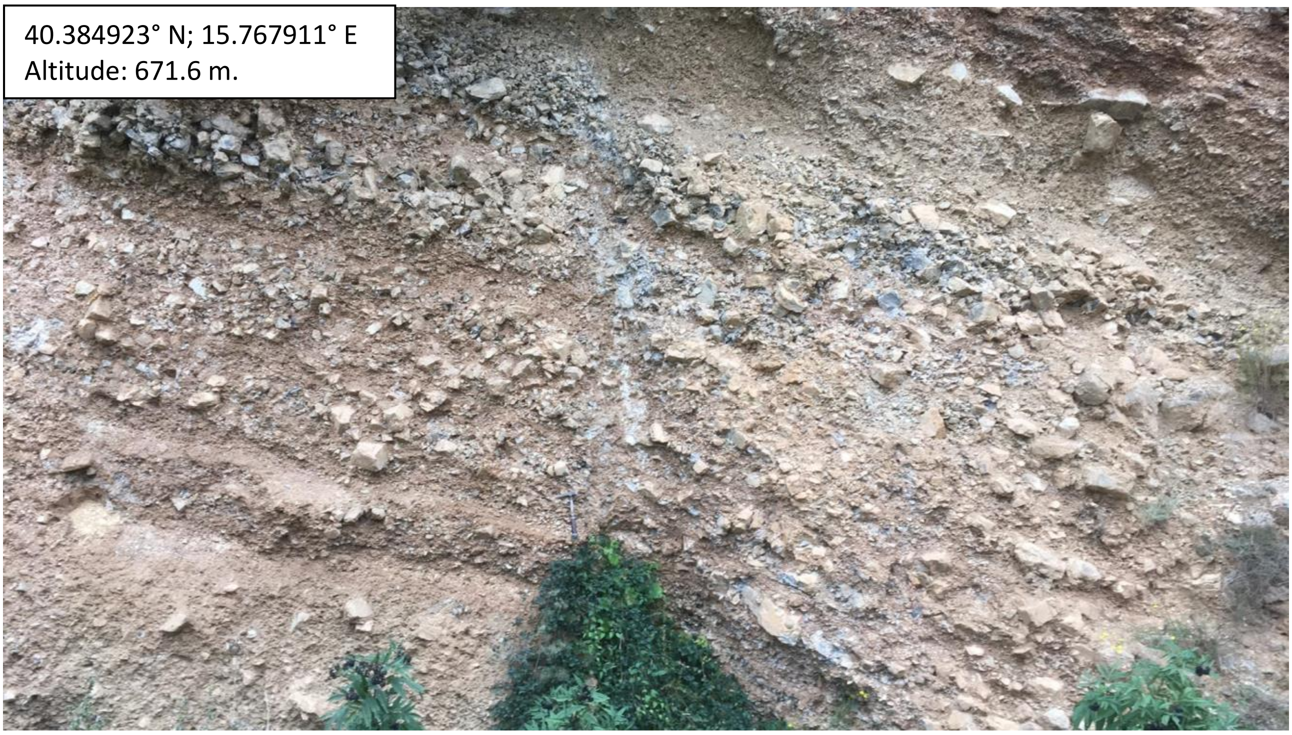

Figure 248.

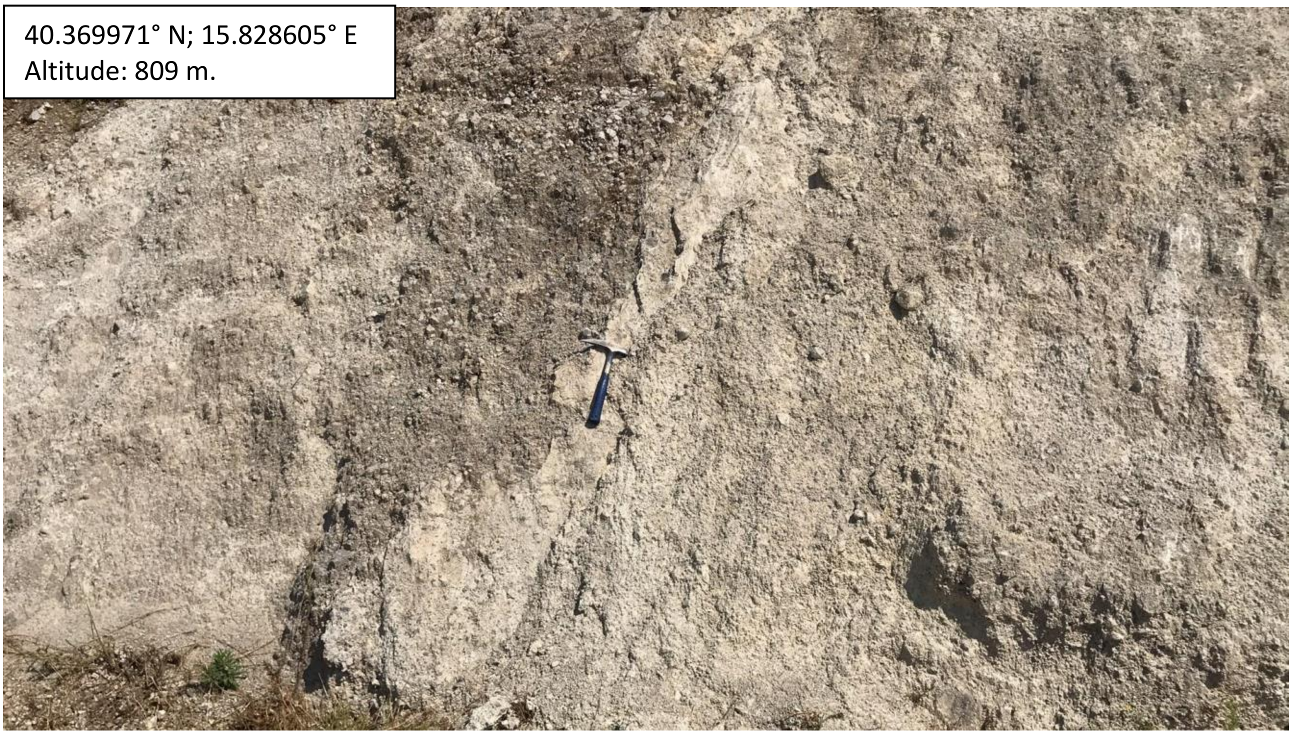

Figure 249.

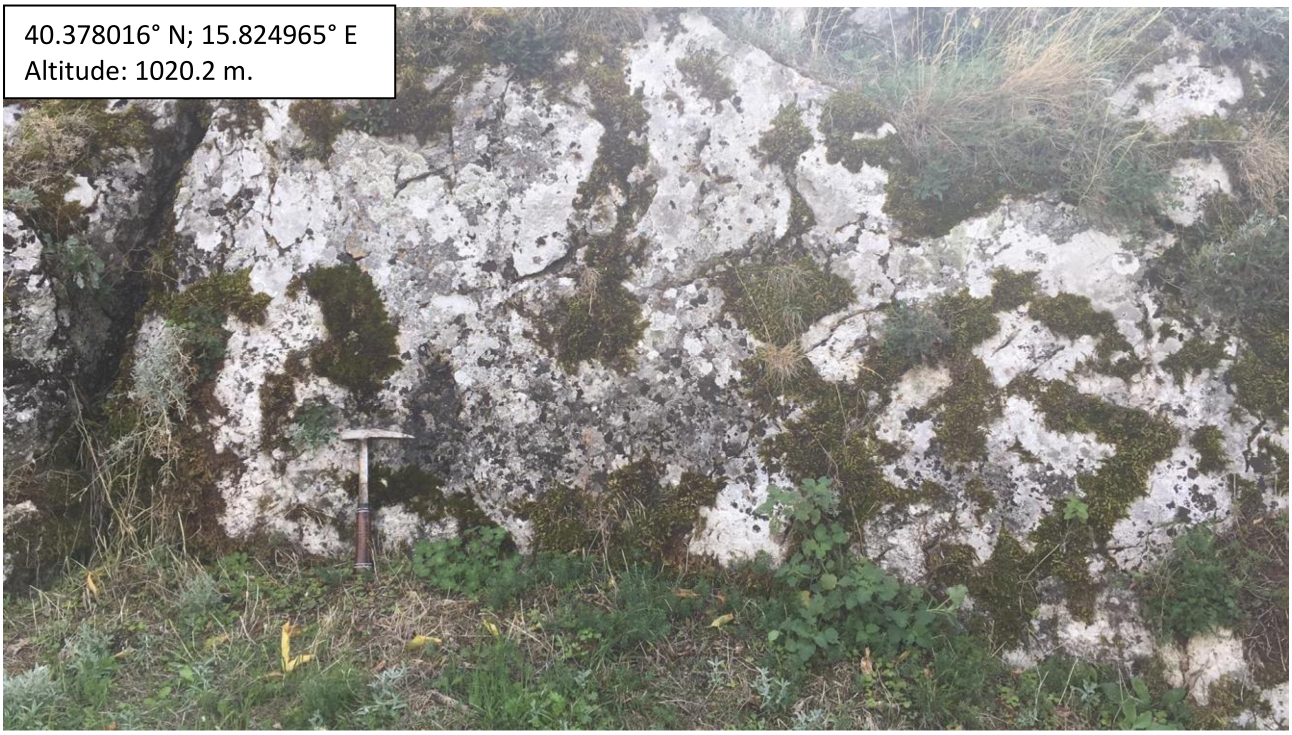

Figure 250.

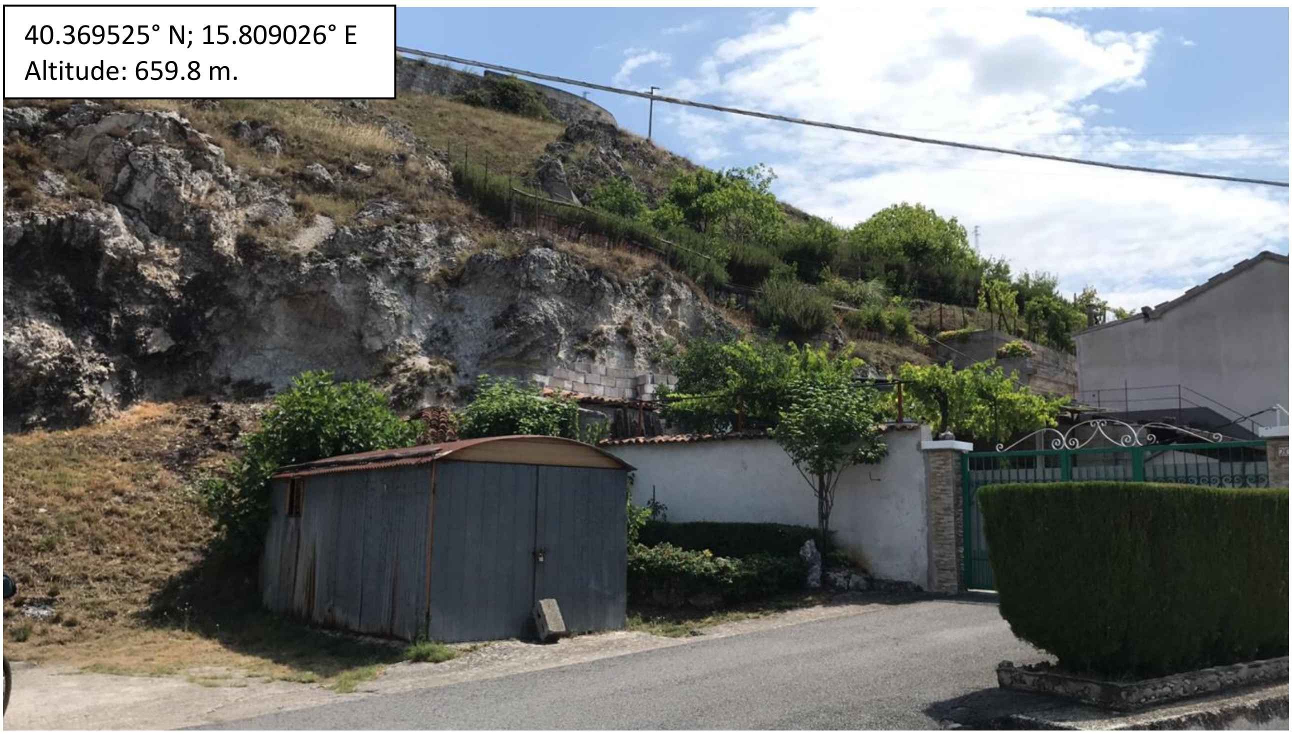

Figure 251.

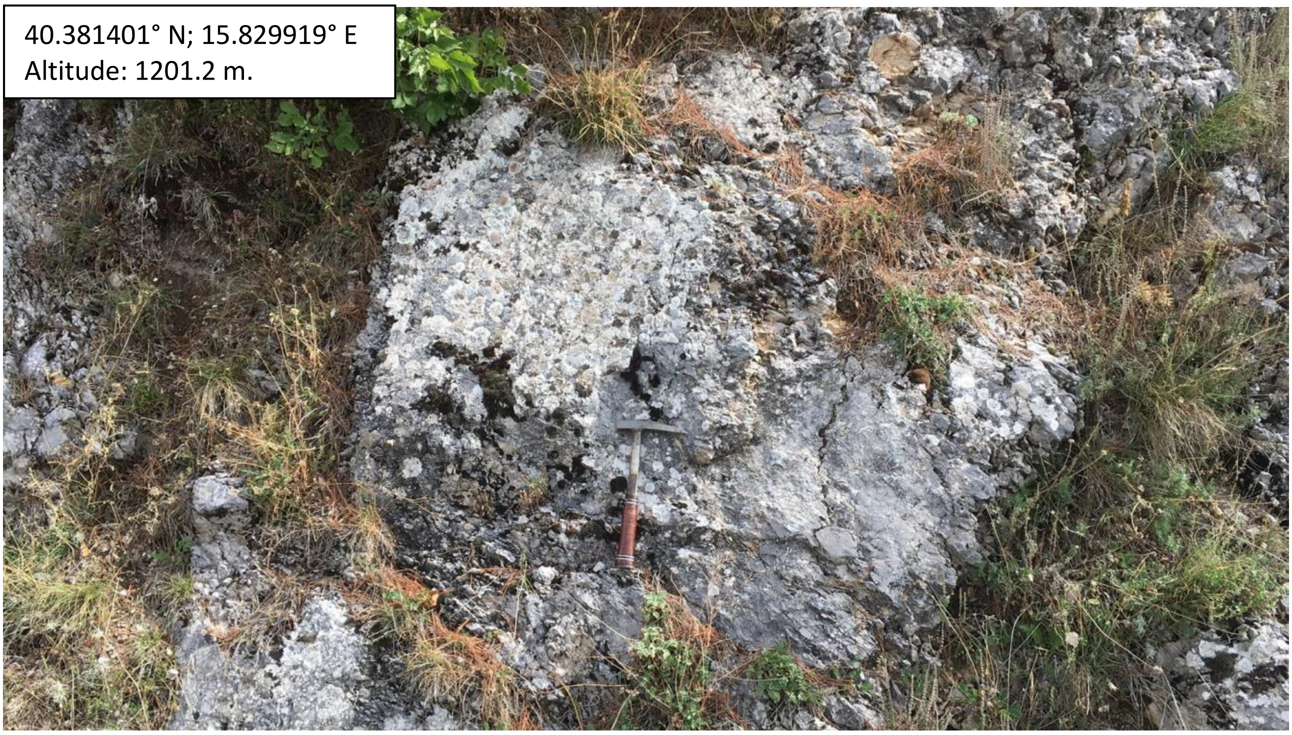

Figure 252.

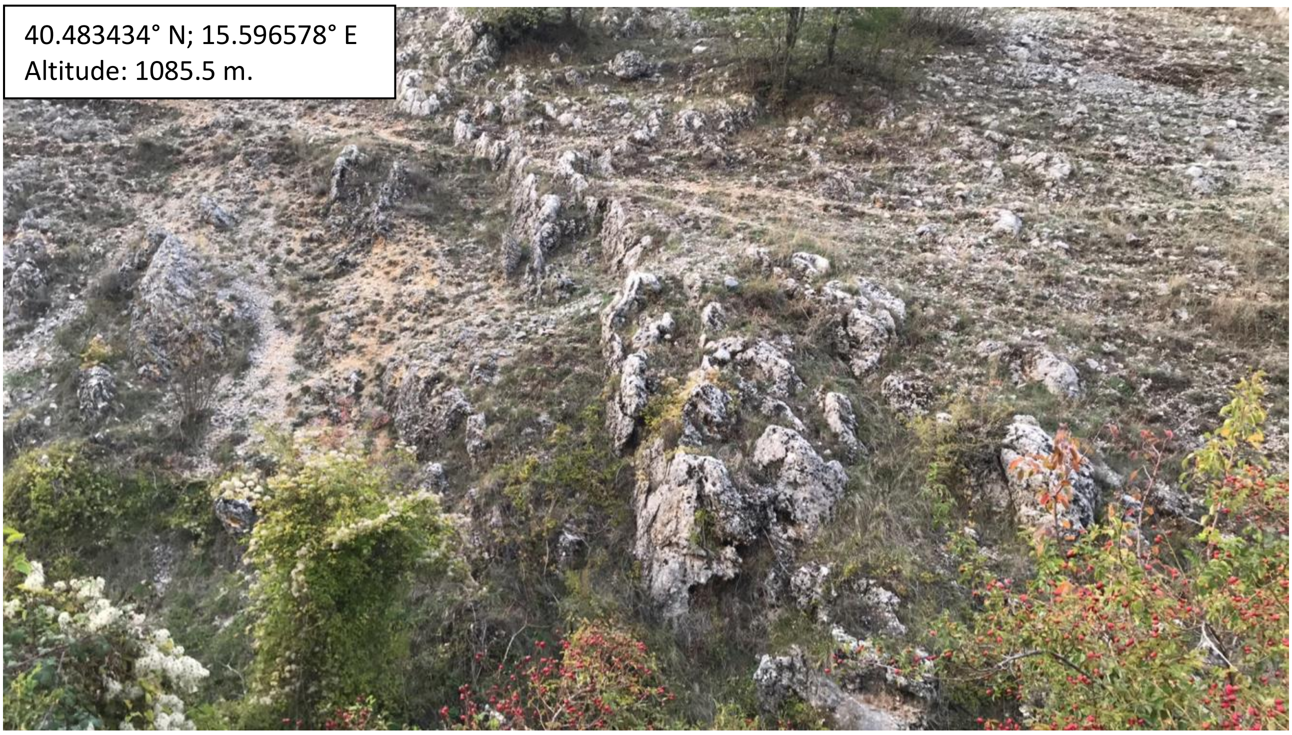

Figure 253.

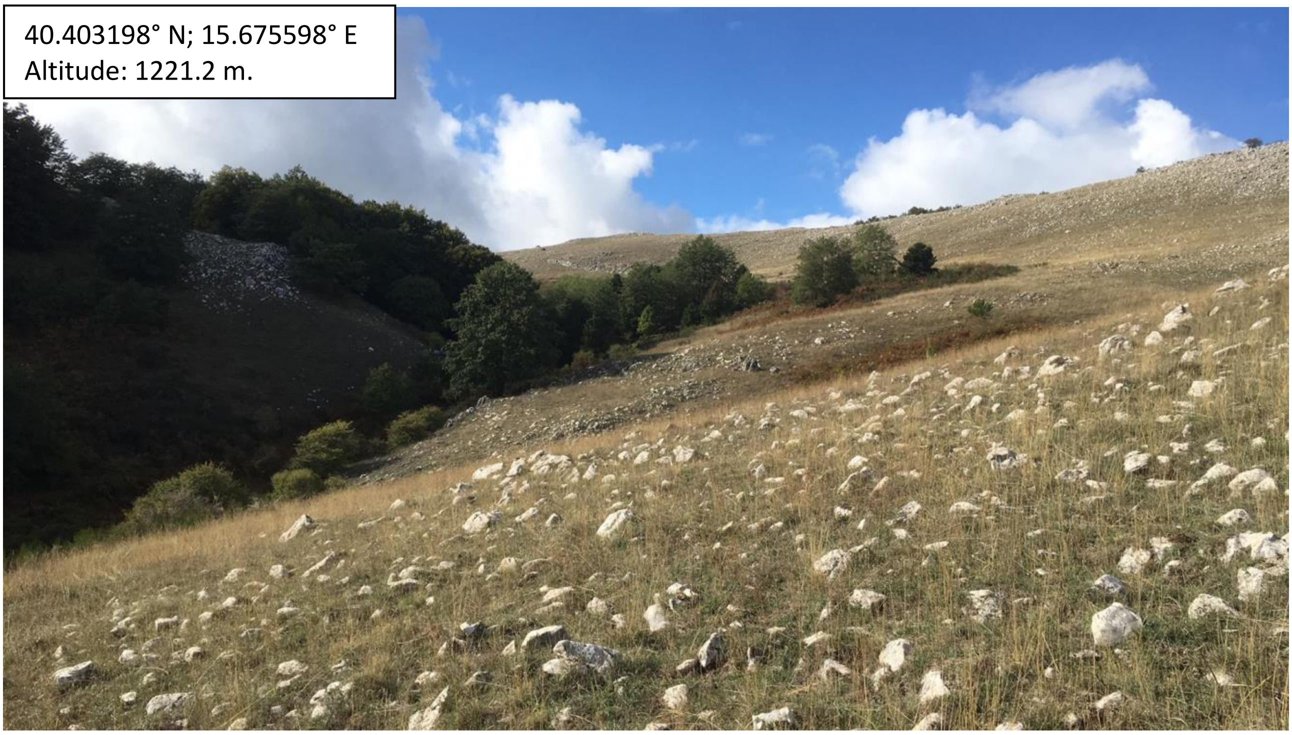

Figure 256.

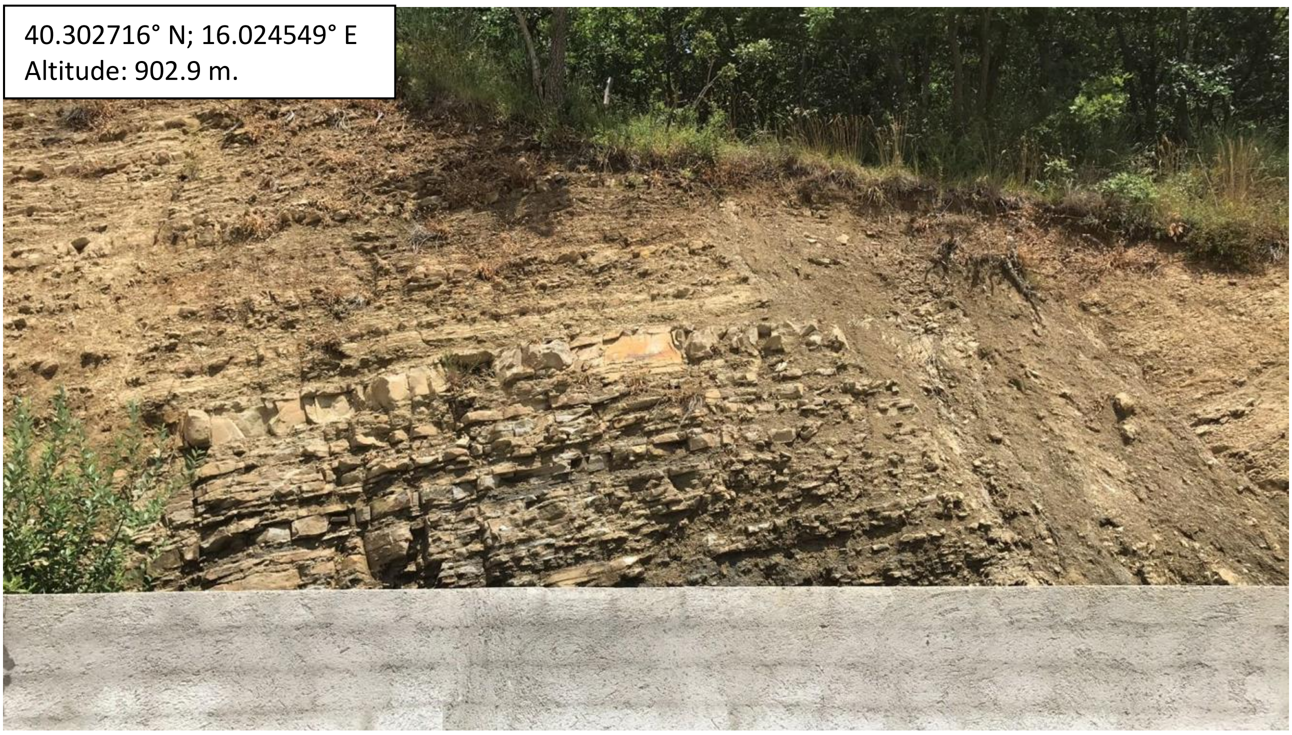

Figure 257.

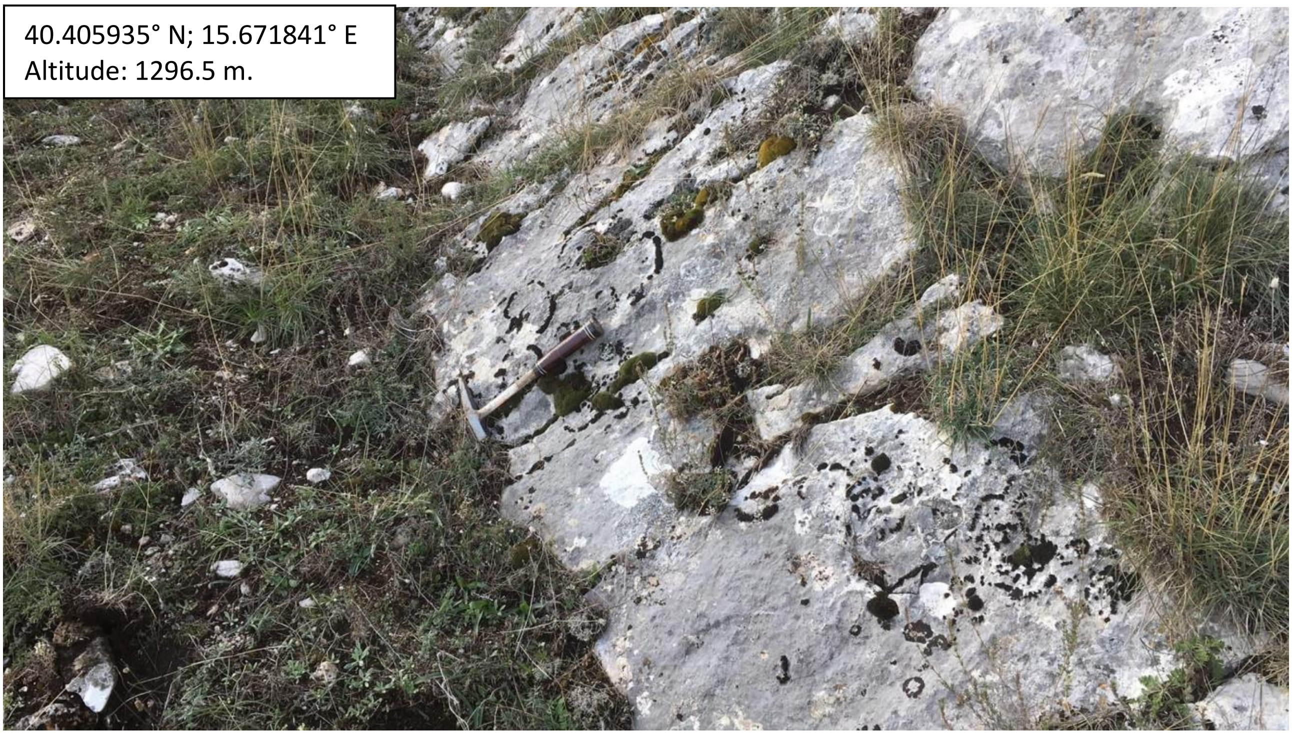

Figure 258.

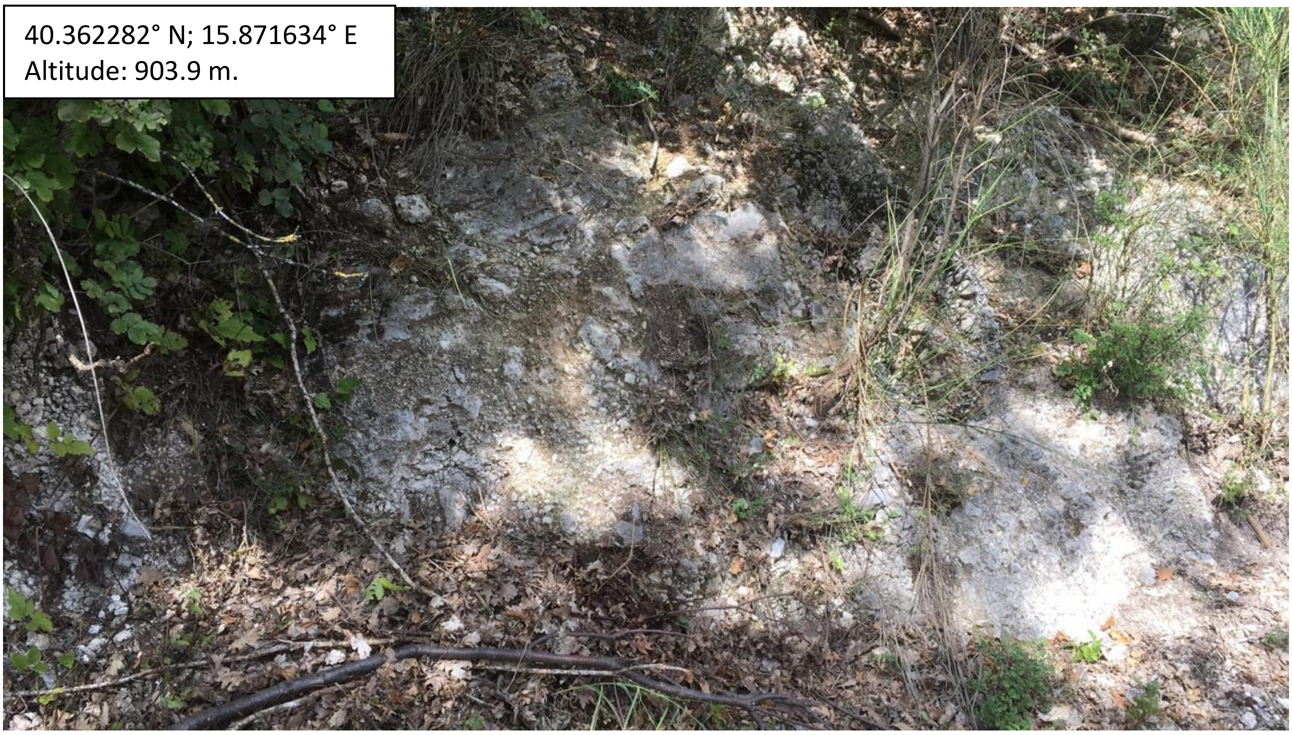

Figure 259

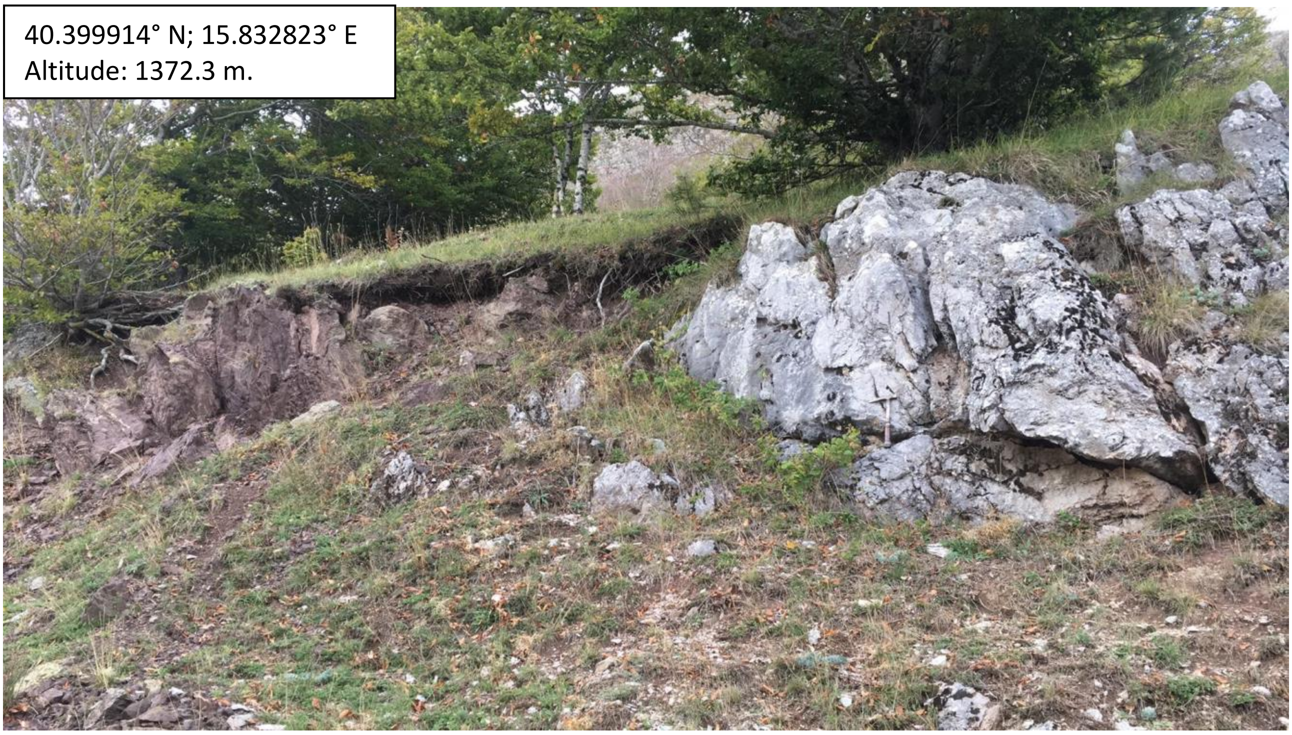

Figure 260.

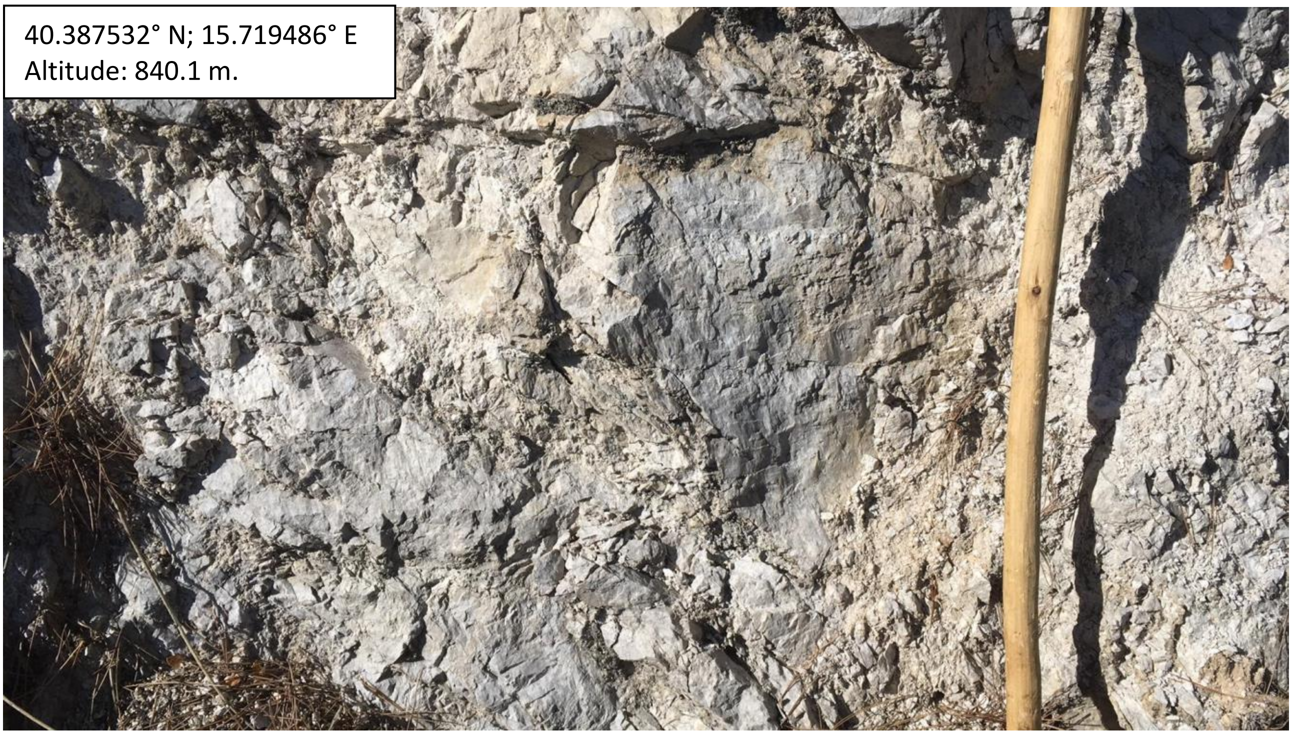

Figure 261.

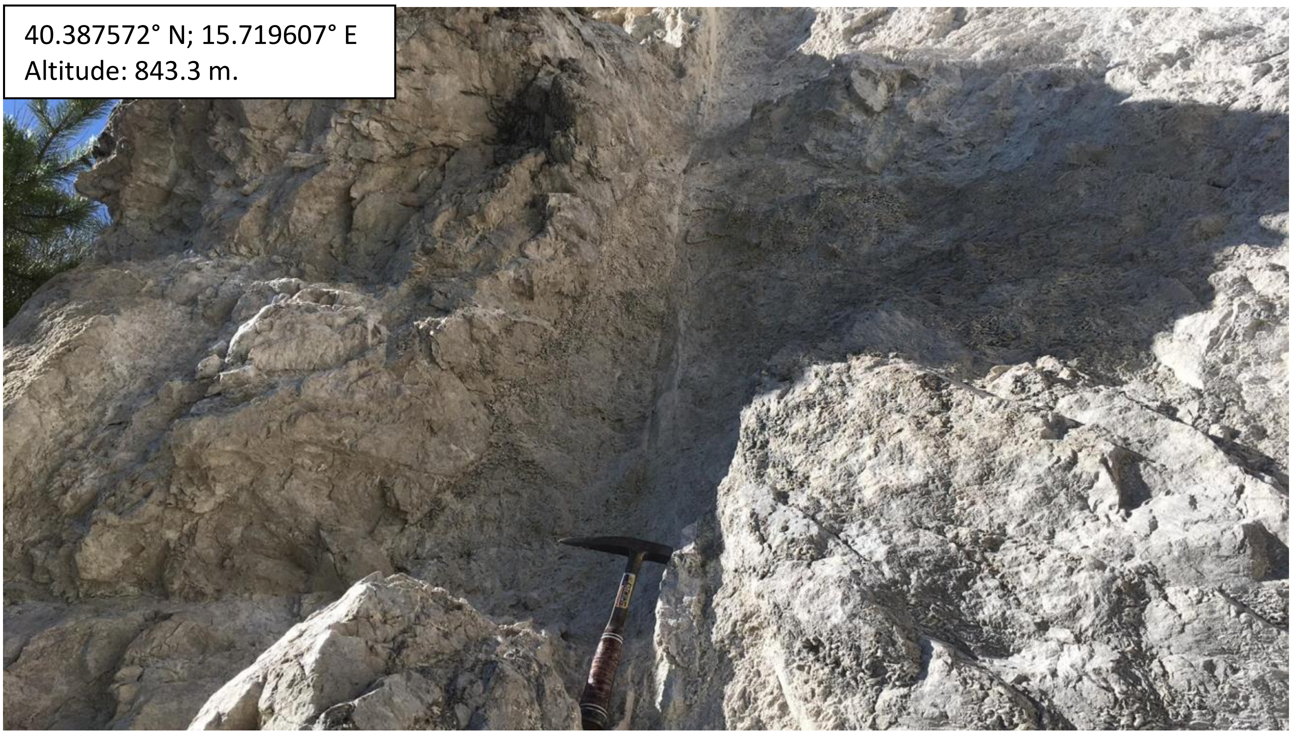

Figure 262.

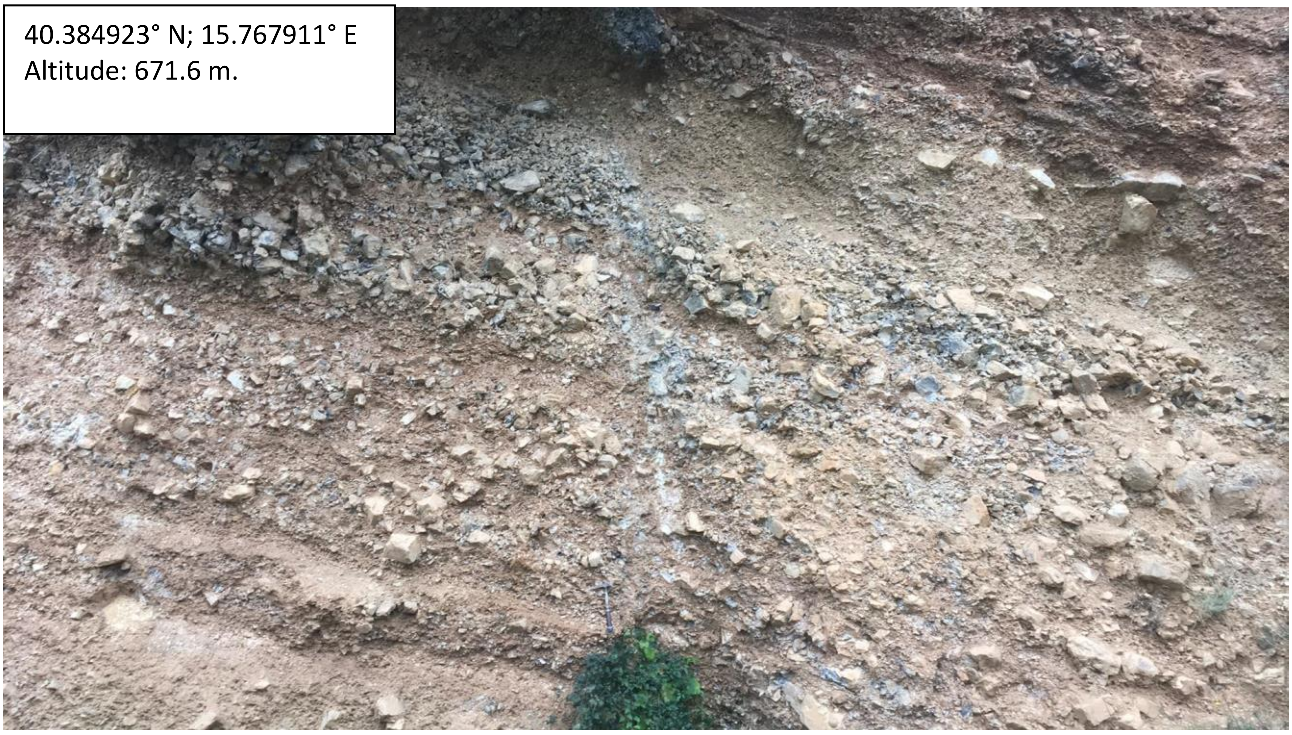

Figure 263.

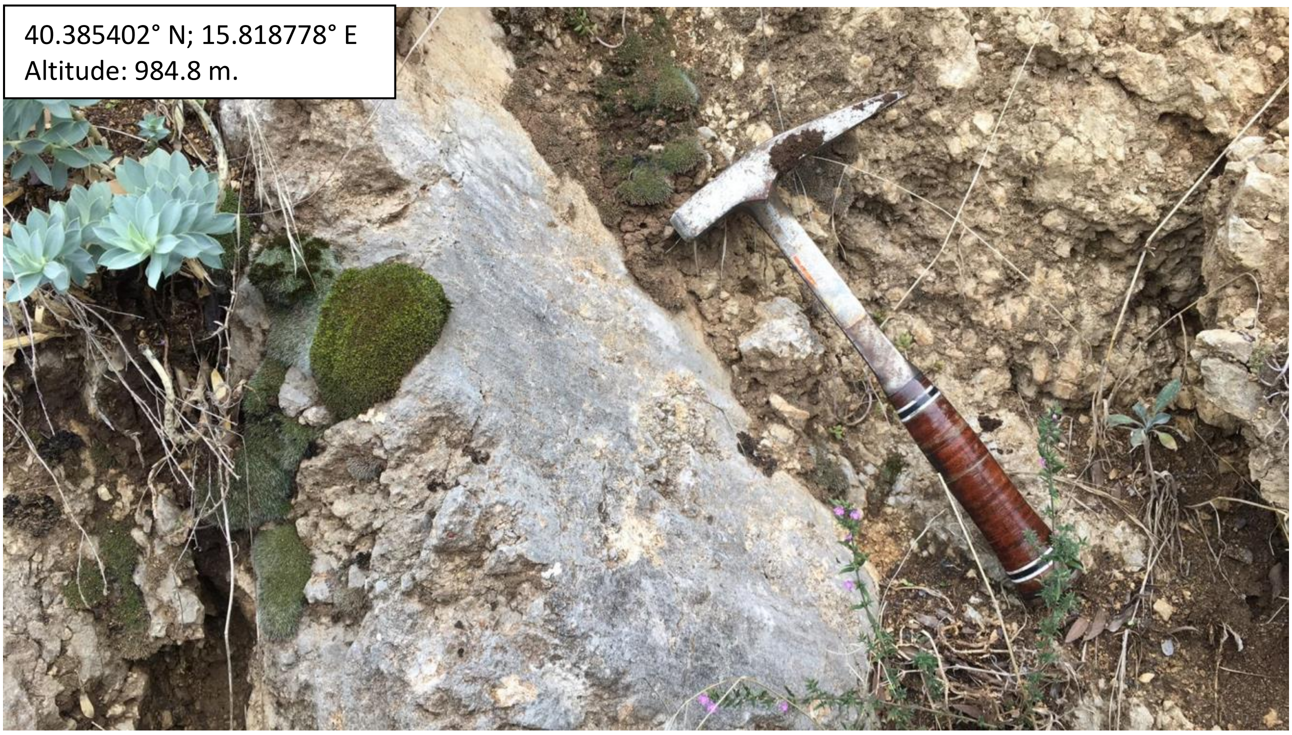

Figure 264.

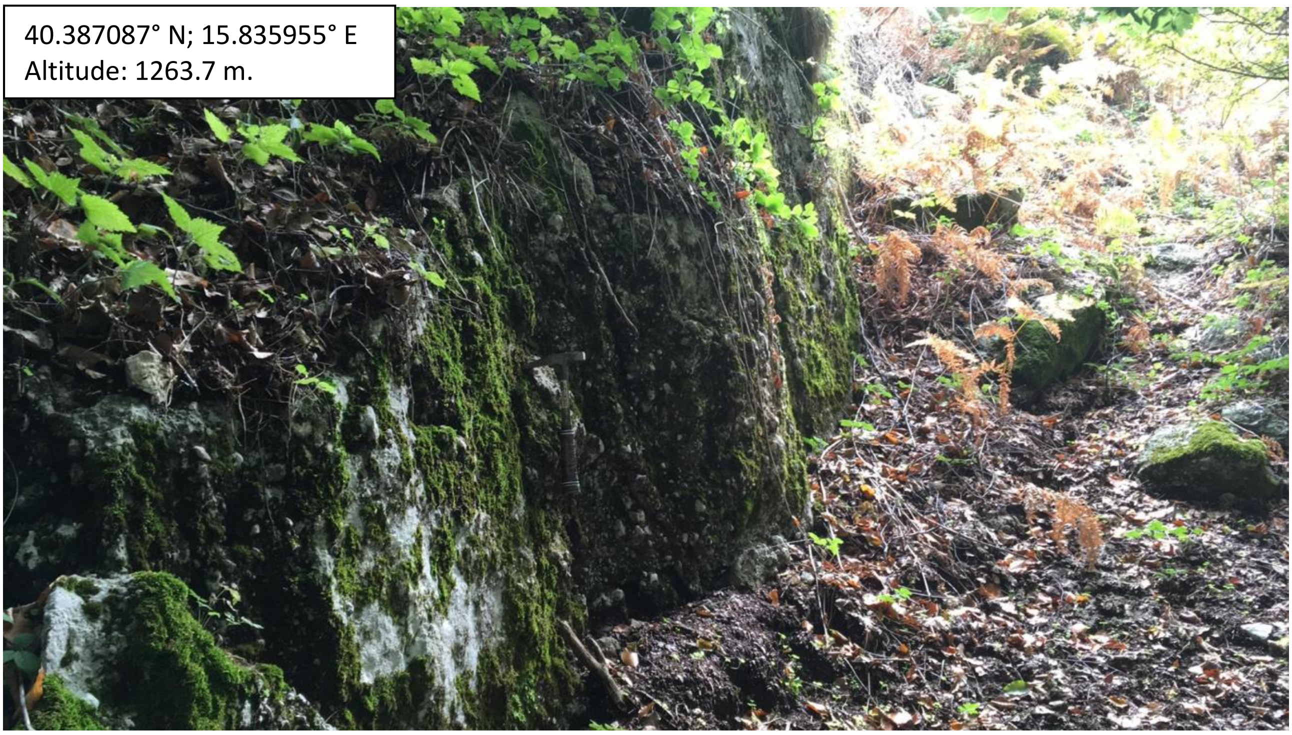

Figure 265.

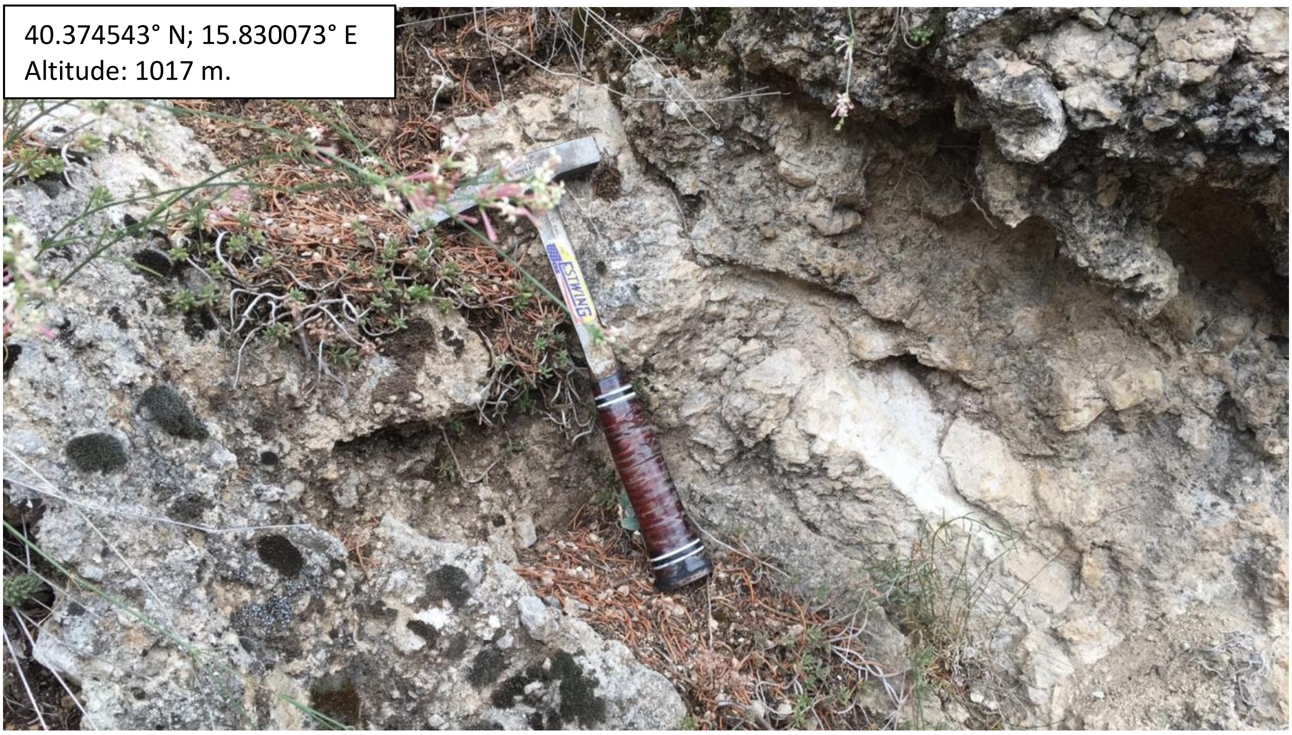

Figure 266.

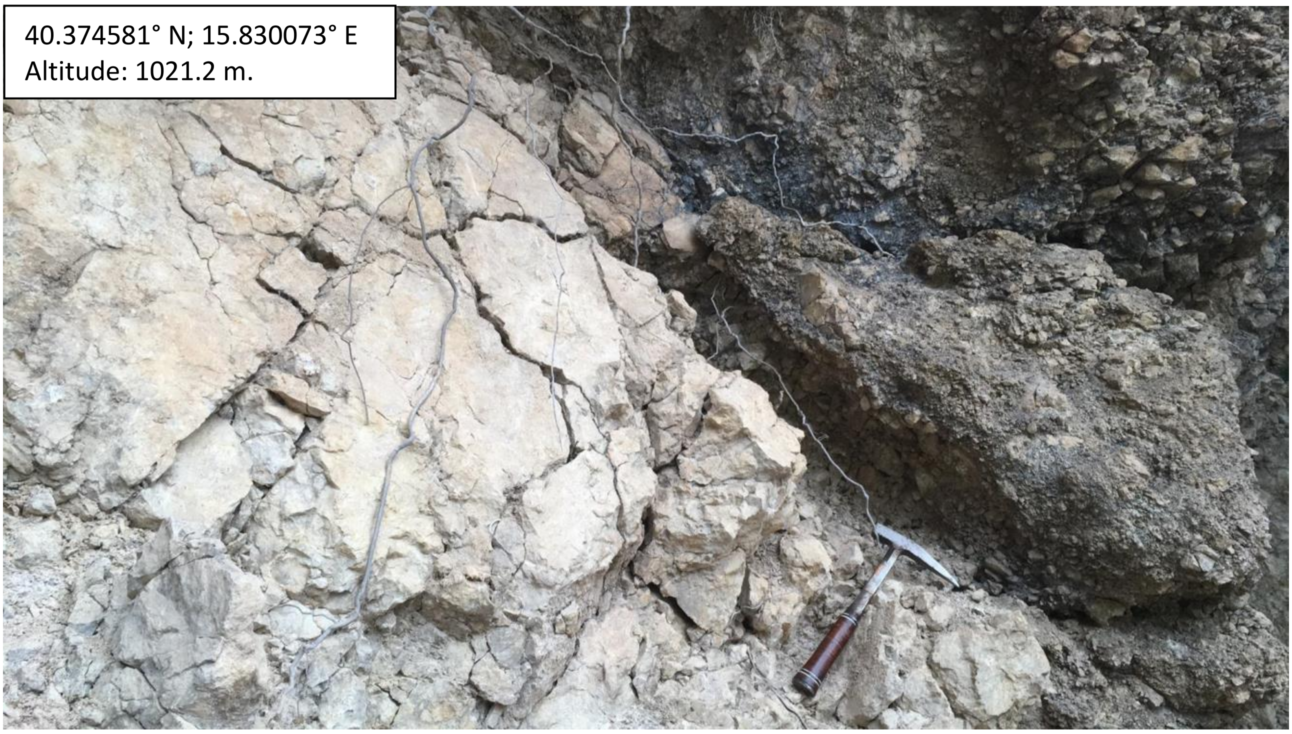

Figure 267.

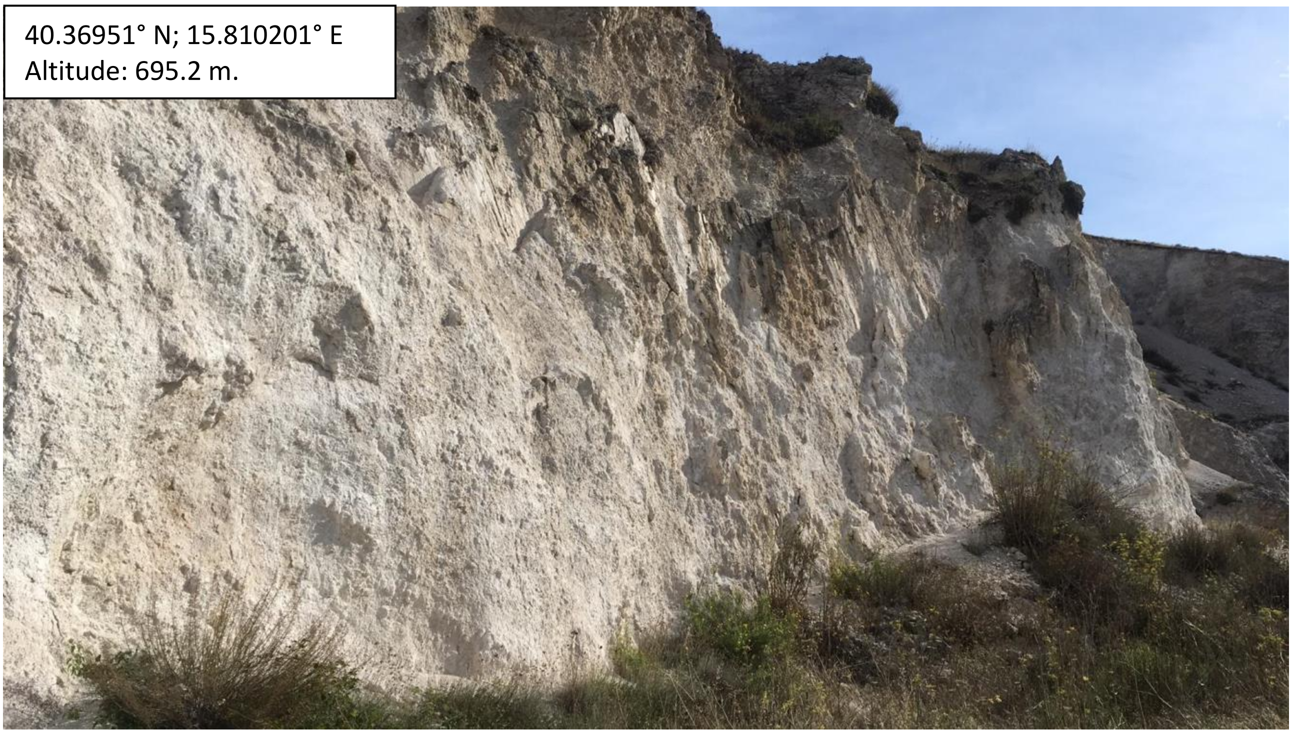

Figure 268.

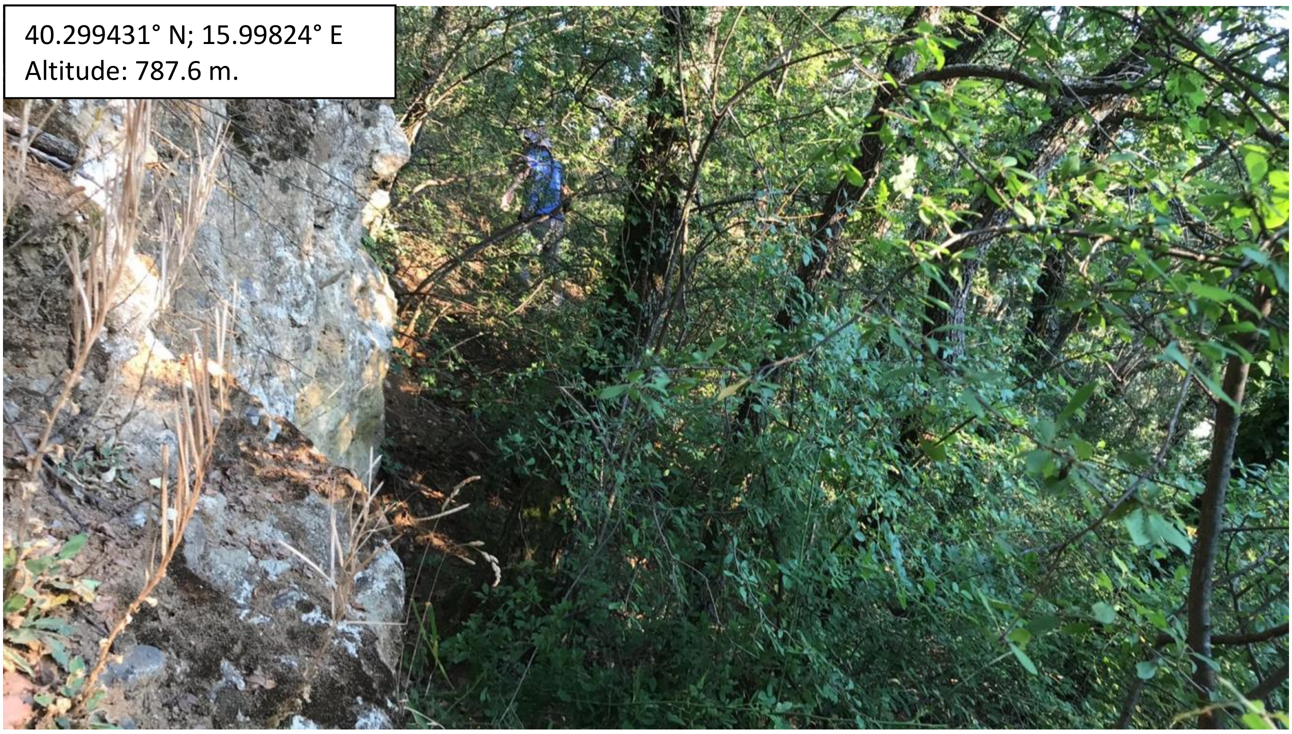

Figure 270.

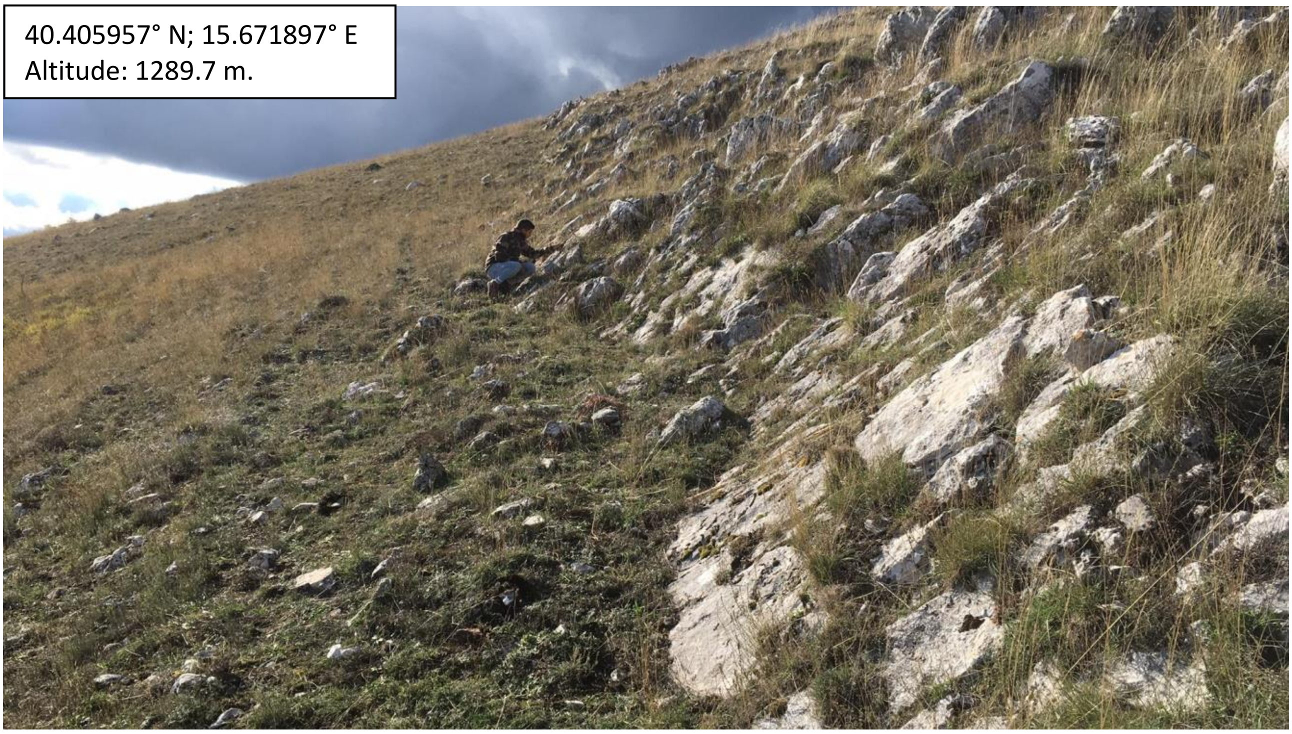

Figure 271.

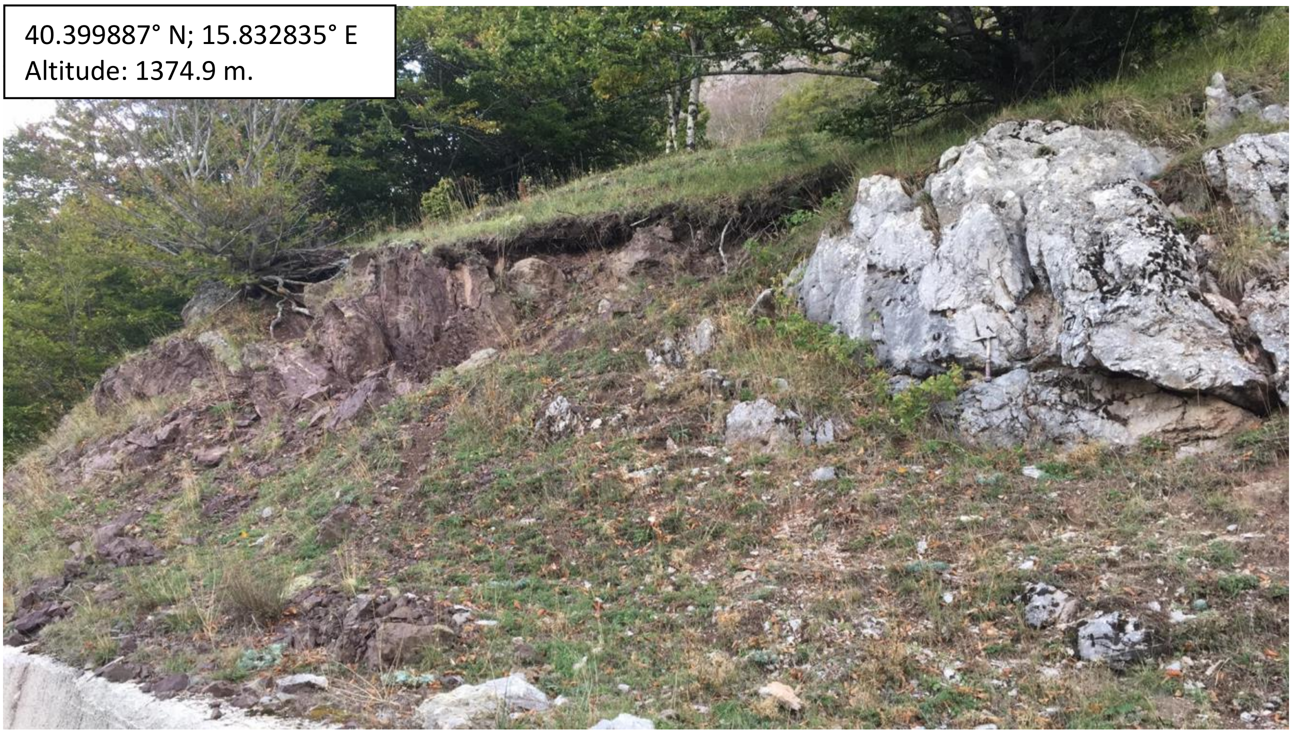

Figure 272.

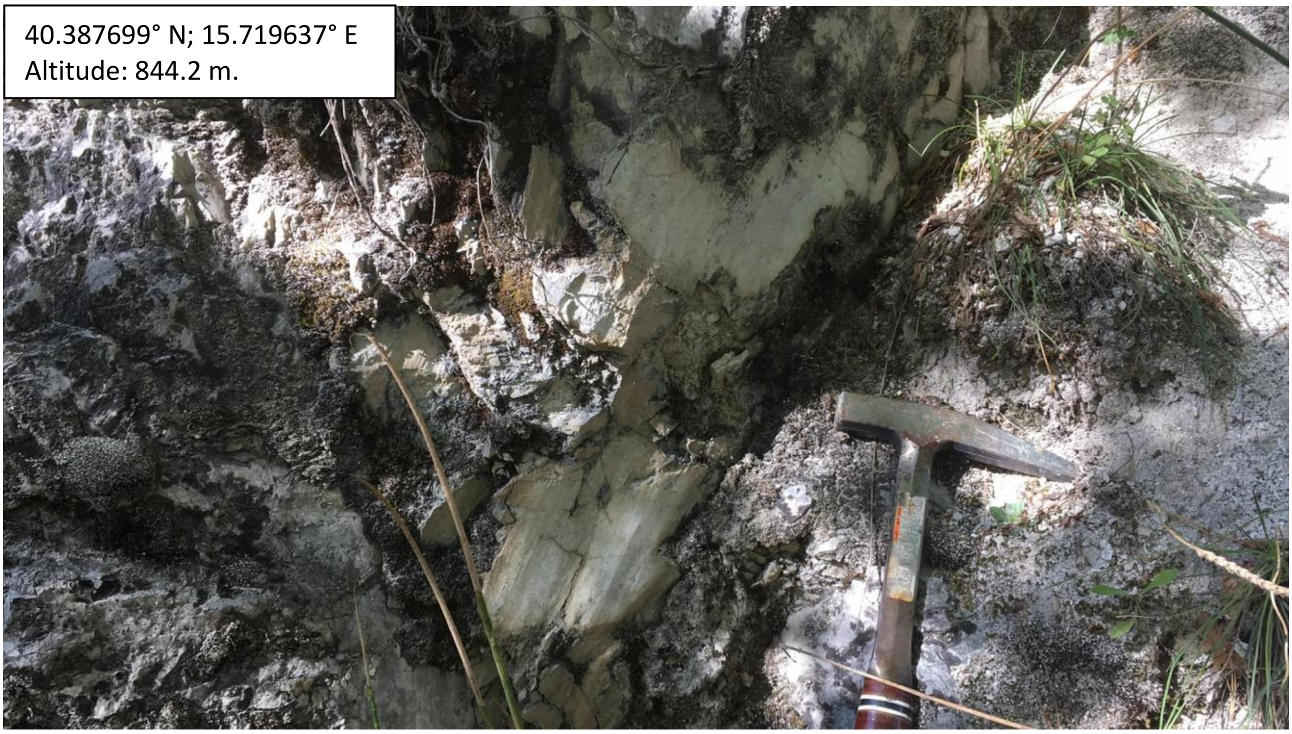

Figure 273.

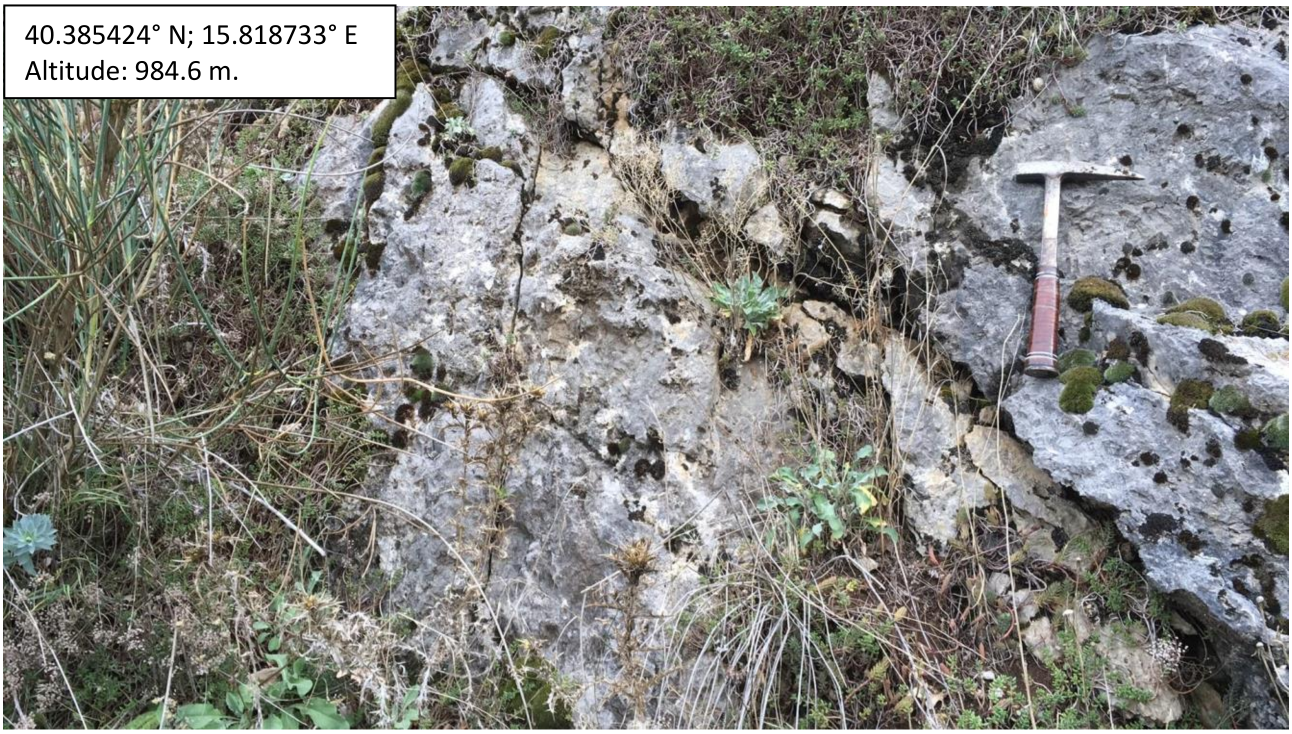

Figure 274.

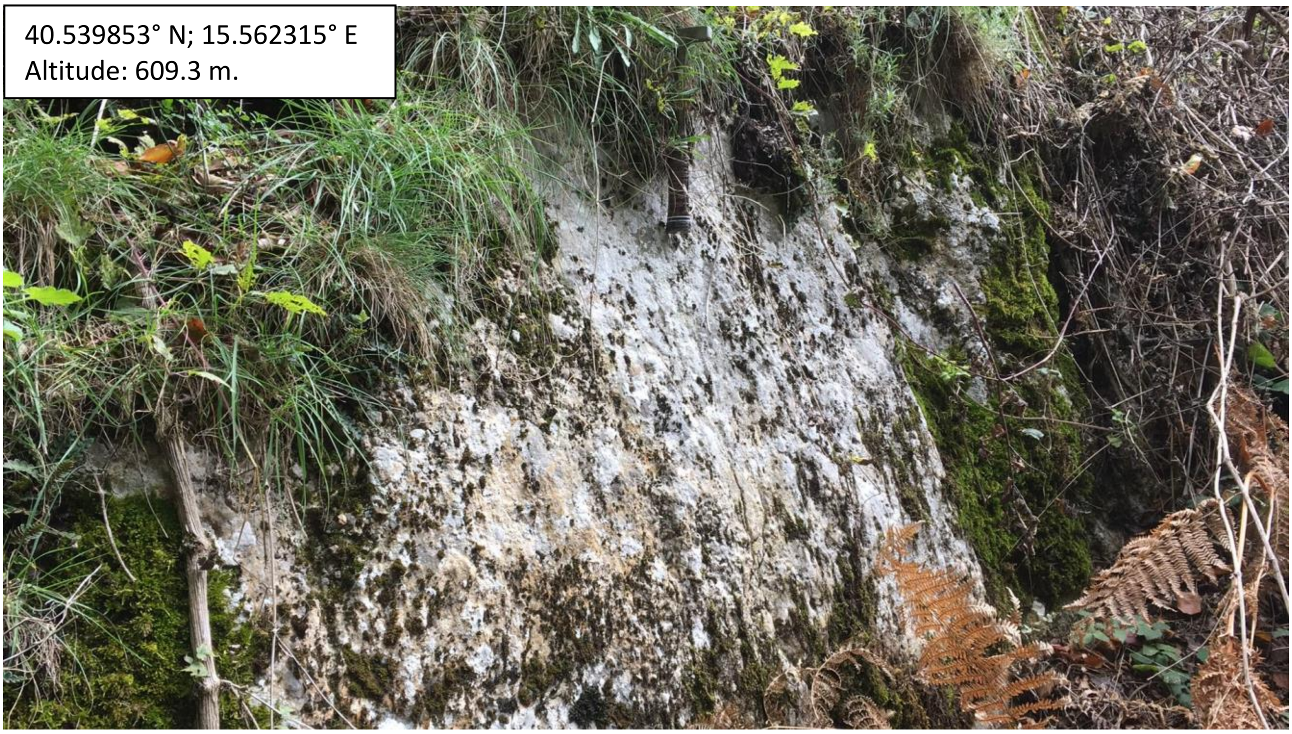

Figure 275.

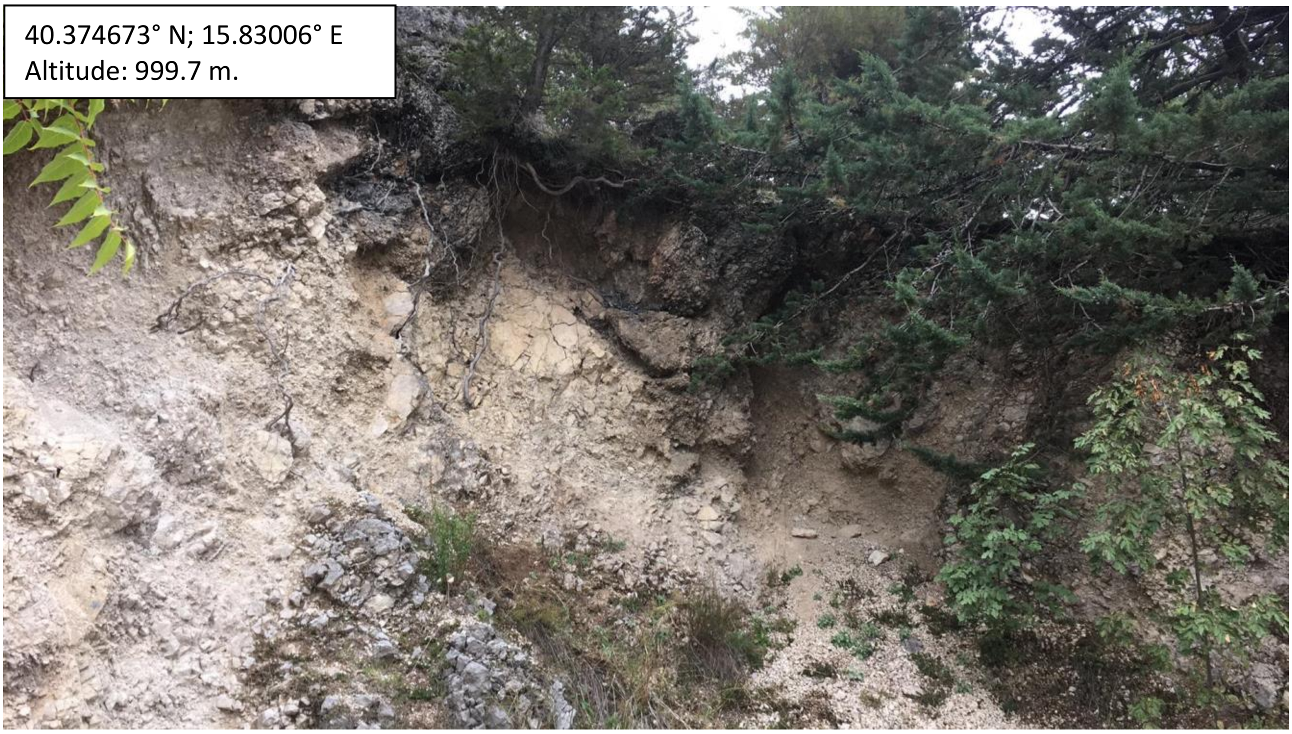

Figure 276.

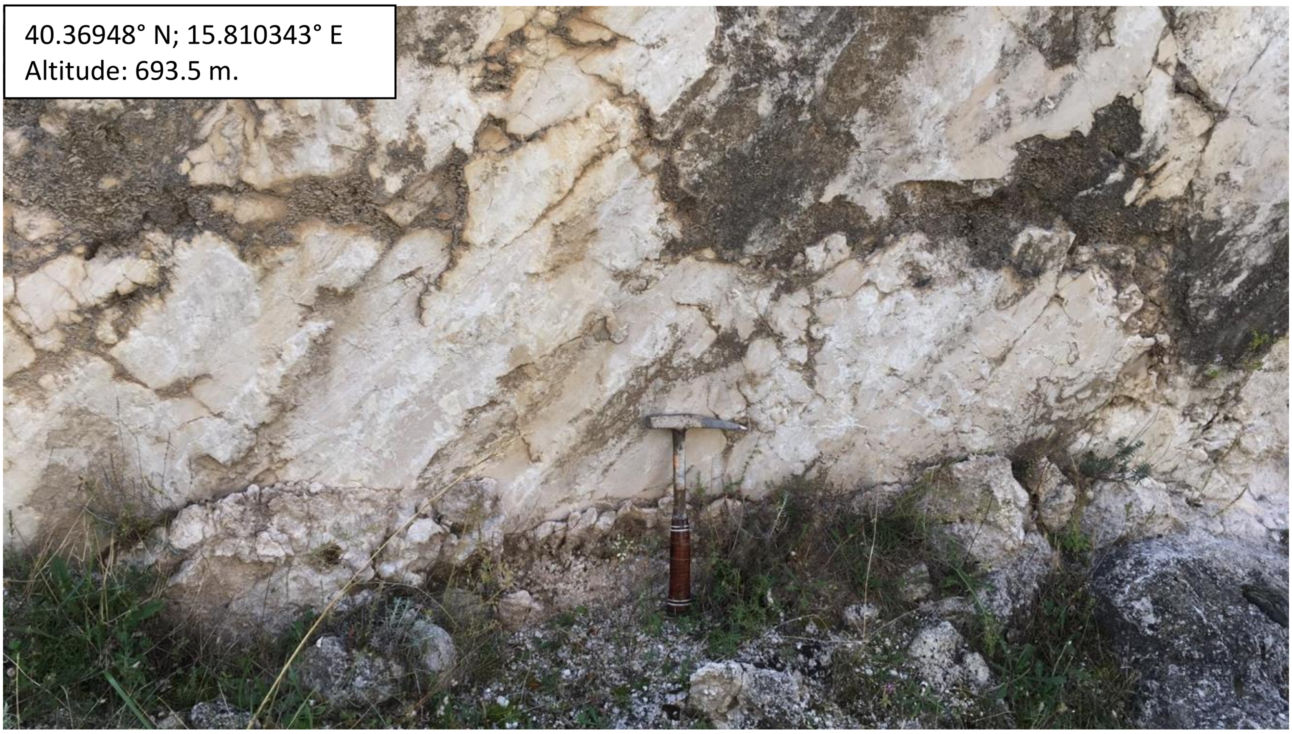

Figure 277.

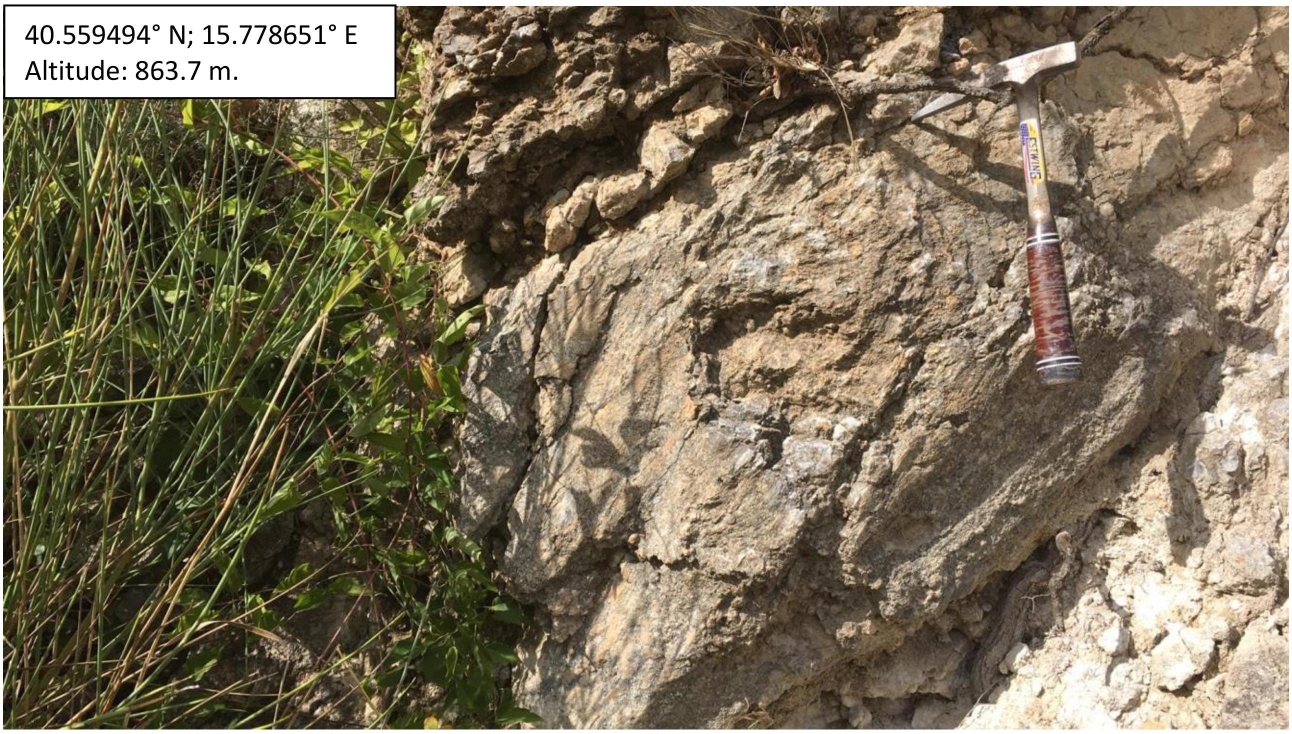

Figure 278.

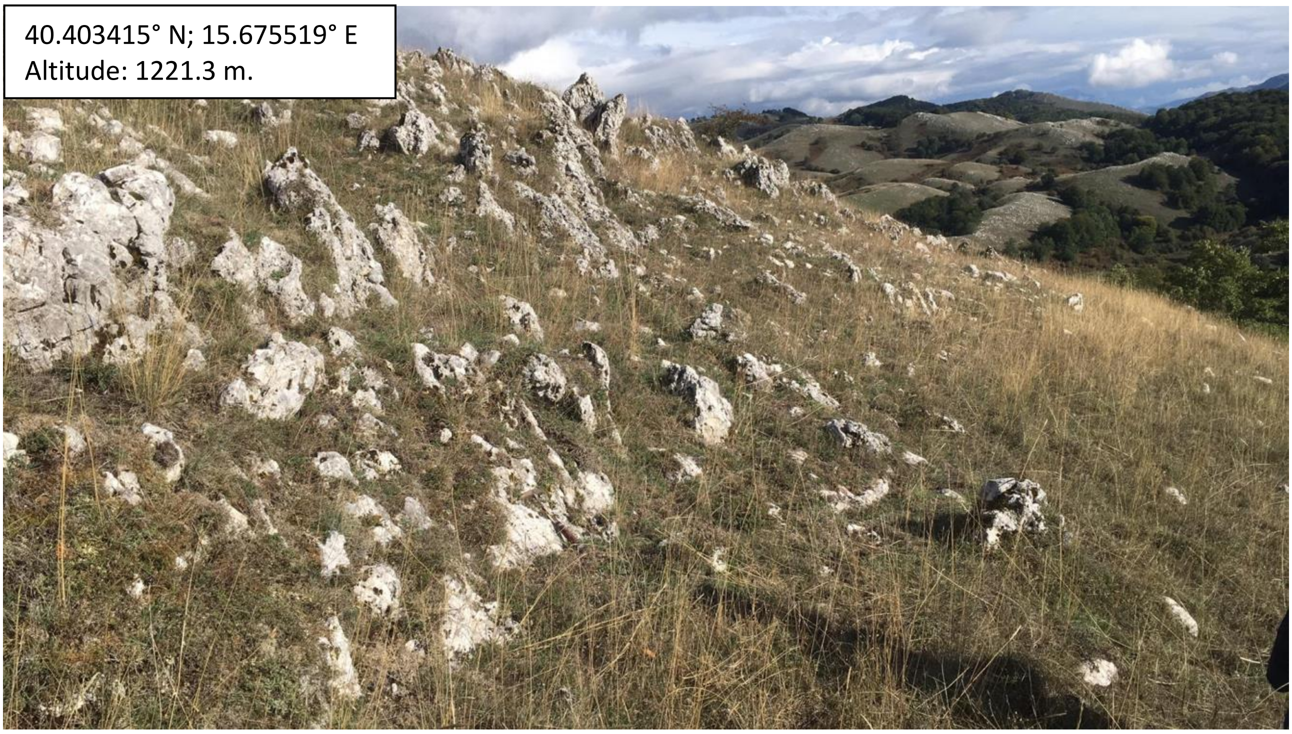

Figure 279.

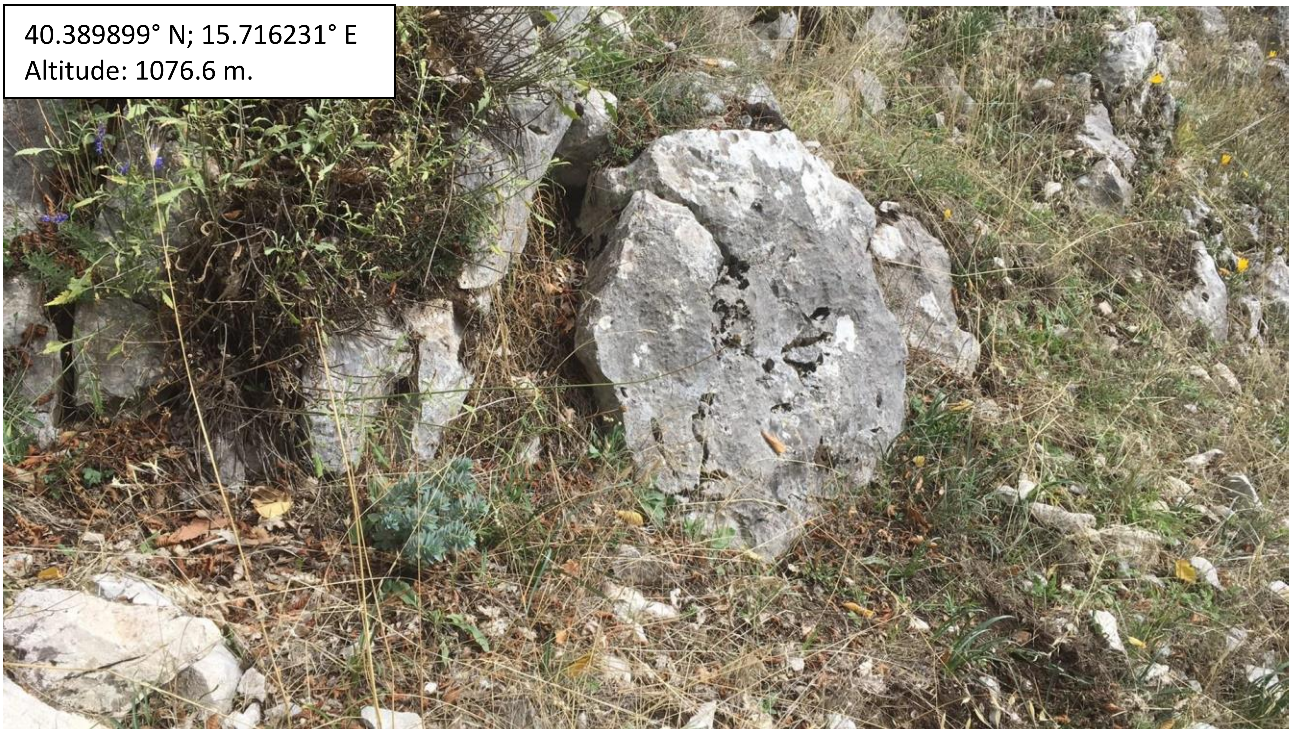

Figure 282.

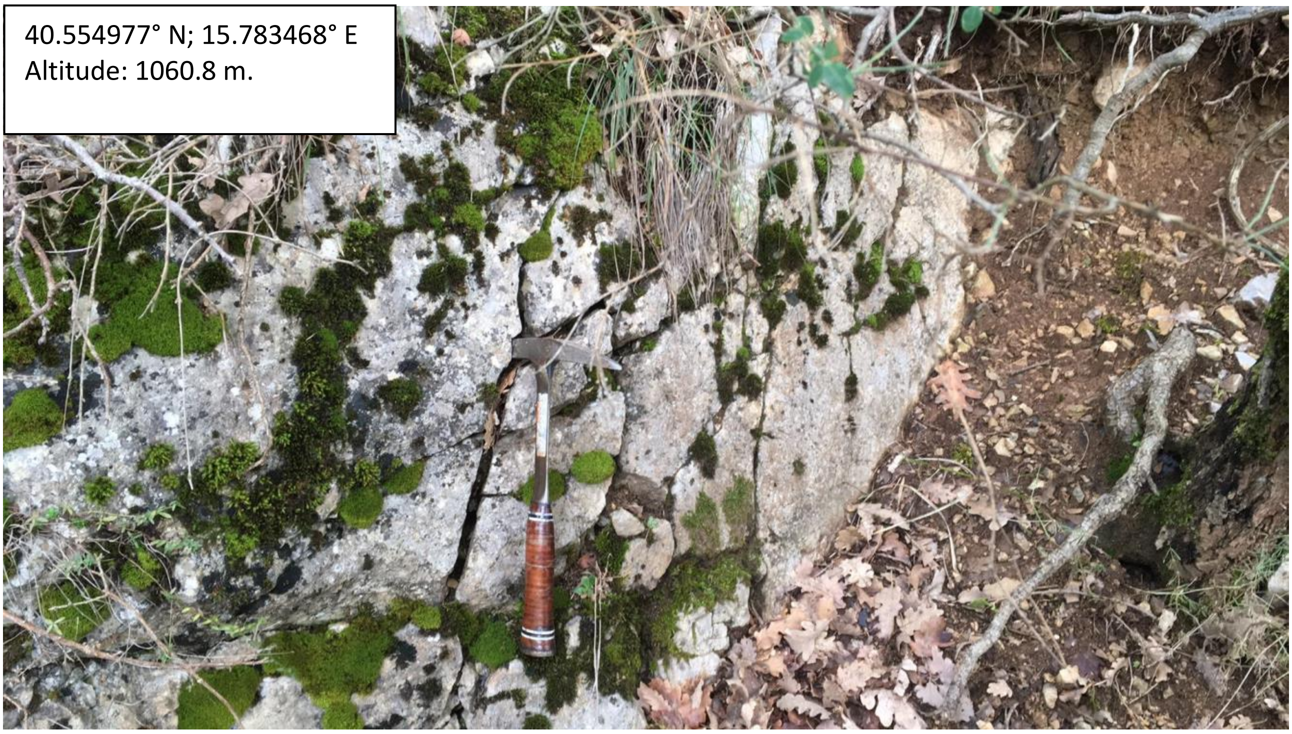

Figure 285.

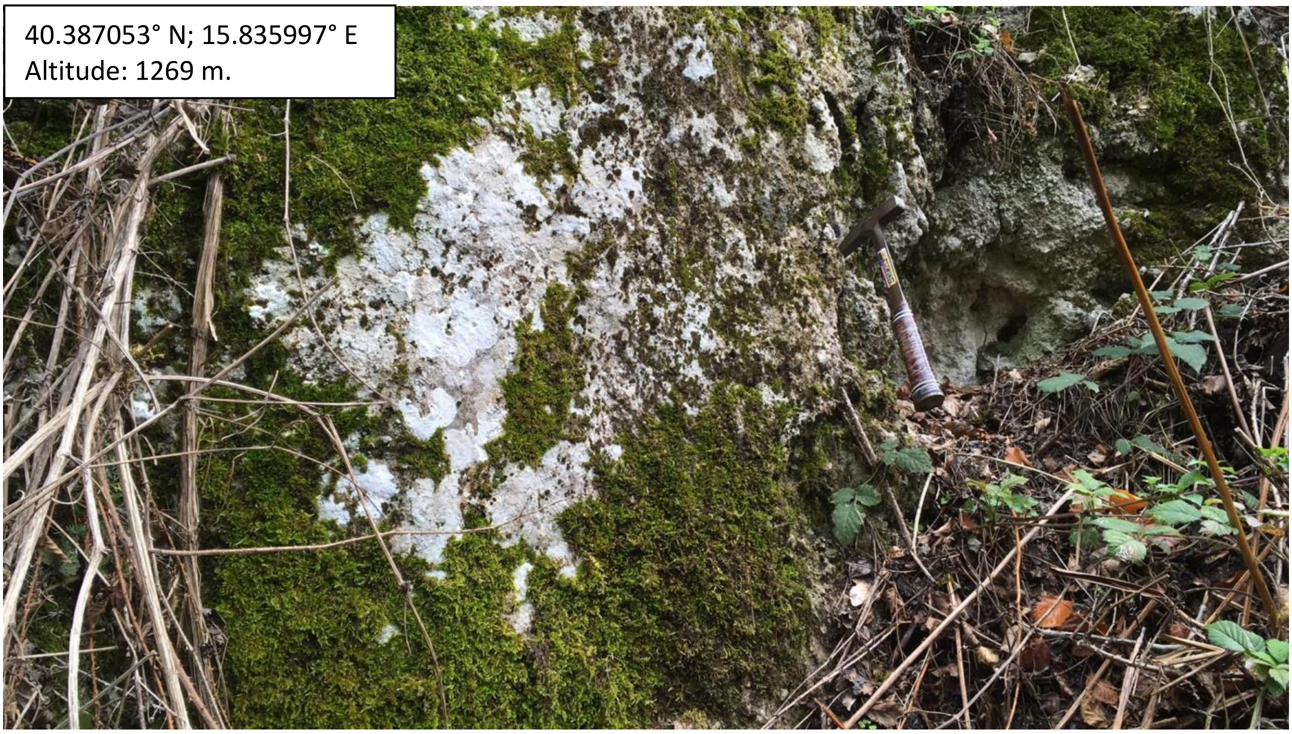

Figure 286.

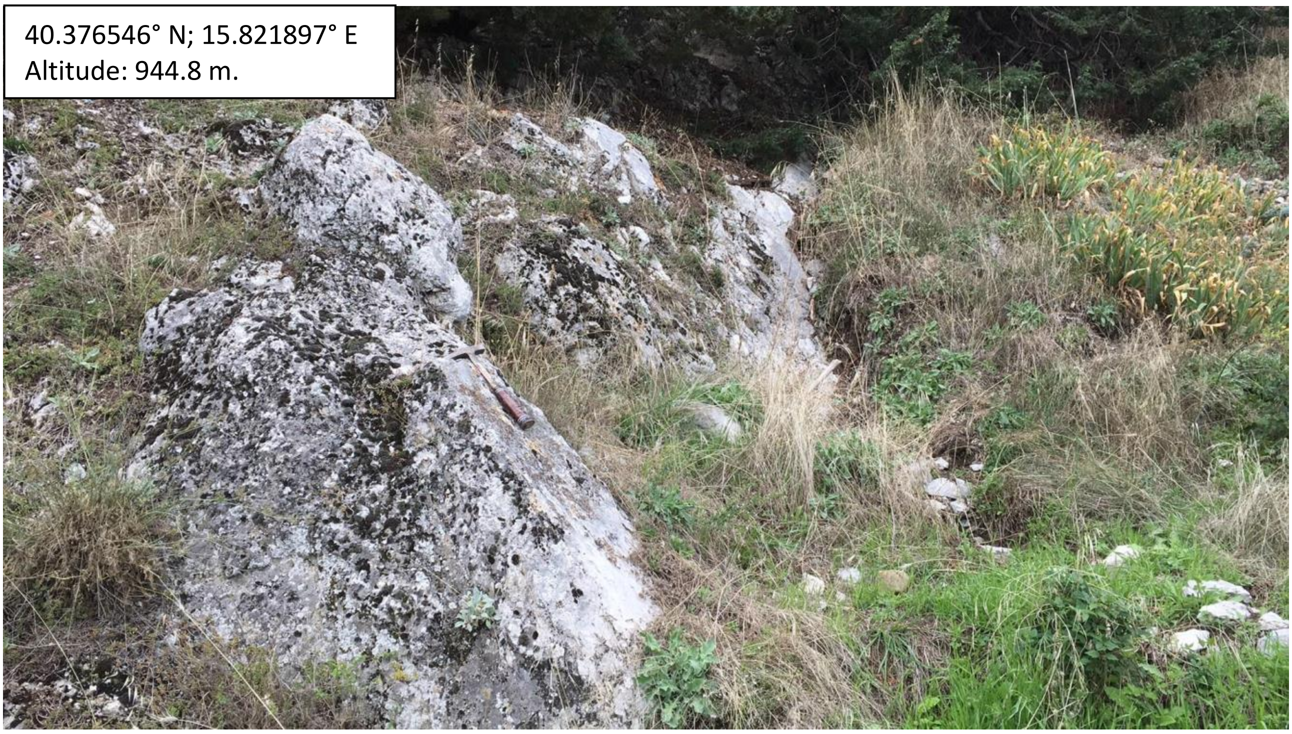

Figure 287.

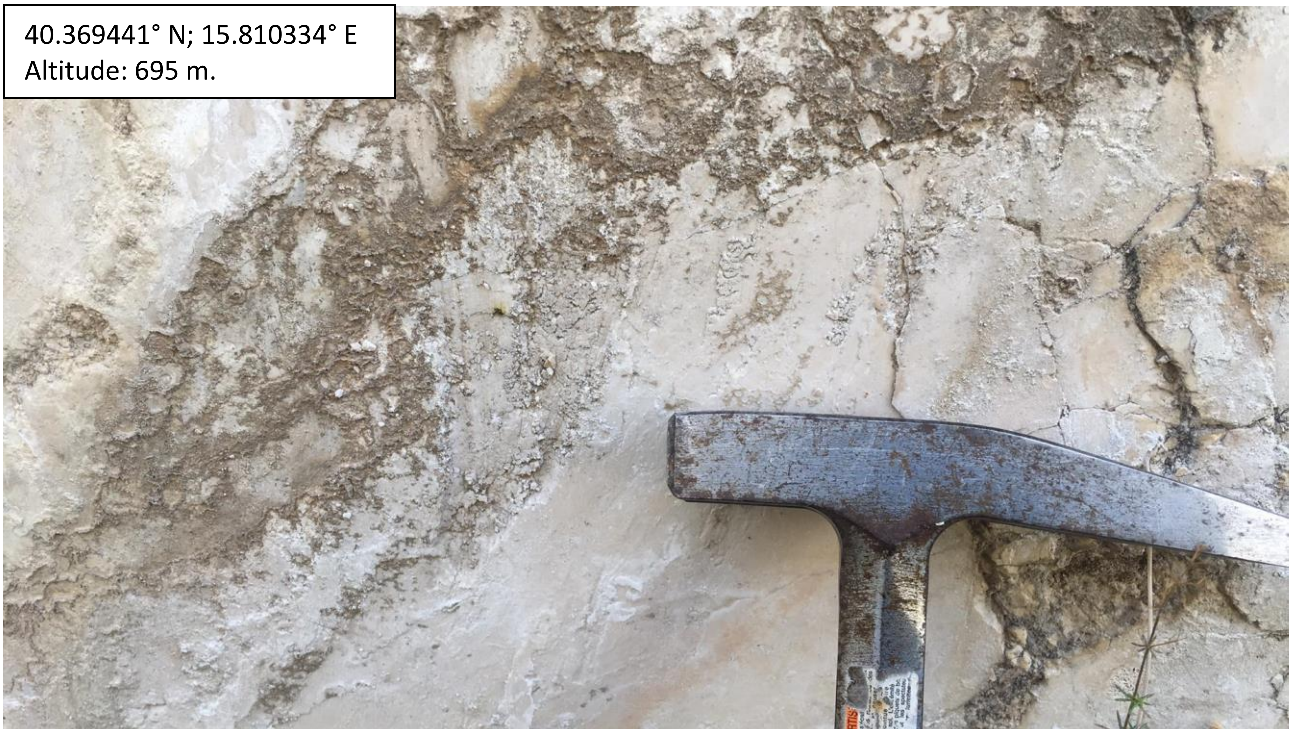

Figure 288.

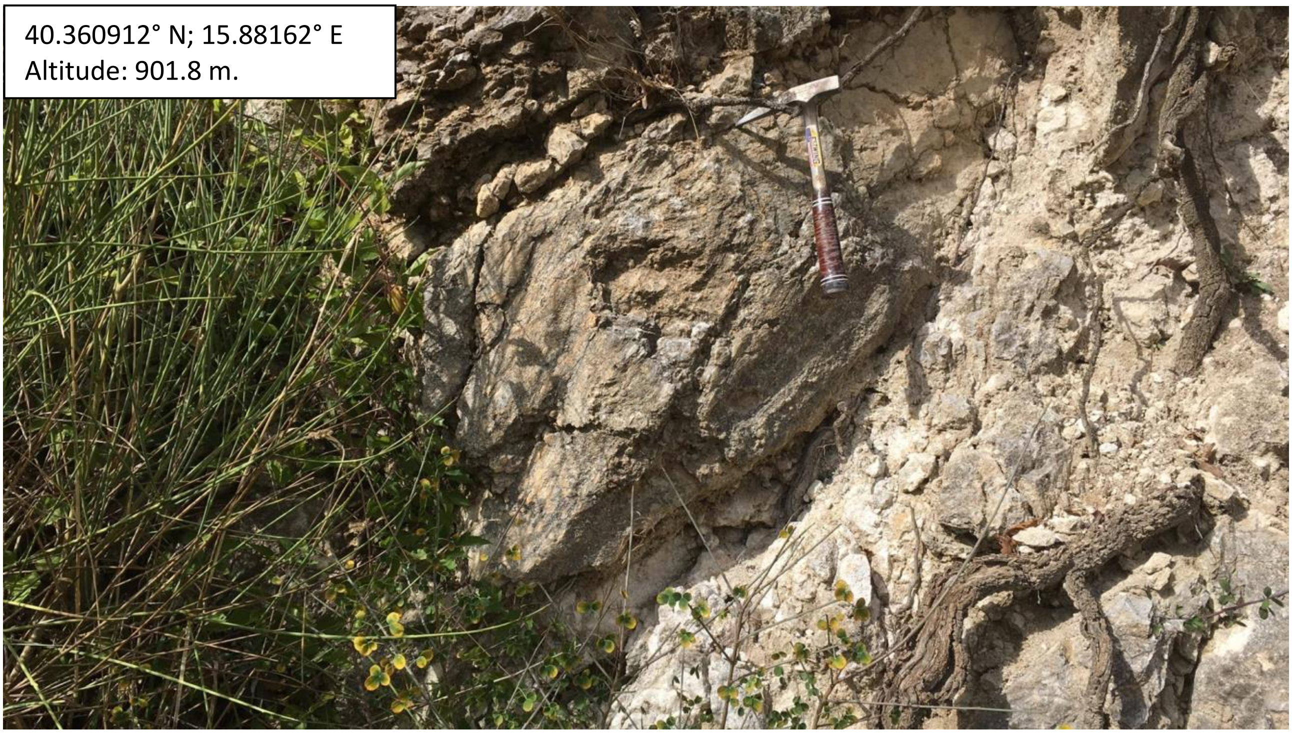

Figure 289

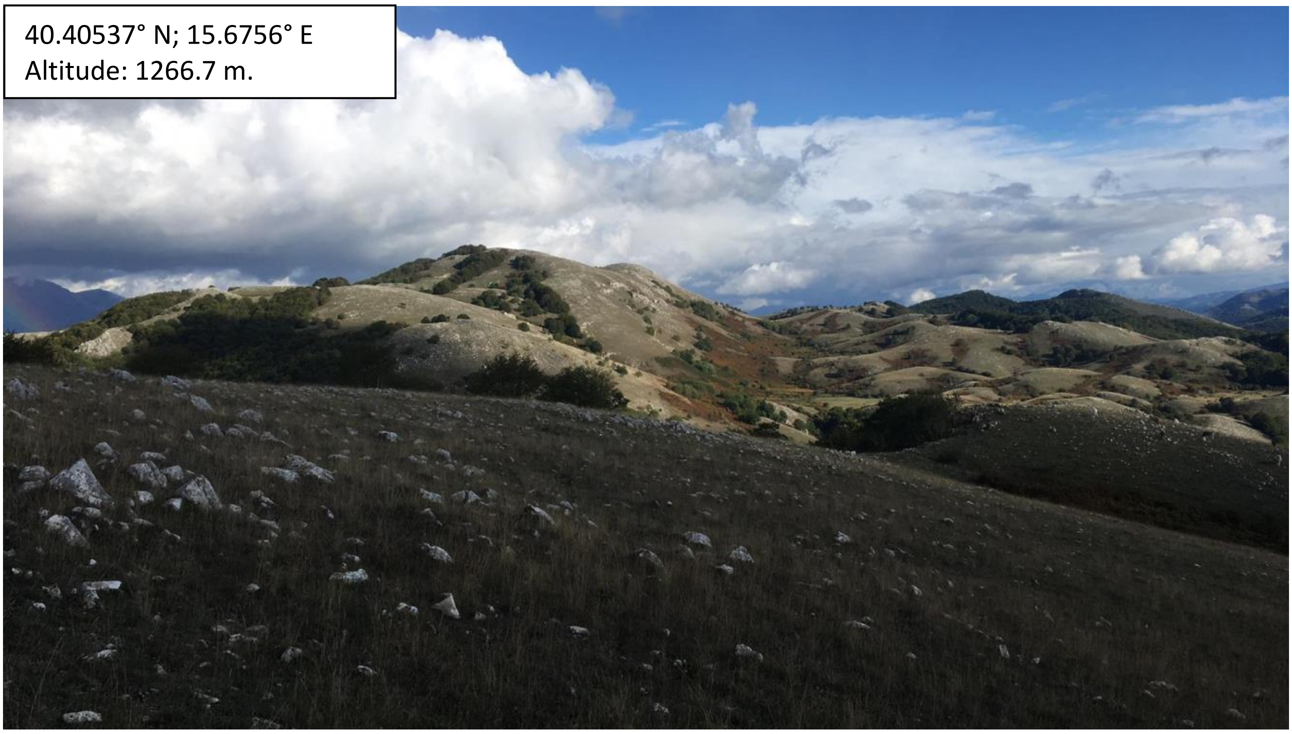

Figure 290.

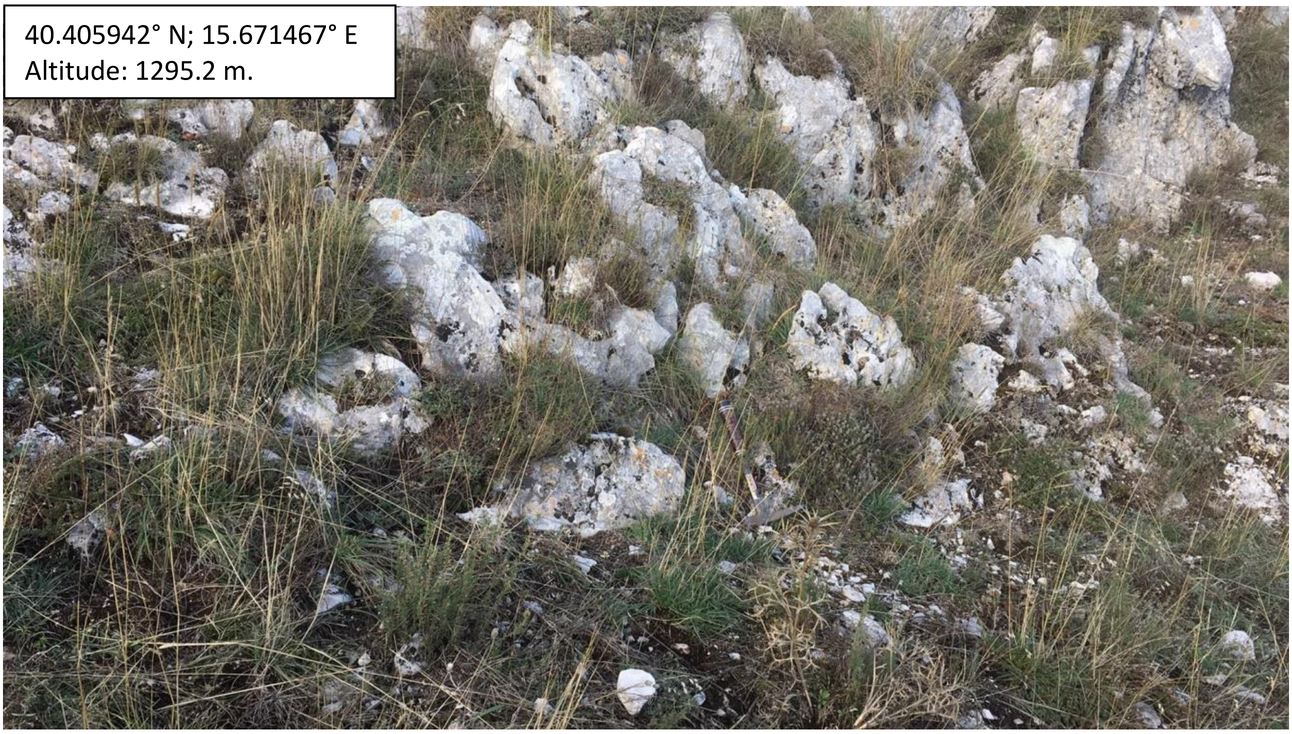

Figure 291.

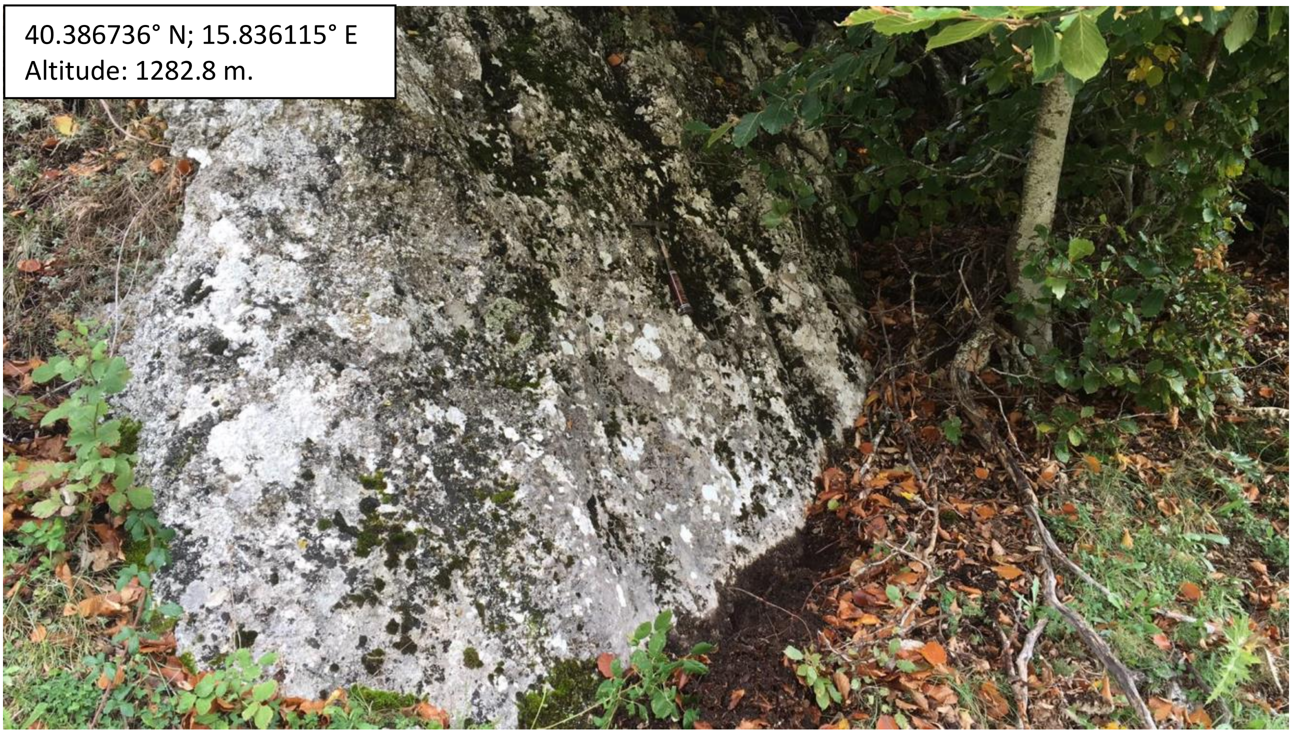

Figure 294.

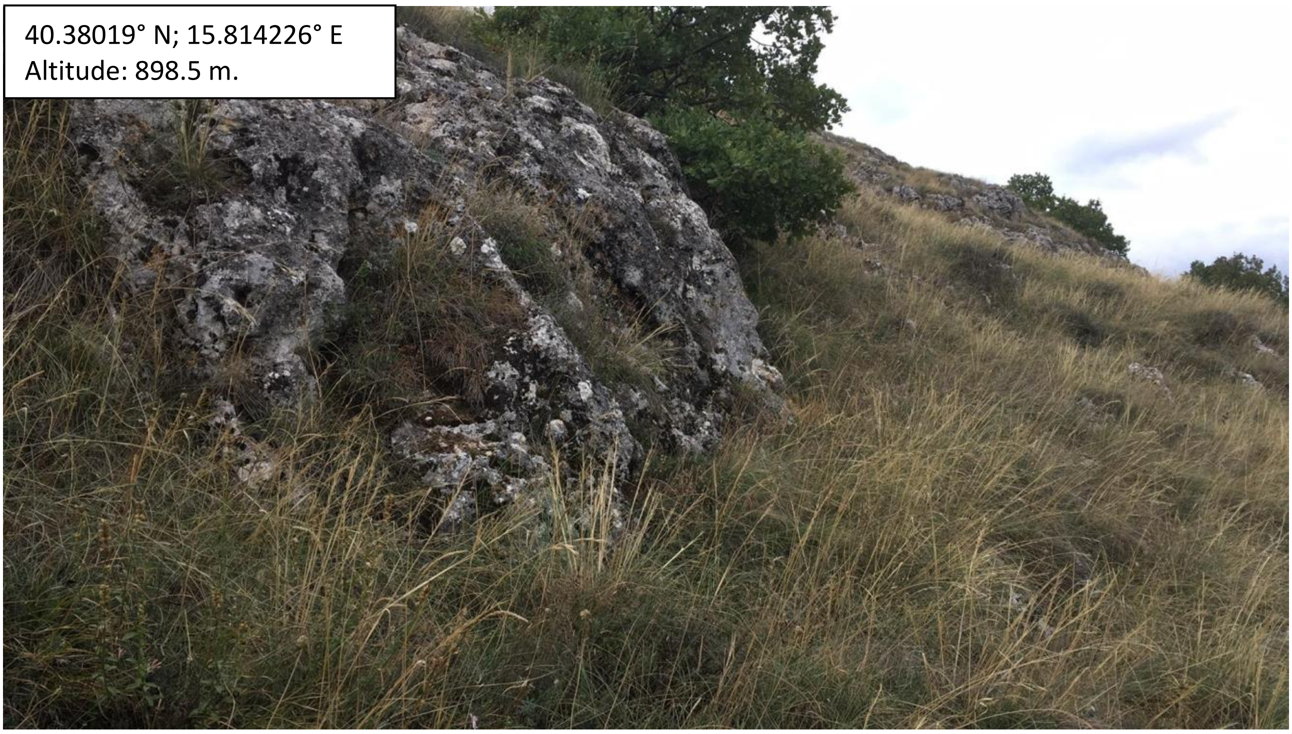

Figure 295.

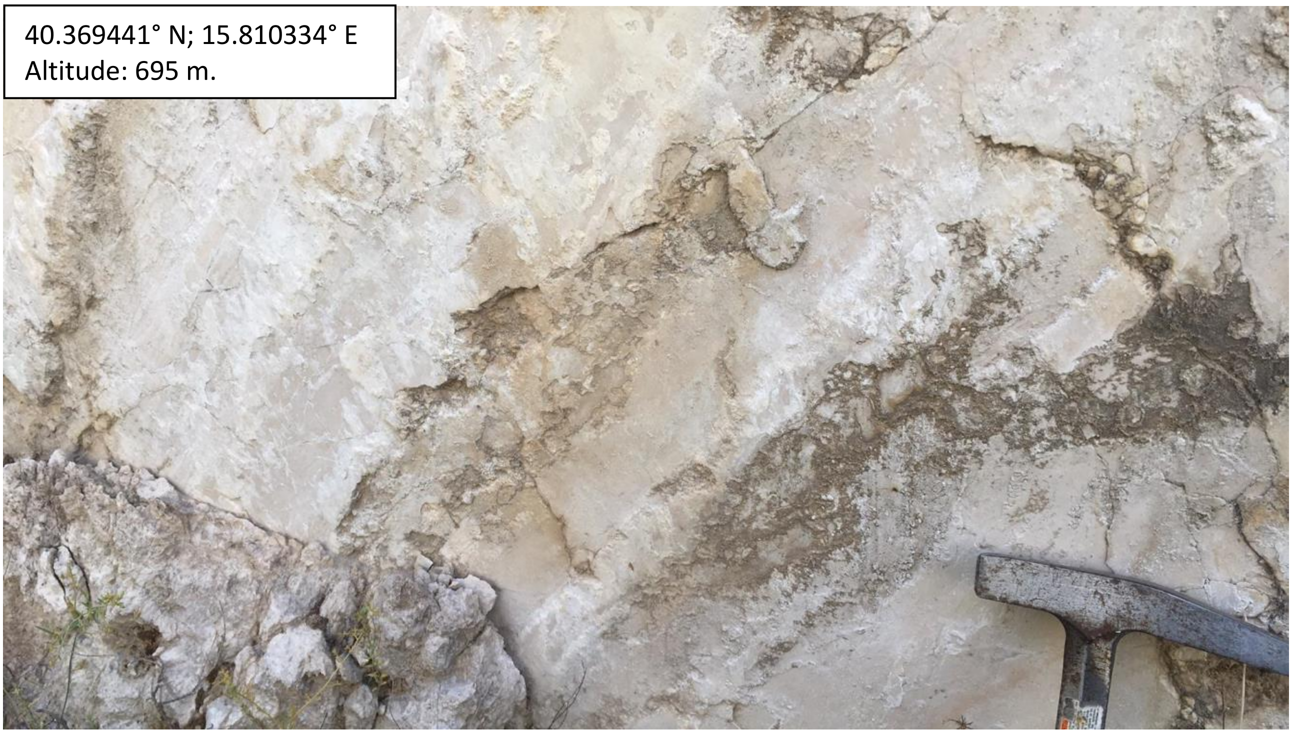

Figure 296.

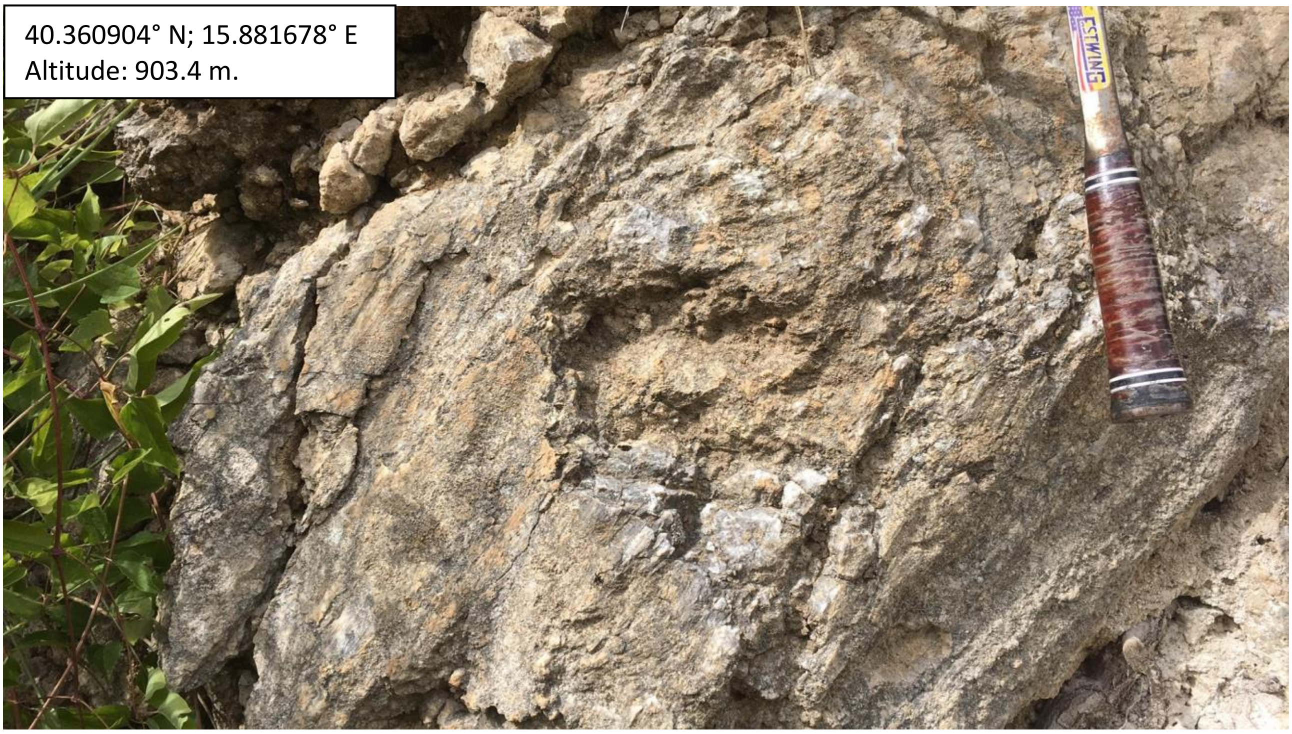

Figure 297.

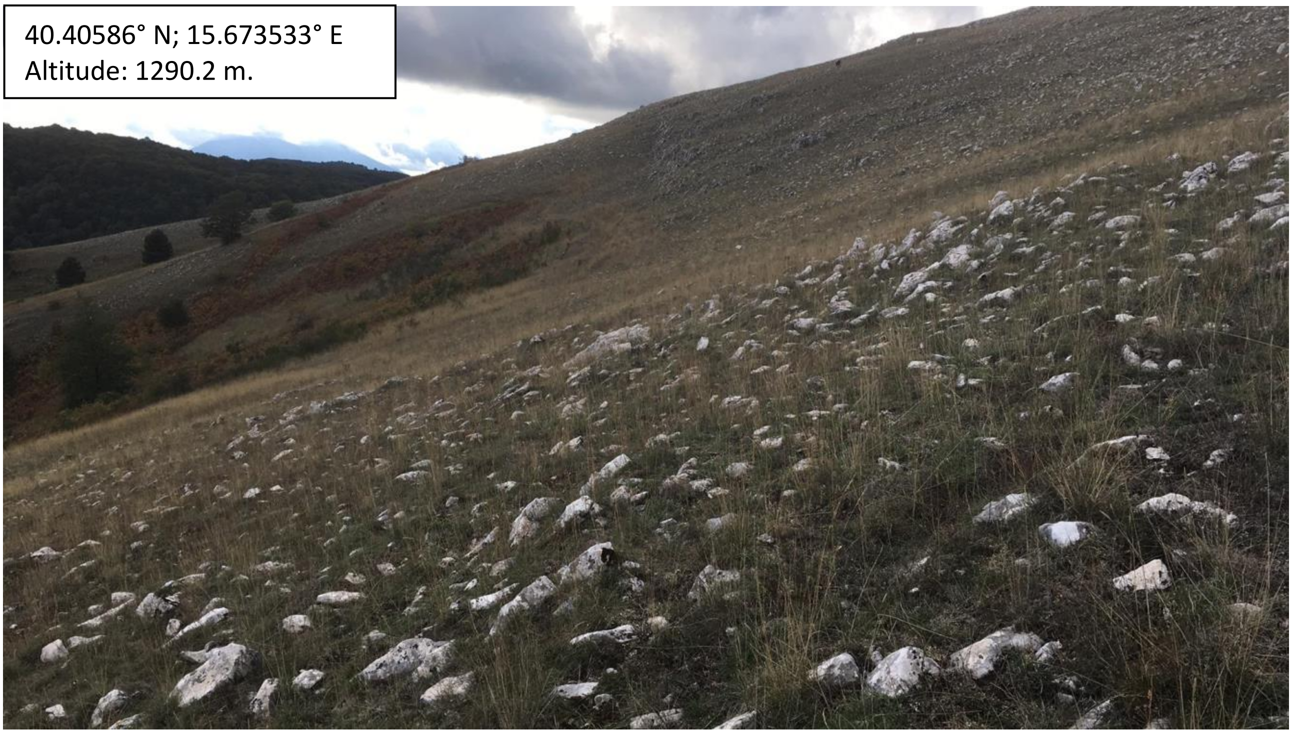

Figure 298.

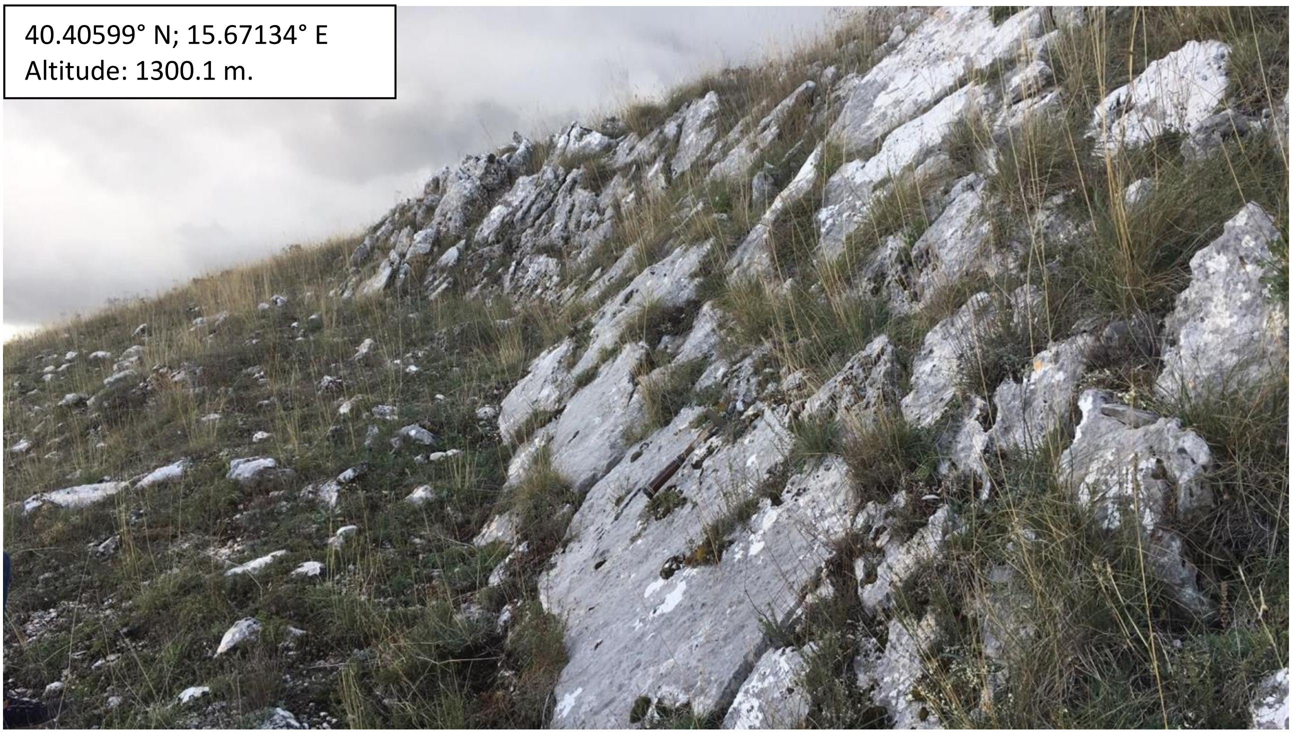

Figure 299.

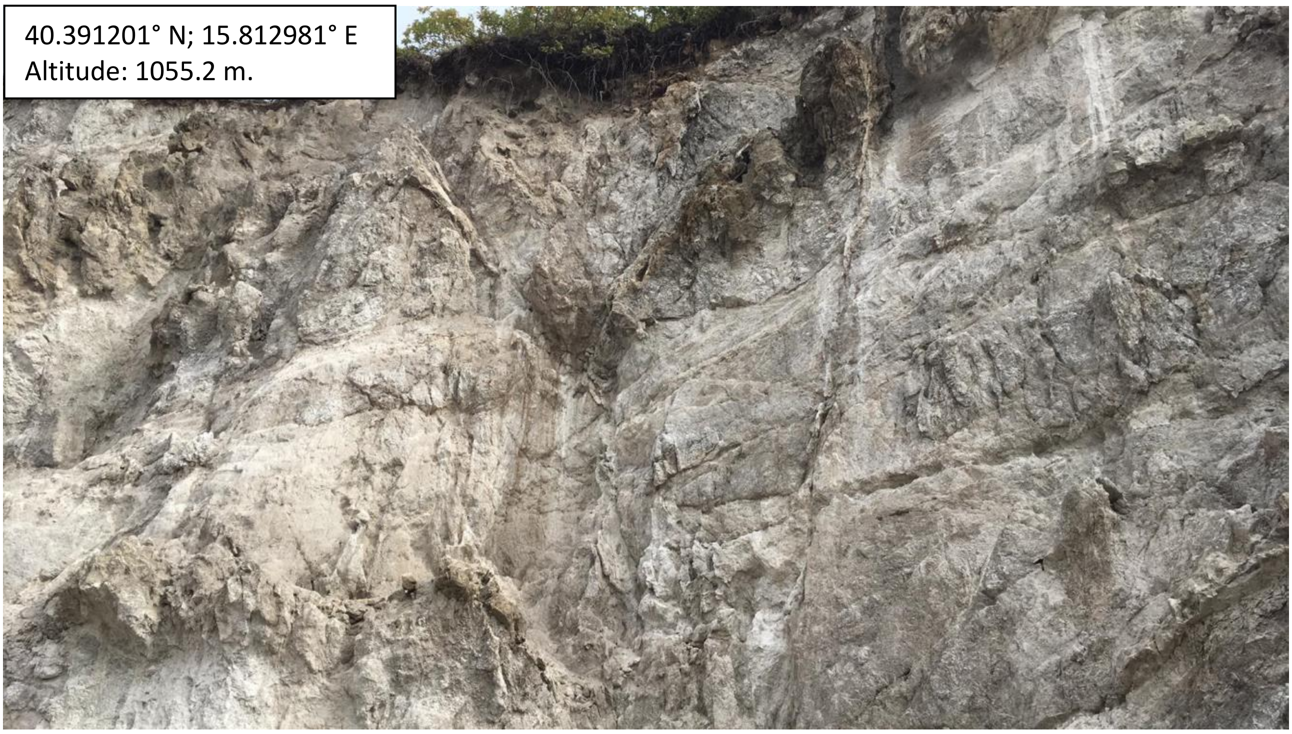

Figure 301.

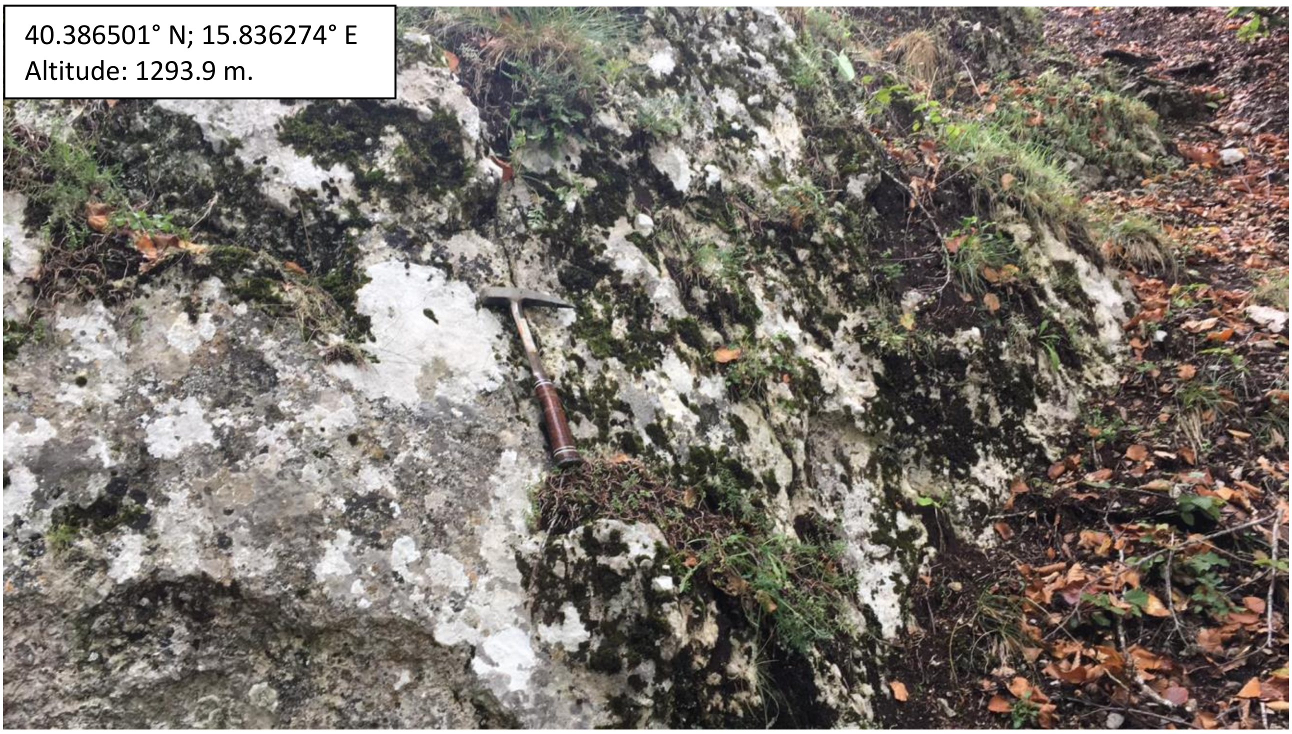

Figure 302.

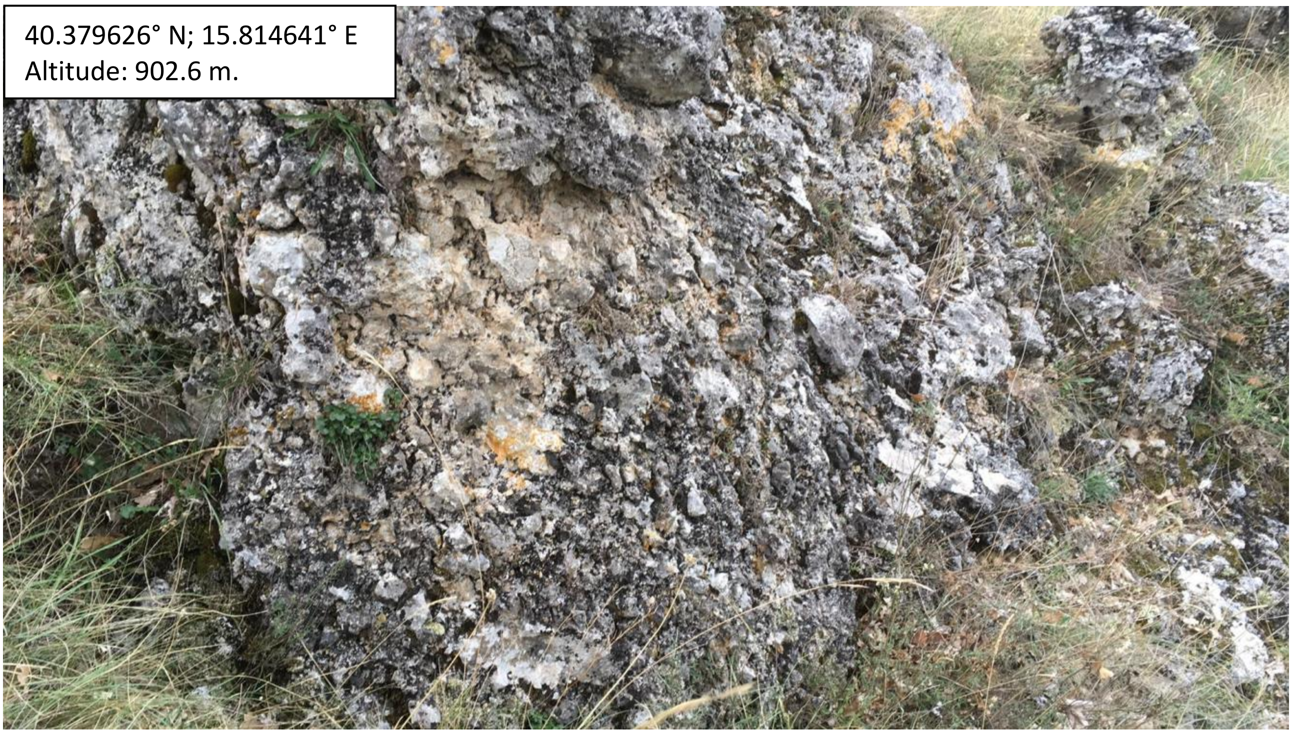

Figure 303.

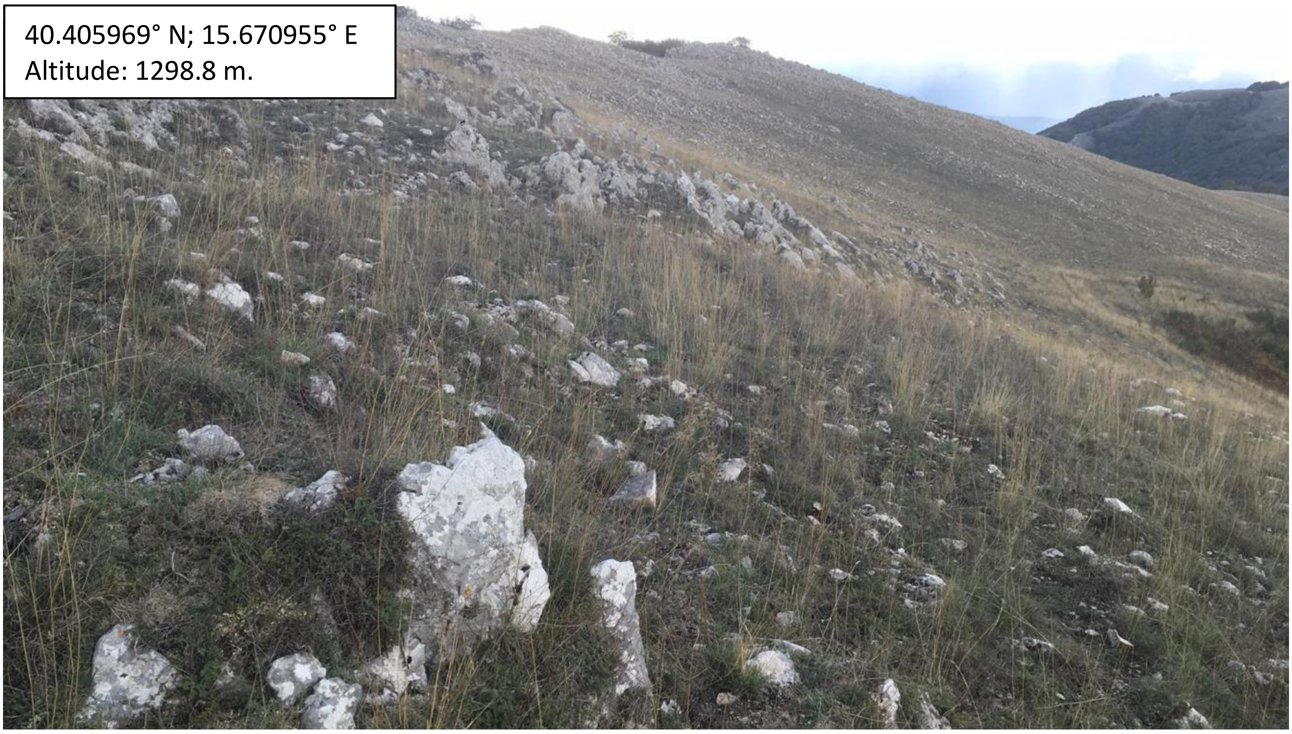

Figure 305.

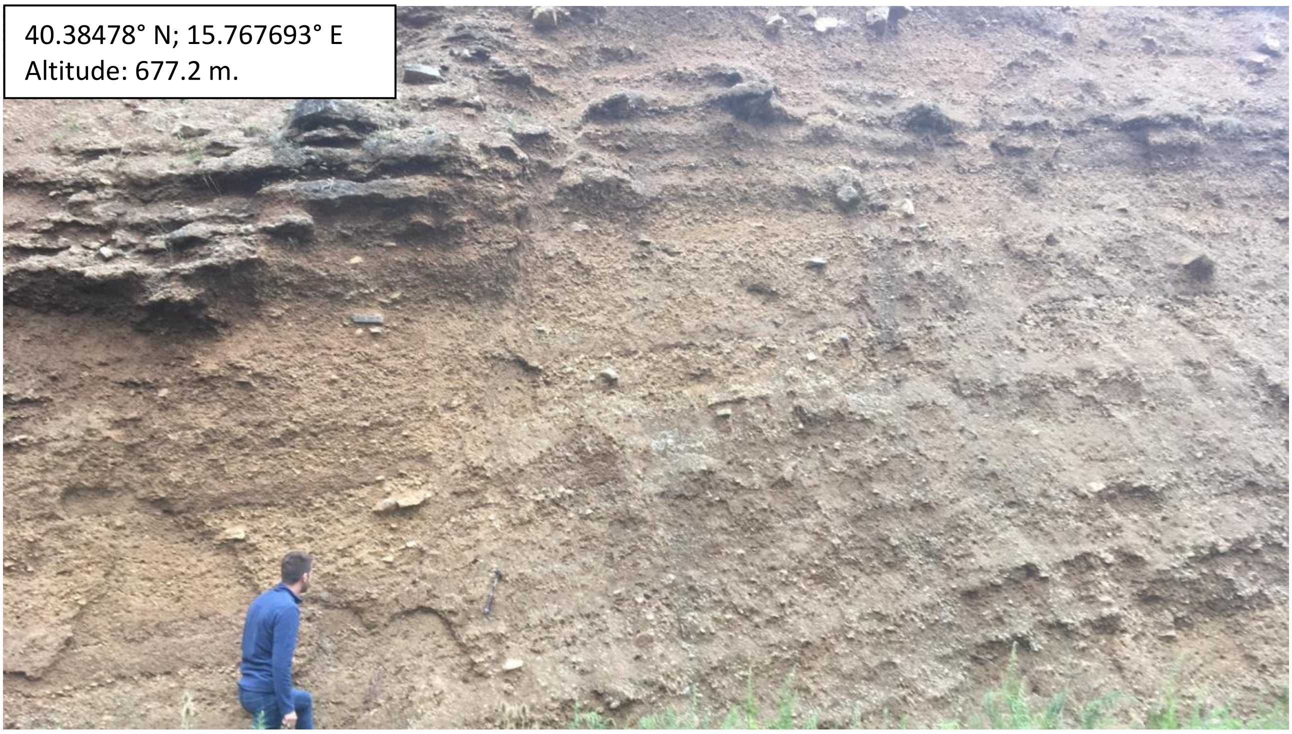

Figure 306.

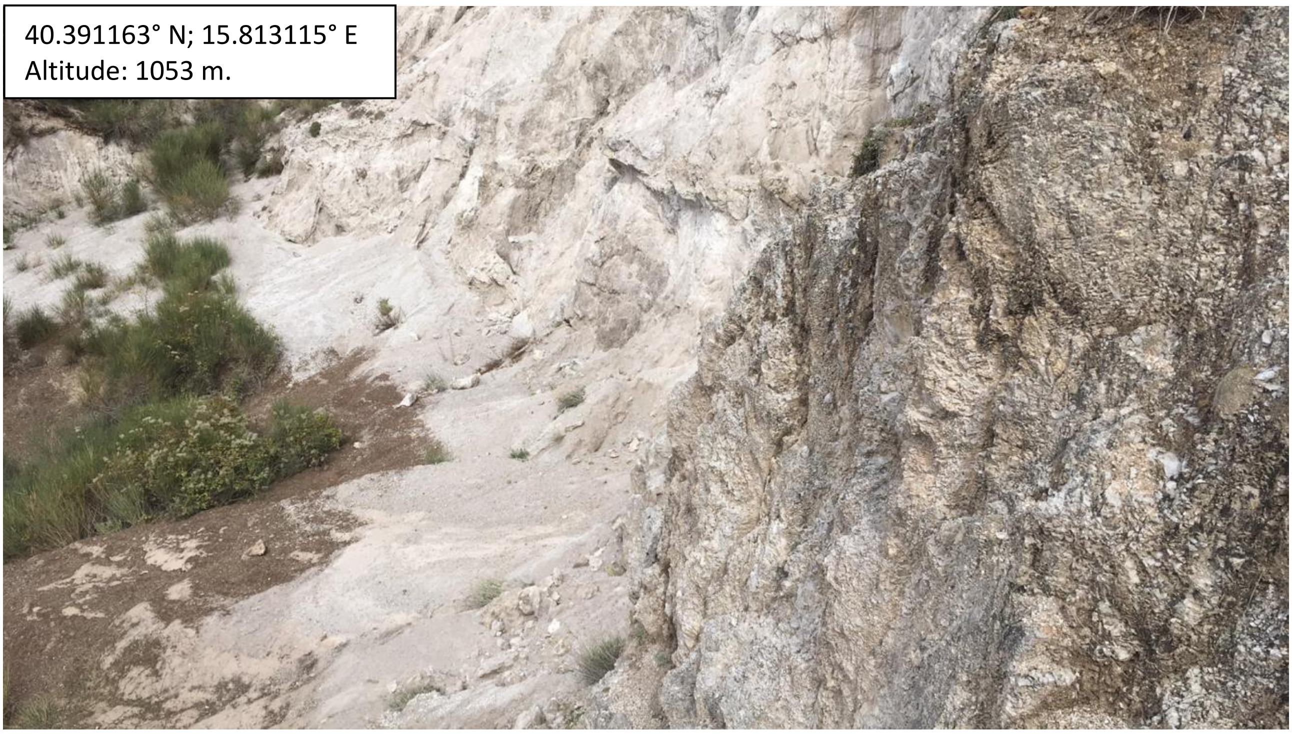

Figure 307.

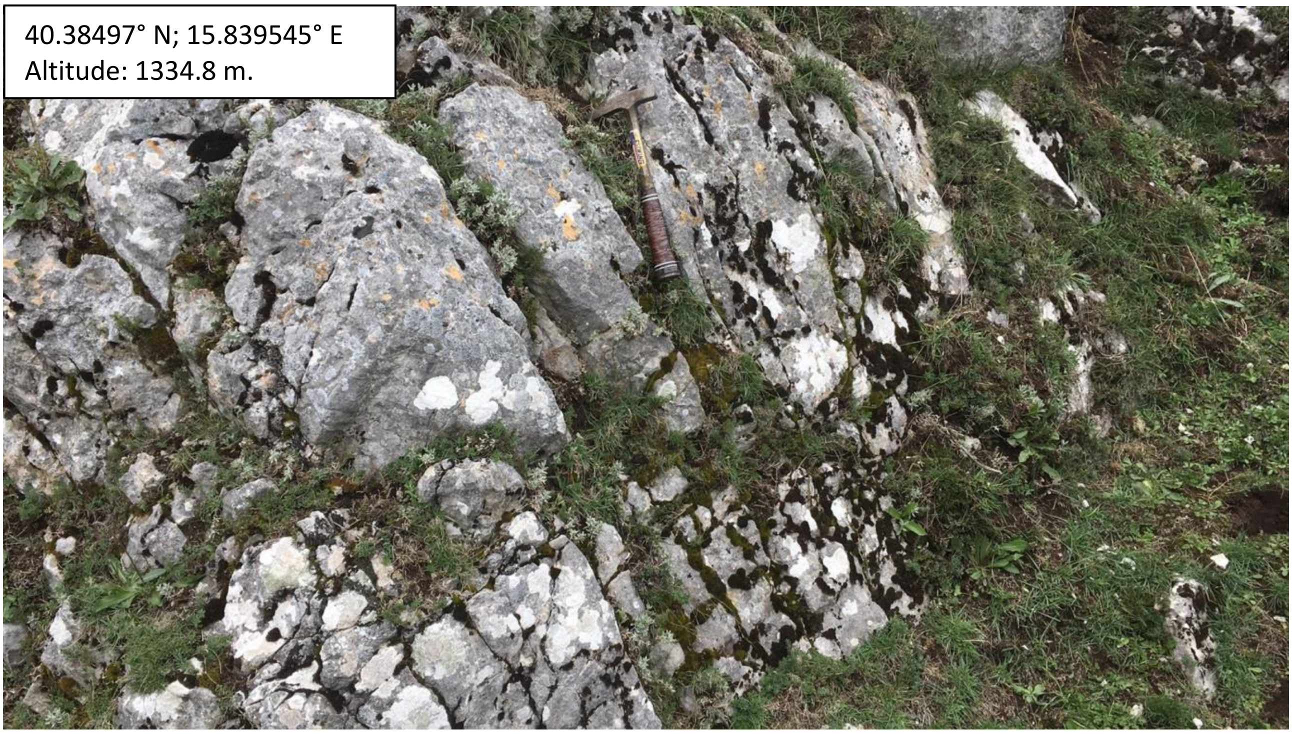

Figure 308.

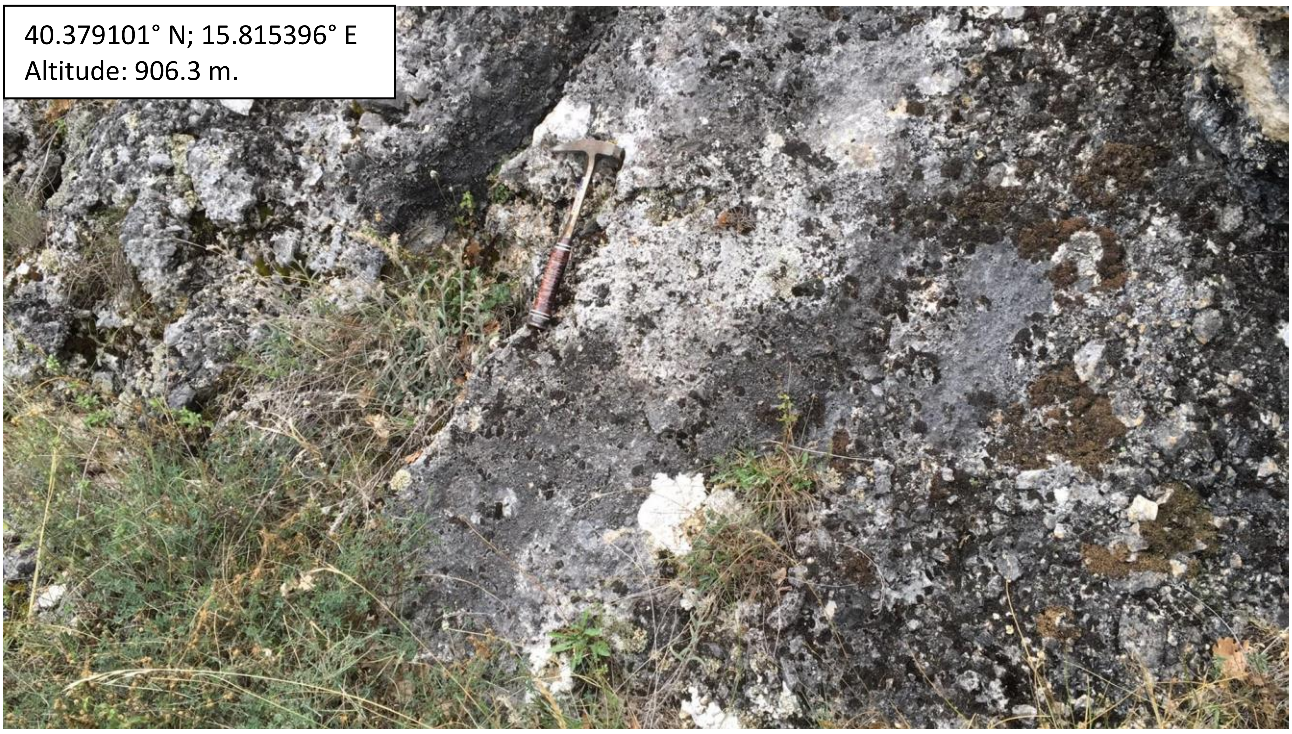

Figure 309.

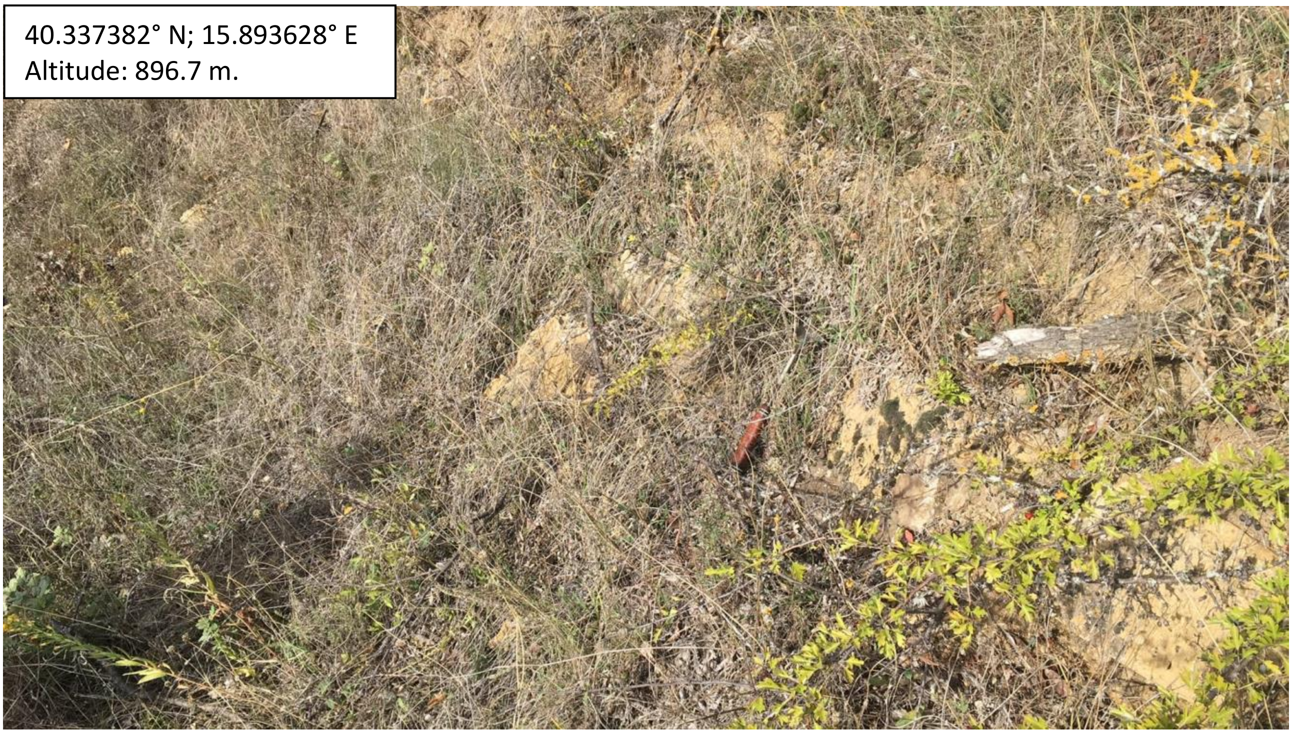

Figure 310.

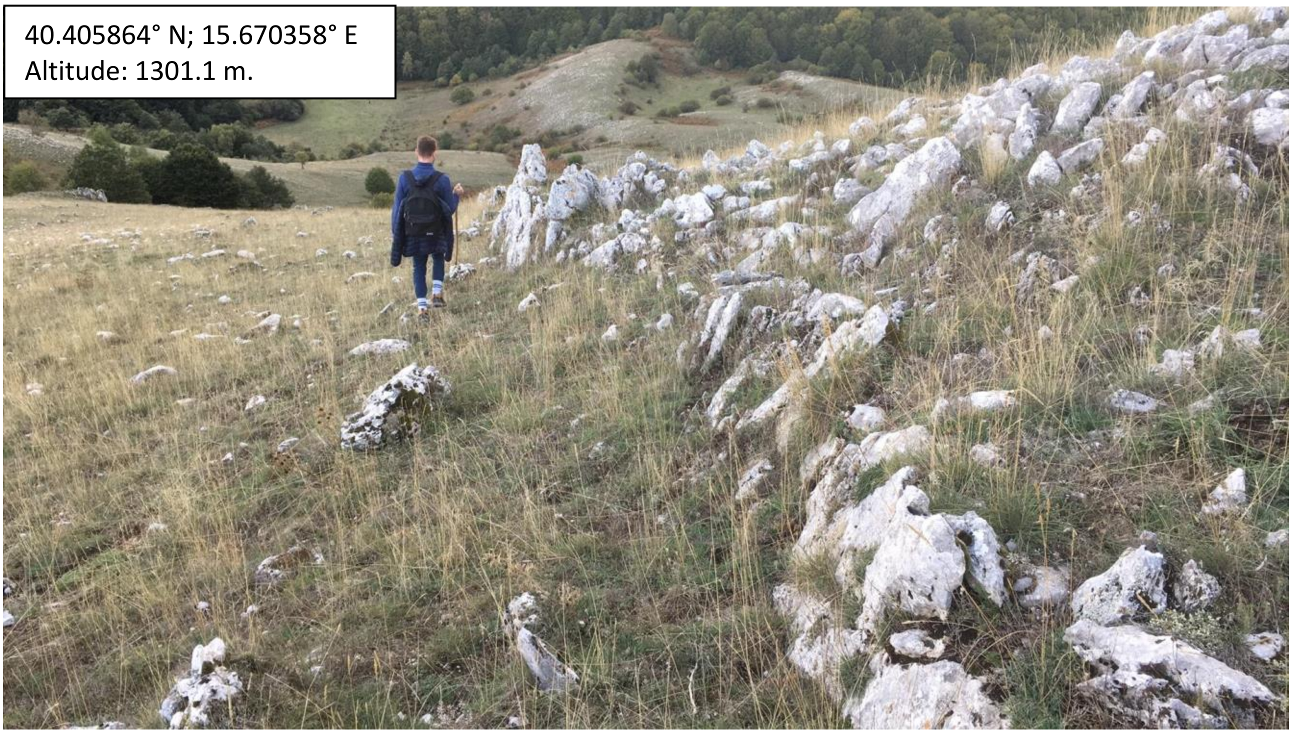

Figure 312.

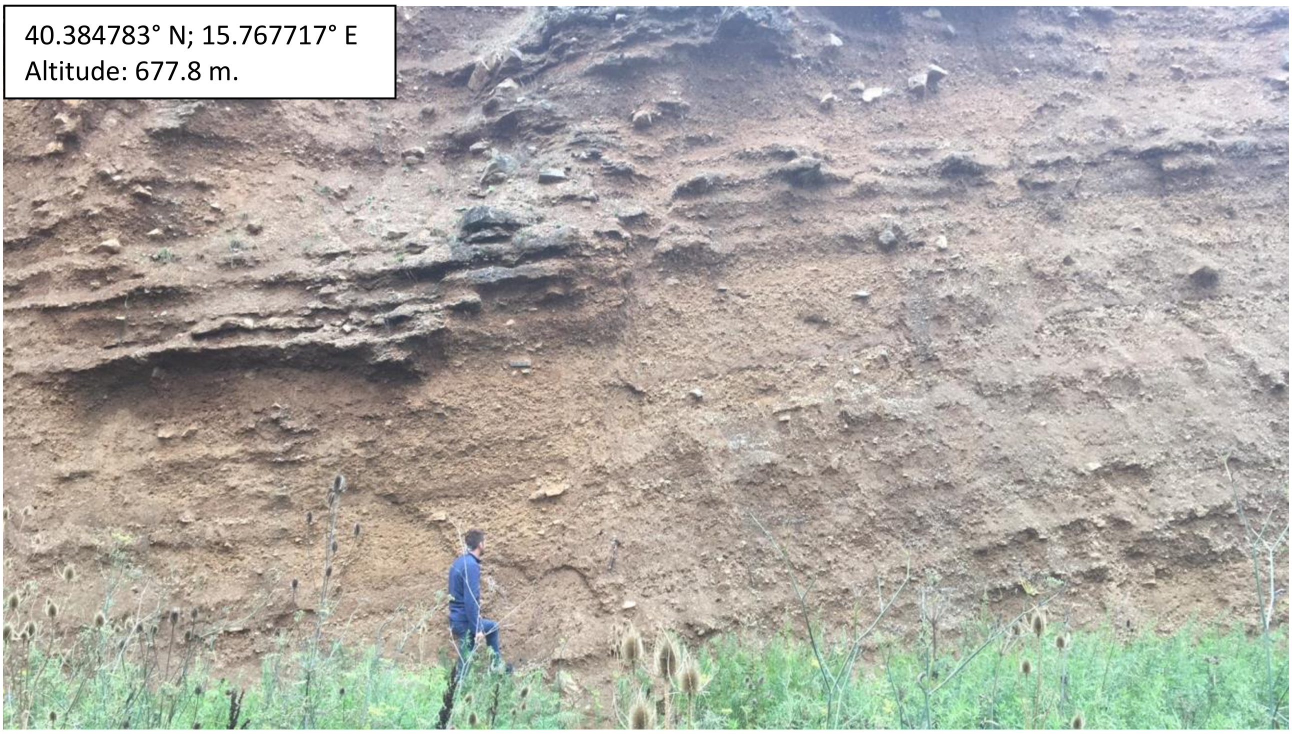

Figure 313.

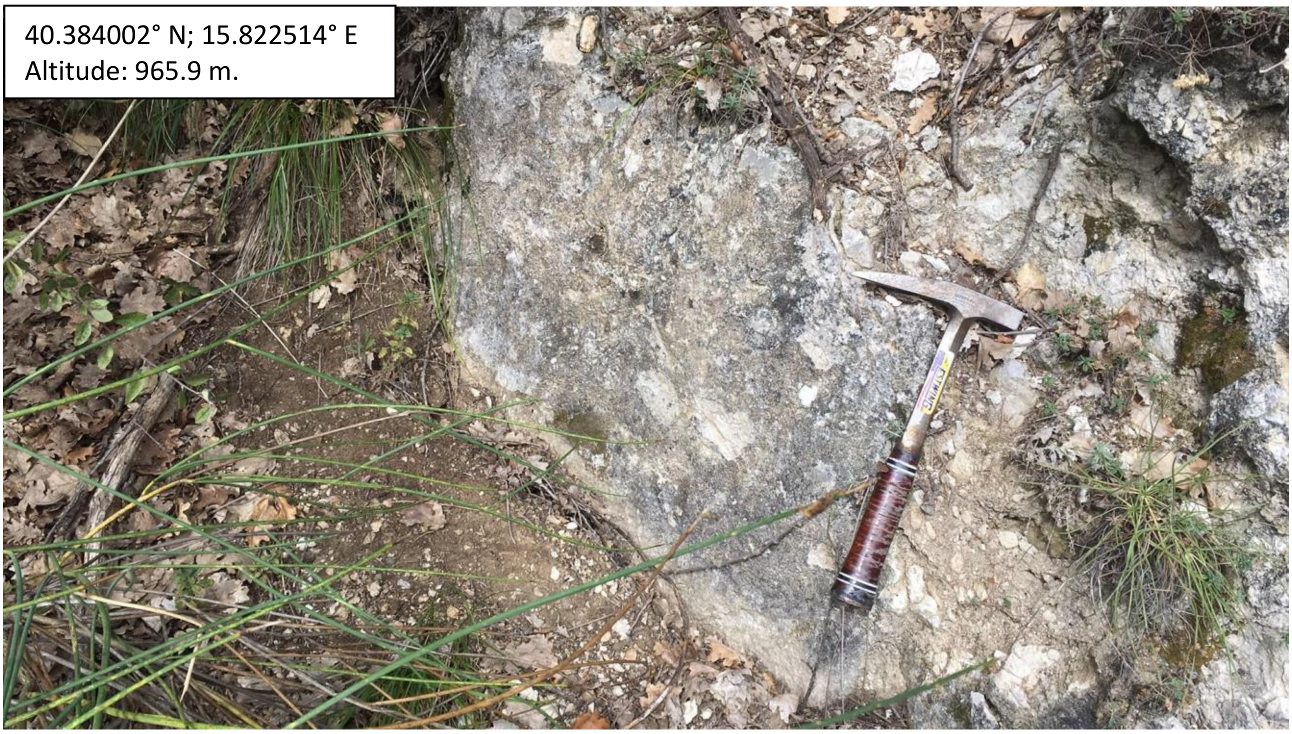

Figure 314.

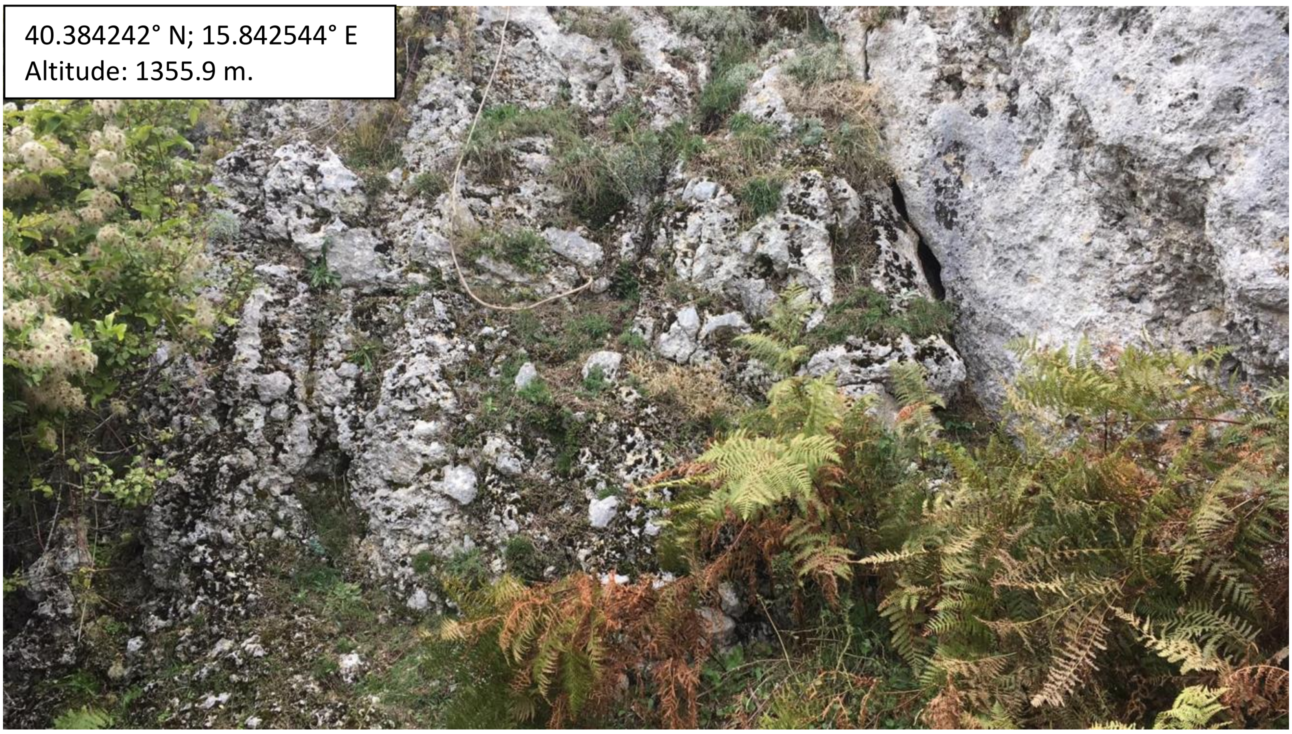

Figure 315.

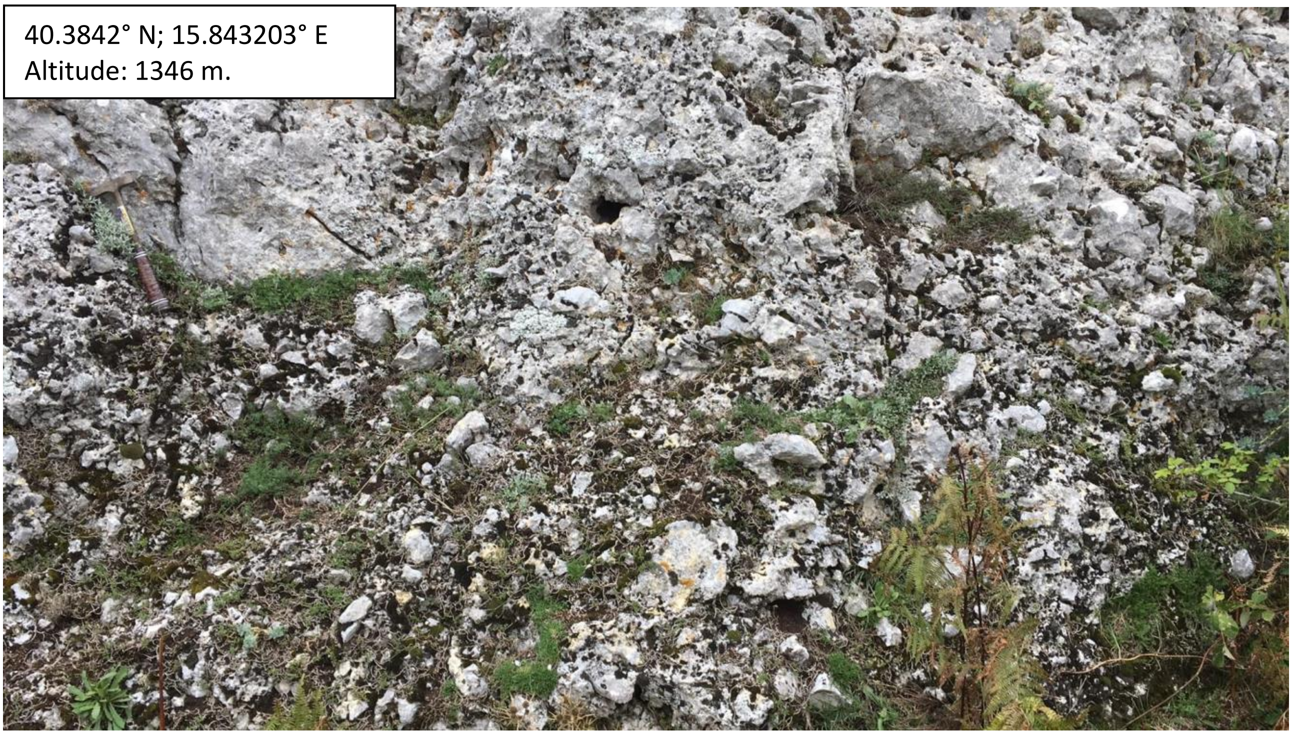

Figure 318.

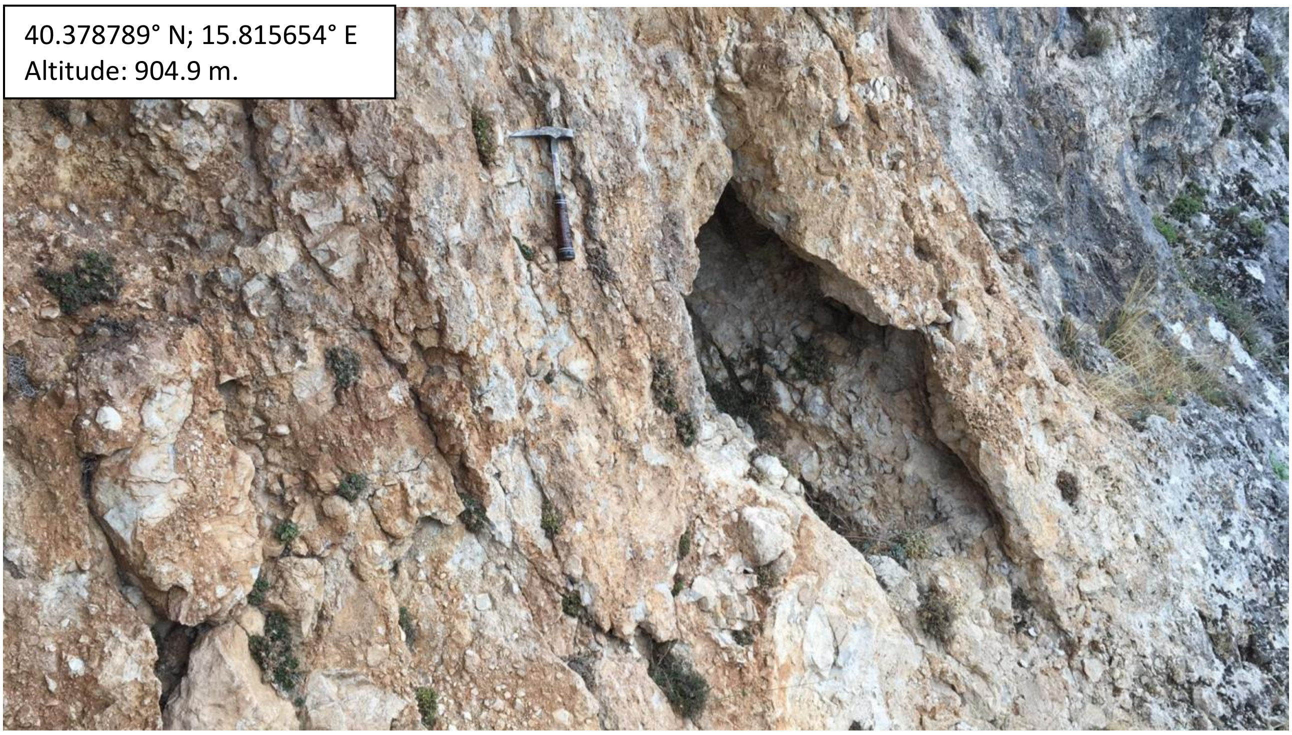

Figure 319.

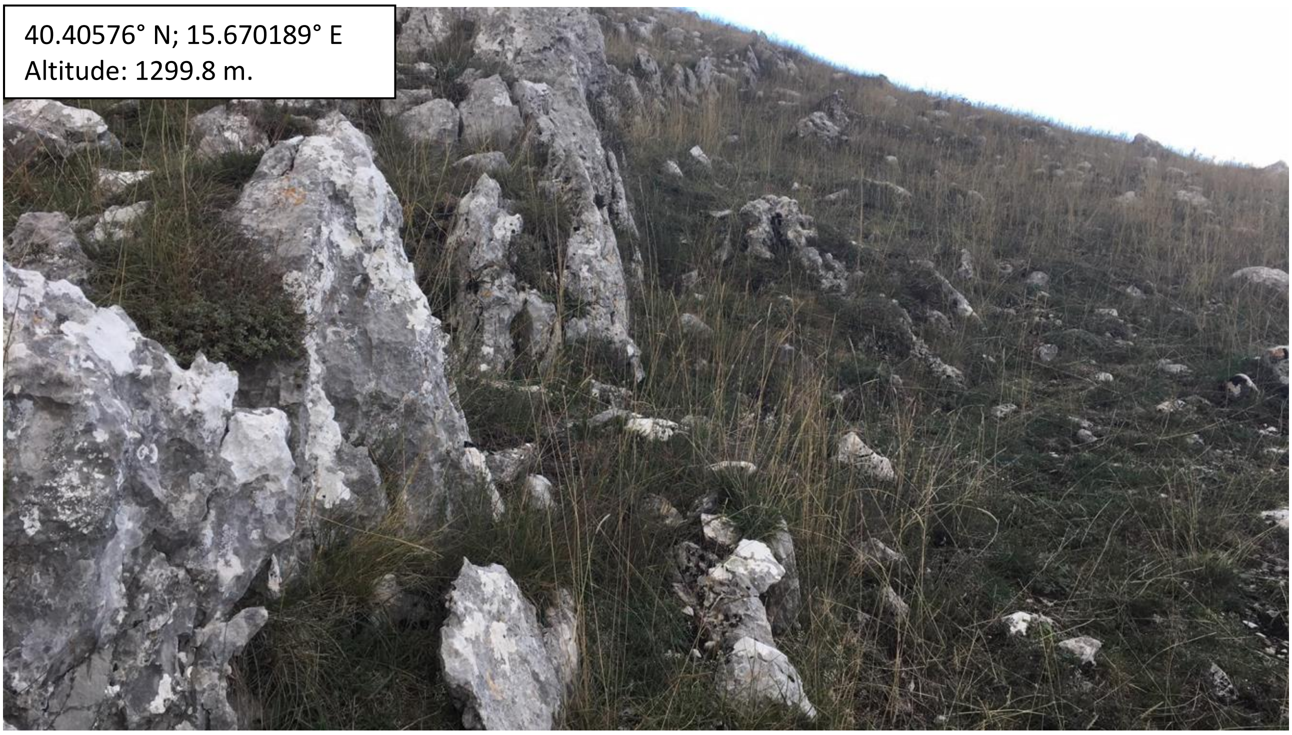

Figure 320.

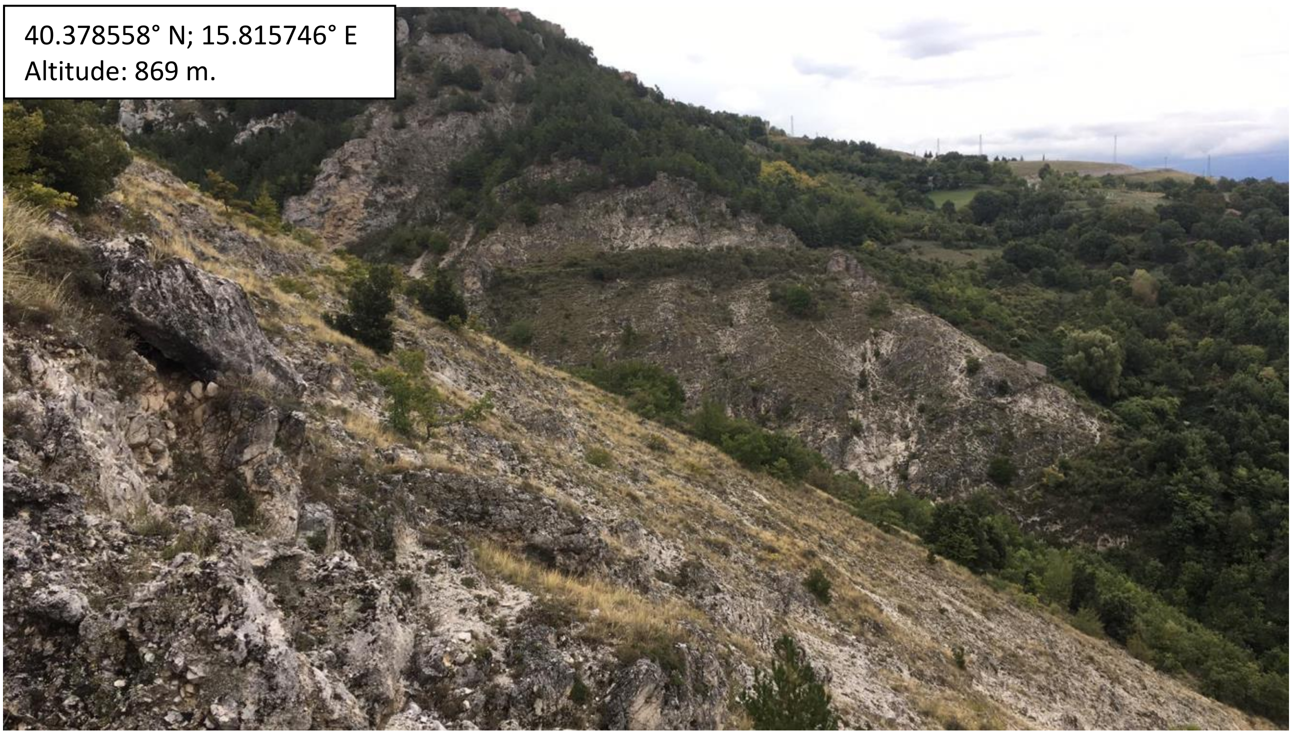

Figure 322.

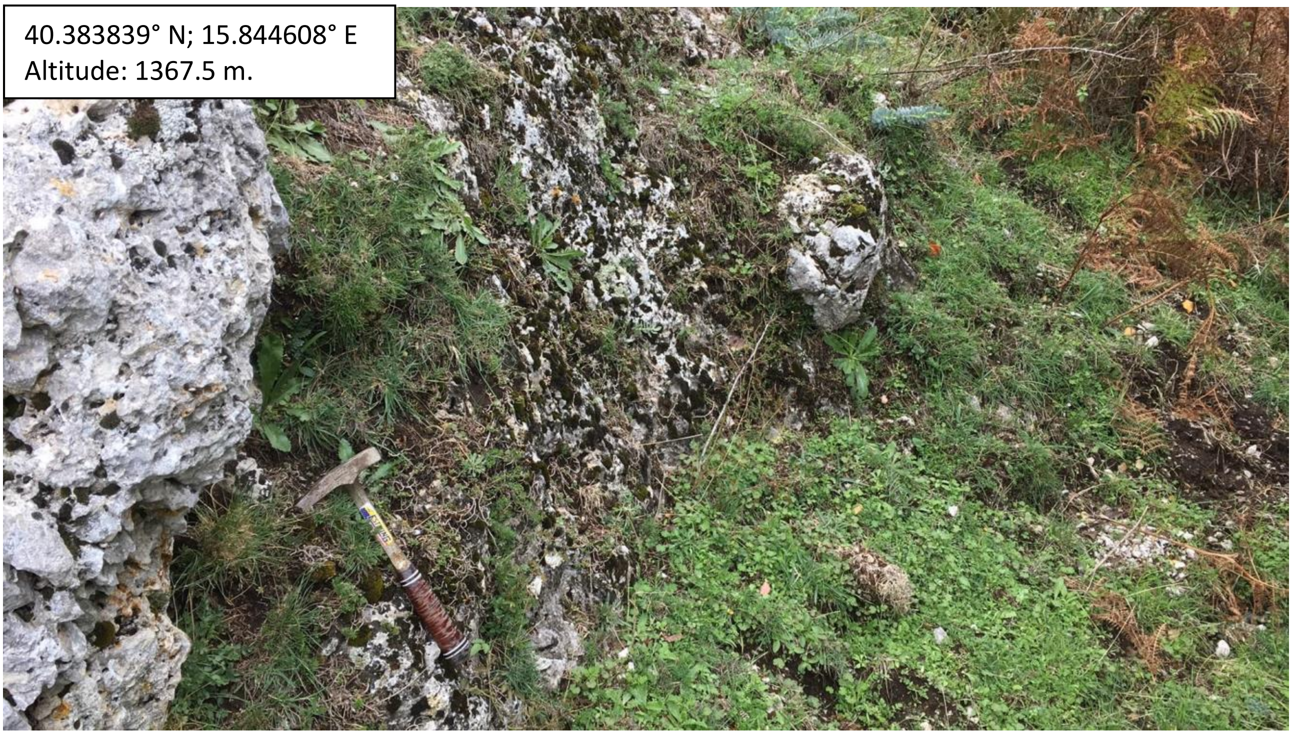

Figure 324.

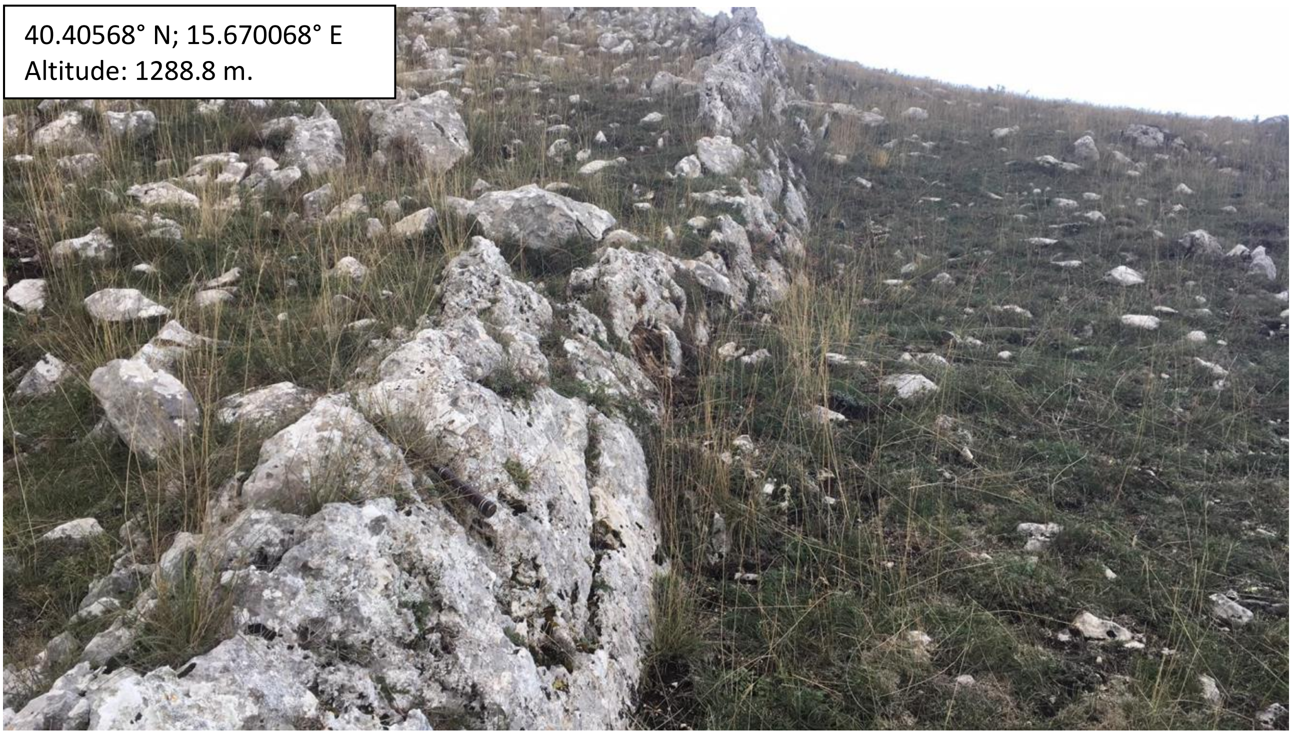

Figure 325.

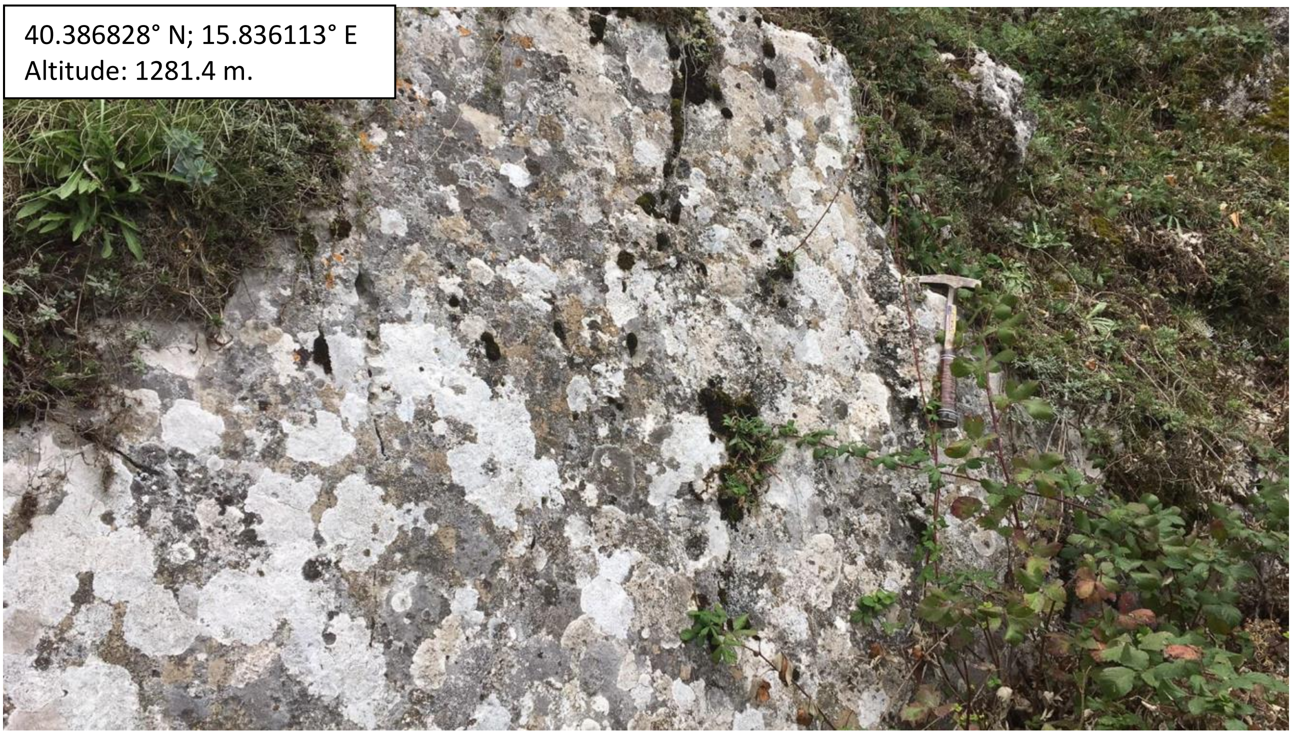

Figure 326.

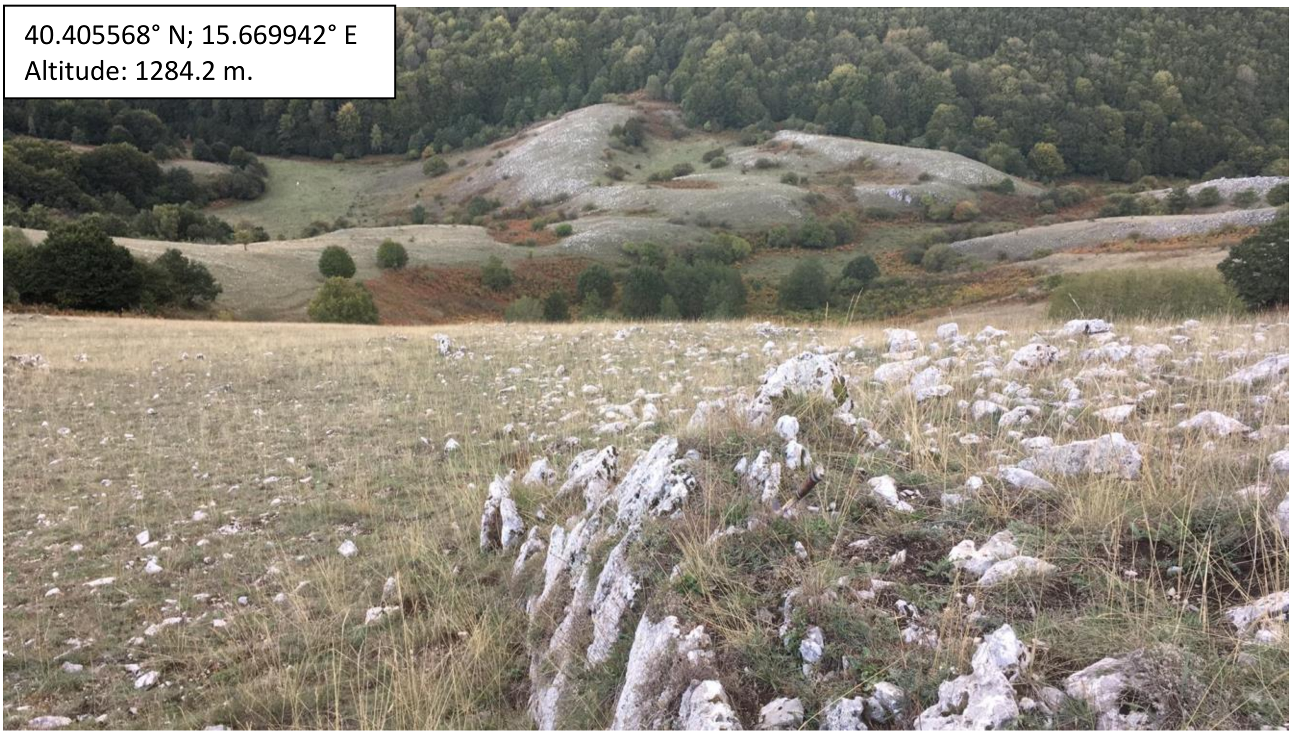

Figure 327.

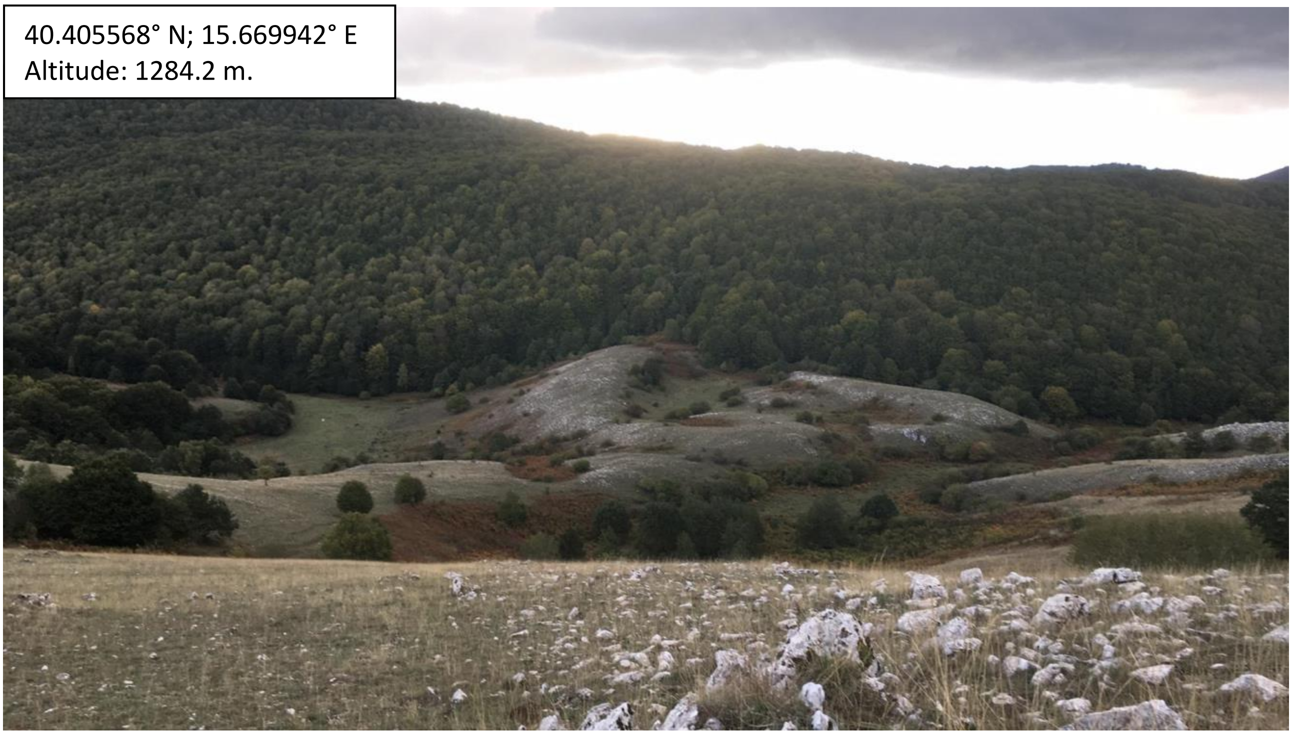

Figure 328.
